# Substituent Tightrope: Balancing Energy Storage and Stability of Dihydroazaborinine Molecular Solar Thermal Energy Storage Candidates

**DOI:** 10.1021/acs.joc.6c00925

**Published:** 2026-07-20

**Authors:** Sonja M. Biebl, Niklas Thiess, Kathrin Zwettler, Markus Ströbele, Holger F. Bettinger

**Affiliations:** † Institut für Organische Chemie, 9188 Eberhard Karls Universität Tübingen, Auf der Morgenstelle 18, 72076 Tübingen, Germany; ‡ Institut für Anorganische Chemie, Eberhard Karls Universität Tübingen, Auf der Morgenstelle 18, 72076 Tübingen, Germany

## Abstract

1,2-Dihydro-1,2-azaborinines (BN-benzenes) are promising candidates for molecular solar thermal energy storage (MOST) as substitution-dependent isomerization to either the Dewar BN-benzene or benzvalene isomers is possible. Since not only photoisomerization behavior but also key MOST parameters, such as the half-life of the photoisomers, the energy storage density, and the absorption wavelength, are strongly influenced by substitution, a systematic investigation of structure–property relationships is essential to accurately predict performance and design optimized storage systems. We report the synthesis of 1,2-disubstituted dihydroazaborinines varying steric demand at the 1- (nitrogen) and 2-position (boron) independently. Comprehensive investigations of their photoswitching behavior, half-life and stored energy of the metastable state demonstrated that a sterically demanding substituent at the boron atom is sufficient to stabilize the dihydroazaborinine itself under ambient conditions, while an additional bulky group at the nitrogen atom is required to suppress the spontaneous thermal back-reaction of the Dewar BN-benzene photoisomer at room temperature. Consequently, the steric demand of both substituents is crucial for achieving a promising MOST system.

## Graphical abstract

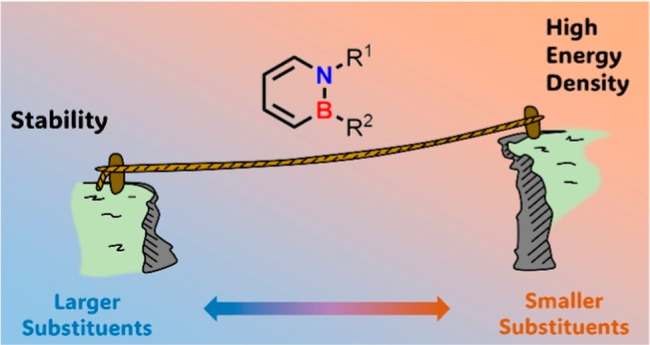

## Introduction

The targeted substitution of selected C=C units with isoelectronic and isosteric B–N motifs in π-conjugated organic systems, particularly in polycyclic aromatic hydrocarbons (PAHs), is an established strategy for the development of novel organic–inorganic hybrid materials.^1–3^ This approach enables controlled redistribution of electron density while preserving the geometry of the carbon analogue, resulting in diversely modified reactivity patterns and physicochemical properties (Scheme 1a).^4–6^ Due to the complementary σ- and π-donating effects between nitrogen and boron, isomers with adjacent B–N substitution represent the thermodynamically most stable constitution.^7^

**Scheme 1:**
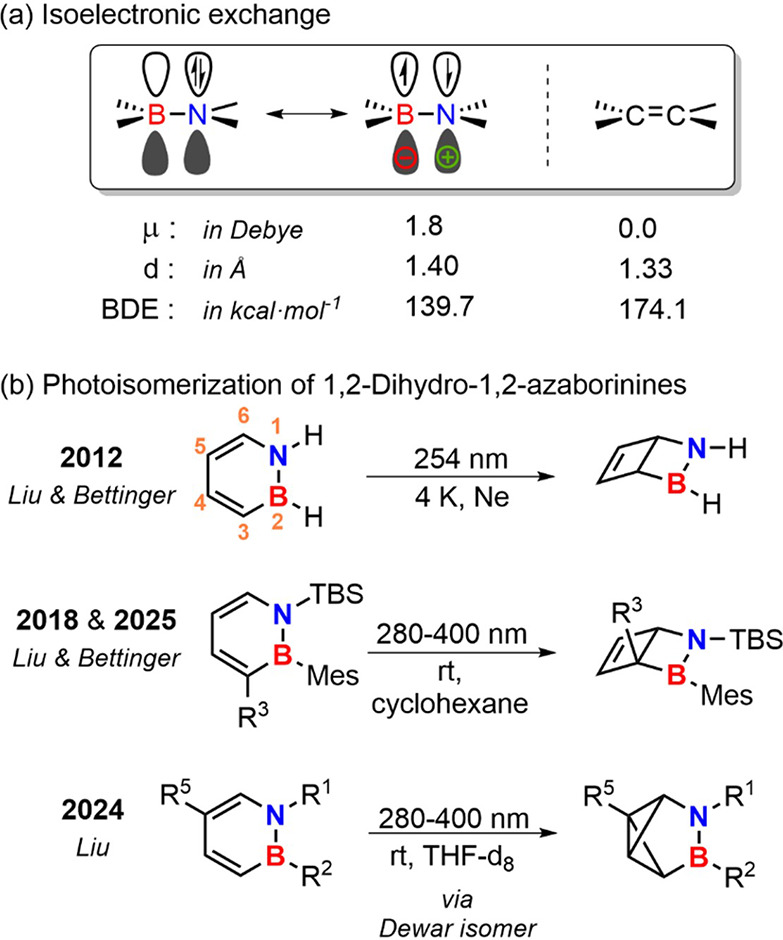
Comparison of the Ethene Fragment with Its BN Analogue^7–10^ (a) and Overview of the Substitution-Dependent Photoisomerization Behavior of ^***BN***^ **Bs** (b)

Even for one of the simplest examples, 1,2-dihydro-1,2-azaborinine (^***BN***^ **B**) remarkable photo reactivity is evident.^2,11,12^ In contrast to its carbon analogue, benzene, the heterocycle undergoes quantitative and exclusive isomerization to the corresponding Dewar isomer (^***BN***^ **D**) upon UV irradiation.^13–100^ While the parent Dewar isomer (^***BN***^ **D**) required stabilization under cryogenic matrix isolation conditions,^13^ the kinetic stabilization (^***BN***^ **B1**) reported by Liu, Bettinger et al. in 2018 enabled a prolonged lifetime of the metastable species.^14^ Among other potential applications, the system therefore offers use in energy storage, as a so-called molecular solar thermal (MOST) energy storage system (Scheme 1b).^14,15^ MOST compounds rearrange to a higher-energy metastable species upon energy input, usually in the form of electromagnetic radiation.^17–22^ Parts of the absorbed energy can thus be stored in the photo isomer and selectively released on demand by disposing a suitable external trigger.^23,24^ For ^***BN***^ **D1**, both thermal^14–16^ as well as homo- ^14,25^ and heterogeneous^26^ catalyzed back-reactions are possible. Thus, the ^***BN***^ **B**/^***BN***^ **D** system represents an integrated energy storage solution, combining energy conversion, storage, and release, that already meets a broad range of requirements for a MOST system (Figure 1).^14,15^

**Figure 1:**
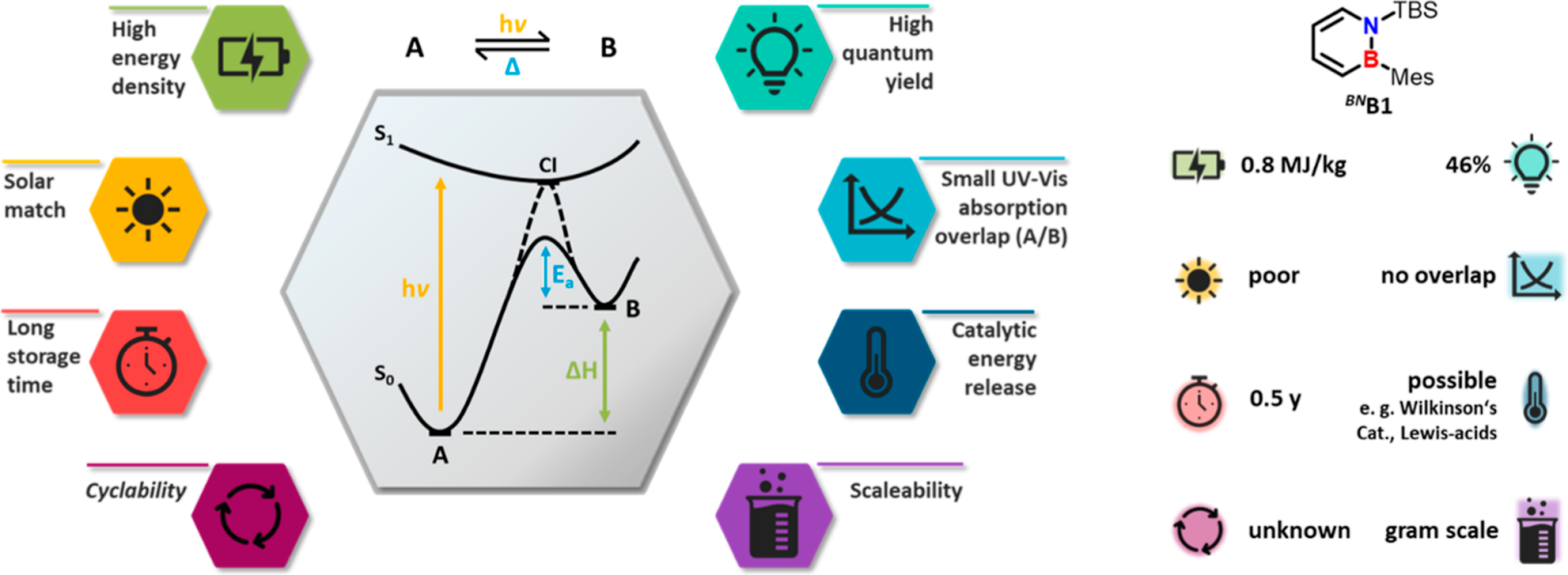
Overview of the operating principle and key parameters of molecular solar thermal (MOST) systems, as well as specific data for ^***BN***^ **B1**.^14^

To enable a targeted application of dihydroazaborinines, insights into the structure–property interdependence of photo isomerization and thermal ring-opening processes are essential. Through rational substitution of the system, various parameters of the central photoreaction can be tuned. For instance, linking multiple ^***BN***^ **B** units within a single molecule results in an additive increase of the stored energy,^15,100^ whereas substitution at C3 of the carbon backbone alters the half-life of the corresponding ^***BN***^ **D** isomers.^16^ Recently, Liu et al. further demonstrated that the presence of a substituent at C5 of the carbon backbone fundamentally changes the photoisomerization by enabling a subsequent reaction of ^***BN***^ **D** to a BN-benzvalene isomer.^27^ Such effects, however, are difficult to predict,^28^ making further systematic investigation of the influence of individual substituents on photoisomerization necessary in order to identify trends and apply them rationally.

Owing to their enhanced reactivity compared to the carbon backbone, the vast majority of late-stage functionalization protocols for ^***BN***^ **B** derivatives remain focused on the substitution of the heteroatoms. Following their electronic properties, the boron center can undergo reactions with C-,^14,29–32^ N-,^33^ O-,^34^ and S-nucleophiles,^33^ while the Lewis-basic nitrogen site typically engages in reactions with electrophiles after deprotonation.^35,36^ In addition, a broad range of aryl and alkenyl groups can be introduced through Stille^37^ and Heck^38^ coupling on boron or Buchwald–Hartwig^35^ coupling reactions on nitrogen.

As no investigation of the influence of these substitution patterns on the key MOST properties of ^***BN***^ **B** has been reported so far, this study aims at providing a first insight into the steric and electronic influence of substitution at the two heteroatoms on the isomerization processes. The starting point of these investigations was the kinetically stabilized dihydroazaborinine ^***BN***^ **B1**.^14^ In order to determine the extent to which the individual substituents at boron and nitrogen contribute to the stability of ^***BN***^ **B1**, or whether a synergistic effect is involved, the steric demand of the substituents at boron and nitrogen was varied independently. Additionally, electron donating (OMe) or withdrawing (CN) substituents were introduced at the aryl substituent on boron, while the established TBS substituent on nitrogen is preserved.

## Results and Discussion

### Synthesis

All compounds investigated were synthesized starting from dihydroazaborinine ^***BN***^ **B2** ^25,39^ (Scheme 2) based on literature-established protocols for the synthesis of ^***BN***^ **B1**,^14,25^ ^***BN***^ **B8** ^35,40^ and ^***BN***^ **B10**.^41^ In the course of a nucleophilic substitution reaction a substituent was first introduced at the boron atom, employing organolithium reagents (^***BN***^ **B1**, ^***BN***^ **B3–** ^***BN***^ **B7**). This step enabled purification of all compounds under consideration *via* column chromatography and allowed for (short-term) handling in air. If required, the TBS protecting group at the nitrogen atom was subsequently cleaved using tetrabutylammonium fluoride (^***BN***^ **B8**–^***BN***^ **B9**),^35,40^ and a new substituent was introduced through a sequence of deprotonation and reaction with an electrophile (^***BN***^ **B10**, ^***BN***^ **B11**, ^***BN***^ **B13**).^35^ Compound ^***BN***^ **B12** was synthesized and investigated earlier by Richter et al.^25^

**Scheme 2:**
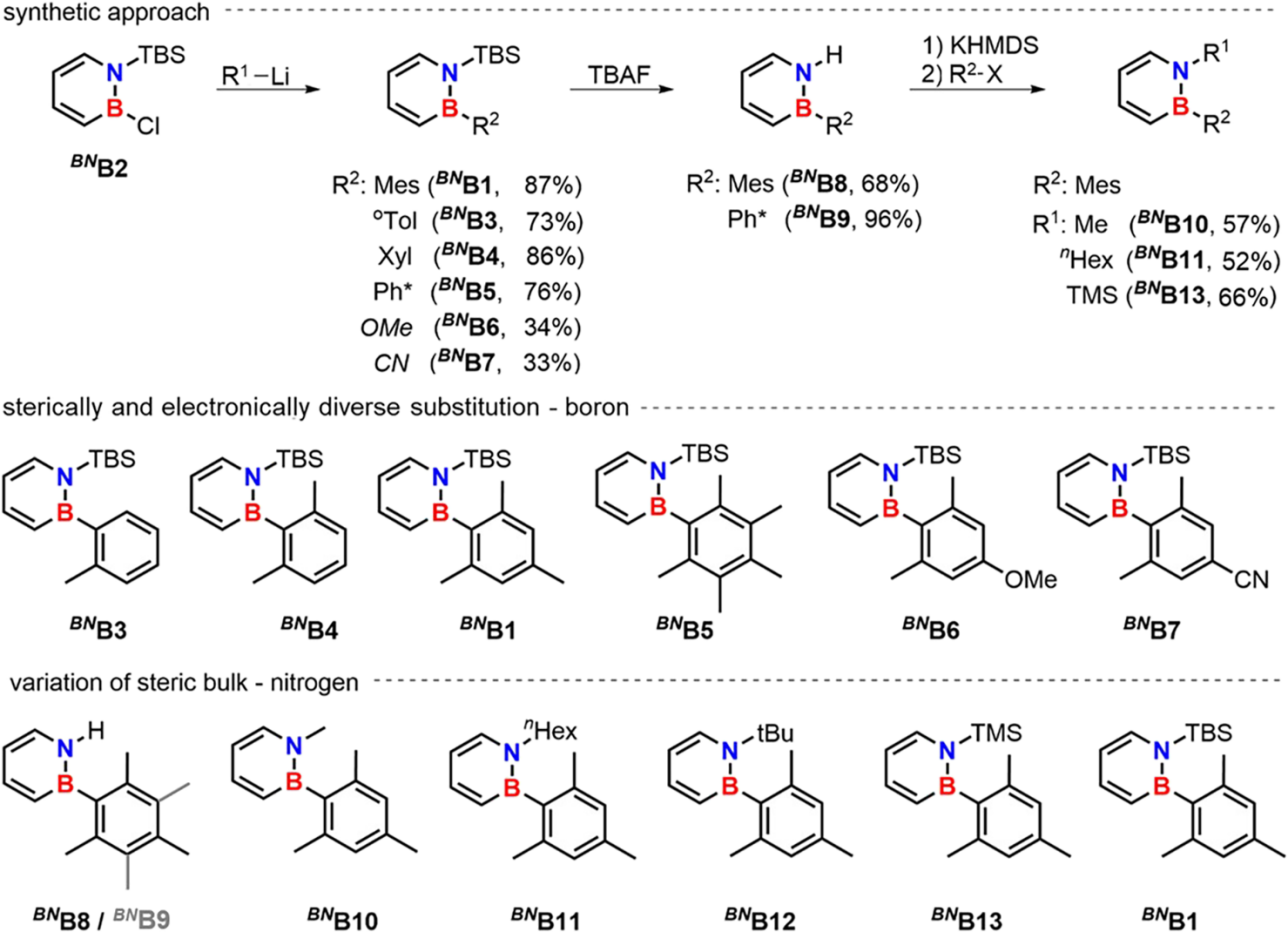
General Synthesis of the 1,2-Substituted ^***BN***^ **B’s** and Substrate Scope with Respect to Varying Steric Demand on Nitrogen and Boron; Ph* = pentamethylphenyl

Most isolated compounds were obtained as colorless, inert solids, while ^***BN***^ **B3** and ^***BN***^ **B11** were found to be colorless oils, that rapidly decomposed in the presence of air. Samples of ^***BN***^ **B3** turned brown to black when left under ambient conditions, with ^1^H NMR spectra of the residue indicating the formation of multiple decomposition products. This observation is consistent with the ^11^B NMR spectrum, which provides additional evidence for several boron containing decomposition products whose chemical shifts suggest hydrolysis to borinic,^30,42^ boronic,^43–45^ and even boric acid derivatives.^46^ It appears plausible that the decomposition of ^***BN***^ **B3** is initiated by a nucleophilic attack of water (atmospheric moisture) on the Lewis-acidic boron center. The fact that the boron atom is less shielded in ^***BN***^ **B3** compared to the other dihydroazaborinine derivatives explains why such degradation processes are not present elsewhere. Moreover, this observation suggests that the steric demand of the nitrogen substituent plays only a minor role in the kinetic stabilization of dihydroazaborinines, as ^***BN***^ **B8**–^***BN***^ **B10**, despite bearing very small substituents on nitrogen, remain inert upon storage in air. Conversely, the sterically demanding *tert*-butyldimethylsilyl (TBS) substituent in ^***BN***^ **B3** is unable to prevent the aforementioned decomposition. These finding complements previous work,^47^ that likewise concluded that the substituent at the boron atom plays a decisive role in the stability of ^***BN***^ **B** toward oxidation and hydrolysis.^47^ Despite the decomposition of ^***BN***^ **B3** and ^***BN***^ **B11** under ambient conditions, all compounds could be characterized by multinuclear NMR spectroscopy and high-resolution mass spectrometry.

### UV–vis Absorption

With increasing degree of methylation of the aromatic substituent at boron the UV–vis absorption changes only marginally, if the substituent on nitrogen remains unchanged (Figure 2). Since methyl groups exert only a weak inductive effect and do not contribute to an extension of the π-system, this observation is not unexpected. Similarly, methylation on nitrogen results only in a minor bathochromic shift compared to the N-unsubstituted analogue ^***BN***^ **B8**. The stronger inductive effects of the TMS (^***BN***^ **B13**), TBS (^***BN***^ **B1**) and *n*-hexyl (^***BN***^ **B11**) substituents lead to a further bathochromic shift of the absorption wavelengths. However, the magnitude of this effect remains small.

**Figure 2:**
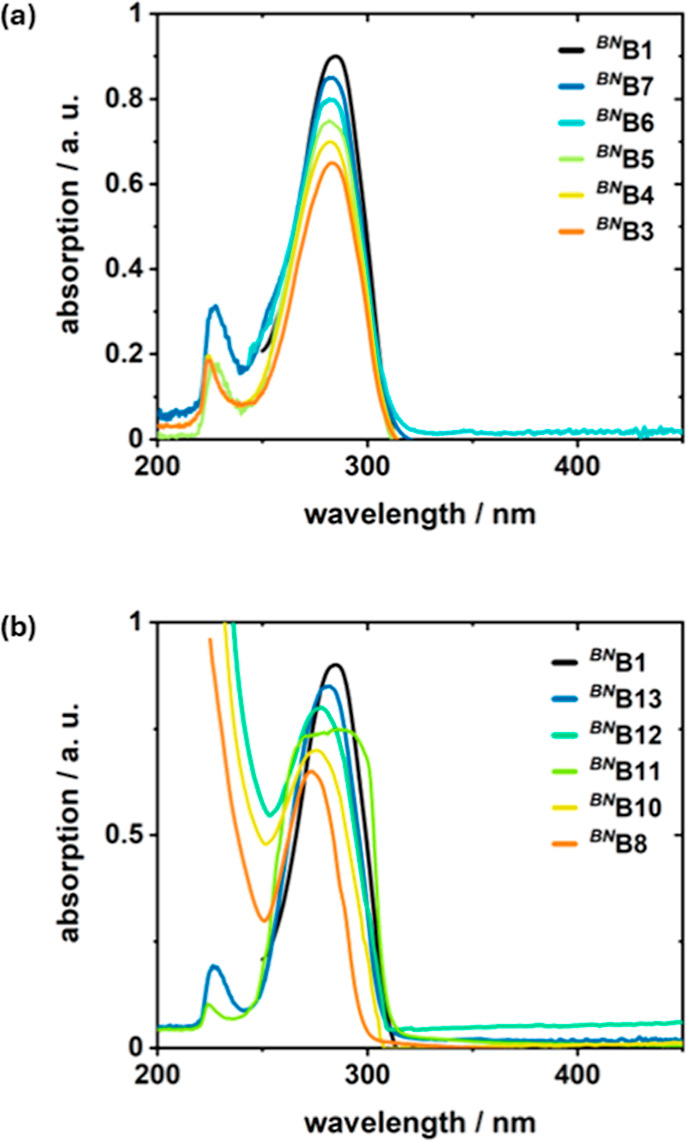
Comparison of the UV–vis absorption of ^***BN***^ **B’s** with different boron (a) or nitrogen (b) substituents. The absorption maxima are scaled to 0.9, 0.85, 0.8, 0.75, 0.7, or 0.65 au, respectively, to enhance the comparability of the highly similar absorption spectra.

### Photoisomerization of ^**BN**^ **B**

Upon broad band irradiation (280–400 nm) using a high-pressure mercury vapor lamp the dihydroazaborinines ^***BN***^ **B3**–^***BN***^ **B7** and ^***BN***^ **B11**–^***BN***^ **B12** isomerize to the corresponding Dewar species (^***BN***^ **D3**–^***BN***^ **D7** and ^***BN***^ **D11**–^***BN***^ **D12**). Comparable to ^***BN***^ **B1** quantitative conversion of the 0.05–0.1 M solutions (*p*-xylol-*d*_10_) is observed by NMR spectroscopy after 5–10 min of irradiation, except for ^***BN***^ **B11**, which required 2 h of irradiation to reach conversion above 95%. In the case of the N-TMS-substituted ^***BN***^ **B13**, decomposition of the starting material is observed already after a few seconds of irradiation and with prolonged light exposure the degree of decomposition increases. In parallel, weak signals of the corresponding ^***BN***^ **D13** were detected by NMR spectroscopy. Both the N-unsubstituted dihydroazaborinines (^***BN***^ **B8**–^***BN***^ **B9**) and the N-methylated ^***BN***^ **B10** exhibit no photoisomerization at room temperature. Upon prolonged irradiation, their *p*-xylol-*d*_10_ solutions develop a pale-yellow coloration, nevertheless only the starting materials can be detected by NMR spectroscopy, with no NMR indication of any (decomposition) products. Since the samples were reliably cooled during irradiation (see the Supporting Information for details), thermal decomposition during the exposure can be considered unlikely. Our group recently demonstrated that irradiation of a UV vis sample of ^***BN***^ **B8** in *n*-hexane at 0 °C causes vanishing of the absorption band of ^***BN***^ **B8**,^48^ supporting the assumption that a photoisomerization of such compounds is in principle possible. As all potential photoisomers are expected to absorb at considerably shorter wavelengths where solvent absorption interferes, the nature of the photoisomerization product could not be conclusively determined by UV-vis spectroscopy for ^***BN***^ **B8**–^***BN***^ **B10**.^48^

The thermal ring opening of ^***BN***^ **D3**–^***BN***^ **D7**, ^***BN***^ **D11** *–* ^***BN***^ **D12** was monitored by NMR spectroscopy in *p*-xylol-*d*_10_ at four different temperatures and the data obtained were analyzed via Arrhenius and Eyring treatments (see Supporting Information for detailed information). Depending on the degree of methylation of the aryl substituent (R^2^), no clear trend in the activation barrier (*E*_a_) or half-life (*t*_1/2_) on the substitution pattern of the investigated ^***BN***^ **D** can be established (Table 1). In most cases, the half-life is on the order of years, as previously reported for ^***BN***^ **D1**,^14^ with activation barriers ranging between 28 and 33 kcal/mol. Only ^***BN***^ **D11** displays a significantly shorter half-life of a few days at 25 °C. Interestingly, the opposing electronic effects in structures ^***BN***^ **D6** and ^***BN***^ **D7** both lead to an increase in the half-life compared to ^***BN***^ **D1** and ^***BN***^ **D3**–^***BN***^ **D5** compounds. Previous studies on the thermal ring opening of C3-substituted ^***BN***^ **D** indicate that a two-electron three-center bond is formed between B, C3, and C6 in the course of the reaction.^16^ Since the electronic effects of the substituents at boron in ^***BN***^ **D6** and ^***BN***^ **D7** perturb the B–N π interaction and likewise the electron distribution in the transition state, a polarization effect appears to be a plausible explanation. Although the Gibbs free activation energy of ^***BN***^ **B1** does not appear as an outlier, it is evident that the individual thermodynamic contributions (Δ*H* ^‡^ and Δ*S* ^‡^) differ from those of all other ^***BN***^ **B** investigated in this study. In particular, a slightly negative activation entropy is observed, whereas the activation enthalpy (and accordingly the comparable *E*_a_ derived from the Arrhenius analysis) is lower than for all other ^***BN***^ **B** derivatives. Since the kinetics of ^***BN***^ **B1** were previously determined in TCE-*d*_2_, a solvent effect appears to be a plausible explanation for this behavior. The change in solvent likely affects both enthalpic and entropic contributions, ultimately leading to enthalpy–entropy compensation and consequently a consistent Δ*G* ^‡^ value.^49^

**Table 1:** Arrhenius and Eyring Parameters for the Thermal Ring Opening of ^***BN***^ **D3**–^***BN***^ **D7** and ^***BN***^ **D11**–^***BN***^ **D12**, Compared to the Literature-Known ^***BN***^ **D1** and ^***BN***^ **D12**

		^***BN***^ **D3**	^***BN***^ **D4**	^***BN***^ **D1** ^a^	^***BN***^ **D5**	^***BN***^ **D6**	^***BN***^ **D7**	^***BN***^ **D11**	^***BN***^ **D12** ^b^
Arrhenius	*E*_a,exp_ (kcal/mol)	29.1	30.0	27.0	29.0	33.3	34.8	27.9	28.7
	ln(*A*) (au)	32.1	32.9	28.8	31.1	37.3	39.5	34.3	28.9
	*t*_1/2,1_ (y)	0.6	1.1	0.5	0.9	3.8	5.2	0.01	7.1
Eyring	Δ*H* ^‡^_ro_ (kcal/mol)	28.4	29.3	26.3	28.3	32.6	34.1	27.2	27.9
	Δ*S* ^‡^_ro_ (cal/(mol K))	2.9	4.6	–3.7	0.9	13.2	17.6	7.4	–3.7
	Δ*G* ^‡^_ro_ (kcal/mol)	27.5	27.9	27.4	28.0	28.7	28.9	25.0	29.0
	Δ*G* ^‡^_ro, calc_ (kcal/mol)	26.1	26.9	26.7	26.9	27.1	26.8	25.9	29.5

a Data from Edel et al.^14^

b Data from Richter et al.^25^

Quantum chemical calculations on the thermal ring opening of ^***BN***^ **D8** and ^***BN***^ **D10** yield even lower activation barriers of just above 20 kcal/mol. In agreement with the previous low-temperature UV–vis measurements,^48^ this supports the hypothesis that photoisomerization of such dihydroazaborinines is feasible at room temperature, while the potential photo isomers are not inert toward unknown decomposition paths and thermal ring opening in this temperature regime. Upon heating, partial recovery of the ^***BN***^ **B8** absorption was observed, accompanied by additional broad UV–vis signals, presumably due to decomposition.^48^ Accordingly, the kinetic and thermodynamic parameters of the thermal back-reaction could not be determined for ^***BN***^ **D8**–^***BN***^ **D10**.

### Energy Density

To access the energy stored in ^***BN***^ **D3**–^***BN***^ **D7** and ^***BN***^ **D11** differential scanning calorimetry (DSC) measurements were performed (Table 2). To avoid decomposition of the ^***BN***^ **D** compounds in the solid state dodecane solutions were used for the DSC measurements as described earlier.^15,100^ The compounds were irradiated in solution and the solvent was never removed (see Supporting Information for additional information). All measurements exhibited the characteristic DSC signal pattern of an exothermic process, which was assigned to the thermal back-conversion of the ^***BN***^ **D** to their corresponding ^***BN***^ **B** isomers. The completeness of energy release was verified by cooling the sample in the device and subsequently subjecting it to the same heating slope again without any intermediate treatment.

**Table 2:** Experimentally Determined Stored Energy of ^***BN***^ **B3**–^***BN***^ **B7** Compared to the Literature-Known Value of ^***BN***^ **B1** and Δ*H*_theor_ at the B3LYP/6-311+G­(d,p) Level of Theory

	^***BN***^ **B1** ^a^	^***BN***^ **B3**	^***BN***^ **B4**	^***BN***^ **B5**	^***BN***^ **B6**	^***BN***^ **B7**	^***BN***^ **B11**
Δ*H*_exp_ (kJ/kg)	639.4	711.9	725.7	619.5	603.4	669.2	707.5
Δ*H*_exp_ (kcal/mol)	48.1	50.6	49.1	50.3	51.6	51.6	47.6
Δ*H*_theor_ (kcal/mol)	50.8	54.2	52.3	50.1	52.6	54.9	59.8

a Data from Edel et al.^14^

Unfortunately, the high storage energy predicted for ^***BN***^ **B11** could not be confirmed experimentally. The DSC measurements initially exhibit the expected strongly exothermic signal, followed by a broad, weakly exothermic feature (Figure S118). Since the DSC experiments were conducted in solution, the possibility of an overlapping phase transition can be excluded. Instead, it appears more likely that decomposition occurs in parallel with the thermal back-reaction under the applied high-temperature conditions.

### Computations

To investigate the influence of B–N-substitution on the activation barrier and the stored energy more broadly, combinations of the B- and N-substituents mentioned above, as well as N-ethyl and *N*-isopropyl substitution, were computationally evaluated (Figure 3).

**Figure 3:**
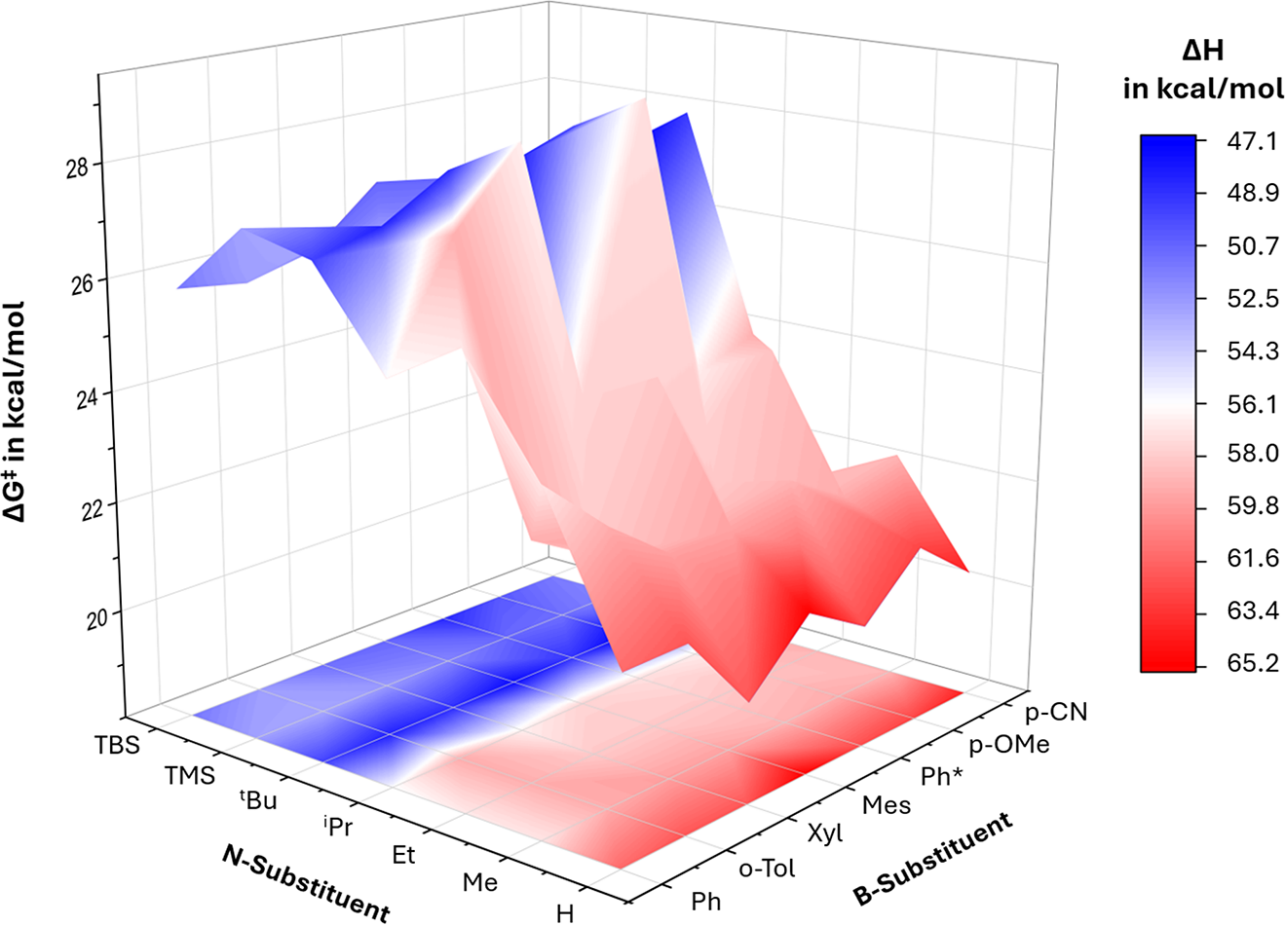
3D-surface plot of the B- (*x* axis) and N-substituent (*y* axis) against the activation barrier (*z* axis). The heatmap on the surface refers to the stored energy. For improved visibility this heatmap is also shown in the *xy* plane.

Small N-substituents lead to an increase in stored energy of up to 10 kcal/mol. However, as mentioned earlier, experimental findings indicate that the corresponding ^***BN***^ **D** cannot be detected at room temperature, rendering these systems unsuitable as MOST candidates. While the activation barrier for the thermal ring-opening increases with the steric demand of the N-substituent, the stored energy decreases simultaneously. A favorable compromise appears to be provided by the experimentally unexplored *N*-isopropyl-*B*-xylyl ^***BN***^ **B**, which combines a high stored energy of approximately 60 kcal/mol with an activation barrier of 32 kcal/mol.

## Conclusions

In this work, a series of dihydroazaborinines bearing various substituents at nitrogen and boron were synthesized starting from ^***BN***^ **B2**. For this purpose, literature-known procedures were employed with modifications.^14,25,35,39–41^ The obtained dihydroazaborinines were subsequently investigated with respect to their stability toward air, their UV–vis absorption and their ability to undergo photoisomerizations. It was shown that the steric demand of the substituent at boron is crucial for the kinetic stabilization of the dihydroazaborinines in air, as it prevents nucleophilic substitution induced by moisture. In contrast, photoisomerization to the corresponding Dewar isomer was observed only when a sufficiently bulky substituent was present at nitrogen (Scheme 3). Low-temperature UV–vis measurements further confirmed that photoisomerization of dihydroazaborinines with small substituents at nitrogen is in principle possible, but the resulting isomers are not inert at room temperature and are susceptible both to thermal back-reaction and to decomposition processes. Especially ^***BN***^ **B4–B7** exhibit promising properties and are able to compete literature-known ^***BN***^ **B** in view of potential MOST applications. With respect to quantum-chemical calculations, systems bearing an isopropyl substituent on nitrogen appear particularly attractive, as they represent a good balance between sufficient steric demand and the molecular weight of the compound. In this way, both increased energy densities and long half-lives could potentially be achieved. Other parameters, such as UV–vis absorption maxima, are only marginally affected by the varying substitution. Hence, a deliberate choice of the nitrogen substituent enables selective tuning of the half-life of the energy-storage state, a key parameter in the design of molecular photoswitches.

**Scheme 3:**
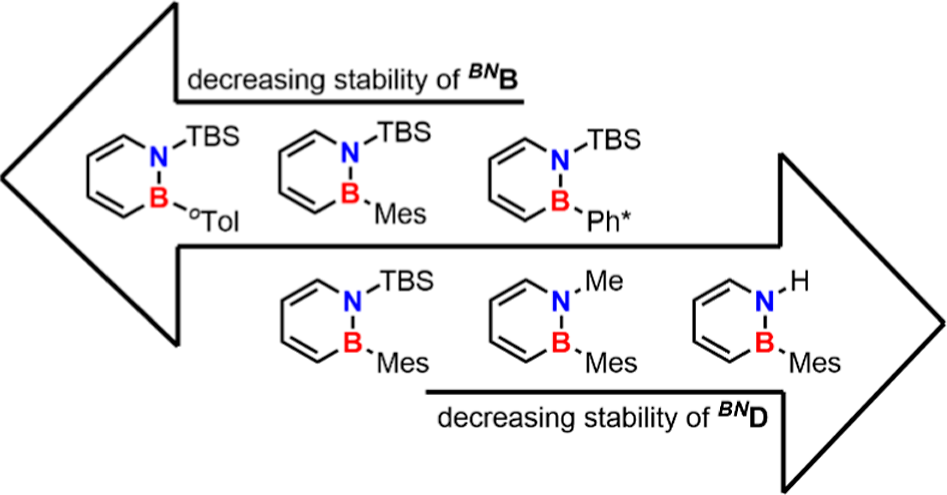
Influence of Sterically Demanding Substituents on Boron (Top) and Nitrogen (Bottom) on the Stability of ^***BN***^ **B**’s and ^***BN***^ **D**’s

## Supplementary Material



## Data Availability

The data underlying this study are available in the published article and its Supporting Information.

## Notes

Experimental details, NMR, MS and DSC spectral data, kinetic analysis data for the thermal ring opening, computational details, Cartesian coordinates and crystal structure data (PDF)
