# Supplementary material for: Substituent Tightrope: Balancing Energy Storage and Stability of Dihydroazaborinine Molecular Solar Thermal Energy Storage Candidates
Source: J Org Chem. 2026 Jul 20;91(30):10454–60. doi: 10.1021/acs.joc.6c00925 (PMC13435307; doi:10.1021/acs.joc.6c00925)
Supplement: Supplementary file 1 [file jo6c00925_si_001.pdf]

Supporting Information

The Substituent Tightrope: Balancing Energy Storage and Stability of Dihydroazaborinine Molecular Solar Thermal Energy Storage Candidates

Sonja M. Biebl,[a] Niklas Thiess,[a] Kathrin Zwettler,[a] Markus Ströbele,[b] Holger F. Bettinger[a,*]

[a] Institut für Organische Chemie, Eberhard Karls Universität Tübingen, Auf der Morgenstelle 18, 72076 Tübingen, Germany, E-Mail: holger.bettinger@uni-tuebingen.de

[b] Institut für Anorganische Chemie, Eberhard Karls Universität Tübingen, Auf der Morgenstelle 18, 72076 Tübingen, Germany

Table of Content

1. Methods 4

2. Analytics 4

3. Synthesis 6

3.1 Synthesis of the aryl lithium species 6

3.1.1 *ortho*-Tolyl lithium 6

3.1.2 2-Xylyl lithium 10

3.1.3 Mesityl lithium 14

3.1.4 Pentamethylphenyl lithium 18

3.2 Synthesis of the Dihydroazaborinines 22

3.2.1 1-(*tert*-Butyldimethylsilyl)-2-mesityl-1,2-dihydro-1,2-azaborinine (^{BN}B1) 22

3.2.3 1-(*tert*-Butyldimethylsilyl)-2-(ortho-tolyl)-1,2-dihydro-1,2-azaborinine (^{BN}B3) ... 27

3.2.2 1-(*tert*-Butyldimethylsilyl)-2-(2-xylyl)-1,2-dihydro-1,2-azaborinine (^{BN}B4) 32

3.2.4 1-(*tert*-Butyldimethylsilyl)-2-(pentamethylphenyl)-1,2-dihydro-1,2-azaborinine (^{BN}B5) 37

3.2.7 1-(*tert*-Butyldimethylsilyl)-2-(4-methoxy-2,6-dimethylphenyl)-1,2-dihydro-1,2-azaborinine (^{BN}B6) 42

3.2.8 1-(*tert*-Butyldimethylsilyl)-2-(4-cyano-2,6-dimethylphenyl)-1,2-dihydro-1,2-azaborinine (^{BN}B7) 46

3.2.5 2-(Pentamethylphenyl)-1,2-dihydro-1,2-azaborinine (^{BN}B9) 50

3.2.9 1-Hexyl-2-mesityl-1,2-dihydro-1,2-azaborinine (^{BN}B11) 55

3.2.6 1-Trimethylsilyl-2-mesityl-1,23-dihydro-1,2-azaborinine (^{BN}B13) 60

4. Irradiation experiments 65

5. Kinetic experiments 90

5.1 Sample preparation 90

5.2 Experimental details 90

5.3 Time dependence (1. order kinetic) 95

6. DSC measurements 96

7. Crystal structures 104

7. Computations 107

7.1 Cartesian coordinates 114

7.2 IRC Scans 206

8. References 214

1. Methods

Unless otherwise noted, all experiments were carried out under inert conditions using Schlenk technique with argon or nitrogen as the protective gas or in a glove box (UNIlab Pro, MBraun). Glassware was dried before use by heating. All reactions conducted at elevated temperatures were heated using an oil bath. Commercial triethylamine (water content ~ 0.2%) was refluxed over KOH for two hours and then distilled before use. The remaining chemicals used were employed as received from the manufacturer without further purification. Anhydrous solvents were obtained from Thermo Fisher Scientific Inc., Acros Organics B.V. B.A., Sigma-Aldrich or, in the case of dichloromethane, diethyl ether, *n*-hexane, tetrahydrofuran and toluene, from an SPS-800 solvent drying system by the manufacturer MBraun.

2. Analytics

NMR Spectroscopy: The acquisition of ^{1}H, ^{13}C-{^{1}H}, and ^{11}B-{^{1}H} NMR spectra were performed on a Bruker Advance III HD 400 MHz instrument (^{1}H spectra) at 101 MHz (^{13}C-{^{1}H} spectra), and 128 MHz (^{11}B-{1H} spectra). Complementarily, spectra of these nuclei were recorded on a Bruker Advance III HD 300 MHz NanoBay at 300 MHz (^{1}H spectra), 76 MHz (^{13}C-{^{1}H} spectra), and 96 MHz (^{11}B-{1H} spectra). For high-temperature NMR measurements (kinetic experiments), a Bruker Avance III HDX 600 spectrometer was employed with a measuring frequency of 600 MHz (^{1}H spectra), 151 MHz (^{13}C-{^{1}H} spectra) or 192 MHz (^{11}B-{1H} spectra) and on a Bruker Avance III HDX 700 with a measuring frequency of 700 MHz (^{1}H spectra) or 176 MHz (^{13}C-{^{1}H} spectra). All obtained NMR spectra were referenced to the solvent peak. Deuterated dichloromethane (5.32 ppm), benzene (7.16 ppm), chloroform (7.26 ppm), cyclohexane (1.38 ppm), or tetrachloroethane (5.91 ppm) from Sigma-Aldrich or Deutero were used for referencing. Structural assignments were made with additional information from gCOSY, gHSQC, and gHMBC experiments.

MPLC Chromatography: Column chromatographic purifications were performed using the puriFlash 430 in combination with pre-packed silica gel columns (particle size: 30 µm) from Interchim. Detection was carried out using a puriFlash One Series UV detector (DAD 200 – 600 nm) from the same manufacturer.

All solvents used were of HPLC-grade purity. Solvent mixtures are indicated as V/V ratio.

GPC Chromatography: The size exclusion chromatography was conducted on a Recycling Preparative HPLC and GPC (LaboACE LC-7080 Plus II) device and a JAIGEL-2.5 HR Plus column both from Japan Analytical Industry Co.

UV-vis Spectroscopy: For determining the absorption maxima of the presented 1,2-substituted 1,2-dihydro-1,2-azaborinines, a Lambda 1050 UV/VIS/NIR spectrometer (Perkin Elmer) with a 3D WB detection module was used, operated with the accompanying UV Win Lab software (Version 2.0.2). The wavelength range covered was from 250 nm to 850 nm. All solvents used had a cut-off wavelength below 250 nm and were water-free.

Irradiation experiments: As light source an Osram HBO-500-W/2 high pressure mercury lamp in an Oriel housing with quartz optics and a dichroic mirror (280 – 400 nm) was applied. No additional filters were used.

The samples for the kinetic studies prepared in quartz glas J.-Young NMR tubes as the reaction vessel.

X-Ray Crystallography: Crystals suitable for X-Ray diffraction were grown by vapor diffusion with *n*-hexane and dichloromethane at room temperature. Single crystals were selected, coated with Parabar 10312 and fixed on a microloop.

Data were collected on a XtaLAB Synergy, Dualflex, HyPix diffractometer using *w* scans with Cu K_{a} radiation. The crystal was kept at a steady temperature during data collection. The diffraction pattern was indexed and the total number of runs and images was based on the strategy calculation from the program CrysAlisPro 1.171.42.49, which was also used for refining the unit cell. The structure was solved with the **ShelXT** 2018/2 solution program[1-3] using dual methods and by using **Olex2** 1.5-ac5-024 as the graphical interface.[4] The model was refined with **olex2.refine** 1.5-ac5-024 using full matrix least squares minimization on $\boldsymbol{F^2}$.[5]

Mass spectrometry: High resolution mass spectra were recorded on a HR-ESI/APCI-TOF device (maXis 4G, Bruker) or a HR-ESI/ASAP-Orbitrap-MS (Q exactive HF, Thermo Scientific) for ESI ionization. The sample was dissolved in dichloromethane and injection was carried out using a syringe pump (ESI/APCI).

3. Synthesis

1-(*tert*-Butyldimethylsilyl)-2-mesityl-1,2-dihydro-1,2-azaborinine (^{BN}**B1**) and 1-(*tert*-butyldimethylsilyl)-2-mesityl-1,2-dihydro-1,2-azaborinine (^{BN}**B15**) was synthesized according to Richter *et al*.[6]

1-(*tert*-Butyldimethylsilyl)-2-chloror-1,2-dihydro-1,2-azaborinine (^{BN}**B2**) and 1-(*tert*-butyldimethylsilyl)-2-chloro-1,2-dihydro-1,2-azaborinine (^{BN}**B16**) was synthesized according to Richter *et al.*[6] and Marwitz *et al*.[7]

1-(*tert*-butyldimethylsilyl)-2-chloro-1,2-dihydro-1,2-azaborinine (^{BN}**B16**) was synthesized according to Richter *et al.*[6] and Lamm *et al*.[8]

2-mesityl-1,2-dihydro-1,2-azaborinine (^{BN}**B8**) and 1-methyl-2-mesityl-1,2-dihydro-1,2-azaborinine (^{BN}**B10**) were synthesized according to Lee *et al*.[9]

3.1 Synthesis of the aryl lithium species

3.1.1 *ortho*-Tolyl lithium

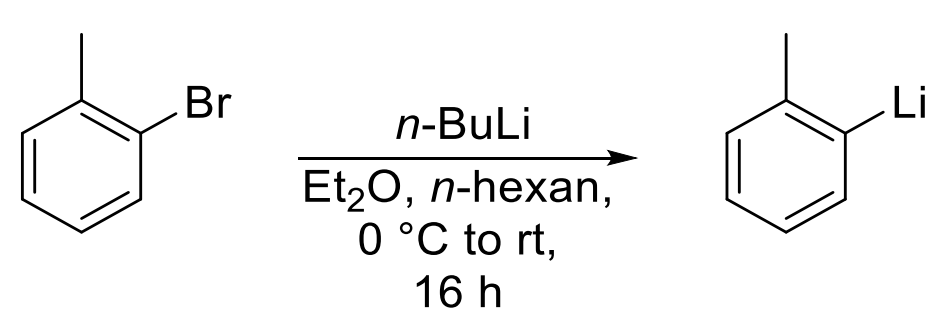

This synthesis is based on a procedure by Pfeffer *et al*.[10]

2-Bromotoluene (3.5 mL, 29 mmol, 1 eq.) was dissolved in dry diethyl ether (5 mL) and cooled to 0 °C. Upon stirring, *n*-butyl lithium (2.5 M in *n*-hexane, 13 mL, 32 mmol, 1.1 eq.) was added dropwise. The reaction mixture was allowed to warm to room temperature and stirred overnight. The reaction mixture was filtered through an inert-gas frit, and the residue was washed with *n*-hexane (2 × 5 mL) and dried *in vacuo*. The product was obtained as a white solid (1.9055 g, 19 mmol, 67%).

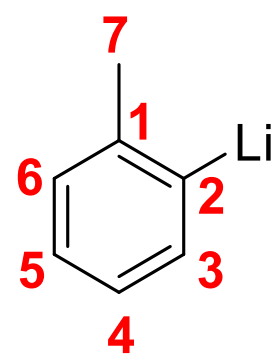

C_7H_7Li (98.07 g/mol)

^{1}H NMR (600 MHz, **C_6D_6**): δ = 7.09 (m, 4H, H-3/H-4/H-5/H-6), 2.13 (s, 3H, H-7) ppm.

^{13}C-{^{1}H} NMR (101 MHz, **C_6D_6**): δ = 141.1, 135.8, 130.2, 129.2,127.6, 125.7, 19.3 ppm.

HRMS measurements were not possible owing to the pronounced air- and moisture-sensitivity of the compound.

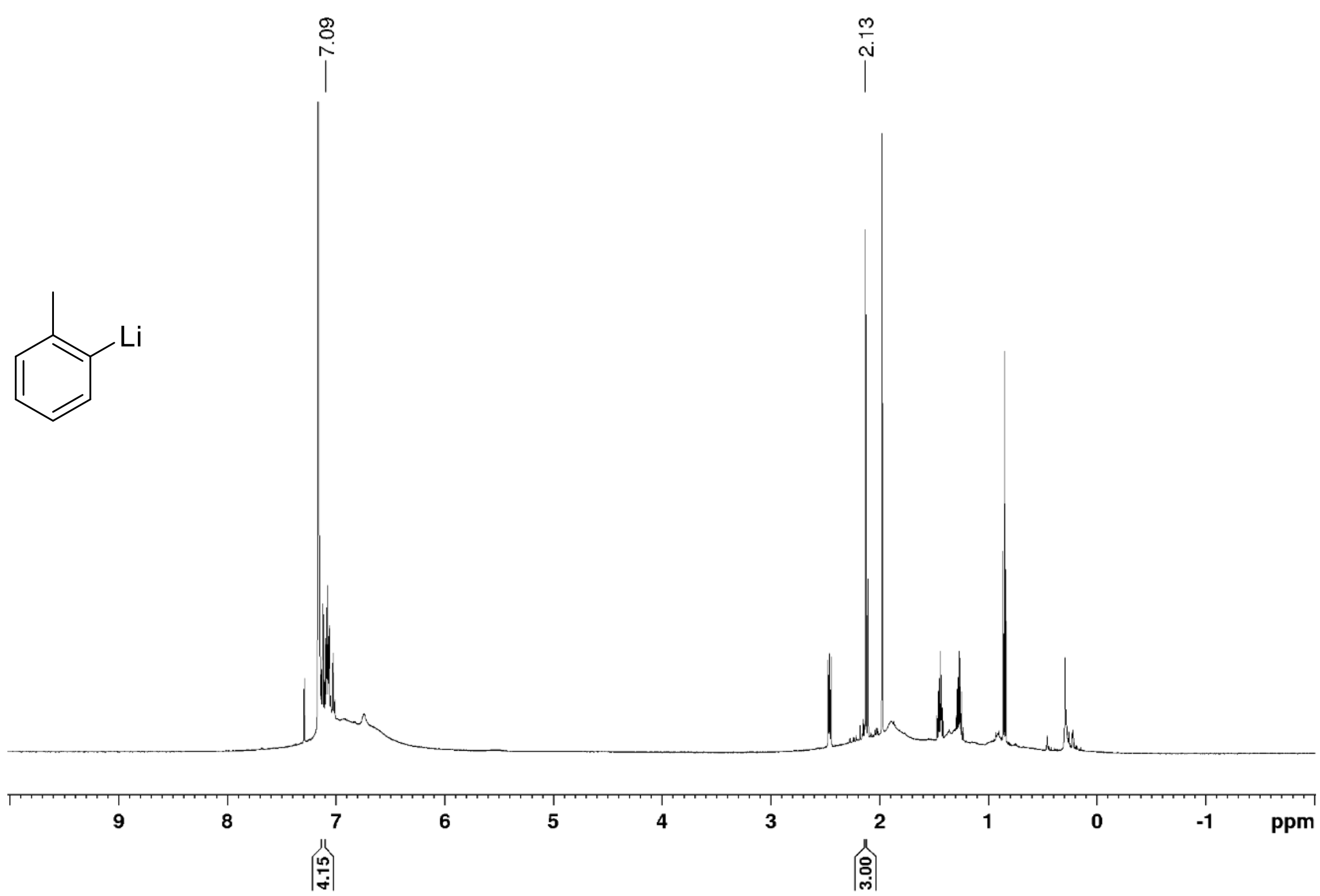

Figure S1. ^{1}H NMR spectrum of *ortho*-tolyl lithium measured in C_6D_6 on a 600 MHz spectrometer.

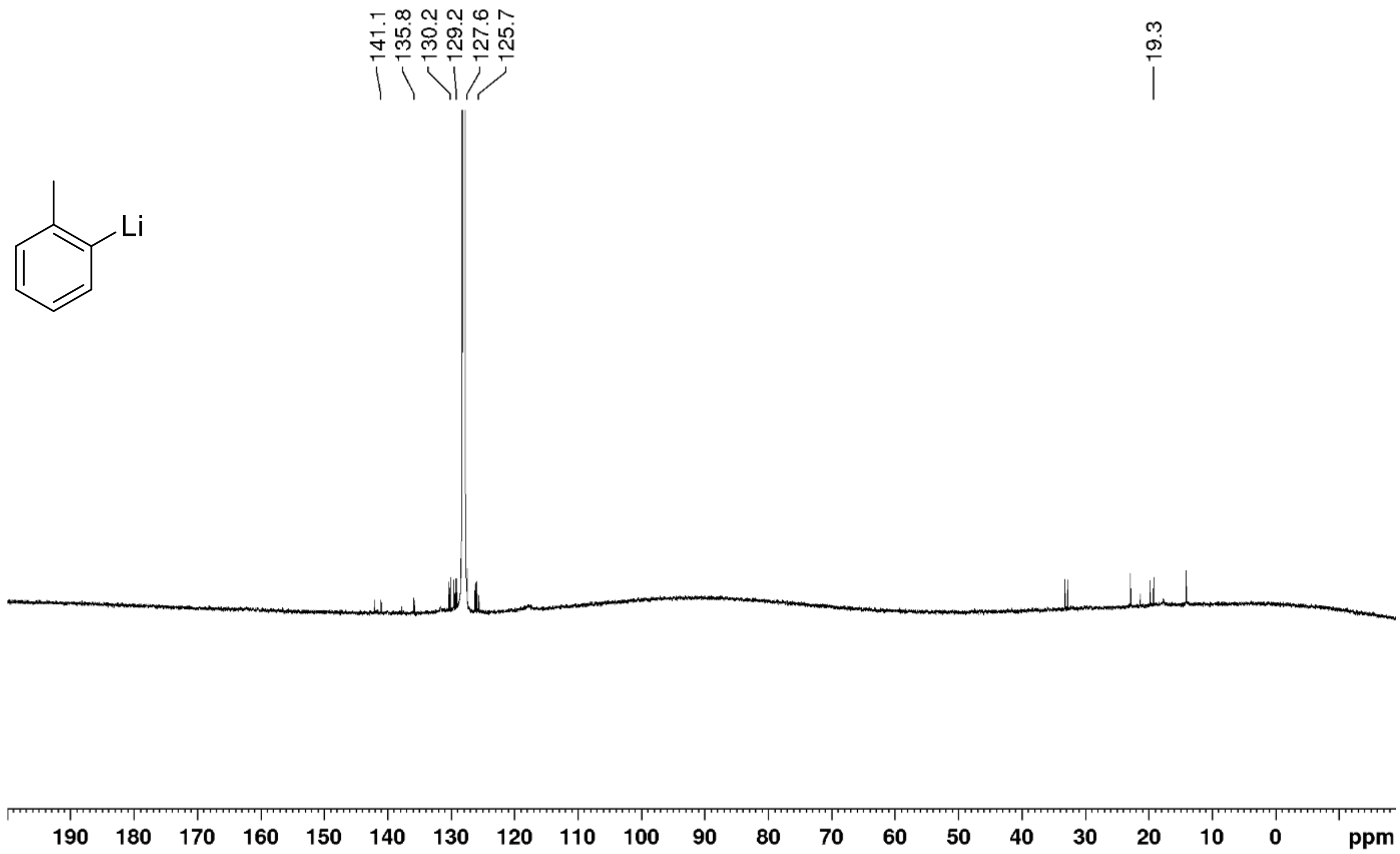

Figure S2. ^{13}C-{^{1}H} NMR spectrum of *ortho*-tolyl lithium in C_6D_6 measured on a 600 MHz spectrometer.

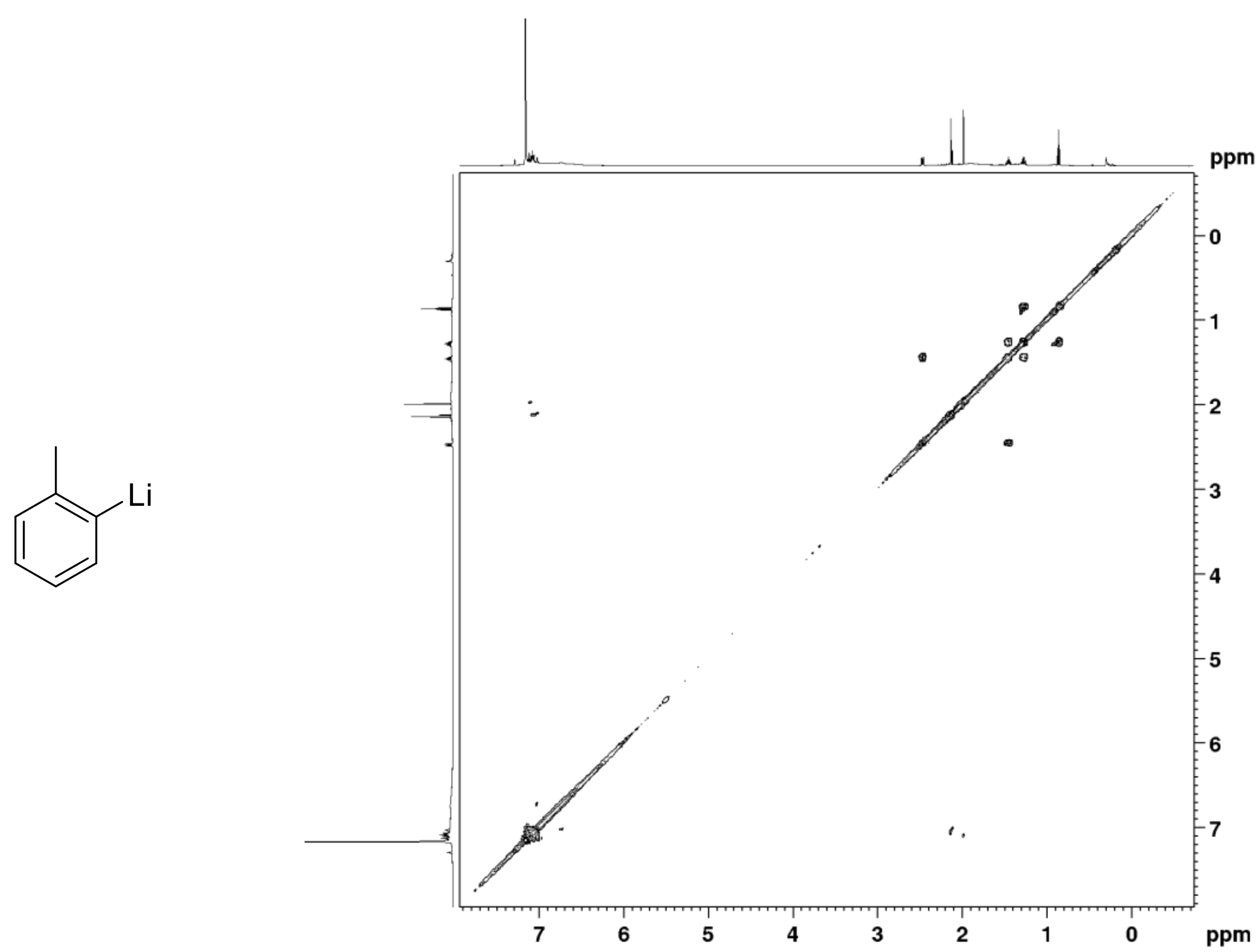

Figure S3. ^{1}H-^{1}H-COSY NMR spectrum of *ortho*-tolyl lithium in C_6D_6 measured on a 600 MHz spectrometer.

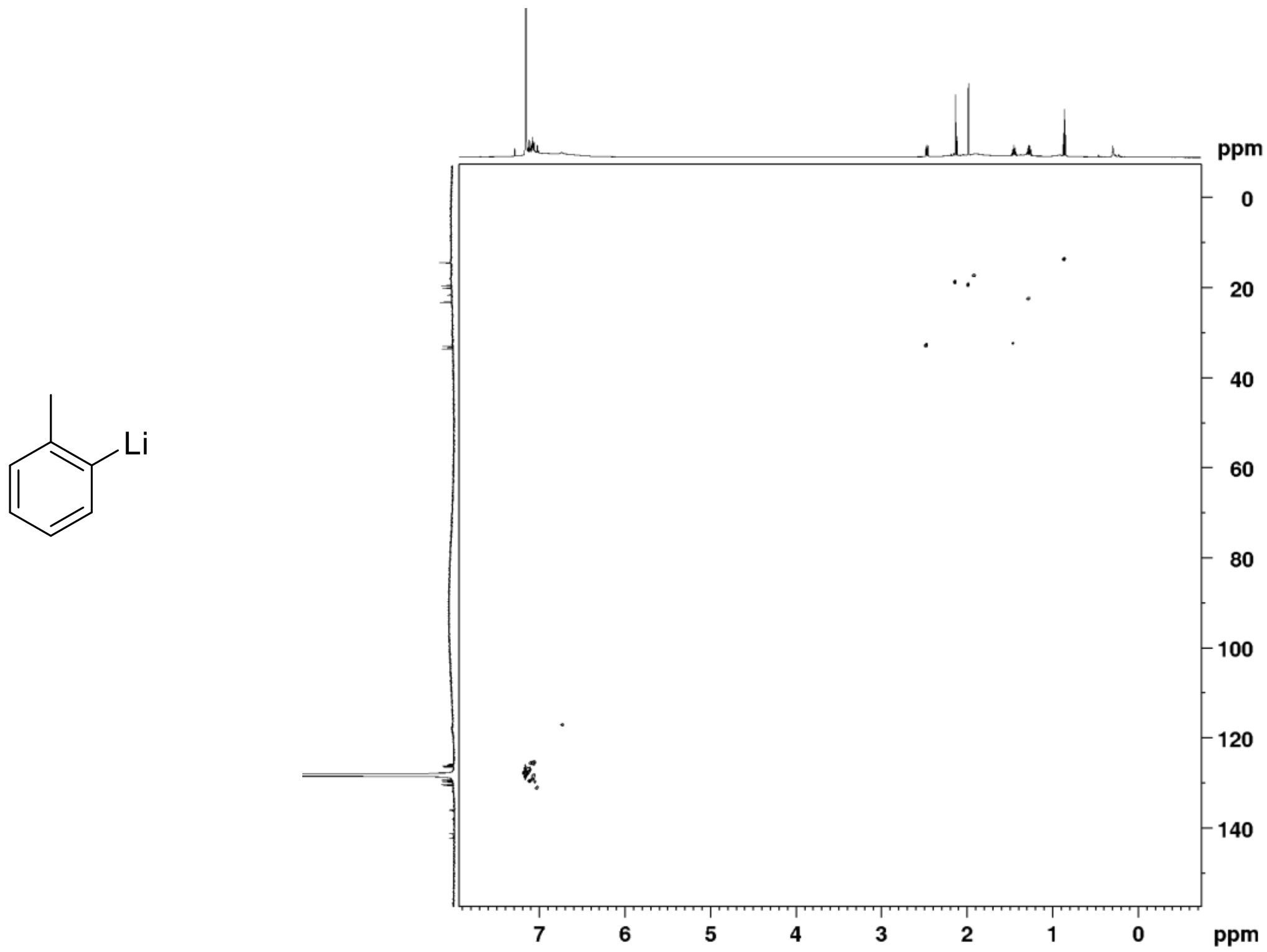

Figure S4. ^{1}H-^{13}C-HSQC NMR spectrum of *ortho*-tolyl lithium in C_6D_6 measured on a 600 MHz spectrometer.

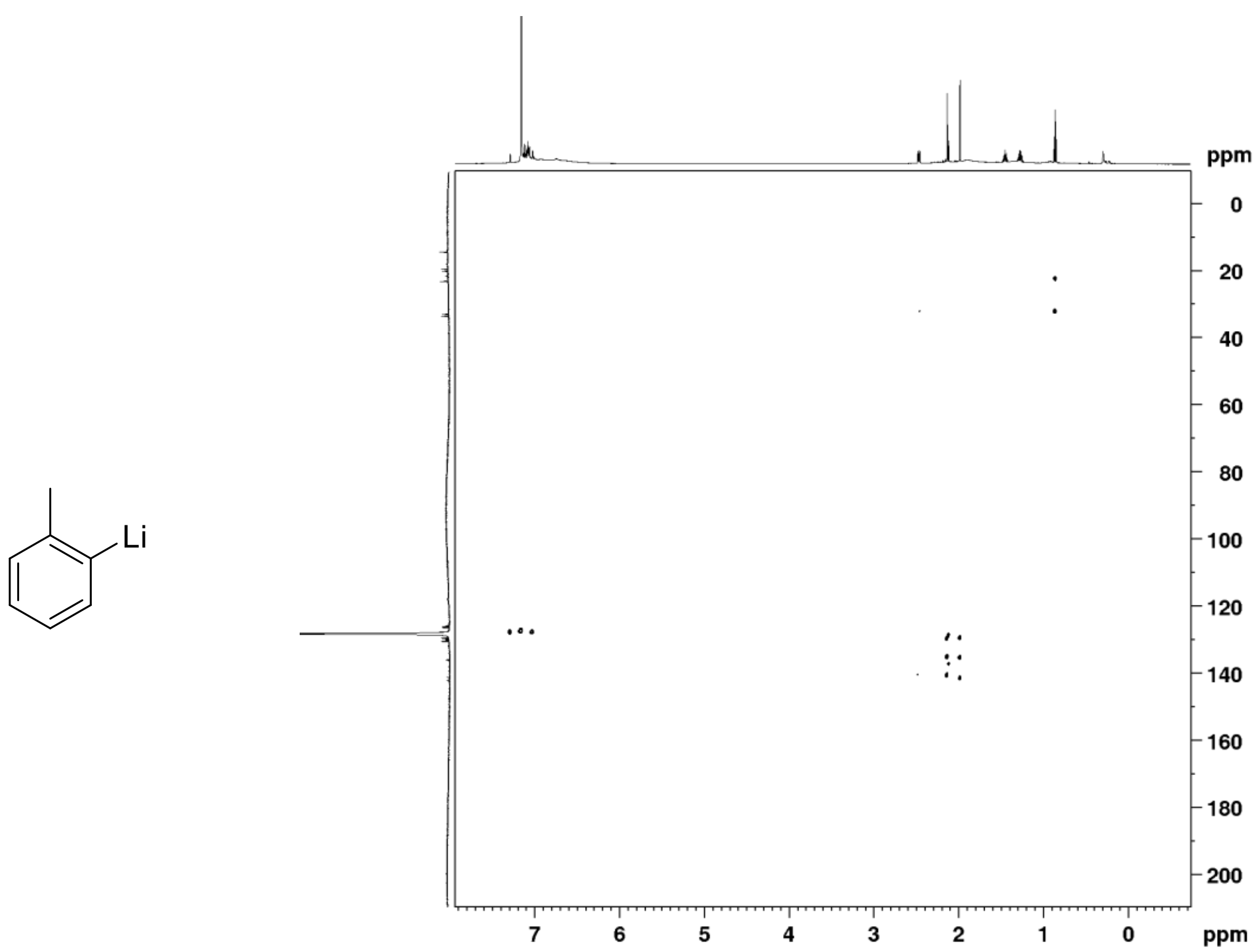

Figure S5. ^{1}H-^{13}C-HMBC NMR spectrum of *ortho*-tolyl lithium in C_6D_6 measured on a 600 MHz spectrometer.

3.1.2 2-Xylyl lithium

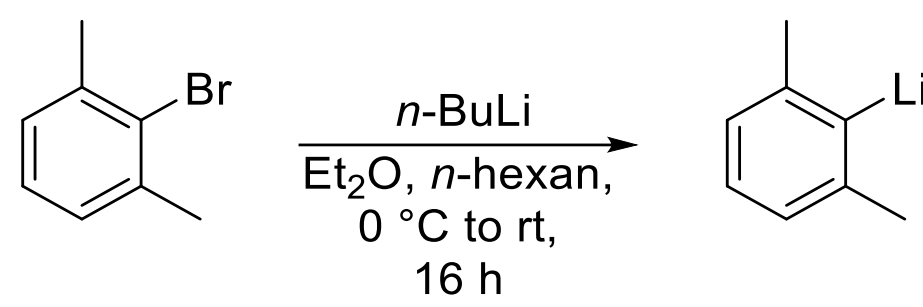

The synthesis is based on a procedure by Pfeffer *et al.*[10]

2-Bromoxylene (3.7 mL, 27 mmol, 1 eq.) was dissolved in dry diethyl ether (5 mL) and cooled to 0 °C. Under stirring, *n*-butyllithium (2.5 M in *n*-hexane, 12 mL, 30 mmol, 1.1 eq.) was added dropwise. The reaction mixture was allowed to warm to room temperature and stirred overnight. The reaction mixture was filtered through an inert gas frit, and the residue was washed with *n*-hexane (2 × 5 mL) and dried *in vacuo*. The product was obtained as a white solid (2.8451 g, 25 mmol, 94%).

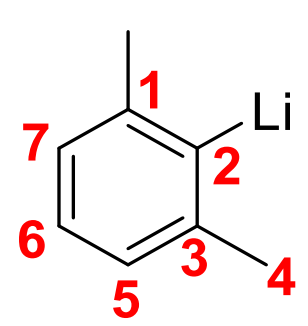

C_8H_9Li (112.10 g/mol)

^{1}H NMR (600 MHz, **C_6D_6**): δ = 7.10 (ps. t, 1H, H-6),6.90 (m, 1H, H-5/H-7), 6.85 (br. s, 1H, H-5/H-7), 2.14 (s, 6H, H-4) ppm.

^{13}C-{^{1}H} NMR (101 MHz, **C_6D_6**): δ = 137.8, 130.2, 128.5, 126.4, 21.4 ppm.

HRMS measurements were not possible owing to the pronounced air- and moisture-sensitivity of the compound.

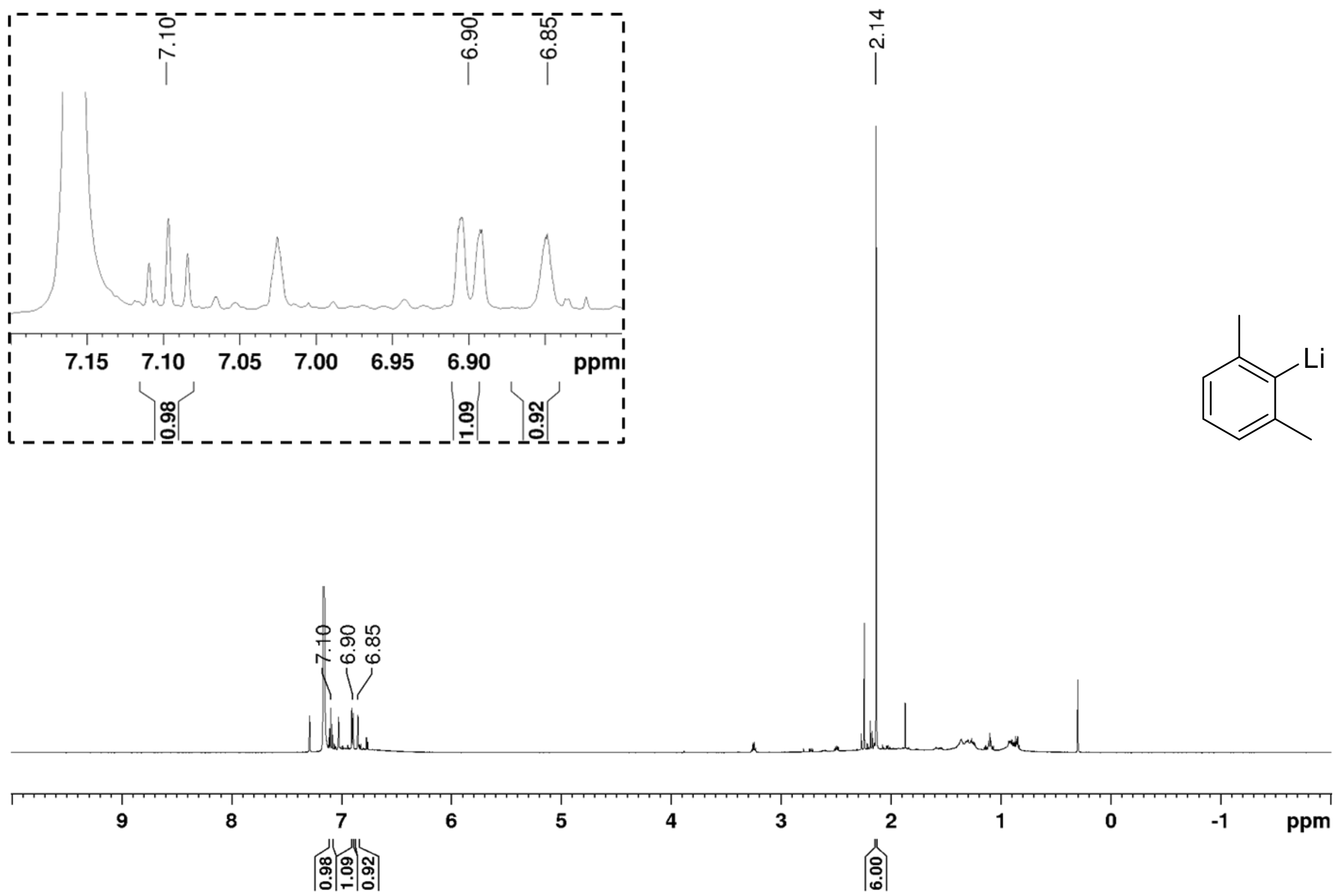

Figure S6. ^{1}H NMR spectrum of 2-xylyl lithium in C_6D_6 measured on a 600 MHz spectrometer.

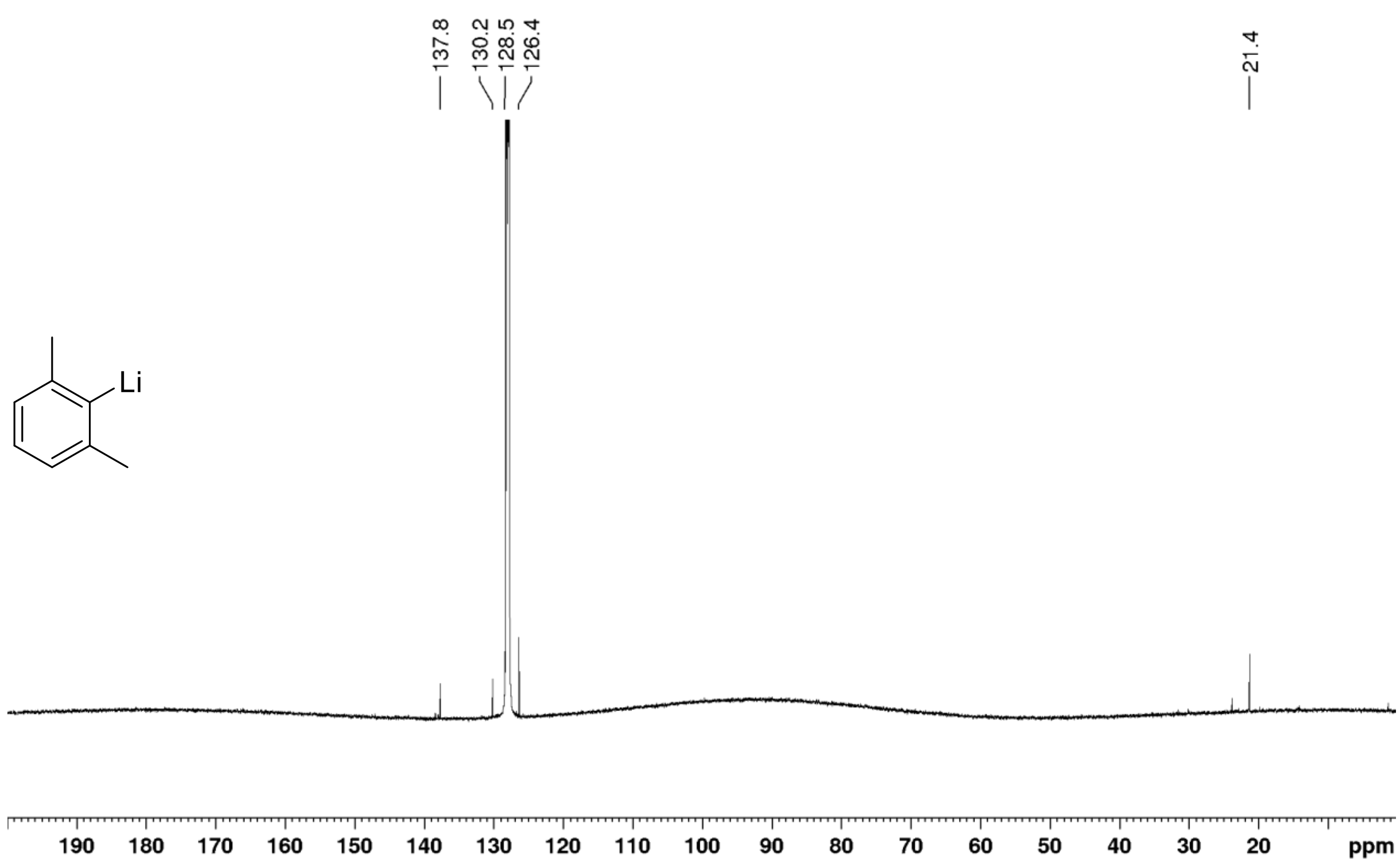

Figure S7. ^{13}C-{^{1}H} NMR spectrum of 2-xylyl lithium in C_6D_6 measured on a 600 MHz spectrometer.

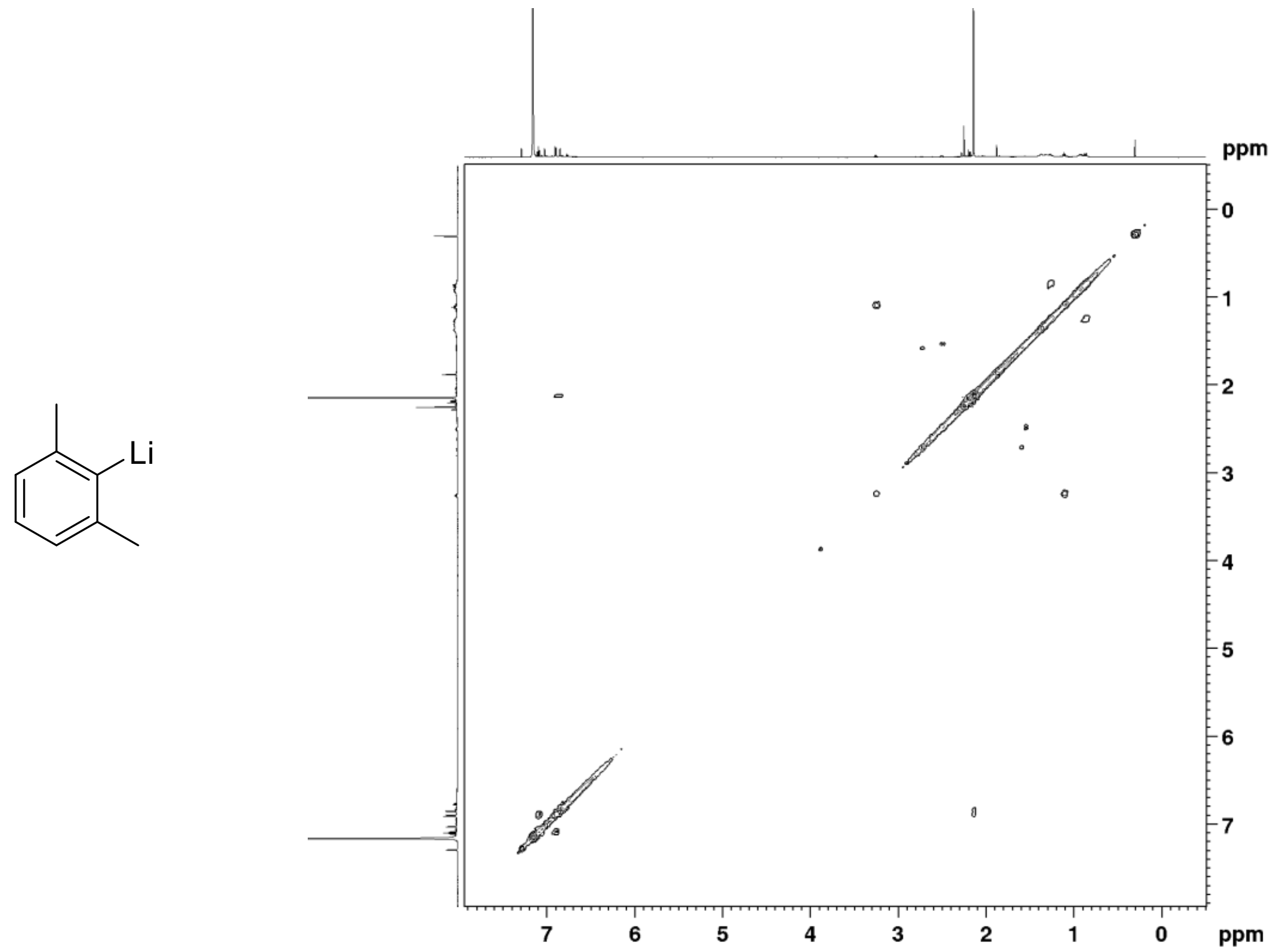

Figure S8. ^{1}H-^{1}H-COSY NMR spectrum of 2-xylyl lithium in C_6D_6 measured on a 600 MHz spectrometer.

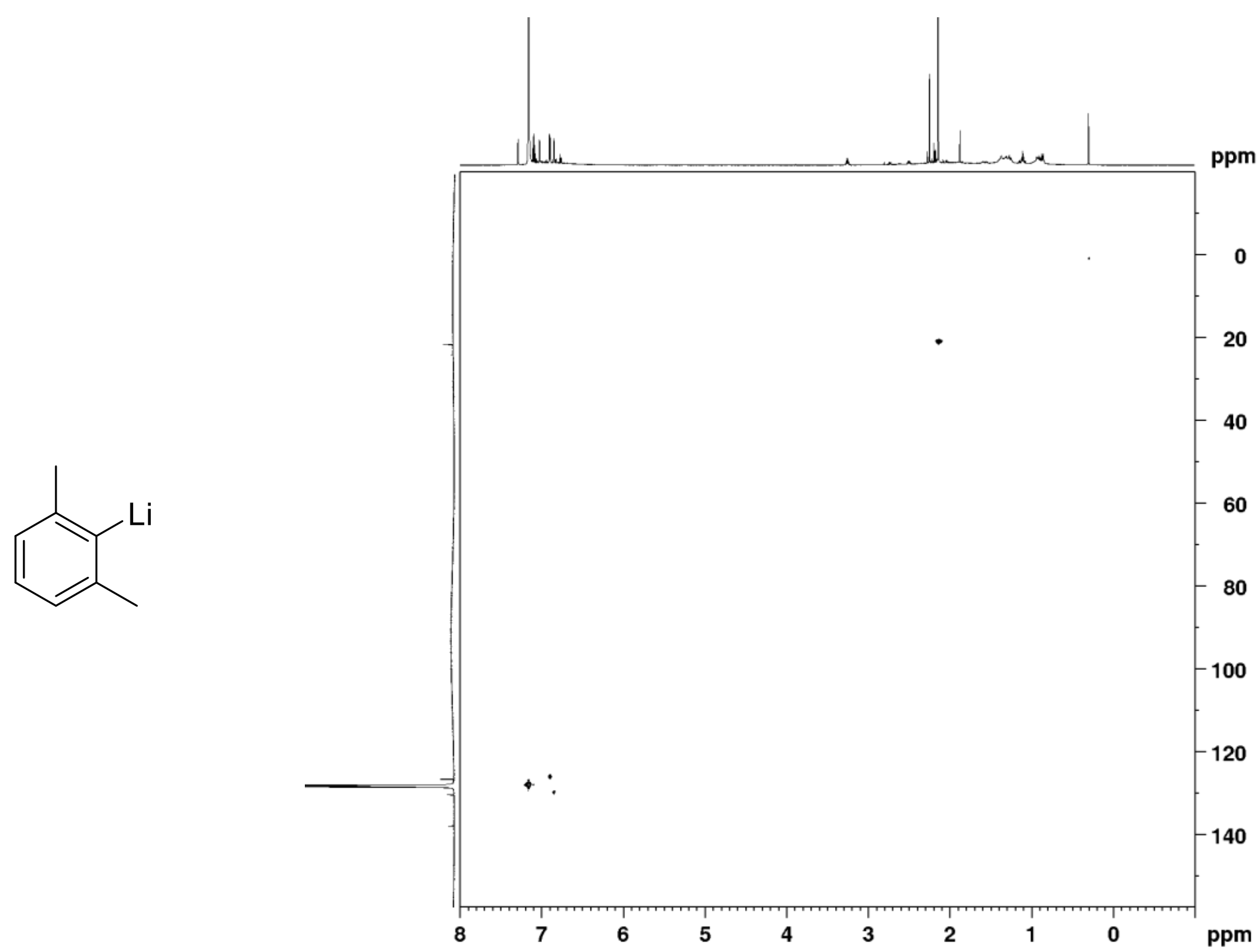

Figure S9. ^{1}H-^{13}C-HSQC NMR spectrum of 2-xylyl lithium in C_6D_6 measured on a 600 MHz spectrometer.

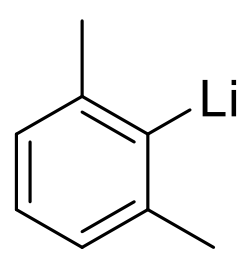

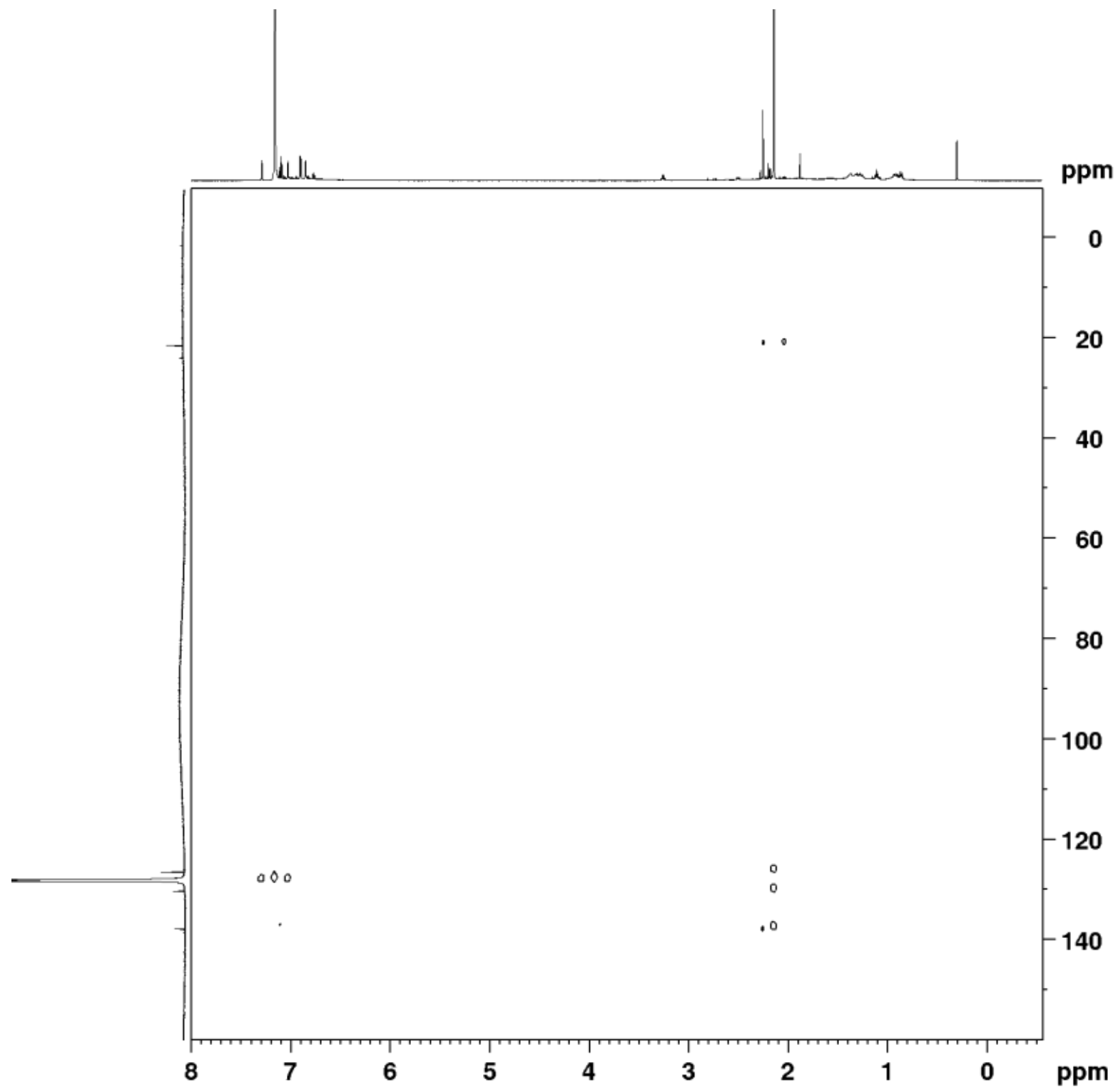

Figure S10. ^{1}H-^{13}C-HMBC NMR spectrum of 2-xylyl lithium in C_6D_6 measured on a 600 MHz spectrometer.

3.1.3 Mesityl lithium

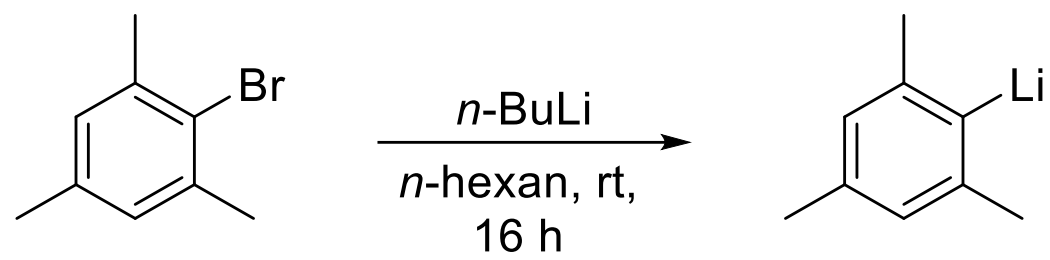

The synthesis is based on a procedure by Edel *et al.*[11]

2-Bromomesitylene (5.0 mL, 32.5 mmol, 1 eq.) was dissolved in dry *n*-hexane (10 mL) and cooled to 0 °C. Under stirring, *n*-butyllithium (2.5 M in *n*-hexane, 14.4 mL, 36 mmol, 1.1 eq.) was added dropwise. The reaction mixture was allowed to warm to room temperature and stirred overnight. The reaction mixture was filtered through an inert-gas frit, and the residue was washed with *n*-hexane (2 × 10 mL) and dried *in vacuo*. The product was obtained as a white solid (3.9956 g, 32 mmol, 97%).

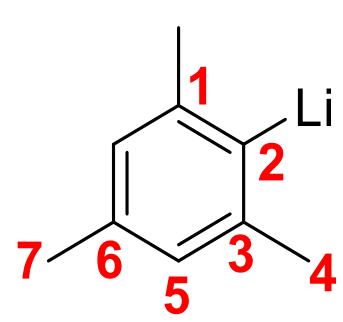

$C_9H_{11}Li$ (126.13 g/mol)

^{1}H NMR (600 MHz, **C_6D_6**): δ = 6.72 (s, 2H, H-5), 2.16 (s, 9H, H-4/H-7) ppm.

^{13}C-{^{1}H} NMR (101 MHz, **C_6D_6**): δ = 137.6, 128.3, 127.4, 21.3 ppm.

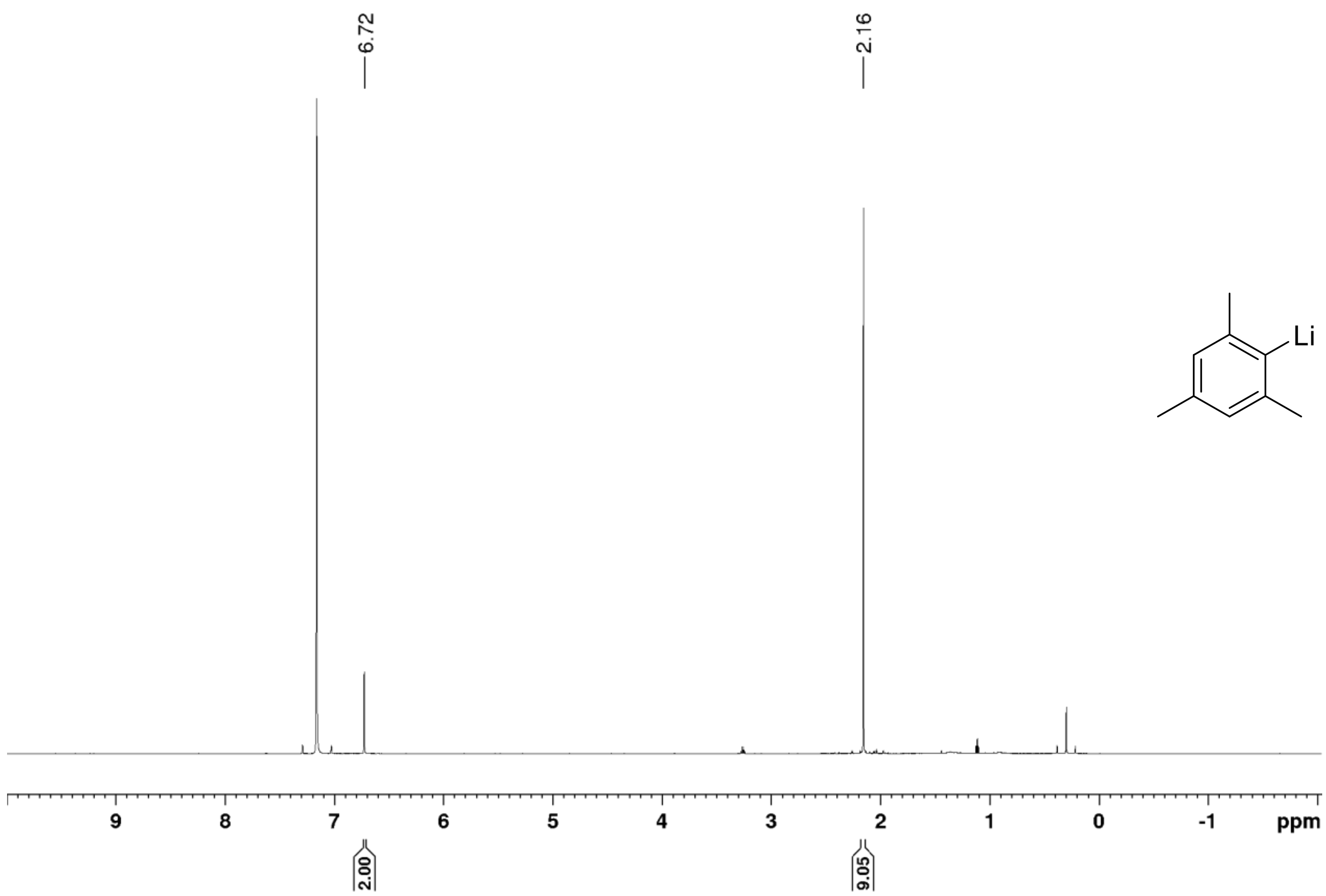

Figure S11. ^{1}H NMR spectrum of mesityl lithium in C_6D_6 measured on a 600 MHz spectrometer.

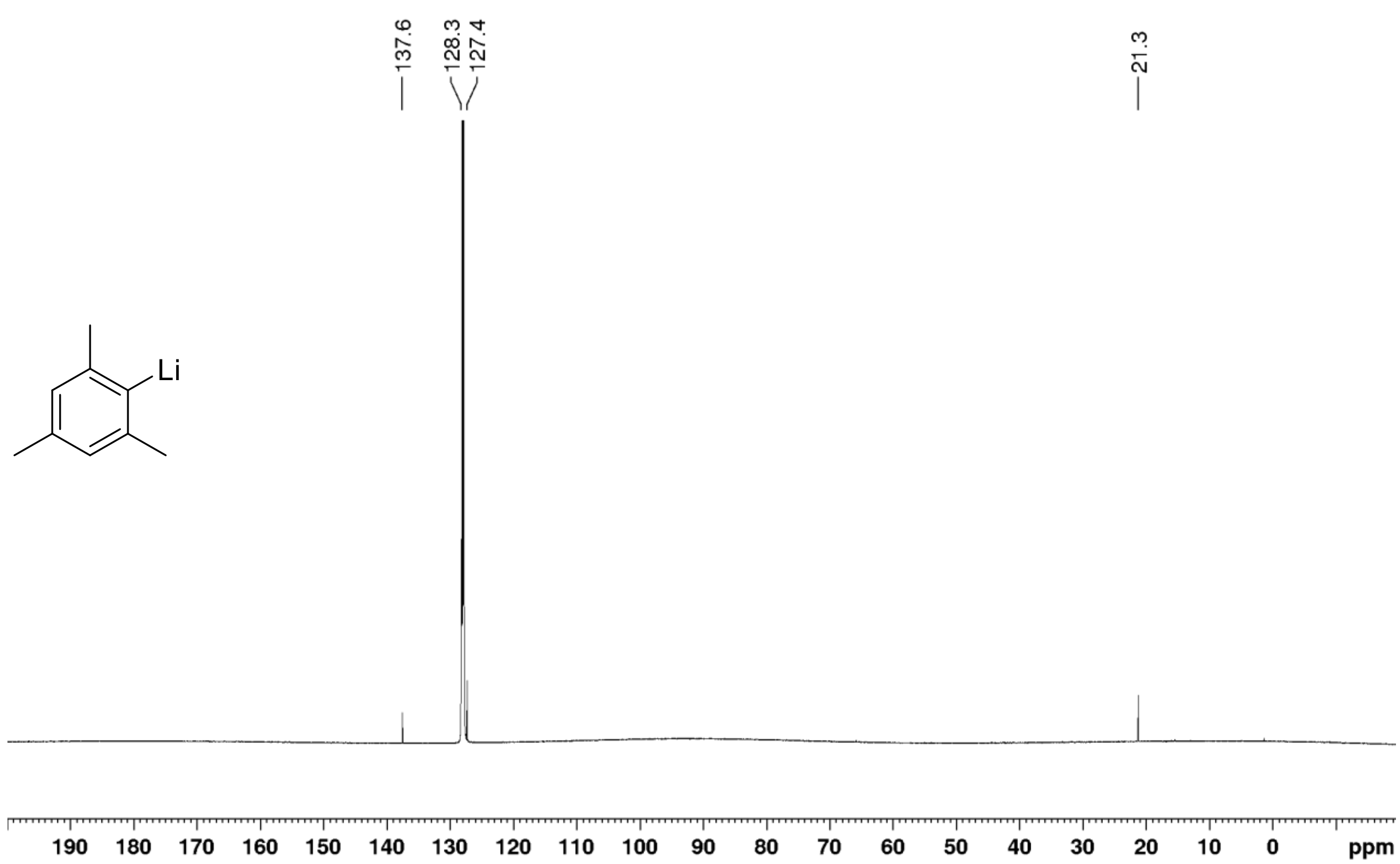

Figure S12. ^{13}C-{^{1}H} NMR spectrum of mesityl lithium in C_6D_6 measured on a 600 MHz spectrometer.

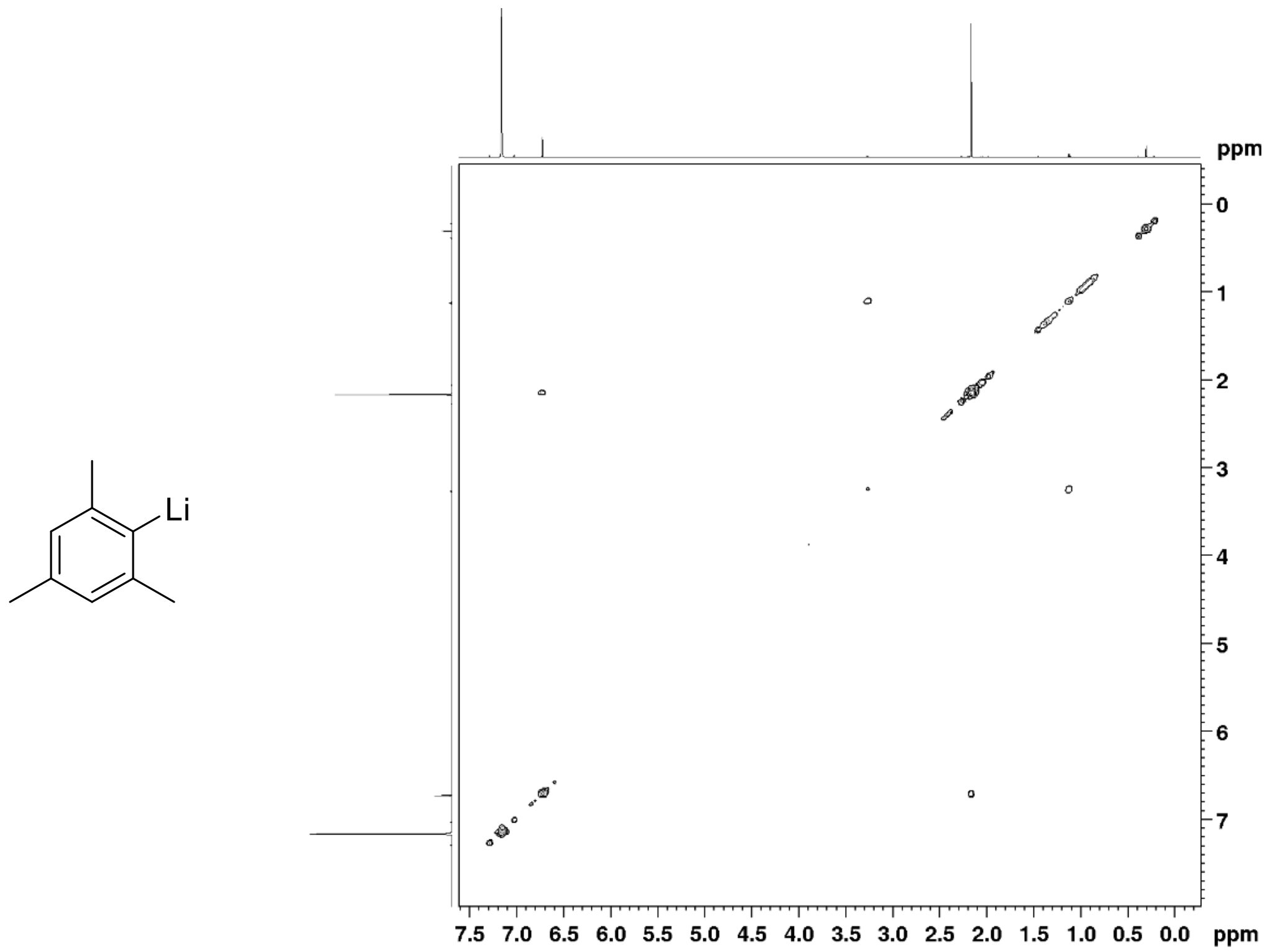

Figure S13. ^{1}H-^{1}H-COSY NMR spectrum of mesityl lithium in C_6D_6 measured on a 600 MHz spectrometer.

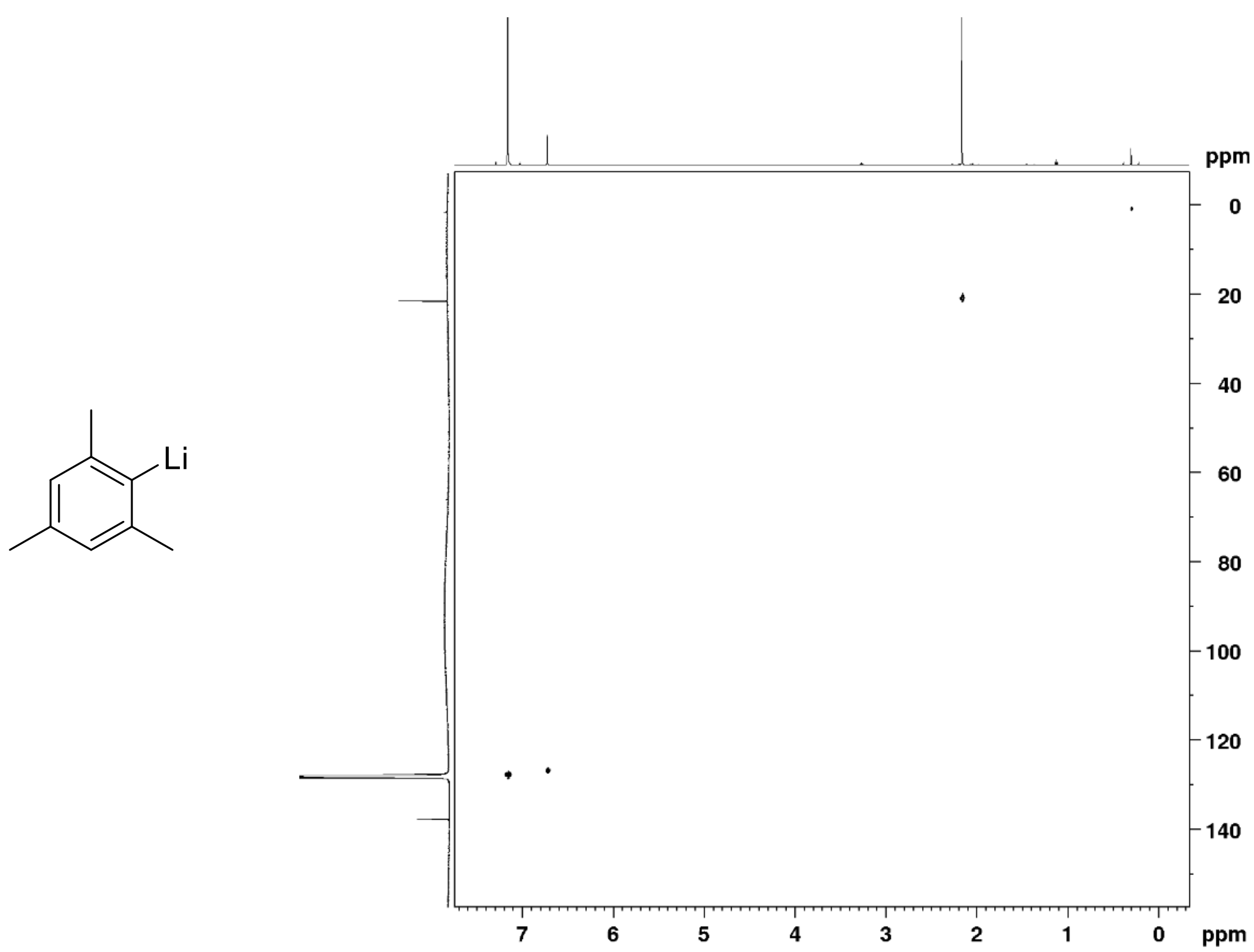

Figure S14. ^{1}H-^{13}C-HSQC NMR spectrum of mesityl lithium in C_6D_6 measured on a 600 MHz spectrometer.

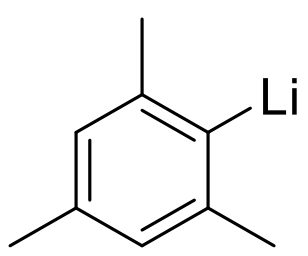

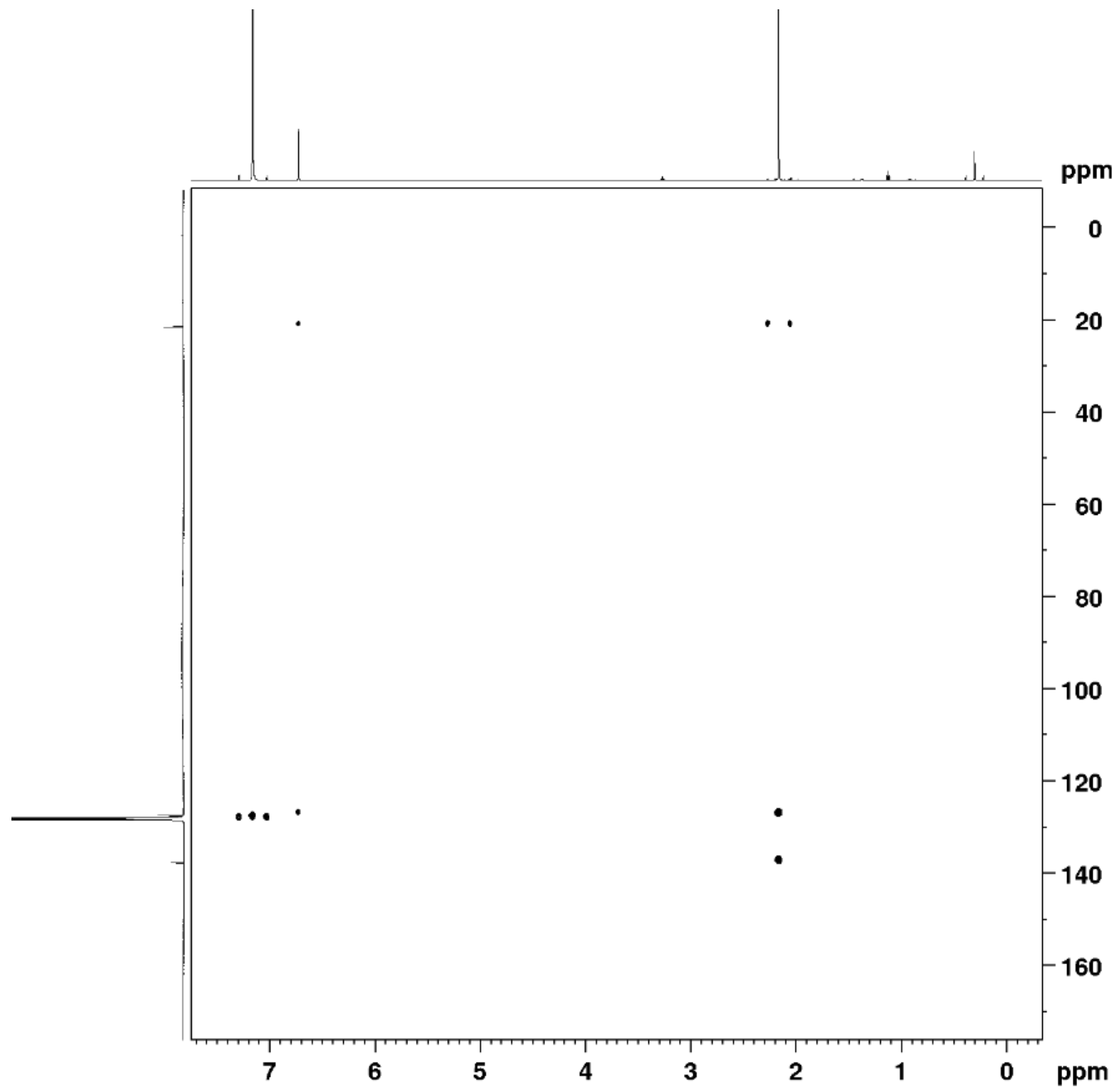

Figure S15. ^{1}H-^{13}C-HMBC NMR spectrum of mesityl lithium in C_6D_6 measured on a 600 MHz spectrometer.

3.1.4 Pentamethylphenyl lithium

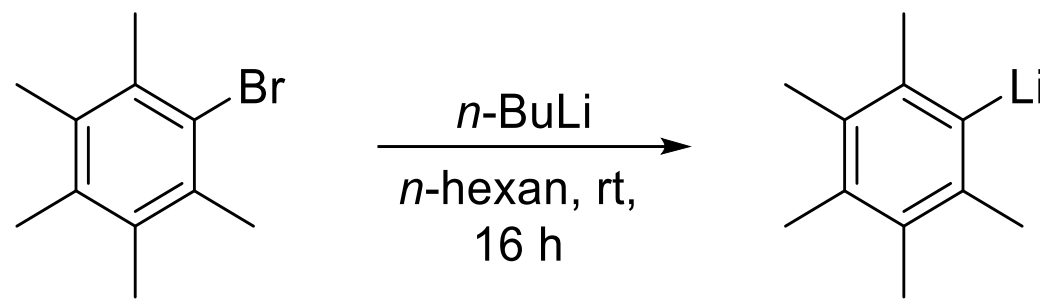

1-Bromo-2,3,4,5,6-pentamethylbenzene (5.0 g, 22 mmol, 1 eq.) was dissolved in dry *n*-hexane (10 mL) and cooled to 0 °C. Under stirring, *n*-butyllithium (2.5 M in *n*-hexane, 9.7 mL, 24 mmol, 1.1 eq.) was added dropwise. The reaction mixture was allowed to warm to room temperature and stirred overnight. The reaction mixture was filtered through an inert-gas frit, and the residue was washed with *n*-hexane (2 × 10 mL) and dried *in vacuo*. The product was obtained as a white solid (2.6795 g, 17 mmol, 79%).

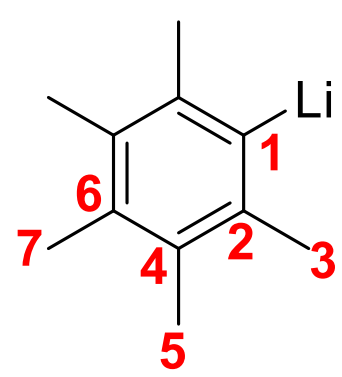

$C_{11}H_{15}Li$ (154.18 g/mol)

^{1}H NMR (600 MHz, **C_6D_6**): δ = 2.16 (s, 6H, H-3), 2.07 (s, 3H, H-7), 2.04 (s, 6H, H-5) ppm.

^{13}C-{^{1}H} NMR (101 MHz, **C_6D_6**): δ = 134.2, 132.7, 131.8, 129.2, 20.4, 15.9, 15.6 ppm.

HRMS measurements were not possible owing to the pronounced air- and moisture-sensitivity of the compound.

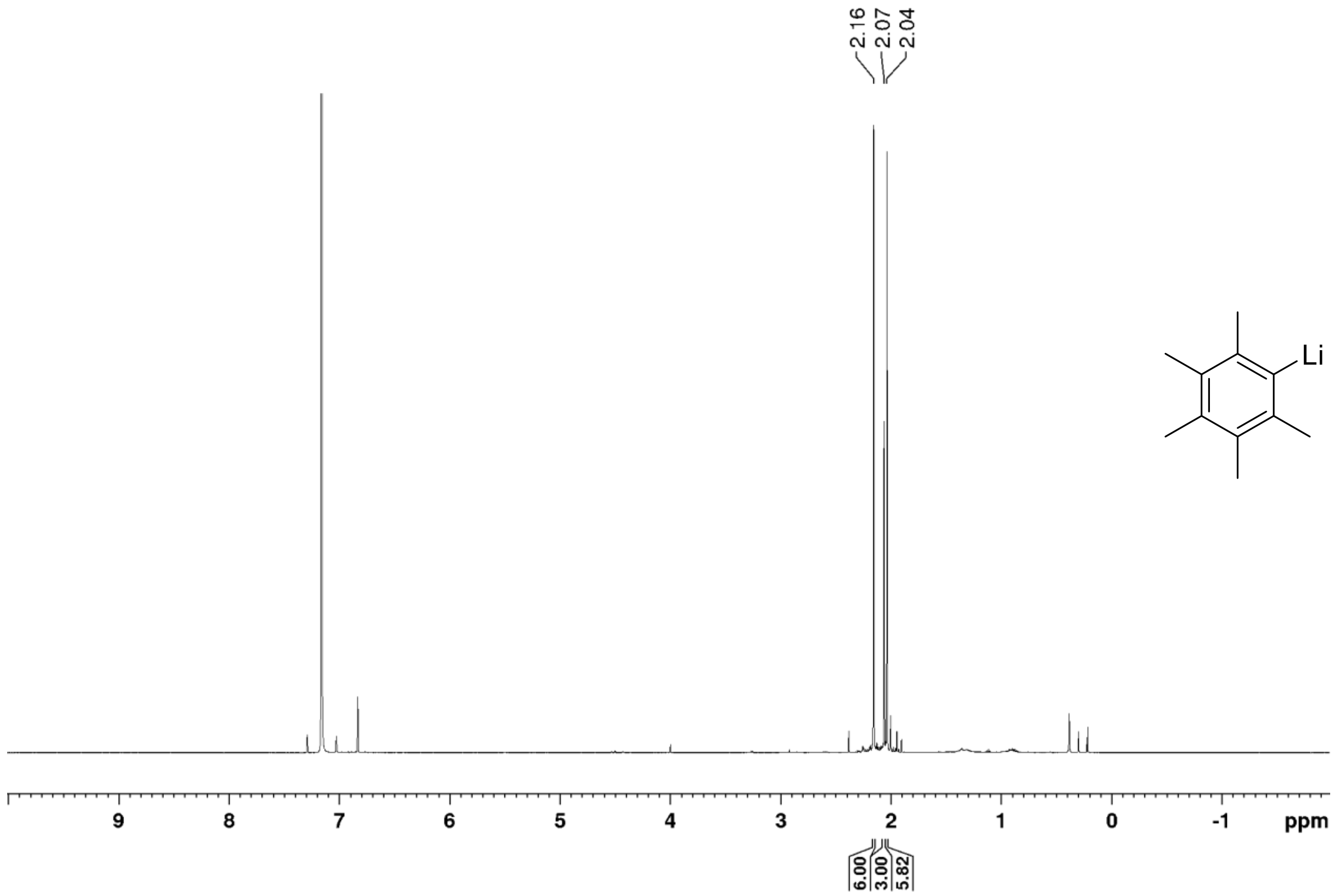

Figure S16. ^{1}H NMR spectrum of pentamethylphenyl lithium in C_6D_6 measured on a 600 MHz spectrometer.

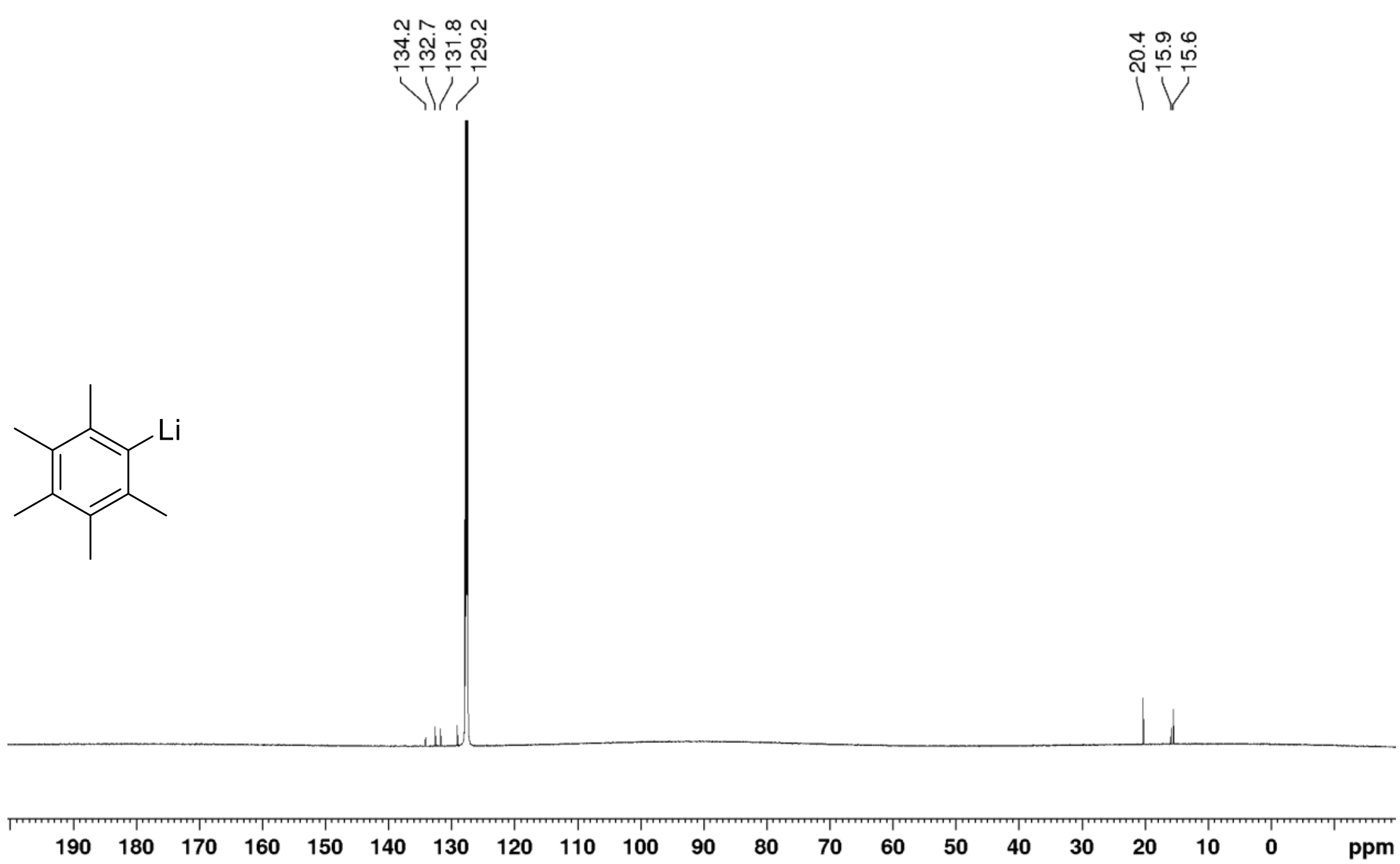

Figure S17. ^{13}C-{^{1}H} NMR spectrum of pentamethylphenyl lithium in C_6D_6 measured on a 600 MHz spectrometer.

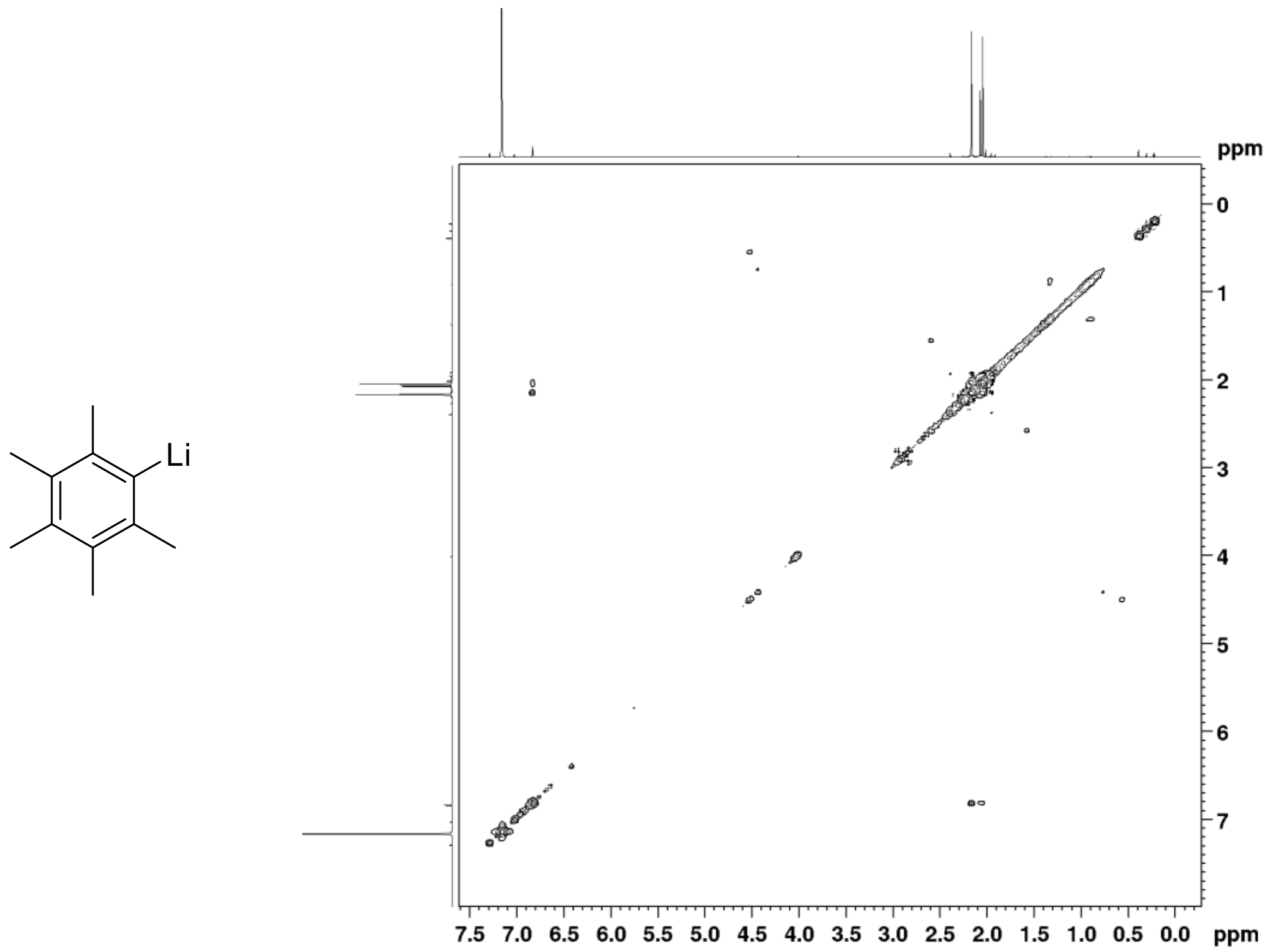

Figure S18. ^{1}H-^{1}H-COSY NMR spectrum of pentamethylphenyl lithium in C_6D_6 measured on a 600 MHz spectrometer.

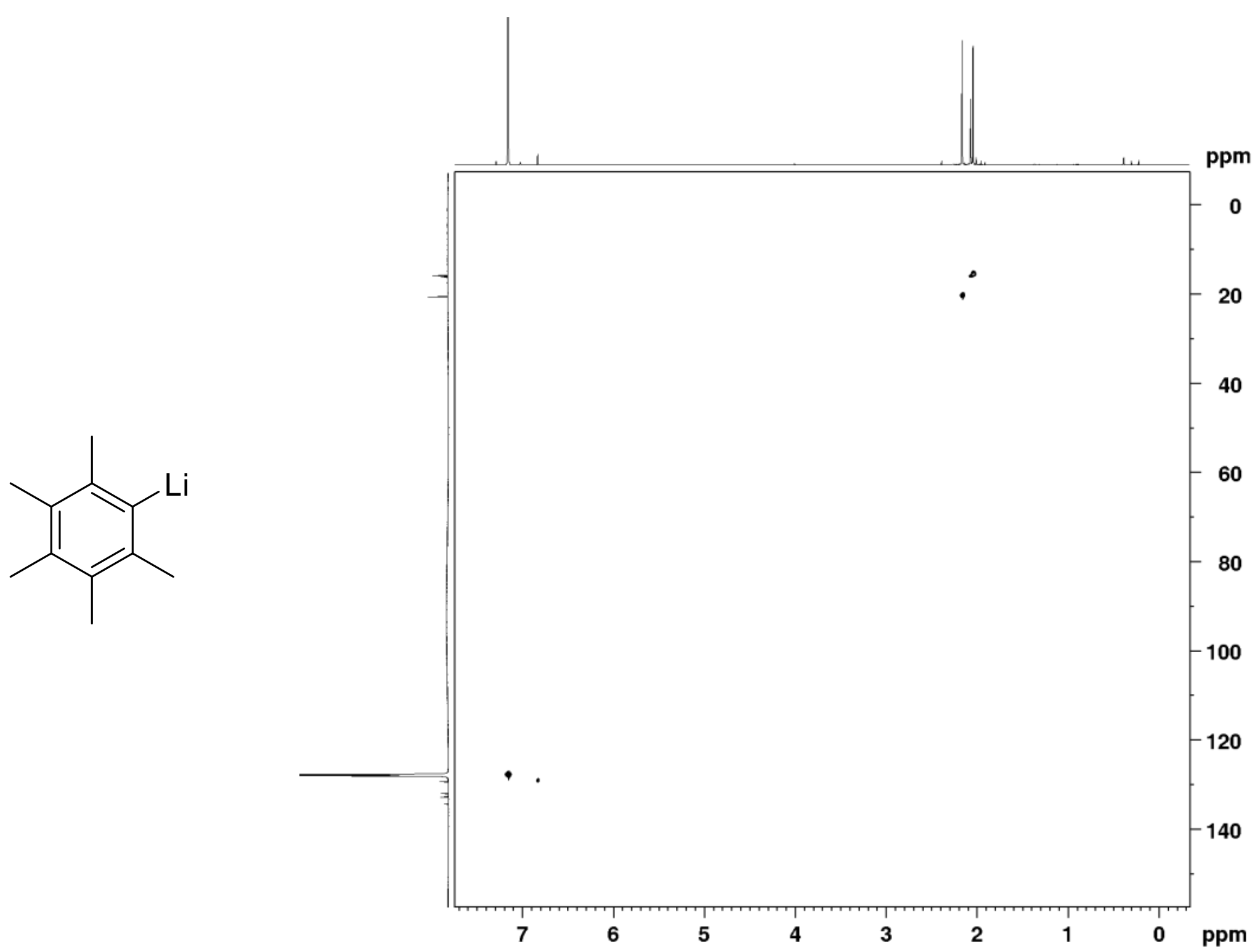

Figure S19. ^{1}H-^{13}C-HSQC NMR spectrum of pentamethylphenyl lithium in C_6D_6 measured on a 600 MHz spectrometer.

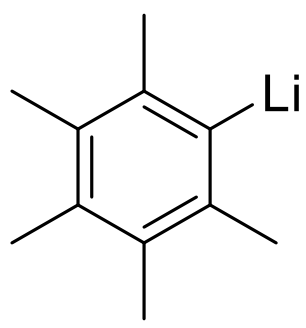

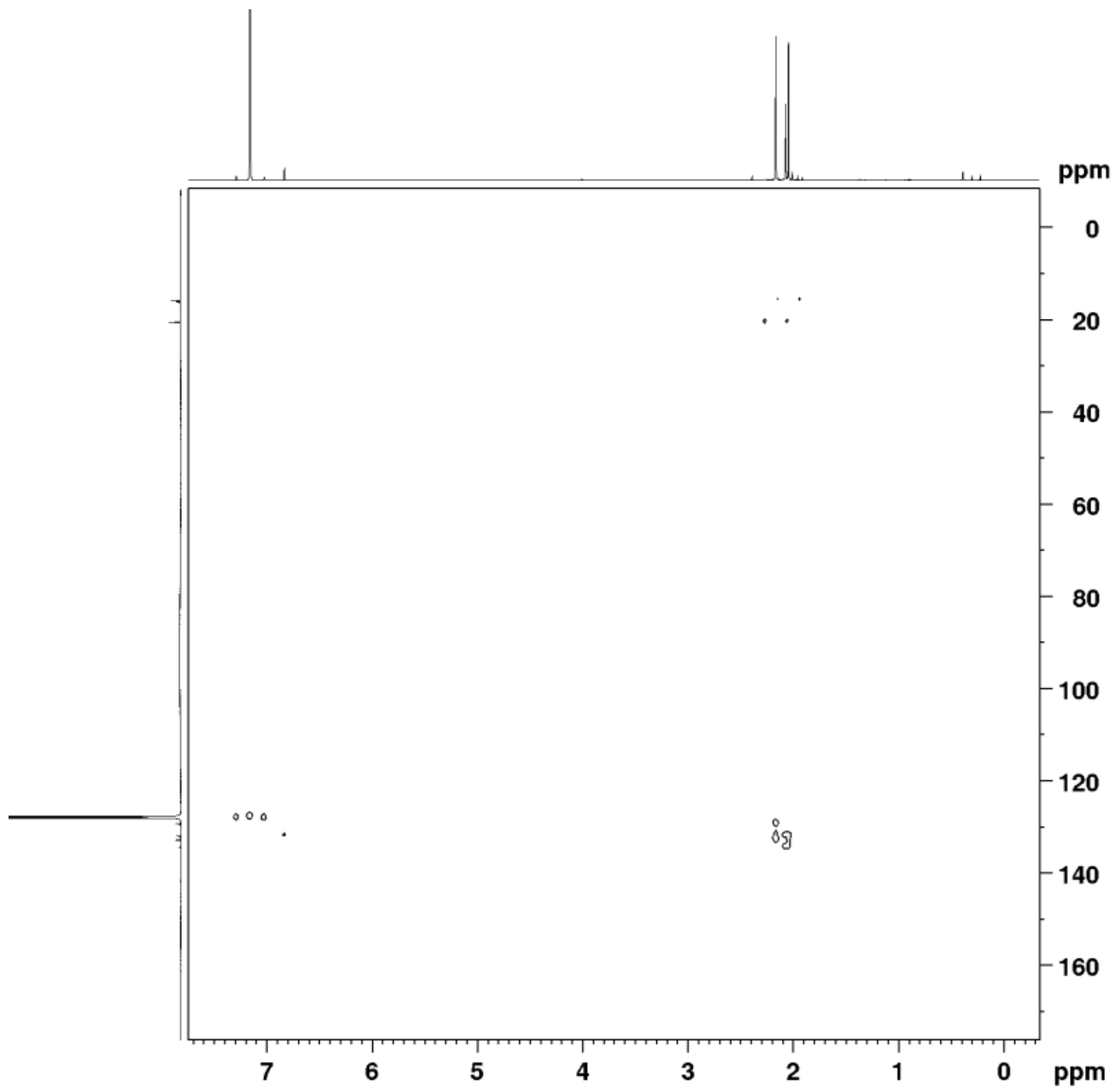

Figure S20. ^{1}H-^{13}C-HMBC NMR spectrum of pentamethylphenyl lithium in C_6D_6 measured on a 600 MHz spectrometer

.

3.2 Synthesis of the Dihydroazaborinines

3.2.1 1-(*tert*-Butyldimethylsilyl)-2-mesityl-1,2-dihydro-1,2-azaborinine (^{BN}**B1**)

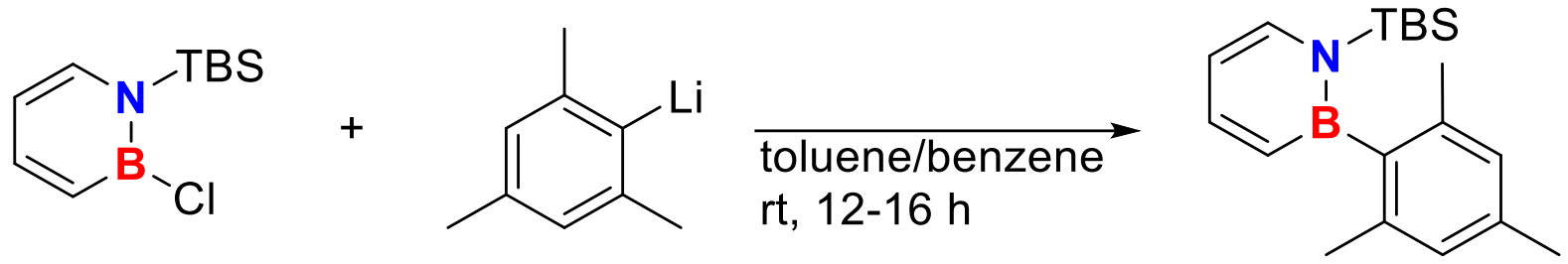

The synthesis is based on procedures by Liu and Bettinger *et al.* [11, 12]

A solution of compound ^{BN}**B2** (2.495 g, 7.4 mmol, 1 eq.) in benzene (15 mL) was slowly treated with mesityl lithium (2.12 g, 12.6 mmol, 1.7 eq.). The reaction mixture was stirred at room temperature for 12 h. In the case of incomplete conversion, the suspension was heated to 60 °C for 30–180 min and only then quenched with distilled water (10 mL). After extraction of the aqueous phase with *n*-hexane (3 × 10 mL), the combined organic phases were washed with saturated aqueous sodium chloride solution (2 × 40 mL) and dried over magnesium sulfate. After removal of the solvent, the crude product was obtained as a pale yellow, amorphous solid and purified by column chromatography (silica gel, *n*-hexane/CH_2Cl_2 gradient from 100:0 to 95:5). Compound ^{BN}**B1** was isolated as a colorless, crystalline solid in a yield of 1.9937 g (6.4 mmol, 87%).

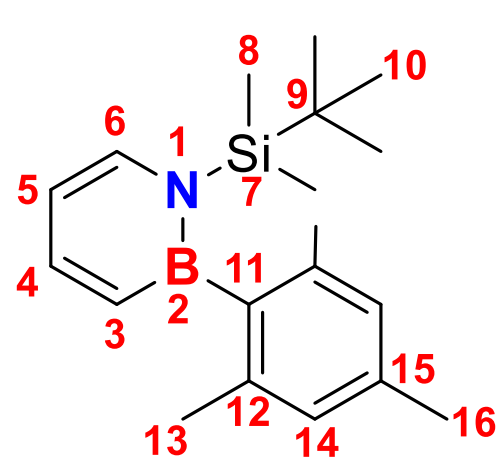

$C_{19}H_{30}BNSi$ (311.35 g/mol)

^{1}H NMR (400 MHz, **CD_2Cl_2**): δ = 0.00 (s, 6H, H-8), 0.91 (s, 9H, H-10), 2.06 (s, 6H, H-13), 2.26 (s, 3H, H-16), 6.39 (ps. dt, 1H, H-5), 6.56 (dd, $^3J_{HH}$ = 11.07 Hz, $^4J_{HH}$ = 0.99 Hz, 1H, H-3), 6.77 (m, 2H, H-14), 7.48 (d, $^3J_{HH}$ = 6.65 Hz, 1H, H-6), 7.56 (ddd, $^3J_{HH}$ = 10.91 Hz, $^3J_{HH}$ = 6.29 Hz, $^4J_{HH}$ = 1.08 Hz, 1H, H-4) ppm.

^{13}**C-{^{1}H} NMR** (101 MHz, **CD_2Cl_2**): δ = -3.6 (C8),19.1 (C9), 20.8 (C16), 23.2 (C13), 27.2 (C10), 111.3 (C5), 127.7 (C14), 131.8 (s. br., C3), 136.2 (C15), 138.4 (C6), 138.6 (C12), 141.8 (C11, s. br.), 142.6 (C4) ppm.

^{11}**B-{^{1}H} NMR** (193 MHz, **CD_2Cl_2**): δ = 39.9 ppm.

^{1}**H NMR** (400 MHz, **TCE-d_2**): δ = -0.09 (s, 6H, H-8), 0.82 (s, 9H, H-10), 2.00 (s, 6H, H-13), 2.21 (s, 3H, H-16), 6.32 (ps. dt, 1H, H-5), 6.56 (dd, $^{3}J_{HH}$ = 11.06 Hz, $^{4}J_{HH}$ = 0.87 Hz, 1H, H-3), 6.69 (m, 2H, H-14), 7.35 (d, $^{3}J_{HH}$ = 6.87 Hz, 1H, H-6), 7.49 (ddd, $^{3}J_{HH}$ = 10.71 Hz, $^{3}J_{HH}$ = 6.26 Hz, $^{4}J_{HH}$ = 1.15 Hz, 1H, H-4) ppm

^{11}**B-{^{1}H} NMR** (128 MHz, **TCE-d_2**): δ = 40.7 ppm.

The obtained signals are in agreement with previously published data.[12]

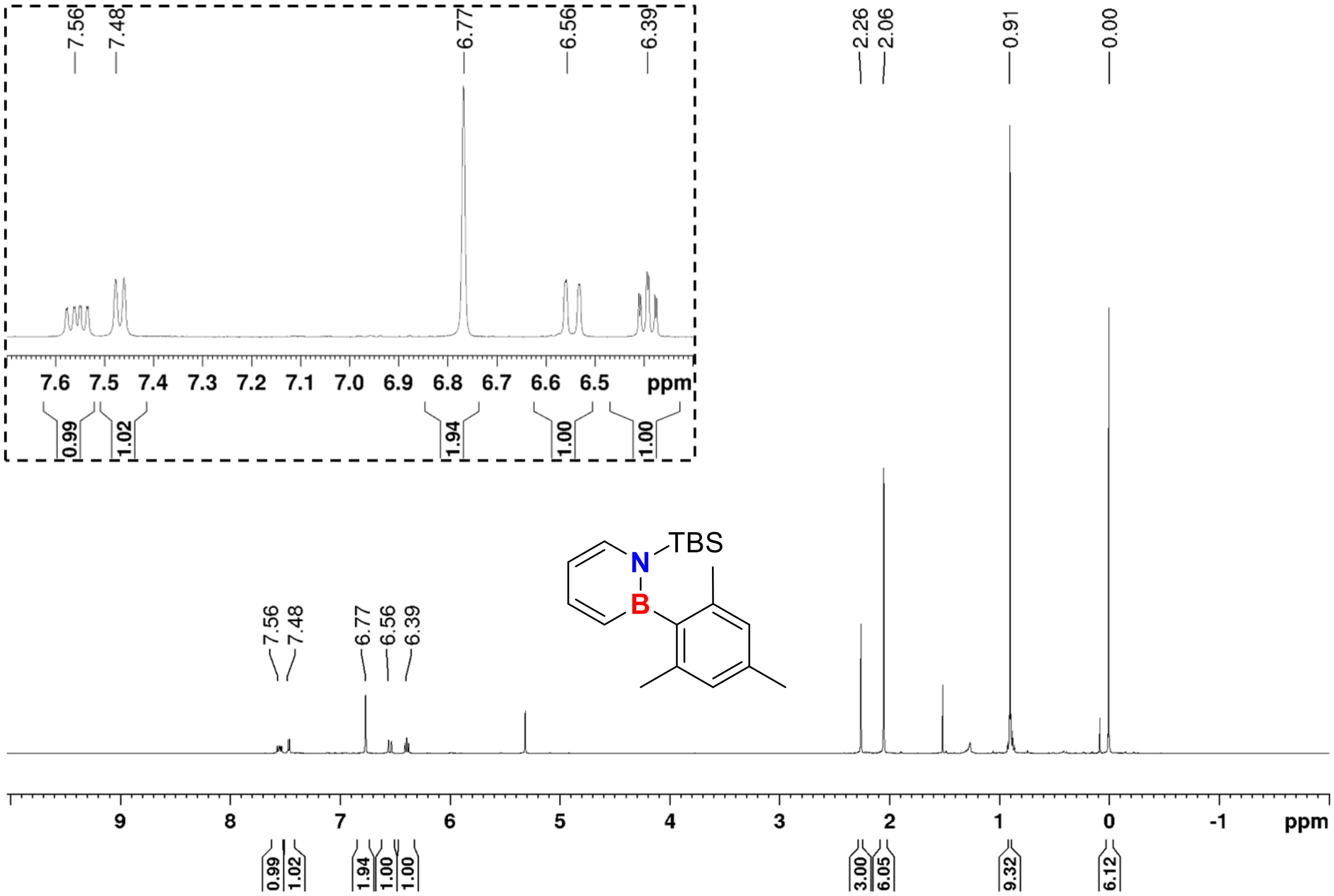

Figure S21. ^{1}H NMR spectrum of ^{BN}**B1** in C_6D_{12} measured on a 600 MHz spectrometer.

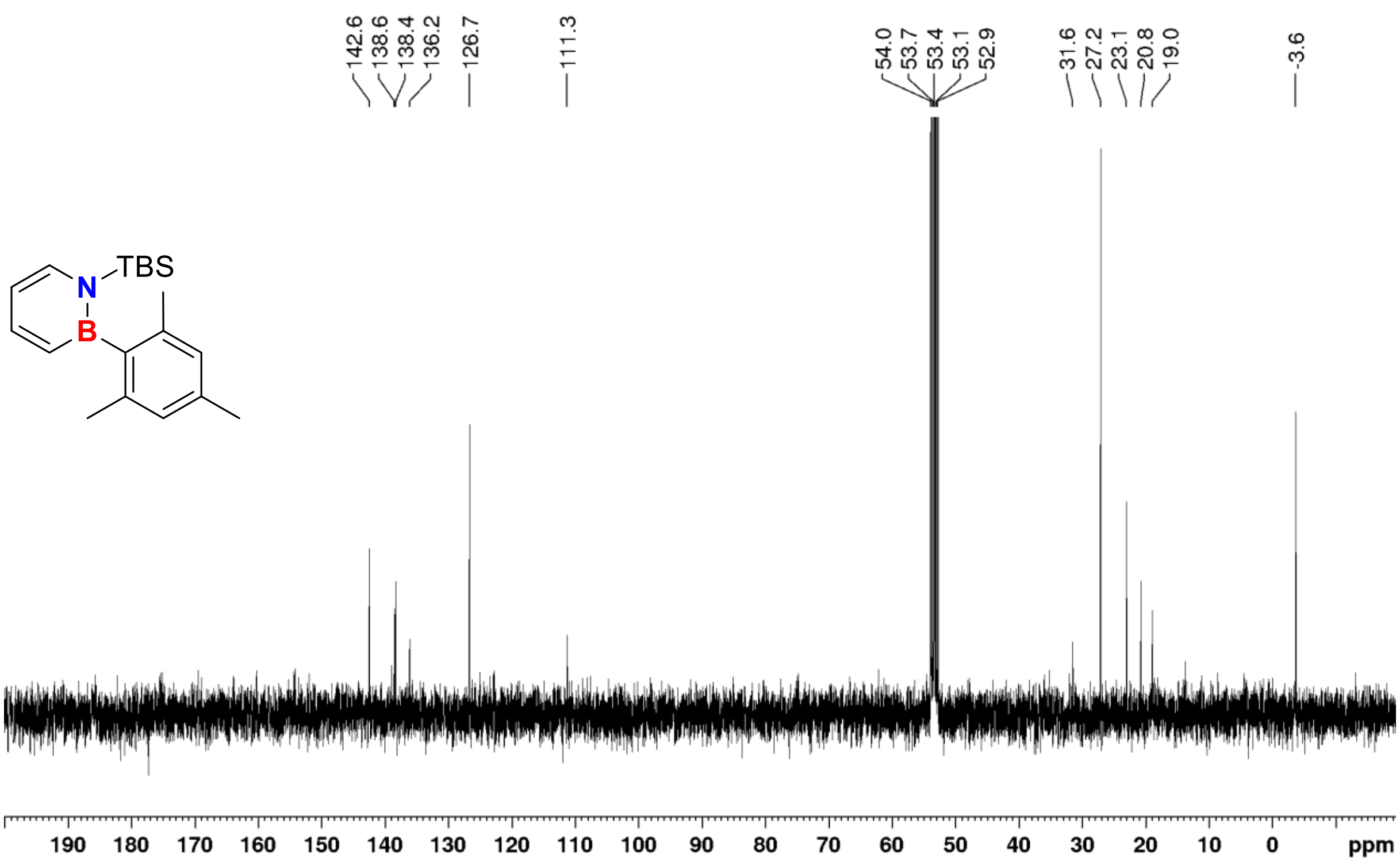

Figure S22. ^{13}C-{^{1}H} NMR spectrum of ^{BN}**B1** in C_6D_{12} measured on a 600 MHz spectrometer.

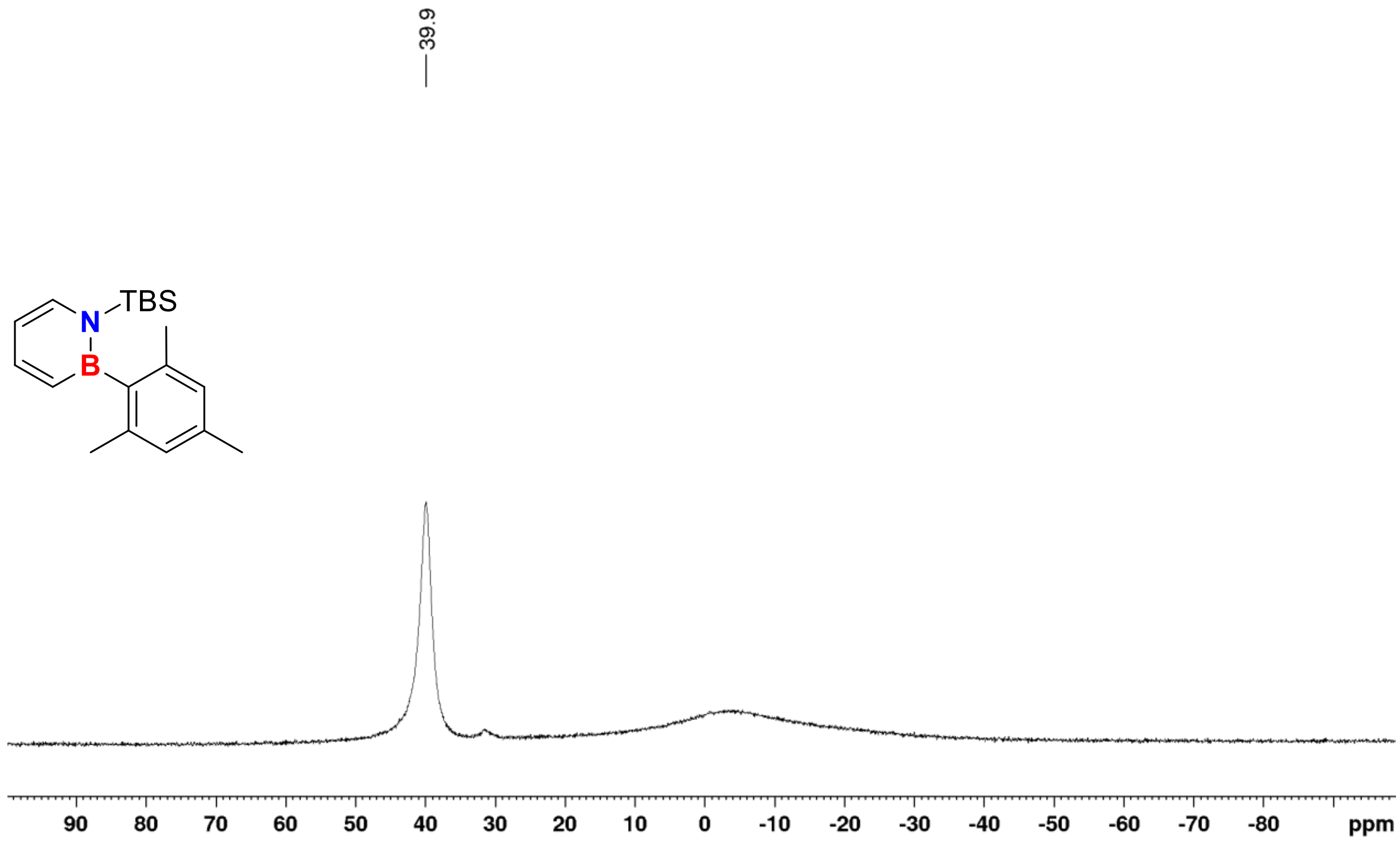

Figure S23. ^{11}B-{^{1}H} NMR spectrum of ^{BN}**B1** in C_6D_{12} measured on a 600 MHz spectrometer.

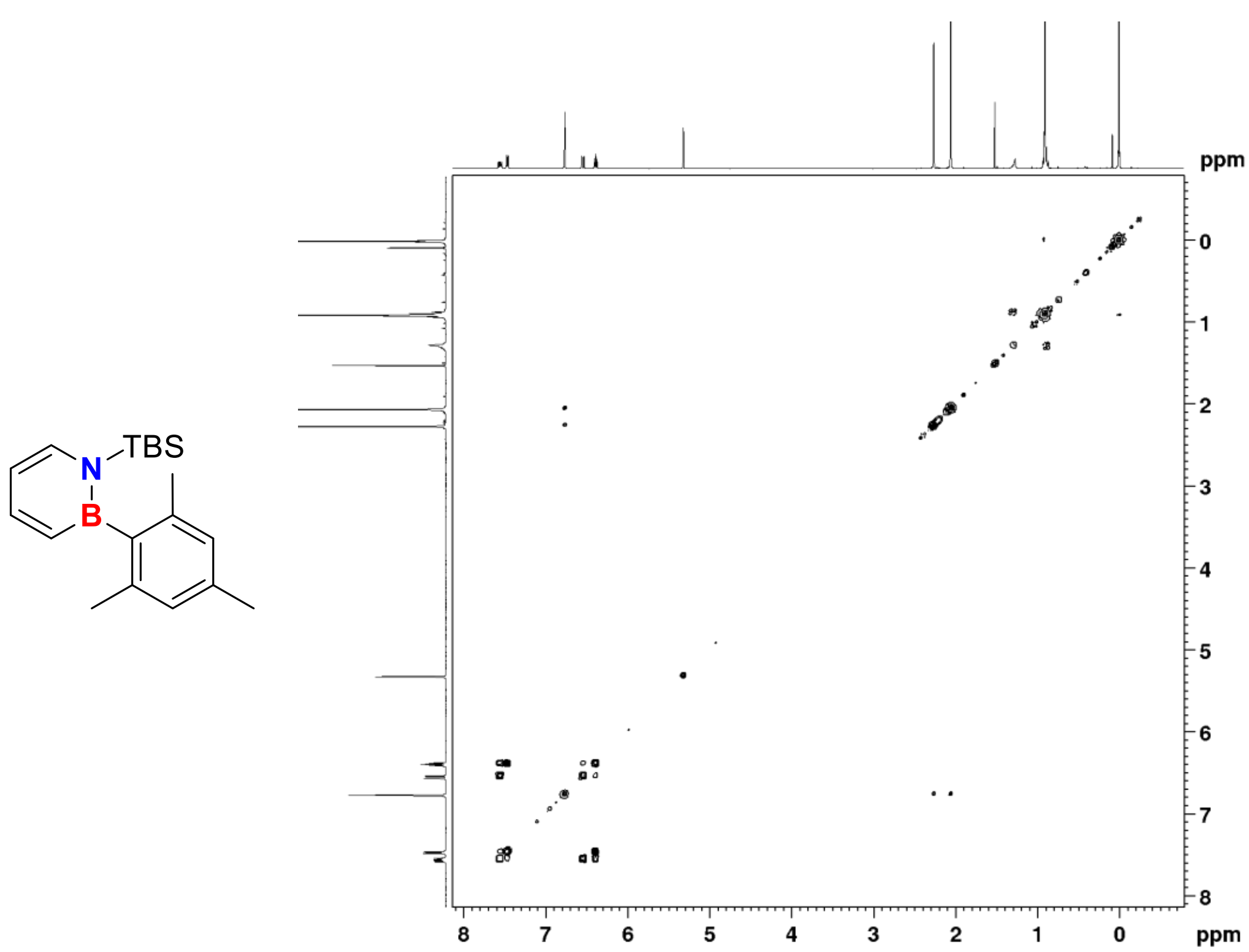

Figure S24. ^{1}H-^{1}H-COSY NMR spectrum of ^{BN}**B1** in C_6D_{12} measured on a 600 MHz spectrometer.

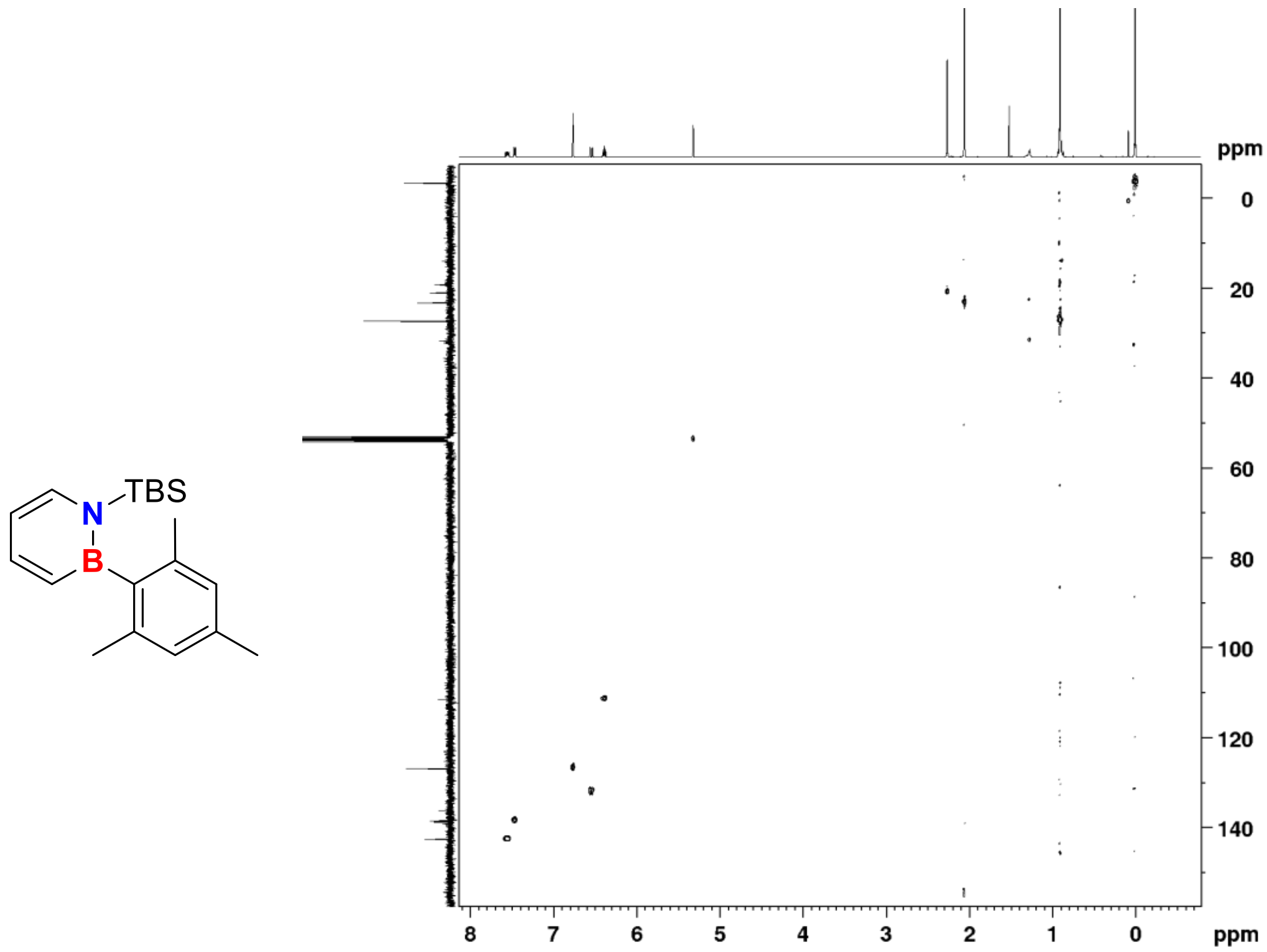

Figure S25. ^{1}H-^{13}C-HSQC NMR spectrum of ***^{BN}*B1** in C_6D_{12} measured on a 600 MHz spectrometer.

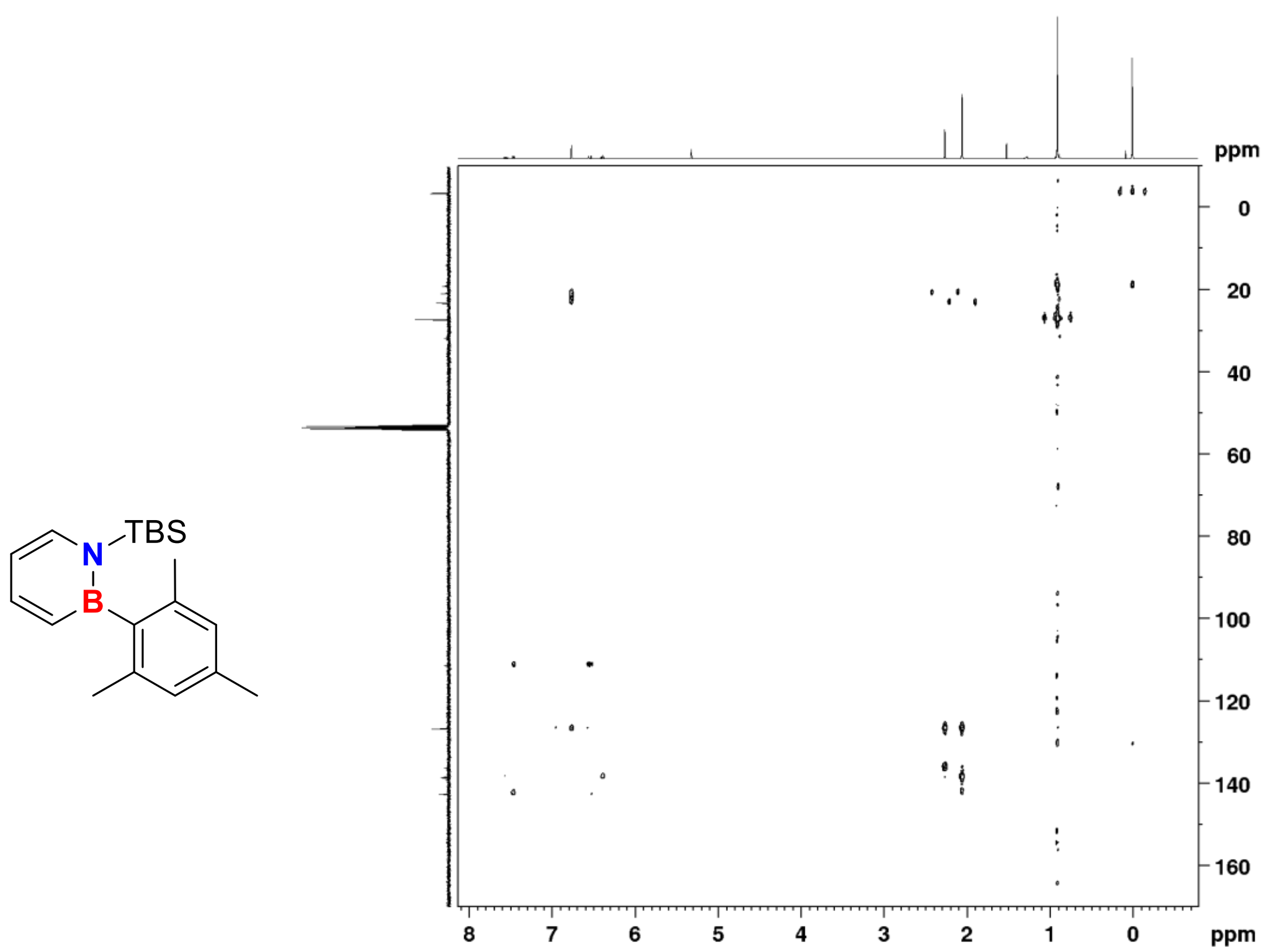

Figure S26. ^{1}H-^{13}C-HMBC NMR spectrum of ***^{BN}*B1** in C_6D_{12} measured on a 600 MHz spectrometer.

3.2.3 1-(*tert*-Butyldimethylsilyl)-2-(ortho-tolyl)-1,2-dihydro-1,2-azaborinine (^{BN}B3)

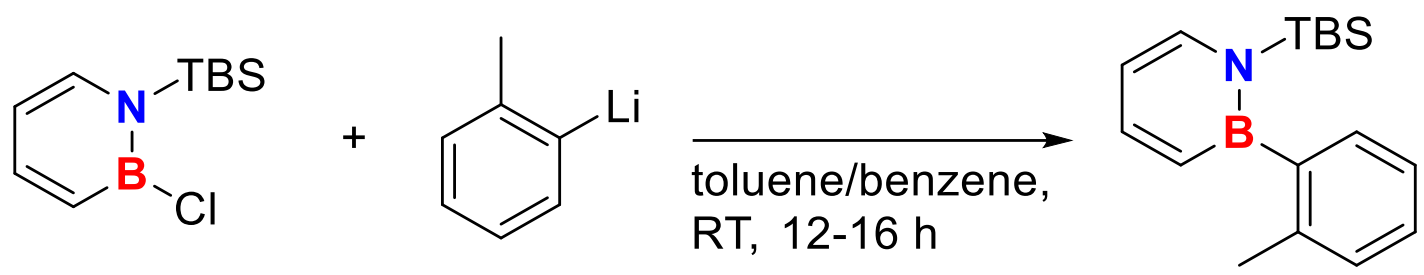

The synthesis is based on a procedure by Edel *et al.*[11]

Dihydroazaborinine ^{BN}**B2** (50 mg, 0.22 mmol, 1 eq.) was dissolved in dry toluene/benzene (2 mL), and 2-tolyllithium (43 mg, 0.44 mmol, 2 eq.) was added portion wise over 30 min. The reaction mixture was stirred at room temperature for 16 h and subsequently quenched with deionized water (5 mL). The aqueous phase was extracted with *n*-hexane (3 × 5 mL). The combined organic phases were dried over magnesium sulfate, filtered, and the solvent was removed under reduced pressure. After purification by column chromatography (SiO_2, *n*-hexane/dichloromethane gradient), the product was obtained as a colorless oil (44.81 mg, 0.16 mmol, 73%).

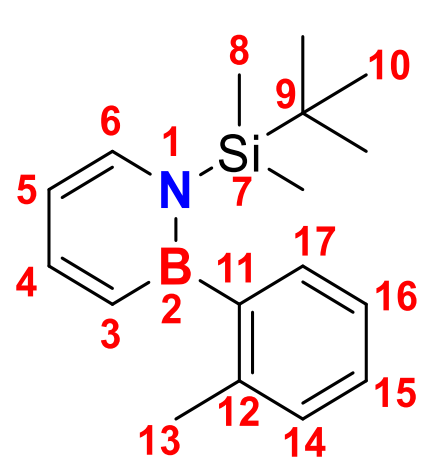

$C_{17}H_{26}BNSi$ (283.30 g/mol)

^{1}H NMR (600 MHz, **C_6D_{12}**): δ = 7.77 (m, 1H, H-4), 7.52 (m, 2H, H-6/H-H14), 7.33 (m, 3H, H-15/H-16/H-17), 7.06 (d, 1H, H-3), 6.54 (ps. t, 1H, H-5), 2.45 (s, 3H, H-13), 1.07 (s, 9H, H-10), 0.35 (s, 3H, H-8), 0.00 (s, 3H, H-8) ppm.

^{13}C-{^{1}H} NMR (151 MHz, **C_6D_{12}**): δ = 145.8, 143.0, 139.0, 137.9, 134.2, 132.6, 131.8, 127.3, 124.3, 112.1, 26.9, 23.3, 18.9, -2.8, -3.4 ppm.

^{11}**B-{^{1}H} NMR** (193 MHz, **C_6D_{12}**): δ = 39.8 ppm.

HRMS (ESI) m/z: [M + H]+ Calcd for C17H26BNSi 284.2000; Found 284.2002.

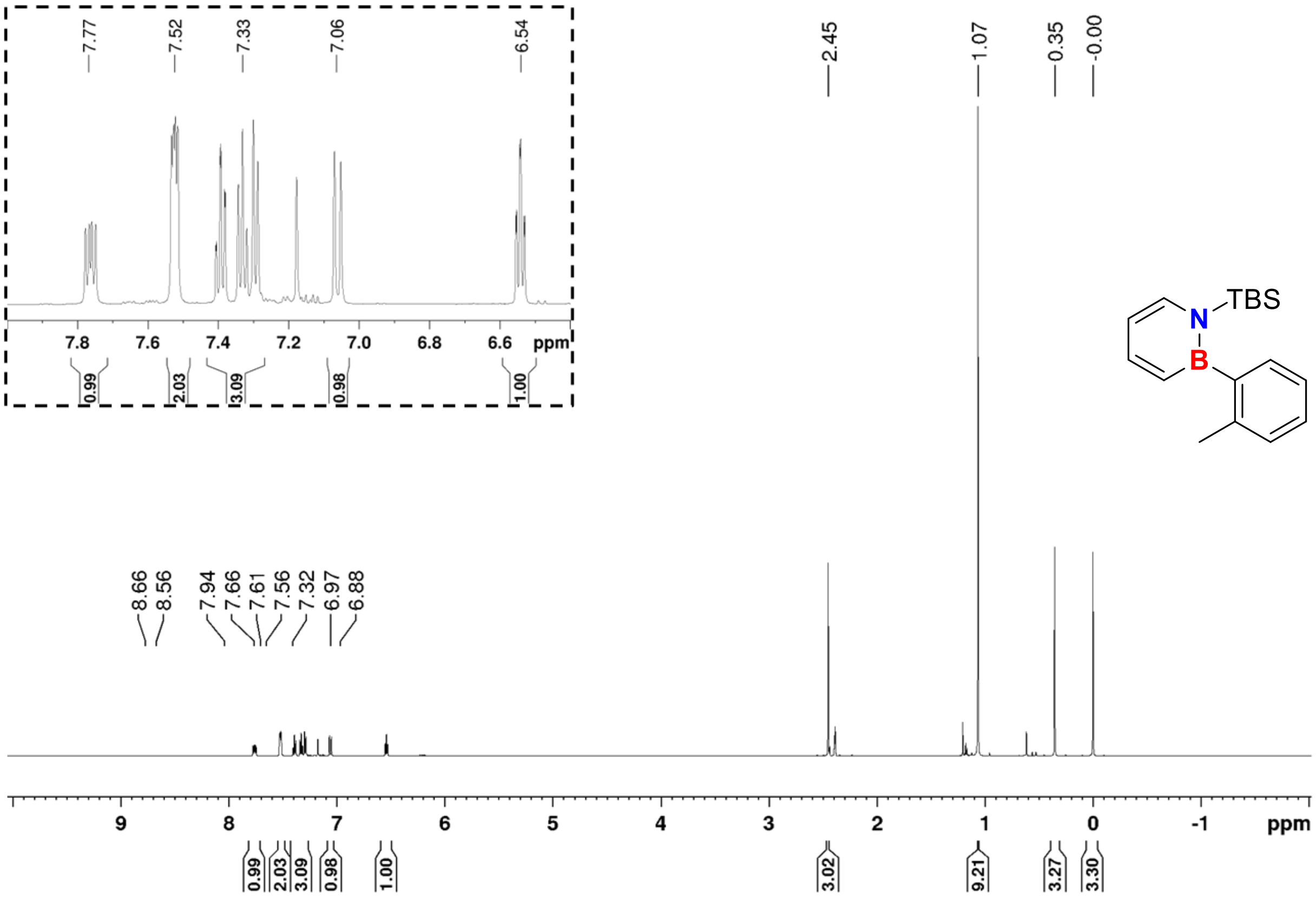

Figure S27. ^{1}H NMR spectrum of ^{BN}**B3** in C_6D_{12} measured on a 600 MHz spectrometer.

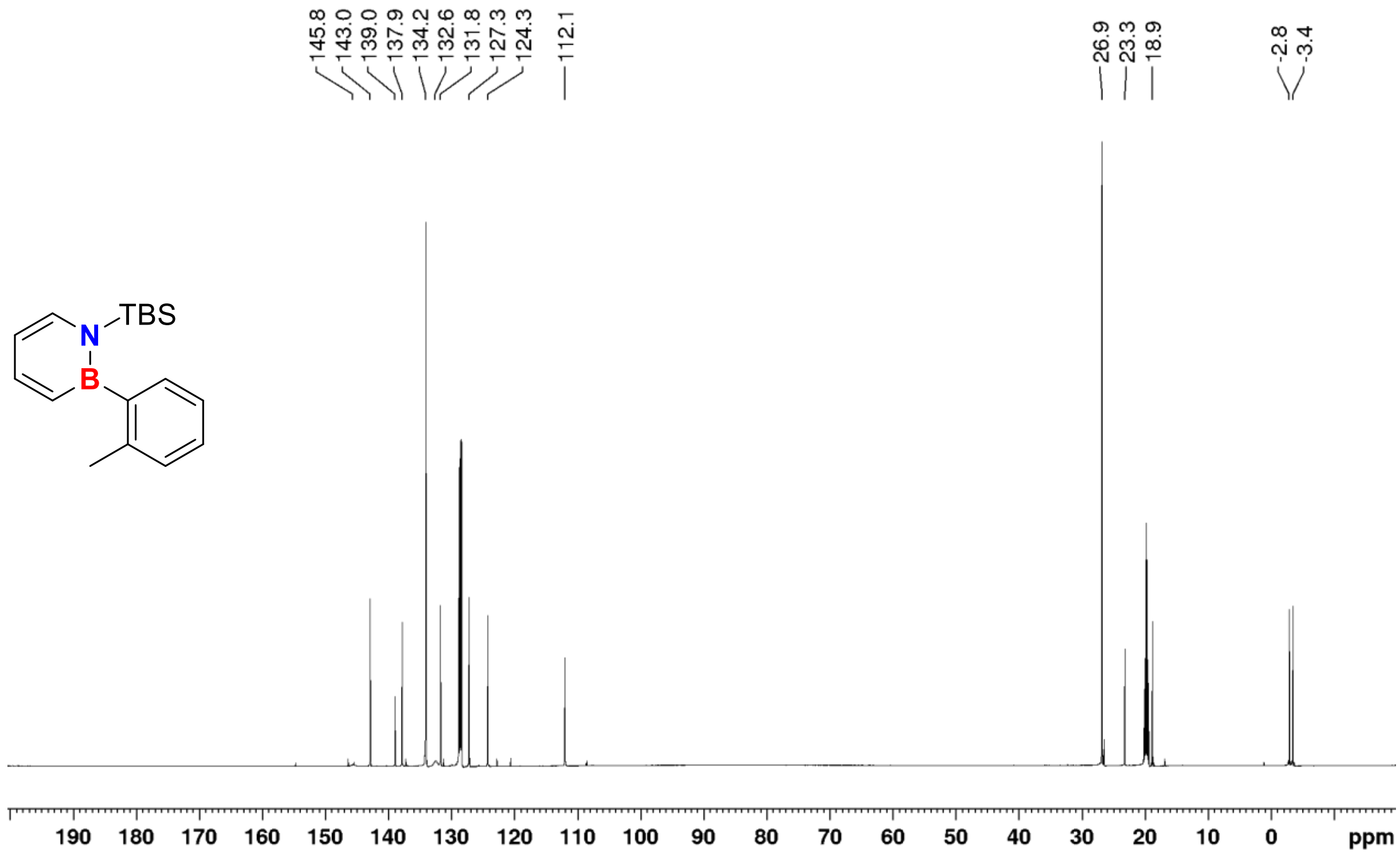

Figure S28. ^{13}C-{^{1}H} NMR spectrum of ***BN*B3** in C_6D_{12} measured on a 600 MHz spectrometer.

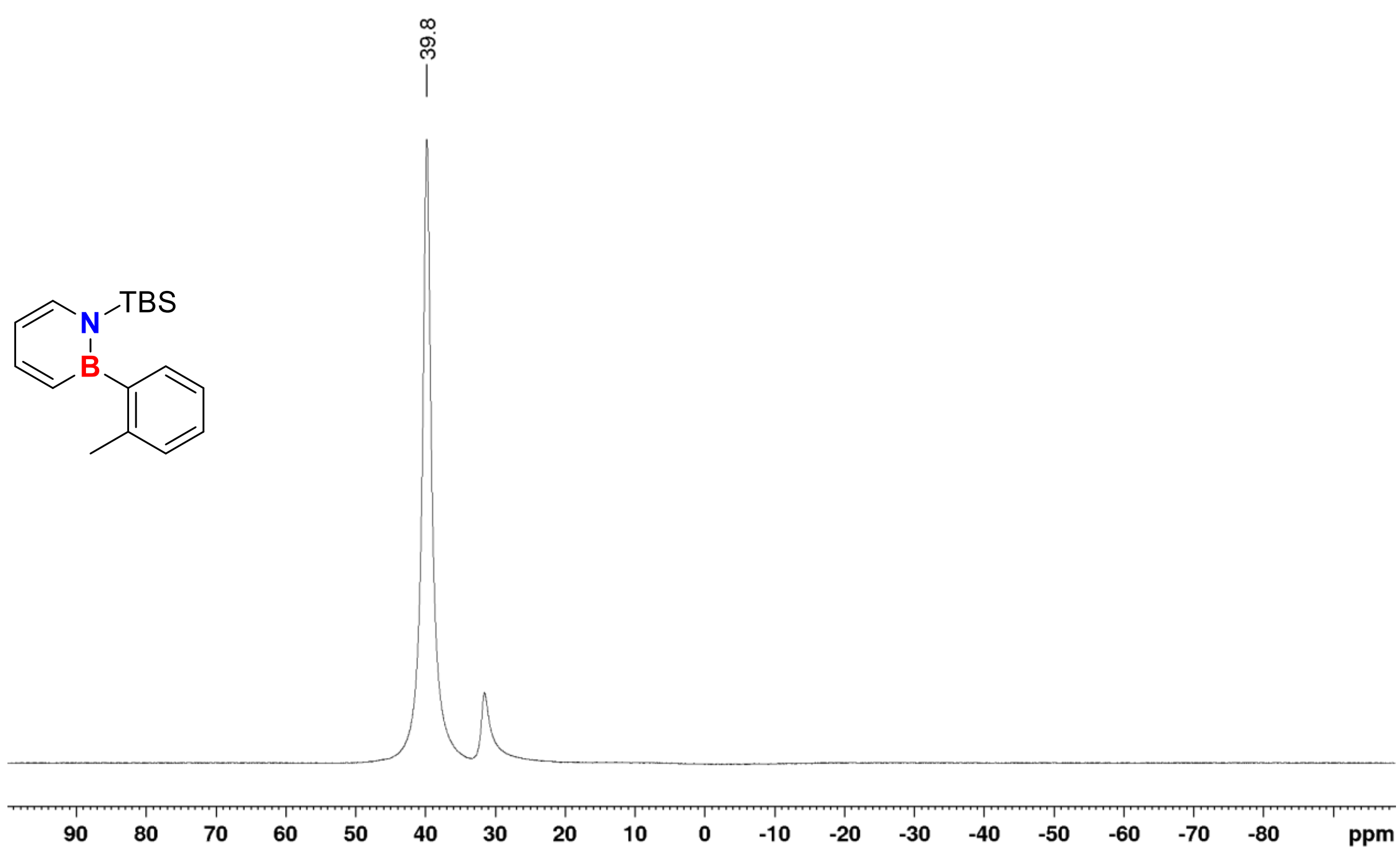

Figure S29. ^{11}B-{^{1}H} NMR spectrum of ***BN*B3** in C_6D_{12} measured on a 600 MHz spectrometer.

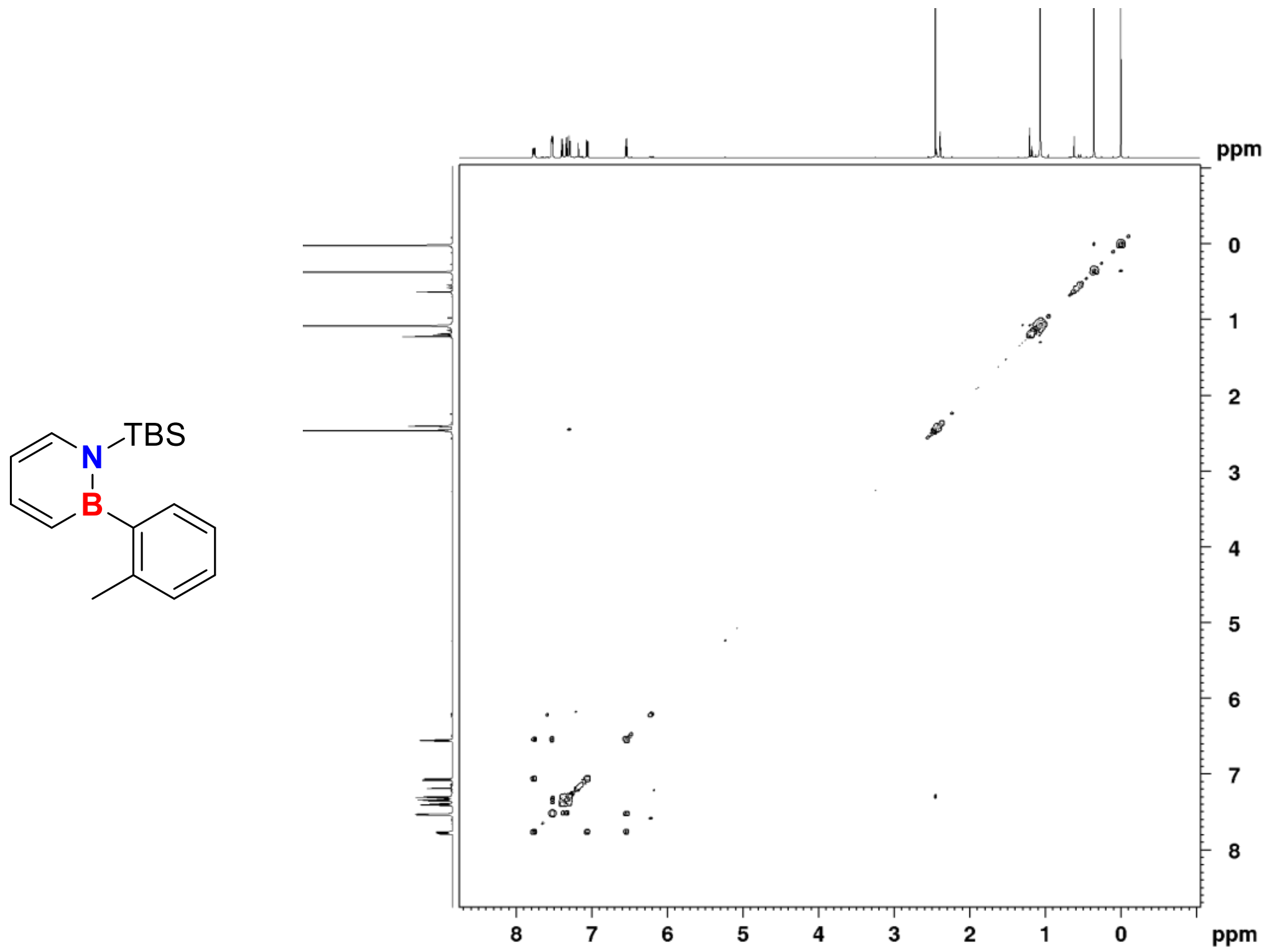

Figure S30. ^{1}H-^{1}H-COSY NMR spectrum of ^{BN}**B3** in C_6D_{12} measured on a 600 MHz spectrometer.

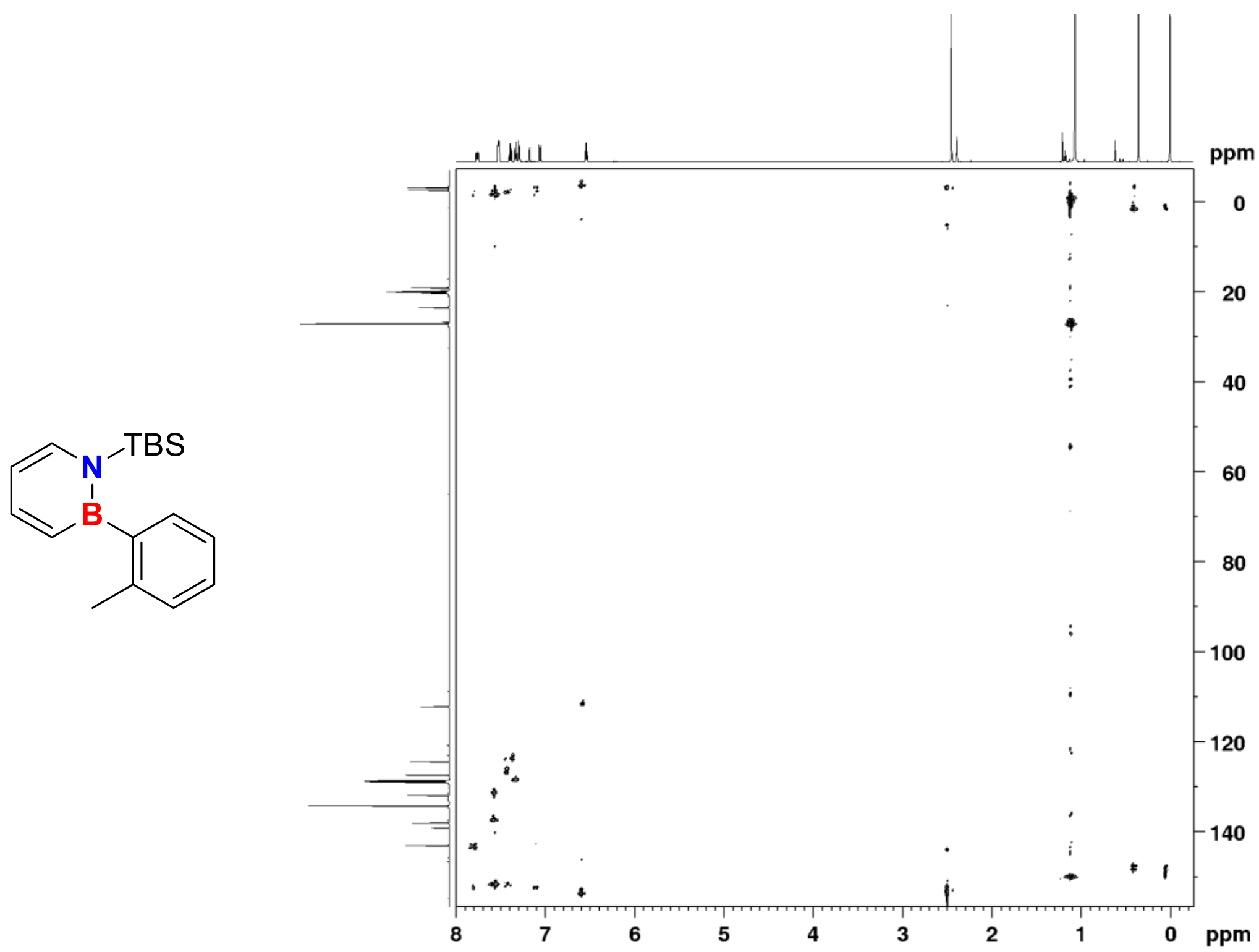

Figure S31. ^{1}H-^{13}C-HSQC NMR spectrum of ^{BN}**B3** in C_6D_{12} measured on a 600 MHz spectrometer.

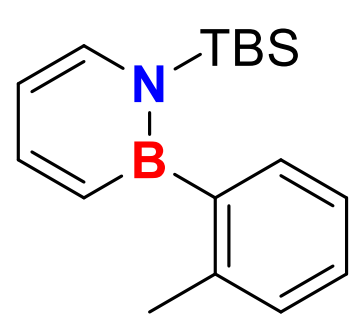

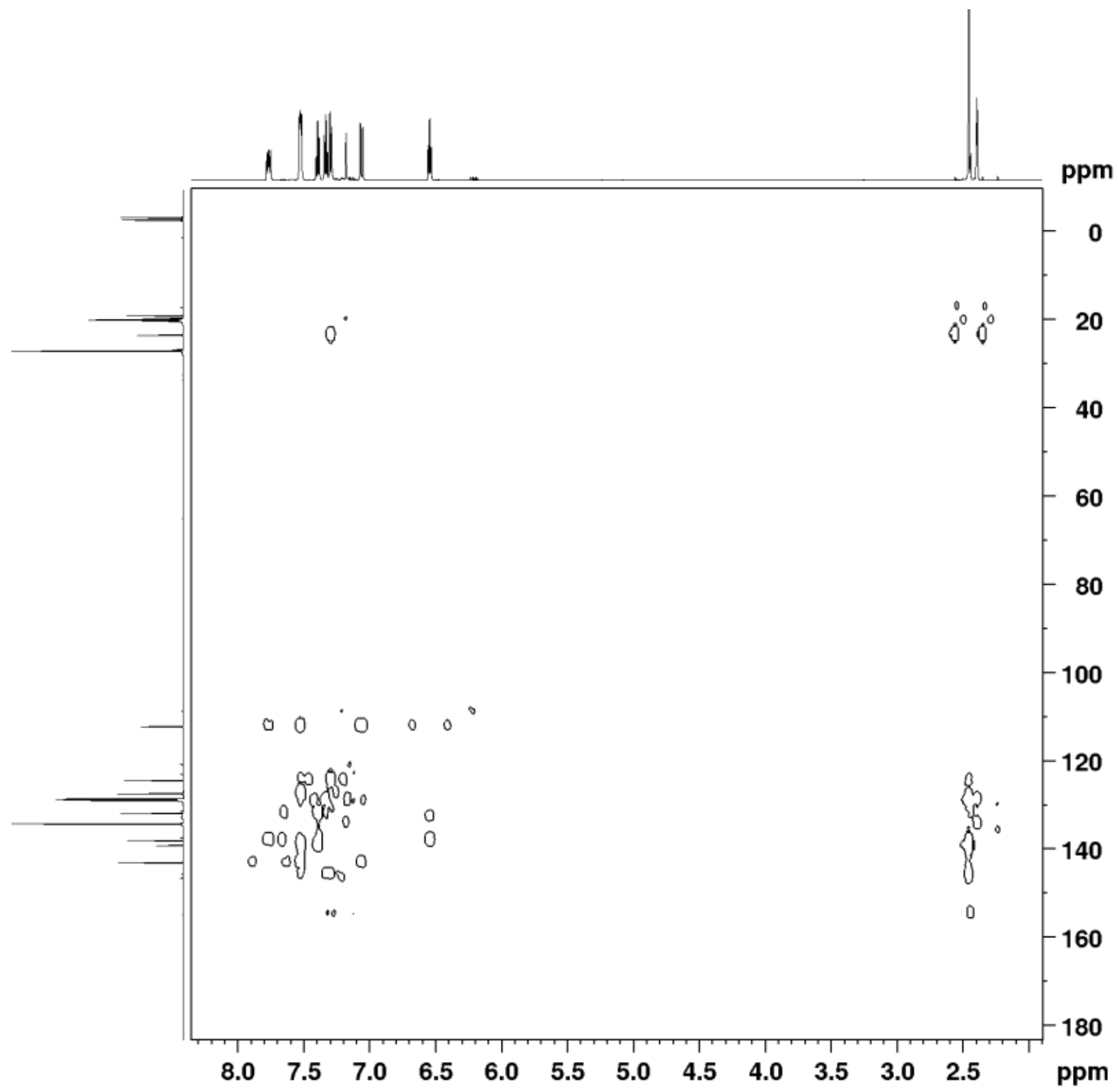

Figure S32. ^{1}H-^{13}C-HMBC NMR spectrum of ^{BN}**B3** in C_6D_{12} measured on a 600 MHz spectrometer.

3.2.2 1-(*tert*-Butyldimethylsilyl)-2-(2-xylyl)-1,2-dihydro-1,2-azaborinine (^{BN}B4)

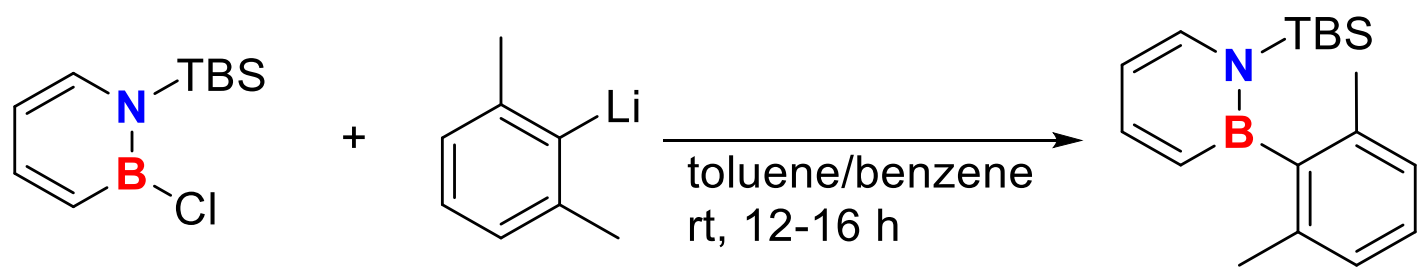

The synthesis is based on a procedure by Edel *et al.*[11]

Dihydroazaborinine ^{BN}**B2** (50 mg, 0.22 mmol, 1.0 eq.) was dissolved in dry toluene/benzene (2 mL), and 2-xylyl lithium (50 mg, 0.44 mmol, 2.0 eq.) was added portion wise over 30 min. The reaction mixture was stirred at room temperature for 16 h and subsequently quenched with deionized water (5 mL). The aqueous phase was extracted with *n*-hexane (3 × 5 mL). The combined organic layers were dried over magnesium sulfate, filtered, and the solvent was removed under reduced pressure. Purification by column chromatography (SiO_2, *n*-hexane/dichloromethane gradient) afforded the product as a colorless oil (55.20 mg, 0.19 mmol, 86%).

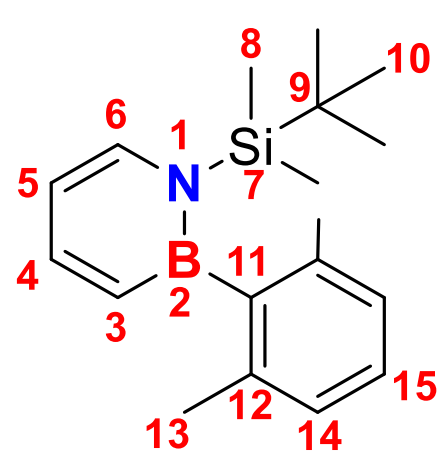

$C_{18}H_{28}BNSi$ (297.32 g/mol)

^{1}H NMR (600 MHz, **C_6D_{12}**): δ = 7.57 (m, 1H, H-4), 7.39 (d, 1H, H-6), 7.17 (ps. t, 1H, H-15), 7.00 (m, 2H, H-14), 6.81 (d, 1H, H-3), 6.34 (ps. t, 1H, H-5), 2.24 (s, 6H, H-13), 0.89 (s, 9H, H-10), H-10), 0.00 (s, 6H, H-8) ppm.

^{13}C-{^{1}H} NMR (151 MHz, **C_6D_{12}**): δ = 145.6, 143.0, 138.6, 138.0, 134.2, 132.4, 132.3, 127.4, 126.4, 111.9, 27.3, 23.6, 19.2, -3.5 ppm.

^{11}B-{^{1}H} NMR (193 MHz, **C_6D_{12}**): δ = 40.3 ppm.

HRMS (APCI) m/z: [M + H]+ Calcd for C18H28BNSi 298.2160; Found 298.2165.

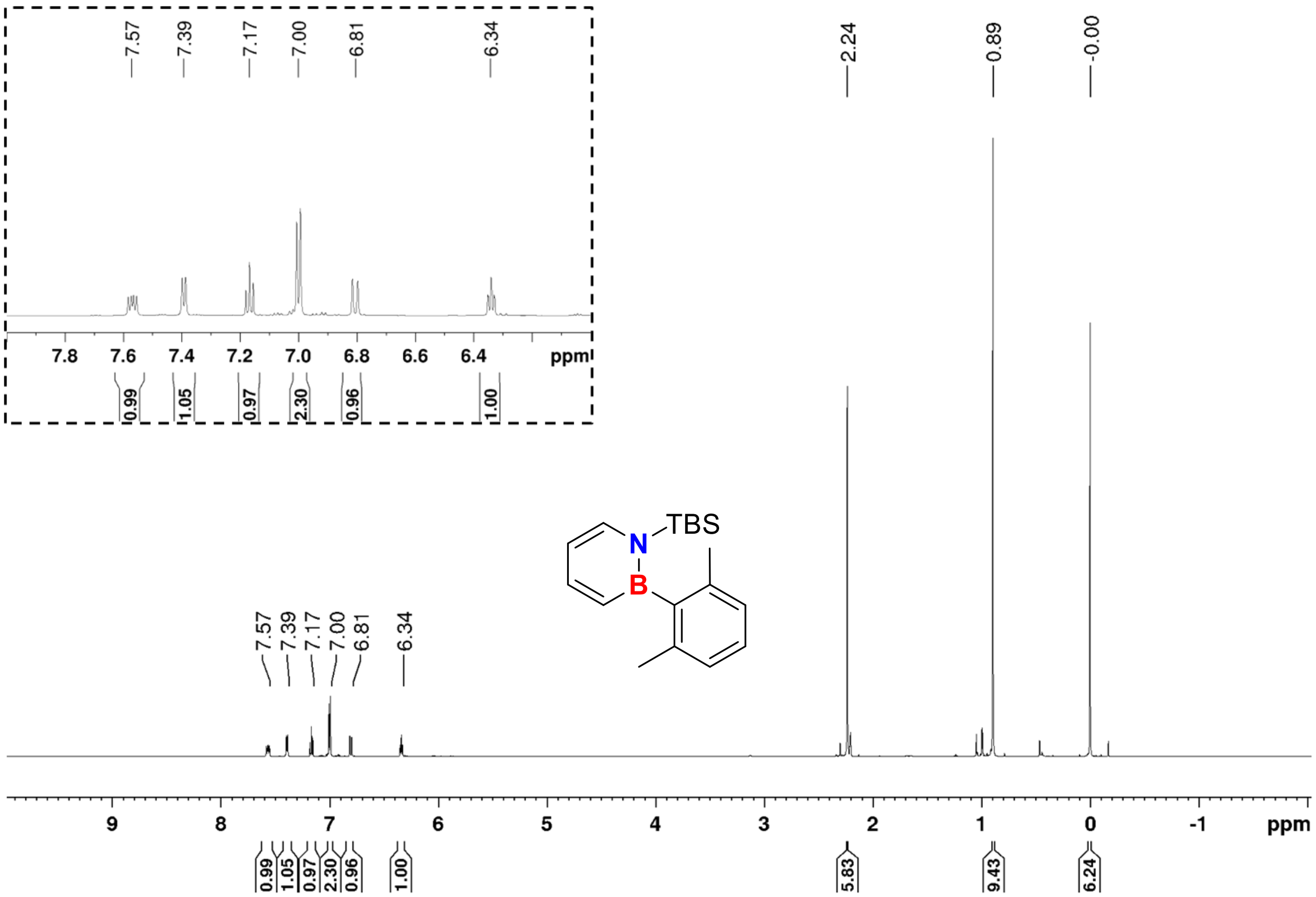

Figure S33. ^{1}H NMR spectrum of ^{BN}**B4** in C_6D_{12} measured on a 600 MHz spectrometer.

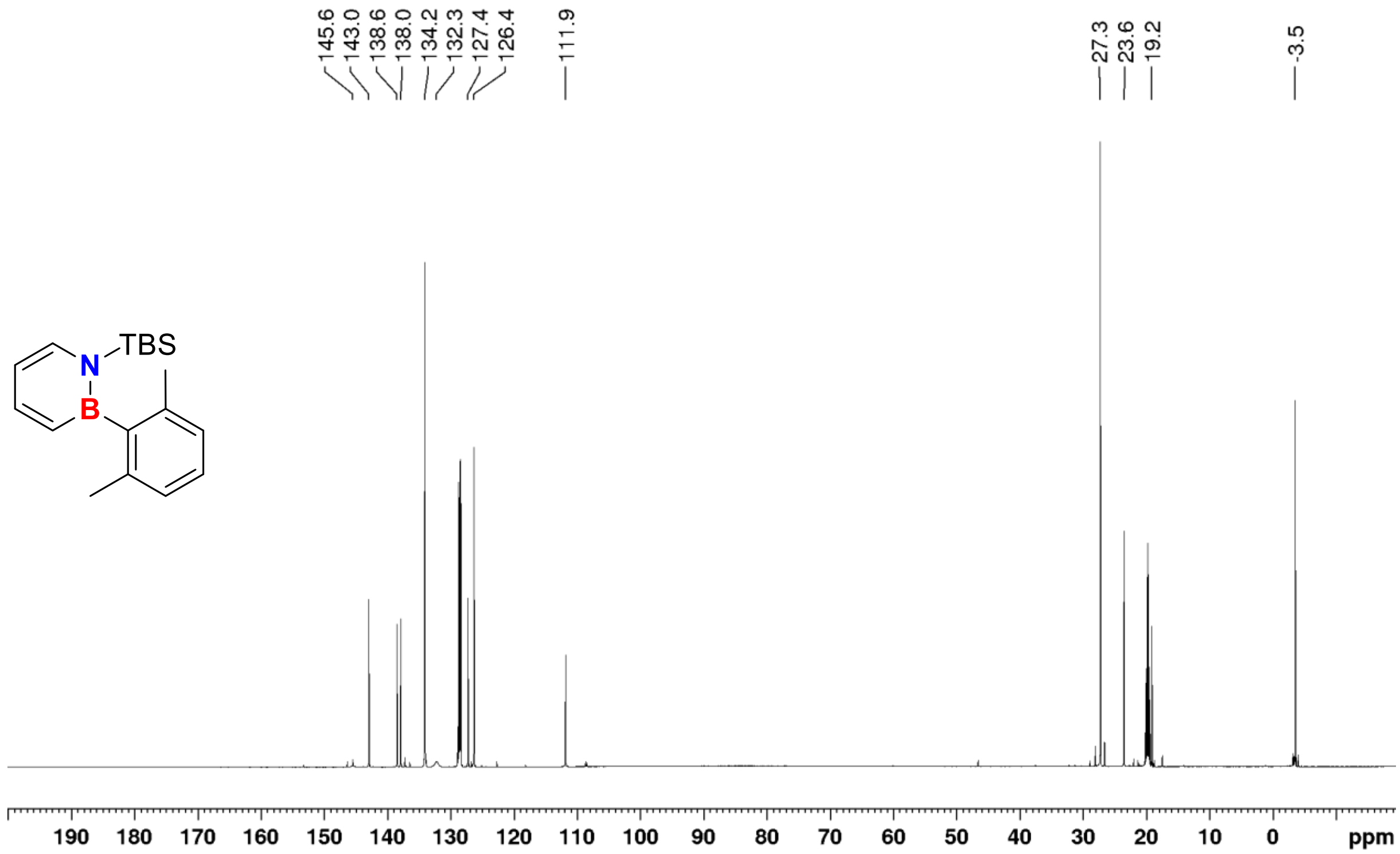

Figure S34. ^{13}C-{^{1}H} NMR spectrum of ***BN*B4** in C_6D_{12} measured on a 600 MHz spectrometer.

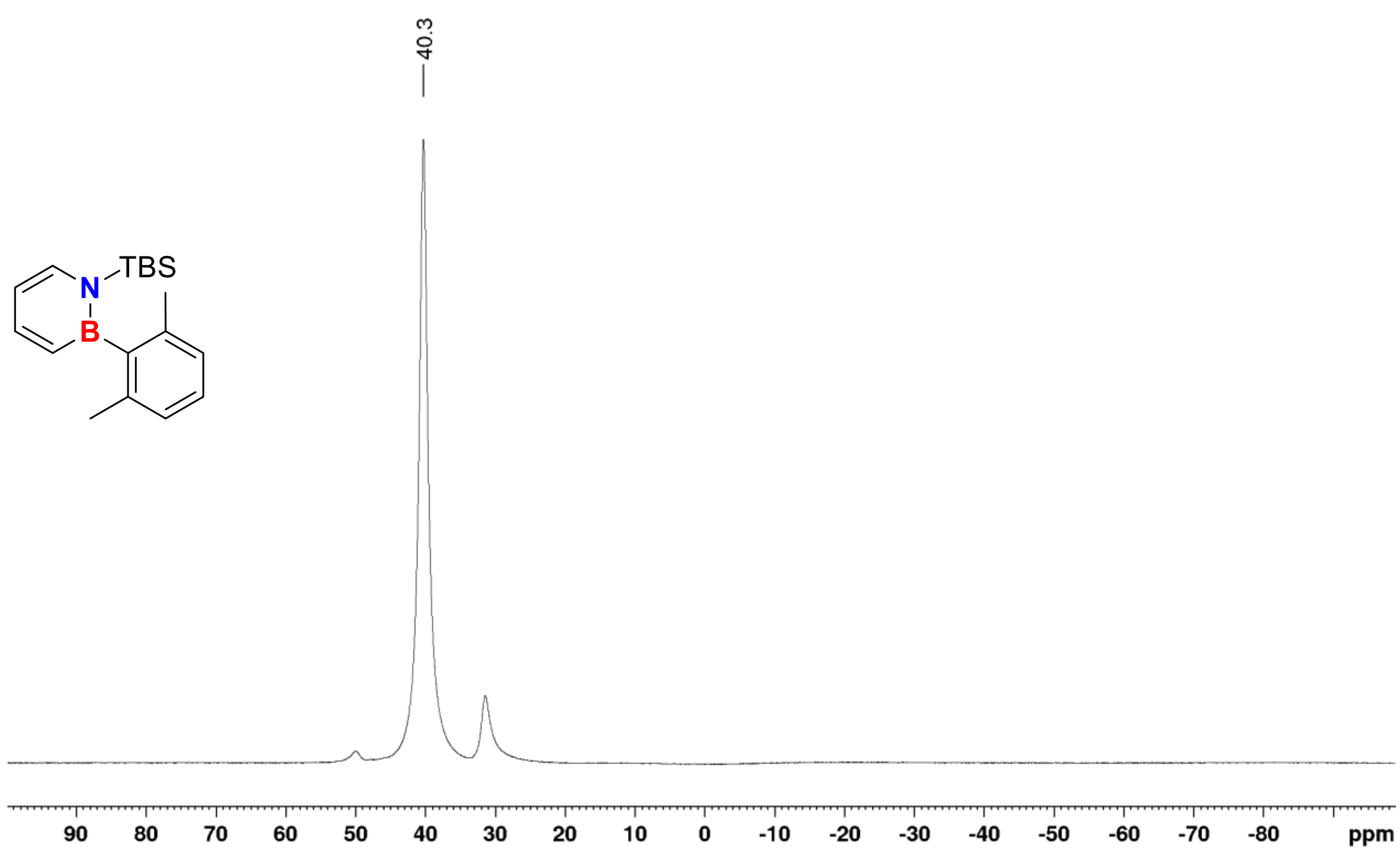

Figure S35. ^{11}B-{^{1}H} NMR spectrum of ***BN*B4** in C_6D_{12} measured on a 600 MHz spectrometer.

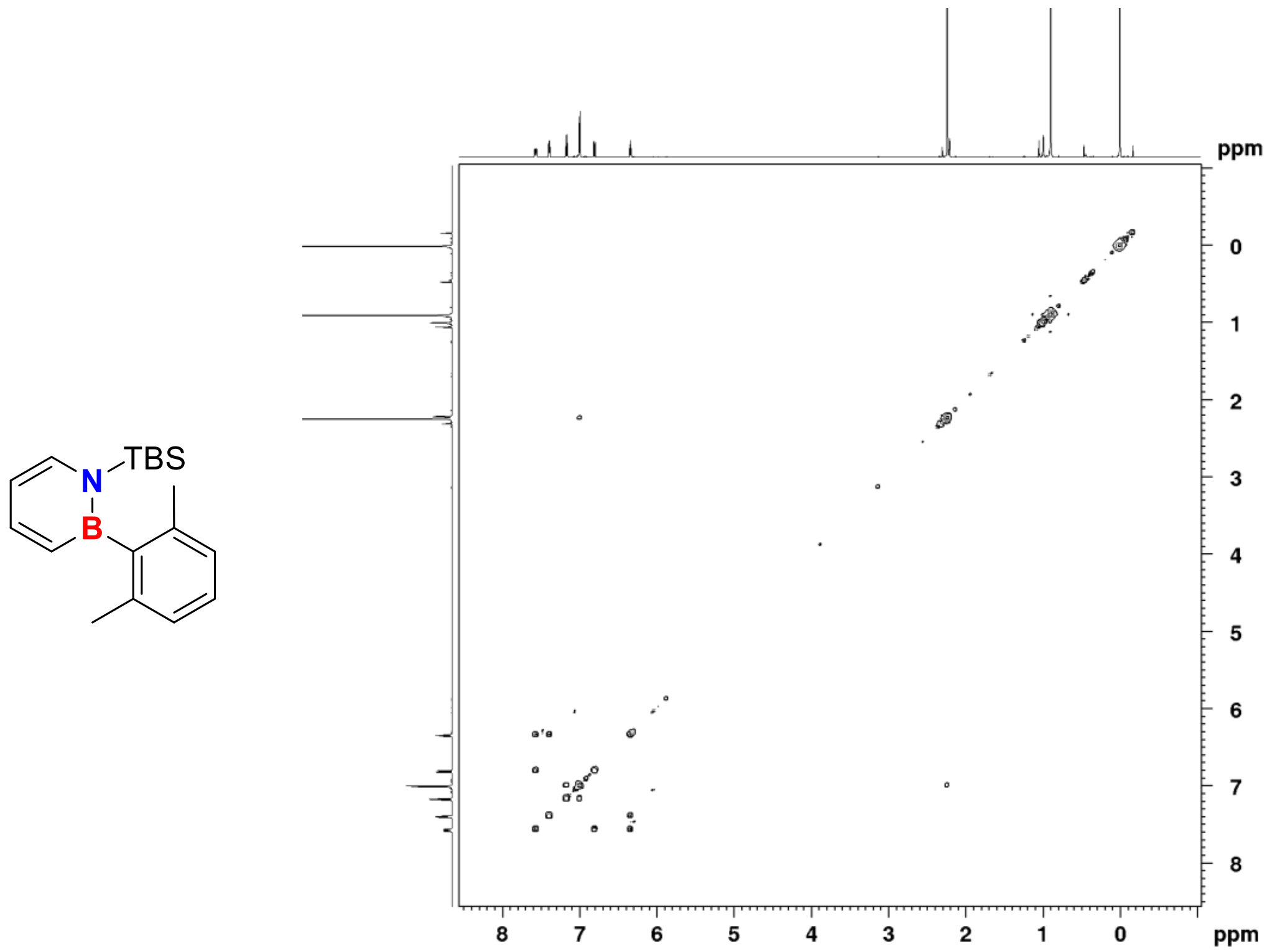

Figure S36. ^{1}H-^{1}H-COSY NMR spectrum of ***^{BN}*B4** in C_6D_{12} measured on a 600 MHz spectrometer.

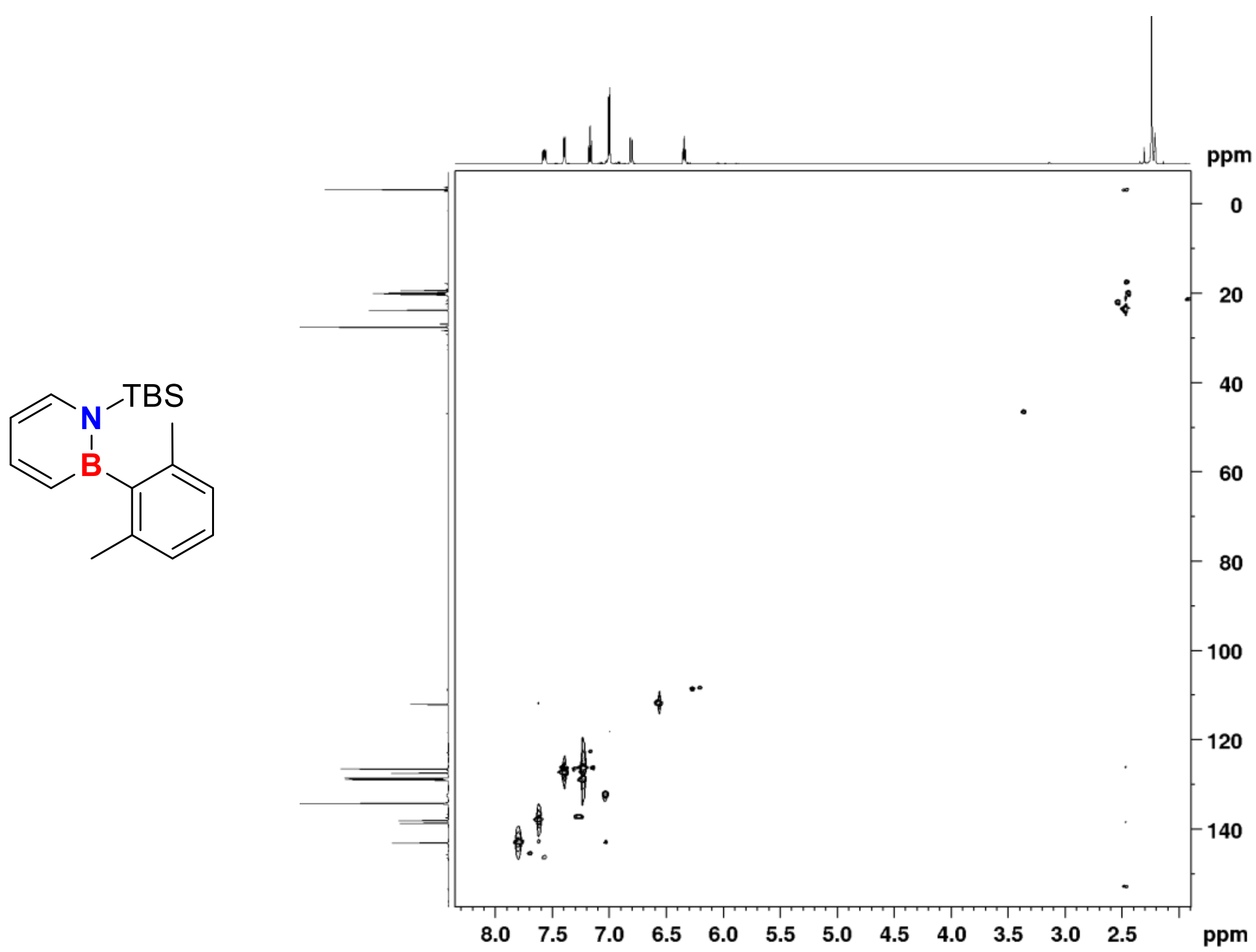

Figure S37. ^{1}H-^{13}C-HSQC NMR spectrum of ***^{BN}*B4** in C_6D_{12} measured on a 600 MHz spectrometer.

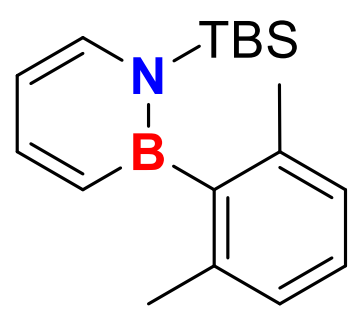

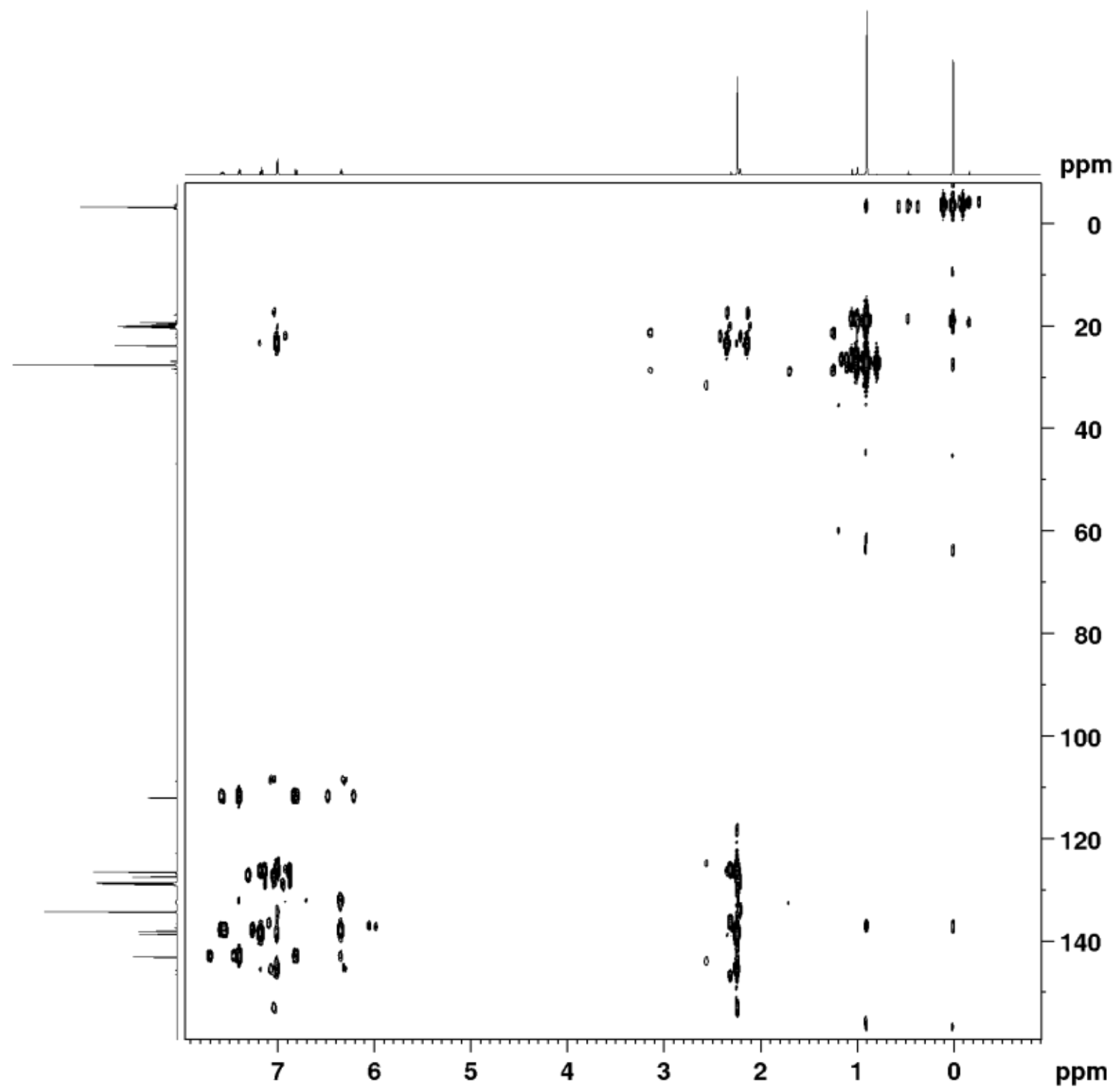

Figure S38. ^{1}H-^{13}C-HMBC NMR spectrum of ^{BN}**B4** in C_6D_{12} measured on a 600 MHz spectrometer.

3.2.4 1-(*tert*-Butyldimethylsilyl)-2-(pentamethylphenyl)-1,2-dihydro-1,2-azaborinine (^{BN}B5)

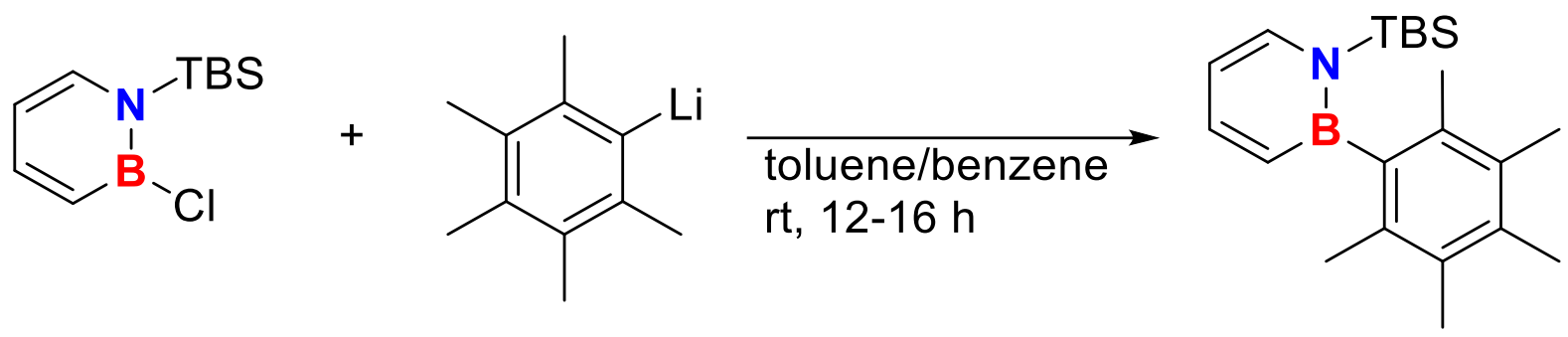

The following synthesis is based on procedures by Edel *et al.*[11] and Liu *et al*.[12]

A solution of compound ^{BN}**B2** (1.50 g, 6.6 mmol, 1 eq.) in toluene (20 mL) was slowly treated with pentamethylphenyl lithium (1.50 g, 9.9 mmol, 1.5 eq.). The reaction mixture was stirred at room temperature for 12 h and subsequently quenched with distilled water (10 mL). After extraction of the aqueous phase with *n*-hexane (3 × 10 mL), the combined organic phases were washed with saturated aqueous sodium chloride solution (2 × 40 mL) and dried over magnesium sulfate. After removal of the solvent, the crude product was obtained as a pale yellow, amorphous solid and purified by column chromatography (silica gel, *n*-hexane/CH_2Cl_2 gradient from 100:0 to 95:5). Compound ^{BN}**B5** was isolated as a colorless, crystalline solid in a yield of 1.7310 g (5.1 mmol, 76%).

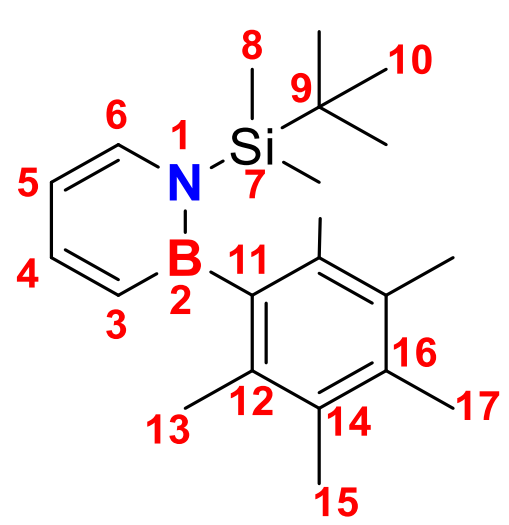

$C_{19}H_{30}BNSi$ (339.41 g/mol)

Mp = 67.3-69.1°C (uncorrected)

^{1}H NMR (400 MHz, **C_6D_6**): δ = 7.57 (ddd, $^3J_{HH}$ = 11.14 Hz, $^3J_{HH}$ = 6.22 Hz, $^4J_{HH}$ = 1.04 Hz, 1H, H-4), 7.36 (d, $^3J_{HH}$ = 6.48 Hz, 1H, H-6), 6.95 (dd, $^3J_{HH}$ = 11.14 Hz, $^3J_{HH}$ = 0.84 Hz, 1H, H-3), 6.30 (ps. t, 1H, H-5), 2.21 (s, 6H, H-13), 2.18 (s, 3H, H-17), 2.14 (s, 6H, H-15), 0.83 (s, 9H, H-10), -0,04 (s, 6H, H-8) ppm.

^{13}C-{^{1}H} NMR (101 MHz, **C_6D_6**): δ = 143.3 (C11), 142.5 (C4), 137.8 (C6), 134.2 (C12), 134.0 (C3), 132.8 (C16), 131.3 (C14), 111.6 (C5), 27.4 (C10), 22.3 (C13), 19.2 (C9), 16.4 (C17), 16.0 (C15), -3.2 (C8) ppm.

^{11}B-{^{1}H} NMR (128 MHz, **C_6D_6**): δ = 41.0 ppm.

HRMS (APCI) m/z: [M + H]+ Calcd for C19H30BNSi 340.2631; Found 340.2635.

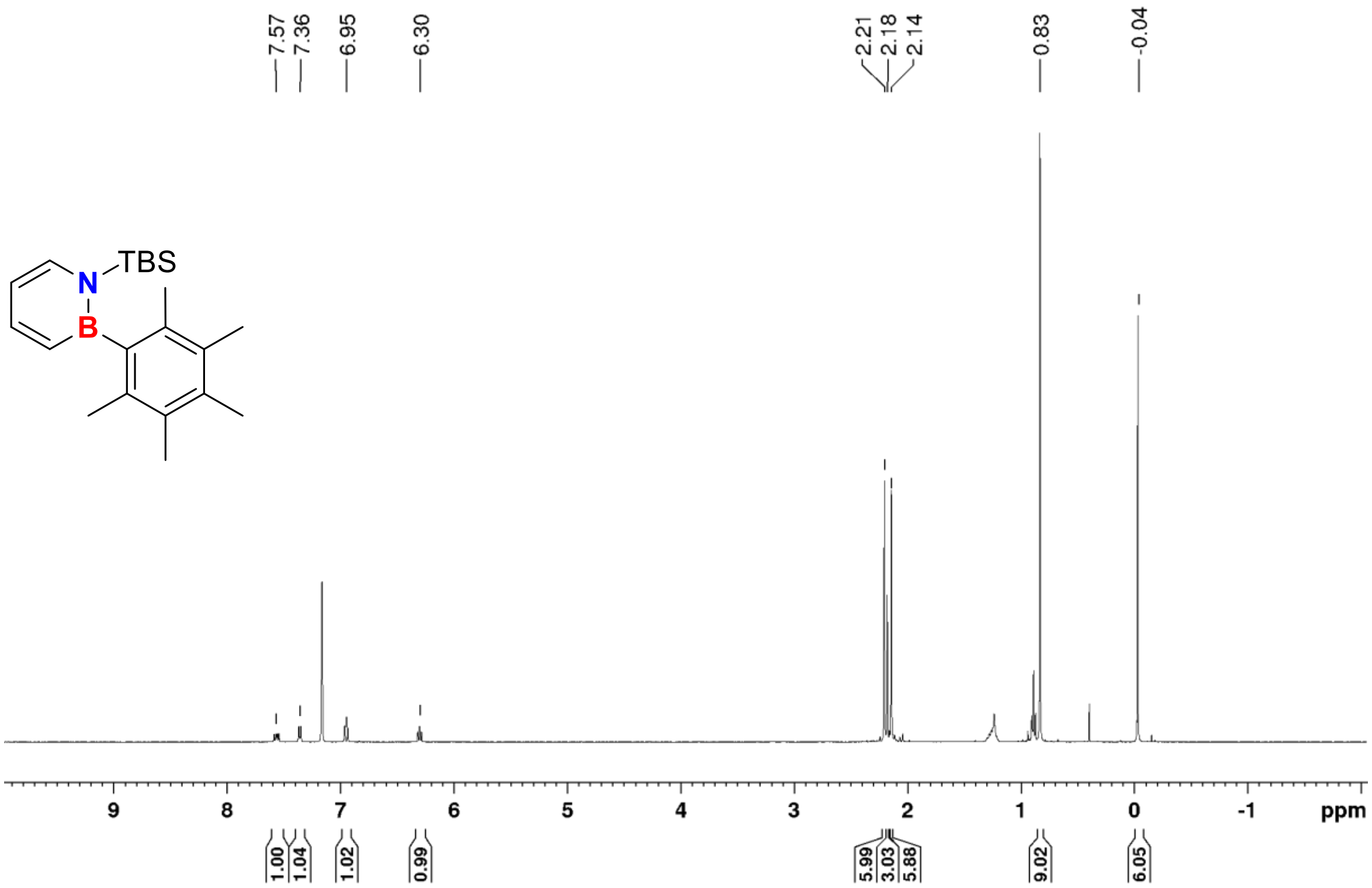

Figure S39. ^{1}H NMR spectrum of ^{BN}**B5** in C_6D_{12} measured on a 600 MHz spectrometer.

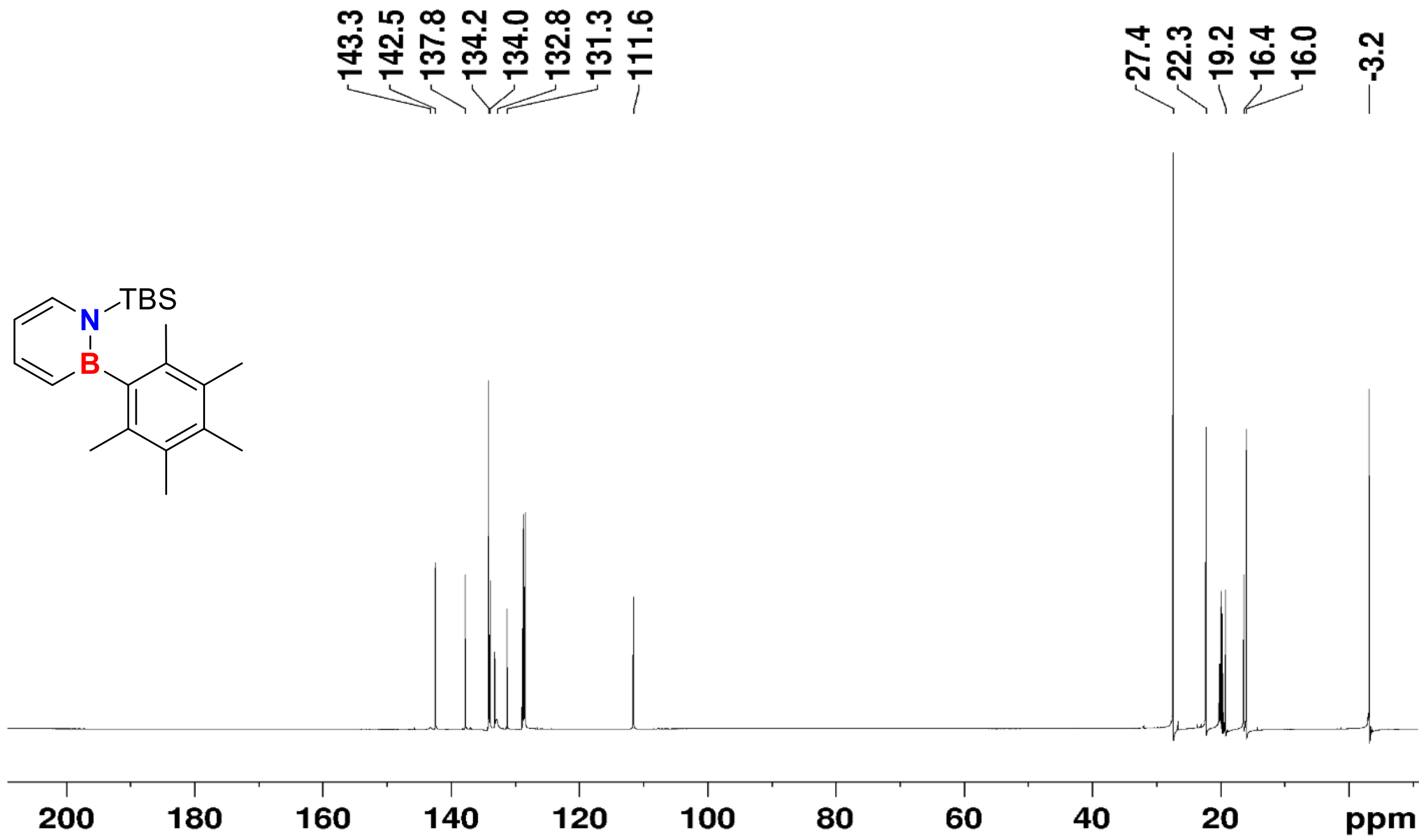

Figure S40. $^{13}C-\{^1H\}$ NMR spectrum of ***BN*B5** in C_6D_6 measured on a 600 MHz spectrometer.

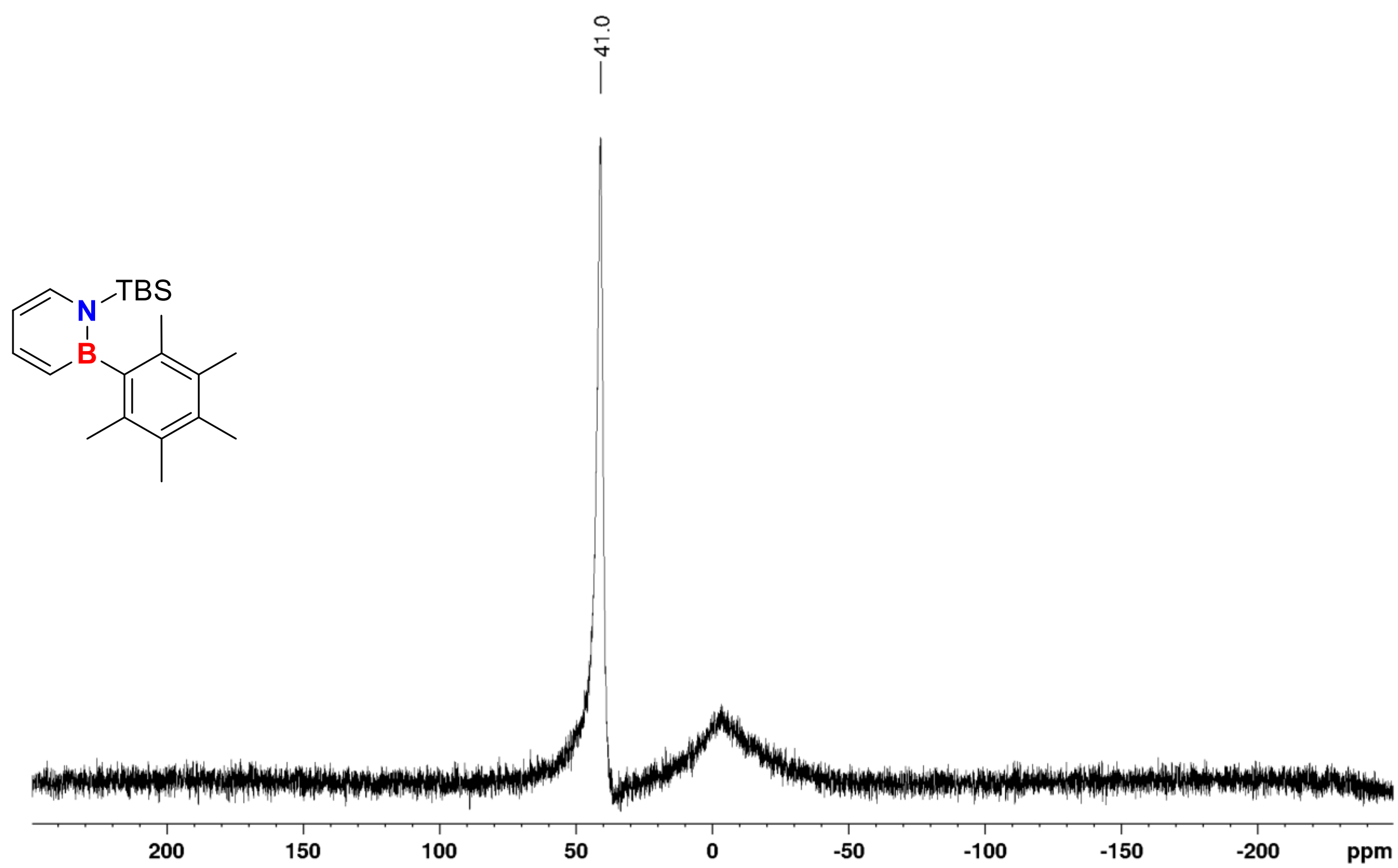

Figure S41. $^{11}B-\{^1H\}$ NMR spectrum of ***BN*B5** in C_6D_{12} measured on a 600 MHz spectrometer.

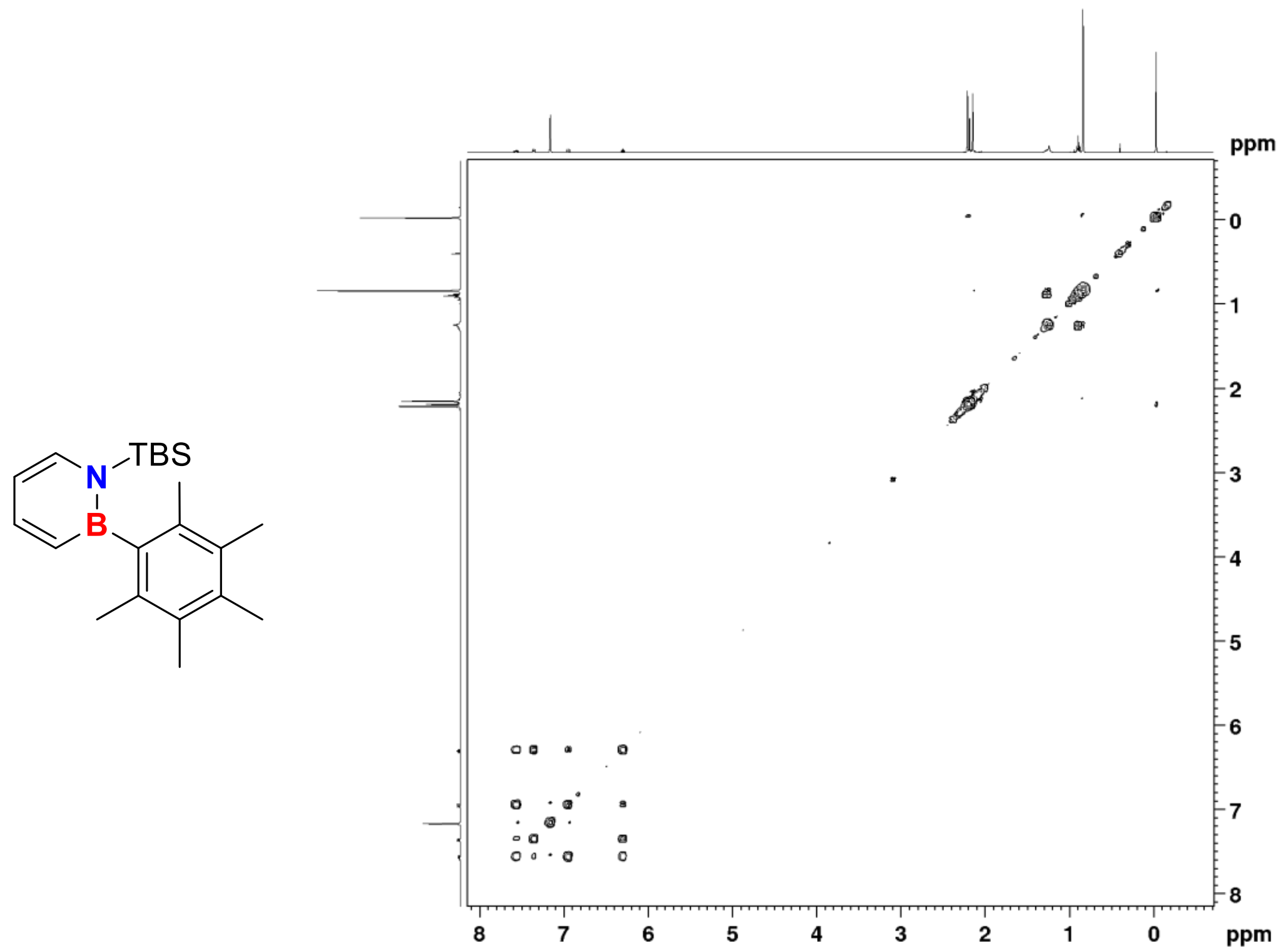

Figure S42. ^{1}H-^{1}H-COSY NMR spectrum of ^{BN}**B5** in C_6D_{12} measured on a 600 MHz spectrometer.

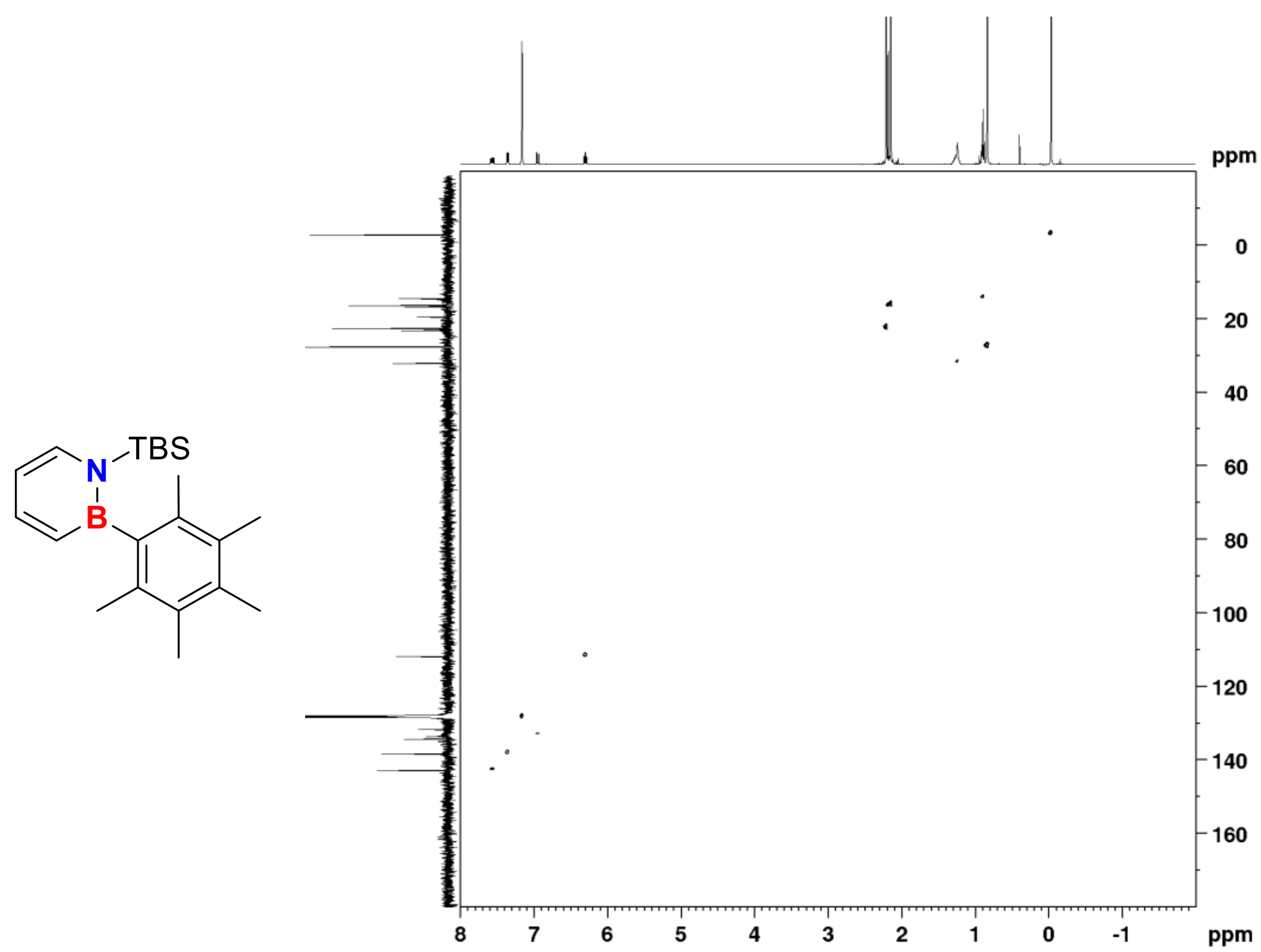

Figure S43. ^{1}H-^{13}C-HSQC NMR spectrum of ^{BN}**B5** in C_6D_{12} measured on a 600 MHz spectrometer.

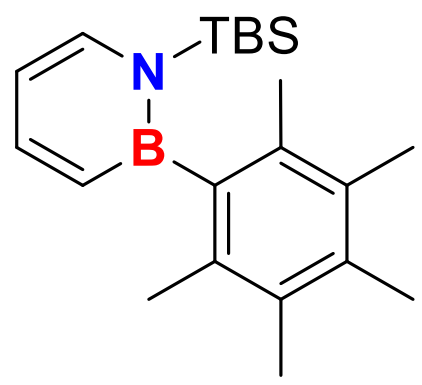

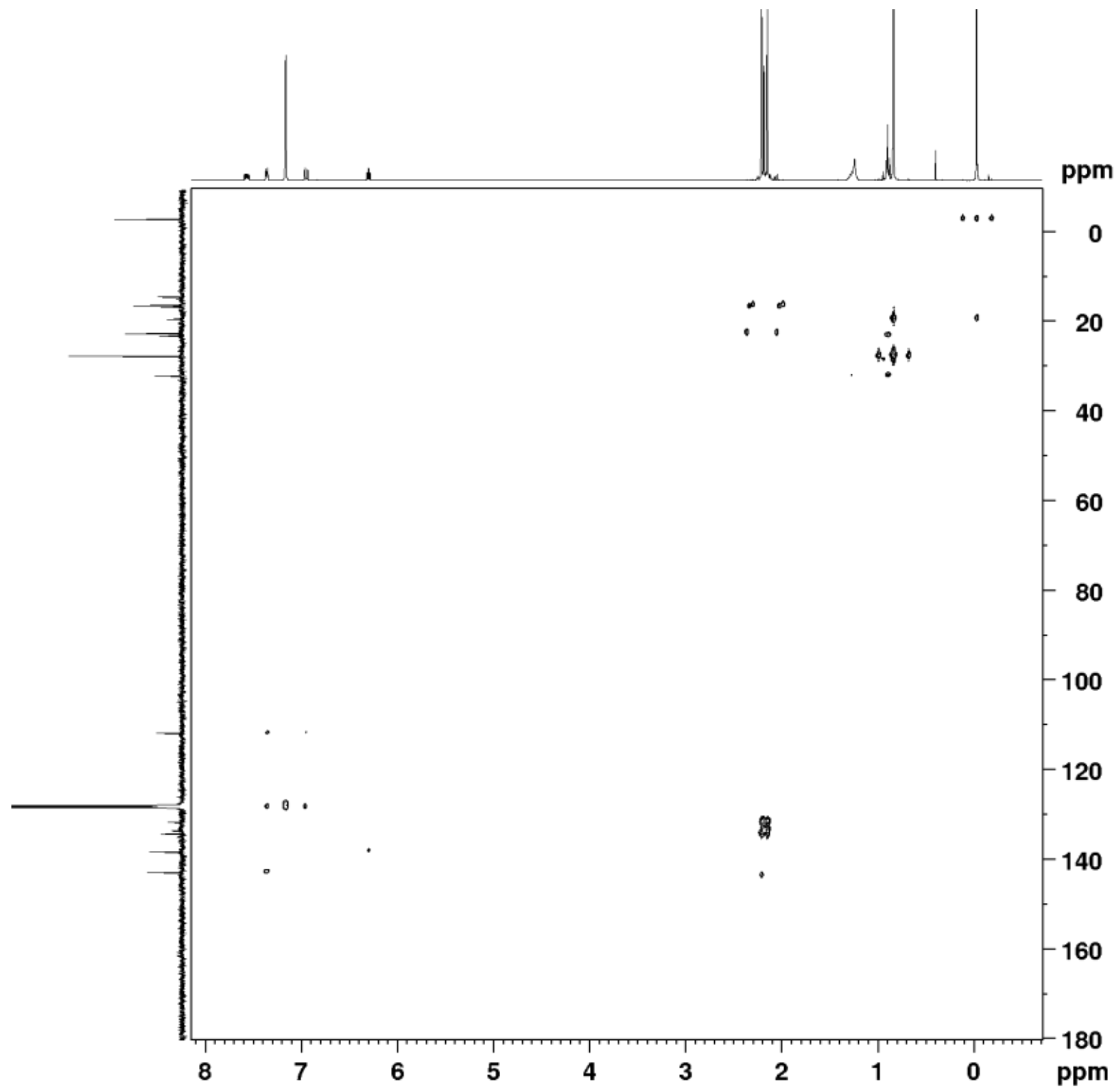

Figure S44. ^{1}H-^{13}C-HMBC NMR spectrum of ^{BN}**B5** in C_6D_{12} measured on a 600 MHz spectrometer.

3.2.7 1-(*tert*-Butyldimethylsilyl)-2-(4-methoxy-2,6-dimethylphenyl)-1,2-dihydro-1,2-azaborinine (^{BN}B6)

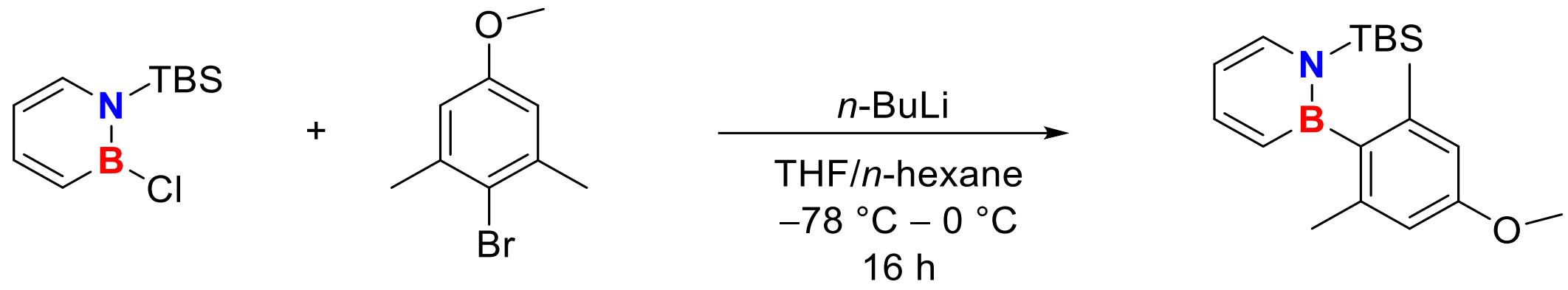

The synthesis is based on a procedure by Edel *et al.*[11]

2-Bromo-5-methoxy-1,3-dimethylbenzene (25 mg, 0.12 mmol, 1.05 eq.) was dissolved in dry THF (2 mL) and cooled to -78 °C. Under stirring, *n*-butyllithium (1.6 M in *n*-hexane, 72 µL, 0.12 mmol, 1.05 eq.) was added dropwise. The resulting suspension was stirred for 1.5 h at -78 °C. A solution of ***BN*B2** (25 mg, 0.11 mmol, 1.0 eq.) in *n*-hexane (0.5 mL) was added carefully and it was further stirred at -78 °C for 2 h. The reaction mixture was allowed to warm to room temperature and stirred overnight. The solvent was removed under reduced pressure, the residue suspended in *n*-hexane (3 mL) and the mixture filtered through a syringe filter. The crude product was purified by column chromatography (silica gel, *n*-hexane/EtOAc gradient from 100:0 to 98:2), size exclusion chromatography (*n*-hexane/dichloromethane 80:20) and ***BN*B6** was obtained as a colorless oil in a yield of 12 mg (37 µmol, 34%).

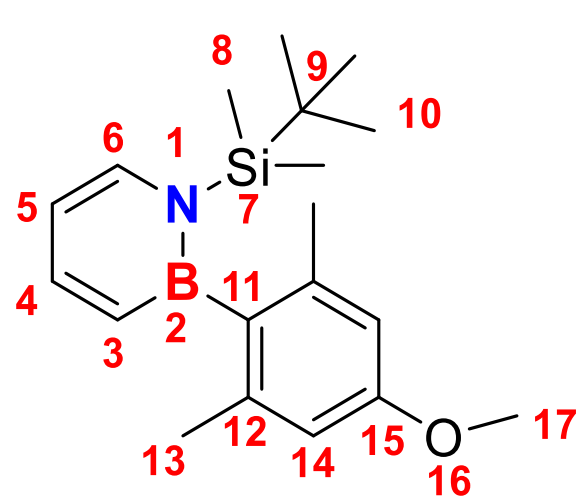

^{1}H NMR (400 MHz, $CDCl_3$) δ = 7.56 (ddd, *J* = 11.0, 6.3, 1.3 Hz, 1H, *H*-4), 7.44 (d, *J* = 6.9, 1H, *H*-6), 6.63 (d, *J* = 11.0 Hz, 1H, *H*-3), 6.54 (s, 3H, *H*-14), 6.38 (ps. td, *J* = 6.6, 1.6 Hz, 1H, *H*-5), 3.80 (s, 3H, *H*-17), 2.10 (s, 6H, *H*-13), 0.91 (s, 9H, *H*-10), 0.01 (s, 6H, *H*-8) ppm.

^{13}C-{^{1}H} NMR (101 MHz, $CDCl_3$) δ = 159.1 (C15), 142.7 (C4), 140.5 (C11), 138.5 (C6), 111.8 (C14), 111.6 (C5), 55.0 (C17), 27.6 (C10), 23.9 (C13), 19.4 (C9), -3.1 (C8) ppm.

C3 and C12 not detectable.

^{11}B-{^{1}H} NMR (128 MHz, $CDCl_3$) δ = 40.2 ppm.

HRMS (ESI) m/z: [M + H]+ Calcd for C19H30BNOSi 328.2262; Found 328.2262.

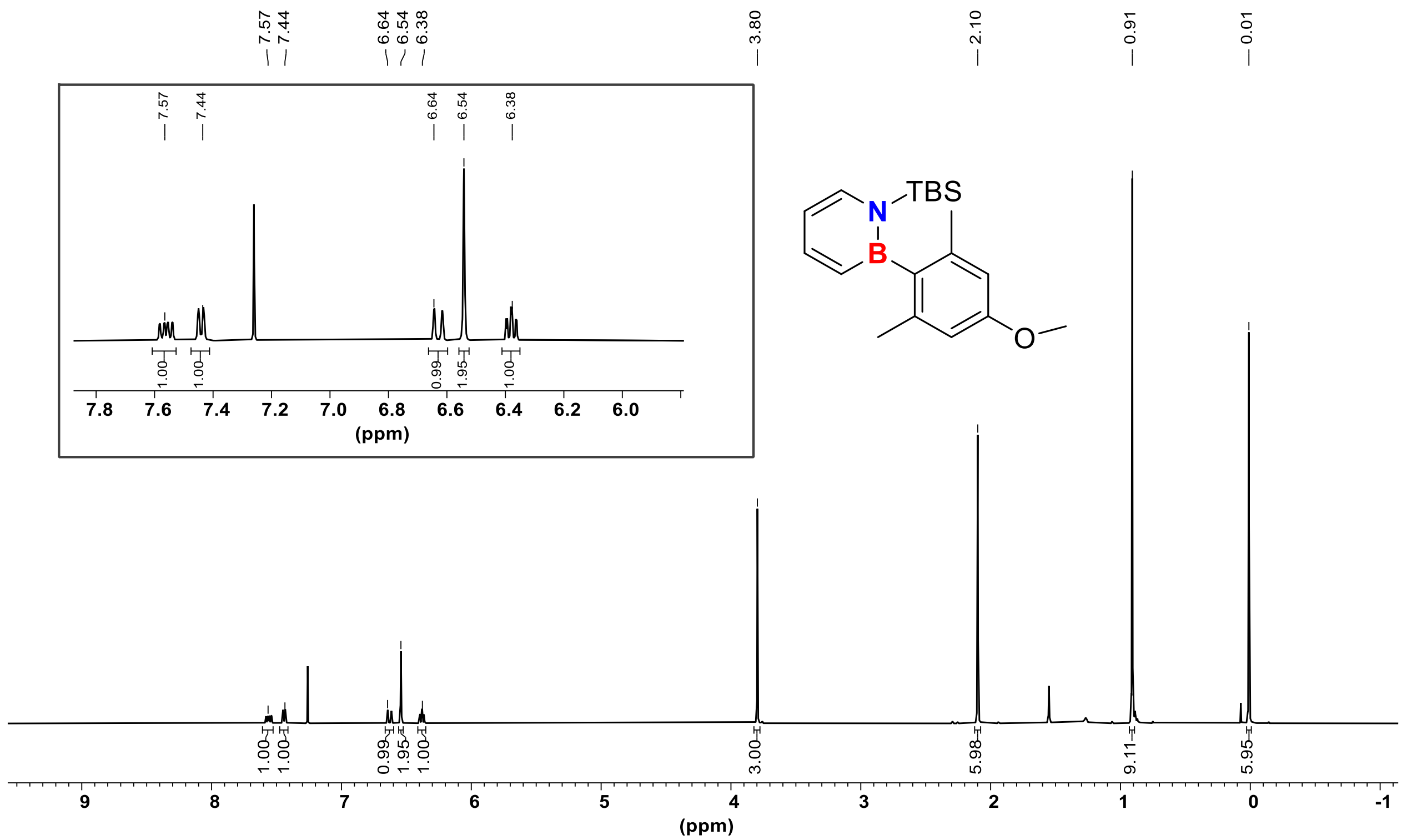

Figure S45: ^{1}H NMR spectrum of ***BN*B6** in $CDCl_3$ measured on a 400 MHz spectrometer.

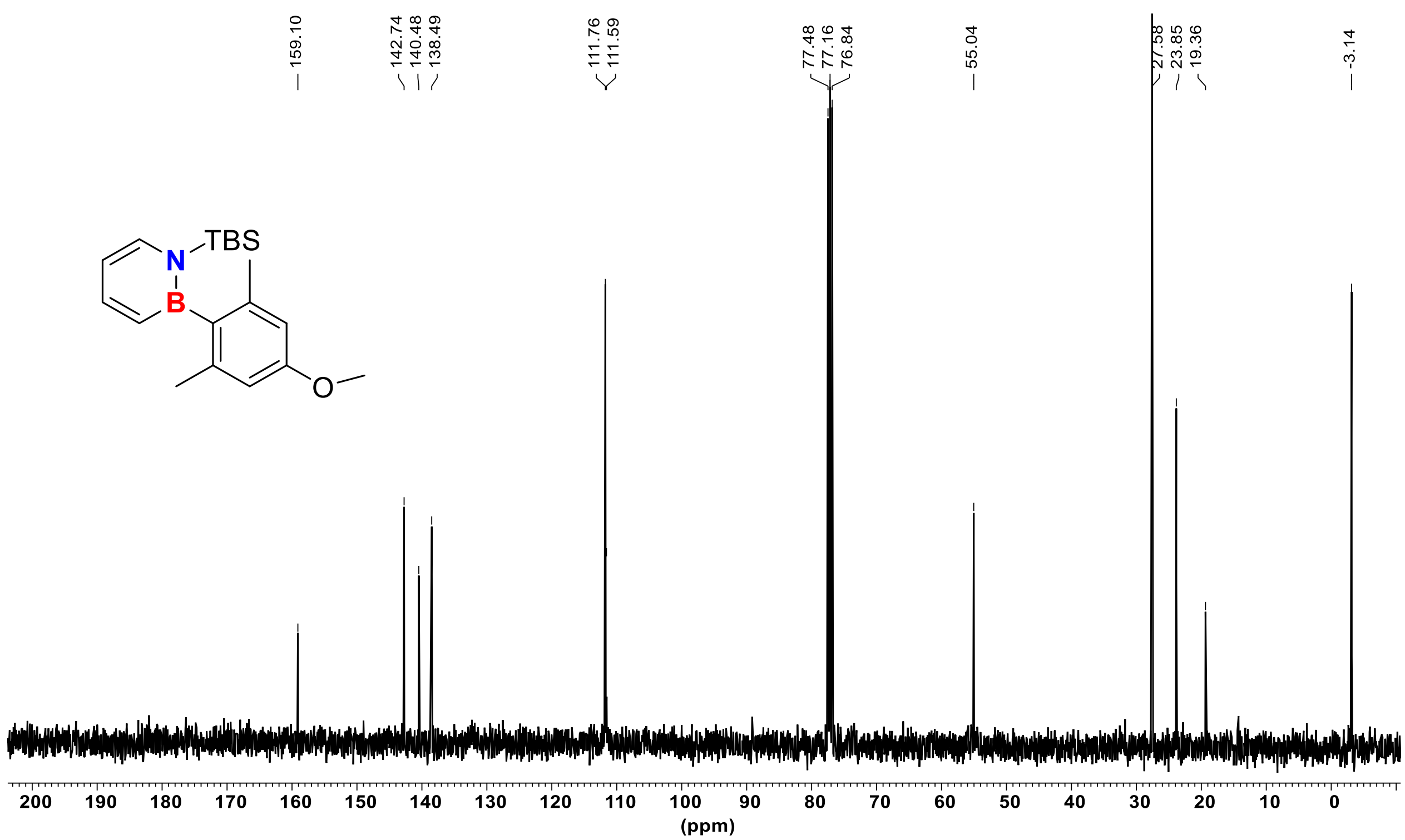

Figure S46: ^{13}C-{^{1}H} NMR spectrum of ***BN*B6** in $CDCl_3$ measured on a 400 MHz spectrometer.

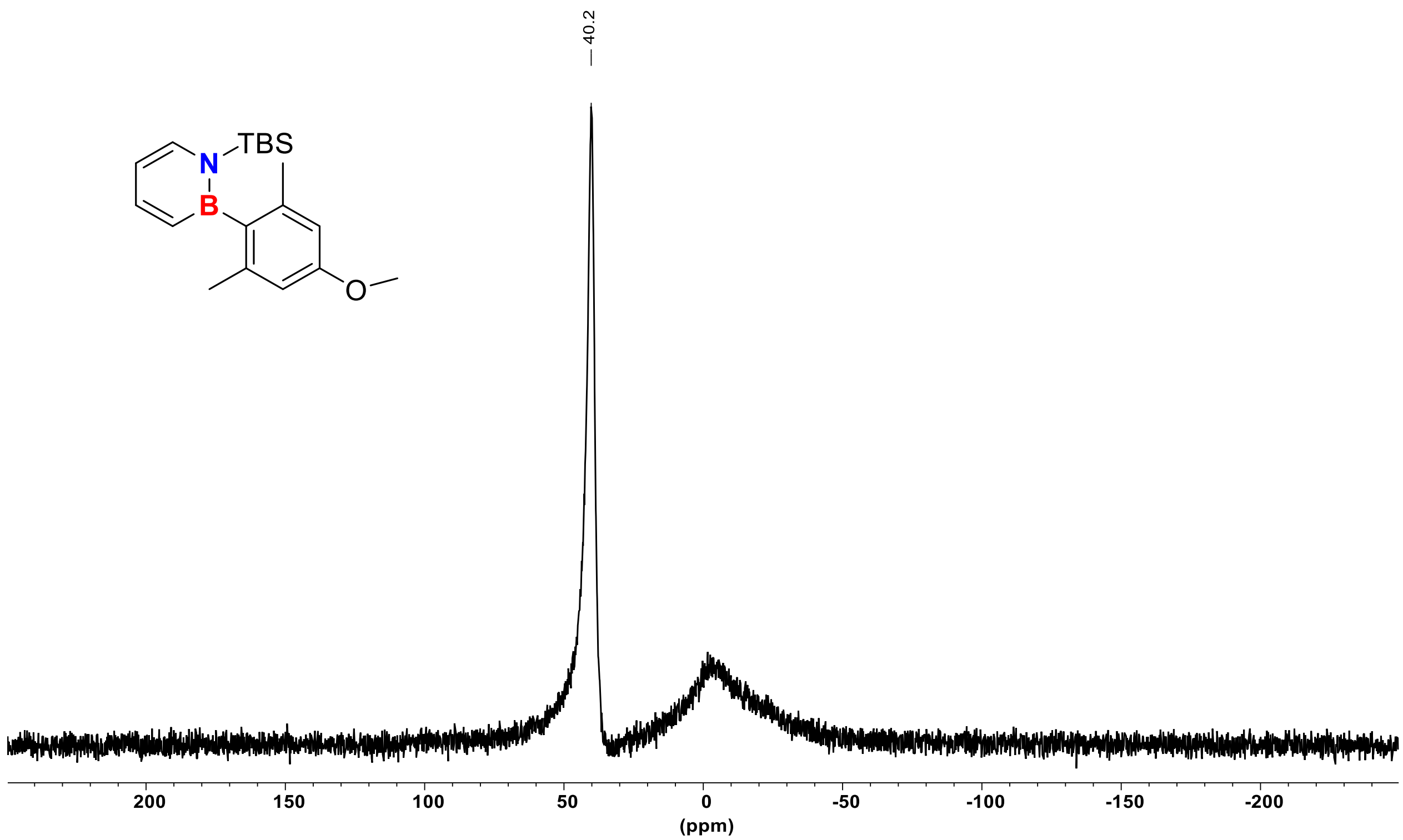

Figure S47: ^{11}B-{^{1}H} NMR spectrum of ***BN*B6** in $CDCl_3$ measured on a 400 MHz spectrometer.

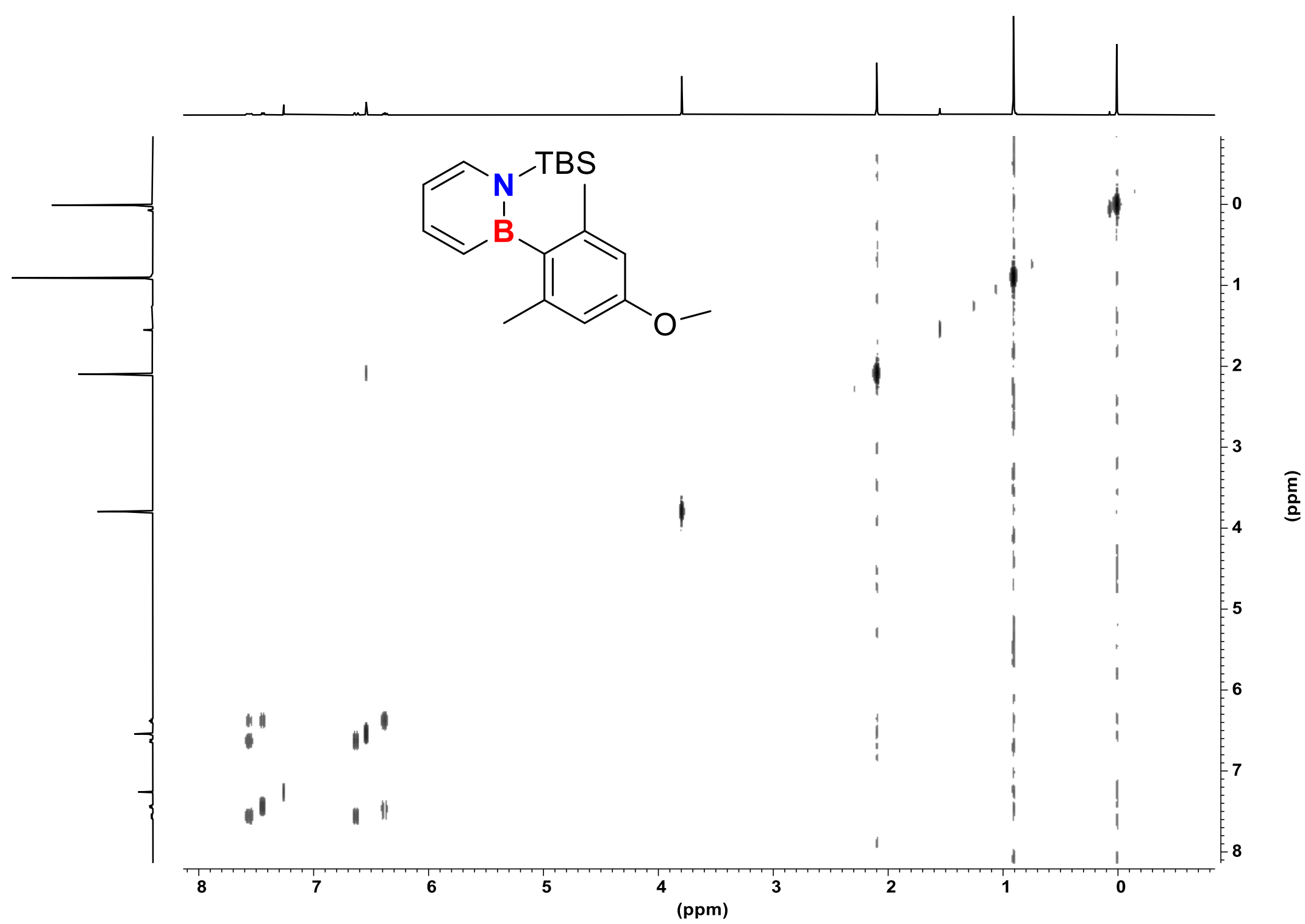

Figure S48: ^{1}H-^{1}H-COSY NMR spectrum of ***BN*B6** in $CDCl_3$ measured on a 400 MHz spectrometer.

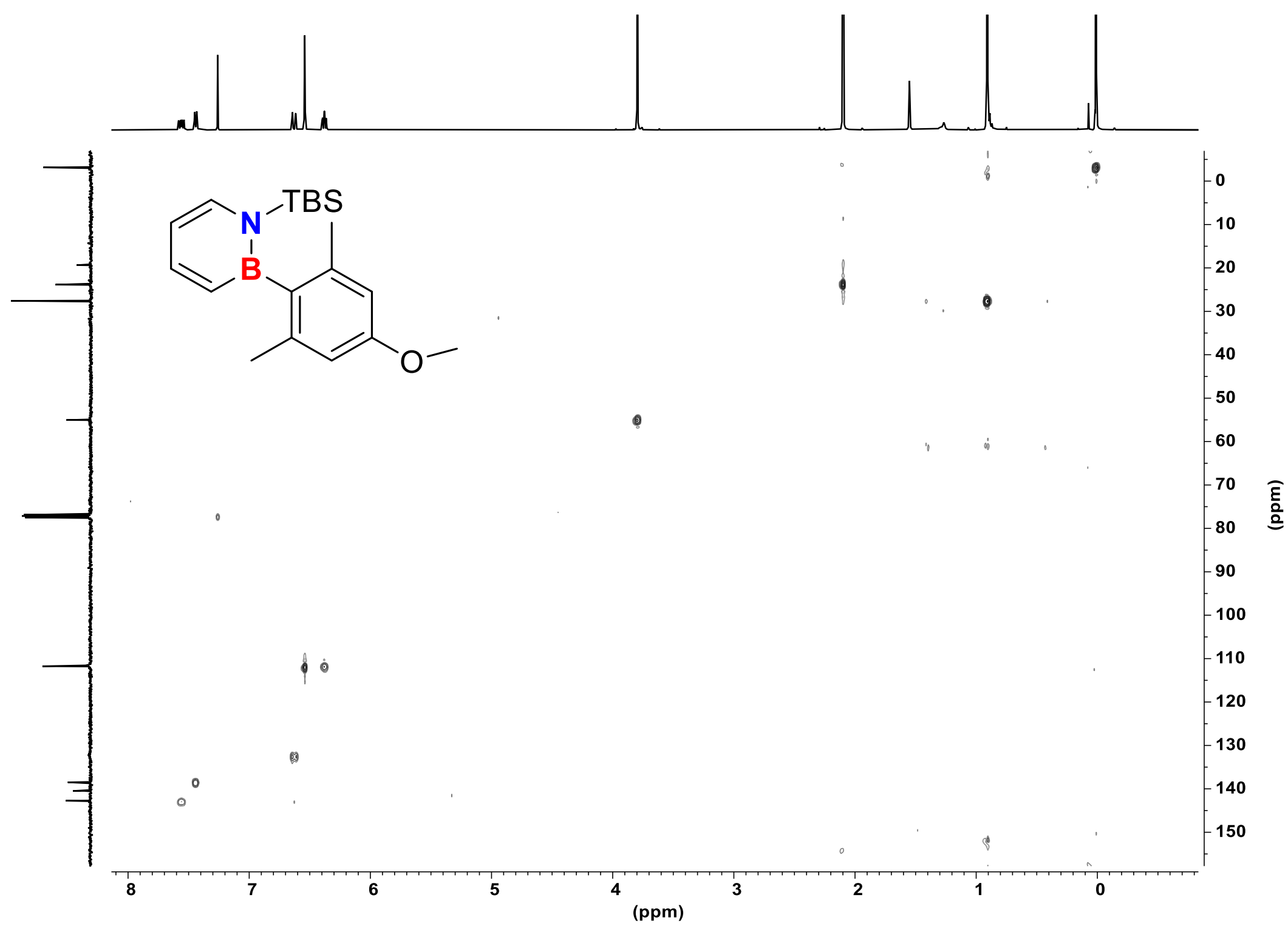

Figure S49: ^{1}H-^{13}C-HSQC NMR spectrum of ***BN*B6** in $CDCl_3$ measured on a 400 MHz spectrometer.

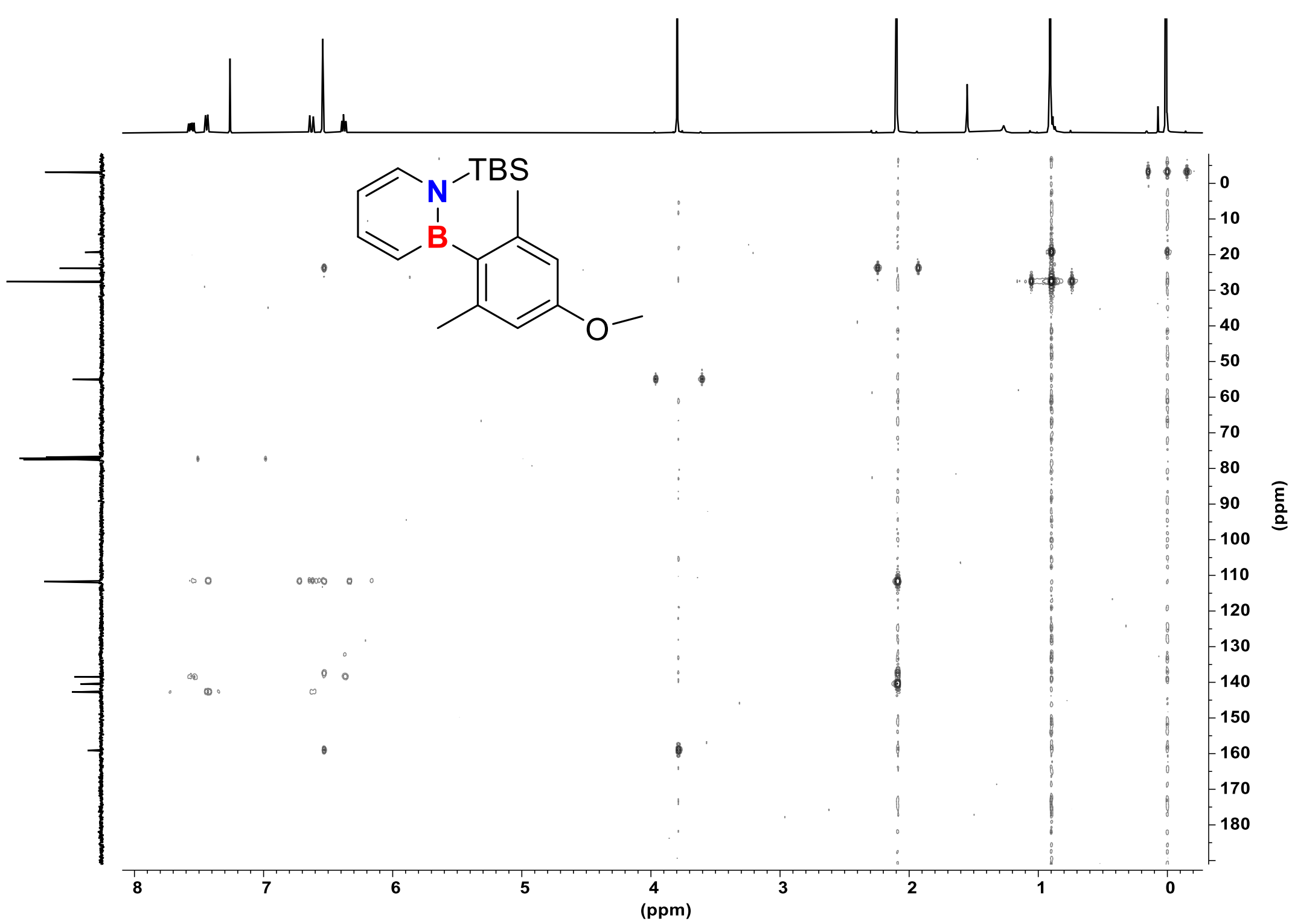

Figure S50: ^{1}H-^{13}C-HMBC NMR spectrum of ***BN*B6** in $CDCl_3$ measured on a 400 MHz spectrometer.

3.2.8 1-(*tert*-Butyldimethylsilyl)-2-(4-cyano-2,6-dimethylphenyl)-1,2-dihydro-1,2-azaborinine (*BN*B7)

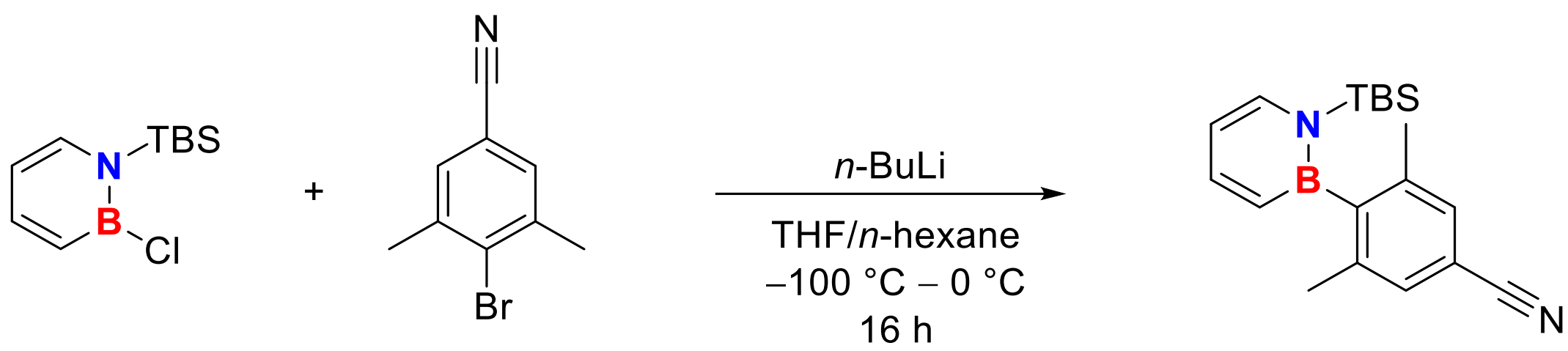

The synthesis is based on a procedure by Edel *et al.*[11]

2-Bromo-5-cyano-1,3-dimethylbenzene (49 mg, 0.23 mmol, 1.05 eq.) was dissolved in dry THF (3 mL) and cooled to -100 °C. Under stirring, *n*-butyllithium (1.6 M in *n*-hexane, 0.14 mL, 0.23 mmol, 1.05 eq.) was added dropwise. The resulting suspension was stirred for 3 h at -100 °C. A solution of ***BN*B2** (25 mg, 0.11 mmol, 1.0 eq.) in *n*-hexane (1 mL) was added carefully and it was further stirred at -100 °C for 2 h. The reaction mixture was allowed to warm to room temperature and stirred overnight. The solvent was removed under reduced pressure, the residue suspended in *n*-hexane (5 mL) and the mixture filtered through a syringe filter. The crude product was purified by column chromatography (silica gel, *n*-hexane/EtOAc gradient from 100:0 to 98:2), size exclusion chromatography (*n*-hexane/dichloromethane 80:20) and ***BN*B7** was obtained as a colorless oil in a yield of 24 mg (75 µmol, 33%).

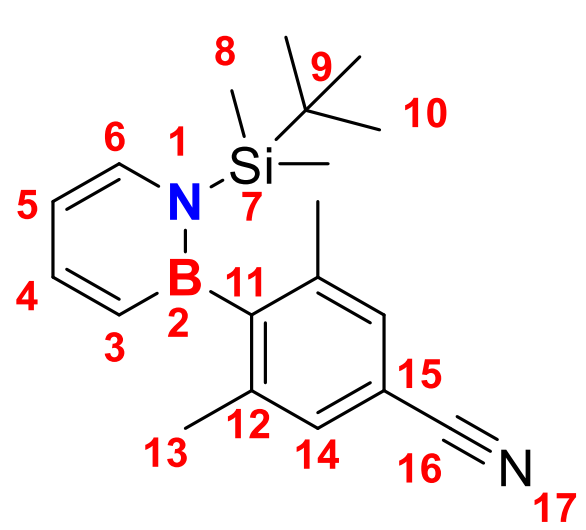

^{1}H NMR (400 MHz, **C_6D_{12}**) δ = 7.58 (ddd, *J* = 11.0, 6.3, 1.2 Hz, 1H, *H*-4), 7.47 (d, *J* = 6.8, 1H, *H*-6), 7.15 (s, 2H, *H*-14), 6.58 (d, *J* = 11.0, 1H, *H*-3), 6.41 (ps. td, *J* = 6.6, 1.5 Hz, 1H, *H*-5), 2.15 (s , 6H, *H*-13), 0.97 (s, 9H, *H*-10), 0.04 (s, 6H, *H*-8) ppm.

^{13}C-{^{1}H} NMR (101 MHz, **C_6D_{12}**) δ = 144.0 (*C*4), 140.3 (*C*12), 138.5 (*C*6), 129.7 (*C*14), 118.4 (*C*11), 113.1 (*C*5), 27.8 (*C*10), 23.5 (*C*13), 19.8 (*C*9), 2.97 (*C*8) ppm.

^{11}B-{^{1}H} NMR (128 MHz, **C_6D_{12}**) δ = 39.6 ppm.

HRMS (ESI) m/z: [M + H]+ Calcd for C19H27BN2Si 323.2109; Found 323.2110

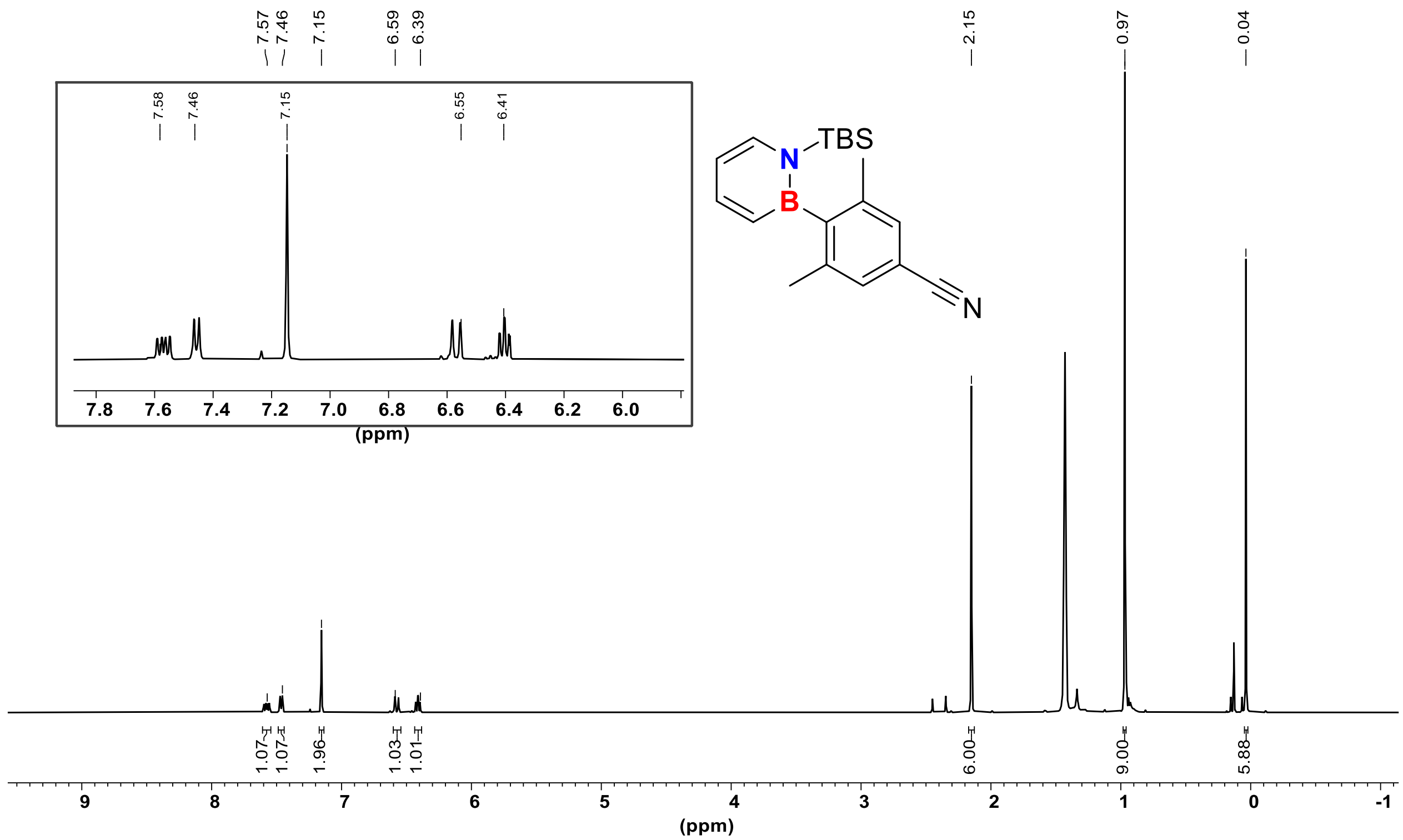

Figure S51: ^{1}H NMR spectrum of ***BN*B7** in C_6D_{12} measured on a 400 MHz spectrometer.

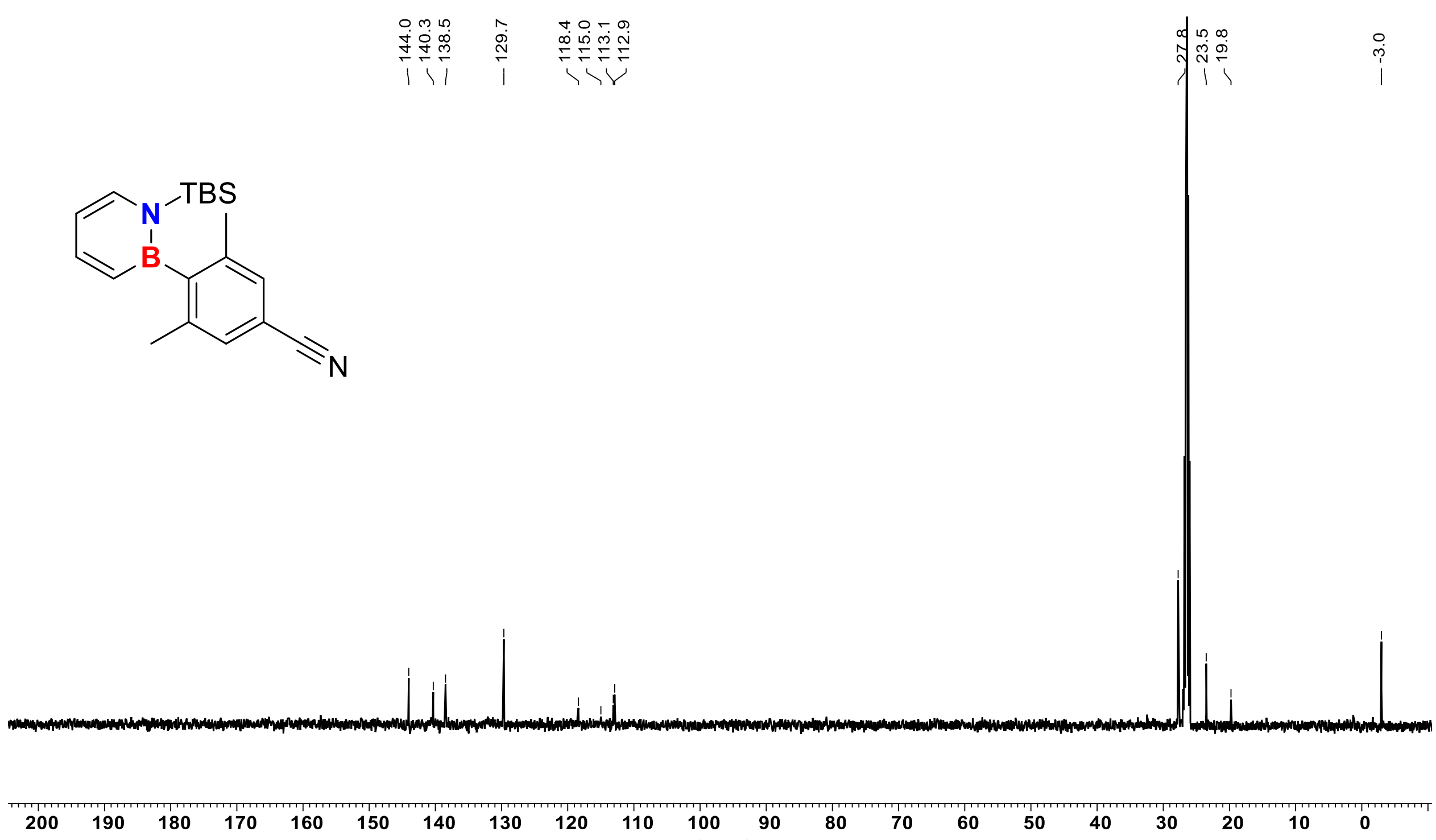

Figure S52: ^{13}C-{^{1}H} NMR spectrum of ***BN*B7** in C_6D_{12} measured on a 400 MHz spectrometer.

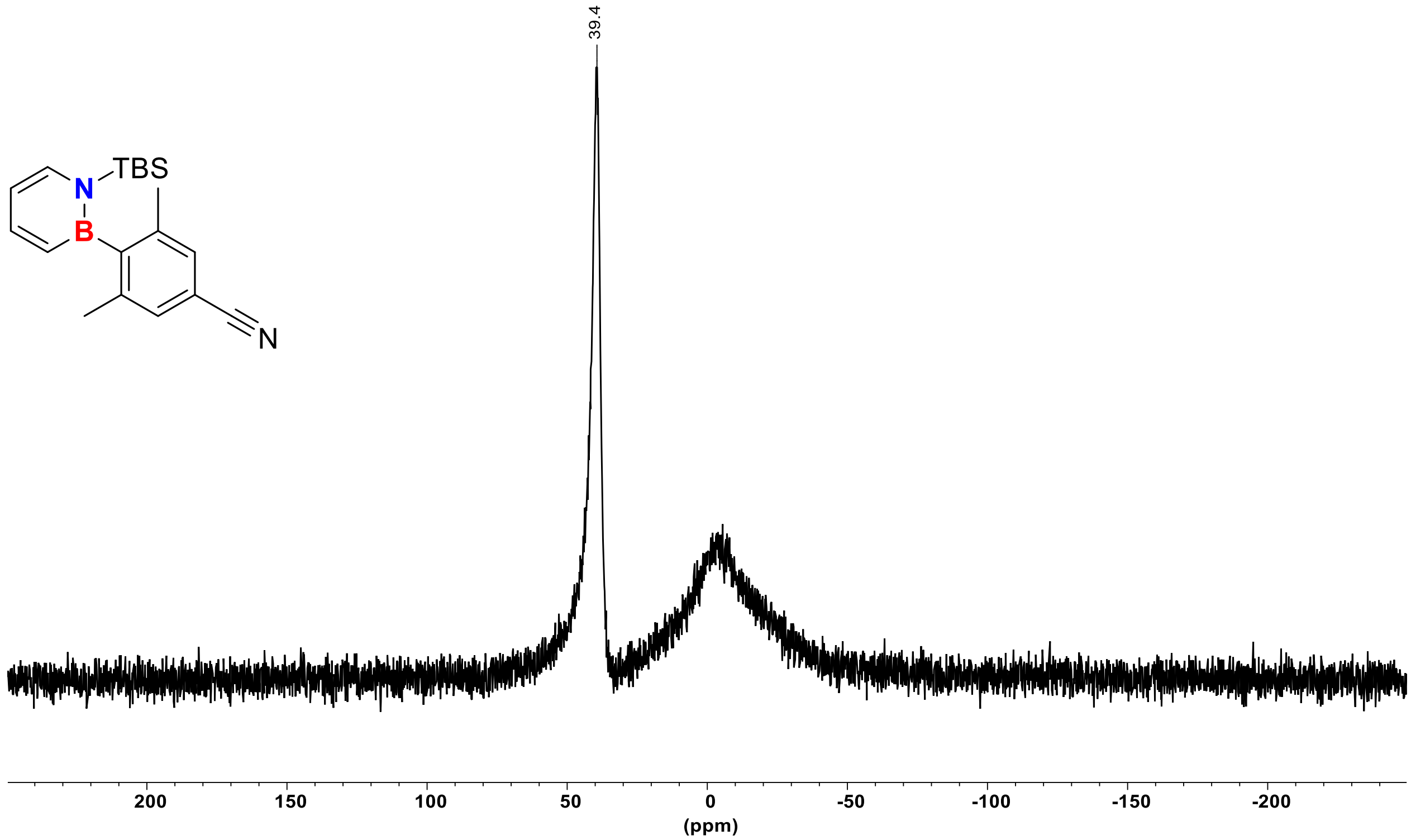

Figure S53: ^{11}B-{^{1}H} NMR spectrum of ^{BN}**B7** in C_6D_{12} measured on a 400 MHz spectrometer.

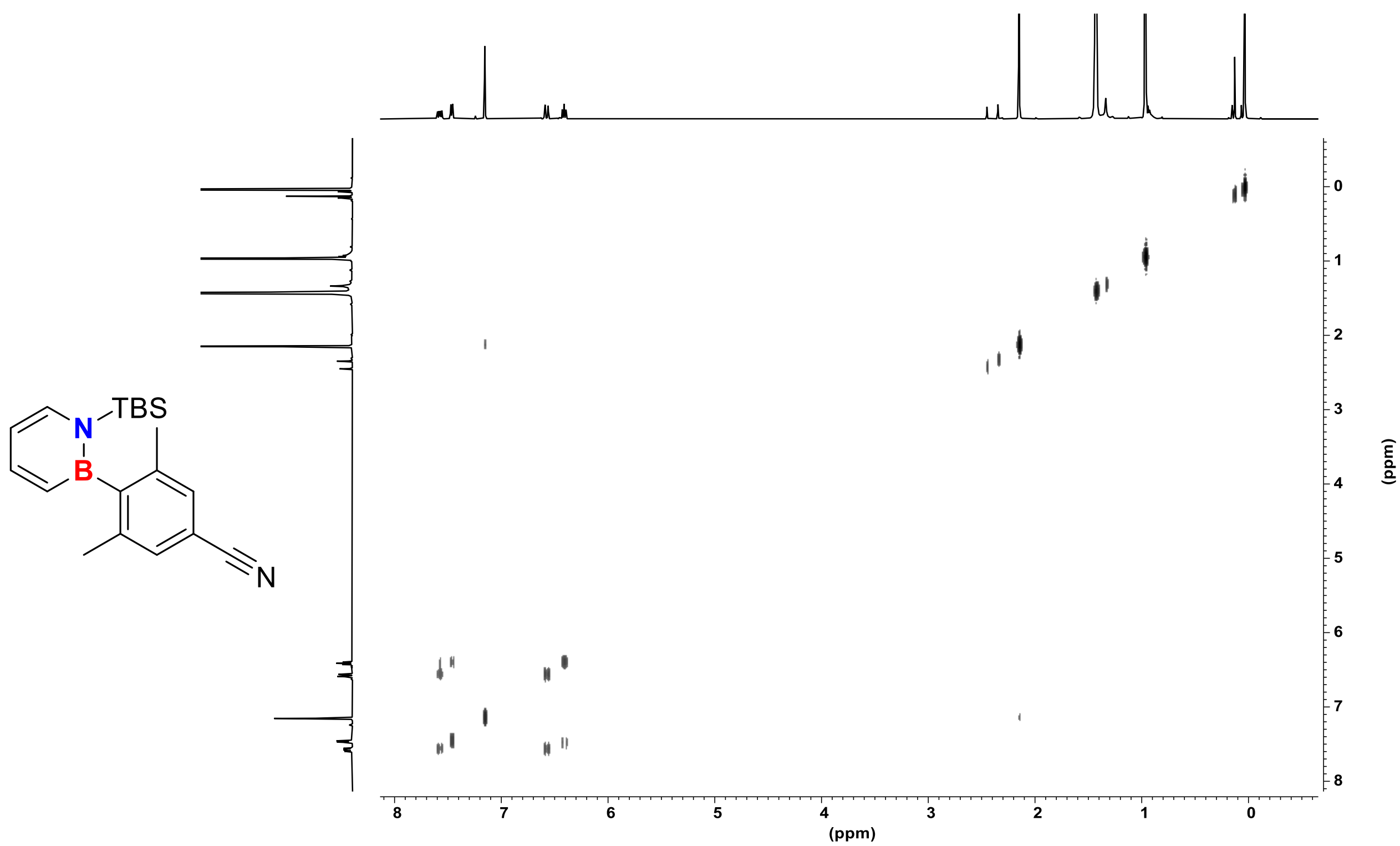

Figure S54: ^{1}H-^{1}H-COSY NMR spectrum of ^{BN}**B7** in C_6D_{12} measured on a 400 MHz spectrometer.

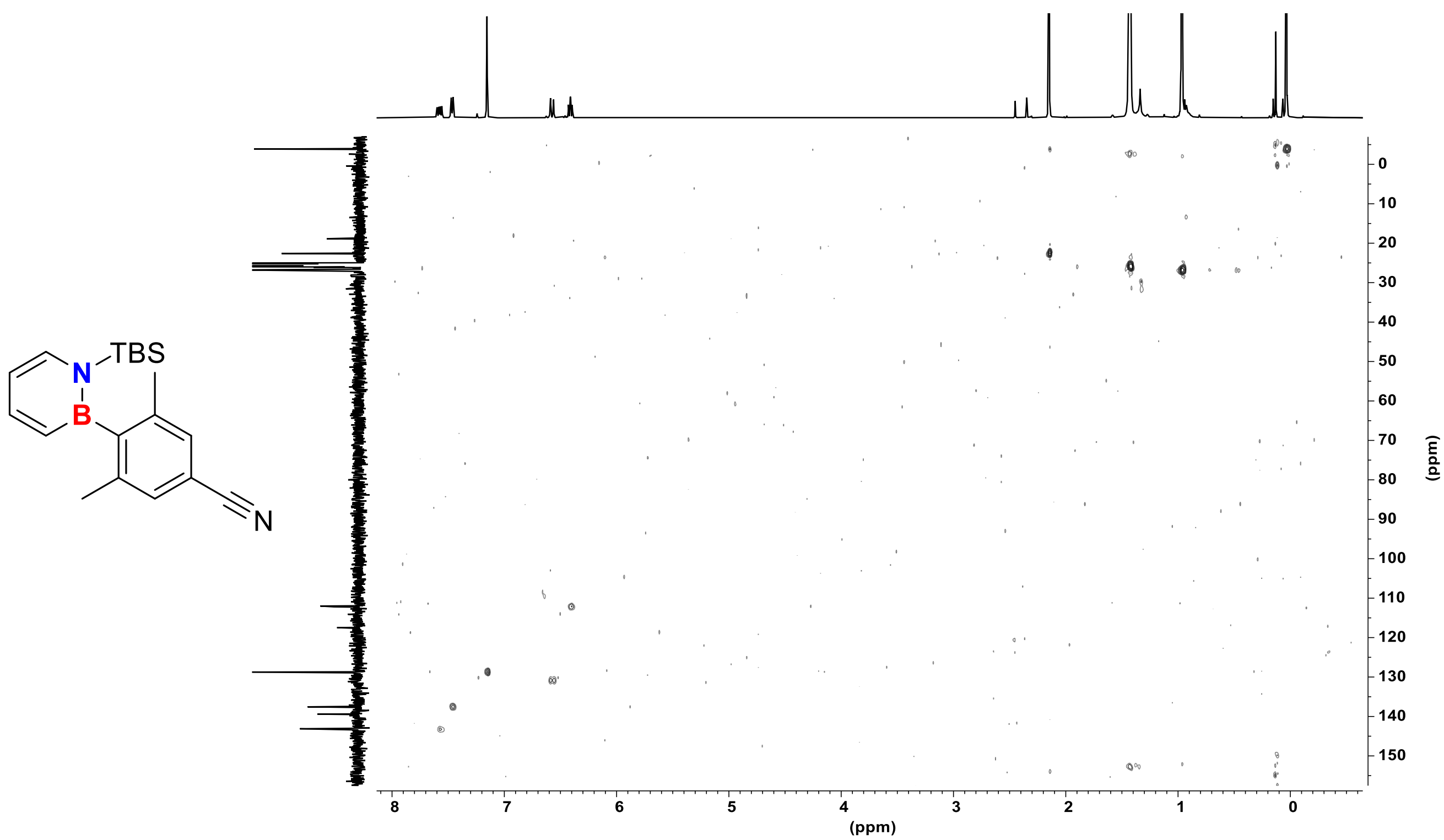

Figure S55: ^{1}H-^{13}C-HSQC NMR spectrum of ***BN*B7** in C_6D_{12} measured on a 400 MHz spectrometer.

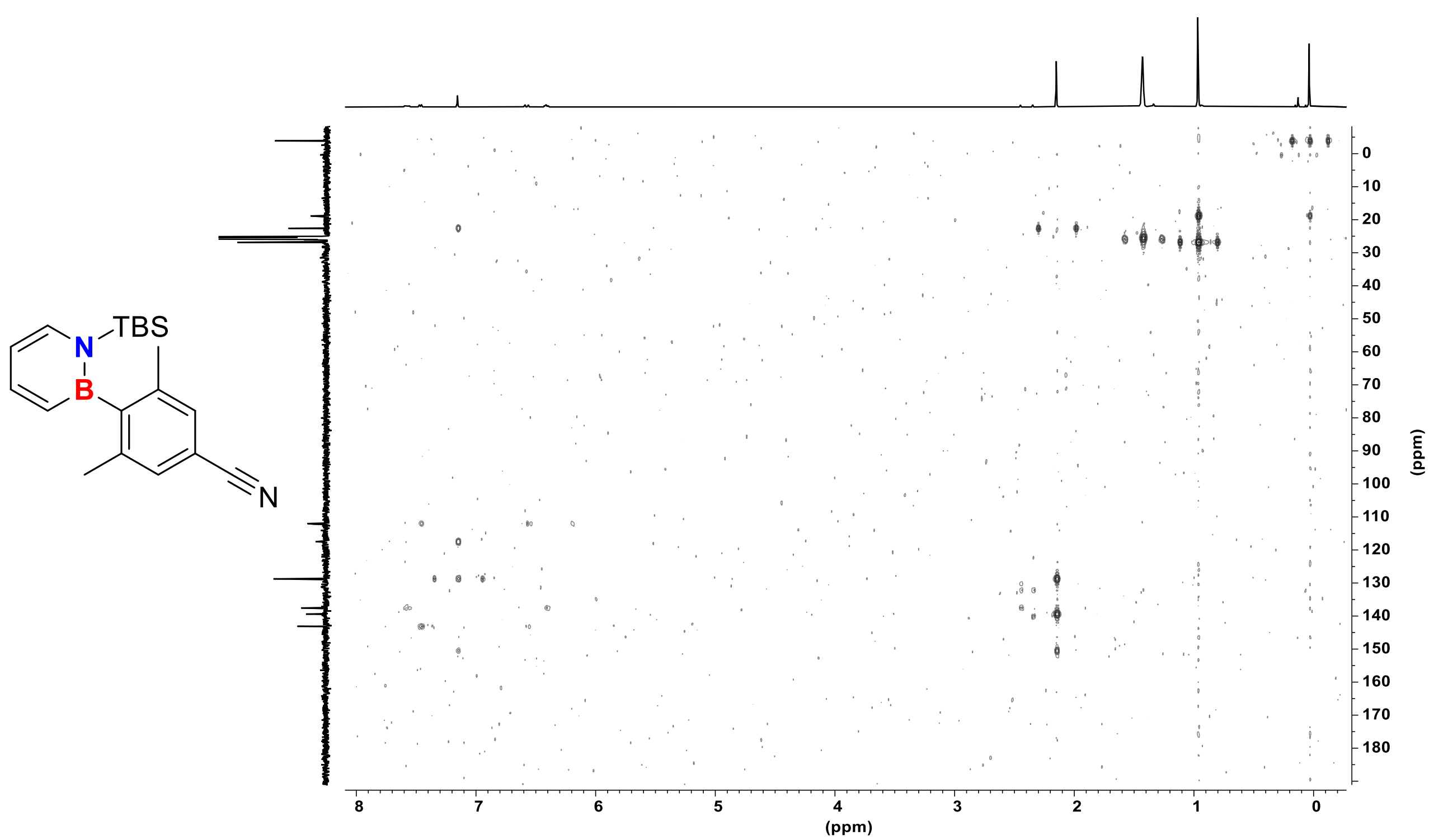

Figure S56: ^{1}H-^{13}C-HMBC NMR spectrum of ***BN*B7** in C_6D_{12} measured on a 400 MHz spectrometer.

3.2.5 2-(Pentamethylphenyl)-1,2-dihydro-1,2-azaborinine (*BN*B9)

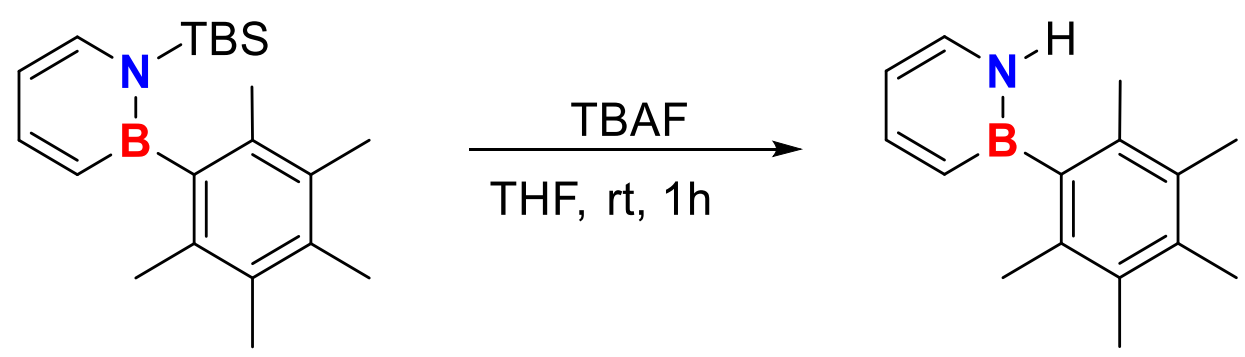

The synthesis is based on a procedure by Liu *et al.*[12]

Dihydroazaborinine ***BN*B1** (1.302 g, 3.8 mmol, 1 eq.) was dissolved in dry tetrahydrofuran (40 mL), and a solution of tetrabutylammonium fluoride (1 M in THF, 4.6 mL, 4.6 mmol, 1.2 eq.) was added dropwise. The reaction mixture was stirred at room temperature for 1 h and subsequently quenched with deionized water (20 mL). The aqueous phase was extracted with *n*-hexane (3 × 20 mL). The combined organic phases were dried over magnesium sulfate, filtered, and the solvent was removed under reduced pressure. After purification by column chromatography (silica gel, *n*-hexane/dichloromethane gradient), the product ***BN*B9** was obtained as a colorless solid (0.824 g, 3.7 mmol, 96%).

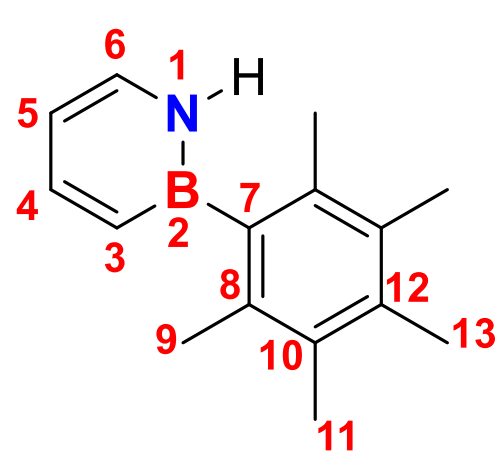

$C_{15}H_{20}BN$ (225.14 g/mol)

Mp = 150.4-152.0 °C (uncorrected)

^{1}H NMR (600 MHz, **C_6D_{12}**): δ = 7.56 (m, 1H, H-4), 7.32 (br. S, 1H, H-1), 7.10 (ps. t, 1H, H-6), 6.75 (d, 1H, H-3), 6.19 (ps. t, 1H, H-5), 2.18 (s, 3H, H-13), 2.13 (s, 6H, H-11), 2.00 (s, 3H, H-9) ppm.

^{13}C-{^{1}H} NMR (151 MHz, **C_6D_{12}**): δ = 142.9, 139.3, 134.3, 133.1, 132.4, 131.4, 130.7, 109.7, 22.6, 20.6, 15.3 ppm.

^{11}B-{^{1}H} NMR (193 MHz, **C_6D_{12}**): δ = 36.8 ppm.

HRMS (ASAP) m/z: [M + H]+ Calcd for C15H20BN 226.1762; Found 226.1760.

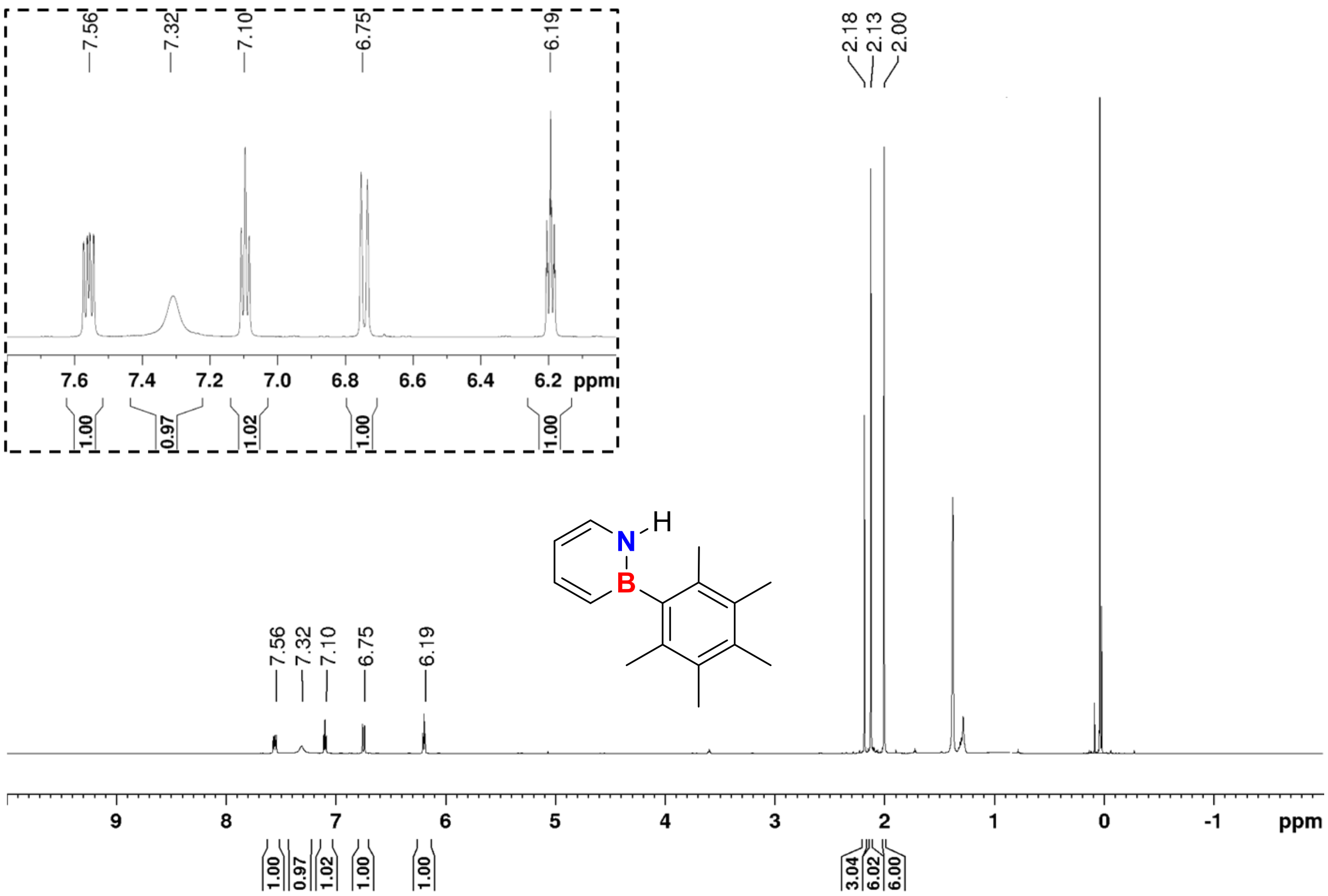

Figure S57. ^{1}H NMR spectrum of compound ^{BN}**B9** in C_6D_{12} measured on a 600 MHz spectrometer.

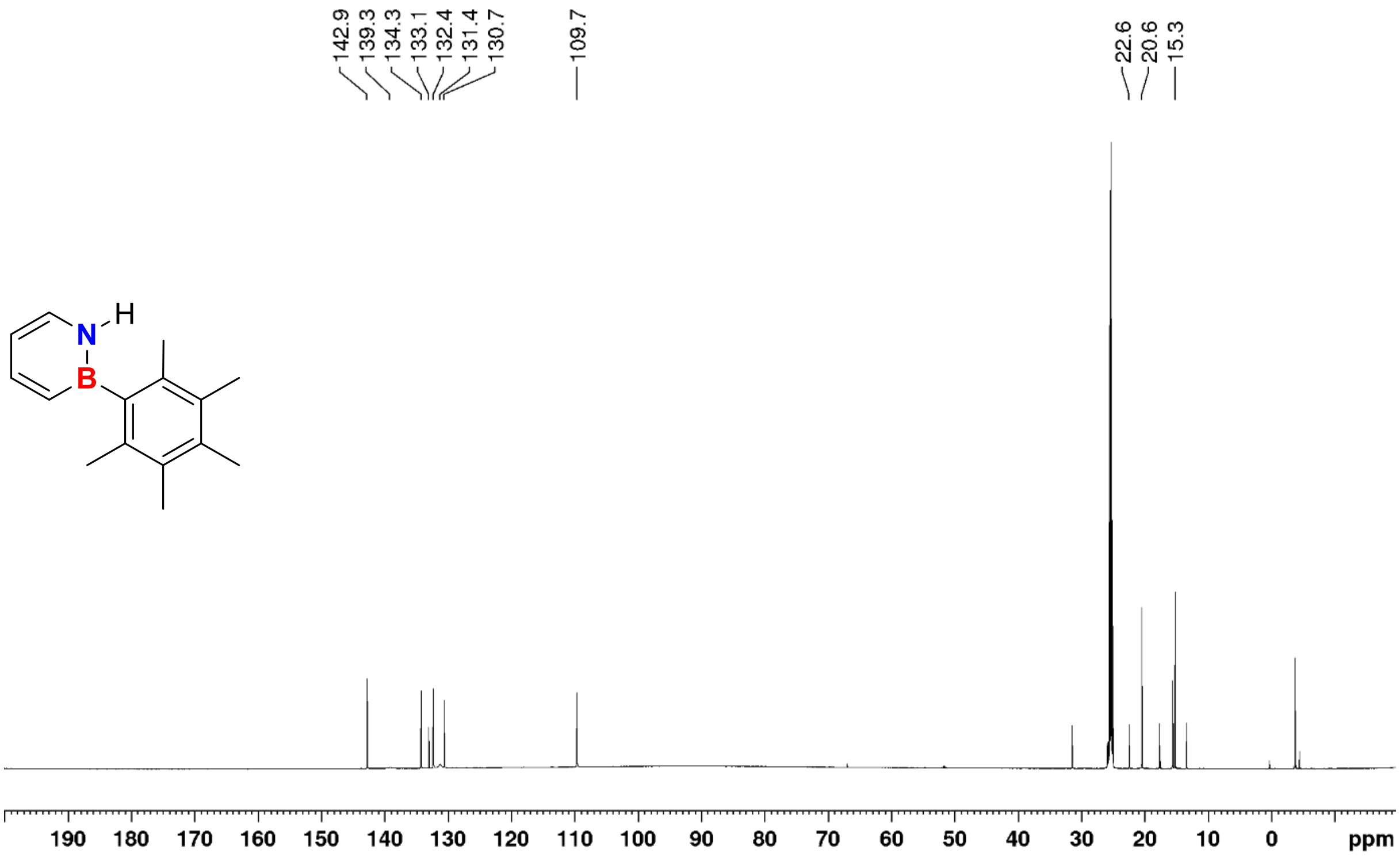

Figure S58. ^{13}C-{^{1}H} NMR spectrum of compound ***BN*B9** in C_6D_{12} measured on a 600 MHz spectrometer.

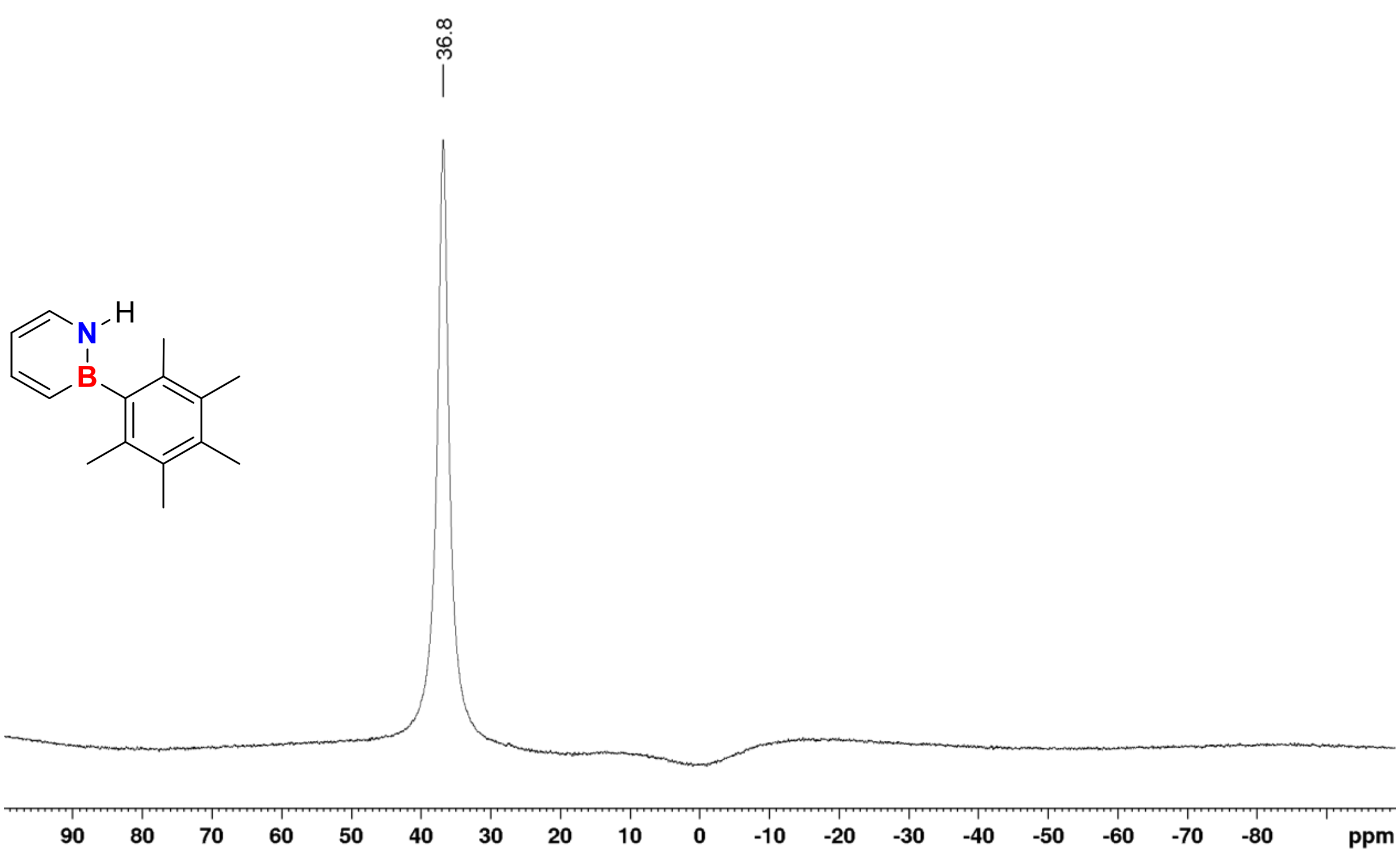

Figure S59. ^{11}B-{^{1}H} NMR spectrum of compound ***BN*B9** in C_6D_{12} measured on a 600 MHz spectrometer.

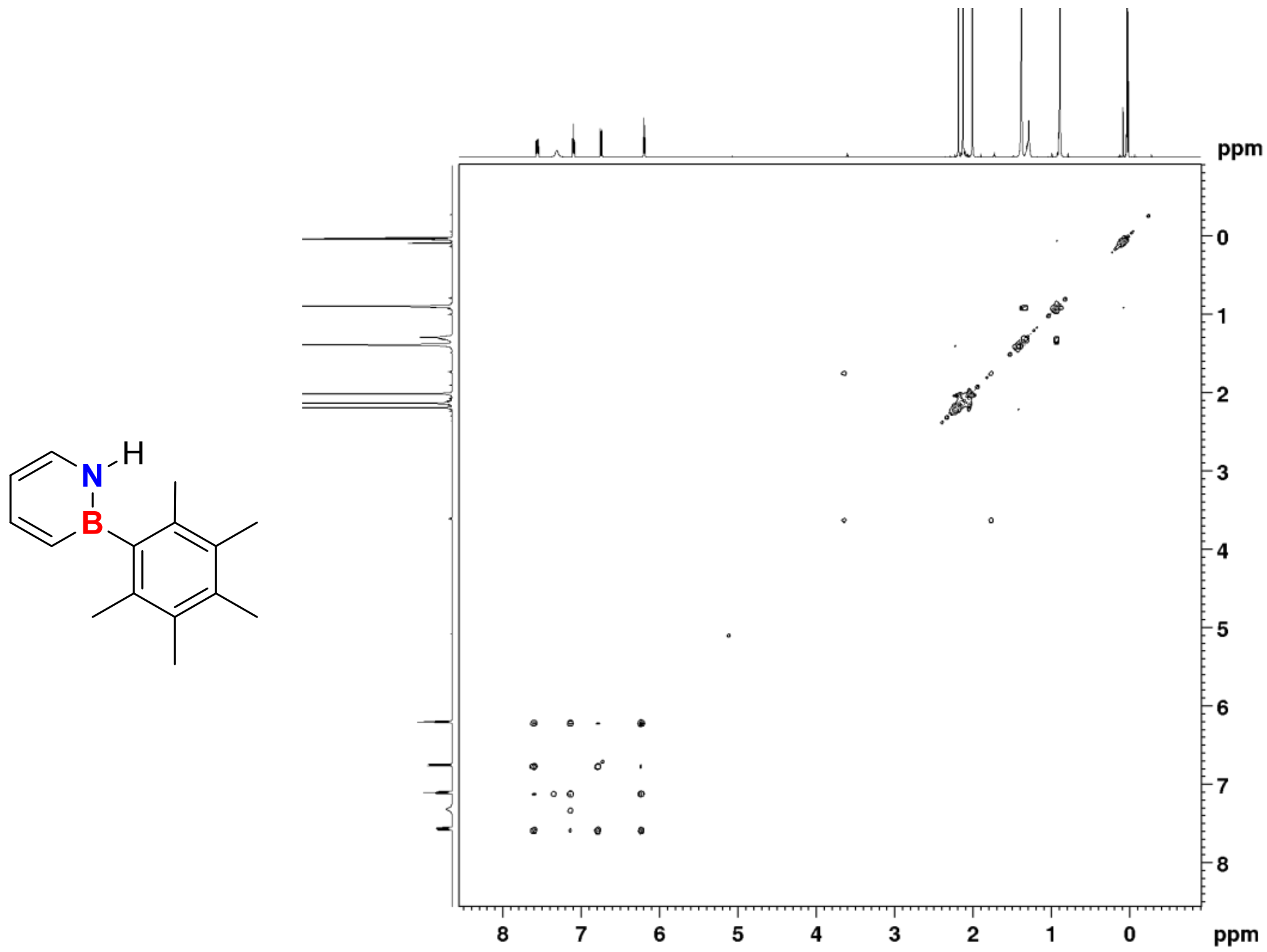

Figure S60. ^{1}H-^{1}H-COSY NMR spectrum of compound ^{BN}**B9** in C_6D_{12} measured on a 600 MHz spectrometer.

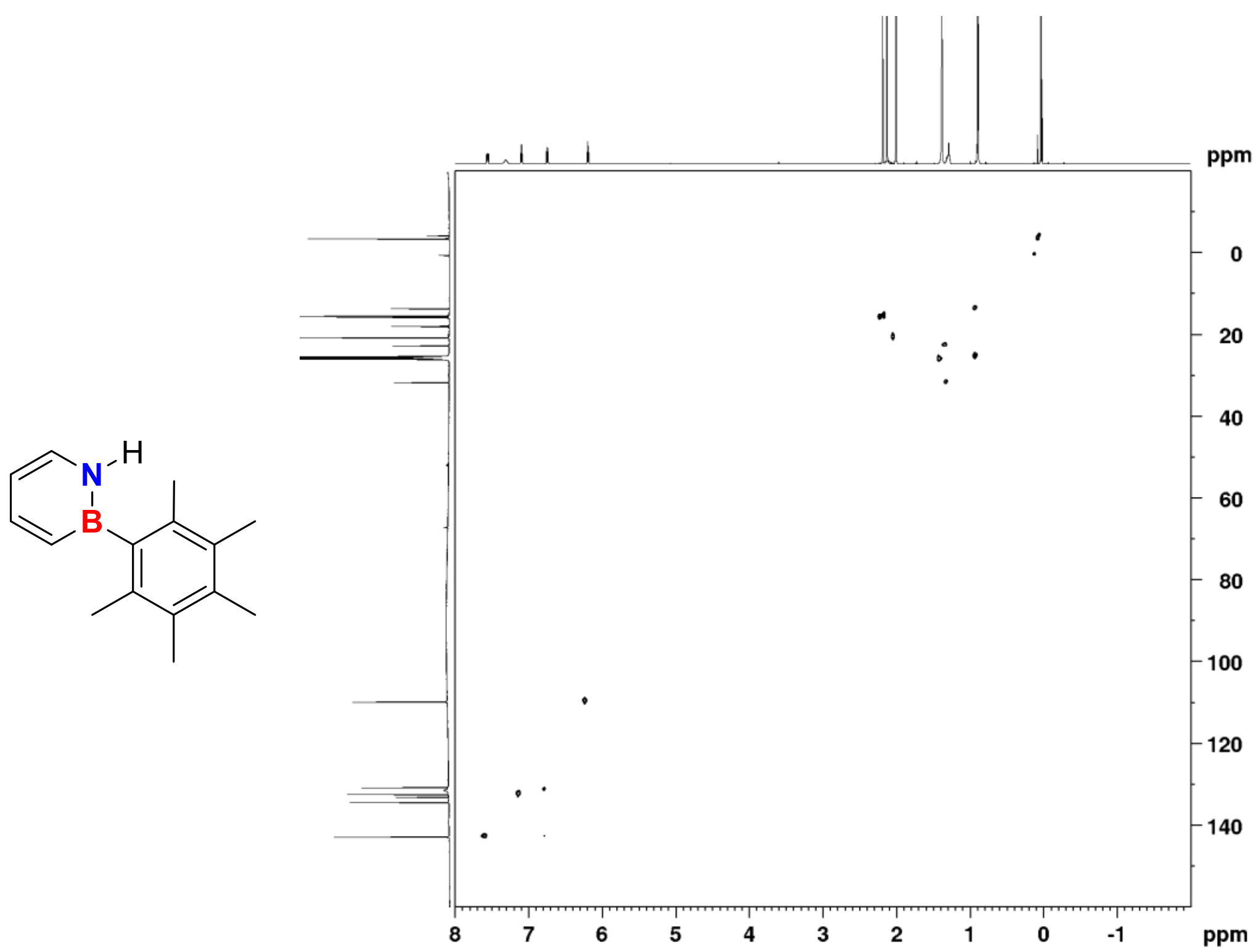

Figure S61. ^{1}H-^{13}C-HSQC NMR spectrum of compound ^{BN}**B9** in C_6D_{12} measured on a 600 MHz spectrometer.

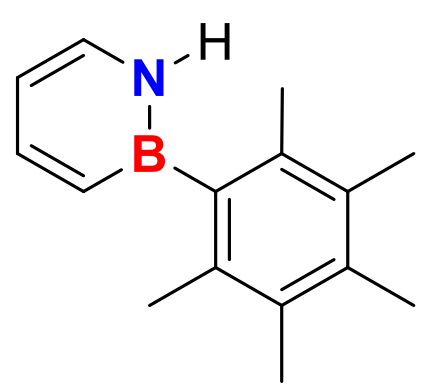

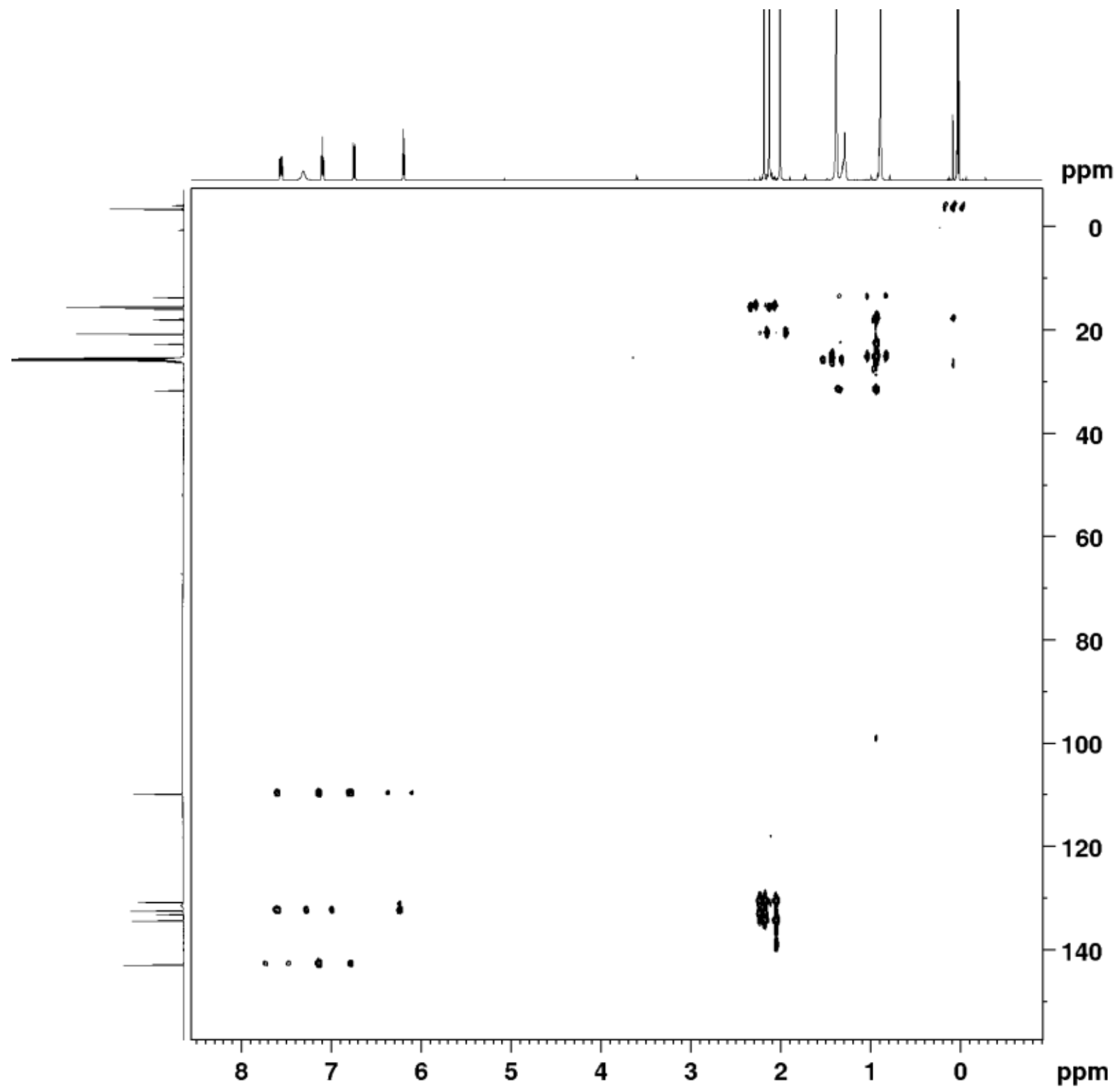

Figure S62. ^{1}H-^{13}C-HMBC NMR spectrum of compound ^{BN}**B9** in C_6D_{12} measured on a 600 MHz spectrometer.

3.2.9 1-Hexyl-2-mesityl-1,2-dihydro-1,2-azaborinine (^{BN}B11)

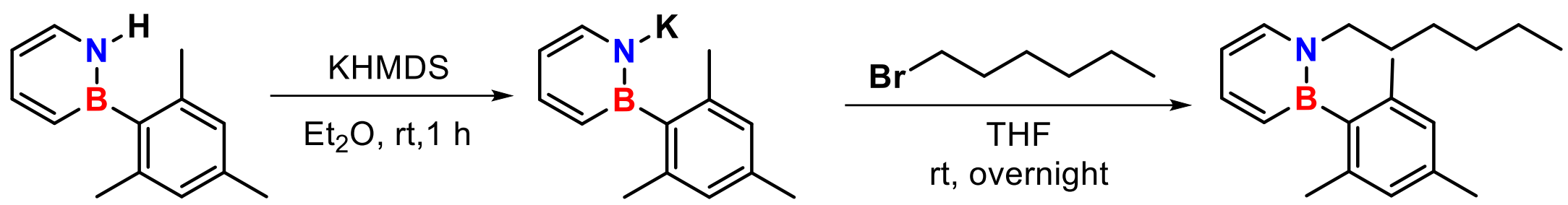

Synthesis is based on a protocol by Liu *et al.*[9]

To a solution of compound ^{BN}**B8** (132 mg, 0.67 mmol, 1 eq.) in diethyl ether (10 mL) was added a solution of potassium hexamethyldisilazide (0.5 M in toluene, 1.5 mL, 0.74 mmol, 1.1 eq.). The resulting reaction mixture was stirred at room temperature for 1 hour. Subsequently, it was centrifuged, the supernatant was removed and the obtained solid was washed with n-hexane and dried under reduced pressure. A portion of resulting 1-potassium-2-mesityl-1,2-dihydro-1,2-azaborinine (35 mg, 0.15 mmol, 1 eq.) was dissolved in THF (2 mL) and 1-bromohexane (23.3 µL, 0.17 mmol, 1.1 eq.) was added. The reaction mixture was stirred at room temperature overnight. After removing the solvent under reduced pressure, the crude product was purified by column chromatography (silica gel, *n*-hexane/CH_2Cl_2 gradient from 100:0 to 90:10). The product was obtained as a colorless oil in a yield of 22.0 mg (78 µmol, 52%).

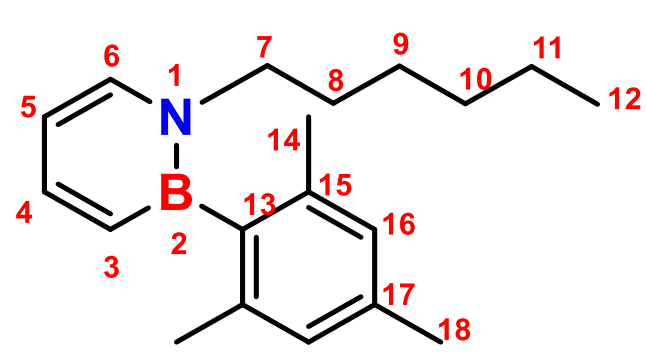

^{1}H NMR (400 MHz, **$CDCl_3$**): δ = 0.83 (m, 3H, H-12), 1.20 (m, 6H, H-9, H-10, H-11), 1.57 (m, 2H, H-8), 2.12 (s, 6H, H-14), 2.33 (s, 3H, H-18), 3.53 (m, 2H, H-7), 6.38 (ps. td, 1H, H-5), 6.73 (dd, $^3J_{HH}$ = 11.0, $^3J_{HH}$ = 1.0 Hz, 1H, H-3), 6.88 (s, 2H, H-16), 7.33 (d, $^3J_{HH}$ = 7.0 Hz, 1H, H-6), 7.61 (ddd, $^3J_{HH}$ = 11.1, $^3J_{HH}$ = 6.5, $^4J_{HH}$ = 1.1 Hz, 1H, H-4) ppm.

^{13}C-{^{1}H} NMR (101 MHz, **$CDCl_3$**): δ = 14.1 (C12), 21.3 (C16), 22.9 (C14), 22.5, 26.5, 31.5 (C9, C10, C11), 32.3 (C8), 53.9 (C7), 110.9 (C5), 127.2 (C16), 131.2 (s. br., C3), 136.7 (C17), 138.5 (C6), 139.5 (C13), 142.2 (C4) ppm.

C15 not detectable.

^{11}B-{^{1}H} NMR (128 MHz, **$CDCl_3$**): δ = 37.2 ppm.

HRMS (ASAP) m/z: [M + H]+ Calcd for C19H28BN 282.2388; Found 282.2389.

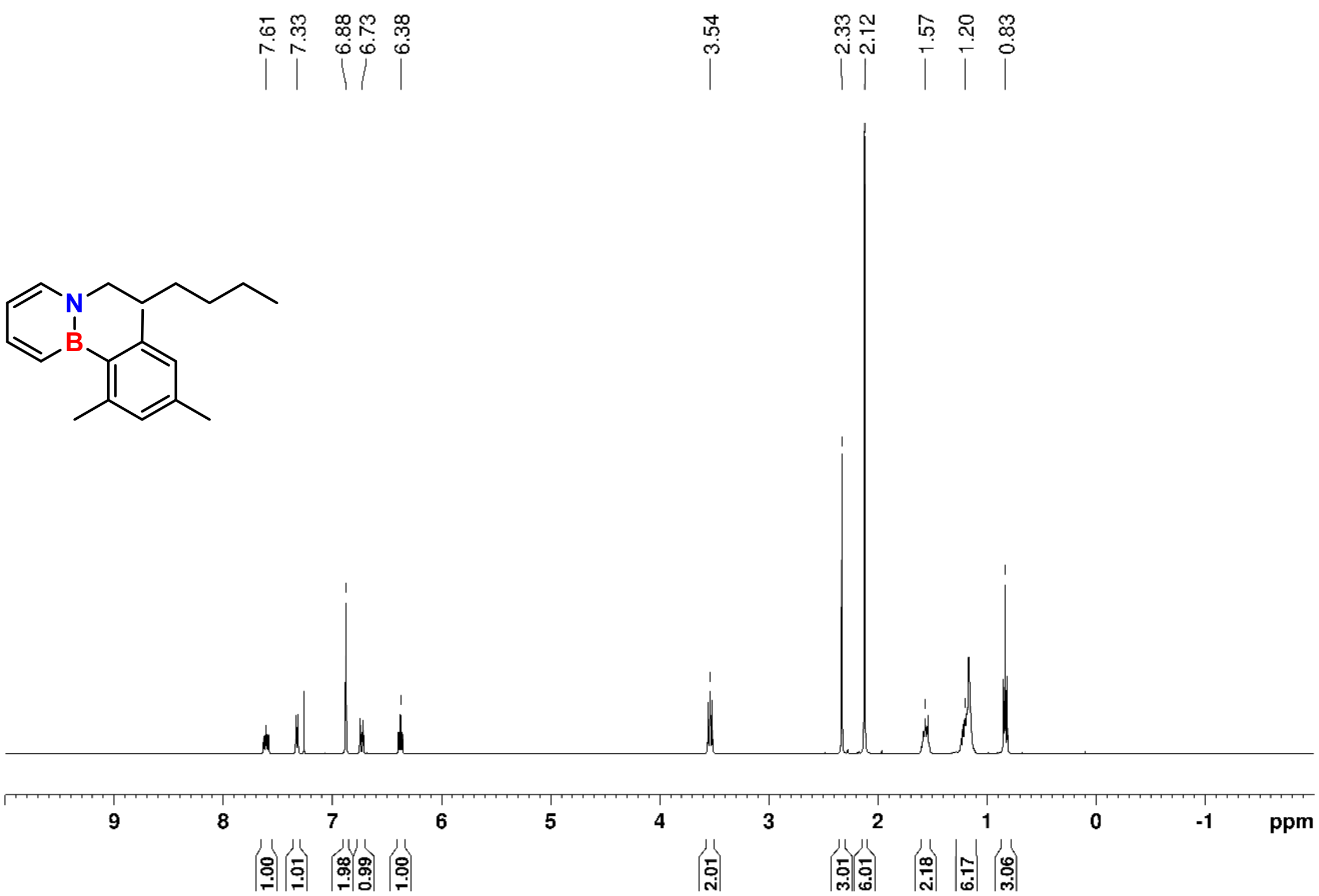

Figure S63. ^{1}H NMR spectrum of ^{BN}**B11** in $CDCl_3$ measured on a 400 MHz spectrometer.

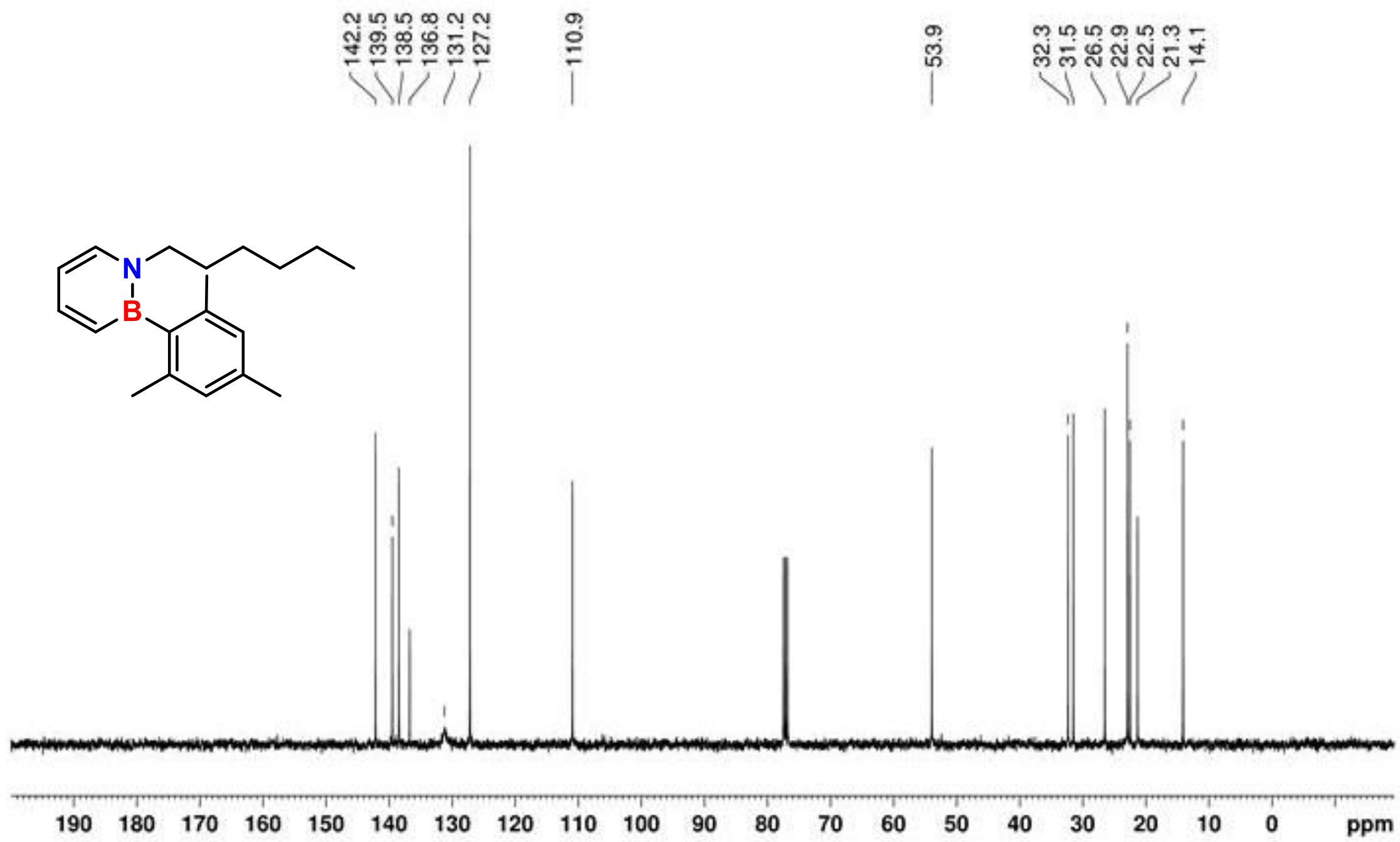

Figure S64. ^{13}C-{^{1}H} NMR spectrum of ***BN*B11** in $CDCl_3$ measured on a 400 MHz spectrometer.

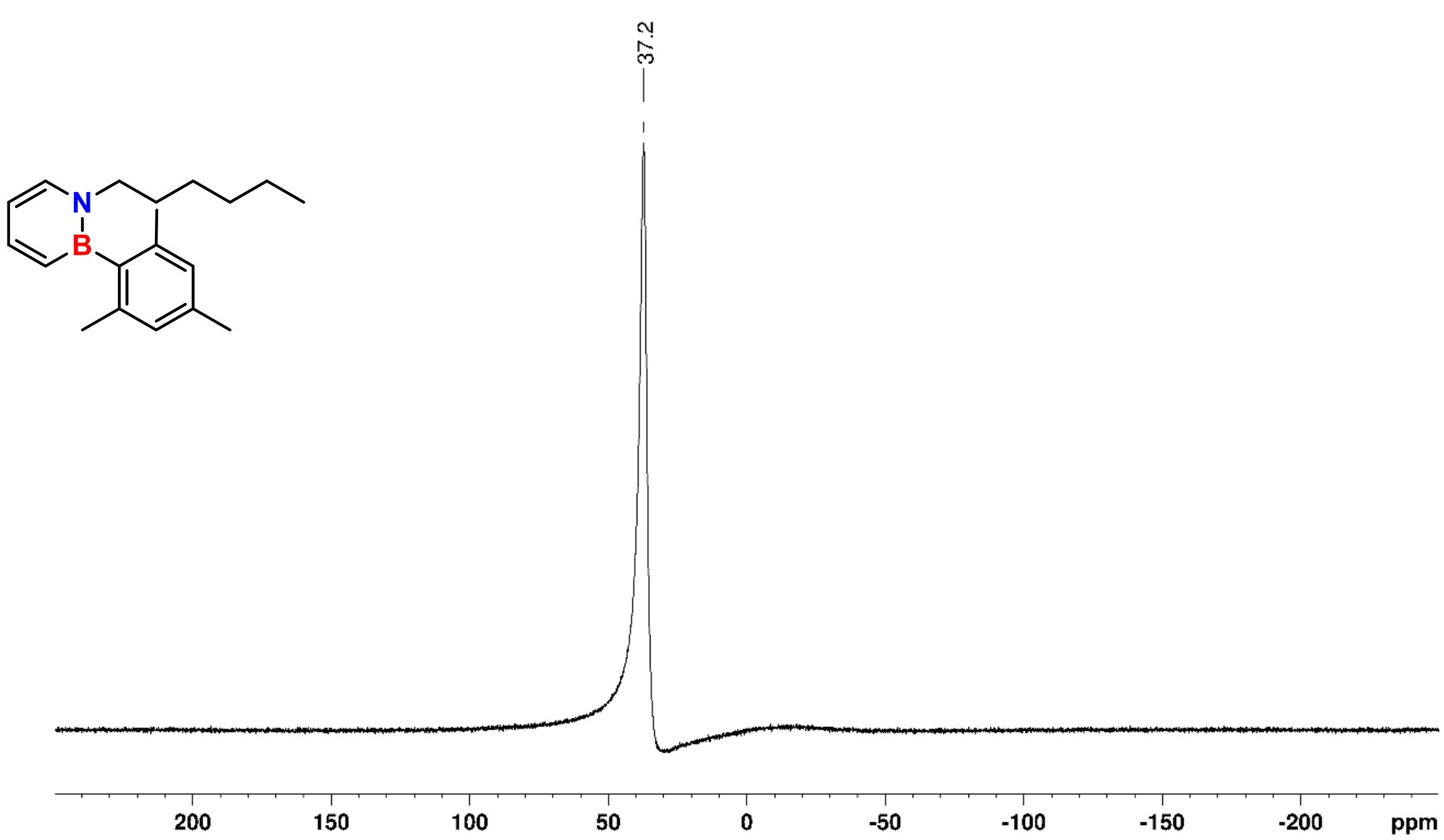

Figure S65. ^{11}B-{^{1}H} NMR spectrum of ***BN*B11** in $CDCl_3$ measured on a 400 MHz spectrometer.

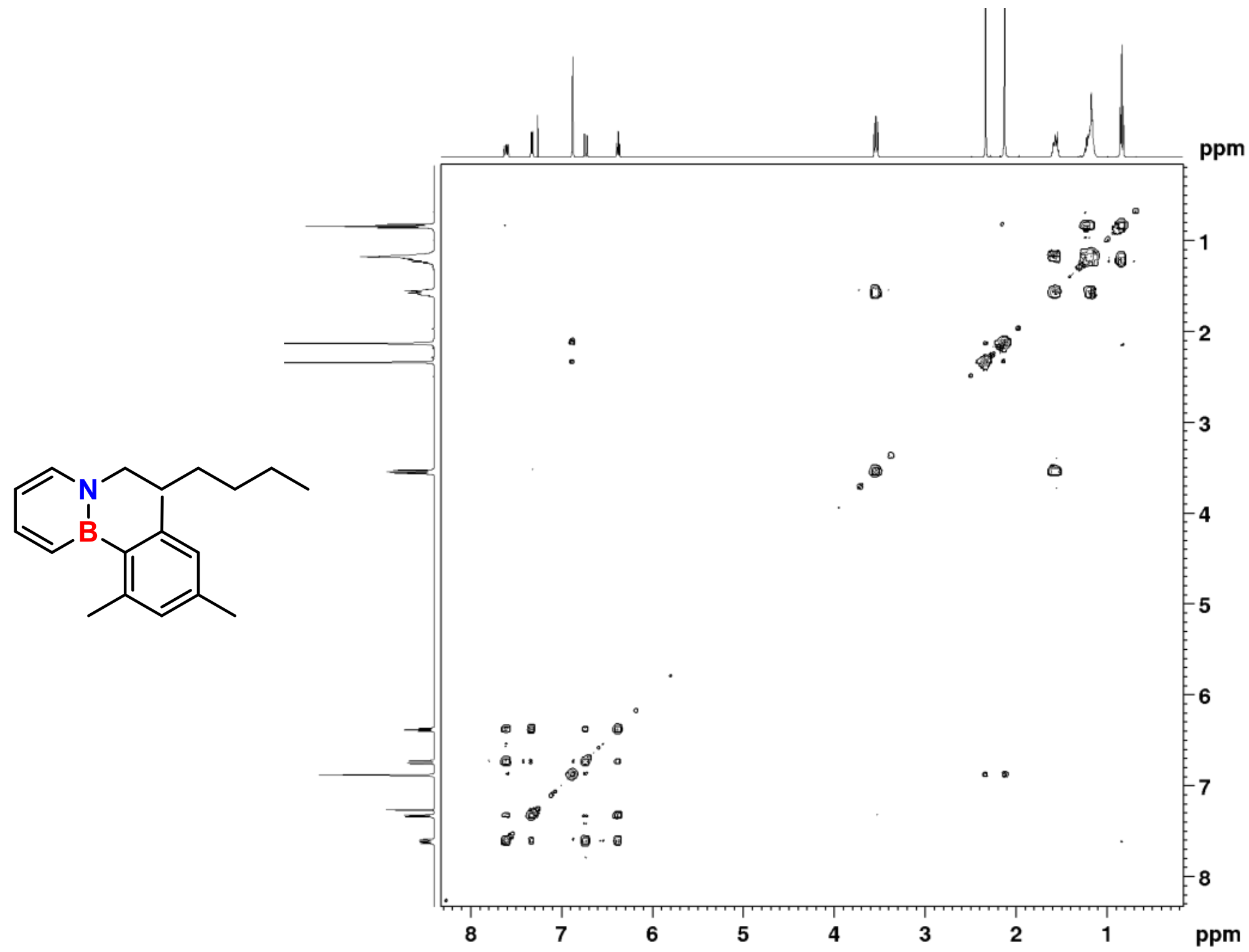

Figure S66. ^{1}H-^{1}H-COSY NMR spectrum of ^{BN}**B11** in $CDCl_3$ measured on a 400 MHz spectrometer.

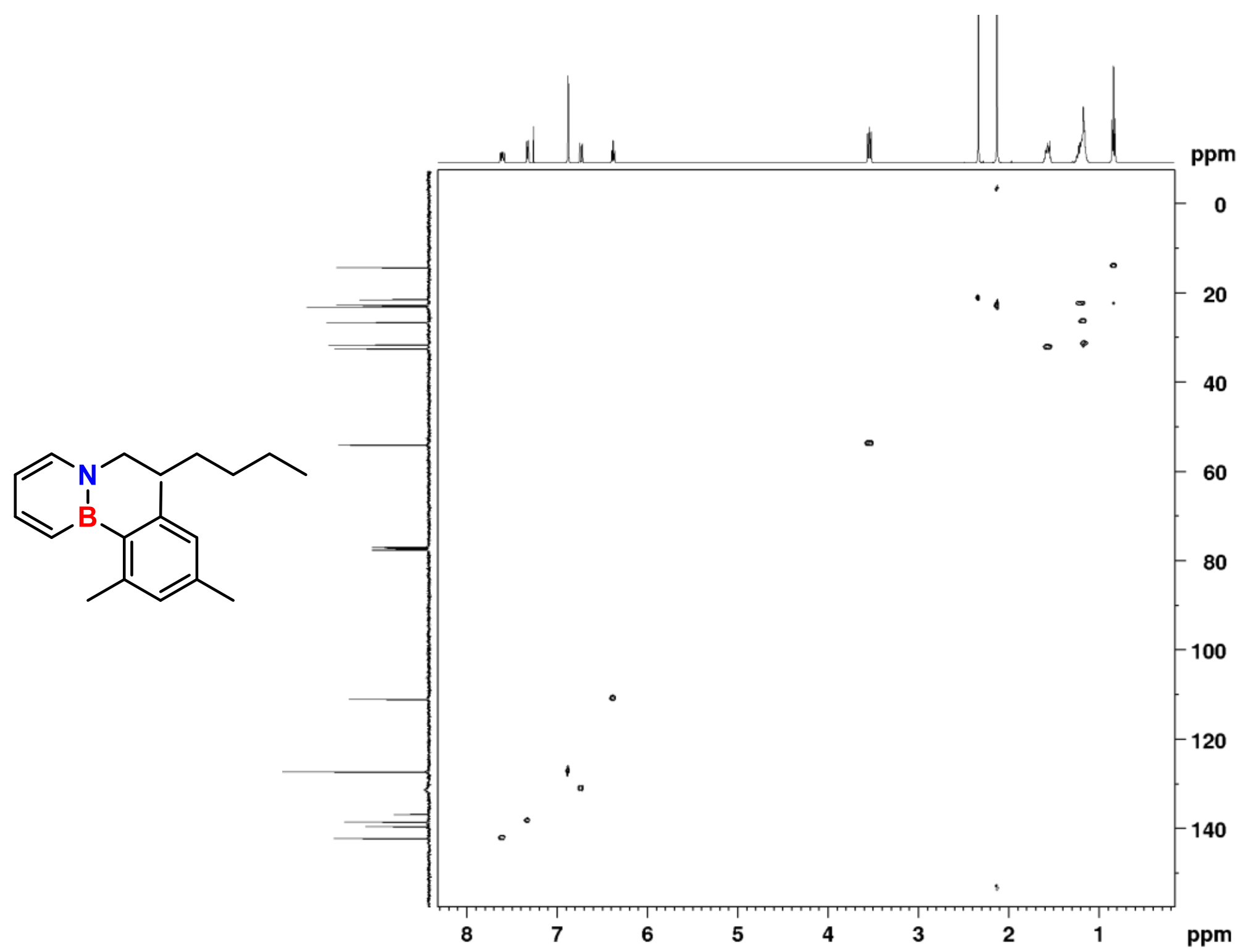

Figure S67. ^{1}H-^{13}C-HSQC NMR spectrum of ^{BN}**B11** in $CDCl_3$ measured on a 400 MHz spectrometer.

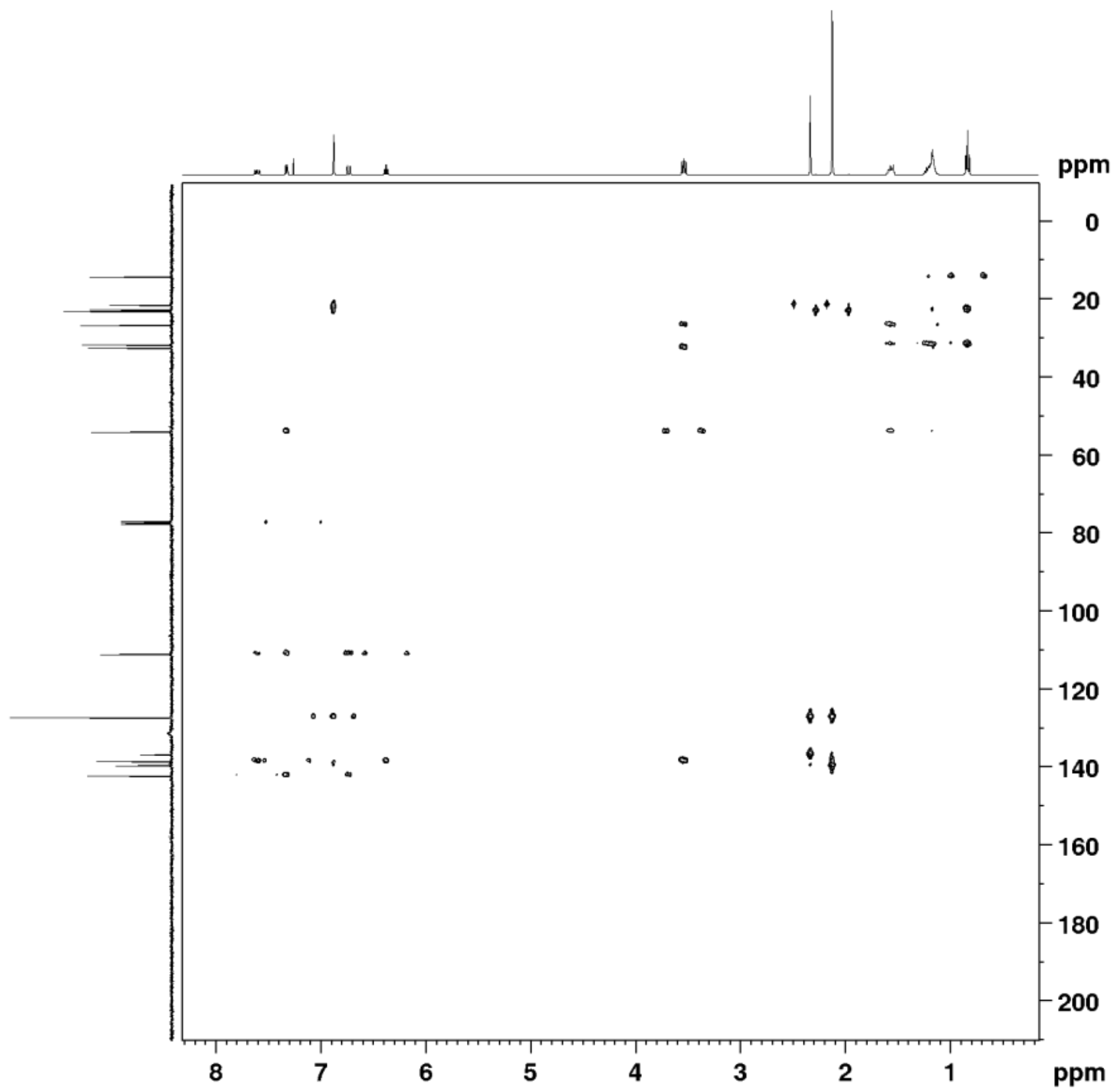

Figure S68. ^{1}H-^{13}C-HMBC NMR spectrum of ^{BN}**B11** in $CDCl_3$ measured on a 400 MHz spectrometer.

3.2.6 1-Trimethylsilyl-2-mesityl-1,23-dihydro-1,2-azaborinine (^{BN}B13)

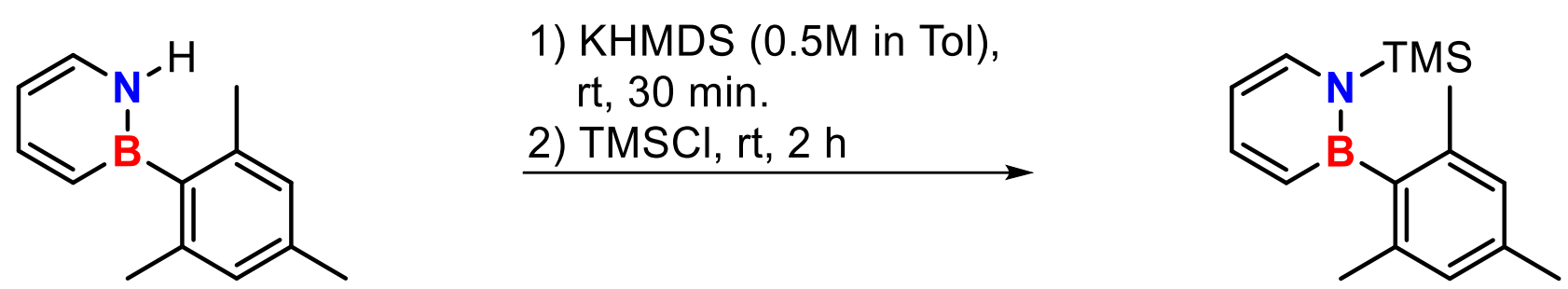

Synthesis is based on a protocol by Liu *et al*.[13]

To a solution of compound ^{BN}**B8** (87 mg, 0.44 mmol, 1 eq.) was added a solution of potassium hexamethyldisilazide (0.5 M in toluene, 1.8 mL, 0.88 mmol, 2 eq.). The resulting reaction mixture was stirred at room temperature for 30 min and subsequently treated with trimethylsilyl chloride (0.12 mL, 0.88 mmol, 2 eq.), whereupon the solution rapidly became colorless, and a white precipitate formed. After an additional 2 h of reaction at room temperature, the solvent was removed under reduced pressure, and the crude product was purified by column chromatography (silica gel, *n*-hexane/CH_2Cl_2 gradient from 100:0 to 95:5). Product ^{BN}**B13** was obtained as a colorless oil in a yield of 78 mg (0.29 mmol, 66%).

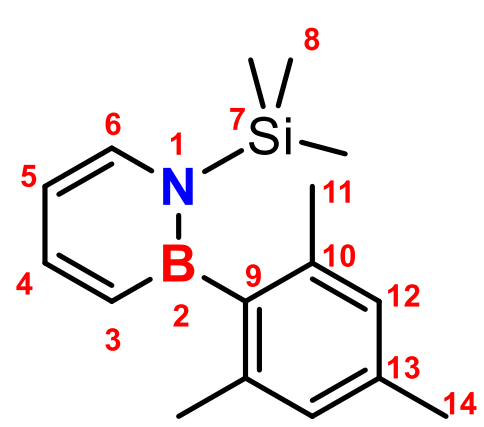

^{1}H NMR (300 MHz, **CD_2Cl_2**): δ = 0.11 (s, 9H, H-8), 2.03 (s, 6H, H-11), 2.27 (s, 3H, H-14), 6.42 (ps. dt, 1H- H-5), 6.57 (dm, $^3J_{HH}$ = 11.00 Hz, 1H- H-3), 6.78 (m, 2H, H-12), 7.48 (d, $^3J_{HH}$ = 6.76 Hz, 1H, H-6), 7.57 (ddd, $^3J_{HH}$ = 10.55 Hz, $^3J_{HH}$ = 6.29 Hz, $^4J_{HH}$ = 1.06 Hz, 1H, H-4) ppm.

^{13}C-{^{1}H} NMR (101 MHz, **CD_2Cl_2**): δ = 1.2 (C8), 21.3 (C14), 23.5 (C11), 112.4 (C5), 127.1 (C12), 131.3 (C3, s. br.), 136.9 (C13, s. br.), 138.0 (C6), 139.4 (C10), 142.2 (C9, s. br.), 143.3 (C4) ppm.

^{11}B-{^{1}H} NMR (128 MHz, **CD_2Cl_2**): δ = 39.7 ppm.

HRMS (ASAP) m/z: [M + H]+ Calcd for C16H24BNSi 270.1844, found 270.1842.

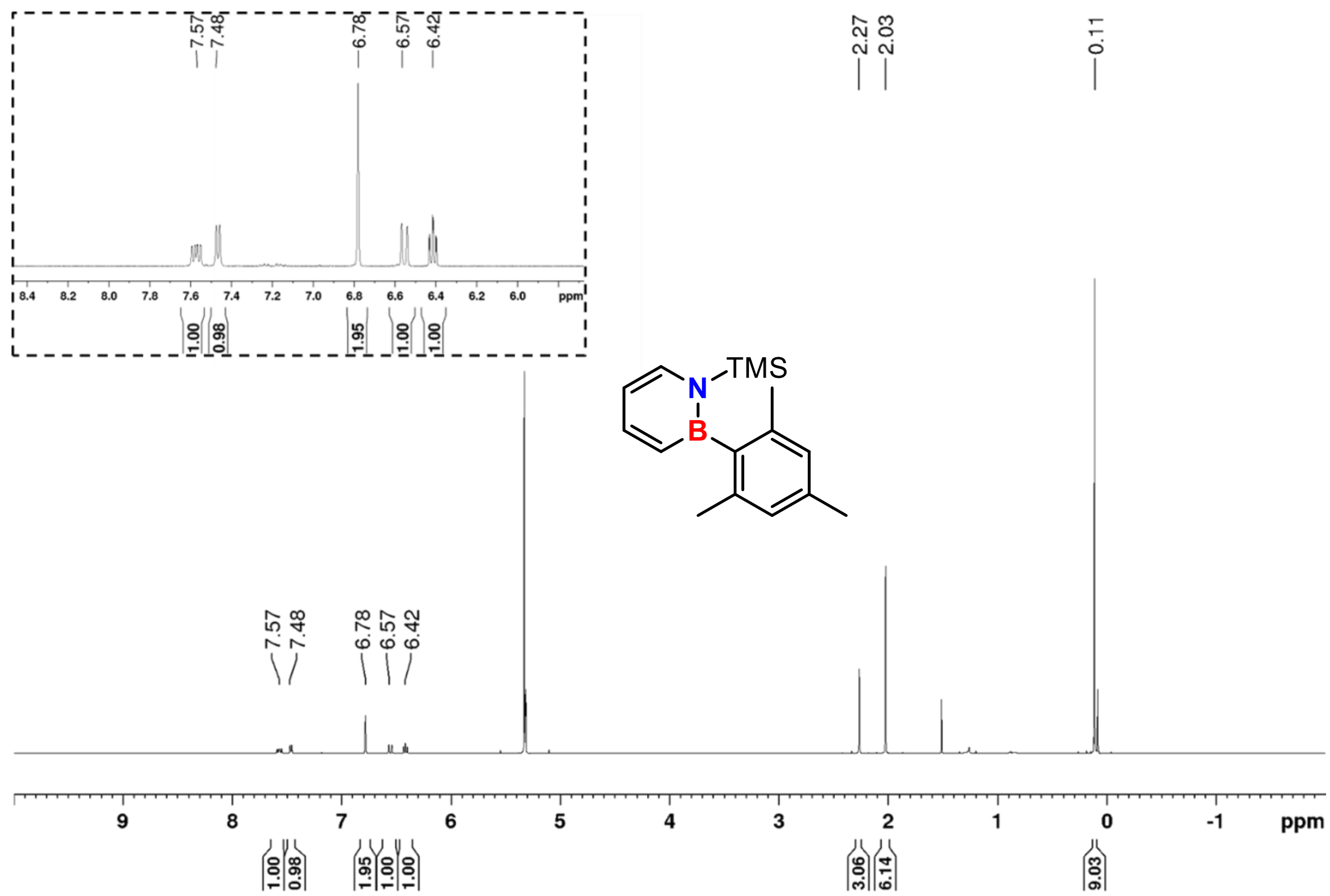

Figure S69: ^{1}H NMR spectrum of ***BN*****B13** in CD_2Cl_2 measured on a 400 MHz spectrometer.

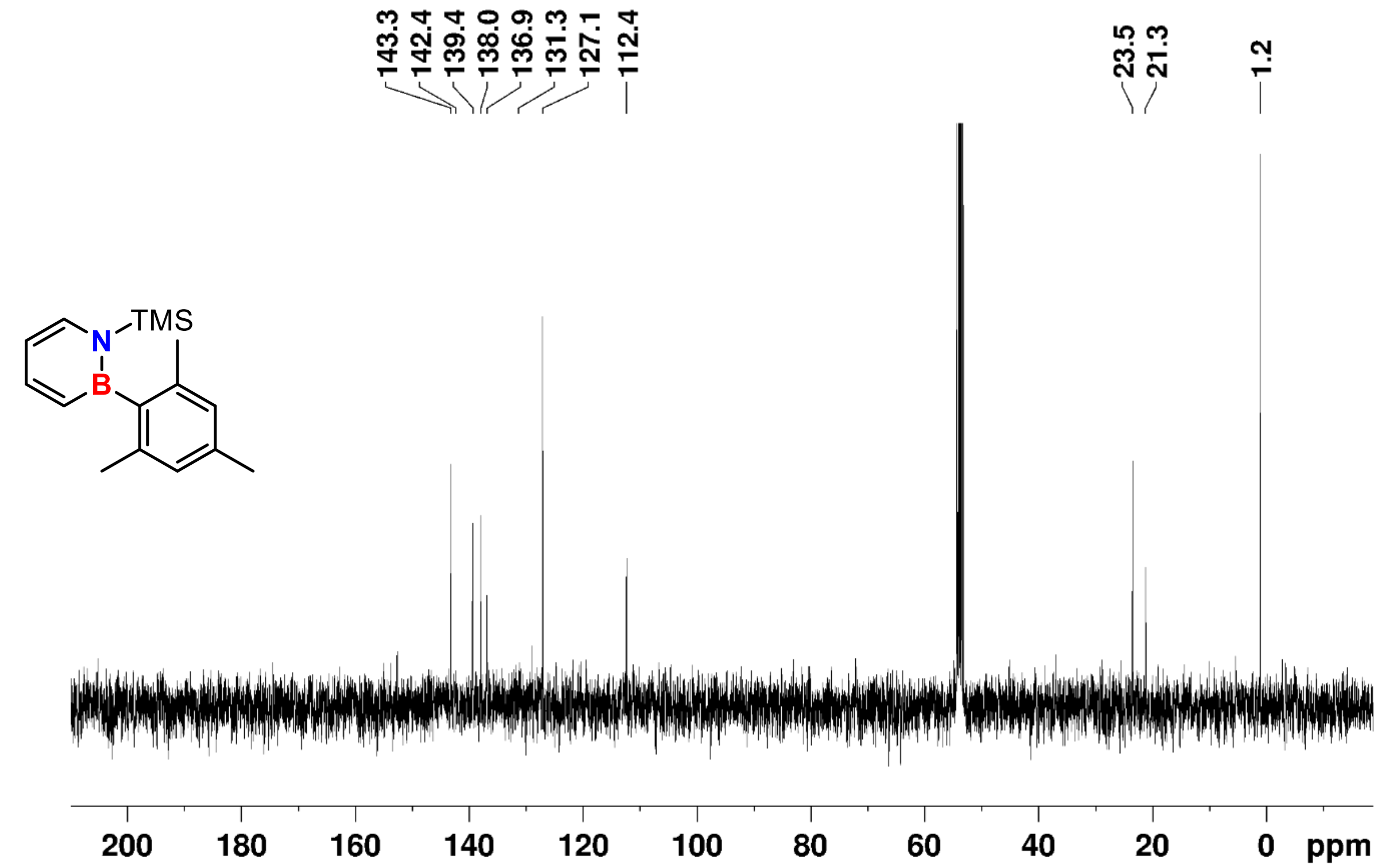

Figure S70: ^{13}C-{^{1}H} NMR spectrum of ^{BN}**B13** in CD_2Cl_2 measured on a 400 MHz spectrometer.

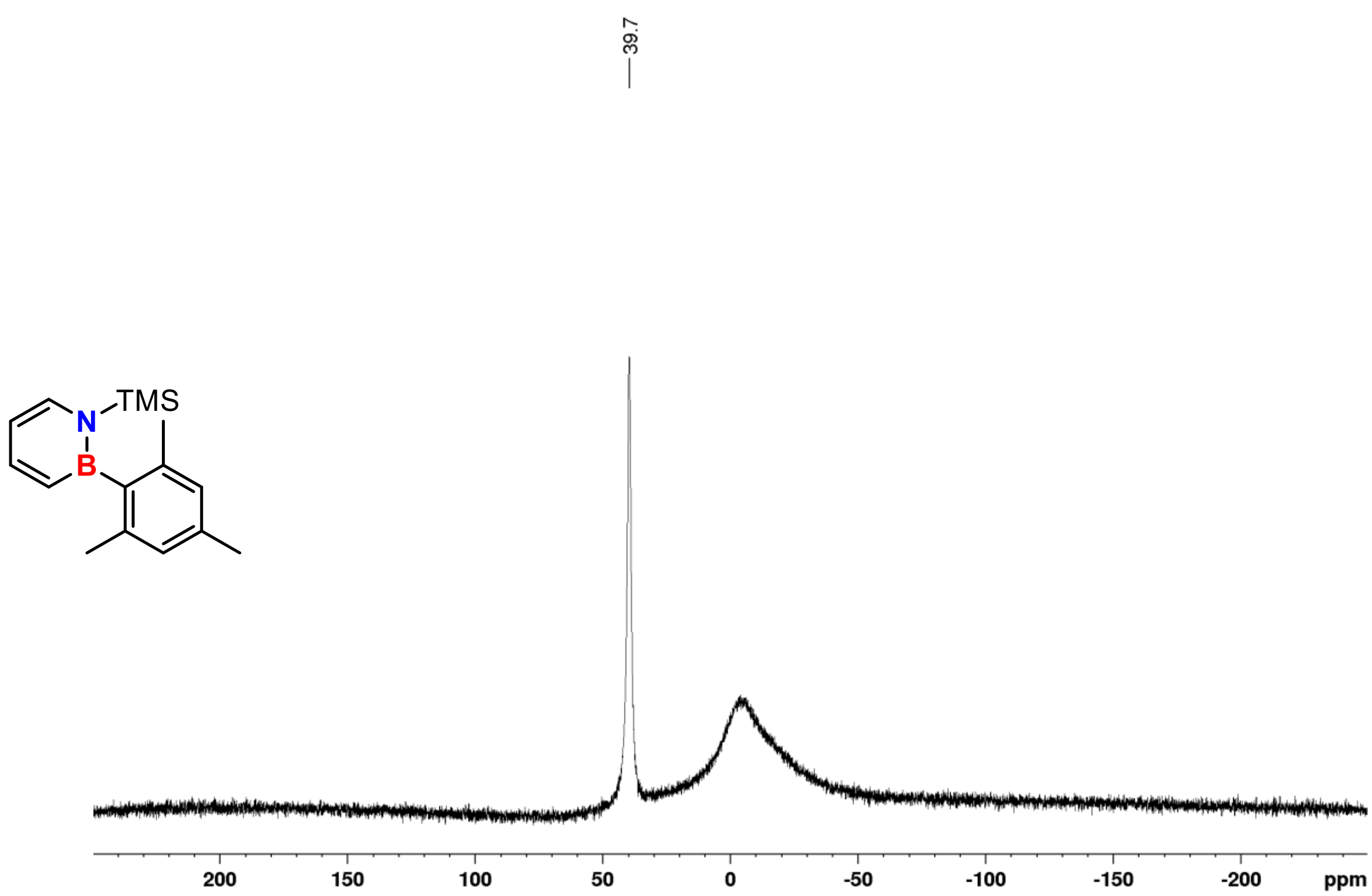

Figure S71: ^{11}B-{^{1}H} NMR spectrum of ^{BN}**B13** in CD_2Cl_2 measured on a 400 MHz spectrometer.

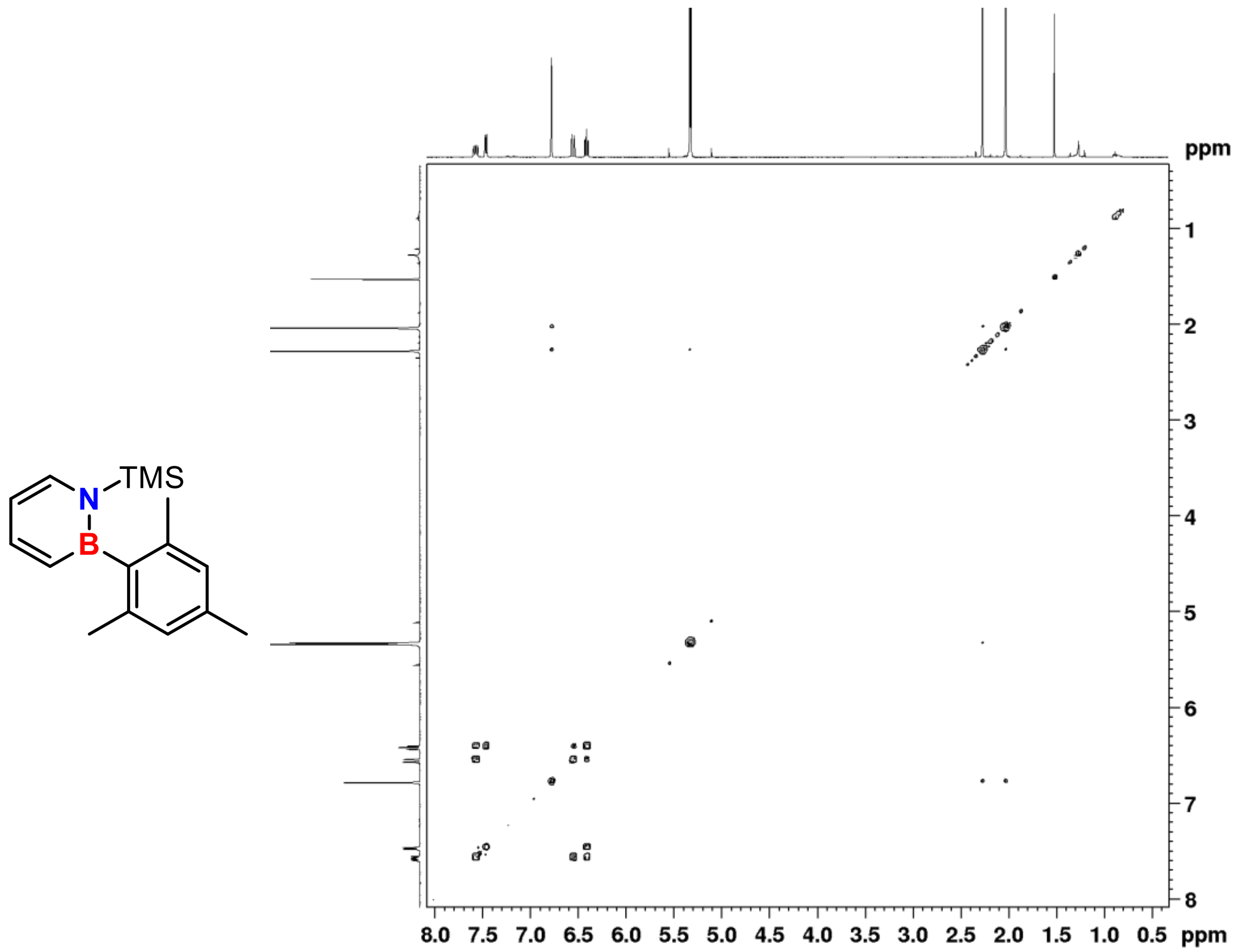

Figure S72: ^{1}H-^{1}H-COSY NMR spectrum of ***BN*B13** in CD_2Cl_2 measured on a 400 MHz spectrometer.

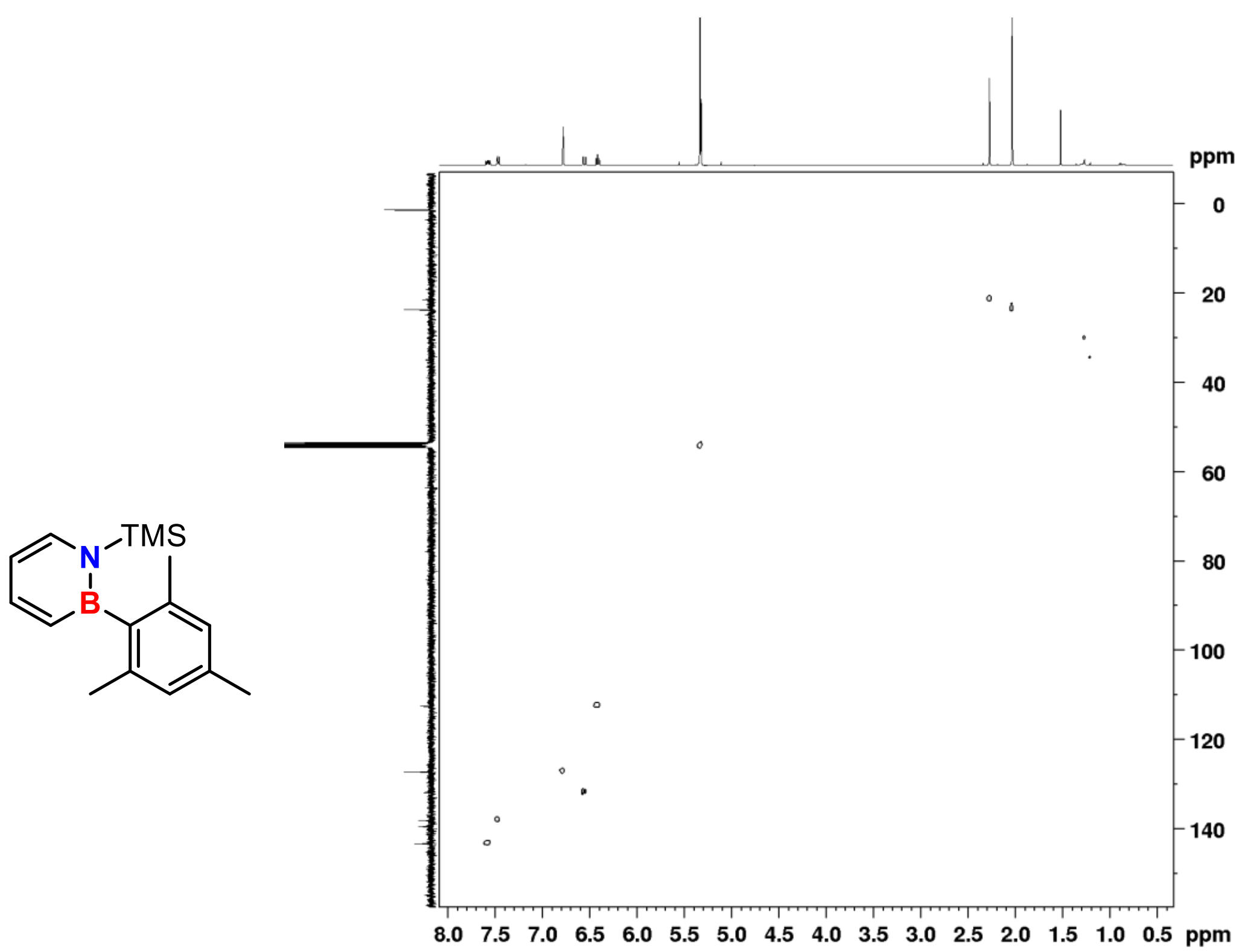

Figure S73: ^{1}H-^{13}C NMR spectrum of ***BN*B13** in CD_2Cl_2 measured on a 400 MHz spectrometer.

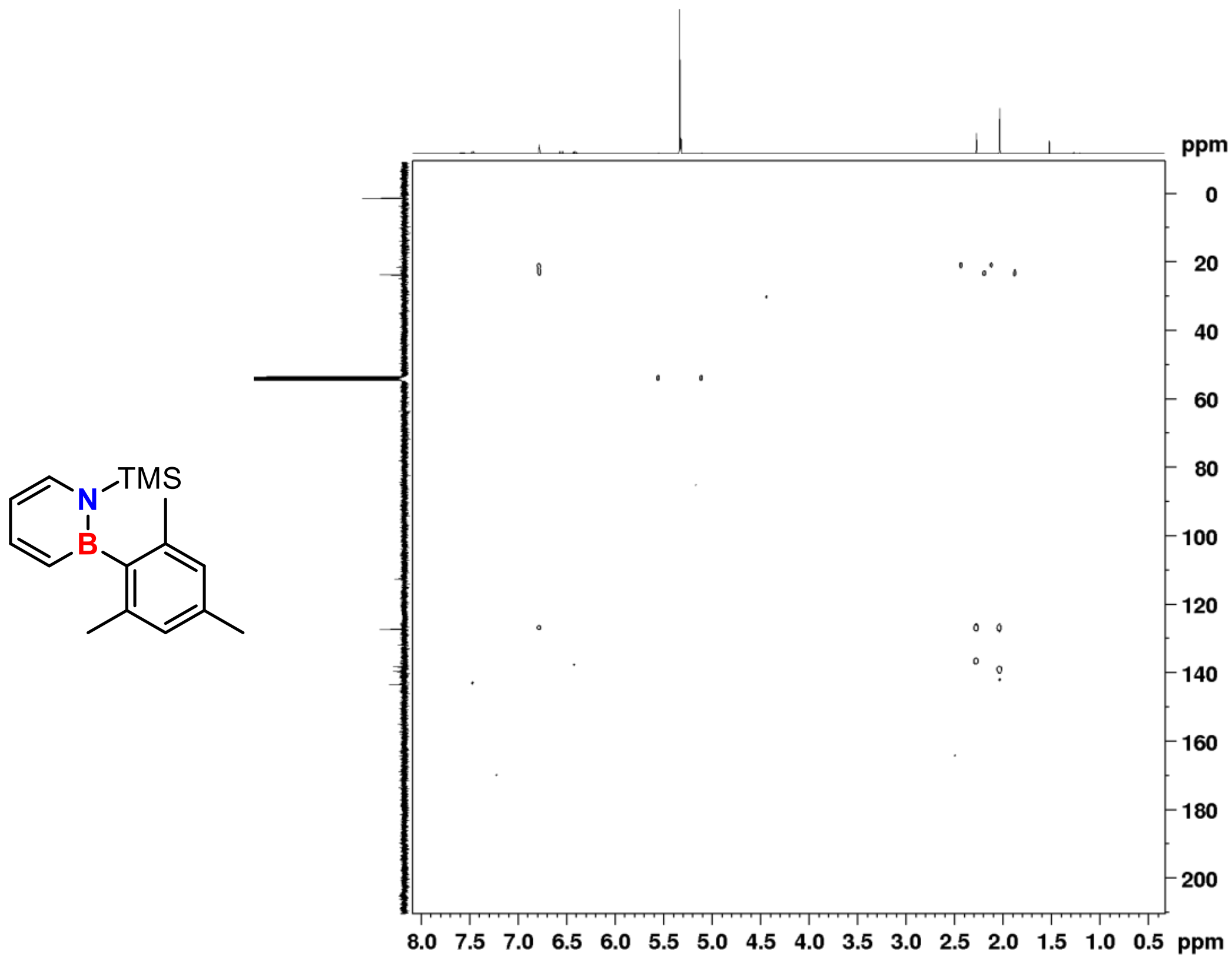

Figure S74: ^{1}H-^{13}C NMR spectrum of ^{BN}**B13** in CD_2Cl_2 measured on a 400 MHz spectrometer.

4. Irradiation experiments

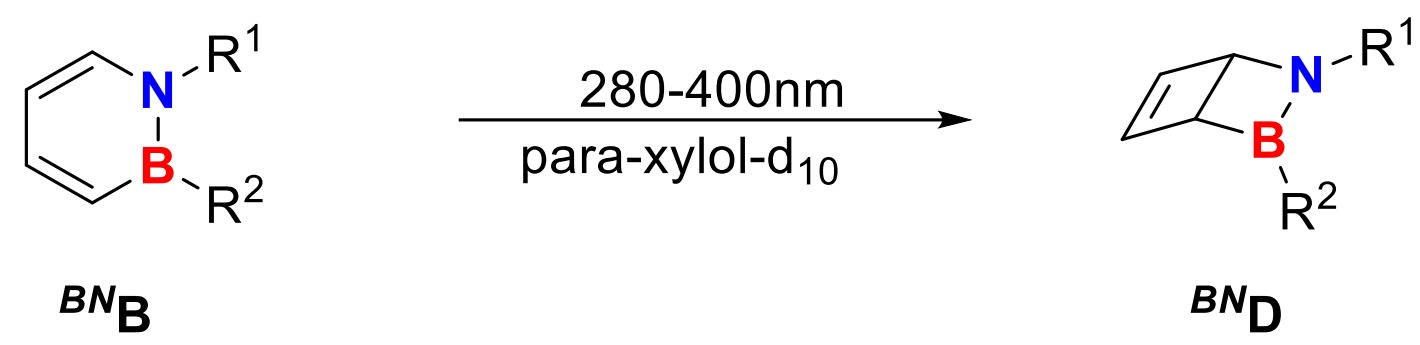

All irradiations were carried out using a dichroic mirror that selects the wavelength range 280 – 400 nm, without additional filters. The samples were placed in quartz J. Young NMR tubes and solved in deuterated p-xylol-d_{10} under argon before the irradiation. Upon irradiation the sample was cooled with compressed air and a fan to prevent thermal cycloreversion. The exact irradiation time was dependent on the concentrations of the solution. The irradiation time to reach above 95% conversion to the *Dewar* isomer in 0.05 M solutions was 5 minutes for ^{BN}**B3**-^{BN}**B7** and ^{BN}**B11**. The irradiation yielded the compounds ^{BN}**D3**-^{BN}**D7** and ^{BN}**D11** in almost quantitative yield.

HRMS data could not be obtained due to the thermal instability of the compound under the ionization conditions.

Previous studies employing an internal NMR standard have demonstrated that quantitative conversion to the photo isomer can be achieved for similar dihydroazaborinines.[14] However, solvent removal is not possible due to the insufficient stability of the Dewar isomer.[15] Consequently, no isolated yields are reported in the following. After irradiation under the conditions described above, no signals of the starting material are observed by NMR spectroscopy.

NMR data ^{BN}**D4**

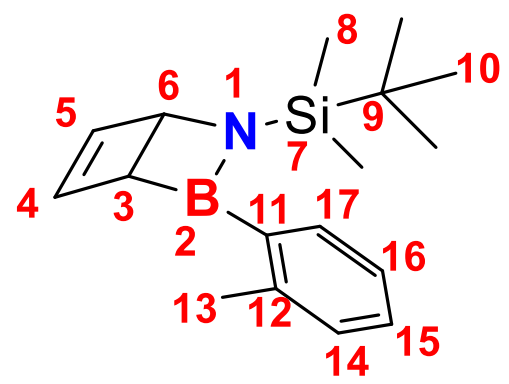

$C_{17}H_{26}BNSi$ (283.30 g/mol)

^{1}H NMR (600 MHz, **C_6D_{12}**): δ = 7.47 (d, $^{3}J_{HH}$ = 7.21 Hz, 1H, H-14), 7.22 (m, 2H, H-15), 7.22 (m, 1H, H-16), 7.12 (m, 1H, H-17), 6.67 (m, 1H, H-4), 6.46 (d, $^{3}J_{HH}$ = 2.27 Hz, 1H, H-5), 4.47 (ps. t, 1H, H-6), 3.12 (br. m, 1H, H-3), 2.46 (s, 3H, H-13), 1.02 (s, 9H, H-10), 0.17 (s, 3H, H-8), 0.10 (s, 3H, H-8) ppm.

^{13}C-{^{1}H} NMR (151 MHz, **C_6D_{12}**): δ = 143.4, 141.0, 140.2, 134.2, 131.4, 129.3, 124.6, 63.7, 43.3, 26.5, 22.1, 18.4, -5.0, -5.1 ppm.

^{11}B-{^{1}H} NMR (193 MHz, **C_6D_{12}**): δ = 51.7 ppm.

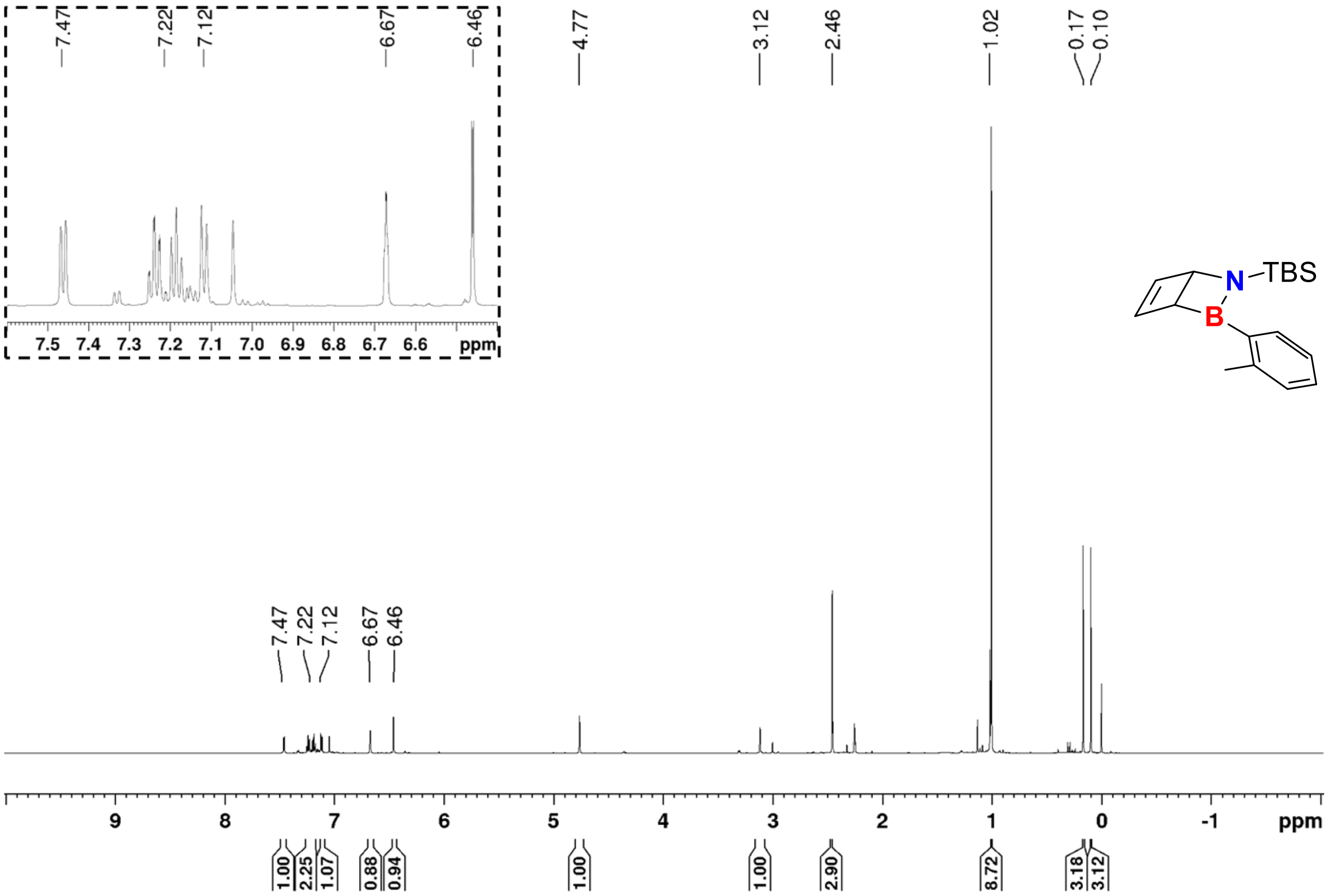

Figure S75. ^{1}H NMR spectrum von ^{BN}**D4** in C_6D_{12} measured on a 600 MHz spectrometer.

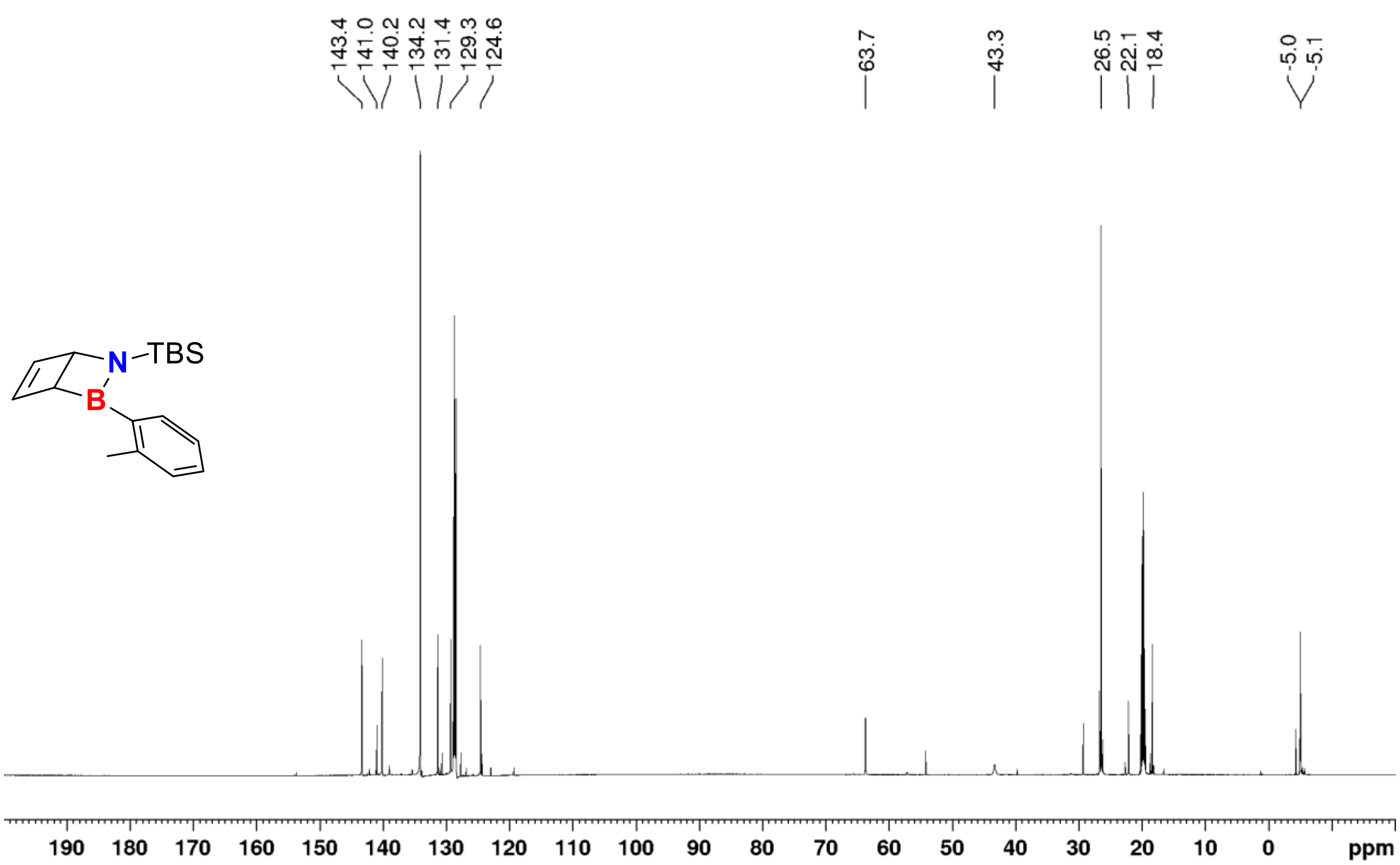

Figure S76. ^{13}C-{^{1}H} NMR spectrum von ^{BN}**D4** in C_6D_{12} measured on a 600 MHz spectrometer.

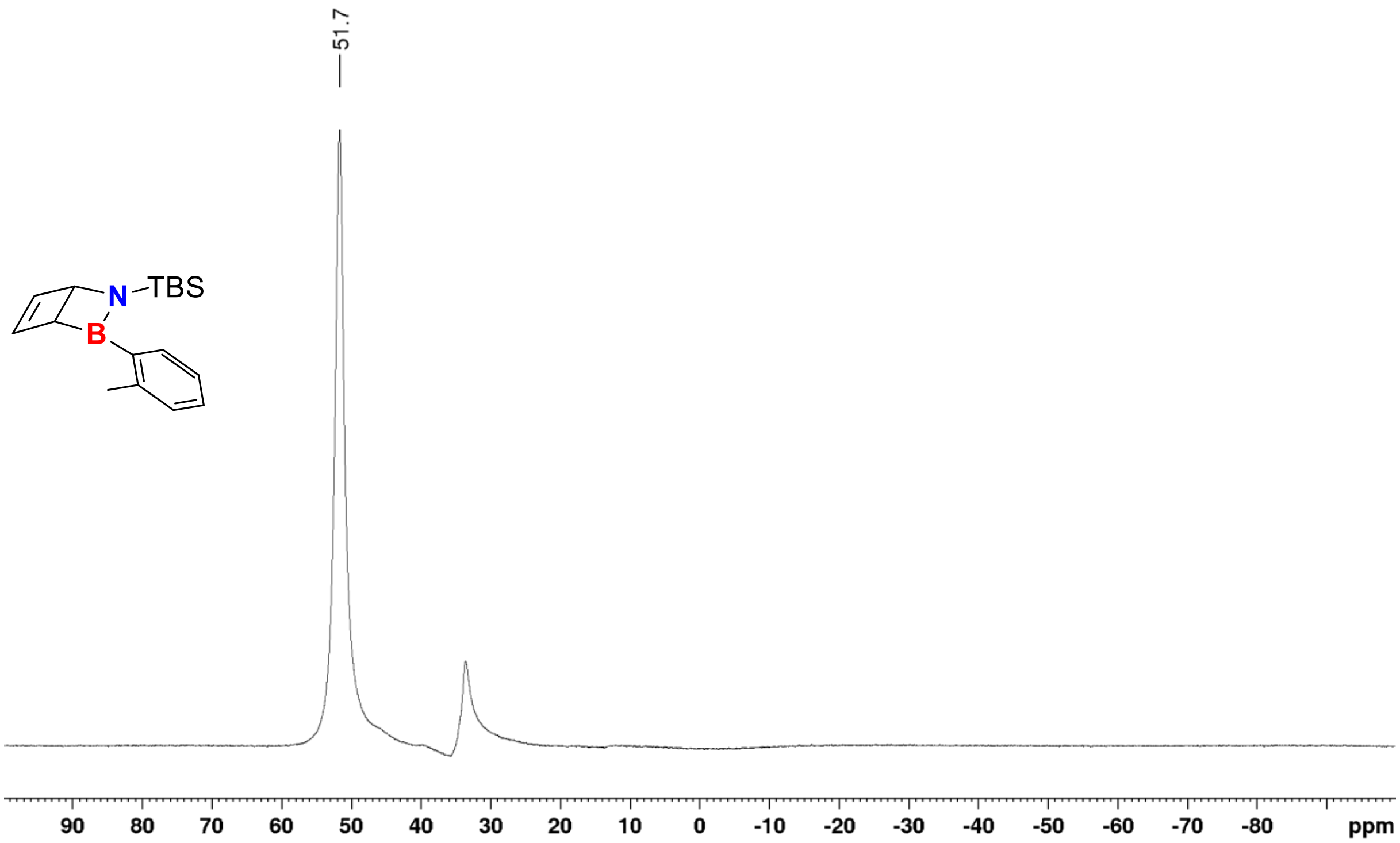

Figure S77. ^{11}B-{^{1}H} NMR spectrum von ***BN*D4** in C_6D_{12} measured on a 600 MHz spectrometer.

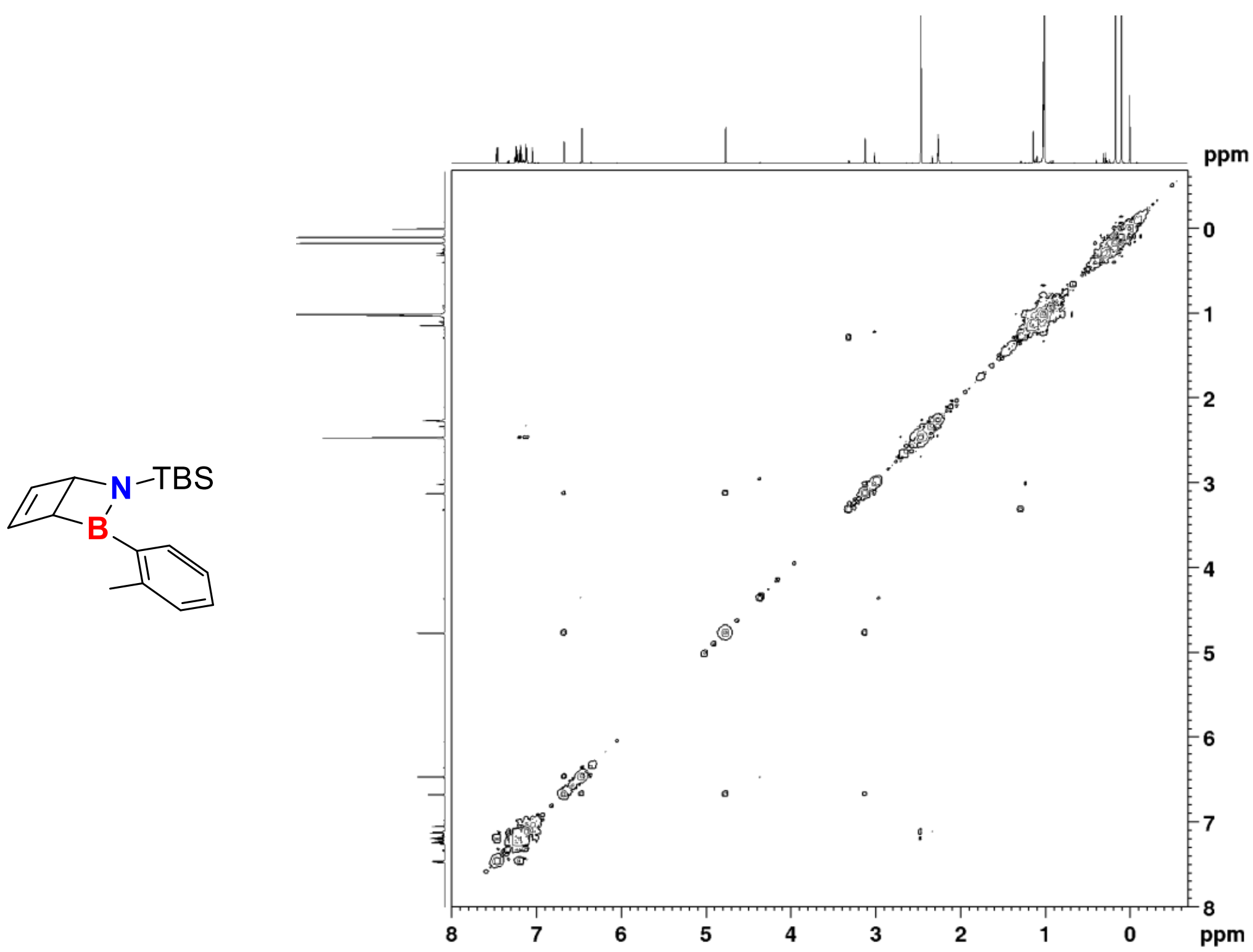

Figure S78. ^{1}H-^{1}H-COSY NMR spectrum von ***BN*D4** in C_6D_{12} measured on a 600 MHz spectrometer.

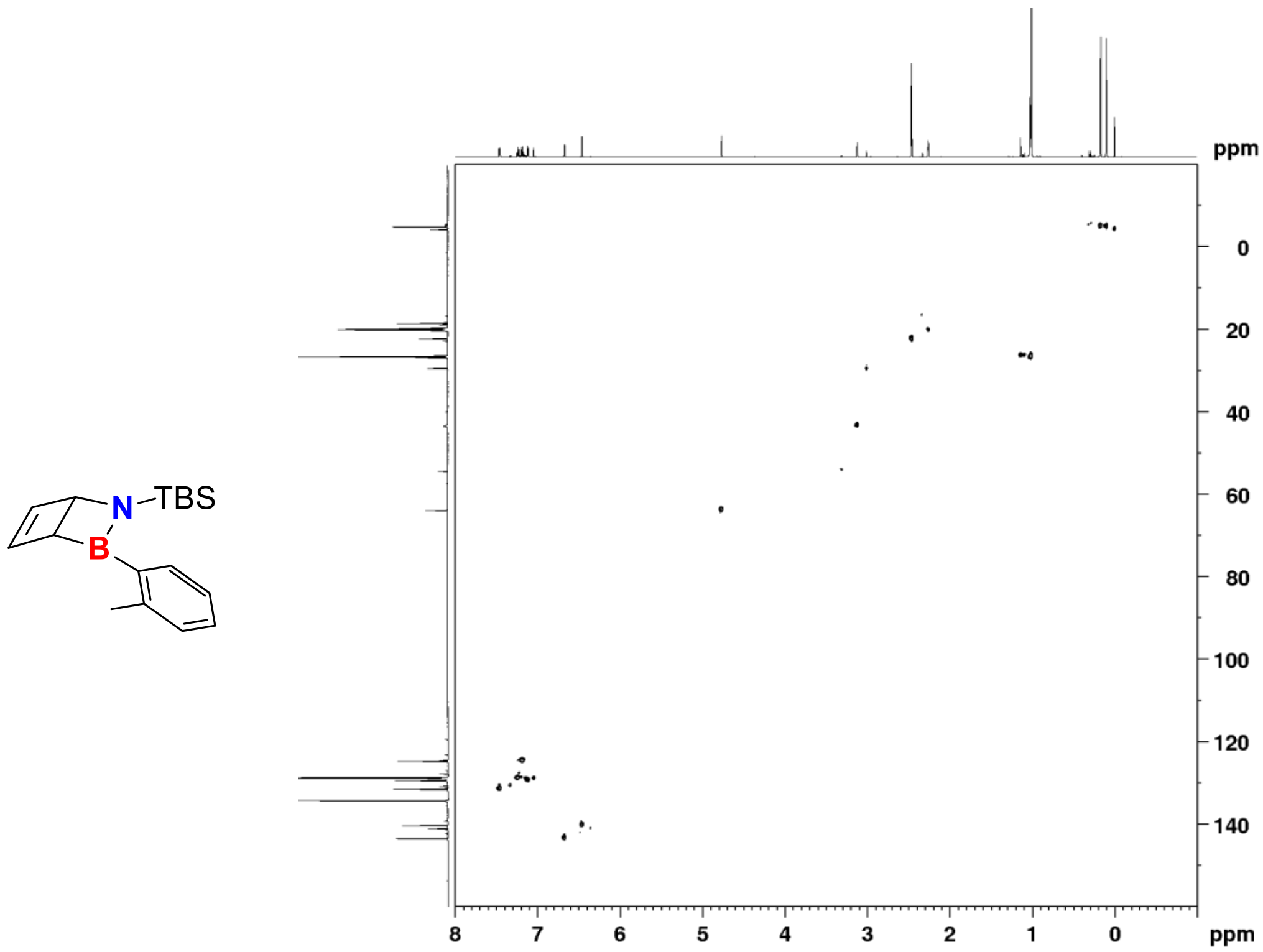

Figure S79. ^{1}H-^{13}C-HSQC NMR spectrum von ^{BN}**D4** in C_6D_{12} measured on a 600 MHz spectrometer.

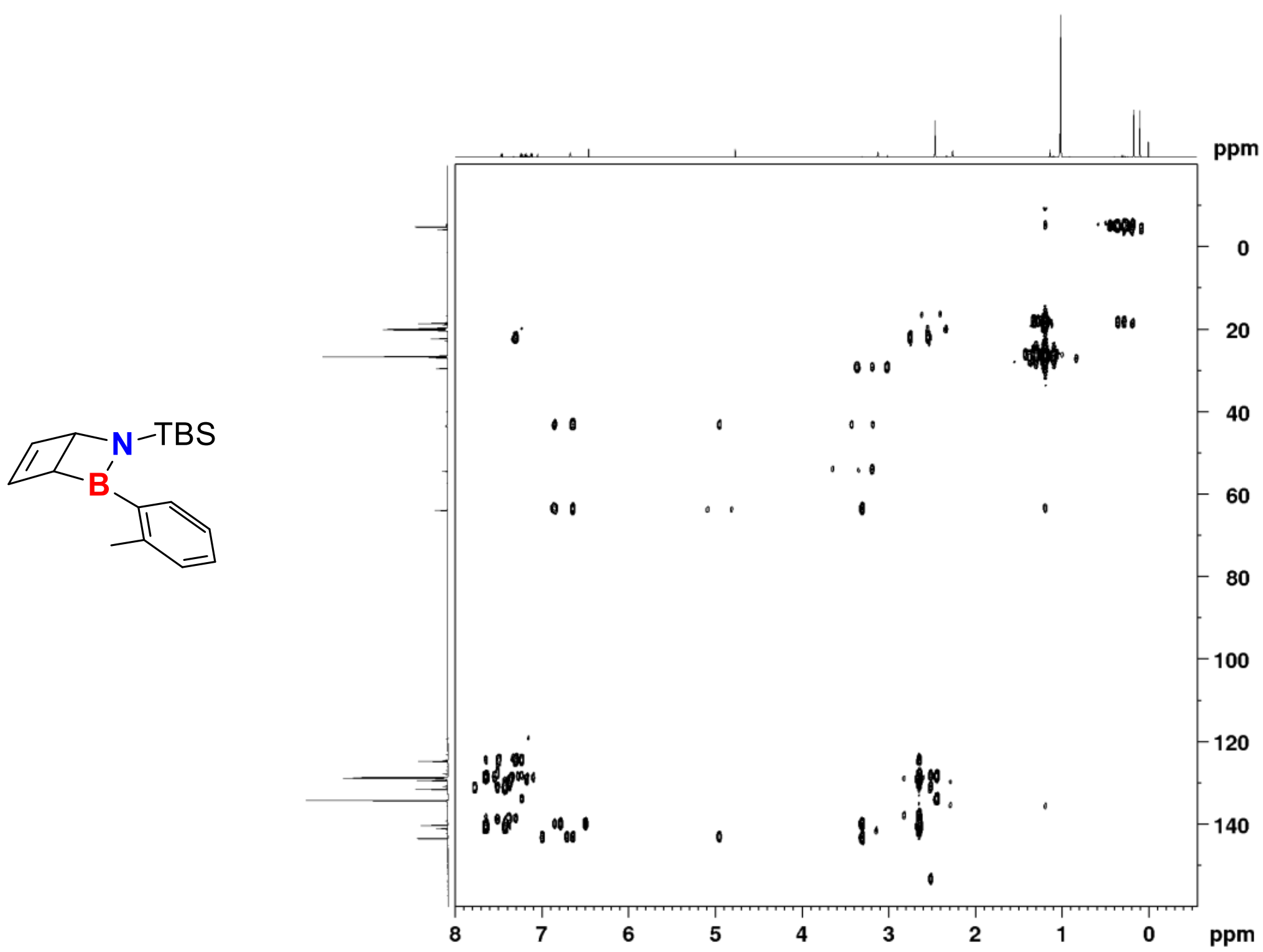

Figure S80. ^{1}H-^{13}C-HMBC NMR spectrum von ^{BN}**D4** in C_6D_{12} measured on a 600 MHz spectrometer.

NMR data of ^{BN}**D3**

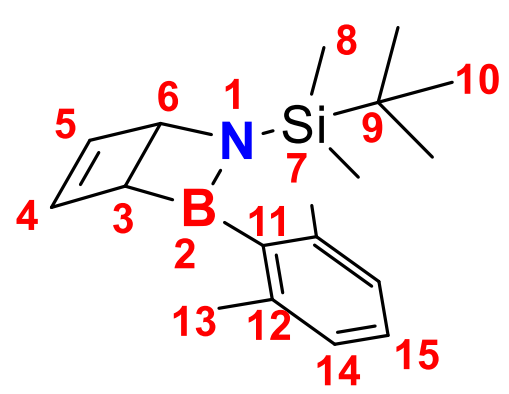

$C_{18}H_{28}BNSi$ (297.32 g/mol)

^{1}H NMR (600 MHz, **C_6D_{12}**): δ = 7.16 (ps. t, 1H, H-15), d, 2H, H-14), 6.61 (br. m, 1H, H-4), 6.44 (d, $^{3}J_{HH}$ = 2.37 Hz, 1H, H-5), 4.80 ps.t, 1H, H-6), 3.15 (br. M, 1H, H-3), 2.37 (s, 6H, H-13), 1.01 (s, 9H, H-10), 0.04 (s, 3H, H-8), 0.00 (s, 3H, H-8) ppm.

^{13}C-{^{1}H} NMR (151 MHz, **C_6D_{12}**): δ = 144.1, 143.6, 143.6, 142.9, 138.9, 137.9, 126.1, 64.1, 44.2, 26.1, 22.0, 18.2, -6.2 ppm.

^{11}B-{^{1}H} NMR (193 MHz, **C_6D_{12}**): δ = 52.8 ppm.

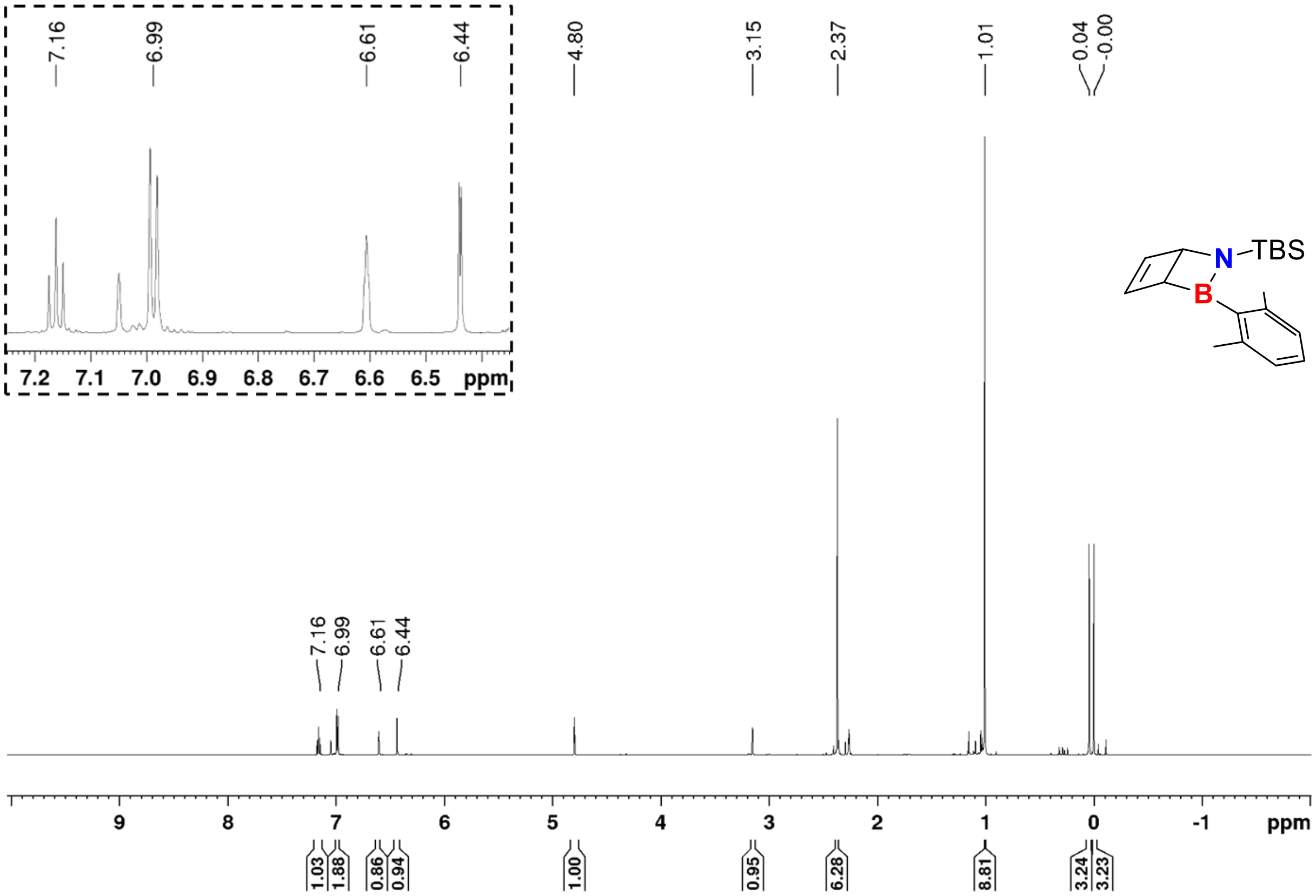

Figure S81. ^{1}H NMR spectrum von ^{BN}**D3** in C_6D_{12} measured on a 600 MHz Spectrometer.

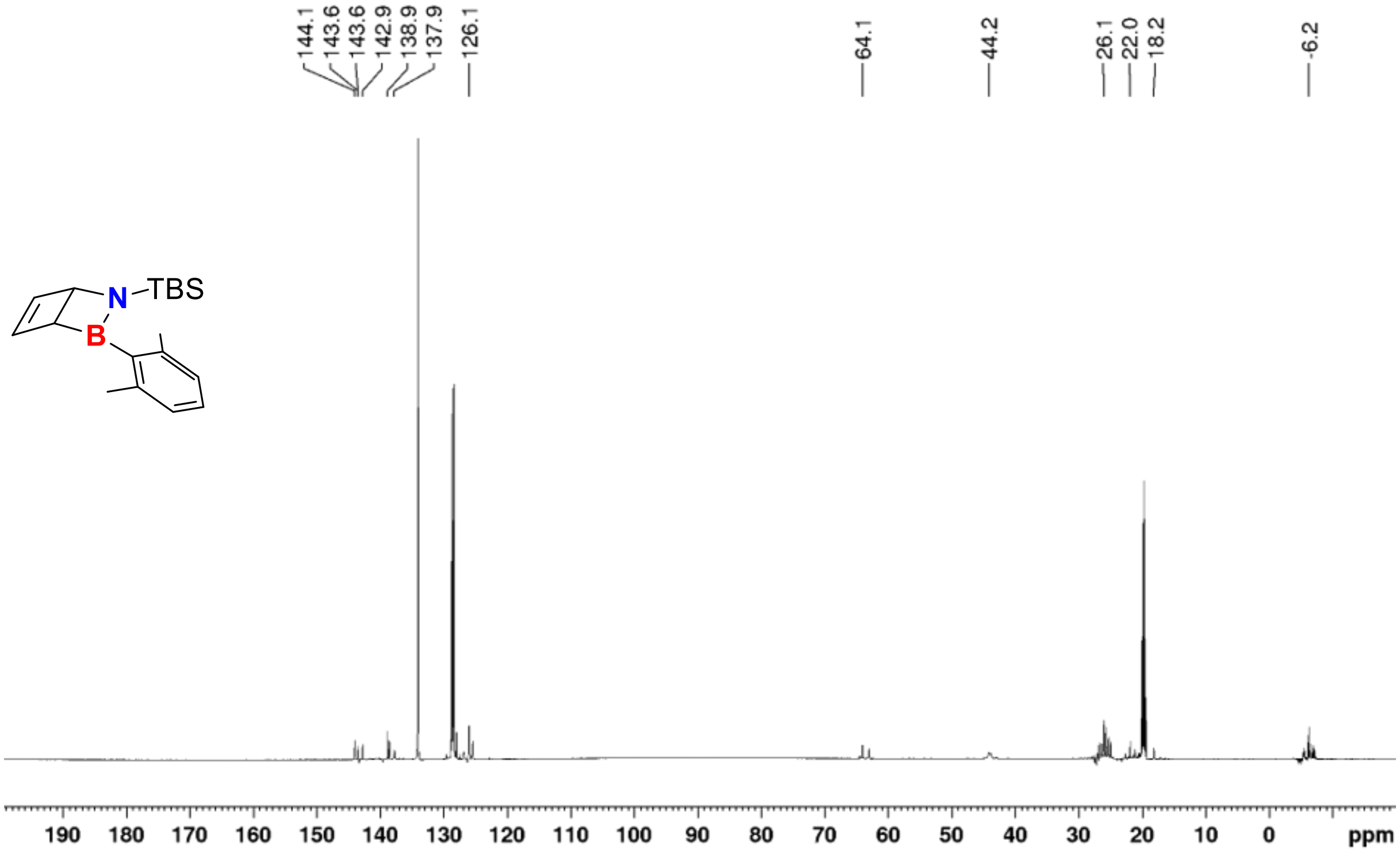

Figure S82. ^{13}C-{^{1}H} NMR spectrum von ^{BN}**D3** in C_6D_{12} measured on a 600 MHz Spectrometer.

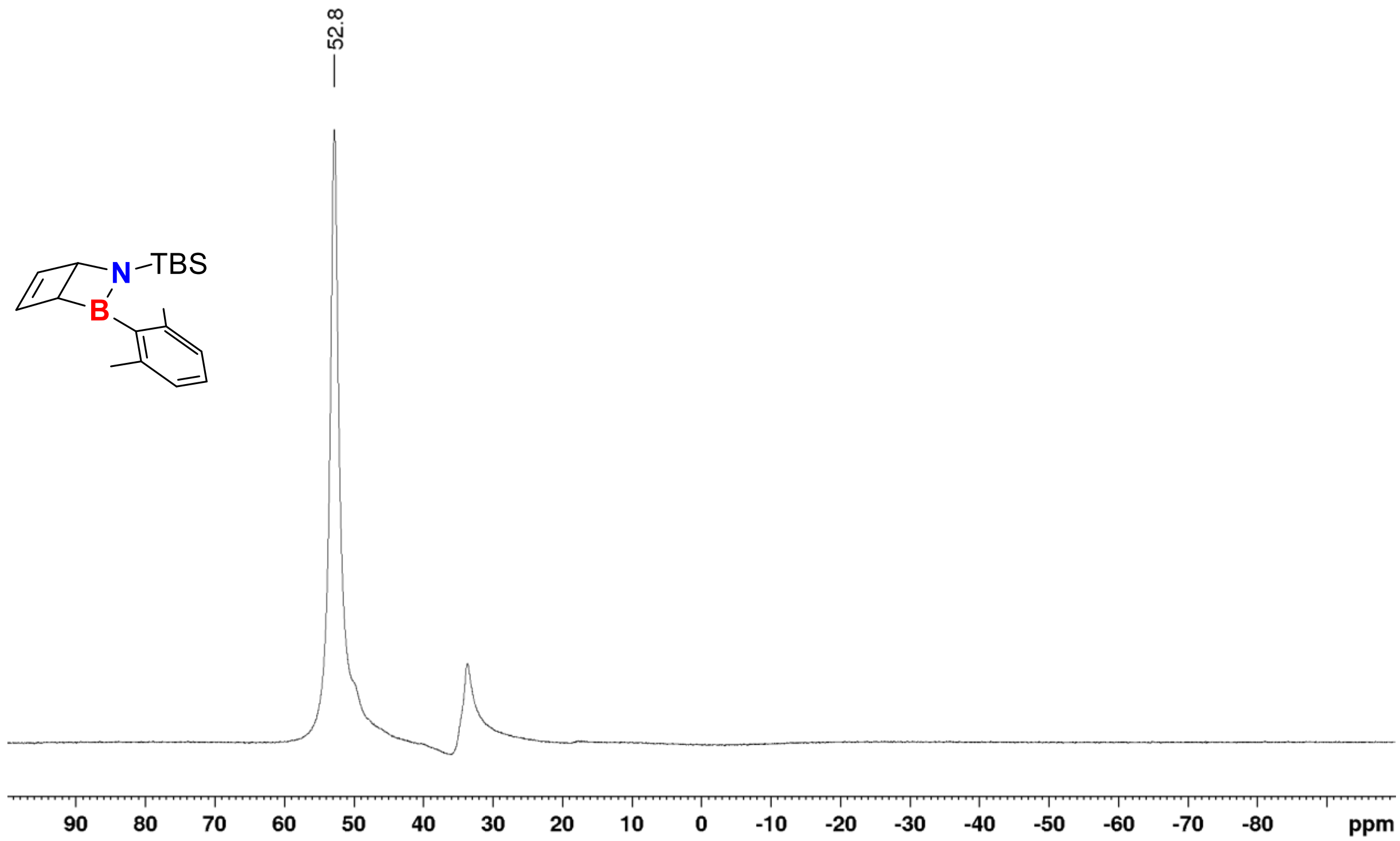

Figure S83. ^{11}B-{^{1}H} NMR spectrum von ^{BN}**D3** in C_6D_{12} measured on a 600 MHz Spectrometer.

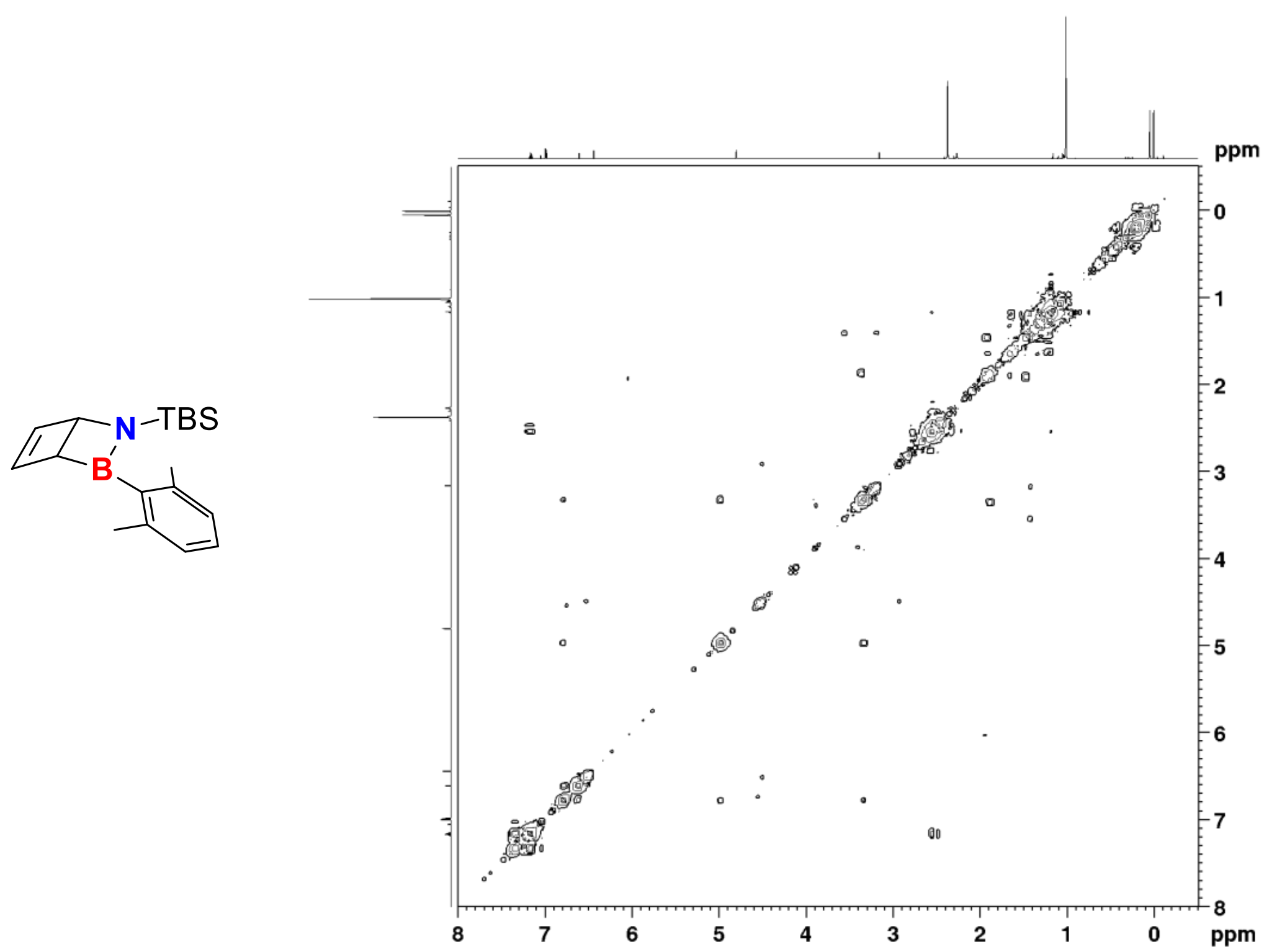

Figure S84. ^{1}H-^{1}H-COSY NMR spectrum von ^{BN}**D3** in C_6D_{12} measured on a 600 MHz Spectrometer.

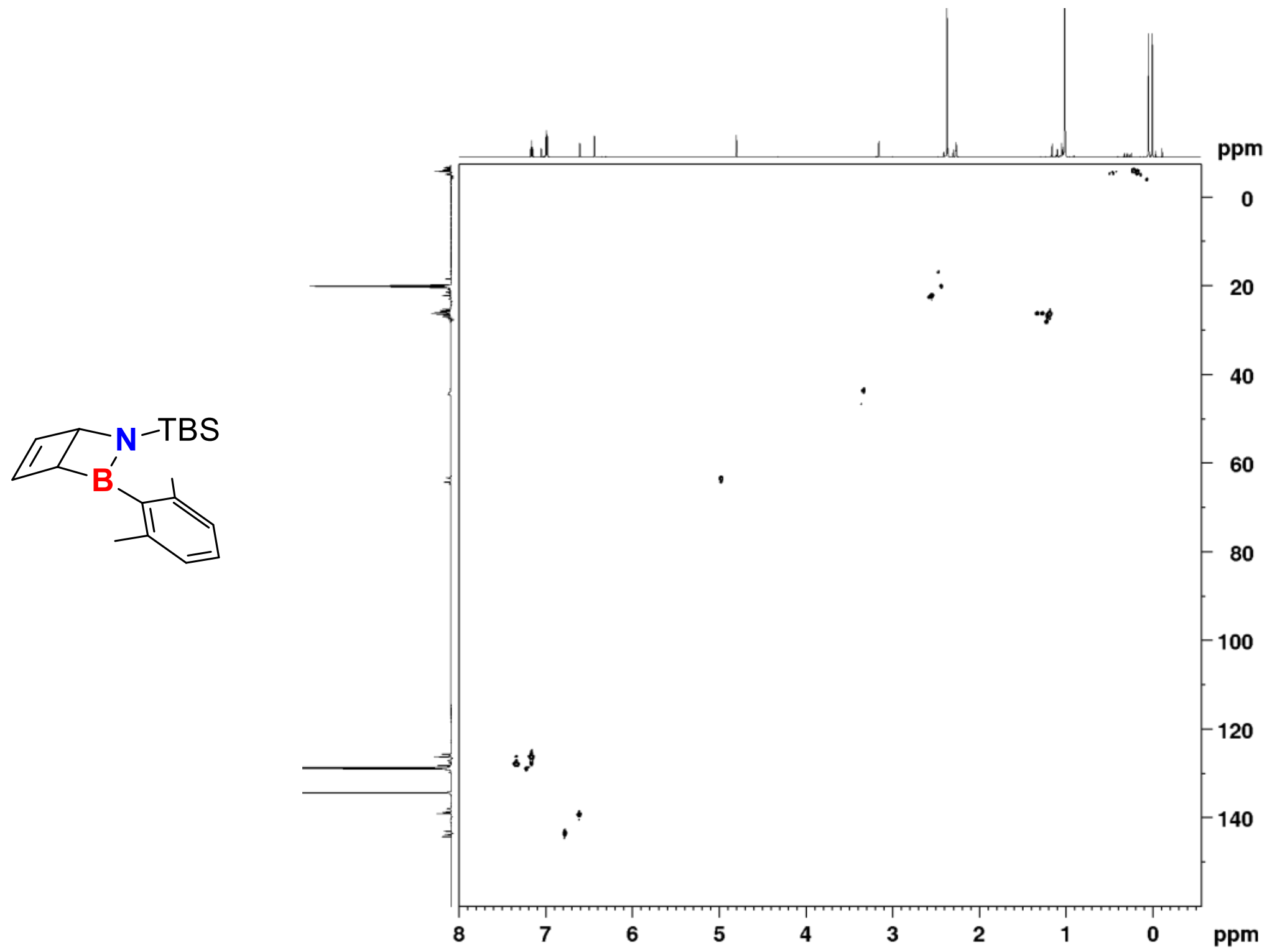

Figure S85. ^{1}H-^{13}C-HSQC NMR spectrum von ^{BN}**D3** in C_6D_{12} measured on a 600 MHz Spectrometer.

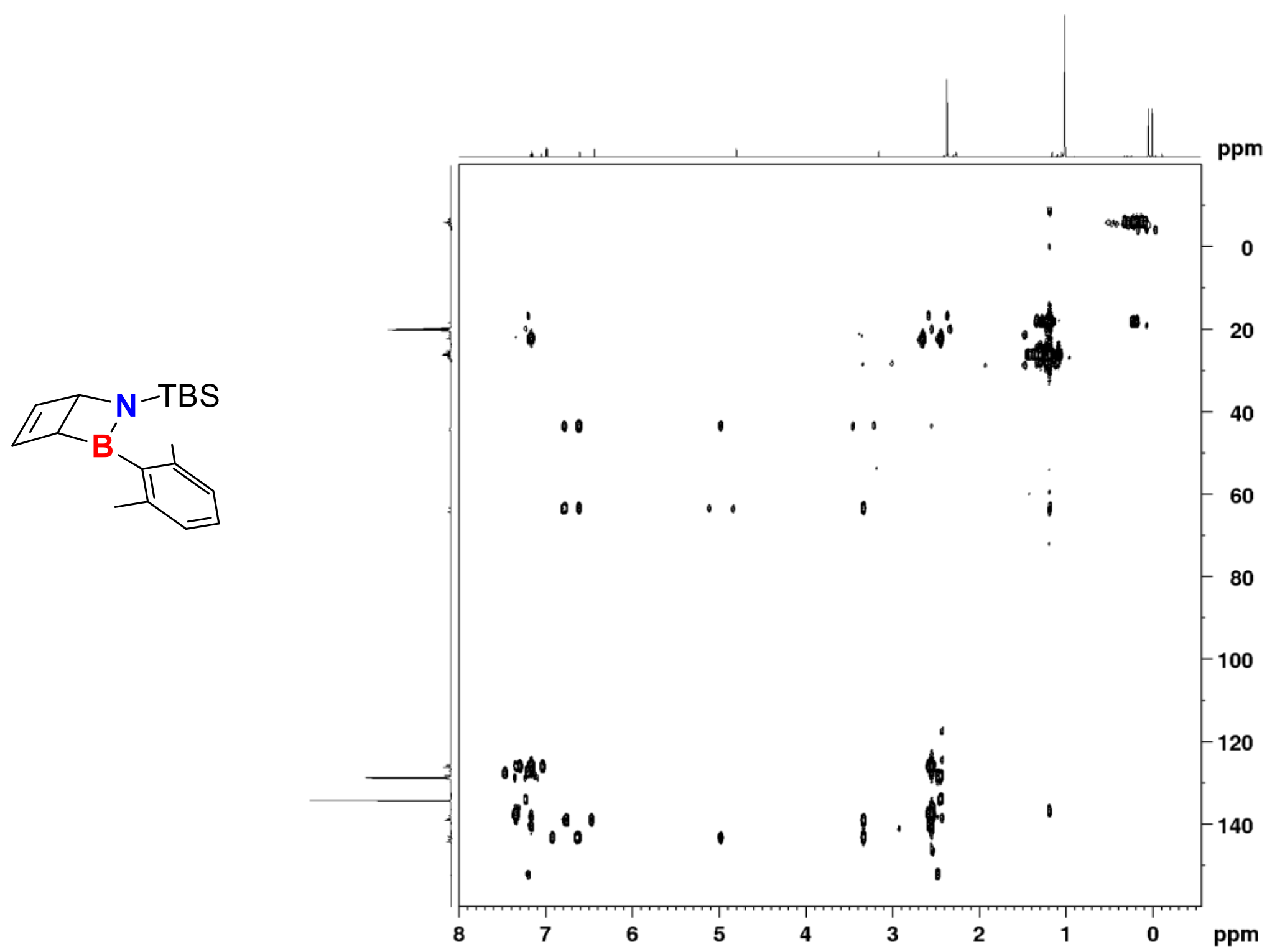

Figure S86. ^{1}H-^{13}C-HMBC NMR spectrum von ^{BN}**D3** in C_6D_{12} measured on a 600 MHz Spectrometer.

NMR data of ^{BN}**D5**

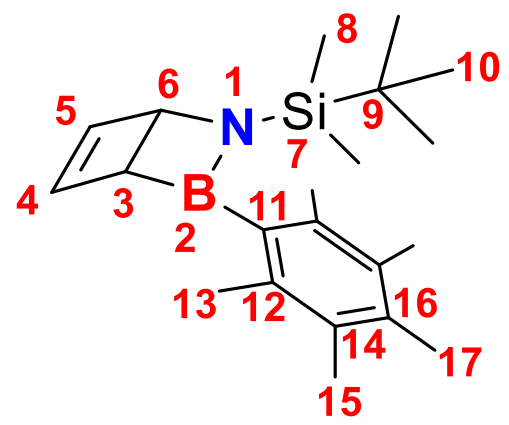

$C_{21}H_{34}BNSi$ (339.41 g/mol)

^{1}H NMR (600 MHz, **C_6D_{12}**): δ = 6.67 (m, 1H, H-4), 6.43 (d, $^3J_{HH}$ = 2.34 Hz, 1H, H-5), 4.82 (ps.t, 1H, H-6), 3.189 (m, 1H, H-3), 2.32 (br. s, 6H, H-13), 2.19 (s, 9H, H-15/H-17), 0.99 (s,)h; H-10), 0.05 (s, 3H, H-8), 0.00 (s, 3H, H-8) ppm.

^{13}C-{^{1}H} NMR (151 MHz, **C_6D_{12}**): δ = 143.7, 139., 138.6, 134.1, 63.4, 44.3, 26.3, 21.4, 20.9, 18.3, 16.3, 15.6, -5.5, -5.9 ppm.

^{11}B-{^{1}H} NMR (193 MHz, **C_6D_{12}**): δ = 53.0 ppm.

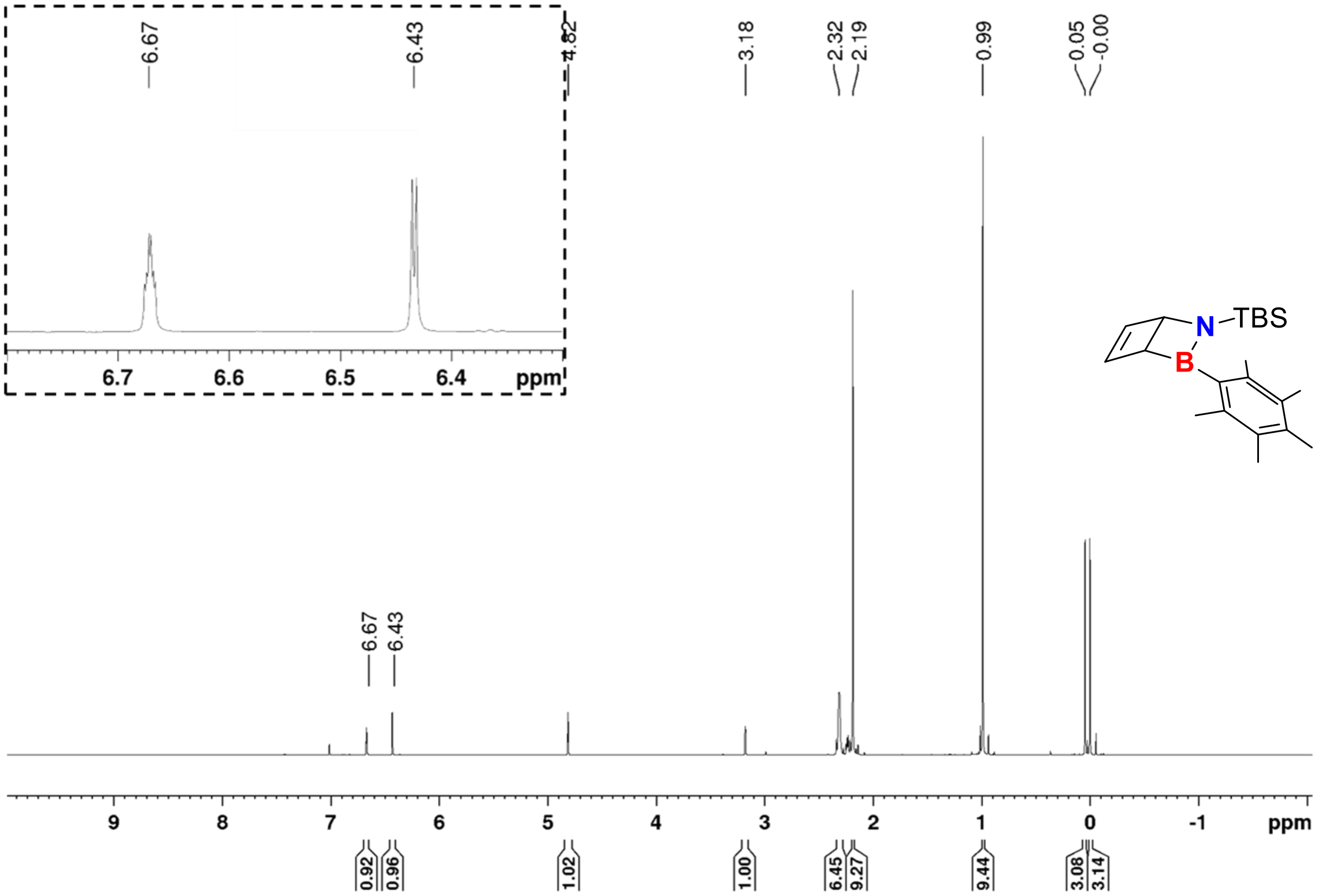

Figure S87. ^{1}H NMR spectrum von ***BN*D5** in C_6D_{12} measured on a 600 MHz Spectrometer.

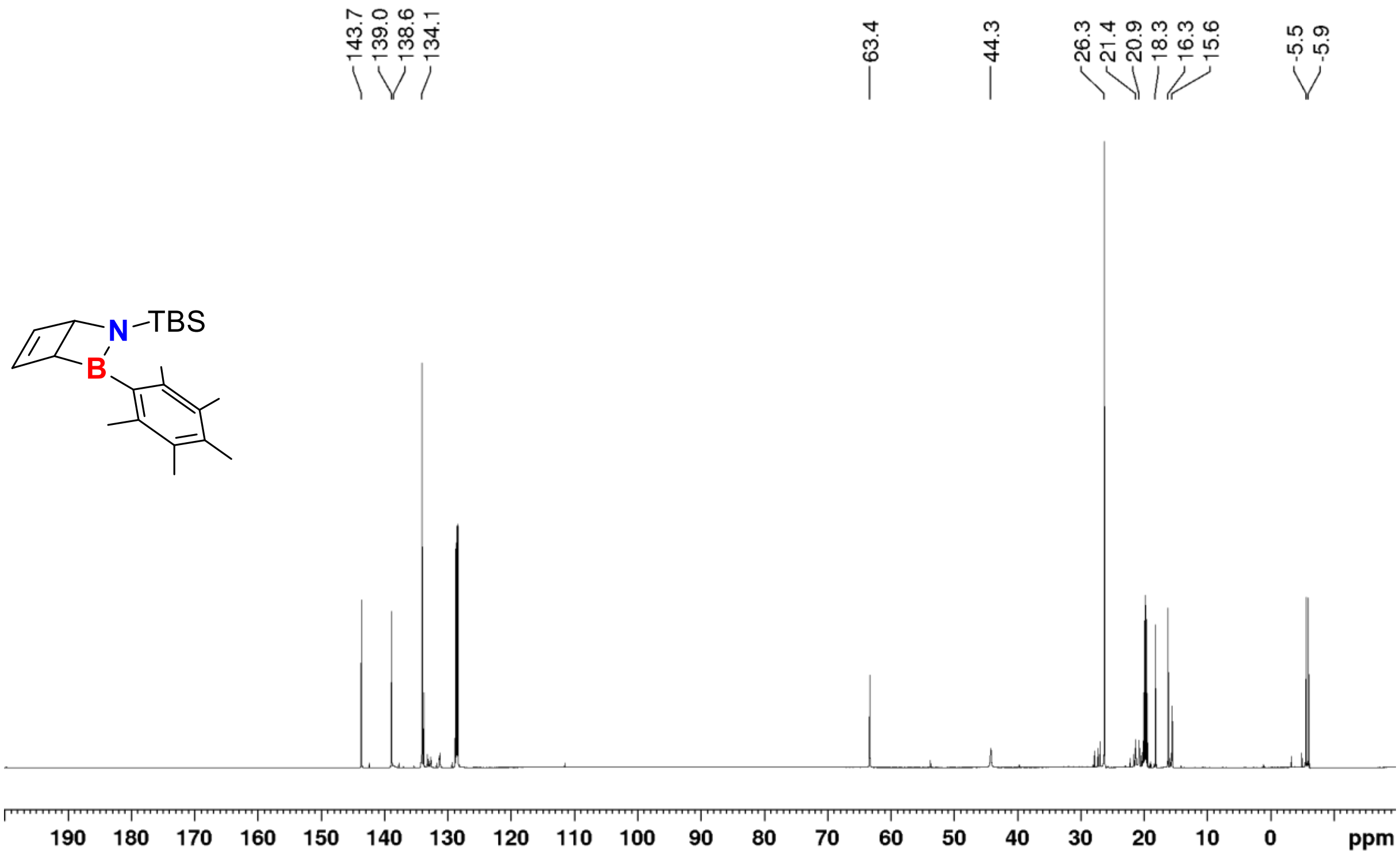

Figure S88. ^{13}C-{^{1}H} NMR spectrum von ***BN*D5** in C_6D_{12} measured on a 600 MHz Spectrometer.

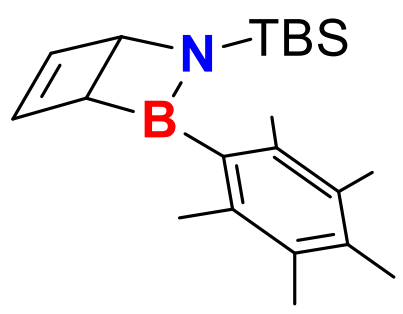

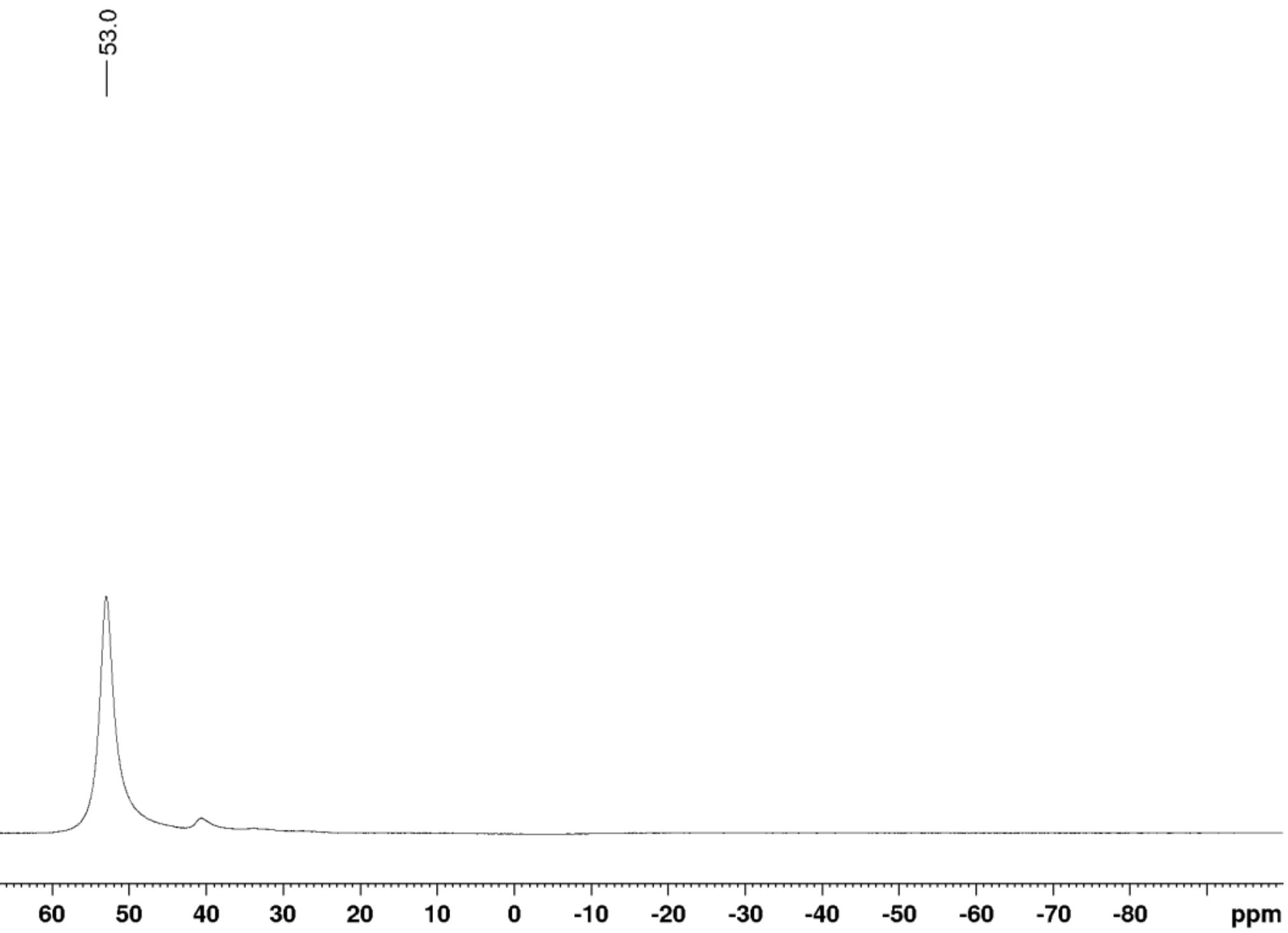

Figure S89. ^{11}B-{^{1}H} NMR spectrum von ^{BN}**D5** in C_6D_{12} measured on a 600 MHz Spectrometer.

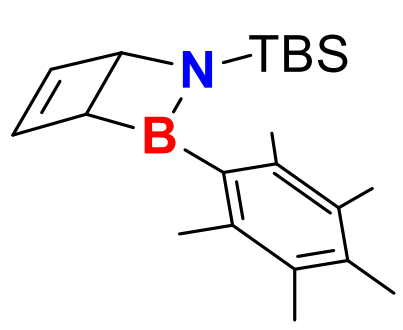

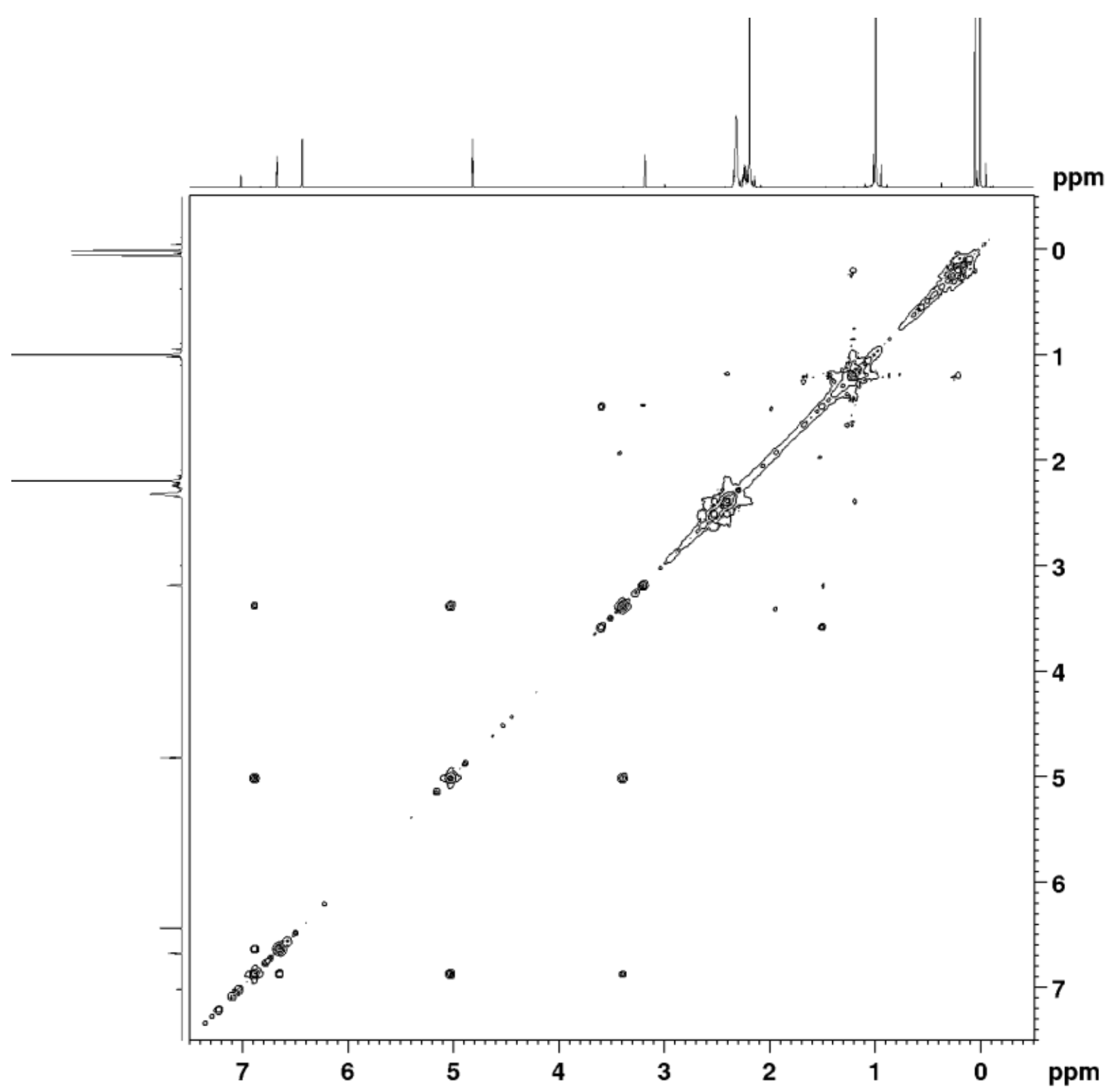

Figure S90. ^{1}H-^{1}H-COSY NMR spectrum von ^{BN}**D5** in C_6D_{12} measured on a 600 MHz Spectrometer.

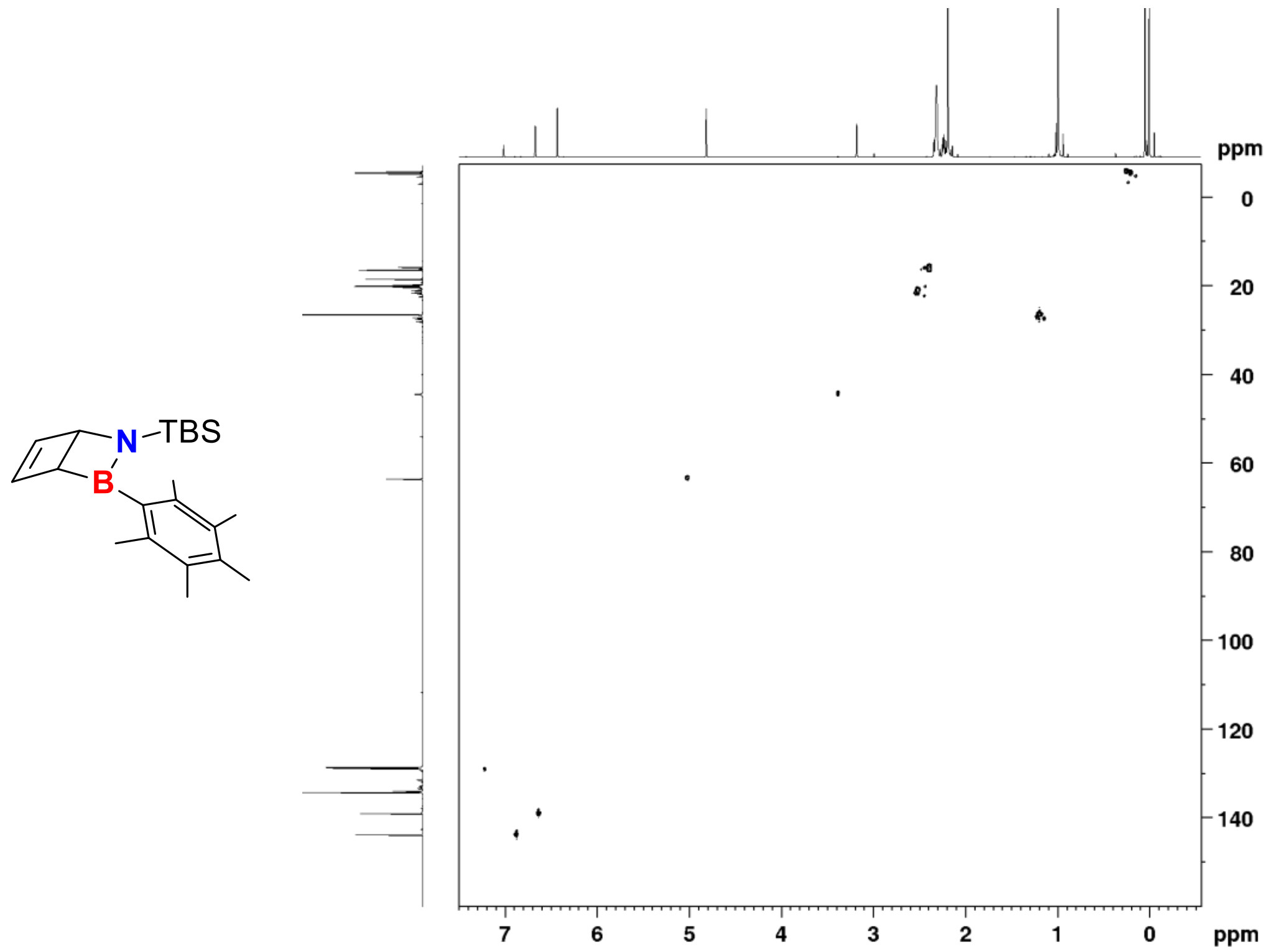

Figure S91. ^{1}H-^{13}C-HSQC NMR spectrum von ^{BN}**D5** in C_6D_{12} measured on a 600 MHz Spectrometer.

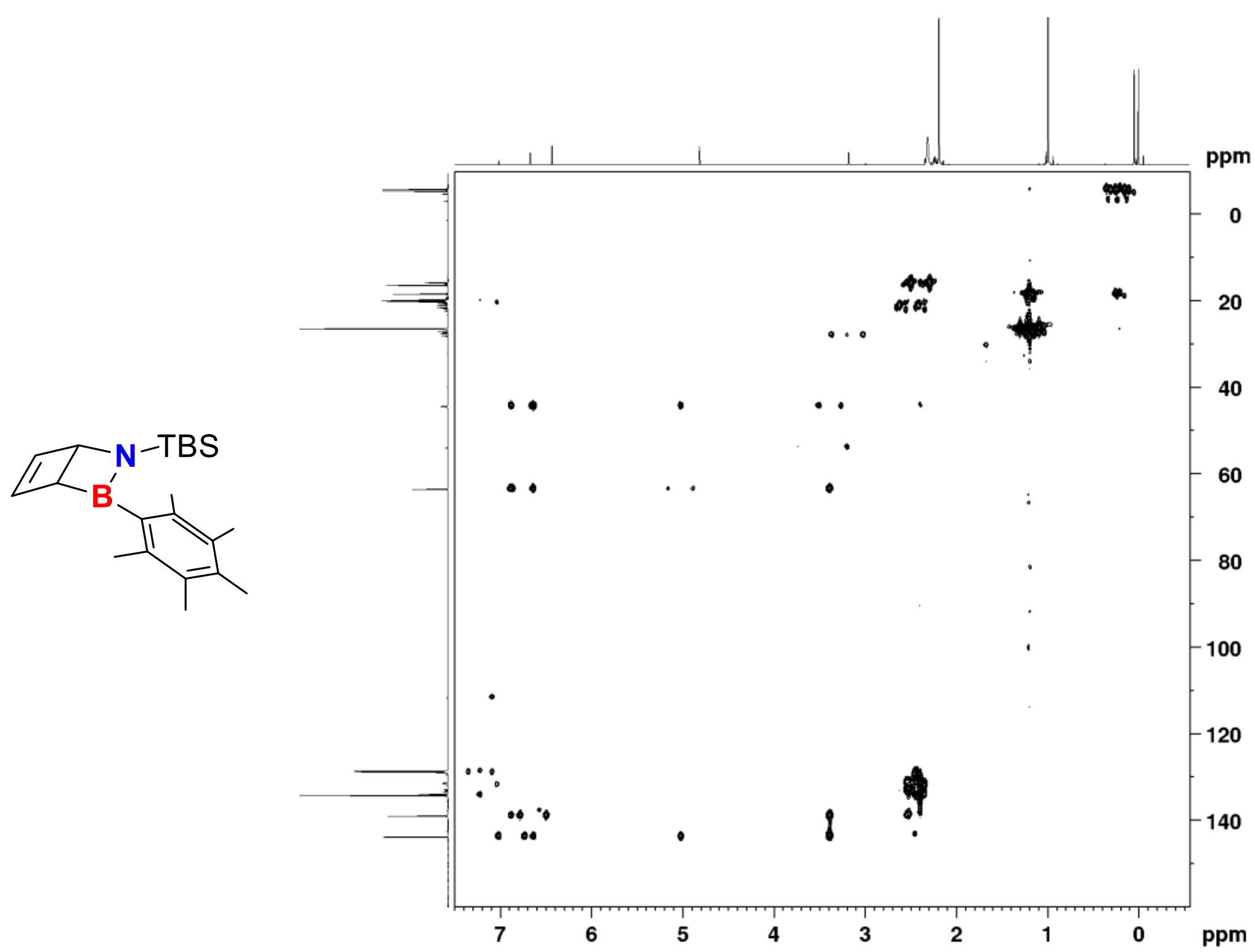

Figure S92. ^{1}H-^{13}C-HMBC NMR spectrum von ^{BN}**D5** in C_6D_{12} measured on a 600 MHz Spectrometer.

NMR data of ^{BN}**D6**

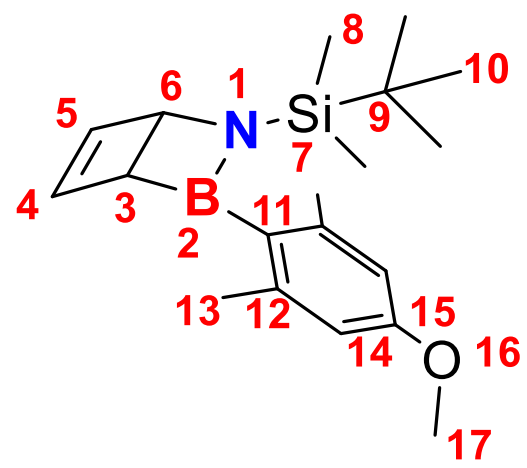

$C_{19}H_{30}BNOSi$ (327.22 g/mol)

^{1}H NMR (400 MHz, toluene-d_8) δ = 6.54 (s, 2H, *H*-13), 6.49 (ps. td, *J* = 2.3, 1.1 Hz, 1H, *H*-4), 6.32 (d, *J* = 2.3 Hz, 1H, *H*-5), 4.68 (t, *J* = 2.3 Hz, 1H, *H*-6), 3.39 (s, 3H, *H*-17), 3.05 (dd, *J* = 2.1, 1.1 Hz, 1H, *H*-3), 2.23 (s, 6H, *H*-13), 0.88 (s, 9H, *H*-10), 0.06 (s, 3H, *H*-8), 0.09 (s, 3H, *H*-8).

^{13}C-{^{1}H}- NMR (101 MHz, toluene-d_8) δ = 160.2, 143.7, 139.8, 139.5, 137.2, 112.3, 63.5, 54.3, 26.5, 22.8, 18.4, -5.4, -5.7.

^{11}B-{^{1}H} NMR (128 MHz, toluene-d_8) δ = 52.9.

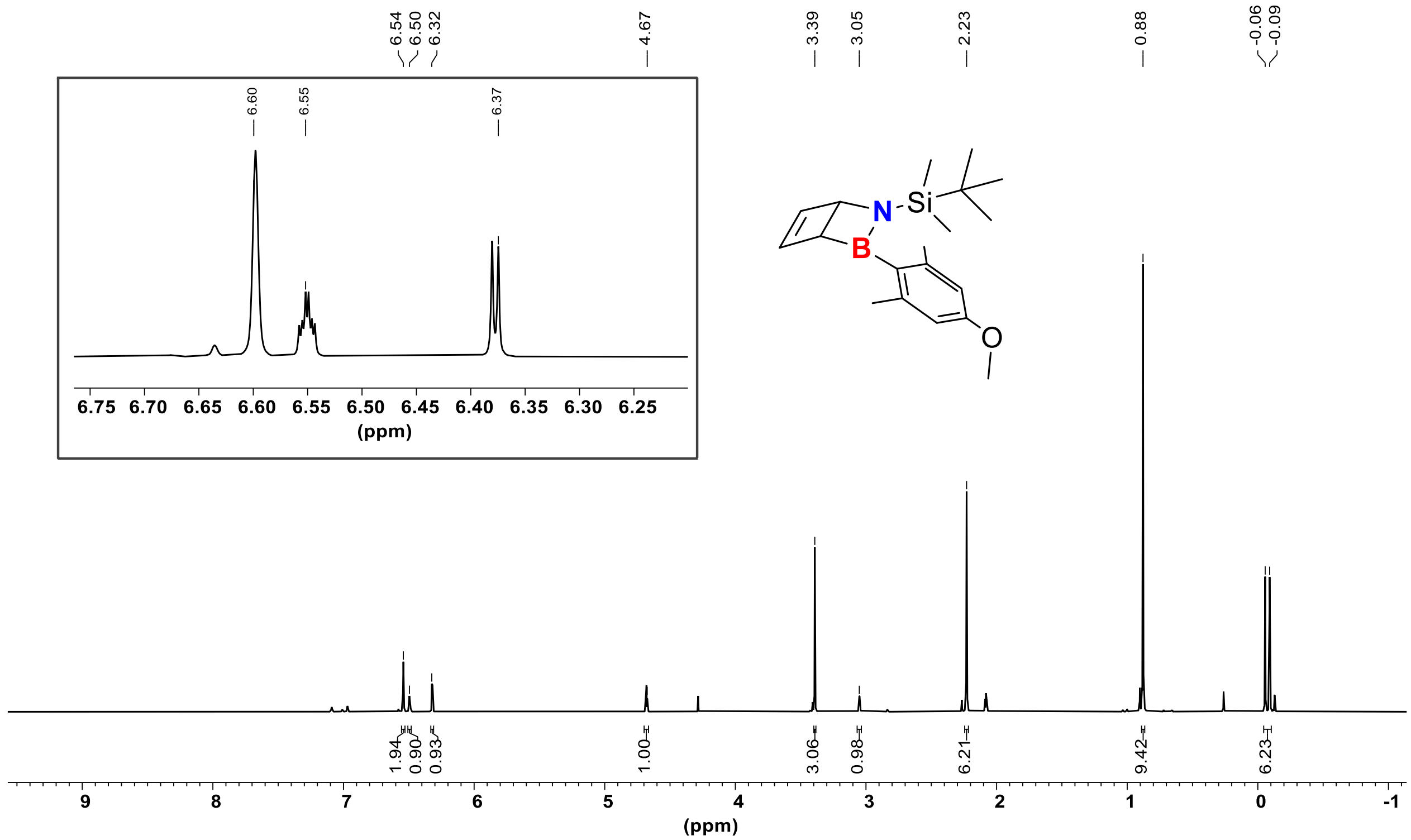

Figure S93: ^{1}H NMR spectrum of ***BN*D6** in toluene-d$_8$ measured on a 400 MHz spectrometer.

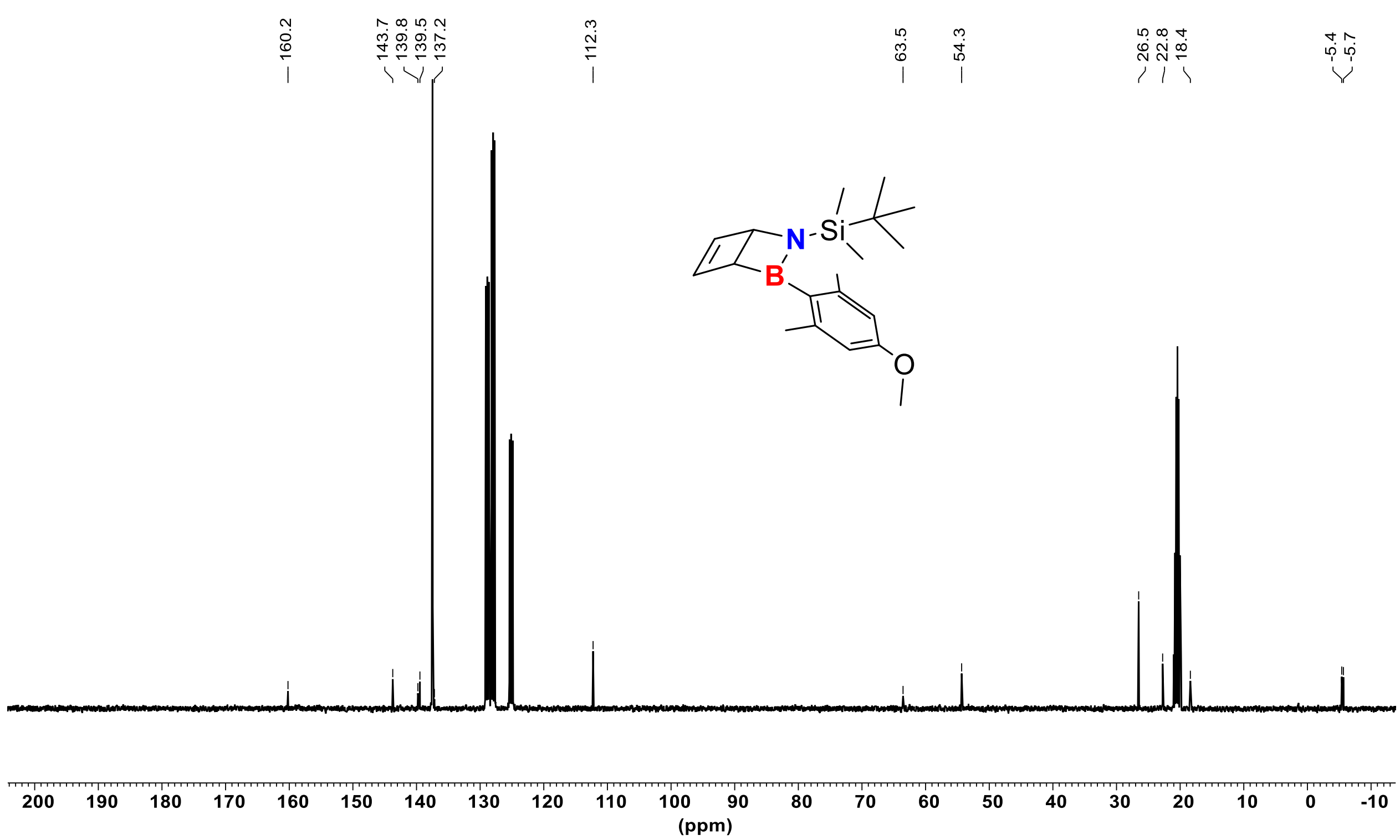

Figure S94: ^{13}C-{^{1}H} NMR spectrum of ***BN*D6** in toluene-d$_8$ measured on a 400 MHz spectrometer.

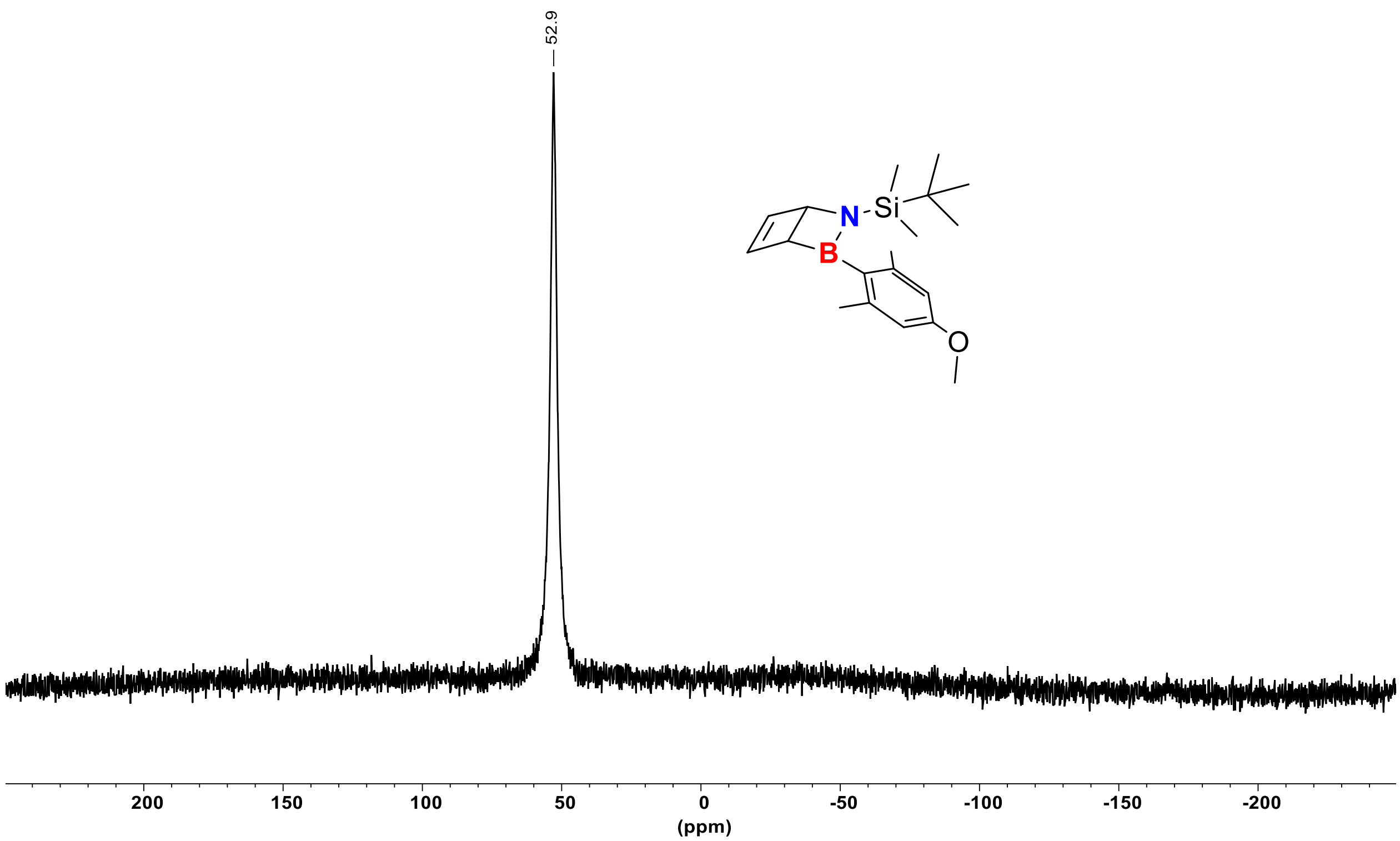

Figure S95: ^{1}B-{^{1}H} NMR spectrum of ***BN*D6** in toluene-d$_8$ measured on a 400 MHz spectrometer.

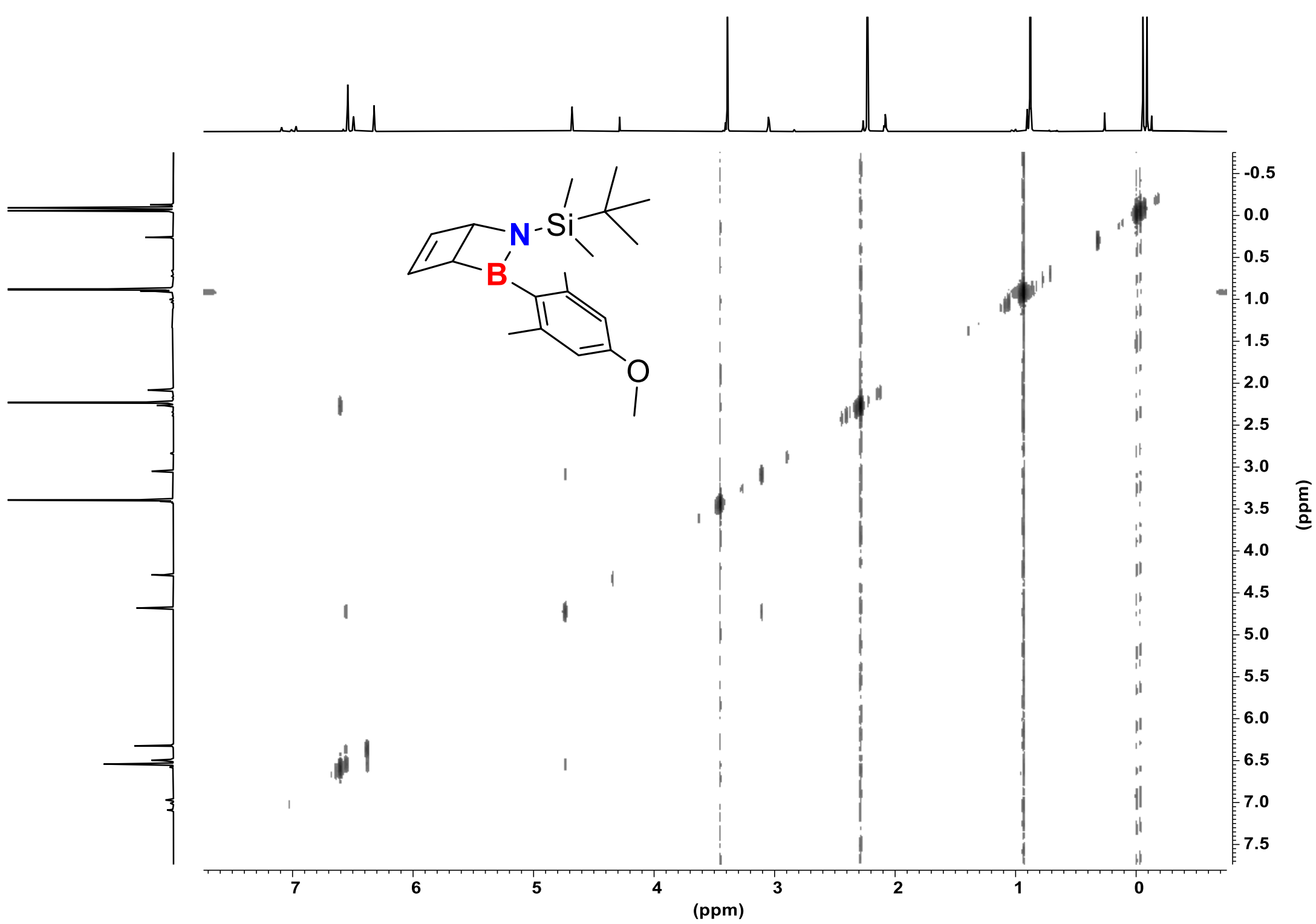

Figure S96: ^{1}H-^{1}H-COSY NMR spectrum of ***BN*D6** in toluene-d$_8$ measured on a 400 MHz spectrometer.

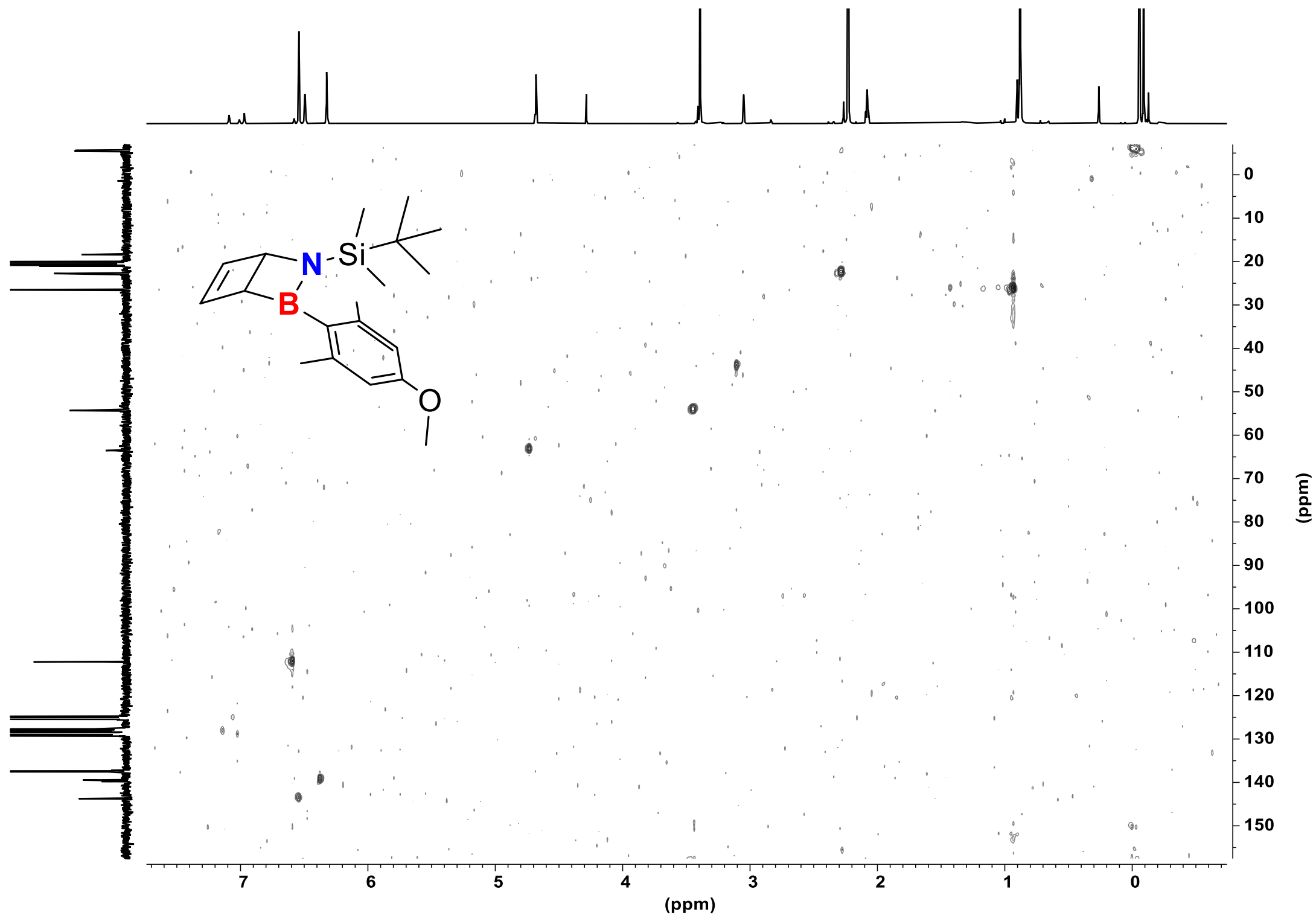

Figure S97: ^{1}H-^{13}C-HSQC NMR spectrum of ***BN*D6** in toluene-d_8 measured on a 400 MHz spectrometer.

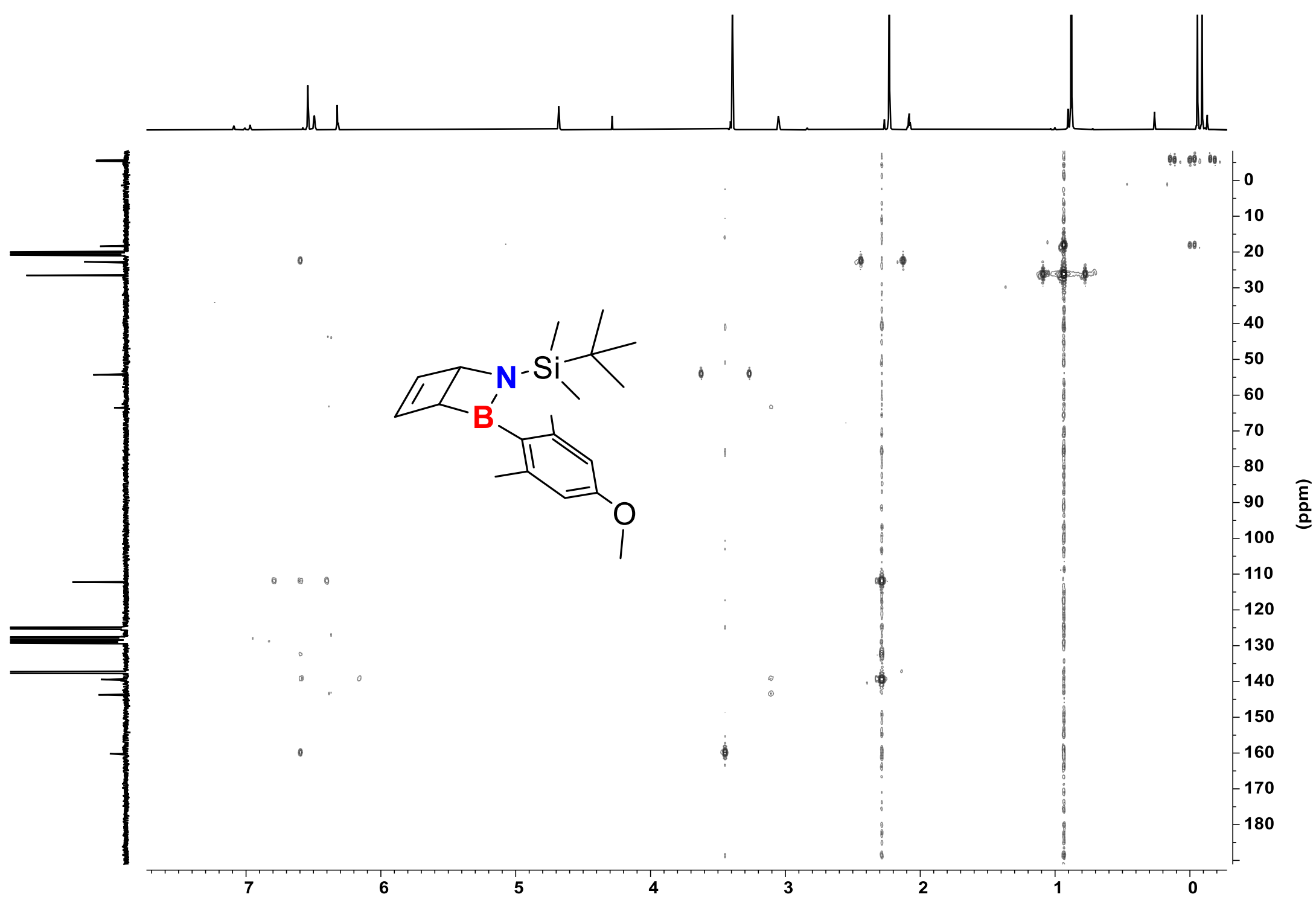

Figure S98: ^{1}H-^{13}C-HMBC NMR spectrum of ***BN*D6** in toluene-d_8 measured on a 400 MHz spectrometer.

NMR data of ^{BN}**D7**

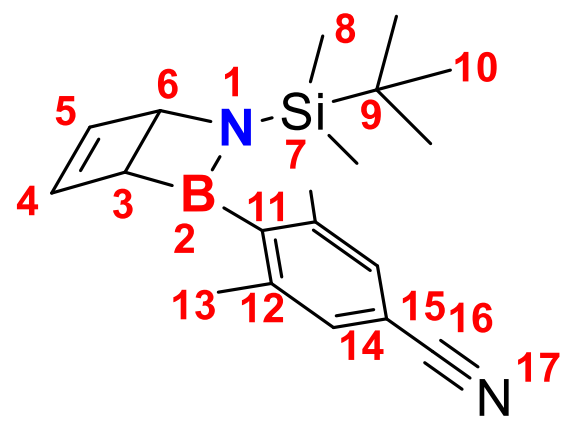

$C_{19}H_{27}BN_2Si$ (327.22 g/mol)

^{1}H NMR (400 MHz, toluene-d$_8$) δ 6.76 (s, 2H, H-14), 6.35 (ps. td, *J* = 2.3, 1.1 Hz, 1H, *H*-4), 6.23 (d, *J* = 2.2 Hz, 1H, *H*-5), 4.59 (ps. t, *J* = 2.3 Hz, 1H, *H*-6), 1.94 (s, 6H, *H*-13), 0.80 (s, 9H, *H*-10), -0.20 (s, 3H, *H*-8), -0.26 (s, 3H, *H*-8).

^{13}C-{^{1}H} NMR (101 MHz, toluene-d$_8$) δ 143.0, 139.3, 136.8, 129.0, 118.9, 111.8, 63.6, 26.7, 26.0, 21.5, 17.9, -5.9, -6.3.

^{11}B-{^{1}H} NMR (128 MHz, toluene-d$_8$) δ 51.5.

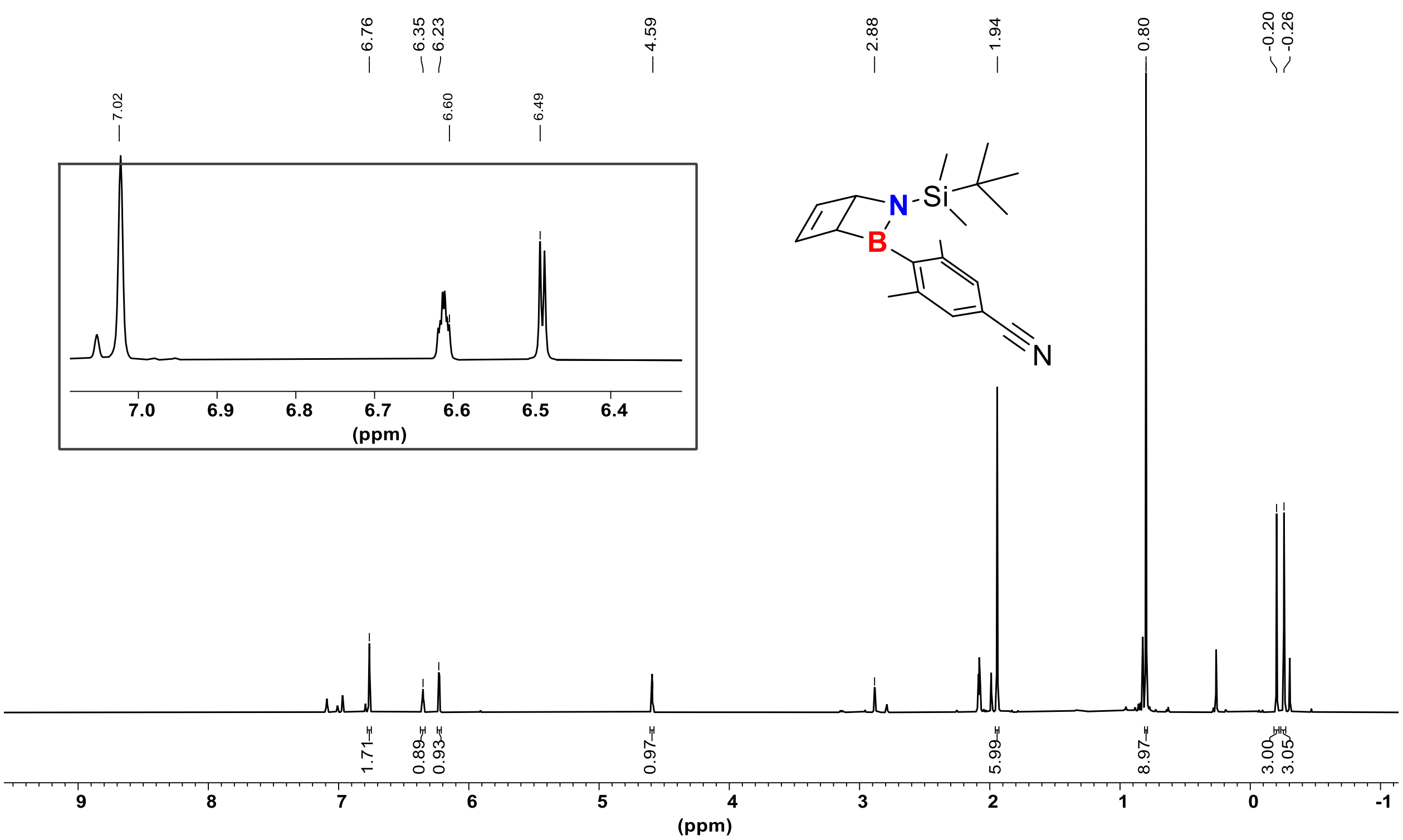

Figure S99: ^{1}H NMR spectrum of ^{BN}**D7** in toluene-d$_8$ measured on a 400 MHz spectrometer.

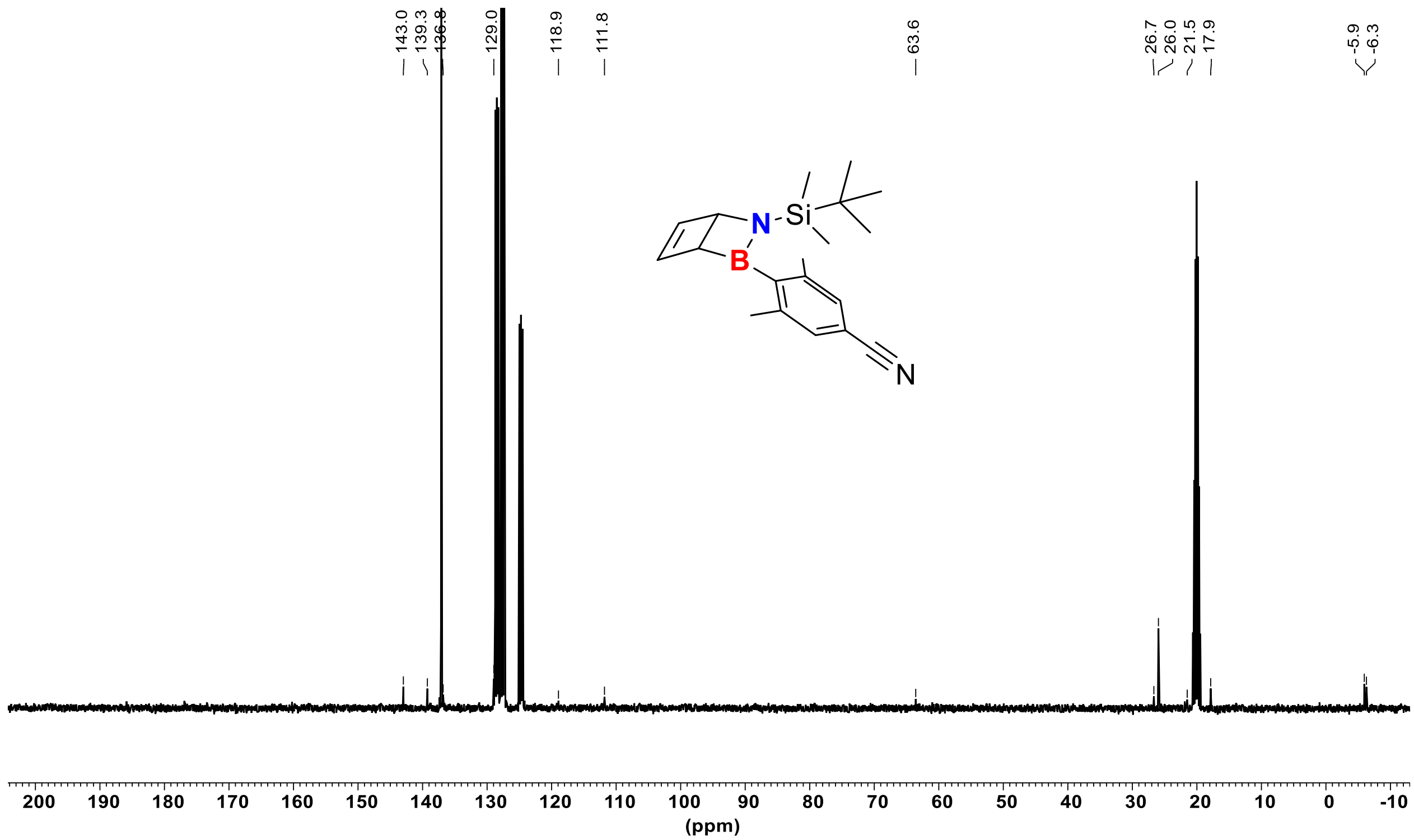

Figure S100: ^{13}C-{^{1}H} NMR spectrum of ^{BN}**D7** in toluene-d$_8$ measured on a 400 MHz spectrometer.

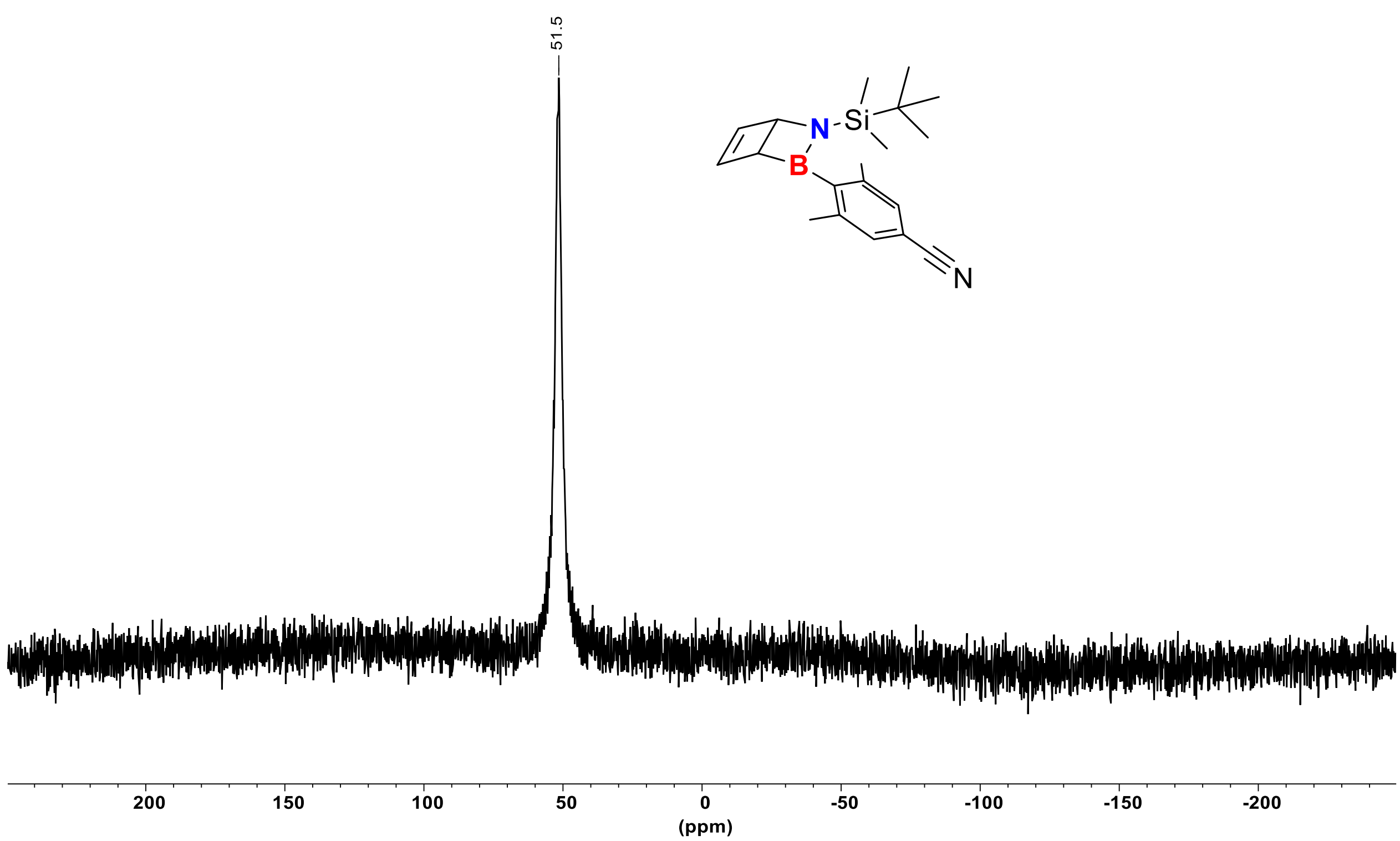

Figure S101: ^{1}B-{^{1}H} NMR spectrum of ^{BN}**D7** in toluene-d$_8$ measured on a 400 MHz spectrometer.

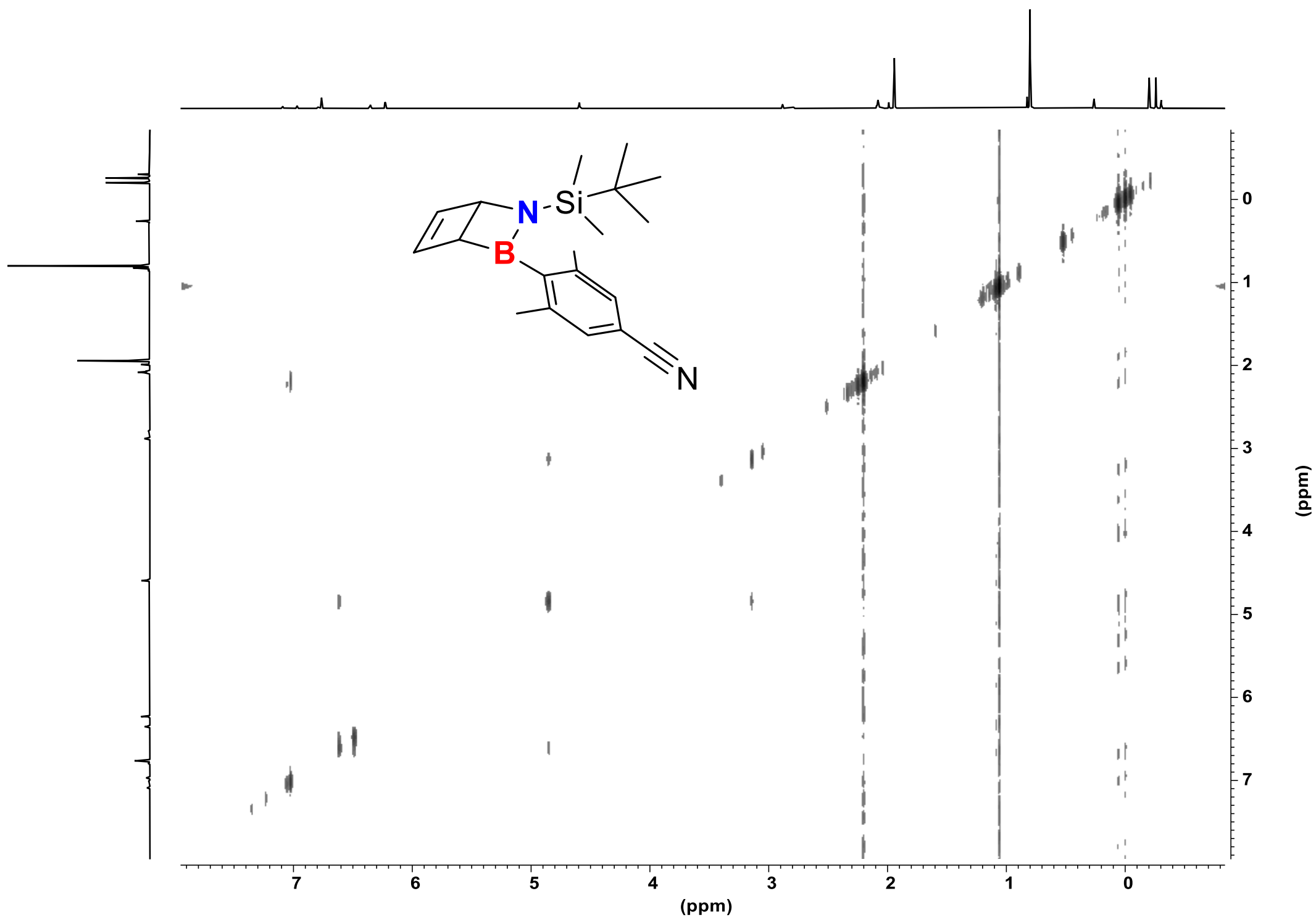

Figure S102: ^{1}H-^{1}H-COSY NMR spectrum of *^{BN}***D7** in toluene-d_8 measured on a 400 MHz spectrometer.

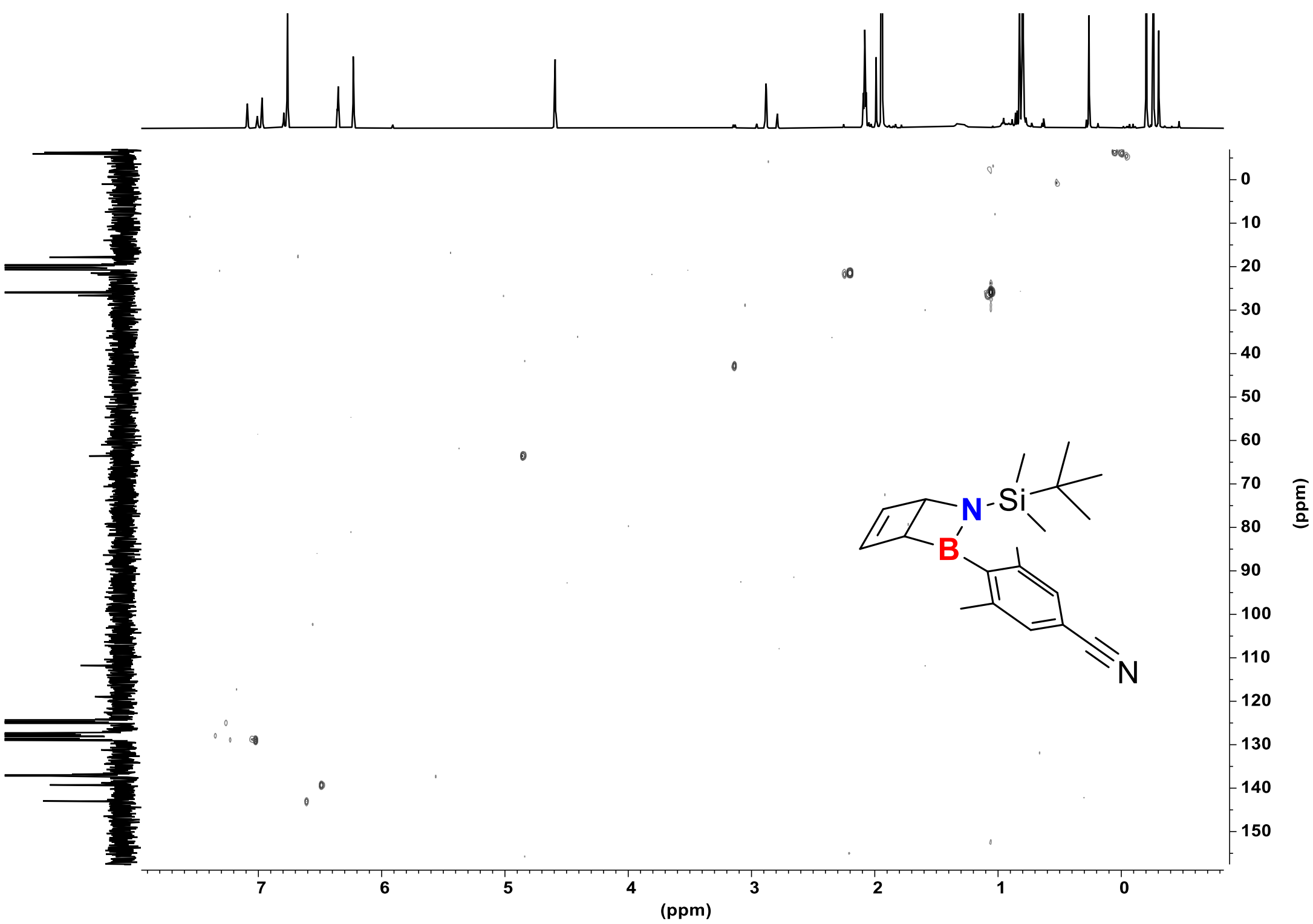

Figure S103: ^{1}H-^{13}C-HSQC NMR spectrum of *^{BN}***D7** in toluene-d_8 measured on a 400 MHz spectrometer.

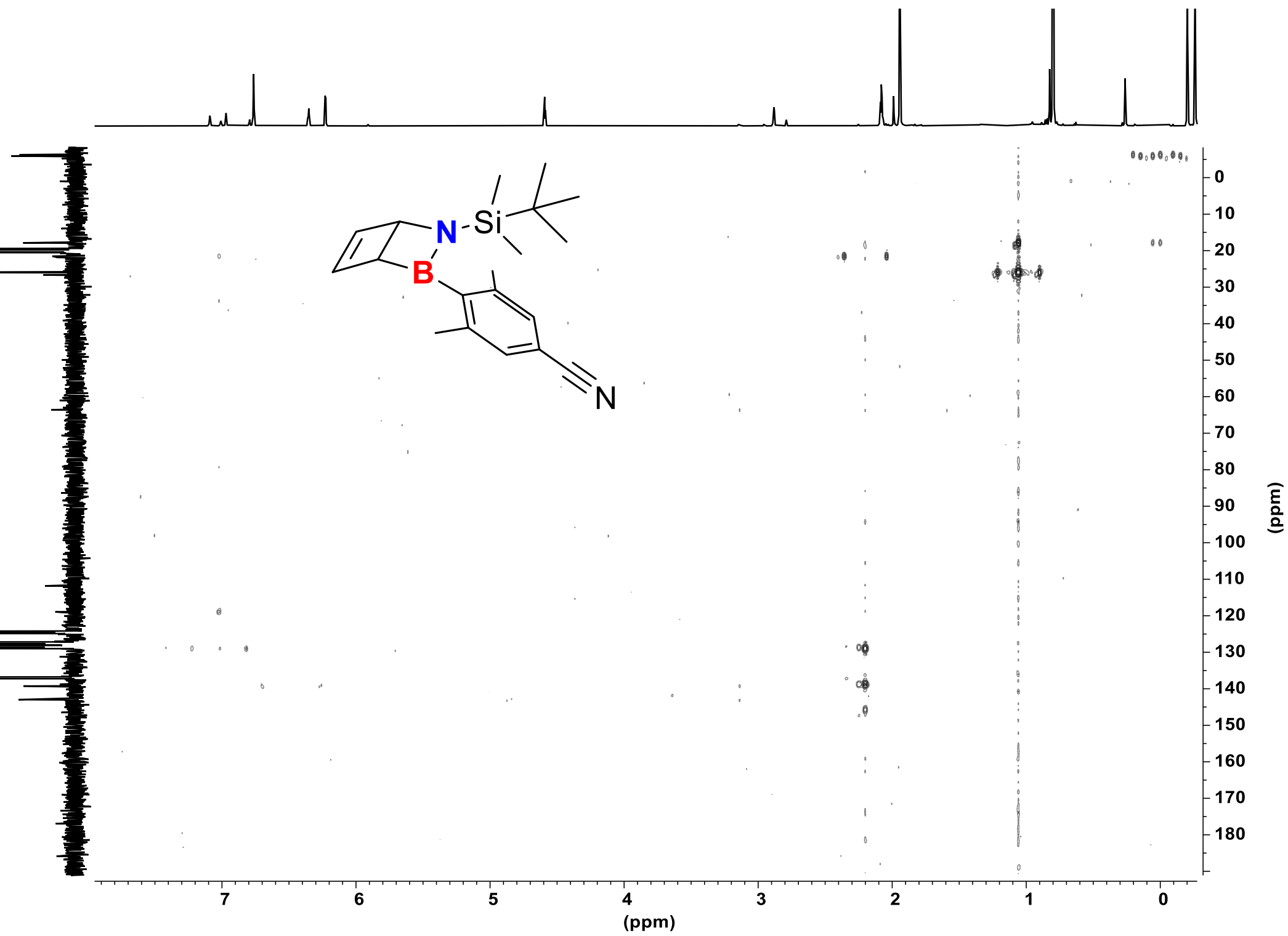

Figure S104: ^{1}H-^{13}C-HMBC NMR spectrum of ^{BN}**D7** in toluene-d_8 measured on a 400 MHz spectrometer.

NMR data ^{BN}**D11**

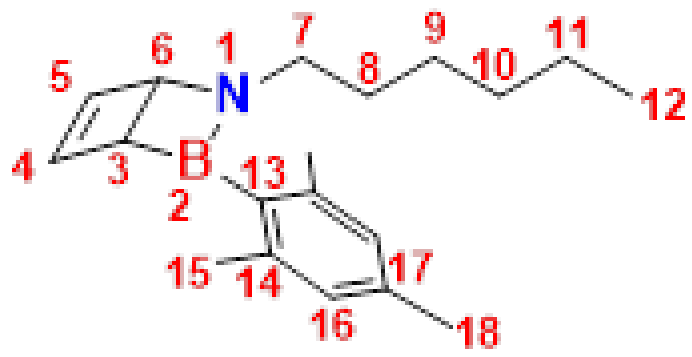

$C_{19}H_{28}BN$ (281.25 g/mol)

^{1}H NMR (600 MHz, **C_6D_{12}**): δ = 6.64 (s, 2H, H-16), 6.54 (m, 1H, H-4), 6.39 (d, $^3J_{HH}$ = 2.43 Hz, 1H, H-5), 4.42 (ps. t, 1H, H-6), 3.00 (m, 2H, H-7) overlapping with 2.95 (m, 1H, H-3), 2.18 (s, 3H, H-18) overlapping with 2.17 (s, 6H, H-15), 1.46 (quint, $^3J_{HH}$ = 7.28 Hz, 2H, H-8), 1.23 (m, 6H, H-9, H-10, H-11), 0.84 (m, 3H, H-12) ppm.

^{13}C-{^{1}H} NMR (151 MHz, **C_6D_{12}**): δ = 145.3, 140.2, 138.7, 137.5, 127.5, 64.2, 48.4, 41.5, 32.4, 29.6, 27.6, 23.5, 22.4, 21.4, 14.3 ppm.

^{11}B-{^{1}H} NMR (193 MHz, **C_6D_{12}**): δ = 45.0 ppm.

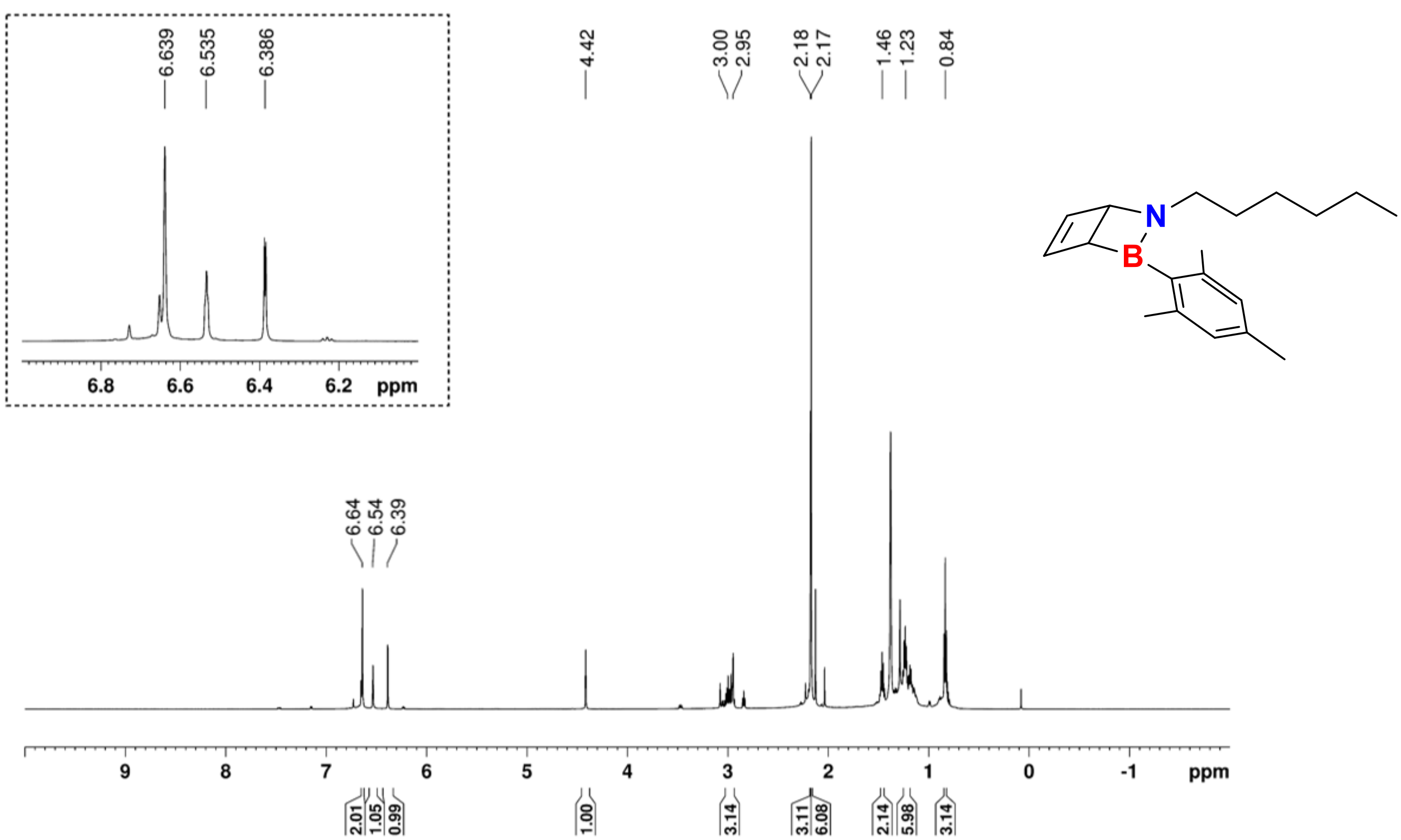

Figure S105. ^{1}H NMR spectrum of ^{BN}**D11** in C_6D_{12} measured on a 600 MHz spectrometer.

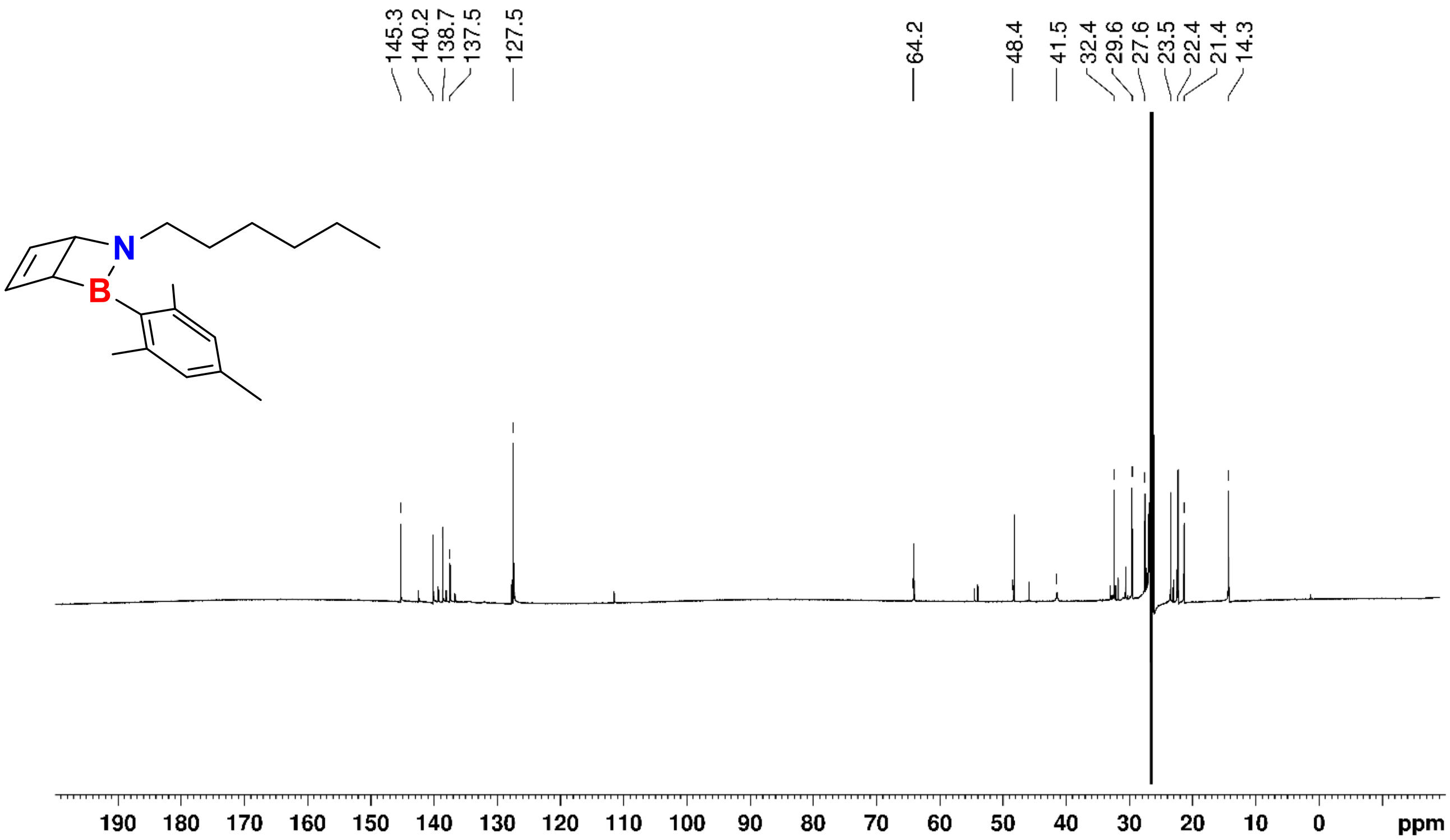

Figure S106. ^{13}C-{^{1}H} NMR spectrum of ***BN*D11** in C_6D_{12} measured on a 600 MHz spectrometer.

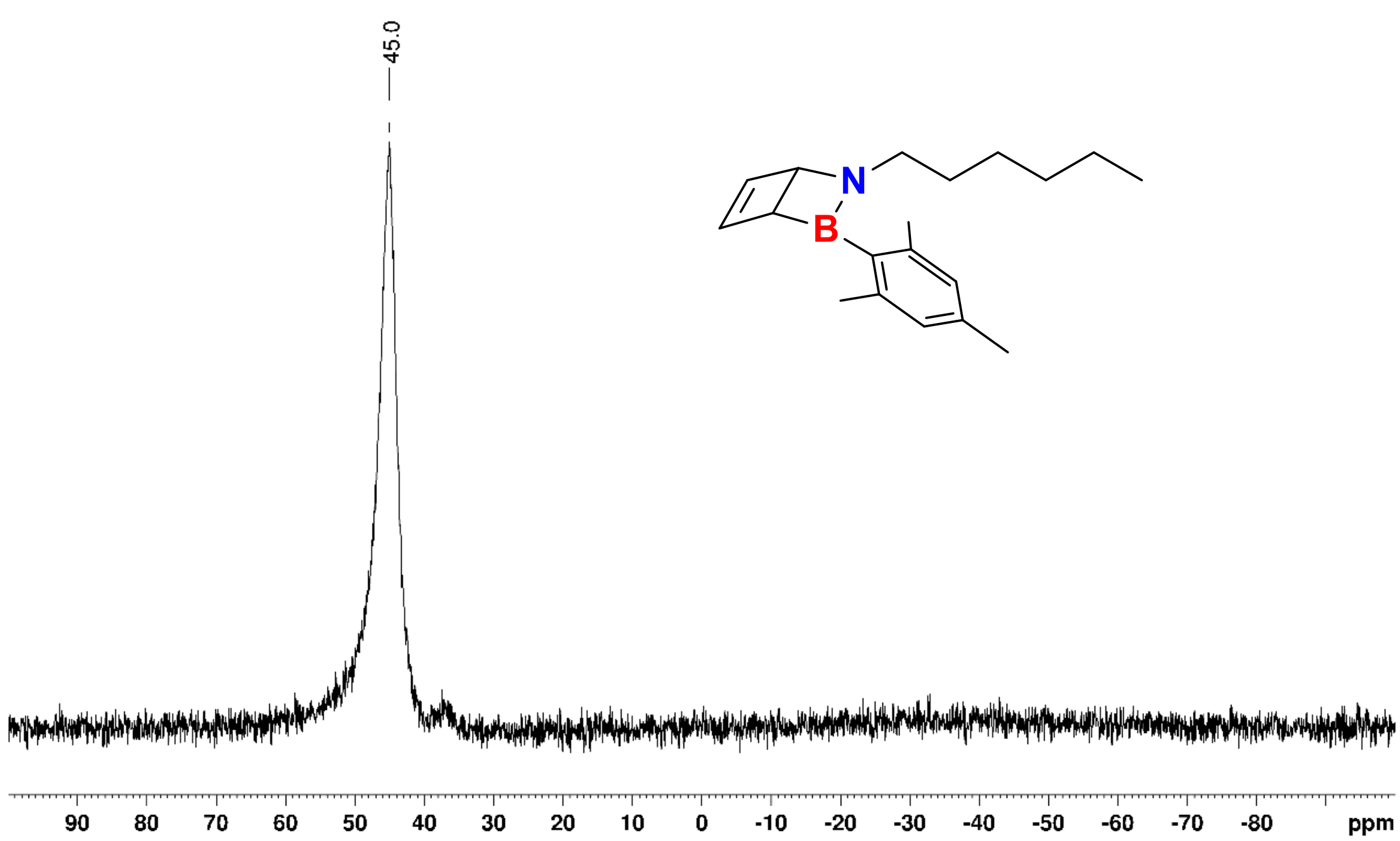

Figure S107. ^{11}B-{^{1}H} NMR spectrum of ***BN*D11** in C_6D_{12} measured on a 600 MHz spectrometer.

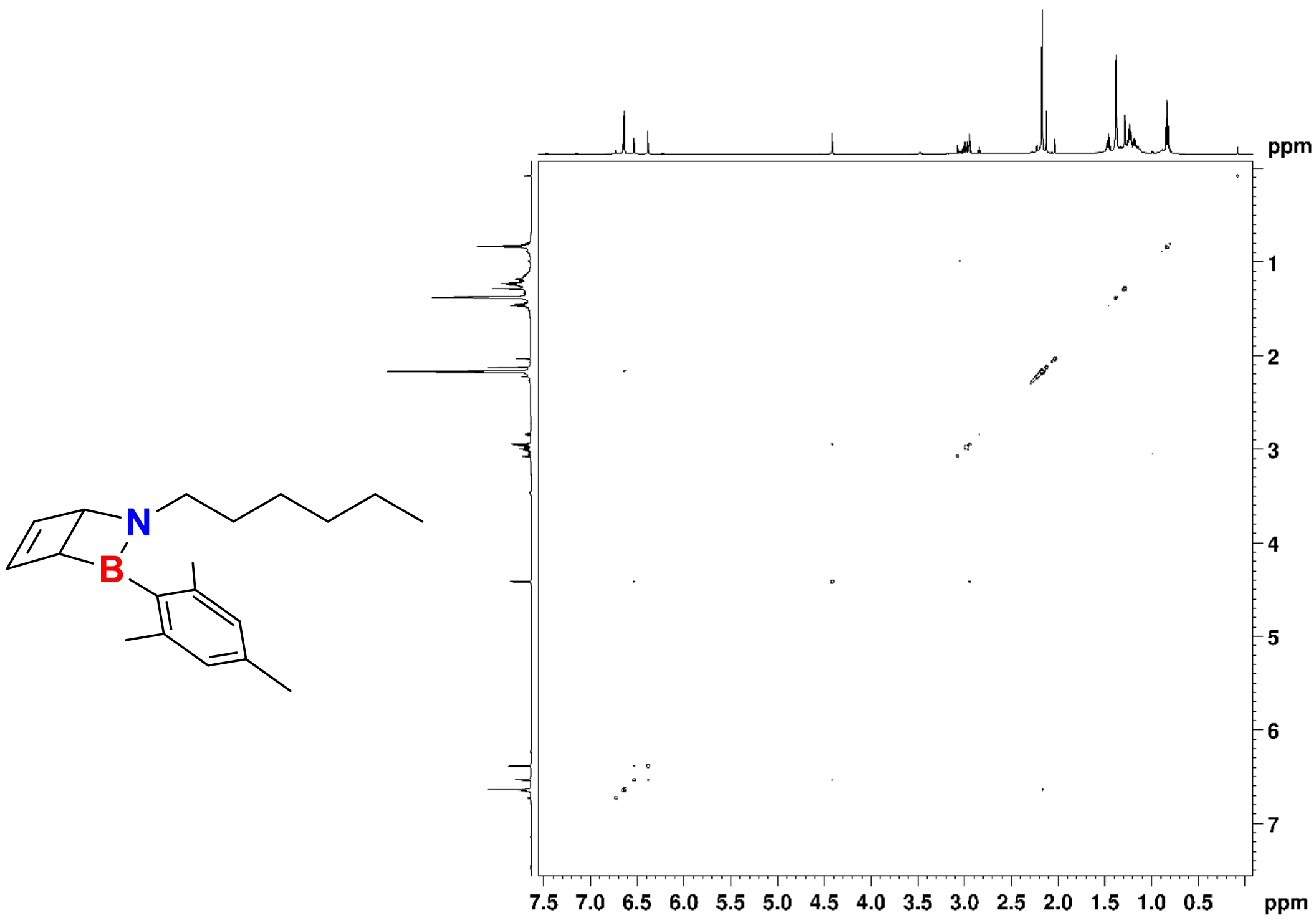

Figure S108. ^{1}H-^{1}H-COSY NMR spectrum of ***BN*D11** in C_6D_{12} measured on a 600 MHz spectrometer.

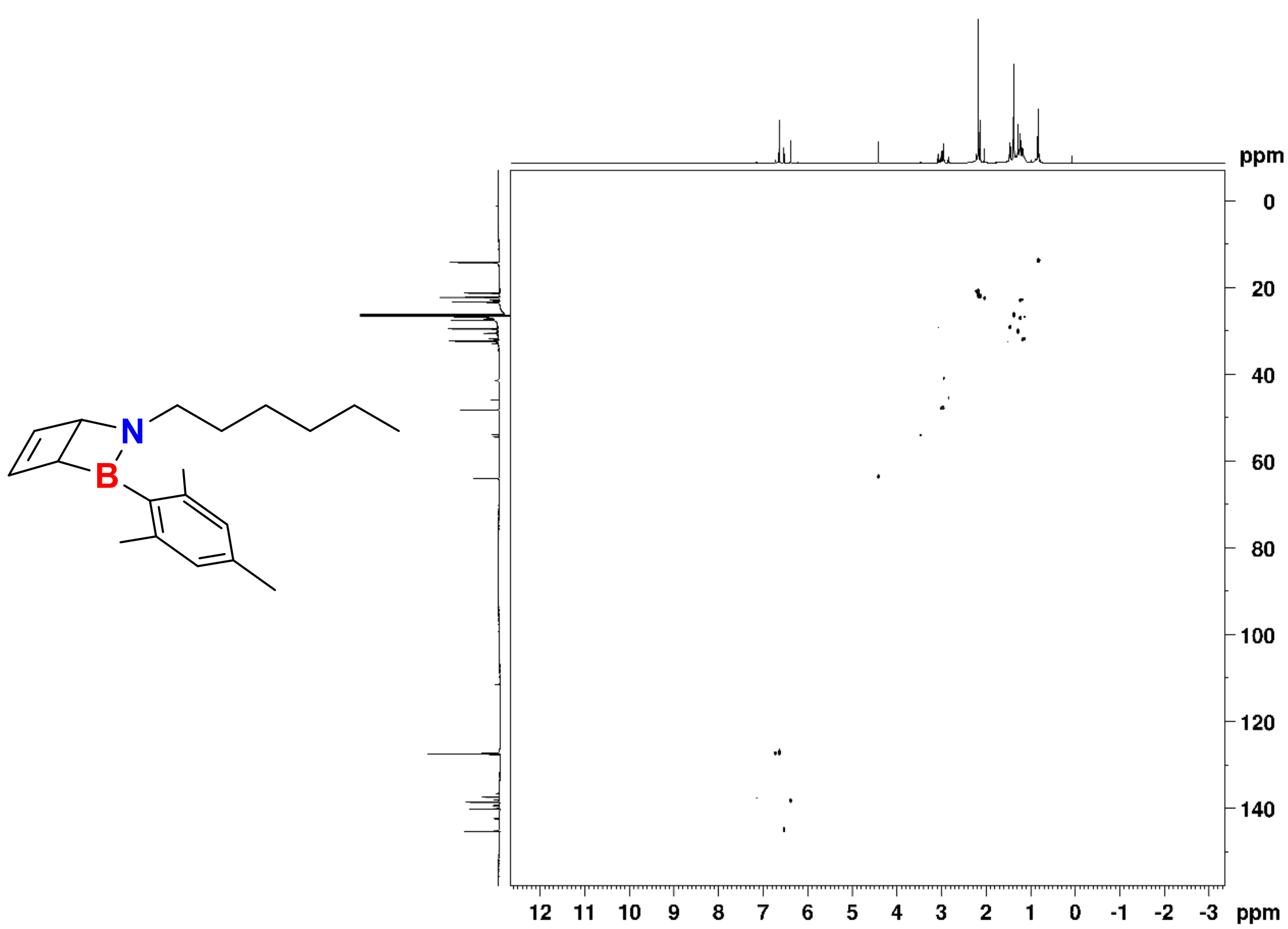

Figure S109. ^{1}H-^{13}C-HMBC NMR spectrum of ***BN*D11** in C_6D_{12} measured on a 600 MHz spectrometer.

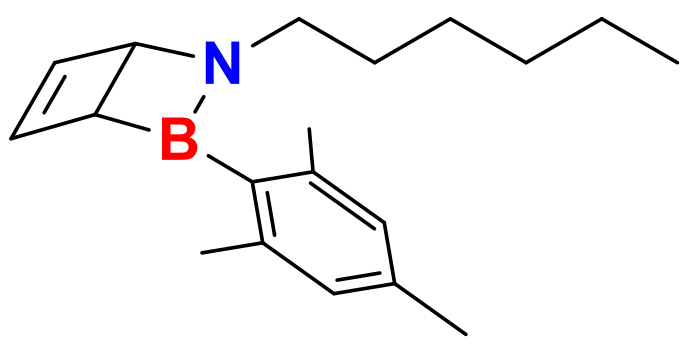

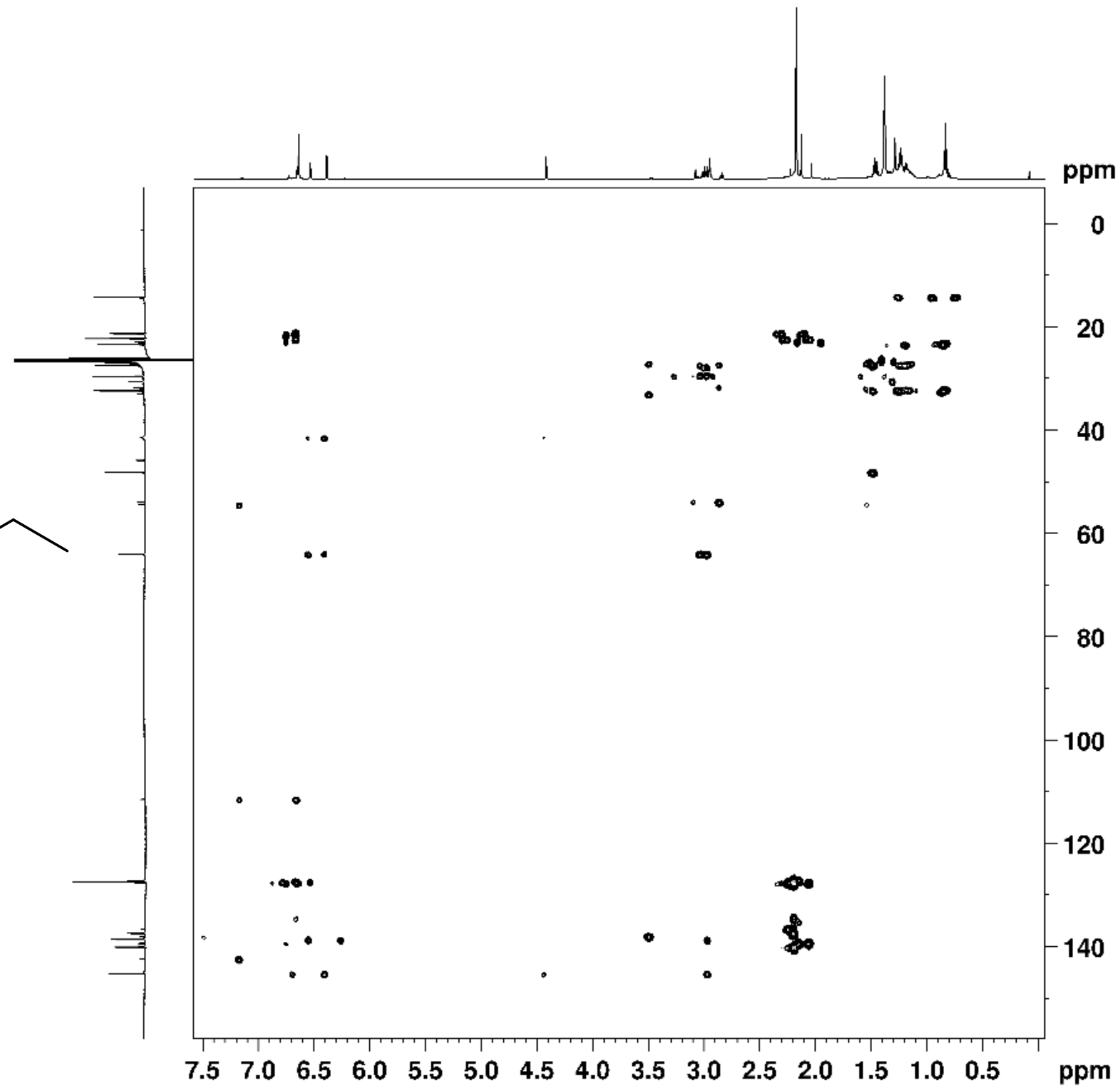

Figure S110. ^{1}H-^{13}C-HMBC NMR spectrum of ***BN*D11** in C_6D_{12} measured on a 600 MHz spectrometer.

5. Kinetic experiments

5.1 Sample preparation

The samples were prepared as described above in section 4 “Irradiation experiments”.

5.2 Experimental details

It was demonstrated earlier[11, 16] that the thermal ring opening follows first order kinetics. In agreement with that, we observed first order kinetics for the dihydroazaborinines ^{BN}**B3**-^{BN}**B7** and ^{BN}**B11**. The thermal ring-opening of each compound was monitored using proton NMR spectroscopy at four different temperatures. Since the half-lives vary, these four temperatures had to be adjusted accordingly. The specific temperatures measured for each compound are listed in Table S1. The sample was allowed to reach the desired temperature, then every 2 min or every 5 min a ^{1}H NMR spectrum was recorded (16 scans; total experiment time = 126 s) until at least 40% conversion to the dihydroazaborinine were observed.

Table S1. Temperatures considered for the kinetic analysis in K.

Subst.	T_1 (K)	T_2 (K)	T_3 (K)	T_4 (K)
^{BN}**B4**	348	353	358	363
^{BN}**B3**	353	358	363	368
^{BN}**B5**	353	358	363	368
^{BN}**B6**	352	363	368	373
^{BN}**B7**	352	363	368	373
^{BN}**B11**	328	333	338	343

Table S2. Rate constants calculated from NMR data at the temperatures given in Table S1 under the estimation of a first order kinetic.

	k_{T1} ($s^{-1} \cdot 10^4$)	k_{T2} ($s^{-1} \cdot 10^4$)	k_{T3} ($s^{-1} \cdot 10^4$)	k_{T4} ($s^{-1} \cdot 10^4$)
***BN*B4**	0.46	0.79	1.51	2.54
***BN*B3**	0.54	0.96	1.83	3.03
***BN*B5**	0.39	0.62	1.15	2.04
***BN*B6**	0.30	1.64	2.71	4.16
***BN*B7**	0.34	1.75	2.76	5.86
***BN*B11**	0.22	0.40	0.78	1.34

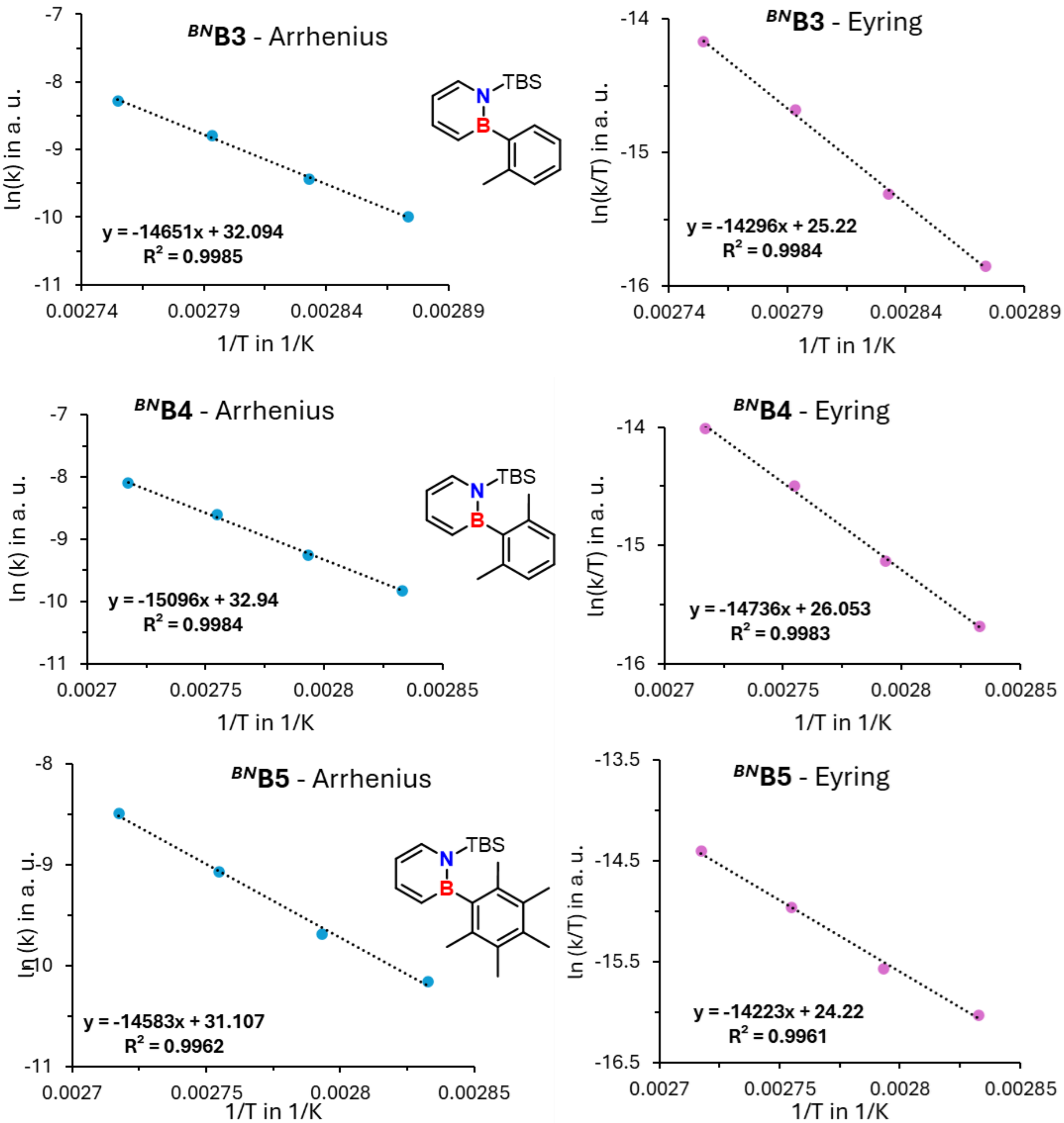

Figure S111. Arrhenius plots for the compounds *BN*B3-*BN*B5. The unit of the rate constants used is s^{-1}, as the reaction is first order.

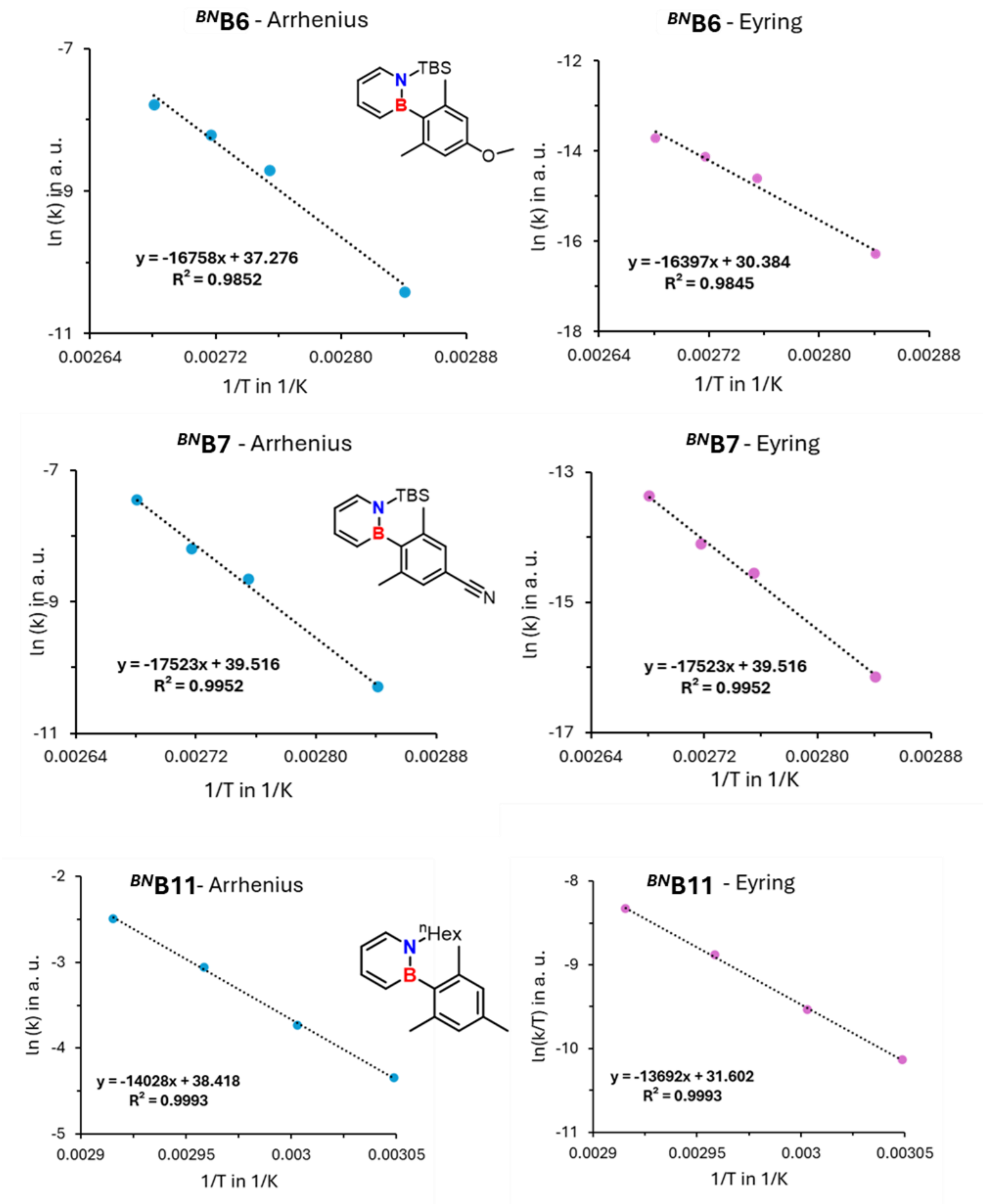

Figure S112. Arrhenius plots for the compounds ^{BN}**B6**-^{BN}**B7**, ^{BN}**B11**. The unit of the rate constants used is s^{-1}, as the reaction is first order.

The parameters from the linear regression of these Arrhenius and Eyring plots enable the determination of the following reaction barriers and half-lives (Table S3).

Table S3. Activation barriers determined from the Arrhenius treatment, $\Delta H^{\ddagger}_{ro}$, $\Delta S^{\ddagger}_{ro}$ and $\Delta G^{\ddagger}_{ro}$ calculated from the parameters of an Eyring plot, as well as calculated data for ***BN*B3-*BN*B7** and ***BN*B11**.

		***BN*D3**	***BN*D4**	***BN*D5**	***BN*D6**	***BN*D7**	***BN*D11**
Arrhenius	$E_{a1, exp}$ (kcal/mol)	29.1	30.0	29.0	33.3	34.8	27.9
	ln(A) (a. u.)	32.1	32.9	31.1	37.3	39.5	34.3
	$t_{1/2,}$ @ 298K (d)	0.6	1.1	0.9	3.8	5.2	0.01
Eyring	$\Delta H^{\ddagger}_{ro}$ (kcal/mol)	28.4	29.3	28.3	32.6	34.1	27.2
	$\Delta S^{\ddagger}_{ro}$ (cal/molK)	2.9	4.6	0.9	13.2	17.6	7.4
	$\Delta G^{\ddagger}_{ro}$ (kcal/mol)	27.5	27.9	28.0	28.7	28.6	25.0
Theory	$\Delta G^{\ddagger}_{ro}$ (kcal/mol)	*26.1*	*26.9*	*26.7*	*26.9*	*27.1*	*26.8*
	$\Delta\Delta G^{\ddagger}_{ro}$ (kcal/mol)	0.6	1.0	1.3	1.8	1.5	1.8

ro: ring opening

5.3 Time dependence (1. order kinetic)

For deriving the concentration dependence, the following assumptions are made. The reaction under consideration is of the type:

$$A \xrightarrow{k_1} B$$

The processe is assumed to be irreversible, follow a first order kinetic and has the rate constants k_1. This is in very good agreement with our experimental findings. The initial concentration of compound A is $A_0 = 1$. While the initial concentrations of B is $B_0 = 0$.

If compound A follows a simple first order kinetic, its concentrations behavior is given as:

$$\frac{d[A]}{dt} = -k_1 \cdot [A]$$

This equation can be transformed to

$$\frac{d[A]}{[A]} = -k_1 \cdot dt$$

An integration from A_0 to A (left side) or 0 to t (right side) respectively, results in

$$\ln[A] - \ln A_0 = -k_1 \cdot t$$

which finally rearranges to the well-known formula equation 1.

$$[\boldsymbol{A}] = \boldsymbol{A_0} \cdot \boldsymbol{e^{-k\ \cdot t}} \qquad \textbf{(1)}$$

6. DSC measurements

Sample preparation

In separate quartz J. Young the dihydroazaborinines ***BN*B3-*BN*B7** and ***BN*B11** were weight in using a fine scale, with an accuracy of 0.01 mg. These NMR tubes were then transferred to a glovebox and non-deuterated dodecane was added via a *Hamilton* syringe. The exact masses and volumes used can be found in **Table S4**. Each sample was irradiated with UV light of 280-400 nm and the conversion was qualitatively judged *via* ^{1}H and ^{11}B NMR spectroscopy. The tubes were again transferred to a glovebox. With an Eppendorf pipette 10 to 20 µL of this solution were transferred to pre-weighted aluminium crucibles. The crucibles were sealed with, also pre-weighted, concave lids in the glovebox. Usually, the curvature of the lid faces downward to ensure that the lid can be placed easily and perfectly centered on the crucible, preventing it from slipping before pressing. Since the crucibles had a relatively high fill level, capillary effects caused some solution to be drawn between the crucible and the lid or even completely out. As a consequence, such crucibles were often not airtight. Therefore, the lids were always placed with the curvature facing upward, keeping the contact surface between the crucible and the lid clean and ensuring that the capsules were properly sealed. The amount of solution was cross-checked by weighing on the fine scale. To about 0.1 mL of the mesitylene solution DCM-$d_2$was added, proton and ^{11}B NMR spectra were recorded and out of this data the conversion of the photoisomerization was determined.

Measurements

The compounds ***BN*B3-*BN*B5** were then heated up to 200 °C, ***BN*B6**, ***BN*B7** were then heated up to 220 °C and ***BN*B11** were then heated up to 180 °C with a rate of 1 K/min. This temperature was held for 10-20 minutes, reduced back to 10 °C (rate: 1 K/min) and held for 10 minutes. To ensure that the energy release is complete each sample was then heated up a second time to 180 °C/200 °C/220 °C.

Table S4. Experimental data according to the sample preparation, results of the DSC measurement as well as corrected stored energy with respect to the conversion.

	M	m	V(solvent)	c(stock)	con	m	ΔH_{exp}	ΔH_{corr}
	g/mol	mg	mL	g/mL	%	mg	kJ/kg	kcal/mol
^{BN}**B3**	283.3	11.13	0.4101	0.0271	quant	0.554	745.8	50.5
						0.542	760.1	51.5
						0.556	719.1	48.7
							**741.7**	**50.2**
^{BN}**B4**	297.32	12.18	0.3910	0.0311	quant	0.570	712.1	50.6
						0.561	695.8	49.4
						0.572	683.9	48.6
							**697.3**	**49.6**
^{BN}**B5**	339.41	16.44	0.3976	0.0413	quant	0.744	620.7	50.4
						0.763	593.0	48.1
						0.747	673.6	54.6
							**629.1**	**51.0**
^{BN}**B6**	327.35	17.47	0.3010	0.0580	quant	1.154	596.4	46.7
						1.114	601.4	47.1
						0.796	612.4	47.9
							**603.4**	**47.2**
^{BN}**B7**	322.33	11.54	0.2959	0.0390	quant	1.136	660.8	50.9
						1.141	668.6	51.5
						1.132	678.1	52.2
							**669.2**	**51.6**
^{BN}**B11**	281.25	24.30	0.3977	0.0611	quant	0.717	715.0	48.1
						0.900	703.4	47.3
						0.887	704.1	47.3
							**707.5**	**47.6**

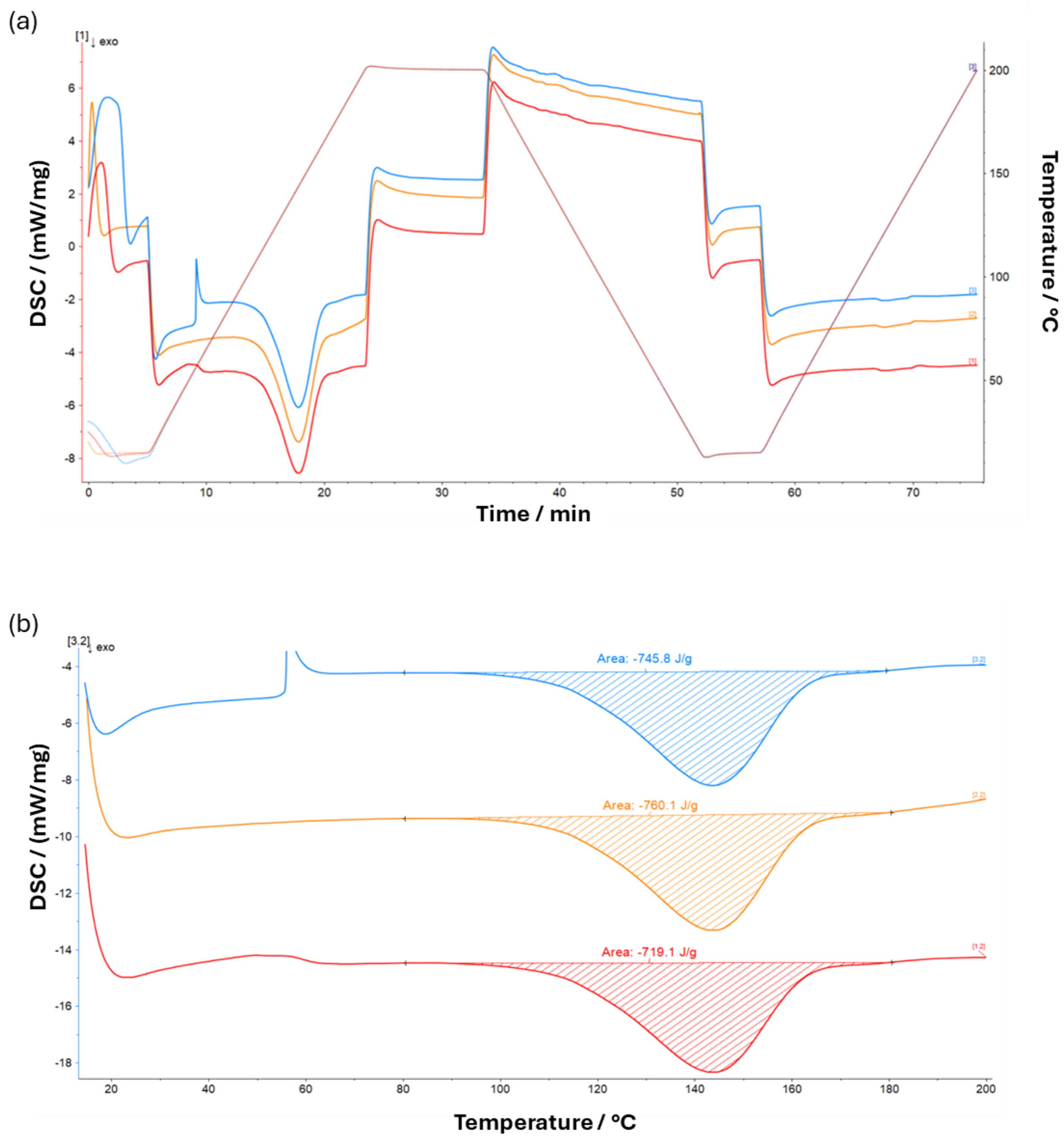

Figure S113. DSC measurements of compound ^{BN}**B3**. The area under the exothermal reaction peak corresponds to the released energy.

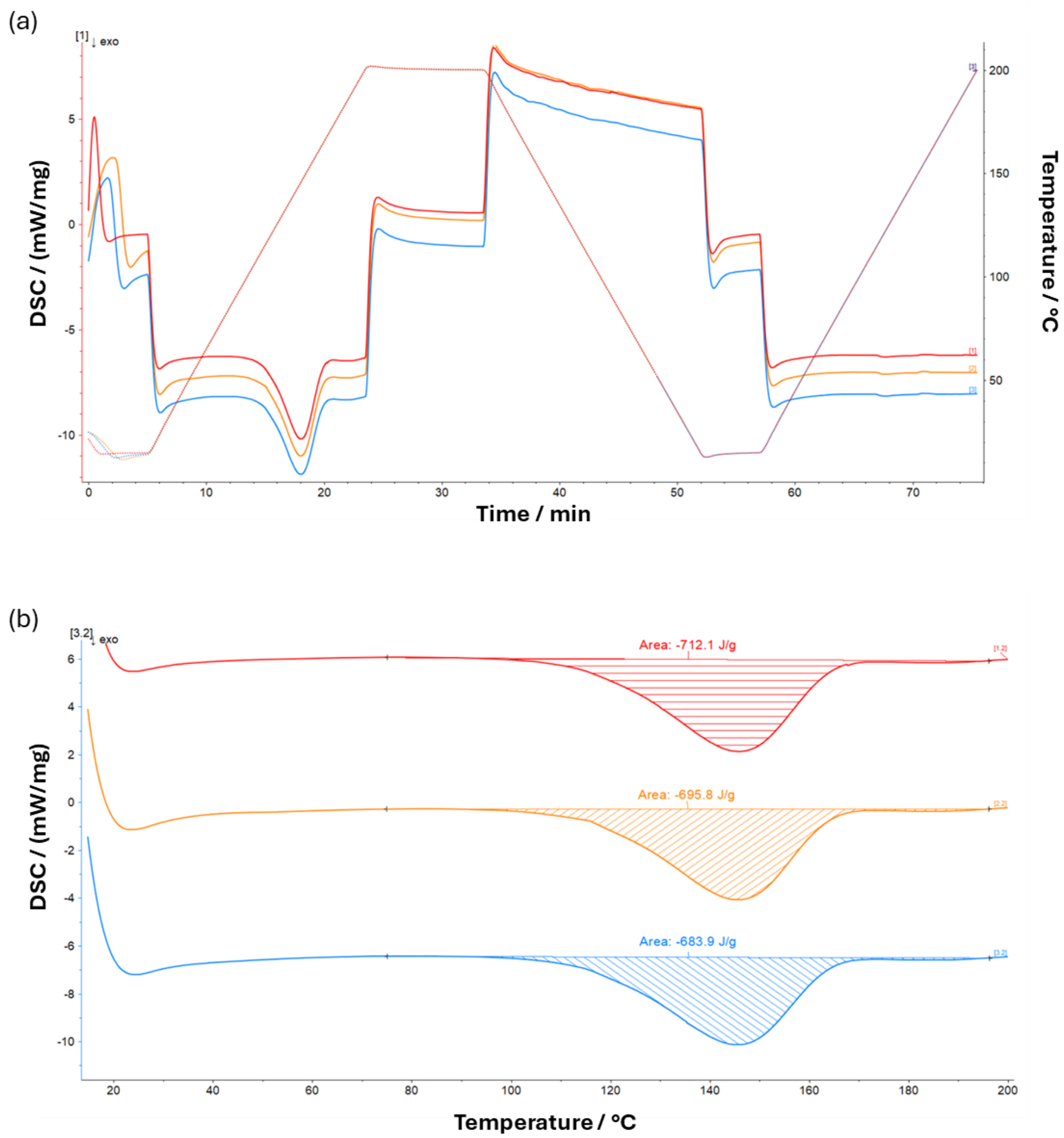

Figure S114. DSC measurements of compound ^{BN}**B4**. The area under the exothermal reaction peak corresponds to the released energy.

(a)

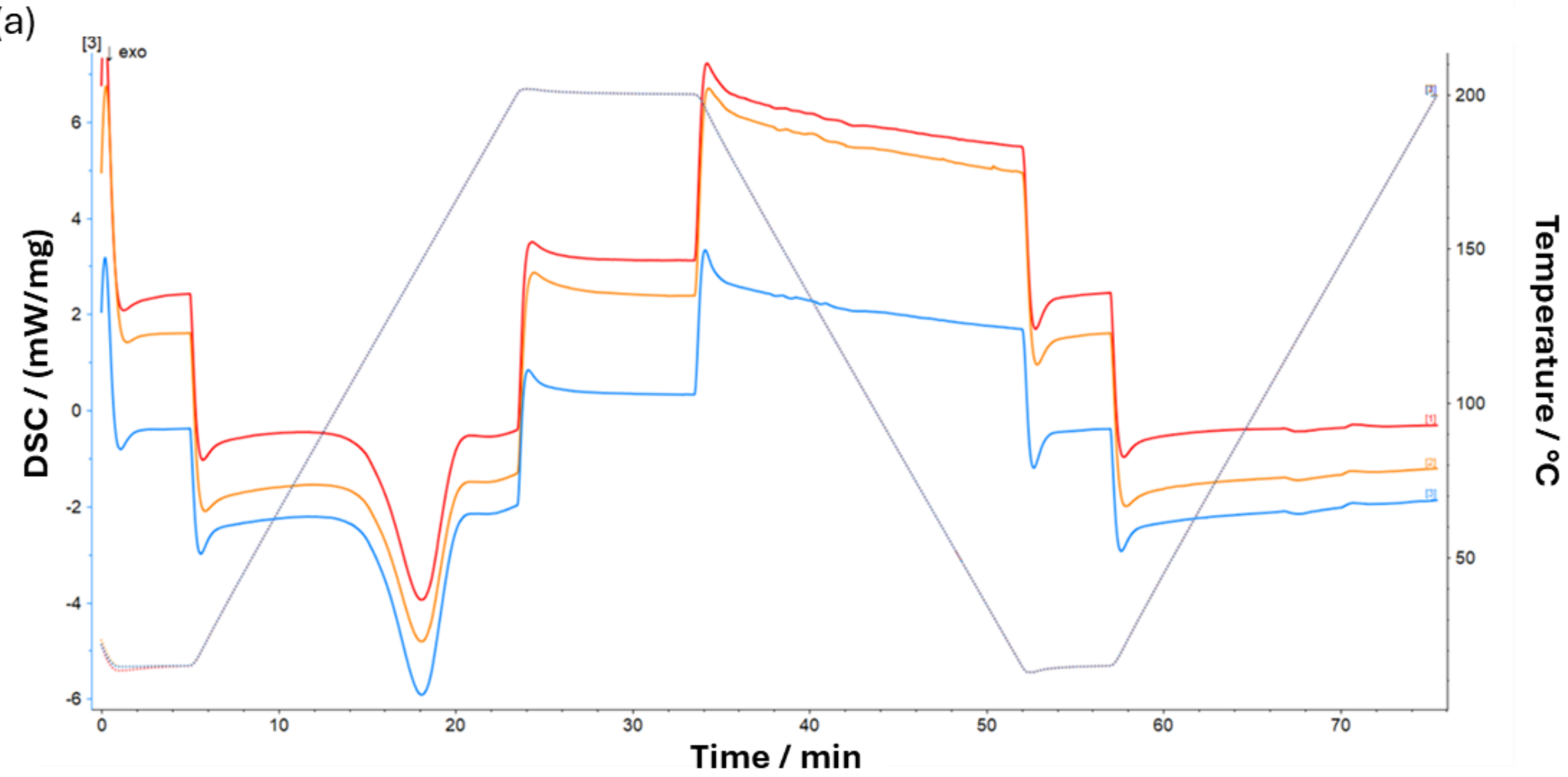

(b)

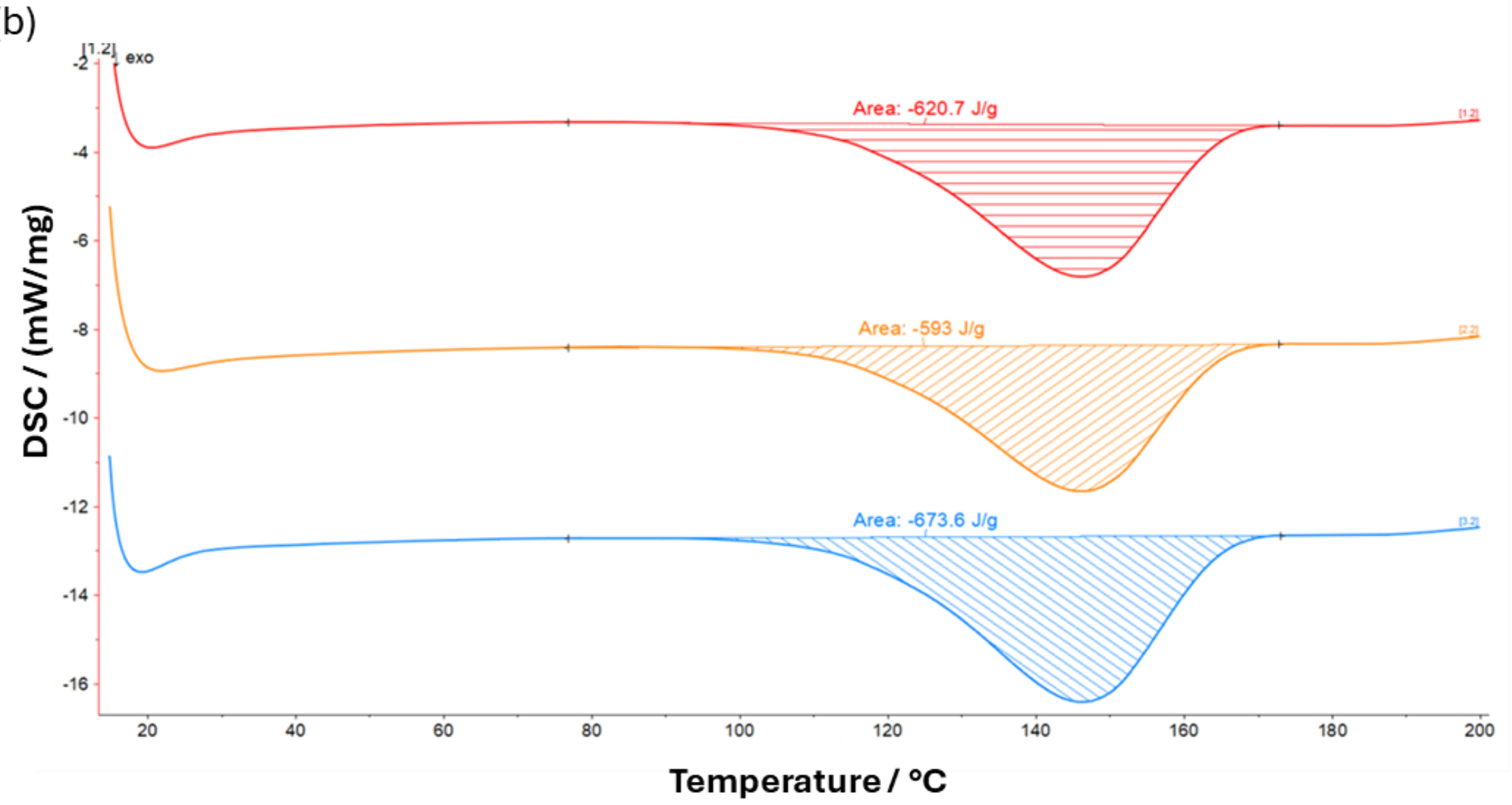

Figure S115. DSC measurements of compound ^{BN}**B5**. The area under the exothermal reaction peak corresponds to the released energy.

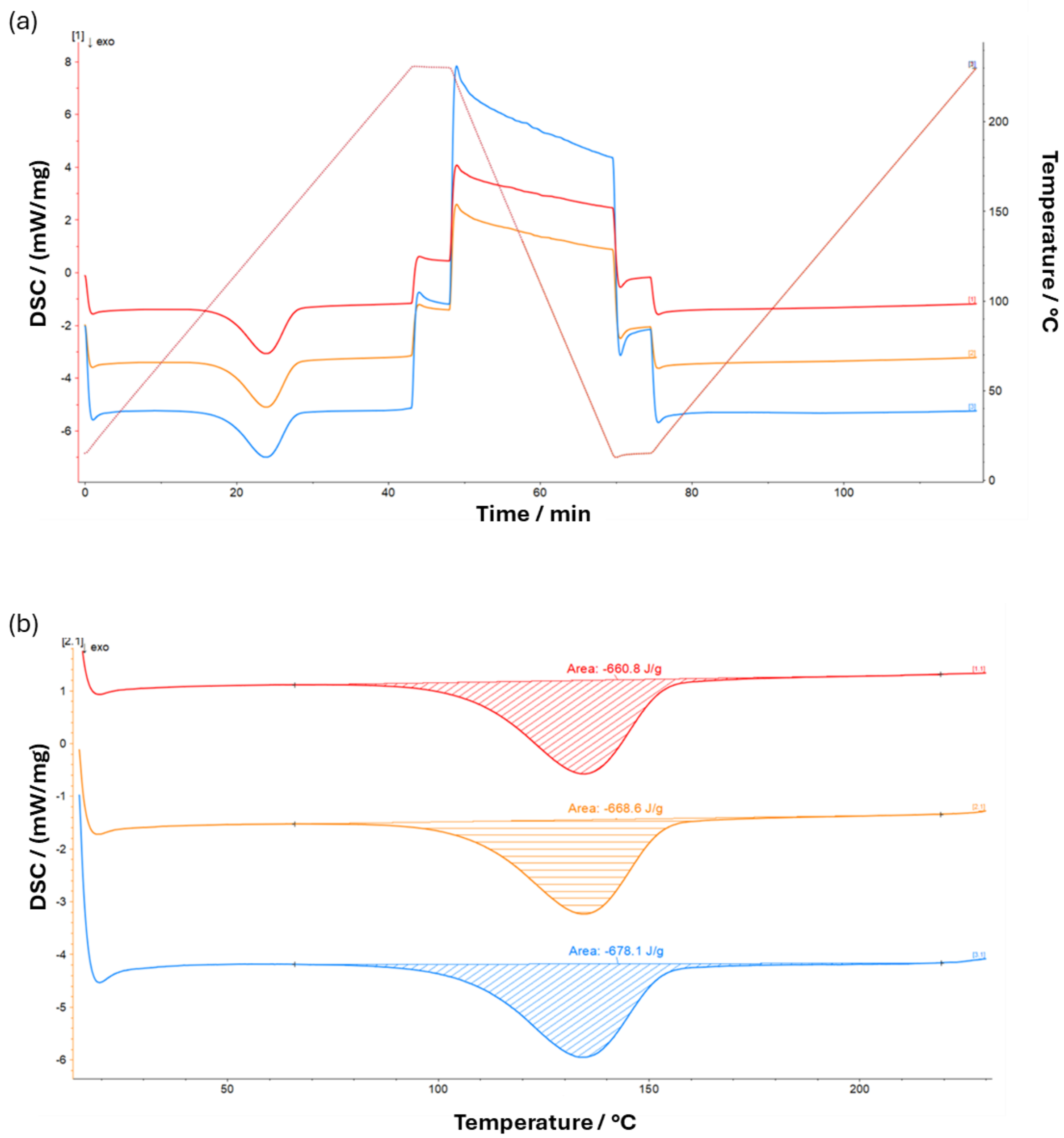

Figure S116. DSC measurements of compound ^{BN}**B6**. The area under the exothermal reaction peak corresponds to the released energy.

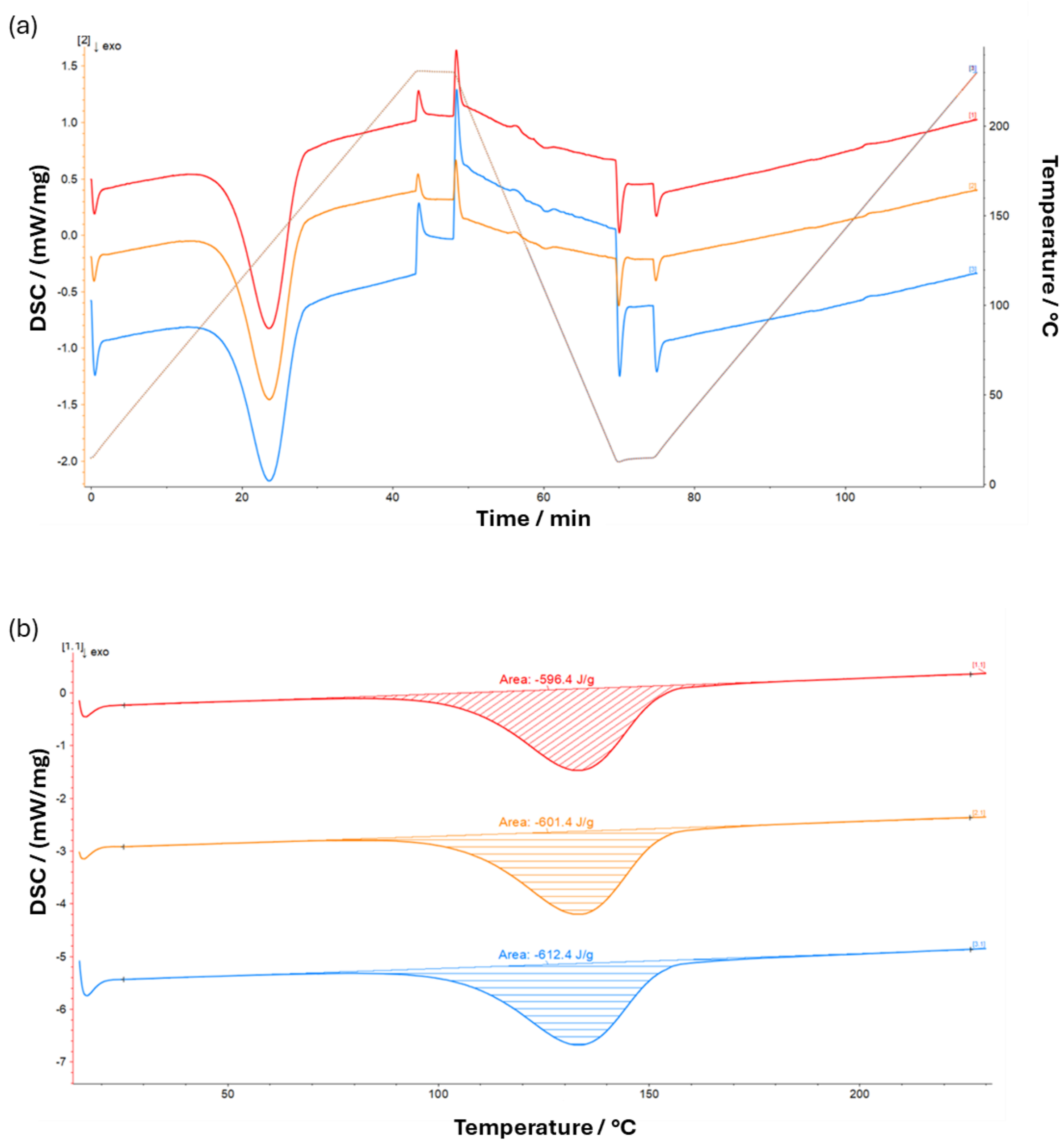

Figure S117. DSC measurements of compound ^{BN}**B7**. The area under the exothermal reaction peak corresponds to the released energy.

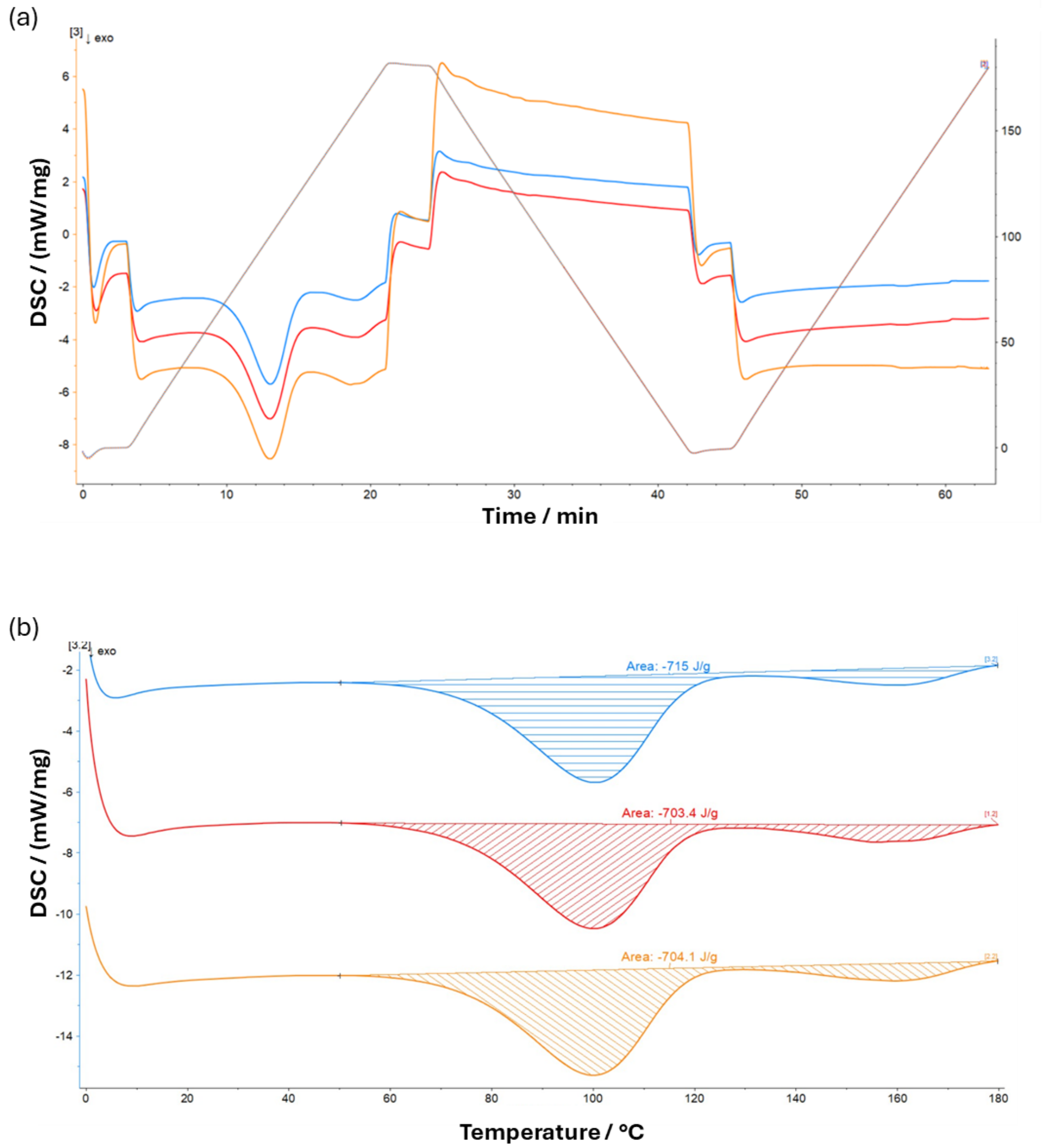

Figure S118. DSC measurements of compound ^{BN}**B11**. The area under the exothermal reaction peak corresponds to the released energy.

7. Crystal structures

Refinement details

The implementation NoSpherA2 for non-spherical atom form factors in *Olex2* makes use of tailor made aspherical atomic form factors calculated from a Hirshfeld-partitioned electron density (ED), not from spherical atom form factors.[17] The electron density is calculated from a Gaussian basis set single determinant SCF wavefunction for a fragment of the crystal. This fragment can be embedded in an electrostatic crystal field by employing cluster charges or modelled using implicit solvation models, depending on the software used. This was computed using B3LYP/6-31G(d,p),[18-23] normal integration accuracy, a charge of 0 and a multiplicity of 1 with Orca 5.0.[24, 25]

Table S5. Parameters and results of the X-ray diffraction measurement of compound ^{BN}**B8**.

Parameter	unit	^{BN}**B8**
Molecular formula		$C_{13}H_{16}BN$
CCDC		2432739
$D_{calc.}$	g cm^{-3}	1.104
μ	mm^{-1}	0.470
Molar weight	g/mol	197.103
Color		clear colorless
Shape		block-shaped
Size	mm^3	0.17×0.12×0.10
T/K		150.00(10)
Crystal System		monoclinic
Space Group		$P2_1/c$
a/Å		8.7146(1)
b/Å		15.8191(2)
c/Å		9.4327(2)
α/°		90
β/°		114.251(2)
γ/°		90
V	Å^3	1185.61(4)
Z		4
Z'		1
Wavelength	Å	1.54184
Radiation type		Cu K□
Θ_{min}	°	5.57
Θ_{max}	°	79.84
Measured Refl's.		43426
Indep't Refl's		2565
Refl's I≥2 σ(I)		2423
R_{int}		0.0165
Parameters		281
Restraints		0
Largest Peak		0.0833
Deepest Hole		-0.0958
GooF		1.2421
wR_2 (all data)		0.0452
wR_2		0.0448
R_1 (all data)		0.0184
R_1		0.0172

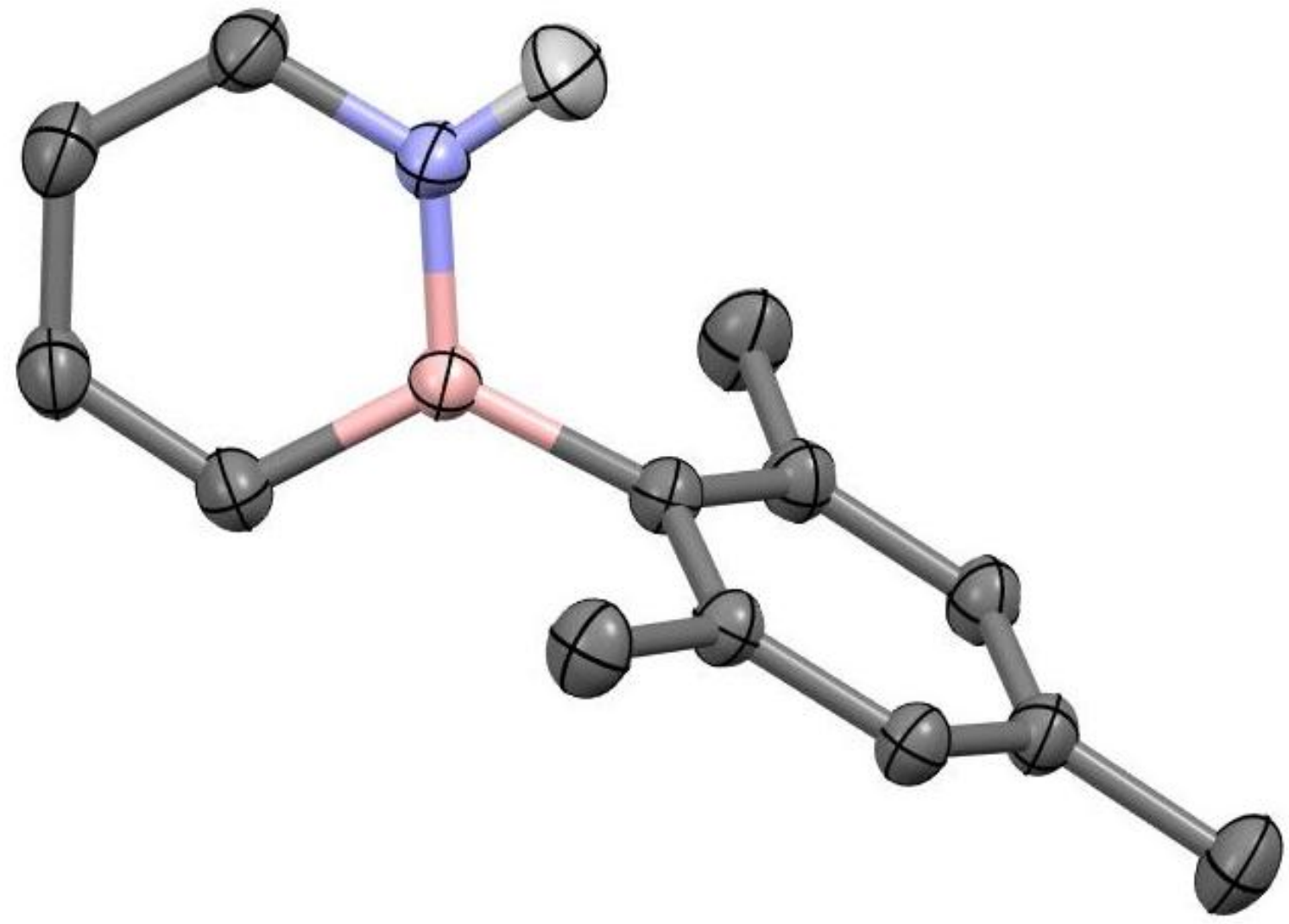

Figure S119. Crystal structure of ^{BN}**B6**. Hydrogens are omitted for clarity and thermal ellipsoids are drawn at the 50% probability level.

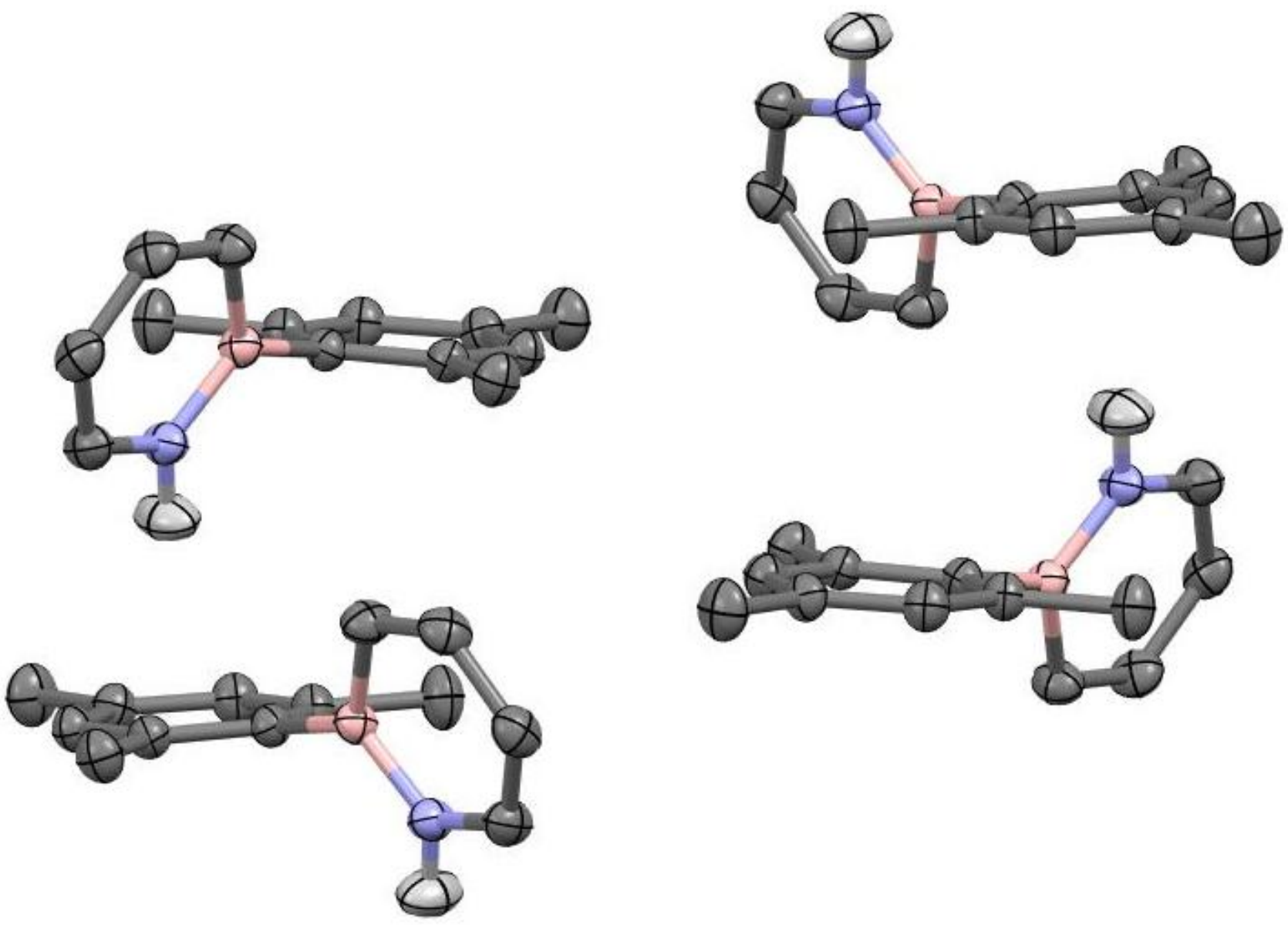

Figure S120. Packing of ^{BN}**B6** in the crystal structure. Hydrogens are omitted for clarity and thermal ellipsoids are drawn at the 50% probability level.

7. Computations

Electronic structure computations: All geometries were optimized using the B3LYP[18, 19, 26] functional and the 6-311+G(d,p) basis set[27-31] in Gaussian 16.[32] Verification of the stationary points involved analytical frequency computations, confirming minima with zero imaginary vibrational frequencies and one imaginary vibrational frequency for transition states. For a verification of the transitions states intrinsic reaction coordinates were calculated by using the local quadratic approximation.[33, 34] Free energies were computed using standard thermochemistry equations implemented in Gaussian. In this section, total energies are given in Hartree/particle and kcal/mol (Tables S6-S13).

Absolute and relative energies of the optimized structures (B3LYP/6-311+G(d,p))

Table S6. Total energy E and Gibbs energy (G, T = 298.15 K, p = 1 atm) of the optimized structures that occur during the thermal ring opening of N-H-B-X substituted ***^{BN}*D** (X see below). Relative Gibbs energies (G_{rel}) are given with respect to the corresponding Dewar isomers.

N	B		E	G	G_{rel}
	X		Hartree/particle	Hartree/particle	kcal/mol
H	Ph	Aza	-466.86793	-466.72424	-61.4
		TS	-466.73089	-466.59206	21.5
		Dewar	-466.76700	-466.62637	0.0
	o-Tol	Aza	-506.19114	-506.02197	-60.5
		TS	-506.05507	-505.89153	21.3
		Dewar	-506.09139	-505.92553	0.0
	Xyl	Aza	-545.51597	-545.32331	-62.1
		TS	-545.37941	-545.19129	20.7
		Dewar	-545.41312	-545.22431	0.0
	Mes	Aza	-584.84616	-584.63033	-65.2
		TS	-584.70707	-584.49520	19.6
		Dewar	-584.74301	-584.52647	0.0
	Ph*	Aza	-663.48482	-663.21499	-62.4
		TS	-663.34817	-663.08283	20.5
		Dewar	-663.38260	-663.11553	0.0
	p-OMe	Aza	-660.07229	-659.85127	-62.7
		TS	-659.93641	-659.71968	19.9
		Dewar	-659.97290	-659.75143	0.0
	p-CN	Aza	-637.78512	-637.59621	-64.0
		TS	-637.64765	-637.46347	19.3
		Dewar	-637.68259	-637.49419	0.0

Table S7. Total energy E and Gibbs energy (G, T = 298.15 K, p = 1 atm) of the optimized structures that occur during the thermal ring opening of N-Me-B-X substituted ^{BN}**D** (X see below). Relative Gibbs energies (G_{rel}) are given with respect to the corresponding Dewar isomers.

N	B		E	G	G_{rel}
	X		Hartree/particle	Hartree/particle	kcal/mol
Me	Ph	Aza	-506.17878	-506.00981	-57.0
		TS	-506.04410	-505.88054	24.1
		Dewar	-506.08511	-505.91900	0.0
	o-Tol	Aza	-545.50409	-545.31121	-59.0
		TS	-545.36949	-545.18092	22.8
		Dewar	-545.40899	-545.21726	0.0
	Xyl	Aza	-584.82975	-584.61160	-59.5
		TS	-584.69513	-584.48083	22.6
		Dewar	-584.73298	-584.51677	0.0
	Mes	Aza	-624.15708	-623.91521	-59.4
		TS	-624.02266	-623.78460	22.6
		Dewar	-624.06054	-623.82058	0.0
	Ph*	Aza	-702.79853	-702.50357	-58.8
		TS	-702.66380	-702.37280	23.2
		Dewar	-702.70237	-702.40984	0.0
	p-OMe	Aza	-699.38600	-699.13949	-59.1
		TS	-699.25190	-699.00895	22.9
		Dewar	-699.28937	-699.04538	0.0
	p-CN	Aza	-677.09894	-676.88461	-58.7
		TS	-676.96364	-676.75325	23.7
		Dewar	-677.00181	-676.79103	0.0

Table S8 Total energy E and Gibbs energy (G, T = 298.15 K, p = 1 atm) of the optimized structures that occur during the thermal ring opening of N-Et-B-X substituted ^{BN}**D** (X see below). Relative Gibbs energies (G_{rel}) are given with respect to the corresponding Dewar isomers.

N	B		E	G	G_{rel}
	X		Hartree/particle	Hartree/particle	kcal/mol
Et	Ph	Aza	-545.50511	-545.30998	-56.9
		TS	-545.36762	-545.17704	26.5
		Dewar	-545.41169	-545.21927	0.0
	o-Tol	Aza	-584.83087	-584.61086	-59.0
		TS	-584.69648	-584.48174	22.0
		Dewar	-584.73500	-584.51684	0.0
	Xyl	Aza	-624.15584	-623.91038	-58.0
		TS	-624.02209	-623.78156	22.8
		Dewar	-624.05931	-623.81788	0.0
	Mes	Aza	-663.48316	-663.21383	-58.0
		TS	-663.34536	-663.07988	26.1
		Dewar	-663.38678	-663.12144	0.0
	Ph*	Aza	-742.12486	-741.80230	-58.0
		TS	-741.99073	-741.67341	22.9
		Dewar	-742.02878	-741.70987	0.0
	p-OMe	Aza	-738.71206	-738.43864	-58.3
		TS	-738.57453	-738.30454	25.9
		Dewar	-738.61575	-738.34575	0.0
	p-CN	Aza	-716.42507	-716.18344	-58.7
		TS	-716.29076	-716.05410	22.5
		Dewar	-716.32784	-716.08992	0.0

Table S9. Total energy E and Gibbs energy (G, T = 298.15 K, p = 1 atm) of the optimized structures that occur during the thermal ring opening of N-^{i}Pr-B-X substituted ^{BN}**D** (X see below). Relative Gibbs energies (G_{rel}) are given with respect to the corresponding Dewar isomers.

N	B		E	G	G_{rel}
	X		Hartree/particle	Hartree/particle	kcal/mol
^{i}Pr	Ph	Aza	-584.82963	-584.60786	-56.1
		TS	-584.69424	-584.47768	25.6
		Dewar	-584.73704	-584.51847	0.0
	o-Tol	Aza	-624.15507	-623.90906	-59.0
		TS	-624.01254	-623.77020	28.2
		Dewar	-624.05862	-623.81507	0.0
	Xyl	Aza	-663.48009	-663.20928	-56.7
		TS	-663.33671	-663.06852	31.6
		Dewar	-663.38473	-663.11889	0.0
	Mes	Aza	-702.80741	-702.51305	-57.7
		TS	-702.67180	-702.38060	25.4
		Dewar	-702.71215	-702.42116	0.0
	Ph*	Aza	-781.44933	-781.10058	-57.3
		TS	-781.30585	-780.96092	30.4
		Dewar	-781.35432	-781.00933	0.0
	p-OMe	Aza	-778.03636	-777.73622	-56.8
		TS	-777.90104	-777.60483	25.7
		Dewar	-777.94108	-777.64573	0.0
	p-CN	Aza	-755.74940	-755.48265	-59.0
		TS	-755.61171	-755.34820	25.4
		Dewar	-755.65251	-755.38866	0.0

Table S10. Total energy E and Gibbs energy (G, T = 298.15 K, p = 1 atm) of the optimized structures that occur during the thermal ring opening of N-^{t}Bu-B-X substituted ^{BN}**D** (X see below). Relative Gibbs energies (G_{rel}) are given with respect to the corresponding Dewar isomers.

N	B		E	G	G_{rel}
	X		Hartree/particle	Hartree/particle	kcal/mol
^{t}Bu	Ph	Aza	-624.13990	-623.89159	-49.5
		TS	-624.01192	-623.76920	27.3
		Dewar	-624.05759	-623.81265	0.0
	o-Tol	Aza	-663.46533	-663.19128	-48.4
		TS	-663.33676	-663.06795	29.0
		Dewar	-663.38273	-663.11422	0.0
	Xyl	Aza	-702.79056	-702.49162	-48.8
		TS	-702.66100	-702.36667	29.6
		Dewar	-702.70801	-702.41389	0.0
	Mes	Aza	-742.11776	-741.79304	-47.6
		TS	-741.98853	-741.67033	29.4
		Dewar	-742.03539	-741.71725	0.0
	Ph*	Aza	-820.75926	-820.38402	-49.0
		TS	-820.63015	-820.25885	29.5
		Dewar	-820.67761	-820.30588	0.0
	p-OMe	Aza	-817.34517	-817.01679	-47.1
		TS	-817.21753	-816.89470	29.6
		Dewar	-817.26425	-816.94180	0.0
	p-CN	Aza	-795.06025	-794.76496	-49.2
		TS	-794.92993	-794.63959	29.5
		Dewar	-794.97712	-794.68654	0.0

Table S11. Total energy E and Gibbs energy (G, T = 298.15 K, p = 1 atm) of the optimized structures that occur during the thermal ring opening of N-TMS-B-X substituted ^{BN}**D** (X see below). Relative Gibbs energies (G_{rel}) are given with respect to the corresponding Dewar isomers.

N	B		E	G	G_{rel}
	X		Hartree/particle	Hartree/particle	kcal/mol
TMS	Ph	Aza	-875.61097	-875.37769	-52.9
		TS	-875.47811	-875.24987	27.3
		Dewar	-875.52435	-875.29341	0.0
	o-Tol	Aza	-914.93669	-914.67685	-53.1
		TS	-914.80315	-914.54914	27.1
		Dewar	-914.84821	-914.59228	0.0
	Xyl	Aza	-954.26063	-953.97675	-52.6
		TS	-954.12785	-953.84791	28.3
		Dewar	-954.17284	-953.89297	0.0
	Mes	Aza	-993.58794	-993.27995	-51.9
		TS	-993.45538	-993.15114	29.0
		Dewar	-993.50025	-993.19731	0.0
	Ph*	Aza	-1072.23033	-1071.86668	-50.8
		TS	-1072.09681	-1071.73987	28.8
		Dewar	-1072.14247	-1071.78575	0.0
	p-OMe	Aza	-1068.81725	-1068.50276	-51.1
		TS	-1068.68468	-1068.37547	28.8
		Dewar	-1068.72911	-1068.42129	0.0
	p-CN	Aza	-1046.53007	-1046.24943	-52.0
		TS	-1046.39669	-1046.12049	28.9
		Dewar	-1046.44180	-1046.16650	0.0

Table S12. Total energy E and Gibbs energy (G, T = 298.15 K, p = 1 atm) of the optimized structures that occur during the thermal ring opening of N-TBS-B-X substituted ^{BN}**D** (X see below). Relative Gibbs energies (G_{rel}) are given with respect to the corresponding Dewar isomers.

N	B		E	G	G_{rel}
	X		Hartree/particle	Hartree/particle	kcal/mol
TBS	Ph	Aza	-993.57018	-993.25551	-51.4
		TS	-993.44027	-993.13210	26.0
		Dewar	-993.48451	-993.17358	0.0
	o-Tol	Aza	-1032.89598	-1032.55559	-52.7
		TS	-1032.76433	-1032.43014	26.1
		Dewar	-1032.80808	-1032.47166	0.0
	Xyl	Aza	-1072.21895	-1071.85370	-51.1
		TS	-1072.08920	-1071.72941	26.9
		Dewar	-1072.13323	-1071.77227	0.0
	Mes [a]	Aza			50.8
		TS			26.7
		Dewar			0.0
	Ph*	Aza	-1190.18487	-1189.74226	-48.6
		TS	-1190.05816	-1189.62194	26.9
		Dewar	-1190.10292	-1189.66473	0.0
	p-OMe	Aza	-1186.77531	-1186.38093	-50.3
		TS	-1186.64594	-1186.25767	27.1
		Dewar	-1186.68951	-1186.30081	0.0
	p-CN	Aza	-1164.48851	-1164.12731	-51.5
		TS	-1164.35818	-1164.00244	26.8
		Dewar	-1164.40219	-1164.04519	0.0

[a] Values for N-TBS-B-Mes were taken from Edel *et al.*[11]

Table S13. Total energy E and Gibbs energy (G, T = 298.15 K, p = 1 atm) of the optimized structures that occur during the thermal ring opening of N-hexyl-B-mesityl substituted ^{BN}**D**. Relative Gibbs energies (G_{rel}) are given with respect to the corresponding Dewar isomer.

N	B		E	G	G_{rel}
			Hartree/particle	Hartree/particle	kcal/mol
Hex	Mes	Aza	-820.78066	-820.40728	-58.3
		TS	-820.64254	-820.27309	25.9
		Dewar	-820.68406	-820.31433	0.0

7.1 Cartesian coordinates

All coordinates are given in Å.

```
----------------------------------------
22
N-H-B-Ph-aza | SCF Done:  E(RB3LYP) =  -466.867926523     A.U. after    1 cycles
C       -2.862632      -1.179932      -0.291653
C       -1.619714       1.292314       0.306654
B       -0.749449       0.081485       0.019367
N       -1.498328      -1.117237      -0.274360
H       -3.309033      -2.137623      -0.533150
H       -1.207785       2.266271       0.555263
C       -2.986888       1.163751       0.280287
C       -3.619385      -0.076171      -0.020067
H       -3.627312       2.016426       0.494159
H       -4.698565      -0.156450      -0.038631
C        0.819884       0.031933       0.017288
C        1.537425      -1.141468       0.312778
C        1.573071       1.180468      -0.285508
C        2.930346      -1.172591       0.302144
C        2.965801       1.158095      -0.306123
C        3.650044      -0.020586      -0.011758
H        3.454364      -2.091274       0.543392
H        3.518327       2.058973      -0.550840
H       -1.018782      -1.975348      -0.511201
H        1.058582       2.106694      -0.520128
H        1.003744      -2.048925       0.583178
H        4.734297      -0.040377      -0.022612

----------------------------------------
22
N-H-B-Ph-TS | SCF Done:  E(RB3LYP) =  -466.730887400     A.U. after    1 cycles
C       -1.714123       1.206753      -0.475577
B       -0.809913       0.075623      -0.237955
N       -1.470950      -1.407124       0.063031
C       -2.373233      -0.821200       0.832639
C       -3.522369      -0.167231       0.159735
C       -3.141623       0.966619      -0.464034
H       -2.290358      -0.843174       1.924630
H       -1.342208       2.191854      -0.740019
H       -3.840286       1.591324      -1.014261
H       -4.449674      -0.712166       0.023392
C        0.749318       0.039079      -0.090718
C        1.449436       1.174239       0.360266
C        1.513626      -1.083467      -0.459171
C        2.839499       1.187079       0.438898
C        2.905040      -1.075899      -0.385723
C        3.573274       0.060407       0.067222
H        3.467631      -1.953423      -0.685851
H        3.352013       2.074987       0.793455
H       -0.789014      -1.946196       0.594198
H        4.655868       0.068488       0.129741
H        0.891630       2.055260       0.658199
H        1.017540      -1.973477      -0.836150

----------------------------------------
22
N-H-B-Ph-Dewar | SCF Done:  E(RB3LYP) =  -466.766998020     A.U. after    1 cycles
C        2.794259      -0.589568      -0.460810
C        1.944512       0.731586      -0.613752
B        0.704976      -0.257404      -0.280198
N        1.568859      -1.367562      -0.186038
H        3.414130      -0.964376      -1.277265
H        1.982886       1.307657      -1.539446
C        2.761502       1.220187       0.576933
C        3.464113       0.089023       0.729433
H        2.727589       2.151105       1.132739
H        4.178258      -0.260421       1.465724
```

```
C       -0.831438      -0.110363      -0.120686
C       -1.435582       1.155337      -0.212716
C       -1.668749      -1.215156       0.120691
C       -2.811886       1.316173      -0.065007
C       -3.044347      -1.064023       0.267147
C       -3.618572       0.205017       0.175314
H       -3.255636       2.303114      -0.138533
H       -3.670768      -1.930021       0.451895
H        1.535739      -2.317963       0.151145
H       -4.690490       0.325405       0.289028
H       -1.236898      -2.208786       0.191641
H       -0.814565       2.024973      -0.402958

----------------------------------------
25
N-H-B-o-Tol-aza | SCF Done:  E(RB3LYP) =  -506.191137148     A.U. after    1 cycles
C        2.949072       0.913421      -0.666297
C        1.717661      -1.268495       0.645766
B        0.838692      -0.201477       0.014872
N        1.584591       0.857904      -0.621645
H        3.390555       1.757456      -1.183490
H        1.305837      -2.124980       1.172102
C        3.084578      -1.151810       0.577510
C        3.711477      -0.057607      -0.082955
H        3.728115      -1.900934       1.033273
H        4.790273       0.016243      -0.130275
C       -0.736498      -0.220258      -0.018371
C       -1.572359       0.896706       0.222047
C       -1.356538      -1.451035      -0.305363
C       -2.962168       0.744029       0.159030
C       -2.739402      -1.589261      -0.380470
C       -3.548782      -0.481076      -0.146595
H       -3.596939       1.601796       0.360414
H       -3.180166      -2.552695      -0.612279
H        1.103826       1.610532      -1.094097
H       -0.733113      -2.321906      -0.482277
H       -4.628905      -0.569398      -0.190868
C       -1.017970       2.264196       0.568272
H       -0.118333       2.199503       1.183381
H       -0.763867       2.840682      -0.330162
H       -1.757313       2.852898       1.115981

----------------------------------------
25
N-H-B-o-Tol-TS | SCF Done:  E(RB3LYP) =  -506.055066560     A.U. after    1 cycles
C        1.838327      -0.810275      -1.086210
B        0.894220      -0.080884      -0.237244
N        1.509697       0.951066       0.913181
C        2.423225       0.039376       1.196954
C        3.595873      -0.049753       0.291623
C        3.257531      -0.584083      -0.899197
H        2.335153      -0.603374       2.079461
H        1.500010      -1.455661      -1.890981
H        3.979897      -0.729783      -1.698024
H        4.508299       0.482206       0.536489
C       -0.664638      -0.212290      -0.083154
C       -1.225323      -1.480498       0.153169
C       -1.540013       0.893427      -0.212904
C       -2.600302      -1.666594       0.273455
C       -2.919233       0.691773      -0.094580
C       -3.451621      -0.571600       0.152570
H       -3.588348       1.539229      -0.208421
H       -3.002335      -2.657425       0.454607
C       -1.019892       2.284450      -0.503231
H       -0.220444       2.266707      -1.247488
H       -1.819610       2.927730      -0.876313
H       -0.607922       2.763399       0.391799
H        0.802254       1.045553       1.640725
H       -4.525207      -0.699064       0.239982
```

```
H       -0.564341      -2.336163       0.241136

----------------------------------------
25
N-H-B-o-Tol-Dewar | SCF Done:  E(RB3LYP) =  -506.091392922     A.U. after    1
cycles
C       -2.849402      -0.823315       0.397783
C       -2.032019       0.494254       0.680914
B       -0.766864      -0.416241       0.229161
N       -1.609425      -1.530754       0.024952
H       -3.446981      -1.300578       1.176724
H       -2.068845       0.965478       1.664350
C       -2.882699       1.088616      -0.436107
C       -3.558074      -0.038231      -0.701348
H       -2.886022       2.076240      -0.884863
H       -4.278683      -0.323539      -1.458681
C        0.776613      -0.285852       0.081581
C        1.458640       0.954711       0.039883
C        1.540426      -1.464839      -0.003849
C        2.851182       0.965237      -0.087989
C        2.925921      -1.443601      -0.126646
C        3.583894      -0.217054      -0.169966
H        3.371884       1.917030      -0.123265
H        3.486047      -2.370246      -0.185661
C        0.719875       2.272355       0.115258
H        0.030664       2.394397      -0.725007
H        0.126644       2.349205       1.030010
H        1.419367       3.110655       0.096129
H       -1.556945      -2.434014      -0.422044
H        4.663726      -0.177975      -0.265310
H        1.033295      -2.423861       0.040056

----------------------------------------
28
N-H-B-Xylyl-aza | SCF Done:  E(RB3LYP) =  -545.515971745     A.U. after    1 cycles
C       -2.971007      -0.000043      -1.231575
C       -1.747240       0.000020       1.322371
B       -0.866534      -0.000071       0.085486
N       -1.606280      -0.000058      -1.153144
H       -3.408607      -0.000052      -2.223167
H       -1.338187       0.000066       2.328793
C       -3.114064       0.000018       1.179746
C       -3.736276      -0.000020      -0.100729
H       -3.761778       0.000057       2.053663
H       -4.814875      -0.000010      -0.191114
C        0.714724      -0.000018       0.034771
C        1.431294      -1.217288       0.019003
C        1.431213       1.217300       0.018984
C        2.829140      -1.202766      -0.017280
C        2.829059       1.202873      -0.017301
C        3.527550       0.000077      -0.036927
H        3.372636      -2.142379      -0.025865
H        3.372493       2.142523      -0.025908
H       -1.113036      -0.000067      -2.036179
H        4.612009       0.000113      -0.062269
C        0.703201      -2.544935       0.042851
H        0.003010      -2.603835       0.881340
H        0.118110      -2.701866      -0.869517
H        1.405313      -3.376690       0.131408
C        0.703025       2.544895       0.042805
H        0.117841       2.701719      -0.869522
H        0.002904       2.603798       0.881352
H        1.405083       3.376708       0.131245

----------------------------------------
28
N-H-B-Xylyl-TS | SCF Done:  E(RB3LYP) =  -545.379406045     A.U. after    1 cycles
C        1.867656      -0.147510      -1.285193
B        0.916475       0.152251      -0.215416
```

```
N        1.526316       0.655199       1.259413
C        2.447985      -0.285495       1.154737
C        3.617904       0.011059       0.290808
C        3.283573       0.000916      -1.015652
H        2.370504      -1.229721       1.704074
H        1.539144      -0.413058      -2.285573
H        4.006062       0.203093      -1.802022
H        4.522061       0.417495       0.730104
C       -0.649913       0.018477      -0.104283
C       -1.294831      -1.239157      -0.040521
C       -1.435130       1.197476      -0.087402
C       -2.690064      -1.297980       0.041414
C       -2.827198       1.103421      -0.008102
C       -3.454135      -0.136402       0.059725
H       -3.423008       2.010545      -0.005150
H       -3.179931      -2.265287       0.088031
C       -0.510616      -2.533007      -0.054742
H        0.173567      -2.579360      -0.904442
H        0.102553      -2.645275       0.846696
H       -1.181729      -3.393202      -0.102741
C       -0.791636       2.563598      -0.189156
H       -0.078081       2.741511       0.620164
H       -0.234685       2.667488      -1.125131
H       -1.545224       3.352996      -0.155478
H        0.816025       0.449986       1.961272
H       -4.535417      -0.197244       0.121587

----------------------------------------
28
N-H-B-Xylyl-Dewar | SCF Done:  E(RB3LYP) =  -545.413118771     A.U. after    1
cycles
C        2.886730      -0.669576       0.497720
C        2.043032      -0.614689      -0.832792
B        0.807166      -0.316714       0.186251
N        1.669518      -0.401535       1.292279
H        3.445317      -1.562837       0.782041
H        2.021318      -1.466141      -1.514168
C        2.941251       0.564845      -1.182775
C        3.642543       0.540667      -0.040272
H        2.956599       1.218907      -2.047984
H        4.405085       1.179698       0.388956
C       -0.733003      -0.054912       0.076721
C       -1.243759       1.261464       0.086235
C       -1.626002      -1.141059      -0.053911
C       -2.620869       1.470828      -0.029806
C       -2.998343      -0.902097      -0.164525
C       -3.496610       0.396865      -0.153145
H       -3.008093       2.484837      -0.024642
H       -3.679902      -1.741188      -0.262237
C       -0.317345       2.451291       0.220151
H        0.191844       2.457306       1.189100
H        0.461943       2.445140      -0.547818
H       -0.867725       3.389931       0.128234
C       -1.110495      -2.564023      -0.070463
H       -0.407283      -2.725144      -0.894060
H       -0.582808      -2.811217       0.856143
H       -1.927693      -3.278472      -0.188864
H        1.629497      -0.205854       2.281954
H       -4.563343       0.571731      -0.242698

----------------------------------------
31
N-H-B-Mes-aza | SCF Done:  E(RB3LYP) =  -584.846158738     A.U. after    1 cycles
C       -3.369149      -0.000565      -1.242402
C       -2.154858       0.000603       1.315902
B       -1.269670       0.000013       0.081755
N       -2.005349      -0.000539      -1.159260
H       -3.802132      -0.001026      -2.235784
H       -1.750037       0.001052       2.324309
```

```
C       -3.522147       0.000556       1.168862
C       -4.139504      -0.000033      -0.113754
H       -4.172971       0.000967       2.040561
H       -5.217791      -0.000071      -0.208335
C        0.312111      -0.000029       0.040509
C        1.034660      -1.213023       0.032281
C        1.034597       1.213002       0.031916
C        2.432686      -1.195809       0.009293
C        2.432632       1.195847       0.008923
C        3.152744       0.000037      -0.007043
H        2.972019      -2.139267       0.009963
H        2.971914       2.139335       0.009292
C        0.312551      -2.544279       0.053234
H       -0.381177      -2.610592       0.896692
H       -0.279247      -2.696137      -0.855489
H        1.018149      -3.374128       0.131271
C        4.662156       0.000058      -0.063504
H        5.079473      -0.884793       0.423302
H        5.017213       0.000081      -1.100278
H        5.079454       0.884904       0.423330
C        0.312444       2.544237       0.052460
H       -0.279560       2.695684      -0.856198
H       -0.381111       2.610883       0.896033
H        1.018031       3.374141       0.129995
H       -1.511970      -0.000931      -2.042680

----------------------------------------
31
N-H-B-Mes-TS | SCF Done:  E(RB3LYP) =  -584.707073297     A.U. after    1 cycles
C        2.276747      -0.189121      -1.270350
B        1.315822       0.144170      -0.218943
N        1.919673       0.661421       1.257785
C        2.825032      -0.297092       1.178570
C        4.010212      -0.034497       0.324861
C        3.691355      -0.061050      -0.985466
H        2.722232      -1.233075       1.737821
H        1.956520      -0.463454      -2.271130
H        4.426774       0.115624      -1.766049
H        4.915125       0.366337       0.767754
C       -0.251743       0.039074      -0.121440
C       -0.924300      -1.202950      -0.062215
C       -1.022378       1.227149      -0.117465
C       -2.320598      -1.234729       0.003263
C       -2.415234       1.154246      -0.054762
C       -3.085539      -0.068384       0.012502
H       -2.991752       2.075120      -0.067150
H       -2.823427      -2.196977       0.042486
C       -0.168027      -2.513563      -0.067178
H        0.519829      -2.577566      -0.912819
H        0.438190      -2.634336       0.837786
H       -0.856499      -3.359942      -0.115505
C       -0.360396       2.584481      -0.220771
H        0.208513       2.674606      -1.151007
H       -1.103497       3.384239      -0.200607
H        0.346670       2.758015       0.595172
C       -4.590767      -0.124236       0.113926
H       -5.058432       0.694660      -0.438378
H       -4.982380      -1.065865      -0.277924
H       -4.917056      -0.042038       1.156831
H        1.196174       0.477894       1.952106

----------------------------------------
31
N-H-B-Mes-Dewar | SCF Done:  E(RB3LYP) =  -584.743009579     A.U. after    1 cycles
C       -3.343525       0.583342      -0.425831
C       -2.497491      -0.735271      -0.560087
B       -1.238322       0.252687      -0.260591
N       -2.116557       1.362104      -0.186167
H       -3.975430       0.941106      -1.241167
```

H	-2.549112	-1.328583	-1.474069
C	-3.301049	-1.204064	0.648147
C	-4.001963	-0.069840	0.785586
H	-3.263928	-2.124094	1.222049
H	-4.711352	0.292304	1.520486
C	0.307090	0.111899	-0.122761
C	1.152759	1.247541	-0.007120
C	0.921378	-1.169333	-0.125887
C	2.536227	1.090981	0.092604
C	2.308494	-1.283847	-0.017599
C	3.136943	-0.167363	0.093316
H	3.162026	1.975401	0.171676
H	2.755908	-2.273659	-0.022619
C	0.604870	2.657217	0.013043
H	-0.019053	2.861541	-0.861022
H	-0.005466	2.833897	0.905105
H	1.416638	3.386893	0.024522
C	0.122688	-2.450857	-0.243437
H	-0.627692	-2.537067	0.545268
H	-0.405557	-2.512354	-1.198196
H	0.782320	-3.318066	-0.172057
C	4.631117	-0.317676	0.238583
H	4.913122	-0.405921	1.293834
H	4.993641	-1.213270	-0.271628
H	5.160890	0.546291	-0.169619
H	-2.084677	2.321050	0.122218

37

N-H-B-pentamethylphenyl-aza | SCF Done: E(RB3LYP) = -663.484818275   A.U. after 1 cycles

C	3.775167	-0.000108	1.268136
C	2.601152	0.000039	-1.308349
B	1.692922	0.000022	-0.089406
N	2.412254	-0.000065	1.162752
H	4.193221	-0.000167	2.268205
H	2.210983	0.000090	-2.322233
C	3.964544	-0.000004	-1.139897
C	4.562770	-0.000076	0.152837
H	4.629115	0.000013	-2.001199
H	5.639451	-0.000111	0.263975
C	0.109390	0.000037	-0.062327
C	-0.601300	-1.217861	-0.054719
C	-0.601303	1.217933	-0.054644
C	-2.007468	-1.219762	-0.015129
C	-2.007492	1.219877	-0.015070
C	-2.705959	0.000051	0.035447
H	1.900371	-0.000094	2.035117
C	0.155525	-2.533674	-0.089352
H	-0.062334	-3.102640	-0.999699
H	1.234107	-2.377246	-0.063870
H	-0.102413	-3.176198	0.758868
C	0.155581	2.533720	-0.089228
H	1.234155	2.377233	-0.063762
H	-0.062247	3.102736	-0.999550
H	-0.102306	3.176220	0.759024
C	-4.218175	-0.000218	0.139564
H	-4.694136	-0.002735	-0.849586
H	-4.586071	-0.876723	0.673706
H	-4.586852	0.878314	0.669724
C	-2.779434	-2.524693	-0.023583
H	-2.165057	-3.361136	-0.351832
H	-3.161521	-2.777331	0.973438
H	-3.642285	-2.475966	-0.692164
C	-2.779349	2.524897	-0.023615
H	-3.162166	2.777225	0.973196
H	-2.164634	3.361388	-0.351090
H	-3.641662	2.476521	-0.692918

37
N-H-B-pentamethylphenyl-TS | SCF Done: E(RB3LYP) = -663.348174650   A.U. after 1 cycles

C	-2.722126	0.096279	-1.267358
B	-1.734976	-0.149278	-0.213840
N	-2.310547	-0.572156	1.306510
C	-3.229104	0.368248	1.173508
C	-4.425737	0.027093	0.365292
C	-4.129046	-0.030917	-0.949968
H	-3.126069	1.346763	1.653933
H	-2.421625	0.305252	-2.289860
H	-4.873777	-0.273140	-1.703830
H	-5.310815	-0.368773	0.850833
C	-0.162244	-0.037556	-0.144363
C	0.509978	1.205012	-0.115654
C	0.587847	-1.236359	-0.117594
C	1.914165	1.245285	-0.030568
C	1.990919	-1.193621	-0.043456
C	2.647908	0.047454	0.038606
C	-0.270270	2.504274	-0.170048
H	-1.341824	2.319958	-0.217452
H	-0.067331	3.138482	0.699841
H	-0.011290	3.090708	-1.057981
C	-0.120426	-2.576657	-0.190647
H	-1.203405	-2.462690	-0.180173
H	0.137627	-3.112396	-1.110468
H	0.151983	-3.226799	0.646731
H	-1.575418	-0.333479	1.971051
C	4.154444	0.095439	0.195364
H	4.662309	0.142504	-0.776546
H	4.472647	0.969042	0.765054
H	4.535121	-0.784507	0.713867
C	2.649576	2.571151	-0.009064
H	2.030403	3.389012	-0.372363
H	2.973892	2.838608	1.004488
H	3.545765	2.542725	-0.633005
C	2.803848	-2.473435	-0.049657
H	3.150928	-2.739376	0.956580
H	2.229817	-3.319050	-0.423610
H	3.691369	-2.381502	-0.679823

37
N-H-B-pentamethylphenyl-Dewar | SCF Done: E(RB3LYP) = -663.382599645   A.U. after   1 cycles

C	-3.744318	-0.644071	-0.417205
C	-2.866847	-0.537639	0.888418
B	-1.642911	-0.352897	-0.174597
N	-2.538545	-0.471310	-1.253178
H	-4.342156	-1.531610	-0.632086
H	-2.856070	-1.344251	1.622952
C	-3.713745	0.693218	1.183440
C	-4.443591	0.624415	0.060512
H	-3.682810	1.401377	2.004591
H	-5.191787	1.264945	-0.391491
C	-0.089369	-0.154002	-0.114108
C	0.757218	-1.276647	-0.026783
C	0.465251	1.140718	-0.129122
C	2.150320	-1.107907	0.051340
C	1.858344	1.313436	-0.051612
C	2.695815	0.189048	0.065836
C	0.155680	-2.669700	-0.009469
H	-0.934756	-2.635954	0.001359
H	0.444660	-3.250484	-0.892238
H	0.472517	-3.239551	0.869484
C	-0.449190	2.348564	-0.214819
H	-0.293982	2.913479	-1.140769
H	-1.500780	2.060319	-0.191115

```
H       -0.283915       3.042415       0.615313
H       -2.515017      -0.335376      -2.253340
C        4.193120       0.375559       0.207978
H        4.647686      -0.430526       0.784441
H        4.694758       0.396201      -0.768201
H        4.437537       1.310305       0.713225
C        2.466190       2.701685      -0.089213
H        1.764586       3.444445      -0.465035
H        2.783337       3.033164       0.907349
H        3.348608       2.736143      -0.732642
C        3.070578      -2.310518       0.122110
H        3.451651      -2.470983       1.138256
H        2.567906      -3.227856      -0.179613
H        3.939654      -2.189499      -0.529024

------------------------------------------
32
N-H-B-p-OMe-aza | SCF Done:  E(RB3LYP) =  -660.072286261     A.U. after    1 cycles
C        3.724212      -0.149571      -1.242471
C        2.516660      -0.079597       1.317535
B        1.628729      -0.020574       0.086217
N        2.362855      -0.063870      -1.156168
H        4.155793      -0.174049      -2.236467
H        2.113785      -0.056031       2.326268
C        3.880046      -0.165455       1.167895
C        4.494276      -0.201197      -0.115947
H        4.531189      -0.208321       2.038295
H        5.570266      -0.268755      -0.212371
C        0.052055       0.080173       0.043326
C       -0.593748       1.340408       0.029503
C       -0.746837      -1.078152       0.035857
C       -1.982606       1.420805       0.002784
C       -2.145689      -0.985055       0.009680
C       -2.763718       0.263289      -0.008576
H       -2.485554       2.381367      -0.003559
H       -2.731790      -1.895108       0.009072
H        1.864996      -0.029950      -2.035964
C        0.214650       2.620615       0.047371
H        0.912644       2.639533       0.889567
H        0.813822       2.730581      -0.862544
H       -0.433351       3.495832       0.125480
C       -0.114563      -2.454917       0.050351
H        0.385994      -2.676650      -0.898398
H        0.641920      -2.540270       0.835334
H       -0.862575      -3.233178       0.216803
O       -4.118309       0.458444      -0.032861
C       -4.967724      -0.678660      -0.032196
H       -4.827720      -1.284162       0.870268
H       -5.985262      -0.290697      -0.050419
H       -4.803403      -1.303840      -0.917066

------------------------------------------
32
N-H-B-p-OMe-TS | SCF Done:  E(RB3LYP) =  -659.936413275     A.U. after    1 cycles
C       -2.618779      -0.325230       1.259912
B       -1.678767       0.106286       0.224527
N       -2.318884       0.614437      -1.245575
C       -3.147238      -0.413040      -1.192374
C       -4.352464      -0.259987      -0.340686
C       -4.037642      -0.297146       0.970605
H       -2.967429      -1.328650      -1.765664
H       -2.283434      -0.596666       2.256698
H       -4.786832      -0.195020       1.751441
H       -5.281356       0.089548      -0.777393
C       -0.110071       0.123183       0.132125
C        0.656955      -1.058973       0.073968
C        0.571412       1.369958       0.128138
C        2.055824      -0.998177       0.009480
C        1.959417       1.411569       0.067314
```

C	2.707407	0.233470	0.004320
H	2.618012	-1.921976	-0.032072
H	2.488985	2.357509	0.077200
C	0.005005	-2.425023	0.070816
H	-0.574742	-2.592168	-0.843874
H	0.754369	-3.217054	0.133467
H	-0.690174	-2.538873	0.904723
C	-0.193496	2.672156	0.225869
H	0.487200	3.525226	0.247019
H	-0.879607	2.805617	-0.615116
H	-0.803486	2.700667	1.133465
O	4.062856	0.391992	-0.054105
C	4.883660	-0.766315	-0.102280
H	5.909408	-0.402479	-0.138343
H	4.751275	-1.388573	0.789512
H	4.680525	-1.365131	-0.996952
H	-1.579469	0.500915	-1.938118

32

N-H-B-p-OMe-Dewar | SCF Done: E(RB3LYP) = -659.972895406   A.U. after   1 cycles

C	-3.684063	0.691218	-0.414073
C	-2.902424	-0.668460	-0.526665
B	-1.593349	0.265465	-0.267769
N	-2.419358	1.417752	-0.211173
H	-4.311387	1.057574	-1.229267
H	-2.995552	-1.280907	-1.424549
C	-3.708445	-1.069374	0.703988
C	-4.353946	0.099430	0.822468
H	-3.704797	-1.975440	1.300943
H	-5.034042	0.512308	1.558403
C	-0.058668	0.052354	-0.145953
C	0.841266	1.142945	-0.045525
C	0.498741	-1.260270	-0.144266
C	2.221949	0.936471	0.044841
C	1.872026	-1.445823	-0.047728
C	2.740646	-0.356577	0.044638
H	2.876674	1.795089	0.116076
H	2.299066	-2.442064	-0.042575
C	0.360268	2.577205	-0.027319
H	-0.260100	2.804154	-0.898121
H	-0.235597	2.783754	0.867808
H	1.203202	3.270772	-0.025337
C	-0.361706	-2.502431	-0.242278
H	-1.106904	-2.543194	0.554844
H	-0.902459	-2.543166	-1.190950
H	0.255187	-3.400169	-0.170733
H	-2.341192	2.379515	0.079443
O	4.067080	-0.658185	0.130692
C	5.006083	0.404572	0.229374
H	4.839888	1.002481	1.131815
H	5.984873	-0.069032	0.285973
H	4.968378	1.054655	-0.651251

29

N-H-B-p-CN-aza | SCF Done: E(RB3LYP) = -637.785120032   A.U. after   1 cycles

C	-3.582633	-0.000003	-1.242066
C	-2.364406	0.000014	1.315767
B	-1.486162	-0.000041	0.080271
N	-2.217498	-0.000023	-1.161030
H	-4.017565	-0.000001	-2.234587
H	-1.958092	0.000036	2.323107
C	-3.731322	0.000026	1.169001
C	-4.349762	0.000013	-0.112564
H	-4.380755	0.000052	2.041308
H	-5.428034	0.000029	-0.205341
C	0.097760	-0.000021	0.040103

```
C        0.812642      -1.219013       0.031776
C        0.812613       1.218988       0.031762
C        2.206878      -1.210137       0.008236
C        2.206849       1.210147       0.008222
C        2.908437       0.000013      -0.005719
H        2.757700      -2.143552       0.003666
H        2.757648       2.143575       0.003641
H       -1.724549      -0.000032      -2.044281
C        0.083977      -2.545400       0.050477
H       -0.614567      -2.603838       0.889833
H       -0.502231      -2.694111      -0.861862
H        0.783921      -3.378764       0.134316
C        0.083912       2.545355       0.050459
H       -0.502382       2.694003      -0.861835
H       -0.614559       2.603813       0.889875
H        0.783838       3.378744       0.134197
C        4.339363       0.000030      -0.030998
N        5.495296       0.000049      -0.053273

----------------------------------------
29
N-H-B-p-CN-TS | SCF Done:  E(RB3LYP) =  -637.647653656     A.U. after    1 cycles
C        2.476311      -0.199412      -1.265338
B        1.535038       0.140856      -0.200457
N        2.139990       0.652999       1.259535
C        3.042047      -0.310680       1.176050
C        4.222702      -0.054680       0.313073
C        3.895863      -0.076564      -0.993842
H        2.946616      -1.237702       1.750973
H        2.144032      -0.480658      -2.259942
H        4.625086       0.097284      -1.780232
H        5.137171       0.325895       0.754015
C       -0.039094       0.043893      -0.114270
C       -0.714784      -1.198232      -0.060032
C       -0.793658       1.243261      -0.118252
C       -2.108527      -1.227674      -0.004012
C       -2.185555       1.190191      -0.065617
C       -2.847206      -0.040503      -0.003975
H       -2.765326       2.105698      -0.077210
H       -2.630288      -2.176540       0.037795
C        0.036433      -2.510811      -0.057623
H        0.717134      -2.582732      -0.907937
H        0.648016      -2.622592       0.844076
H       -0.654510      -3.355131      -0.093599
C       -0.114477       2.591868      -0.209832
H        0.547043       2.772216       0.641844
H        0.505825       2.657307      -1.108035
H       -0.849010       3.398278      -0.244262
H        1.428100       0.479655       1.968437
C       -4.276544      -0.085190       0.055815
N       -5.430868      -0.121294       0.106678

----------------------------------------
29
N-H-B-p-CN-Dewar | SCF Done:  E(RB3LYP) =  -637.682585819     A.U. after    1
cycles
C        3.560450       0.612501       0.355675
C        2.697751      -0.665356       0.669204
B        1.459383       0.281630       0.210413
N        2.343748       1.357389      -0.018484
H        4.179851       1.084490       1.120273
H        2.726940      -1.115373       1.662755
C        3.519301      -1.314234      -0.440304
C        4.232688      -0.218008      -0.731936
H        3.487680      -2.311253      -0.866210
H        4.963136       0.025190      -1.494146
C       -0.096017       0.147118       0.092108
C       -0.934588       1.291546       0.019864
C       -0.702470      -1.137714       0.083389
```

```
C       -2.319769       1.145732      -0.040992
C       -2.089151      -1.258755       0.010725
C       -2.902632      -0.123866      -0.046645
H       -2.956133       2.021457      -0.087716
H       -2.547855      -2.240272       0.000546
C       -0.380912       2.698551      -0.000734
H        0.323191       2.870147       0.816149
H        0.144747       2.900730      -0.939941
H       -1.185081       3.431172       0.084065
C        0.108858      -2.414230       0.135190
H        0.808866      -2.479777      -0.700689
H        0.694747      -2.482558       1.054502
H       -0.546395      -3.285832       0.089716
H        2.322694       2.264617      -0.458130
C       -4.325779      -0.261315      -0.112594
N       -5.475129      -0.372060      -0.165935

---------------------------------------
25
N-Me-B-Ph-aza | SCF Done:  E(RB3LYP) =  -506.178776715     A.U. after    1 cycles
C       -2.884432       0.764372       0.165480
C       -1.395786      -1.560012      -0.350836
B       -0.664484      -0.264083      -0.050878
N       -1.520332       0.877219       0.220032
H       -3.447845       1.665741       0.381845
H       -0.860338      -2.481404      -0.562305
C       -2.767127      -1.583317      -0.387744
C       -3.520732      -0.407487      -0.130829
H       -3.307864      -2.499146      -0.614587
H       -4.602935      -0.423004      -0.155585
C        0.908811      -0.156280      -0.033982
C        1.622809       0.791454      -0.789207
C        1.665216      -1.081171       0.708292
C        3.016501       0.820173      -0.800335
C        3.058712      -1.049809       0.715989
C        3.740080      -0.097825      -0.040757
H        3.537703       1.556766      -1.402673
H        3.612850      -1.770840       1.307587
H        1.152370      -1.837225       1.294858
H        1.083300       1.511167      -1.397600
H        4.824353      -0.074837      -0.043048
C       -1.009186       2.207740       0.586367
H        0.020366       2.126214       0.924594
H       -1.044818       2.890567      -0.267756
H       -1.611597       2.628850       1.394202

---------------------------------------
25
N-Me-B-Ph-TS | SCF Done:  E(RB3LYP) =  -506.044099130     A.U. after    1 cycles
C        1.558308      -1.532070      -0.437827
B        0.748319      -0.320060      -0.270777
N        1.503498       1.153980      -0.133038
C        2.366503       0.565658       0.674608
C        3.453655      -0.244286       0.067012
C        2.996365      -1.400404      -0.454227
H        2.325441       0.755705       1.756505
H        1.099225      -2.502854      -0.597139
H        3.645408      -2.117086      -0.950011
H        4.415158       0.222226      -0.119755
C       -0.815114      -0.225936      -0.114088
C       -1.485988      -0.852244       0.949754
C       -1.602755       0.411939      -1.089232
C       -2.877474      -0.834979       1.042344
C       -2.993971       0.425625      -1.007150
C       -3.636001      -0.194665       0.064007
H       -3.576796       0.915883      -1.779682
H       -3.369290      -1.326595       1.875012
H       -4.718307      -0.183566       0.132108
H       -0.909364      -1.365171       1.712155
```

```
H       -1.121124       0.888410      -1.938146
C        0.745508       2.291535       0.403889
H       -0.172445       2.430976      -0.160026
H        0.496531       2.159337       1.464815
H        1.365271       3.184137       0.294779
```

```
25
N-Me-B-Ph-Dewar | SCF Done:  E(RB3LYP) =  -506.085106207     A.U. after    1 cycles
C        2.661604       0.098960       0.527891
C        1.680237      -1.120375       0.692014
B        0.560230      -0.014323       0.311596
N        1.537364       0.995098       0.189806
H        3.295949       0.437736       1.350866
H        1.640176      -1.672375       1.631980
C        2.468403      -1.722131      -0.466045
C        3.289712      -0.674621      -0.628458
H        2.351781      -2.659336      -1.000010
H        4.056252      -0.425825      -1.352739
C       -0.984962      -0.031991       0.141688
C       -1.773313       1.133702       0.110811
C       -1.650733      -1.265023       0.019945
C       -3.155699       1.072041      -0.043408
C       -3.032309      -1.335176      -0.144572
C       -3.788367      -0.164324      -0.176630
H       -3.741585       1.984812      -0.058498
H       -3.520151      -2.299078      -0.241586
H       -4.864925      -0.214092      -0.298767
H       -1.072119      -2.182713       0.054854
H       -1.302566       2.104349       0.224046
C        1.687415       2.334435      -0.329326
H        0.723334       2.720264      -0.662718
H        2.088638       3.015716       0.430295
H        2.370592       2.343482      -1.187804
```

```
28
N-Me-B-o-Tol-aza | SCF Done:  E(RB3LYP) =  -545.504088530     A.U. after    1
cycles
C        2.934588       0.563769      -0.602257
C        1.522167      -1.345734       0.901797
B        0.750072      -0.339700       0.068476
N        1.567468       0.599353      -0.674507
H        3.465682       1.302407      -1.193523
H        1.017954      -2.102722       1.495796
C        2.894339      -1.311452       0.919150
C        3.609203      -0.345806       0.161627
H        3.466034      -2.022554       1.510993
H        4.691428      -0.319221       0.173831
C       -0.829031      -0.304747      -0.031592
C       -1.623221       0.587952       0.723551
C       -1.481637      -1.224512      -0.867957
C       -3.016327       0.538317       0.608907
C       -2.871045      -1.263511      -0.980119
C       -3.642511      -0.374727      -0.237666
H       -3.620893       1.223037       1.196091
H       -3.344551      -1.984963      -1.637202
H       -0.889225      -1.929453      -1.444212
H       -4.724607      -0.394119      -0.309341
C       -0.985057       1.590788       1.659994
H       -0.288391       1.105846       2.350088
H       -0.414561       2.349336       1.112929
H       -1.739028       2.112383       2.253466
C        1.002874       1.634674      -1.553853
H       -0.079285       1.535972      -1.582015
H        1.260126       2.632486      -1.187204
H        1.390635       1.524530      -2.570029
```

```
28
N-Me-B-o-Tol-TS | SCF Done:  E(RB3LYP) =  -545.369486891     A.U. after    1 cycles
C        1.646293      -0.850175      -1.341469
B        0.818025       0.012860      -0.494110
N        1.555741       1.136567       0.492974
C        2.411835       0.187722       0.820685
C        3.516156      -0.106592      -0.130341
C        3.082156      -0.728116      -1.245399
H        2.355768      -0.291665       1.808104
H        1.206437      -1.538474      -2.056821
H        3.748907      -1.004654      -2.057700
H        4.471809       0.388396       0.007697
C       -0.751101       0.038248      -0.337485
C       -1.491658      -0.909103       0.405000
C       -1.454412       1.035392      -1.034751
C       -2.888581      -0.823934       0.421424
C       -2.845775       1.107581      -1.014847
C       -3.566423       0.172024      -0.278121
H       -3.359811       1.883298      -1.571824
H       -3.455353      -1.556124       0.988366
H       -4.649995       0.211609      -0.250817
H       -0.897489       1.763704      -1.617077
C        0.770987       1.757628       1.565704
C       -0.808110      -2.010368       1.183031
H       -1.535518      -2.728022       1.568675
H       -0.085618      -2.546285       0.563494
H       -0.253904      -1.612452       2.041231
H        1.363510       2.577752       1.977242
H        0.538598       1.051333       2.373839
H       -0.155849       2.159476       1.164732

----------------------------------------
28
N-Me-B-o-Tol-Dewar | SCF Done:  E(RB3LYP) =  -545.408989568     A.U. after    1
cycles
C       -2.775431      -0.130647      -0.440057
C       -1.778807      -1.248763       0.034228
B       -0.664098      -0.096687      -0.223179
N       -1.656400       0.808702      -0.650955
H       -3.431368      -0.270527      -1.303207
H       -1.751931      -2.208999      -0.481275
C       -2.533785      -1.162028       1.356984
C       -3.371101      -0.190473       0.965500
H       -2.390352      -1.680024       2.299177
H       -4.133706       0.392264       1.468578
C        0.871728       0.095495      -0.026689
C        1.342884       1.323725       0.472586
C        1.820757      -0.920563      -0.296205
C        2.695831       1.558402       0.706551
C        3.177074      -0.669646      -0.071447
C        3.618336       0.554880       0.427801
H        3.024902       2.513583       1.100828
H        3.902044      -1.447977      -0.288600
H        4.676771       0.720009       0.598058
H        0.630584       2.109547       0.701853
C       -1.797183       2.202668      -1.001291
C        1.400035      -2.271626      -0.830634
H        0.779281      -2.809225      -0.108240
H        0.815038      -2.179115      -1.750208
H        2.271149      -2.892217      -1.050766
H       -2.410733       2.315231      -1.902067
H       -0.816079       2.636114      -1.201482
H       -2.272342       2.775314      -0.194016

----------------------------------------
31
N-Me-B-Xylyl-aza | SCF Done:  E(RB3LYP) =  -584.829751672     A.U. after    1
cycles
C       -2.983894       0.000186       0.840435
```

C	-1.554035	-0.000384	-1.580016
B	-0.789135	-0.000078	-0.268095
N	-1.616504	0.000209	0.922525
H	-3.521293	0.000413	1.783046
H	-1.043863	-0.000613	-2.539391
C	-2.926260	-0.000390	-1.573972
C	-3.650456	-0.000103	-0.351555
H	-3.491630	-0.000614	-2.503065
H	-4.733060	-0.000107	-0.346426
C	0.792241	-0.000045	-0.161721
C	1.509320	1.216710	-0.137461
C	1.509355	-1.216770	-0.137092
C	2.906666	1.202681	-0.083752
C	2.906702	-1.202683	-0.083398
C	3.605232	0.000012	-0.055470
H	3.449727	2.142489	-0.067616
H	3.449792	-2.142471	-0.066994
H	4.689273	0.000035	-0.016330
C	0.780200	2.543285	-0.165903
H	0.101767	2.610068	-1.021459
H	0.171220	2.688709	0.733029
H	1.482425	3.377523	-0.226216
C	0.780279	-2.543378	-0.165093
H	0.171814	-2.688797	0.734193
H	0.101372	-2.610229	-1.020264
H	1.482509	-3.377588	-0.225755
C	-1.059296	0.000568	2.283782
H	-1.385806	0.888944	2.831567
H	-1.385977	-0.887415	2.832100
H	0.026611	0.000452	2.229667

31

N-Me-B-Xylyl-TS | SCF Done: E(RB3LYP) = -584.695126367   A.U. after   1 cycles

C	1.702177	-0.043092	-1.561331
B	0.858086	0.134712	-0.375289
N	1.579657	0.500515	1.089647
C	2.467661	-0.431579	0.798535
C	3.566439	-0.072223	-0.135243
C	3.134510	0.053965	-1.406567
H	2.437919	-1.404309	1.308667
H	1.273120	-0.204964	-2.545853
H	3.795089	0.326411	-2.225307
H	4.504944	0.289434	0.271731
C	-0.708261	-0.001776	-0.234836
C	-1.356481	-1.255105	-0.154237
C	-1.489763	1.178037	-0.239893
C	-2.751982	-1.309692	-0.073162
C	-2.882625	1.090494	-0.157599
C	-3.513485	-0.146090	-0.071255
H	-3.475307	1.999780	-0.166084
H	-3.244224	-2.275243	-0.014224
H	-4.594892	-0.202997	-0.007869
C	0.790044	0.337751	2.315841
C	-0.573312	-2.549244	-0.149050
H	0.148470	-2.583504	-0.967887
H	-0.003172	-2.673322	0.779348
H	-1.240686	-3.409235	-0.237698
H	1.354583	0.787647	3.135630
H	0.604135	-0.717732	2.554245
H	-0.162307	0.853031	2.219331
C	-0.841659	2.539983	-0.368959
H	-0.022207	2.675628	0.342333
H	-0.413007	2.676690	-1.366562
H	-1.570065	3.336958	-0.204346

31

```
N-Me-B-Xylyl-Dewar | SCF Done:  E(RB3LYP) =  -584.732978870     A.U. after    1
cycles
C        2.771972      -0.564707      -0.337201
C        1.885753       0.393850      -1.216134
B        0.690269      -0.152370      -0.255231
N        1.593325      -0.956561       0.460713
H        3.331157      -1.393494      -0.778512
H        1.837272       0.266021      -2.298458
C        2.783889       1.492677      -0.661084
C        3.524184       0.684584       0.111696
H        2.777501       2.566650      -0.813663
H        4.310682       0.851726       0.837982
C       -0.858906       0.034228      -0.116424
C       -1.394866       1.296246       0.228003
C       -1.742322      -1.044794      -0.351950
C       -2.777164       1.456749       0.351589
C       -3.121979      -0.850759      -0.233922
C       -3.639993       0.390086       0.121394
H       -3.179081       2.426707       0.626945
H       -3.794849      -1.681143      -0.423225
H       -4.712074       0.526357       0.214523
C        1.615266      -1.744610       1.670182
C       -1.226770      -2.413313      -0.746550
H       -0.531109      -2.357576      -1.588732
H       -0.693029      -2.898868       0.076137
H       -2.050026      -3.069109      -1.037775
H        1.958182      -2.767449       1.474238
H        0.610753      -1.790144       2.094831
H        2.283488      -1.299868       2.417788
C       -0.487471       2.484047       0.462281
H        0.312816       2.253903       1.171734
H       -0.007985       2.803198      -0.468844
H       -1.047880       3.335152       0.854920

----------------------------------------
34
N-Me-B-Mes-aza | SCF Done:  E(RB3LYP) =  -624.157075310     A.U. after    1 cycles
C       -3.361828      -0.000287       0.845173
C       -1.936217       0.000453      -1.577400
B       -1.167866       0.000090      -0.267032
N       -1.994368      -0.000285       0.924902
H       -3.897690      -0.000580       1.788686
H       -1.427644       0.000750      -2.537668
C       -3.308404       0.000427      -1.569283
C       -4.030587       0.000053      -0.345624
H       -3.875390       0.000691      -2.497435
H       -5.113197       0.000026      -0.338585
C        0.412750       0.000094      -0.162602
C        1.135235       1.212414      -0.141600
C        1.135303      -1.212281      -0.142132
C        2.532099       1.195462      -0.094075
C        2.532092      -1.195330      -0.094607
C        3.251644       0.000100      -0.066211
H        3.071241       2.139018      -0.084920
H        3.071247      -2.138894      -0.085878
C        0.411992       2.542361      -0.171171
H       -0.268943       2.609736      -1.024742
H       -0.193821       2.691895       0.729268
H        1.117057       3.373937      -0.235382
C        0.412029      -2.542198      -0.172200
H       -0.193387      -2.692254       0.728423
H       -0.269285      -2.609046      -1.025505
H        1.117054      -3.373745      -0.237209
C       -1.434997      -0.000670       2.285156
H       -1.760561       0.887417       2.834042
H       -1.760699      -0.888984       2.833593
H       -0.349224      -0.000737       2.228381
C        4.759686      -0.000094       0.015946
H        5.097715      -0.008540       1.058388
```

```
H        5.185627      -0.880547      -0.471574
H        5.185027       0.888372      -0.457252

----------------------------------------
34
N-Me-B-Mes-TS | SCF Done:  E(RB3LYP) =  -624.022658705     A.U. after    1 cycles
C        2.081306      -0.084310      -1.556036
B        1.232438       0.124599      -0.378205
N        1.956679       0.499341       1.087904
C        2.828583      -0.452266       0.812780
C        3.937769      -0.124566      -0.119927
C        3.514031      -0.009638      -1.395283
H        2.777287      -1.418959       1.332673
H        1.654796      -0.251229      -2.540891
H        4.183176       0.239825      -2.214454
H        4.879569       0.229324       0.286285
C       -0.334595       0.017714      -0.240229
C       -1.010424      -1.219386      -0.154136
C       -1.100567       1.206519      -0.257618
C       -2.406326      -1.246100      -0.079636
C       -2.493975       1.141037      -0.181815
C       -3.168006      -0.077267      -0.086350
H       -3.066933       2.064039      -0.205495
H       -2.911688      -2.206163      -0.020554
C       -0.255355      -2.530218      -0.138797
H        0.467915      -2.584745      -0.955270
H        0.309951      -2.659613       0.791835
H       -0.940089      -3.376879      -0.223574
C       -0.434021       2.558456      -0.399227
H       -0.003387       2.679537      -1.398049
H       -1.150723       3.367510      -0.242133
H        0.387525       2.688682       0.310680
C       -4.672732      -0.127215       0.028815
H       -5.141353       0.704735      -0.502617
H       -5.073135      -1.058853      -0.378118
H       -4.989061      -0.065101       1.076205
C        1.158140       0.364700       2.311618
H        1.726464       0.814159       3.129126
H        0.215369       0.895112       2.203833
H        0.952398      -0.684525       2.561269

----------------------------------------
34
N-Me-B-Mes-Dewar | SCF Done:  E(RB3LYP) =  -624.060544513     A.U. after    1
cycles
C       -3.180780       0.441677      -0.342263
C       -2.260492      -0.529954      -1.170164
B       -1.080950       0.129494      -0.260869
N       -2.014670       0.934215       0.416382
H       -3.780612       1.215323      -0.828259
H       -2.227875      -0.468074      -2.258938
C       -3.104050      -1.630102      -0.537733
C       -3.872784      -0.808779       0.192220
H       -3.051130      -2.710283      -0.623130
H       -4.643937      -0.964900       0.937232
C        0.475480       0.024761      -0.139502
C        1.086103      -1.209142       0.183582
C        1.305390       1.145634      -0.367995
C        2.474484      -1.296631       0.292181
C        2.693869       1.017759      -0.265859
C        3.299946      -0.193209       0.067046
H        2.923825      -2.250143       0.556318
H        3.317662       1.887919      -0.451222
C       -2.066445       1.786983       1.579968
C        0.728591       2.497581      -0.735409
H       -0.036317       2.417095      -1.512007
H        0.259433       2.985076       0.125138
H        1.510351       3.165662      -1.103343
H       -2.438946       2.787051       1.327717
```

```
H       -1.066603       1.885946       2.006218
H       -2.725152       1.363145       2.347928
C        0.249842      -2.447928       0.419578
H       -0.533325      -2.276282       1.163938
H       -0.248302      -2.768046      -0.501138
H        0.867240      -3.278059       0.769280
C        4.802367      -0.318153       0.148082
H        5.216649      -0.692370      -0.794844
H        5.272540       0.646633       0.352192
H        5.102809      -1.015663       0.934190
```

```
40
N-Me-B-pentamethylphenyl-aza | SCF Done:  E(RB3LYP) =  -702.798525531     A.U.
after    1 cycles
C       -3.770115      -0.000107       0.856609
C       -2.355784       0.000225      -1.571907
B       -1.578631       0.000035      -0.265396
N       -2.402018      -0.000125       0.930200
H       -4.301498      -0.000235       1.802704
H       -1.850765       0.000359      -2.533925
C       -3.727379       0.000232      -1.557959
C       -4.444670       0.000064      -0.330659
H       -4.298563       0.000366      -2.483606
H       -5.527244       0.000071      -0.318600
C        0.004945       0.000021      -0.166185
C        0.716144       1.217216      -0.145084
C        0.716134      -1.217183      -0.145228
C        2.121602       1.219570      -0.083712
C        2.121593      -1.219557      -0.083843
C        2.819747       0.000001      -0.023004
C       -0.040674       2.532855      -0.182627
H        0.179097       3.102442      -1.092226
H       -1.119358       2.375172      -0.161648
H        0.214629       3.175316       0.666429
C       -0.040700      -2.532809      -0.182926
H       -1.119384      -2.375111      -0.161987
H        0.179108      -3.102319      -1.092563
H        0.214548      -3.175348       0.666087
C        4.330208      -0.000003       0.104725
H        4.821807       0.000142      -0.876766
H        4.690115       0.877390       0.642828
H        4.690137      -0.877535       0.642581
C        2.892463       2.525222      -0.080972
H        2.284285       3.359303      -0.426560
H        3.252660       2.783139       0.922873
H        3.769455       2.474545      -0.730614
C        2.892446      -2.525215      -0.081235
H        3.252792      -2.783144       0.922551
H        2.284215      -3.359289      -0.426742
H        3.769342      -2.474530      -0.731008
C       -1.836977      -0.000340       2.288029
H       -0.751726      -0.000239       2.226392
H       -2.160211       0.888180       2.837799
H       -2.160060      -0.889125       2.837459
```

```
40
N-Me-B-pentamethylphenyl-TS | SCF Done:  E(RB3LYP) =  -702.663803798     A.U. after
1 cycles
C       -2.484864       0.022392      -1.566005
B       -1.631493      -0.133654      -0.380492
N       -2.358646      -0.459158       1.097294
C       -3.236507       0.475074       0.781497
C       -4.342859       0.096877      -0.134411
C       -3.915949      -0.060362      -1.405145
H       -3.188911       1.465540       1.254539
H       -2.057400       0.153546      -2.555913
H       -4.580327      -0.349588      -2.215216
```

```
H       -5.275030      -0.266170       0.285899
C       -0.061189      -0.024447      -0.239208
C        0.612458       1.213628      -0.175159
C        0.684858      -1.223981      -0.199115
C        2.014954       1.251270      -0.068233
C        2.088019      -1.185988      -0.113267
C        2.752138       0.053598      -0.073434
C       -1.564282      -0.271253       2.316715
C       -0.163604       2.514814      -0.233810
H       -1.196907       2.340875      -0.529100
H       -0.170917       3.032391       0.733963
H        0.270116       3.208771      -0.959333
H       -2.131920      -0.693007       3.149423
H       -1.366176       0.788316       2.525541
H       -0.618001      -0.799146       2.230353
C       -0.029076      -2.561547      -0.277629
H       -1.098720      -2.435182      -0.442634
H        0.355192      -3.172143      -1.100328
H        0.096040      -3.147347       0.640273
C        2.736376       2.580216       0.045772
H        3.088848       2.938874      -0.929776
H        2.092091       3.356175       0.457426
H        3.609790       2.509464       0.696257
C        4.267253       0.099344      -0.047102
H        4.651752       0.980790      -0.561261
H        4.657685       0.127505       0.978332
H        4.705401      -0.772095      -0.533264
C        2.891706      -2.470999      -0.066959
H        2.276803      -3.327201       0.204212
H        3.349490      -2.698229      -1.037748
H        3.702484      -2.411571       0.662724

----------------------------------------
40
N-Me-B-pentamethylphenyl-Dewar | SCF Done:  E(RB3LYP) =  -702.702374900     A.U.
after    1 cycles
C        3.597433      -0.599225       0.011126
C        2.645357      -0.521236      -1.239138
B        1.490099      -0.335739      -0.105222
N        2.447601      -0.422592       0.920681
H        4.217419      -1.477392       0.208764
H        2.605577      -1.338414      -1.960640
C        3.456998       0.716682      -1.599998
C        4.255334       0.671225      -0.522851
H        3.368381       1.414066      -2.426297
H        5.025409       1.324794      -0.130236
C       -0.066536      -0.144497      -0.061337
C       -0.626134       1.146486       0.011341
C       -0.912816      -1.270160      -0.110458
C       -2.022292       1.312677       0.026722
C       -2.308829      -1.108343      -0.095571
C       -2.860300       0.185608      -0.054687
C        2.516841      -0.217250       2.348097
C       -0.307298      -2.659502      -0.186672
H        0.780208      -2.618058      -0.264157
H       -0.539369      -3.255872       0.702531
H       -0.674568      -3.217531      -1.053562
H        2.981954      -1.073251       2.850342
H        1.507783      -0.090554       2.744812
H        3.100594       0.679074       2.593460
C        0.285837       2.358520       0.055415
H        1.334755       2.075344      -0.040508
H        0.064789       3.062369      -0.753095
H        0.186900       2.910982       0.996497
C       -2.632811       2.696814       0.125251
H       -1.912359       3.437120       0.468903
H       -3.012828       3.042891      -0.844001
H       -3.473543       2.716093       0.822832
C       -4.364569       0.365336      -0.098384
```

```
H       -4.803968       0.363073       0.907570
H       -4.645312       1.308288      -0.568183
H       -4.850263      -0.432069      -0.661452
C       -3.226315      -2.314772      -0.126070
H       -4.053853      -2.207709       0.579380
H       -3.669115      -2.463002      -1.118799
H       -2.701684      -3.233574       0.130472

----------------------------------------
35
N-Me-B-p-OMe-aza | SCF Done:  E(RB3LYP) =  -699.386000231     A.U. after    1
cycles
C       -3.702990      -0.140366       0.843177
C       -2.277412      -0.093956      -1.578370
B       -1.510677      -0.023112      -0.268628
N       -2.338354      -0.054001       0.923100
H       -4.239138      -0.157167       1.786416
H       -1.768693      -0.078291      -2.538476
C       -3.646972      -0.179186      -1.570770
C       -4.369187      -0.202888      -0.347461
H       -4.211711      -0.230234      -2.498925
H       -5.449722      -0.270126      -0.340400
C        0.065856       0.078972      -0.163343
C        0.711979       1.338778      -0.150847
C        0.865206      -1.078483      -0.129363
C        2.100264       1.420234      -0.098769
C        2.263411      -0.985225      -0.077492
C        2.881337       0.263309      -0.061152
H        2.602742       2.381066      -0.091741
H        2.849179      -1.895250      -0.055115
C       -0.097140       2.617549      -0.193416
H       -0.766717       2.636503      -1.058326
H       -0.726732       2.724364       0.696477
H        0.551048       3.494485      -0.247530
C        0.231390      -2.454146      -0.140728
H       -0.335313      -2.642542       0.777706
H       -0.469265      -2.564553      -0.973173
H        0.985917      -3.238968      -0.228178
O        4.235339       0.458970      -0.012663
C        5.084286      -0.677974       0.016695
H        4.962135      -1.294015      -0.881246
H        6.101420      -0.289957       0.050607
H        4.902317      -1.292921       0.905326
C       -1.782273       0.005946       2.283340
H       -0.697936       0.062033       2.226673
H       -2.155154       0.887689       2.812111
H       -2.061768      -0.885704       2.851549

----------------------------------------
35
N-Me-B-p-OMe-TS | SCF Done:  E(RB3LYP) =  -699.251901604     A.U. after    1 cycles
C        2.423102      -0.030367      -1.558882
B        1.562460       0.141941      -0.383663
N        2.271529       0.581541       1.080320
C        3.195574      -0.321155       0.811851
C        4.281081       0.061058      -0.127317
C        3.848969       0.131870      -1.403189
H        3.199500      -1.285909       1.337676
H        2.005979      -0.233889      -2.540999
H        4.500288       0.409694      -2.227601
H        5.199881       0.478626       0.270998
C        0.007340      -0.054549      -0.236841
C       -0.598027      -1.332452      -0.138260
C       -0.827453       1.081488      -0.256582
C       -1.982115      -1.447836      -0.055278
C       -2.220141       0.949056      -0.174094
C       -2.798112      -0.314405      -0.070525
H       -2.455863      -2.420220       0.018890
H       -2.833427       1.840644      -0.198705
```

```
C        0.234592      -2.594946      -0.115216
H        0.819665      -2.673548       0.808524
H       -0.398047      -3.482649      -0.178160
H        0.948497      -2.615611      -0.941416
C       -0.246526       2.471358      -0.412803
H        0.628360       2.625379       0.223610
H        0.083564       2.640104      -1.442822
H       -0.985659       3.238063      -0.169363
O       -4.142100      -0.547256       0.018356
C       -5.027837       0.562614      -0.004731
H       -6.030529       0.144819       0.072795
H       -4.850044       1.235768       0.841258
H       -4.941500       1.125189      -0.940814
C        1.484848       0.410656       2.307211
H        1.339423      -0.646569       2.565403
H        2.028830       0.897773       3.119792
H        0.513105       0.885544       2.198092

----------------------------------------
35
N-Me-B-p-OMe-Dewar | SCF Done:  E(RB3LYP) =  -699.289369021     A.U. after    1
cycles
C       -3.522569       0.587334       0.226743
C       -2.543537       1.035506      -0.918780
B       -1.418428       0.348908       0.037038
N       -2.395794       0.017363       0.992526
H       -4.139350       1.305893       0.772374
H       -2.486096       2.083155      -1.215929
C       -3.356721       0.085718      -1.790969
C       -4.179422      -0.319720      -0.812223
H       -3.255148      -0.191918      -2.834768
H       -4.965527      -1.063038      -0.750757
C        0.124041       0.090175      -0.003534
C        0.645829      -1.215503      -0.176273
C        1.030275       1.162445       0.109244
C        2.020903      -1.421053      -0.227493
C        2.412191       0.940722       0.067262
C        2.908758      -0.350755      -0.102131
H        2.430491      -2.414970      -0.368875
H        3.082223       1.785086       0.165334
C        0.528826       2.580388       0.285658
H        1.356008       3.280891       0.418118
H       -0.047977       2.907382      -0.584934
H       -0.125928       2.670034       1.158011
O        4.237506      -0.668440      -0.161987
C        5.192203       0.376776      -0.052605
H        5.116517       0.890515       0.912216
H        6.167555      -0.101613      -0.129383
H        5.082636       1.106881      -0.862163
C       -2.505442      -0.759158       2.204562
H       -1.507550      -1.021443       2.560900
H       -3.013087      -0.190439       2.991756
H       -3.070270      -1.685333       2.036953
C       -0.273983      -2.410297      -0.317872
H       -0.766977      -2.650465       0.629682
H       -1.063596      -2.228412      -1.051585
H        0.280623      -3.296245      -0.633456

----------------------------------------
32
N-Me-B-p-CN-aza | SCF Done:  E(RB3LYP) =  -677.098939050     A.U. after    1 cycles
C        3.567740       0.000725       0.838041
C        2.128842      -0.001417      -1.578477
B        1.374197      -0.000260      -0.263342
N        2.200230       0.000809       0.925064
H        4.108076       0.001566       1.778729
H        1.616068      -0.002264      -2.536296
C        3.501637      -0.001424      -1.576209
C        4.229399      -0.000348      -0.356651
```

```
H        4.063456      -0.002255      -2.507141
H        5.311885      -0.000356      -0.355545
C       -0.209594      -0.000149      -0.157999
C       -0.925224      -1.218576      -0.137313
C       -0.925181       1.218320      -0.138812
C       -2.318824      -1.210085      -0.090318
C       -2.318787       1.209932      -0.091813
C       -3.020506      -0.000047      -0.065605
H       -2.869278      -2.143677      -0.075368
H       -2.869208       2.143562      -0.078018
C       -0.195866      -2.544049      -0.162966
H        0.481278      -2.609057      -1.019135
H        0.413713      -2.683101       0.735881
H       -0.896096      -3.379466      -0.220404
C       -0.195786       2.543744      -0.165891
H        0.412396       2.684488       0.733650
H        0.482665       2.607155      -1.021133
H       -0.895931       3.379050      -0.225937
C       -4.450696       0.000006      -0.015958
N       -5.606085       0.000031       0.026207
C        1.648472       0.002110       2.289151
H        0.562195       0.001773       2.242600
H        1.977751      -0.885245       2.836359
H        1.977273       0.890799       2.834481

----------------------------------------
32
N-Me-B-p-CN-TS | SCF Done:  E(RB3LYP) =  -676.963639758     A.U. after    1 cycles
C        2.266196      -0.061495      -1.556589
B        1.439122       0.130472      -0.362566
N        2.163874       0.470744       1.093527
C        3.029930      -0.481892       0.797947
C        4.134206      -0.146176      -0.139319
C        3.702784      -0.000191      -1.407321
H        2.984927      -1.450875       1.313723
H        1.828760      -0.213668      -2.538745
H        4.366503       0.260415      -2.226897
H        5.087132       0.175650       0.267325
C       -0.134225       0.028213      -0.231955
C       -0.810774      -1.211077      -0.161007
C       -0.886739       1.227211      -0.243036
C       -2.203937      -1.238838      -0.091506
C       -2.278367       1.178097      -0.172896
C       -2.941447      -0.050777      -0.093055
H       -2.726316      -2.186882      -0.037919
H       -2.856445       2.094804      -0.183615
C       -0.058496      -2.522647      -0.155194
H        0.508467      -2.655921       0.773125
H       -0.744259      -3.367489      -0.243011
H        0.661358      -2.571012      -0.974549
C       -0.205581       2.572767      -0.364177
H       -0.917206       3.387612      -0.218203
H        0.601576       2.689849       0.363717
H        0.246686       2.691346      -1.353150
N       -5.523830      -0.125477       0.047453
C       -4.369959      -0.092589      -0.015500
C        1.376849       0.327498       2.324281
H        1.960231       0.754555       3.142667
H        0.442149       0.876034       2.238164
H        1.157883      -0.722275       2.559297

----------------------------------------
32
N-Me-B-p-CN-Dewar | SCF Done:  E(RB3LYP) =  -677.001807284     A.U. after    1
cycles
C       -3.393790       0.517123      -0.044385
C       -2.452138       0.274138      -1.281393
B       -1.290288       0.278542      -0.145864
N       -2.231217       0.494848       0.869848
```

```
H       -4.025174       1.405382       0.030826
H       -2.435959       0.979156      -2.113179
C       -3.245191      -1.017087      -1.447406
C       -4.032623      -0.827492      -0.378560
H       -3.156474      -1.822732      -2.168197
H       -4.791426      -1.425041       0.112034
C        0.271456       0.127280      -0.094256
C        0.864563      -1.141269       0.091579
C        1.097457       1.261880      -0.256883
C        2.252819      -1.261840       0.120420
C        2.483799       1.123279      -0.225838
C        3.066714      -0.134597      -0.036516
H        2.712049      -2.233001       0.264048
H        3.120983       1.991315      -0.348911
C        0.492369       2.632610      -0.465368
H        1.266071       3.391928      -0.592091
H       -0.144391       2.653990      -1.355278
H       -0.130996       2.927242       0.384102
N        5.641887      -0.375673       0.021920
C        4.491072      -0.268002      -0.004686
C        0.008357      -2.377336       0.256438
H       -0.650044      -2.295529       1.126436
H       -0.633183      -2.535789      -0.615335
H        0.623168      -3.269963       0.384968
C       -2.286119       0.505260       2.313713
H       -1.273620       0.452767       2.718307
H       -2.760147       1.420682       2.684392
H       -2.856594      -0.351021       2.693677

----------------------------------------
28
N-Et-B-Ph-aza | SCF Done:  E(RB3LYP) =  -545.505113393     A.U. after    1 cycles
C       -2.823457       0.356927      -0.292847
C       -1.207852      -1.910325       0.078986
B       -0.552416      -0.552669      -0.089726
N       -1.469429       0.559323      -0.264615
H       -3.436620       1.241080      -0.432191
H       -0.621347      -2.813976       0.219319
C       -2.575281      -2.022238       0.034497
C       -3.392894      -0.877871      -0.158651
H       -3.063291      -2.988321       0.140835
H       -4.471045      -0.964950      -0.204382
C        1.014037      -0.350525      -0.074647
C        1.729153       0.127183      -1.187371
C        1.763680      -0.723239       1.054604
C        3.118721       0.237791      -1.172122
C        3.152811      -0.603875       1.083192
C        3.835835      -0.122123      -0.031978
H        3.642047       0.600869      -2.050416
H        3.702051      -0.892451       1.973196
H        1.250952      -1.113533       1.928617
H        1.193918       0.401414      -2.092144
H        4.916630      -0.033999      -0.015628
C       -1.031689       1.970978      -0.317532
H       -0.012723       1.997097      -0.696654
H       -1.663033       2.499187      -1.038314
C       -1.094646       2.654584       1.048828
H       -0.773179       3.696399       0.963306
H       -2.109929       2.643823       1.454108
H       -0.436857       2.151848       1.761529

----------------------------------------
28
N-Et-B-Ph-TS | SCF Done:  E(RB3LYP) =  -545.367615969     A.U. after    1 cycles
C       -1.452010      -1.884769       0.017110
B       -0.696530      -0.645604       0.239943
N       -1.507218       0.758786       0.580274
C       -2.345731       0.425850      -0.384599
C       -3.395319      -0.579334      -0.058655
```

```
C       -2.892878      -1.822579       0.075987
H       -2.339844       0.944829      -1.348574
H       -0.951786      -2.835529      -0.139450
H       -3.508710      -2.681393       0.328711
H       -4.366272      -0.228273       0.275877
C        0.862690      -0.458609       0.109025
C        1.530247      -0.707201      -1.102697
C        1.654200      -0.140379       1.227273
C        2.919447      -0.630186      -1.197935
C        3.043661      -0.069207       1.141132
C        3.680887      -0.308746      -0.075630
H        3.628827       0.167339       2.023461
H        3.407408      -0.827373      -2.146608
H        4.761617      -0.252255      -0.146299
H        0.951772      -0.972908      -1.981306
H        1.178380       0.031026       2.188383
C       -0.800908       2.058373       0.550547
H        0.123179       1.931996       1.110637
H       -1.432572       2.736428       1.134114
C       -0.501324       2.675769      -0.816890
H        0.053777       3.604731      -0.664626
H        0.115279       2.013489      -1.428346
H       -1.405644       2.930524      -1.375615

----------------------------------------
28
N-Et-B-Ph-Dewar | SCF Done:  E(RB3LYP) =  -545.411685356     A.U. after    1 cycles
C        2.437025      -0.555688       0.438550
C        1.273827      -1.576978       0.724780
B        0.339481      -0.337367       0.266517
N        1.463360       0.484897       0.040538
H        3.142767      -0.267836       1.220654
H        1.172597      -2.035094       1.709579
C        1.916219      -2.395341      -0.389556
C        2.892319      -1.515706      -0.657046
H        1.630781      -3.341425      -0.837396
H        3.665527      -1.458280      -1.413937
C       -1.192345      -0.114951       0.120333
C       -1.780225       1.157100      -0.012644
C       -2.054017      -1.226704       0.126951
C       -3.158004       1.311012      -0.141659
C       -3.432752      -1.082251      -0.010770
C       -3.987896       0.189442      -0.145566
H       -3.587022       2.302672      -0.237521
H       -4.074228      -1.956892      -0.007466
H       -5.061254       0.307365      -0.247445
H       -1.632347      -2.220272       0.241809
H       -1.153737       2.042585      -0.001241
C        1.802530       1.736109      -0.608662
H        0.895609       2.141403      -1.064160
H        2.500107       1.529003      -1.431829
C        2.420627       2.767098       0.340745
H        2.668811       3.684945      -0.200093
H        1.726889       3.018499       1.147117
H        3.340727       2.387031       0.792843

----------------------------------------
31
N-Et-B-o-Tol-aza | SCF Done:  E(RB3LYP) =  -584.830872625     A.U. after    1
cycles
C        2.887248       0.315238       0.320840
C        1.329152      -1.708862      -0.854145
B        0.642358      -0.493041      -0.262632
N        1.530509       0.496952       0.316559
H        3.476172       1.102521       0.779688
H        0.767682      -2.514514      -1.318732
C        2.697907      -1.810582      -0.808834
C        3.486034      -0.791496      -0.212176
H        3.209999      -2.674682      -1.225973
```

```
H        4.564416      -0.876400      -0.167651
C       -0.925250      -0.270219      -0.274475
C       -1.749428      -0.614478       0.821407
C       -1.540502       0.239295      -1.428929
C       -3.132352      -0.426183       0.731108
C       -2.920183       0.426033      -1.509527
C       -3.720231       0.093377      -0.420853
H       -3.759814      -0.695997       1.575284
H       -3.364319       0.822559      -2.416192
H       -0.927134       0.490365      -2.289669
H       -4.795323       0.229278      -0.467285
C       -1.153609      -1.192918       2.086409
H       -0.552664      -2.082014       1.873179
H       -0.492639      -0.477712       2.588068
H       -1.933230      -1.475434       2.797115
C        1.043551       1.771696       0.884677
H        0.003838       1.630231       1.176426
H        1.615606       1.984080       1.793117
C        1.153007       2.938320      -0.097370
H        0.794123       3.858763       0.371626
H        2.187341       3.098908      -0.412972
H        0.548546       2.751269      -0.987574
```

```
31
N-Et-B-o-Tol-TS | SCF Done:  E(RB3LYP) =  -584.696482669     A.U. after    1 cycles
C        1.238402      -1.904447      -0.768735
B        0.601601      -0.618831      -0.467234
N        1.555607       0.719606      -0.193900
C        2.311913      -0.056051       0.559686
C        3.284836      -0.952086      -0.120423
C        2.681274      -1.972218      -0.762657
H        2.282231       0.038808       1.654515
H        0.654658      -2.781863      -1.030219
H        3.235106      -2.715943      -1.329267
H        4.304257      -0.607600      -0.260190
C       -0.934793      -0.288274      -0.327888
C       -1.718078      -0.610851       0.803242
C       -1.570337       0.309892      -1.429334
C       -3.088280      -0.325452       0.790019
C       -2.936325       0.584970      -1.433156
C       -3.698854       0.267494      -0.312830
H       -3.399095       1.038131      -2.303063
H       -3.688537      -0.579006       1.658468
H       -4.763621       0.473602      -0.297055
H       -0.981604       0.553504      -2.308974
C        0.958706       1.915719       0.426349
C        1.923288       3.096478       0.315718
H        2.855671       2.903246       0.853710
H        2.168932       3.300731      -0.728731
H        1.464855       3.991434       0.744350
C       -1.108771      -1.256705       2.026816
H       -0.516716      -2.135013       1.760040
H       -0.434987      -0.569401       2.551657
H       -1.881899      -1.560992       2.735746
H        0.718921       1.720268       1.481754
H        0.026682       2.136195      -0.090101
```

```
31
N-Et-B-o-Tol-Dewar | SCF Done:  E(RB3LYP) =  -584.734996101     A.U. after    1
cycles
C       -2.487821      -0.787632      -0.461700
C       -1.317290      -1.728956      -0.001822
B       -0.413587      -0.398605      -0.224943
N       -1.543130       0.336884      -0.640362
H       -3.099776      -1.017863      -1.337783
H       -1.120692      -2.652382      -0.547387
C       -2.088465      -1.818050       1.310052
```

```
C       -3.077911      -0.995769       0.932624
H       -1.867195      -2.331973       2.239246
H       -3.935602      -0.570981       1.440788
C        1.071600       0.039485      -0.030390
C        1.340762       1.339764       0.435745
C        2.171862      -0.819442      -0.271926
C        2.637840       1.793231       0.662834
C        3.470108      -0.349983      -0.053515
C        3.709366       0.941926       0.411897
H        2.809036       2.798846       1.030993
H        4.310352      -1.009082      -0.248701
H        4.727397       1.277445       0.578048
H        0.512202       2.008338       0.644434
C       -1.895814       1.686125      -1.037124
C       -2.694414       2.455849       0.020602
H       -2.130270       2.543835       0.952674
H       -2.926737       3.462585      -0.338640
H       -3.639794       1.955631       0.246115
C        1.977013      -2.234460      -0.769839
H        1.439467      -2.843573      -0.037785
H        1.397527      -2.261570      -1.697093
H        2.938515      -2.714449      -0.963675
H       -2.475981       1.639930      -1.967954
H       -0.969630       2.217580      -1.270660

---------------------------------------
34
N-Et-B-Xylyl-aza | SCF Done:  E(RB3LYP) =  -624.155840009     A.U. after    1
cycles
C       -2.939243       0.101213      -0.457432
C       -1.330982      -1.336746       1.342984
B       -0.669308      -0.429838       0.321187
N       -1.584266       0.272655      -0.559926
H       -3.548524       0.655044      -1.163869
H       -0.749587      -1.916763       2.054565
C       -2.698537      -1.448166       1.378663
C       -3.512915      -0.723426       0.468377
H       -3.190140      -2.097029       2.099944
H       -4.591168      -0.818624       0.488874
C        0.900264      -0.251437       0.183046
C        1.623593      -0.995051      -0.776967
C        1.607278       0.607222       1.054254
C        3.012276      -0.857031      -0.867484
C        2.995684       0.730384       0.938530
C        3.697941       0.005683      -0.018743
H        3.558919      -1.434012      -1.606881
H        3.529621       1.396905       1.608678
H        4.774955       0.107538      -0.098342
C        0.913213      -1.953274      -1.709813
H        0.296688      -2.667489      -1.156061
H        0.245366      -1.429133      -2.402111
H        1.628466      -2.520020      -2.309710
C        0.886160       1.384589       2.135449
H        0.049549       1.965274       1.736472
H        0.468024       0.714629       2.893341
H        1.563692       2.077135       2.639698
C       -1.131555       1.219271      -1.601922
H       -1.682945       1.004971      -2.522914
H       -0.078867       1.019143      -1.794615
C       -1.316612       2.684543      -1.205874
H       -0.981562       3.335474      -2.018178
H       -2.364503       2.916018      -0.997651
H       -0.730972       2.924896      -0.316100

---------------------------------------
34
N-Et-B-Xylyl-TS | SCF Done:  E(RB3LYP) =  -624.022092576     A.U. after    1 cycles
C       -1.304656      -1.734864       1.049770
B       -0.648939      -0.565572       0.454920
```

```
N       -1.588579       0.709120      -0.080864
C       -2.381889      -0.198866      -0.618549
C       -3.351600      -0.889597       0.271583
C       -2.747649      -1.762068       1.103505
H       -2.380752      -0.360718      -1.705626
H       -0.731017      -2.551338       1.478632
H       -3.295818      -2.347803       1.836577
H       -4.356082      -0.490057       0.364694
C        0.888107      -0.316909       0.191204
C        1.575706      -0.902622      -0.895761
C        1.608634       0.481548       1.110886
C        2.947909      -0.677878      -1.050108
C        2.979520       0.687108       0.929801
C        3.648268       0.114129      -0.146834
H        3.525680       1.297563       1.642020
H        3.470846      -1.132563      -1.885523
C        0.859543      -1.769742      -1.907433
H        0.256505      -2.538506      -1.419659
H        0.176591      -1.182011      -2.532337
H        1.571884      -2.258531      -2.575674
C        0.922571       1.093234       2.313634
H        1.586643       1.789045       2.831060
H        0.012250       1.632929       2.037848
H        0.621255       0.320891       3.027961
H        4.712211       0.279766      -0.278378
C       -0.983003       1.710898      -0.976835
H       -0.774436       1.267952      -1.961343
H       -0.032078       2.020844      -0.547533
C       -1.918796       2.910231      -1.127418
H       -2.868298       2.621119      -1.586730
H       -2.134152       3.360595      -0.155917
H       -1.452478       3.667220      -1.763293

----------------------------------------
34
N-Et-B-Xylyl-Dewar | SCF Done:  E(RB3LYP) =  -624.059311219     A.U. after    1
cycles
C       -2.603242       0.041079      -0.657043
C       -1.496282      -0.523594      -1.621893
B       -0.492214      -0.043660      -0.436684
N       -1.570282       0.431008       0.329278
H       -3.279560       0.837696      -0.972938
H       -1.402821      -0.144761      -2.640291
C       -2.200333      -1.859130      -1.414081
C       -3.129874      -1.389135      -0.569143
H       -1.978128      -2.851095      -1.792926
H       -3.915234      -1.851418       0.016962
C        1.050173      -0.061863      -0.155741
C        1.621197      -1.049342       0.677335
C        1.890020       0.903373      -0.754290
C        3.001111      -1.054718       0.901581
C        3.265529       0.876354      -0.509598
C        3.821725      -0.097117       0.314356
H        3.434392      -1.817718       1.540584
H        3.904470       1.623240      -0.970153
C        0.758352      -2.111735       1.324574
H        0.021467      -1.674673       2.005753
H        0.200140      -2.686312       0.579334
H        1.365700      -2.814181       1.899196
C        1.312930       1.972368      -1.657060
H        0.800635       1.533213      -2.519070
H        0.580705       2.592348      -1.130035
H        2.095237       2.632516      -2.037301
H        4.891028      -0.111012       0.495871
C       -1.803081       0.906393       1.678987
H       -0.832199       0.957102       2.179648
H       -2.407868       0.167400       2.223393
C       -2.492000       2.272253       1.733196
H       -2.641775       2.581768       2.771302
```

```
H       -1.887875       3.032642       1.231787
H       -3.472624       2.243377       1.250293

----------------------------------------
37
N-Et-B-Mes-aza | SCF Done:  E(RB3LYP) =  -663.483157512     A.U. after    1 cycles
C       -3.292766       0.140349      -0.481076
C       -1.722984      -1.349676       1.310444
B       -1.037337      -0.433877       0.312024
N       -1.934746       0.295805      -0.566018
H       -3.887204       0.714561      -1.183948
H       -1.156941      -1.949788       2.017762
C       -3.092113      -1.444042       1.329630
C       -3.887225      -0.692850       0.423702
H       -3.599856      -2.099152       2.033942
H       -4.966789      -0.774314       0.430862
C        0.534731      -0.272939       0.193084
C        1.263590      -1.003811      -0.770918
C        1.249386       0.552480       1.088188
C        2.654474      -0.883539      -0.839578
C        2.640054       0.654640       0.989616
C        3.363291      -0.051451       0.027680
H        3.197707      -1.458963      -1.584443
H        3.172267       1.296341       1.686764
C        0.556475      -1.936304      -1.732199
H       -0.072012      -2.656968      -1.200588
H       -0.099697      -1.391800      -2.419955
H        1.273398      -2.496103      -2.336645
C        0.533075       1.316334       2.182390
H       -0.285630       1.926067       1.789376
H        0.090673       0.635628       2.916544
H        1.219115       1.979940       2.713375
C       -1.458689       1.255899      -1.584790
H       -2.005769       1.069303      -2.514376
H       -0.407630       1.043096      -1.772628
C       -1.624140       2.715476      -1.159993
H       -1.272549       3.377880      -1.955932
H       -2.669981       2.958887      -0.954954
H       -1.041992       2.927841      -0.260851
C        4.862091       0.095962      -0.084436
H        5.314401       0.322180       0.884335
H        5.324857      -0.815944      -0.469877
H        5.128034       0.910885      -0.767232

----------------------------------------
37
N-Et-B-Mes-TS | SCF Done:  E(RB3LYP) =  -663.345355336     A.U. after    1 cycles
C        1.932421       1.169191      -1.420901
B        1.147608       0.546098      -0.348249
N        1.944285      -0.068520       0.989496
C        2.787620      -0.614149       0.132791
C        3.850732       0.272214      -0.418746
C        3.370074       1.167787      -1.304777
H        2.779795      -1.688544      -0.075908
H        1.455591       1.657037      -2.266196
H        3.999546       1.907699      -1.791892
H        4.806381       0.317816       0.094124
C       -0.412839       0.322287      -0.274700
C       -1.078950      -0.641303      -1.066912
C       -1.189698       1.165654       0.553388
C       -2.469140      -0.765959      -0.988923
C       -2.578053       1.013079       0.603532
C       -3.238077       0.044407      -0.152542
H       -3.157927       1.673785       1.242309
H       -2.965403      -1.511698      -1.603919
C       -0.325697      -1.531541      -2.031549
H        0.201939      -0.940608      -2.784325
H        0.434691      -2.137288      -1.528822
H       -1.007330      -2.212817      -2.545573
```

```
C       -0.549781       2.285795       1.346512
H       -0.264589       3.111159       0.686004
H       -1.240149       2.681493       2.094925
H        0.363146       1.968845       1.856252
C       -4.734239      -0.134129      -0.056750
H       -4.993699      -0.871638       0.711206
H       -5.231565       0.801861       0.208748
H       -5.155336      -0.486345      -1.001658
C        1.219011      -0.887690       1.983999
H        1.788198      -0.766987       2.912190
H        0.251047      -0.416445       2.144621
C        1.030186      -2.375773       1.689172
H        0.446985      -2.537423       0.780768
H        1.977925      -2.913780       1.600425
H        0.481298      -2.826184       2.519946

----------------------------------------
37
N-Et-B-Mes-Dewar | SCF Done:  E(RB3LYP) =  -663.386777247     A.U. after    1
cycles
C       -2.993211       0.046290      -0.590605
C       -1.914294      -0.455196      -1.619604
B       -0.873895      -0.019408      -0.447947
N       -1.931004       0.404266       0.376188
H       -3.691321       0.846560      -0.843797
H       -1.862884      -0.026643      -2.621168
C       -2.592431      -1.809654      -1.451945
C       -3.497370      -1.394466      -0.553228
H       -2.370444      -2.779196      -1.885159
H       -4.254710      -1.896052       0.037493
C        0.676151      -0.035022      -0.220989
C        1.284880      -1.023964       0.581411
C        1.496342       0.937085      -0.833766
C        2.671219      -1.022789       0.756891
C        2.878046       0.912397      -0.633443
C        3.487751      -0.063128       0.157331
H        3.124519      -1.791168       1.377318
H        3.493584       1.673010      -1.106113
C        0.455447      -2.097444       1.254348
H       -0.237001      -1.672745       1.988111
H       -0.149501      -2.651955       0.530926
H        1.091326      -2.815627       1.776317
C        0.890283       2.018555      -1.702327
H        0.350005       1.590674      -2.552767
H        0.175400       2.630528      -1.142964
H        1.659291       2.684731      -2.098916
C       -2.122254       0.815293       1.753056
H       -1.134766       0.860727       2.220613
H       -2.695558       0.043053       2.285424
C       -2.829480       2.165721       1.892594
H       -2.946081       2.426912       2.948125
H       -2.255519       2.957243       1.404069
H       -3.826484       2.141643       1.444172
C        4.987280      -0.097704       0.331566
H        5.267827      -0.550125       1.285875
H        5.461722      -0.686648      -0.461508
H        5.416940       0.906412       0.292635

----------------------------------------
43
N-Et-B-pentamethylphenyl-aza | SCF Done:  E(RB3LYP) =  -742.124856553     A.U.
after    1 cycles
C       -3.691758       0.199032       0.486497
C       -2.143700       0.229588      -1.857777
B       -1.442748       0.150741      -0.511894
N       -2.331762       0.138934       0.637438
H       -4.277131       0.195382       1.400054
H       -1.585525       0.247602      -2.789880
C       -3.513315       0.284871      -1.919700
```

```
C       -4.298285       0.270741      -0.735385
H       -4.030162       0.345458      -2.874879
H       -5.378597       0.324148      -0.782469
C        0.133220       0.095234      -0.328556
C        0.864811       1.278397      -0.094558
C        0.821981      -1.128966      -0.451515
C        2.263800       1.235295       0.046449
C        2.221088      -1.176607      -0.310461
C        2.935284       0.001657      -0.028639
C        0.137648       2.607762       0.003960
H        0.429694       3.289225      -0.802440
H       -0.942778       2.480396      -0.066190
H        0.348338       3.121589       0.947698
C        0.050001      -2.405113      -0.735712
H       -1.025643      -2.228490      -0.747874
H        0.312651      -2.828762      -1.711328
H        0.249683      -3.179833       0.011852
C        4.434369      -0.057225       0.188408
H        4.987684       0.068222      -0.751319
H        4.775137       0.725070       0.867198
H        4.742947      -1.011496       0.616359
C        3.056570       2.506709       0.279177
H        2.487405       3.396842       0.017022
H        3.355598       2.613332       1.329519
H        3.972700       2.522771      -0.315846
C        2.968013      -2.487830      -0.458426
H        3.254176      -2.905375       0.515150
H        2.371113      -3.242809      -0.967123
H        3.888121      -2.362561      -1.033986
C       -1.839760       0.043173       2.028124
H       -0.800742       0.368616       2.033209
H       -2.409384       0.746979       2.643127
C       -1.946567      -1.369258       2.604036
H       -1.582208      -1.381409       3.635170
H       -2.980819      -1.723913       2.606487
H       -1.346578      -2.071994       2.022358

----------------------------------------
43
N-Et-B-pentamethylphenyl-TS | SCF Done:  E(RB3LYP) =  -741.990727748     A.U. after
1 cycles
C       -2.155343       0.588794      -1.909866
B       -1.429256       0.128042      -0.718862
N       -2.308133      -0.476240       0.575716
C       -3.122403       0.548724       0.401374
C       -4.144107       0.445307      -0.672285
C       -3.597605       0.549961      -1.901660
H       -3.092283       1.404648       1.089992
H       -1.627237       0.914319      -2.801497
H       -4.186784       0.484242      -2.812620
H       -5.125869       0.051787      -0.429891
C        0.123948       0.123454      -0.423025
C        0.824915       1.280200      -0.021630
C        0.827439      -1.089936      -0.594979
C        2.211596       1.222520       0.210577
C        2.216838      -1.142345      -0.384450
C        2.910490       0.021511      -0.005617
C        0.096673       2.598741       0.151629
H       -0.907452       2.548379      -0.265972
H        0.009905       2.884061       1.207836
H        0.619152       3.415641      -0.354066
C        0.084591      -2.338600      -1.035325
H        0.524436      -2.767496      -1.940829
H        0.107537      -3.120866      -0.267872
H       -0.961090      -2.125155      -1.255330
C       -1.639559      -0.609056       1.882490
H       -1.426758       0.382847       2.306624
H       -0.686953      -1.111556       1.726461
C       -2.520322      -1.413436       2.838674
```

```
H       -3.471072      -0.907098       3.028330
H       -2.737754      -2.402069       2.428304
H       -2.008955      -1.541447       3.796397
C        2.974942      -2.443027      -0.565607
H        2.312837      -3.306824      -0.550738
H        3.515015      -2.468070      -1.520262
H        3.715228      -2.589812       0.224226
C        4.417078      -0.016565       0.159329
H        4.709750      -0.247991       1.191606
H        4.873367      -0.771995      -0.480233
H        4.874125       0.939602      -0.097312
C        2.957499       2.453820       0.687301
H        3.412353       3.004448      -0.146059
H        2.299715       3.148796       1.207886
H        3.761973       2.194667       1.377741

---------------------------------------
43
N-Et-B-pentamethylphenyl-Dewar | SCF Done:  E(RB3LYP) =  -742.028782055     A.U.
after    1 cycles
C        3.410495      -0.234332      -0.549946
C        2.336979      -0.023459      -1.681099
B        1.289471      -0.113140      -0.438029
N        2.344325      -0.298665       0.474220
H        4.099427      -1.081269      -0.570093
H        2.276808      -0.709033      -2.527468
C        3.031456       1.316222      -1.891041
C        3.932230       1.154702      -0.910205
H        2.819526       2.131909      -2.574220
H        4.694735       1.791999      -0.478080
C       -0.264277      -0.037124      -0.231353
C       -0.878534       1.176682       0.135898
C       -1.055063      -1.186185      -0.431678
C       -2.274096       1.243915       0.294368
C       -2.450267      -1.123153      -0.274257
C       -3.059282       0.100057       0.061647
C       -0.029007       2.417226       0.341028
H       -0.068969       2.773853       1.376362
H        1.019491       2.229074       0.107220
H       -0.359549       3.244664      -0.294618
C       -0.391263      -2.492722      -0.825450
H        0.680852      -2.365356      -0.982202
H       -0.510031      -3.260622      -0.053097
H       -0.809514      -2.900020      -1.751174
C        2.528667      -0.300867       1.911910
H        1.540739      -0.188702       2.367005
H        3.117784       0.580362       2.202990
C        3.208374      -1.568110       2.437050
H        3.321228      -1.517523       3.523742
H        2.617841      -2.455017       2.193626
H        4.205124      -1.693799       2.005089
C       -2.941064       2.540680       0.708761
H       -2.231491       3.244959       1.139769
H       -3.416520       3.043039      -0.142902
H       -3.720981       2.370629       1.454743
C       -4.568360       0.186235       0.172819
H       -4.913868      -0.022804       1.193574
H       -4.936543       1.177596      -0.092849
H       -5.063537      -0.527978      -0.485408
C       -3.305126      -2.360398      -0.466046
H       -4.070623      -2.443726       0.309154
H       -3.826658      -2.347989      -1.431144
H       -2.714056      -3.274101      -0.433849

---------------------------------------
38
N-Et-B-p-OMe-aza | SCF Done:  E(RB3LYP) =  -738.712059225     A.U. after    1
cycles
C       -3.632434       0.174971      -0.424014
```

```
C       -2.061766      -1.001731       1.586101
B       -1.374524      -0.350091       0.399078
N       -2.273470       0.233326      -0.582060
H       -4.227779       0.625825      -1.211100
H       -1.495790      -1.474595       2.384191
C       -3.431886      -1.013323       1.668028
C       -4.227580      -0.420692       0.651870
H       -3.940299      -1.480453       2.508511
H       -5.308558      -0.438452       0.709704
C        0.196127      -0.302570       0.198333
C        0.851144      -1.256160      -0.620149
C        0.986453       0.645903       0.873147
C        2.234781      -1.232564      -0.765239
C        2.380230       0.663558       0.715577
C        3.004591      -0.272478      -0.105042
H        2.742636      -1.959897      -1.388810
H        2.957713       1.411381       1.243969
C        0.058859      -2.325175      -1.342720
H       -0.565833      -2.894149      -0.647787
H       -0.613678      -1.895165      -2.092632
H        0.719590      -3.026914      -1.855590
C        0.360927       1.652913       1.816692
H       -0.548059       2.095772       1.402319
H        0.075885       1.179529       2.762212
H        1.055321       2.463748       2.049125
O        4.354354      -0.335990      -0.324428
C        5.192799       0.602276       0.332095
H        5.119907       0.512682       1.421764
H        6.208609       0.364810       0.019058
H        4.954612       1.630272       0.036480
C       -1.794842       0.940179      -1.789242
H       -0.768458       0.625787      -1.970600
H       -2.398066       0.611546      -2.641460
C       -1.854253       2.462388      -1.656880
H       -1.507547       2.931488      -2.581917
H       -2.872508       2.809620      -1.462503
H       -1.214509       2.807278      -0.841805

----------------------------------------
38
N-Et-B-p-OMe-TS | SCF Done:  E(RB3LYP) =  -738.574533943     A.U. after    1 cycles
C       -2.254626      -0.437978      -1.786827
B       -1.480695      -0.339609      -0.543037
N       -2.304530      -0.234504       0.917231
C       -3.069884       0.660945       0.321449
C       -4.158702       0.137209      -0.549618
C       -3.693168      -0.376199      -1.706495
H       -2.982579       1.729814       0.540201
H       -1.770509      -0.596351      -2.746369
H       -4.342987      -0.821464      -2.455150
H       -5.136516      -0.037557      -0.111801
C        0.082481      -0.278289      -0.354259
C        0.843697       0.857333      -0.699392
C        0.769364      -1.429879       0.110844
C        2.237551       0.855371      -0.547439
C        2.152980      -1.416498       0.254882
C        2.892043      -0.275749      -0.063880
H        2.794104       1.743407      -0.817544
H        2.684845      -2.293534       0.606463
C        0.195051       2.102014      -1.266786
H       -0.320051       1.883655      -2.205417
H       -0.559529       2.518155      -0.592498
H        0.938079       2.879931      -1.456092
C        0.024100      -2.716114       0.398266
H        0.653244      -3.421769       0.944924
H       -0.888177      -2.549451       0.975716
H       -0.284491      -3.196070      -0.536054
O        4.243019      -0.368956       0.123224
C        5.054437       0.749831      -0.203284
```

```
H        6.077525       0.450592       0.019727
H        4.975791       1.005995      -1.265551
H        4.793683       1.625362       0.401664
C       -1.576258       0.085227       2.163661
H       -2.197650      -0.331895       2.963684
H       -0.651081      -0.487976       2.152390
C       -1.273008       1.553259       2.463844
H       -0.737998       1.608583       3.415211
H       -0.634558       1.998411       1.698509
H       -2.176444       2.160484       2.566971
```

```
----------------------------------------
38
N-Et-B-p-OMe-Dewar | SCF Done:  E(RB3LYP) =  -738.615748346     A.U. after    1
cycles
C        3.321511      -0.233808      -0.599830
C        2.246295       0.141240      -1.684444
B        1.207946      -0.057826      -0.447878
N        2.262121      -0.392047       0.421528
H        3.985500      -1.090733      -0.729328
H        2.164779      -0.437776      -2.605059
C        2.977199       1.477660      -1.740413
C        3.880305       1.173866      -0.796447
H        2.784284       2.374982      -2.318839
H        4.665433       1.731945      -0.300163
C       -0.333956       0.067183      -0.208113
C       -0.879421       1.165807       0.500193
C       -1.216034      -0.906124      -0.714760
C       -2.253978       1.266396       0.689624
C       -2.597386      -0.797571      -0.510468
C       -3.117421       0.288390       0.191580
H       -2.682490       2.106838       1.224057
H       -3.248252      -1.566089      -0.907089
C       -0.687208      -2.097834      -1.484899
H       -1.497021      -2.758777      -1.800873
H       -0.146005      -1.781951      -2.382013
H        0.008503      -2.689129      -0.881188
O       -4.447992       0.486157       0.439201
C       -5.379332      -0.467380      -0.049560
H       -5.203543      -1.458763       0.382725
H       -6.360916      -0.110028       0.258480
H       -5.346415      -0.537104      -1.142520
C        2.460965      -0.594027       1.842979
H        1.479565      -0.535951       2.321688
H        3.065728       0.230801       2.246170
C        3.127433      -1.930286       2.180076
H        2.521996      -2.767164       1.822662
H        4.118429      -2.007783       1.724466
H        3.250305      -2.031916       3.261988
C        0.015854       2.255124       1.052130
H        0.666968       1.876186       1.846146
H        0.667043       2.670989       0.278155
H       -0.573489       3.073792       1.469737
```

```
----------------------------------------
35
N-Et-B-p-CN-aza | SCF Done:  E(RB3LYP) =  -716.425069091     A.U. after    2 cycles
C       -3.483967       0.145809      -0.484233
C       -1.915542      -1.344673       1.310352
B       -1.235673      -0.429963       0.311149
N       -2.125409       0.300276      -0.568994
H       -4.077336       0.719794      -1.187783
H       -1.350936      -1.944734       2.018600
C       -3.285420      -1.437430       1.327929
C       -4.078640      -0.686428       0.421306
H       -3.793703      -2.091348       2.032486
H       -5.158129      -0.766984       0.428449
C        0.340816      -0.276466       0.197488
C        1.063953      -1.024542      -0.760118
```

```
C        1.048133       0.557800       1.092881
C        2.452331      -0.916159      -0.830535
C        2.436339       0.658790       1.005793
C        3.143184      -0.071606       0.044775
H        3.007083      -1.489428      -1.564311
H        2.979121       1.303306       1.687553
C        0.350397      -1.957645      -1.714253
H       -0.280513      -2.669705      -1.174885
H       -0.303370      -1.410934      -2.401537
H        1.062112      -2.525046      -2.316765
C        0.323717       1.335486       2.170364
H       -0.487591       1.942440       1.759719
H       -0.128632       0.662044       2.904425
H        1.005904       2.001582       2.702041
C        4.567270       0.040575      -0.039007
N        5.717714       0.132761      -0.108080
C       -1.648405       1.257379      -1.591582
H       -0.595401       1.049279      -1.774777
H       -2.188776       1.060562      -2.522646
C       -1.824630       2.719005      -1.179039
H       -1.470925       3.376329      -1.977891
H       -2.873103       2.958408      -0.984135
H       -1.250784       2.943620      -0.277501

----------------------------------------
35
N-Et-B-p-CN-TS | SCF Done:  E(RB3LYP) =  -716.290760970     A.U. after    1 cycles
C       -1.942613      -1.716425       0.989023
B       -1.237559      -0.557360       0.435477
N       -2.104252       0.753584      -0.099615
C       -2.912534      -0.114268      -0.682052
C       -3.933387      -0.789296       0.163027
C       -3.388353      -1.695359       0.997676
H       -2.889469      -0.245485      -1.772835
H       -1.409956      -2.562118       1.413605
H       -3.978853      -2.275050       1.701564
H       -4.930119      -0.364661       0.220192
C        0.320519      -0.370633       0.229372
C        1.019984      -0.964266      -0.846206
C        1.040172       0.373309       1.195039
C        2.401650      -0.800810      -0.950935
C        2.420699       0.524772       1.072777
C        3.105529      -0.055858       0.000319
H        2.941411      -1.255000      -1.773708
H        2.973516       1.092252       1.812399
C        0.304874      -1.778573      -1.900608
H       -0.336245      -1.148590      -2.527526
H        1.017826      -2.277237      -2.560024
H       -0.339915      -2.534552      -1.448329
C        0.337738       0.986699       2.386390
H        1.013132       1.632255       2.951046
H       -0.531076       1.579254       2.087726
H       -0.032077       0.209836       3.061917
N        5.665512       0.245680      -0.222260
C        4.521860       0.109553      -0.121911
C       -1.438942       1.755801      -0.952852
H       -0.496829       2.031979      -0.482608
H       -1.206665       1.326499      -1.938037
C       -2.334645       2.983933      -1.112570
H       -1.824213       3.738879      -1.715788
H       -3.273902       2.730573      -1.612029
H       -2.572885       3.421655      -0.140770

----------------------------------------
35
N-Et-B-p-CN-Dewar | SCF Done:  E(RB3LYP) =  -716.327844546     A.U. after    1
cycles
C        3.158337      -0.232388      -0.739448
C        2.130711       0.642833      -1.548956
```

```
B        1.059825     -0.022386     -0.523927
N        2.069621     -0.745903      0.125479
H        3.761032     -1.002722     -1.226295
H        2.023061      0.509462     -2.626337
C        2.955522      1.820851     -1.047248
C        3.817574      1.082734     -0.333664
H        2.840263      2.889388     -1.192625
H        4.634669      1.320470      0.336648
C       -0.492787      0.015143     -0.294729
C       -1.098500      1.152340      0.285729
C       -1.302410     -1.075780     -0.686265
C       -2.477286      1.182180      0.485570
C       -2.681167     -1.024798     -0.487348
C       -3.273249      0.097589      0.101729
H       -2.943083      2.049421      0.939044
H       -3.305502     -1.858226     -0.787702
C       -0.698016     -2.303251     -1.333218
H       -1.472624     -3.013984     -1.626799
H       -0.128573     -2.042032     -2.230309
H       -0.010826     -2.817797     -0.655692
N       -5.831987      0.167470      0.477303
C       -4.688570      0.136559      0.309074
C       -0.267068      2.350173      0.686427
H        0.590668      2.061221      1.300066
H        0.128426      2.863993     -0.195413
H       -0.859123      3.071182      1.253084
C        2.209614     -1.620413      1.274828
H        1.202970     -1.840855      1.640941
H        2.649192     -2.572676      0.949988
C        3.058171     -1.026216      2.402216
H        2.625141     -0.092305      2.768653
H        3.118497     -1.728849      3.237749
H        4.077990     -0.820068      2.066965

---------------------------------------
31
N-iPr-B-Ph-aza | SCF Done:  E(RB3LYP) =  -584.829626566     A.U. after    1 cycles
C        2.683467     -0.213291     -0.055574
C        0.753737     -2.243998     -0.236577
B        0.303955     -0.801272     -0.098786
N        1.372451      0.178415      0.003025
H        3.428972      0.567792      0.025872
H        0.039372     -3.058856     -0.314608
C        2.091650     -2.543858     -0.289678
C        3.067083     -1.516778     -0.202687
H        2.434571     -3.569921     -0.401172
H        4.123982     -1.748025     -0.245886
C       -1.223523     -0.398659     -0.064311
C       -2.075058     -0.967613      0.899375
C       -1.814151      0.458242     -1.010440
C       -3.438860     -0.679708      0.933823
C       -3.179233      0.740671     -0.992405
C       -3.996525      0.176557     -0.014029
H       -4.066963     -1.128009      1.696425
H       -3.605515      1.398028     -1.742924
H       -1.201111      0.901865     -1.789591
H       -1.662030     -1.647399      1.638451
H       -5.058148      0.397339      0.005368
C        1.118778      1.630598      0.233211
H        0.035374      1.727020      0.268008
C        1.633671      2.490561     -0.926622
H        1.330861      3.530731     -0.779856
H        2.724589      2.471542     -1.000302
H        1.224631      2.147324     -1.879695
C        1.666695      2.088980      1.590310
H        1.273772      1.465544      2.396596
H        2.758757      2.049649      1.629303
H        1.365564      3.122616      1.780401
```

```
----------------------------------------
31
N-iPr-B-Ph-TS | SCF Done:  E(RB3LYP) =  -584.694236435     A.U. after    1 cycles
C        0.964227      -2.284522      -0.279392
B        0.425908      -0.917993      -0.277164
N        1.462960       0.371369      -0.320750
C        2.185074      -0.268960       0.581595
C        3.080808      -1.354875       0.093641
C        2.397866      -2.451226      -0.290519
H        2.208947       0.054792       1.627161
H        0.312490      -3.151887      -0.319817
H        2.884004      -3.337467      -0.689205
H        4.111730      -1.111577      -0.143040
C       -1.092355      -0.512938      -0.150703
C       -1.853106      -0.867647       0.976509
C       -1.766466       0.123253      -1.208571
C       -3.217796      -0.589672       1.051159
C       -3.131765       0.396840      -1.144279
C       -3.861462       0.045706      -0.009232
H       -3.626881       0.878430      -1.980851
H       -3.779359      -0.875052       1.934441
H       -4.923416       0.259042       0.044755
H       -1.369187      -1.376608       1.803519
H       -1.219867       0.388549      -2.108842
C        0.977575       1.755278      -0.069105
H        0.036353       1.837144      -0.610716
C        0.718115       2.112814       1.398549
H        1.637950       2.148212       1.989892
H        0.270127       3.108395       1.443829
H        0.022728       1.413211       1.866899
C        1.989065       2.711316      -0.716292
H        2.141360       2.463569      -1.768356
H        1.626454       3.740534      -0.649499
H        2.957638       2.658884      -0.209259

----------------------------------------
31
N-iPr-B-Ph-Dewar | SCF Done:  E(RB3LYP) =  -584.737037743     A.U. after    1
cycles
C        2.123224       0.921342      -0.622786
C        0.852253       1.838307      -0.769446
B        0.070108       0.487656      -0.341509
N        1.276344      -0.234576      -0.237745
H        2.794810       0.751701      -1.467076
H        0.641314       2.329839      -1.720572
C        1.491346       2.660575       0.341667
C        2.561944       1.867979       0.492449
H        1.152139       3.551412       0.859603
H        3.389743       1.848878       1.191133
C       -1.427351       0.117600      -0.136644
C       -1.905625      -1.205415      -0.088387
C       -2.373384       1.149871      -0.000104
C       -3.257490      -1.483805       0.095958
C       -3.726192       0.880218       0.194501
C       -4.171369      -0.440004       0.242912
H       -3.600883      -2.512391       0.123774
H       -4.433201       1.695748       0.302124
H       -5.224454      -0.655181       0.388177
H       -2.038676       2.181309      -0.047569
H       -1.214627      -2.032393      -0.210791
C        1.745924      -1.540683       0.212060
H        0.865401      -2.061423       0.600226
C        2.315664      -2.358946      -0.956273
H        2.614691      -3.356121      -0.620750
H        1.574325      -2.469736      -1.751117
H        3.198506      -1.871319      -1.380938
C        2.760531      -1.413511       1.356267
H        2.342587      -0.843722       2.189031
H        3.044126      -2.403642       1.722995
```

```
H        3.672393      -0.910376       1.020470

----------------------------------------
34
N-iPr-B-o-Tol-aza | SCF Done:  E(RB3LYP) =  -624.155074672     A.U. after    1
cycles
C        2.780118      -0.128705      -0.078381
C        0.899810      -2.178420      -0.477287
B        0.415955      -0.758680      -0.251026
N        1.459115       0.230561      -0.048333
H        3.504788       0.661546       0.073446
H        0.207524      -2.999443      -0.642018
C        2.245510      -2.448797      -0.489910
C        3.195506      -1.413841      -0.288251
H        2.614411      -3.458232      -0.656985
H        4.257580      -1.624201      -0.299304
C       -1.114801      -0.348536      -0.269770
C       -1.950025      -0.474873       0.863459
C       -1.692867       0.101035      -1.468189
C       -3.305230      -0.142946       0.763020
C       -3.044862       0.429666      -1.559603
C       -3.855451       0.308708      -0.435009
H       -3.941086      -0.243738       1.637483
H       -3.460176       0.771569      -2.501500
H       -1.072641       0.189031      -2.355845
H       -4.909807       0.557590      -0.488311
C       -1.395702      -0.959612       2.185324
H       -0.857927      -1.905058       2.071333
H       -0.686644      -0.241547       2.611402
H       -2.192536      -1.109007       2.917167
C        1.154702       1.671392       0.190952
H        0.066982       1.726187       0.217150
C        1.683650       2.149642       1.548402
H        1.327454       3.164133       1.745710
H        1.331613       1.502418       2.354850
H        2.776381       2.171825       1.582571
C        1.642413       2.549038      -0.967287
H        2.733022       2.547448      -1.049991
H        1.228382       2.203695      -1.917141
H        1.322474       3.582741      -0.811378

----------------------------------------
34
N-iPr-B-o-Tol-TS | SCF Done:  E(RB3LYP) =  -624.012536132     A.U. after    1
cycles
C        1.350292      -1.886327      -0.845731
B        0.713069      -0.606860      -0.496189
N        1.658971       0.705085      -0.191853
C        2.409916      -0.083861       0.555902
C        3.385955      -0.965421      -0.155216
C        2.791390      -1.956437      -0.845430
H        2.440364       0.030098       1.646438
H        0.759626      -2.756503      -1.116582
H        3.346922      -2.668873      -1.449232
H        4.400407      -0.599716      -0.286104
C       -0.851511      -0.406163      -0.349395
C       -1.608150      -0.884671       0.746399
C       -1.545165       0.146176      -1.438849
C       -3.001947      -0.758794       0.724186
C       -2.934440       0.256418      -1.455355
C       -3.666952      -0.190361      -0.360115
H       -3.437072       0.682129      -2.317055
H       -3.577495      -1.124420       1.569073
H       -4.748588      -0.110148      -0.351968
H       -0.983638       0.482540      -2.304849
C        1.214704       2.030456       0.341431
C        0.384627       1.997849       1.631540
H        0.269696       3.019963       2.002430
H       -0.608261       1.584558       1.455582
```

```
H        0.870056       1.421252       2.422909
C        0.541054       2.845901      -0.761505
H        1.138633       2.823684      -1.675350
H       -0.456846       2.470043      -0.986399
H        0.446225       3.886199      -0.438534
C       -0.950029      -1.546558       1.935526
H       -0.350997      -2.406623       1.626837
H       -0.272563      -0.863763       2.458292
H       -1.697031      -1.886939       2.655984
H        2.163947       2.525975       0.572126

----------------------------------------
34
N-iPr-B-o-Tol-Dewar | SCF Done:  E(RB3LYP) =  -624.058617030     A.U. after    1
cycles
C        2.410413      -1.035695       0.372297
C        1.191816      -1.948238      -0.006547
B        0.358573      -0.561039       0.126957
N        1.524668       0.146632       0.486197
H        3.025134      -1.230927       1.254690
H        0.953824      -2.819792       0.604001
C        1.936199      -2.166436      -1.317668
C        2.969727      -1.368895      -1.010507
H        1.675973      -2.733163      -2.205044
H        3.840851      -1.025849      -1.556204
C       -1.120494      -0.122380      -0.130899
C       -1.388727       0.842394      -1.116756
C       -2.216373      -0.706593       0.545993
C       -2.689062       1.228066      -1.437391
C       -3.515483      -0.300320       0.229516
C       -3.758365       0.655857      -0.755553
H       -2.863332       1.970498      -2.208546
H       -4.352524      -0.741788       0.761737
H       -4.776671       0.949580      -0.986138
H       -0.561179       1.294486      -1.654561
C        1.991870       1.500580       0.776632
C        2.567537       2.186316      -0.472179
H        1.784005       2.353683      -1.216514
H        3.003444       3.154876      -0.210151
H        3.348778       1.578593      -0.934365
C        0.894404       2.340668       1.433639
H        0.050867       2.494446       0.757667
H        0.519647       1.853538       2.336826
H        1.290692       3.321145       1.711282
C       -2.003102      -1.758601       1.611690
H       -1.591643      -2.677899       1.183703
H       -1.301973      -1.419803       2.380424
H       -2.942435      -2.013544       2.106831
H        2.808850       1.390355       1.502588

----------------------------------------
37
N-iPr-B-Xylyl-aza | SCF Done:  E(RB3LYP) =  -663.480087610     A.U. after    1
cycles
C       -2.832317      -0.153402      -0.000635
C       -0.921445      -2.211454      -0.015953
B       -0.455542      -0.766473      -0.005671
N       -1.516383       0.226378       0.001848
H       -3.569261       0.640118       0.005265
H       -0.216249      -3.038241      -0.022138
C       -2.262856      -2.500936      -0.017770
C       -3.229001      -1.461115      -0.010020
H       -2.616112      -3.529616      -0.025147
H       -4.288185      -1.685802      -0.011412
C        1.077617      -0.353616      -0.002786
C        1.782085      -0.194608      -1.217739
C        1.782426      -0.215088       1.214626
C        3.144979       0.118984      -1.200879
C        3.145164       0.098968       1.202746
```

```
C        3.825784       0.271496       0.002176
H        3.676895       0.235607      -2.140020
H        3.677266       0.200022       2.143574
H        4.883705       0.511239       0.004089
C        1.082687      -0.361849      -2.550250
H        0.480543      -1.274053      -2.577355
H        0.405086       0.473011      -2.758661
H        1.804178      -0.407939      -3.368944
C        1.083380      -0.406814       2.544001
H        0.389735       0.413093       2.758425
H        0.498017      -1.330218       2.561201
H        1.803824      -0.447448       3.363876
C       -1.238618       1.692386       0.012180
H       -0.151540       1.769752       0.012802
C       -1.763863       2.380857      -1.253629
H       -1.432739       3.422522      -1.272619
H       -1.389441       1.888145      -2.153111
H       -2.856665       2.380780      -1.296295
C       -1.763948       2.363150       1.287416
H       -2.856741       2.362389       1.330115
H       -1.389454       1.858121       2.180013
H       -1.432911       3.404476       1.320848

----------------------------------------
37
N-iPr-B-Xylyl-TS | SCF Done:  E(RB3LYP) =  -663.336713799     A.U. after    1
cycles
C        1.427621      -1.838607      -0.856618
B        0.759554      -0.606245      -0.409250
N        1.678000       0.730224      -0.074194
C        2.492114      -0.076784       0.583533
C        3.461553      -0.874936      -0.225267
C        2.867745      -1.853803      -0.933819
H        2.569976      -0.036001       1.676455
H        0.855396      -2.715332      -1.146234
H        3.415421      -2.513794      -1.601114
H        4.452017      -0.460746      -0.390040
C       -0.802374      -0.463538      -0.177063
C       -1.441712      -0.952720       0.988637
C       -1.598987       0.049898      -1.225416
C       -2.830408      -0.854053       1.112242
C       -2.987945       0.135382      -1.071709
C       -3.602599      -0.300191       0.094964
H       -3.588764       0.539625      -1.880760
H       -3.311785      -1.223767       2.012065
H       -4.678948      -0.224978       0.206893
C        1.220423       2.002220       0.572166
C        0.528549       1.866466       1.934916
H        0.403238       2.864168       2.364178
H       -0.456954       1.411795       1.836191
H        1.116511       1.283645       2.647959
C        0.396828       2.840002      -0.403832
H        0.900916       2.918062      -1.368959
H       -0.596085       2.417589      -0.557607
H        0.275727       3.848644       0.000457
C       -0.660345      -1.619534       2.099151
H       -0.114455      -2.489769       1.726160
H        0.083245      -0.951076       2.542627
H       -1.327190      -1.949182       2.898932
H        2.163939       2.535143       0.733069
C       -0.997939       0.447898      -2.557388
H       -1.043751      -0.392458      -3.258607
H       -1.548396       1.278767      -3.007245
H        0.051030       0.733126      -2.475799

----------------------------------------
37
N-iPr-B-Xylyl-Dewar | SCF Done:  E(RB3LYP) =  -663.384729465     A.U. after    1
cycles
```

```
C       -2.340713      -0.139077      -1.039751
C       -1.141414      -0.830160      -1.787875
B       -0.264439      -0.167794      -0.589414
N       -1.412773       0.399099      -0.014016
H       -2.967591       0.600743      -1.541291
H       -0.932453      -0.591046      -2.831653
C       -1.879755      -2.125825      -1.481270
C       -2.890493      -1.546255      -0.817437
H       -1.630756      -3.159849      -1.694793
H       -3.740832      -1.925893      -0.263678
C        1.247640      -0.100553      -0.176135
C        1.795076      -1.046436       0.718130
C        2.086535       0.897272      -0.720061
C        3.149235      -0.978639       1.058227
C        3.436690       0.942193      -0.361902
C        3.968604       0.010635       0.524074
H        3.563788      -1.709986       1.744938
H        4.075383       1.711831      -0.783761
H        5.018379       0.052895       0.793736
C       -1.755202       1.146204       1.191304
C       -2.330070       2.527458       0.847468
H       -3.276697       2.435945       0.305925
H       -2.522413       3.101358       1.758194
H       -1.634640       3.095209       0.224998
C       -2.707700       0.354444       2.097604
H       -3.674187       0.196324       1.609181
H       -2.290477      -0.623215       2.348695
H       -2.890974       0.900058       3.027215
C        1.538441       1.920303      -1.691772
H        1.097813       1.440897      -2.571895
H        0.754409       2.531372      -1.233697
H        2.324123       2.593858      -2.040249
H       -0.808507       1.289834       1.723019
C        0.930721      -2.136211       1.314901
H        0.157188      -1.722265       1.969726
H        0.416371      -2.712478       0.540228
H        1.528108      -2.832436       1.907311

----------------------------------------
40
N-iPr-B-Mes-aza | SCF Done:  E(RB3LYP) =  -702.807408522     A.U. after    1 cycles
C       -3.181478      -0.052707      -0.000515
C       -1.364254      -2.193375       0.023668
B       -0.833216      -0.770609       0.008495
N       -1.850000       0.268086      -0.003387
H       -3.882370       0.772852      -0.009787
H       -0.696461      -3.050650       0.033454
C       -2.717137      -2.423087       0.025568
C       -3.636162      -1.341364       0.013306
H       -3.115842      -3.435030       0.036454
H       -4.704321      -1.518640       0.014721
C        0.715817      -0.426147       0.005175
C        1.431559      -0.321373      -1.208214
C        1.430813      -0.290593       1.215357
C        2.805806      -0.067262      -1.193921
C        2.805585      -0.037201       1.195104
C        3.513934       0.080708      -0.000751
H        3.337557       0.009626      -2.138675
H        3.336984       0.063356       2.137796
C        0.729906      -0.480190      -2.540770
H        0.089912      -1.366635      -2.554873
H        0.088236       0.378771      -2.764563
H        1.450630      -0.569946      -3.356540
C        0.729019      -0.412481       2.551760
H        0.112236       0.467236       2.765309
H        0.065311      -1.280799       2.580228
H        1.449550      -0.510080       3.366850
C       -1.507507       1.720060      -0.018467
H       -0.418054       1.748565      -0.018957
```

```
C       -2.002223       2.409304      -1.296106
H       -1.625066       3.434738      -1.333101
H       -1.650500       1.884755      -2.186715
H       -3.093982       2.457154      -1.339226
C       -2.001955       2.435215       1.244967
H       -3.093721       2.483759       1.287285
H       -1.650027       1.928808       2.145929
H       -1.624928       3.461242       1.261016
C        5.005621       0.316809      -0.004289
H        5.326098       0.858103       0.889268
H        5.554194      -0.631590      -0.026404
H        5.316574       0.892951      -0.879459
```

```
40
N-iPr-B-Mes-TS | SCF Done:  E(RB3LYP) =  -702.671803099     A.U. after    1 cycles
C        1.570725      -2.043645      -0.845350
B        0.933612      -0.778931      -0.457636
N        1.896918       0.547730      -0.131363
C        2.646449      -0.272597       0.581591
C        3.615071      -1.120613      -0.169911
C        3.011609      -2.105404      -0.865448
H        2.642450      -0.251012       1.675935
H        0.985020      -2.906900      -1.148061
H        3.561223      -2.806180      -1.488231
H        4.623960      -0.747951      -0.317682
C       -0.603958      -0.483212      -0.254128
C       -1.331594      -0.982995       0.850773
C       -1.304303       0.237754      -1.248969
C       -2.701179      -0.723706       0.957299
C       -2.674591       0.475231      -1.109798
C       -3.391531       0.012257      -0.006634
H       -3.195237       1.027600      -1.887432
H       -3.244984      -1.114079       1.813170
C       -0.670983      -1.827591       1.918874
H       -0.241623      -2.737827       1.493038
H        0.152574      -1.302572       2.412804
H       -1.391087      -2.114644       2.688320
C       -0.608326       0.706463      -2.509566
H       -0.439249      -0.133870      -3.190933
H       -1.210967       1.448019      -3.039051
H        0.372850       1.142377      -2.308074
C       -4.863191       0.311509       0.149146
H       -5.018495       1.236964       0.715196
H       -5.349673       0.437149      -0.821150
H       -5.378375      -0.488740       0.686219
C        1.324475       1.790216       0.451637
H        0.346086       1.909646      -0.012896
C        1.135916       1.792340       1.971608
H        0.497128       0.972140       2.303326
H        2.087185       1.741553       2.510194
H        0.651131       2.726932       2.263835
C        2.222551       2.949660      -0.003933
H        2.329391       2.957175      -1.090069
H        1.790178       3.903594       0.310014
H        3.221181       2.867172       0.436104
```

```
40
N-iPr-B-Mes-Dewar | SCF Done:  E(RB3LYP) =  -702.712148371     A.U. after    1
cycles
C       -2.748022      -0.114508      -0.954267
C       -1.592163      -0.789748      -1.780709
B       -0.649791      -0.155079      -0.616626
N       -1.765243       0.400873       0.030997
H       -3.400410       0.635863      -1.405158
H       -1.439761      -0.528396      -2.828965
C       -2.314508      -2.091609      -1.462542
C       -3.287054      -1.526184      -0.732622
```

H	-2.078612	-3.120957	-1.710894
H	-4.106322	-1.917510	-0.141363
C	0.881597	-0.101663	-0.284349
C	1.471101	-1.040154	0.588657
C	1.705484	0.879531	-0.877302
C	2.841992	-0.982307	0.852978
C	3.071712	0.911200	-0.589777
C	3.662246	-0.014113	0.272675
H	3.280000	-1.712903	1.527748
H	3.690632	1.675580	-1.051890
C	-2.041478	1.122948	1.267918
C	-2.624291	2.514432	0.983670
H	-3.596895	2.439517	0.487465
H	-2.768231	3.068898	1.915184
H	-1.956783	3.092236	0.340239
C	-2.951945	0.316320	2.203814
H	-3.942785	0.174169	1.761403
H	-2.528725	-0.668878	2.411599
H	-3.084752	0.842012	3.153305
C	1.121545	1.905597	-1.824852
H	0.630239	1.427508	-2.678458
H	0.369395	2.528241	-1.330129
H	1.896167	2.567989	-2.216856
H	-1.068141	1.250144	1.753767
C	0.632883	-2.110218	1.255203
H	-0.043215	-1.681234	2.002294
H	0.010369	-2.645359	0.532586
H	1.261923	-2.844368	1.763236
C	5.147981	0.011095	0.541385
H	5.382181	-0.397568	1.527419
H	5.691674	-0.587486	-0.198216
H	5.545236	1.028058	0.492553

46

N-iPr-B-pentamethylphenyl-aza | SCF Done: E(RB3LYP) = -781.449334703   A.U. after   1 cycles

C	3.569921	-0.000082	0.025142
C	1.830035	-0.002633	-2.179853
B	1.246851	-0.000787	-0.776941
N	2.227561	0.000389	0.297085
H	4.240478	0.000893	0.875645
H	1.193167	-0.003694	-3.060273
C	3.189928	-0.002986	-2.360671
C	4.070515	-0.001683	-1.246383
H	3.624899	-0.004282	-3.357694
H	5.144303	-0.001953	-1.385633
C	-0.312357	-0.000449	-0.471935
C	-1.016680	-1.217260	-0.361520
C	-1.016656	1.216587	-0.363786
C	-2.401202	-1.219386	-0.111831
C	-2.401158	1.219193	-0.113941
C	-3.084320	0.000050	0.044673
C	-0.275549	-2.533884	-0.511316
H	-0.626583	-3.099618	-1.381232
H	0.794642	-2.377368	-0.647904
H	-0.406792	-3.179776	0.363185
C	-0.275470	2.532902	-0.516113
H	0.794586	2.376042	-0.653350
H	-0.627142	3.097419	-1.386551
H	-0.405898	3.180091	0.357561
C	-4.562872	0.000343	0.378666
H	-5.185170	-0.001008	-0.525620
H	-4.845025	-0.876382	0.962358
H	-4.845261	0.878615	0.959894
C	-3.165780	-2.524628	-0.007690
H	-2.611329	-3.358251	-0.435060
H	-3.385895	-2.785071	1.035257
H	-4.123227	-2.471628	-0.531342

C	-3.165738	2.524601	-0.011875
H	-3.387051	2.785967	1.030579
H	-2.610758	3.357814	-0.439328
H	-4.122603	2.471184	-0.536579
C	1.829706	0.002158	1.734746
H	0.740305	0.002429	1.720948
C	2.292904	-1.270419	2.453935
H	1.887826	-1.289878	3.469268
H	1.943637	-2.163130	1.930804
H	3.382493	-1.325020	2.531879
C	2.293566	1.276151	2.450989
H	3.383181	1.330320	2.528877
H	1.944835	2.167830	1.925748
H	1.888434	1.298210	3.466246

46

N-iPr-B-pentamethylphenyl-TS | SCF Done: E(RB3LYP) = -781.305852054   A.U. after   1 cycles

C	-2.105489	0.096371	-2.034017
B	-1.451582	-0.068519	-0.723139
N	-2.395355	-0.374497	0.606694
C	-3.210411	0.566876	0.160732
C	-4.160410	0.189088	-0.925159
C	-3.542141	0.017375	-2.110309
H	-3.293626	1.533755	0.670818
H	-1.517190	0.239033	-2.936079
H	-4.068372	-0.282362	-3.012730
H	-5.144231	-0.175956	-0.645132
C	0.112453	0.025873	-0.467643
C	0.796355	1.254443	-0.339678
C	0.853785	-1.176564	-0.501579
C	2.193292	1.273827	-0.169962
C	2.251859	-1.156127	-0.347277
C	2.912580	0.065852	-0.131210
C	-1.953554	-0.338904	2.038112
C	-1.279005	0.955939	2.511104
H	-1.181231	0.922875	3.599705
H	-0.283723	1.061304	2.080500
H	-1.861575	1.847531	2.267411
C	-1.120173	-1.572783	2.378078
H	-1.611541	-2.480909	2.023798
H	-0.124806	-1.516469	1.937442
H	-1.007917	-1.644990	3.463420
C	0.036082	2.565408	-0.393059
H	-1.033143	2.397141	-0.505950
H	0.196280	3.173254	0.503564
H	0.349328	3.171081	-1.250154
H	-2.901535	-0.434356	2.578914
C	0.147858	-2.500240	-0.733749
H	0.418476	-2.926291	-1.706166
H	0.408001	-3.243061	0.026381
H	-0.935354	-2.385398	-0.730806
C	2.937271	2.587972	-0.031186
H	2.350384	3.430742	-0.391412
H	3.196516	2.799551	1.013913
H	3.872403	2.581100	-0.595786
C	4.406103	0.084249	0.126305
H	4.979804	0.181149	-0.804466
H	4.693457	0.918274	0.767342
H	4.741915	-0.829809	0.616566
C	3.052850	-2.441997	-0.414582
H	3.998744	-2.298184	-0.940834
H	3.296300	-2.824577	0.584789
H	2.512208	-3.230288	-0.935740

46

N-iPr-B-pentamethylphenyl-Dewar | SCF Done: E(RB3LYP) = -781.354321242   A.U. after   1 cycles

C	-3.173276	0.288851	-0.904290
C	-2.035682	-0.022299	-1.946229
B	-1.068638	0.113555	-0.643562
N	-2.171602	0.381089	0.185978
H	-3.824725	1.157422	-1.020838
H	-1.892346	0.620516	-2.816260
C	-2.767570	-1.342607	-2.141230
C	-3.724385	-1.097042	-1.234005
H	-2.544641	-2.199116	-2.768577
H	-4.539083	-1.682753	-0.824896
C	0.469009	0.031962	-0.336876
C	1.056500	-1.191425	0.041429
C	1.272786	1.183095	-0.457675
C	2.437415	-1.265907	0.294880
C	2.654129	1.112753	-0.206834
C	3.237592	-0.118666	0.143676
C	-2.419220	0.568151	1.610850
C	-3.014676	1.953920	1.896656
H	-4.000501	2.061748	1.433536
H	-3.134992	2.106611	2.972859
H	-2.366744	2.742987	1.508074
C	-3.299745	-0.549746	2.186037
H	-4.299857	-0.530475	1.741987
H	-2.861385	-1.531760	1.995196
H	-3.414135	-0.427527	3.266673
C	0.641397	2.498491	-0.874969
H	-0.417033	2.377040	-1.109725
H	0.707403	3.253849	-0.084069
H	1.125659	2.919002	-1.761796
H	-1.433797	0.511806	2.085407
C	0.192470	-2.433311	0.158444
H	-0.836054	-2.236736	-0.146710
H	0.566672	-3.248560	-0.468659
H	0.156076	-2.811214	1.186380
C	3.072575	-2.573471	0.725215
H	2.333048	-3.288300	1.082108
H	3.612619	-3.053742	-0.100213
H	3.792777	-2.423289	1.533037
C	4.735269	-0.209510	0.357817
H	5.009999	-0.009446	1.401617
H	5.119539	-1.199188	0.109751
H	5.275637	0.509366	-0.258689
C	3.521787	2.351253	-0.314222
H	4.235118	2.414565	0.510979
H	4.105395	2.358895	-1.243200
H	2.931497	3.265951	-0.300053

41

N-iPr-B-p-OMe-aza | SCF Done: E(RB3LYP) = -778.036358741   A.U. after   1 cycles

C	3.496041	0.039517	-0.135721
C	1.741520	-2.128465	-0.458789
B	1.171208	-0.754153	-0.153855
N	2.158686	0.302965	-0.003707
H	4.173010	0.875674	-0.011275
H	1.098552	-2.994406	-0.592034
C	3.097865	-2.302139	-0.574715
C	3.985306	-1.206514	-0.411156
H	3.523410	-3.278993	-0.793425
H	5.056121	-1.339289	-0.501397
C	-0.382601	-0.473314	-0.002459
C	-1.028230	-0.636348	1.249389
C	-1.174293	-0.132695	-1.114481
C	-2.399759	-0.438225	1.369373
C	-2.557750	0.062051	-0.982316
C	-3.169893	-0.084159	0.259098

```
H       -2.899968      -0.565703       2.322966
H       -3.136839       0.317486      -1.860698
C       -0.240231      -1.038107       2.478055
H        0.351574      -1.939377       2.294601
H        0.462254      -0.254704       2.780362
H       -0.901624      -1.232670       3.324733
C       -0.559303       0.048196      -2.487157
H       -0.148781       1.056940      -2.610359
H        0.257380      -0.656196      -2.660587
H       -1.302702      -0.095409      -3.275017
O       -4.508642       0.083982       0.490946
C       -5.347212       0.428846      -0.600832
H       -5.334677      -0.344462      -1.377097
H       -6.352933       0.509888      -0.190675
H       -5.058216       1.389454      -1.042044
C        1.776908       1.713880       0.294481
H        0.689162       1.698665       0.359599
C        2.327939       2.181352       1.647172
H        1.927011       3.169427       1.888225
H        2.042272       1.494466       2.446408
H        3.418743       2.260049       1.640679
C        2.172606       2.662325      -0.843966
H        3.257933       2.760602      -0.936625
H        1.781270       2.310350      -1.800556
H        1.765852       3.659158      -0.653977

---------------------------------------
41
N-iPr-B-p-OMe-TS | SCF Done:  E(RB3LYP) =  -777.901038941     A.U. after    1
cycles
C       -1.957996      -1.928859       1.011776
B       -1.264011      -0.733098       0.517542
N       -2.169861       0.631060       0.147784
C       -2.995677      -0.186932      -0.477676
C       -3.974949      -0.926038       0.368245
C       -3.397344      -1.902196       1.097359
H       -3.037571      -0.239024      -1.570178
H       -1.410229      -2.805278       1.346400
H       -3.956587      -2.529160       1.786727
H       -4.951504      -0.483399       0.538467
C        0.273942      -0.541326       0.227903
C        0.923208      -1.163614      -0.870559
C        1.059924       0.206880       1.127653
C        2.291079      -0.997621      -1.062253
C        2.438420       0.361615       0.922978
C        3.053100      -0.232618      -0.175797
H        2.795633      -1.464289      -1.900759
H        3.011855       0.940702       1.635608
C        0.164413      -2.040127      -1.842847
H       -0.285323      -2.894976      -1.331863
H       -0.658509      -1.504518      -2.325813
H        0.825360      -2.415508      -2.626801
C        0.458811       0.809561       2.380743
H       -0.566802       1.150474       2.229104
H        0.424589       0.064803       3.183209
H        1.053918       1.653172       2.739567
O        4.384933      -0.133423      -0.468165
C        5.218295       0.614876       0.404744
H        6.220677       0.555481      -0.016795
H        4.908134       1.664599       0.454068
H        5.224762       0.190575       1.414806
C       -1.554607       1.795830      -0.541934
H       -0.555395       1.893449      -0.118567
C       -1.419761       1.680387      -2.063278
H       -0.897424       2.563982      -2.437907
H       -0.836533       0.805089      -2.353826
H       -2.390633       1.643352      -2.566829
C       -2.373386       3.032306      -0.142983
H       -2.440324       3.122432       0.942660
```

```
H       -1.903060       3.936389      -0.539180
H       -3.390250       2.974366      -0.543426

----------------------------------------
41
N-iPr-B-p-OMe-Dewar | SCF Done:  E(RB3LYP) =  -777.941081213     A.U. after    1
cycles
C       -3.075305       0.127587      -0.970271
C       -1.939075      -0.445193      -1.894705
B       -0.982600      -0.055474      -0.637513
N       -2.082242       0.431557       0.089551
H       -3.695223       0.967402      -1.290413
H       -1.770074      -0.019585      -2.884939
C       -2.712306      -1.753471      -1.798934
C       -3.669586      -1.279541      -0.988111
H       -2.512263      -2.736001      -2.213035
H       -4.508419      -1.731379      -0.472147
C        0.543932      -0.131540      -0.292387
C        1.061136      -1.159653       0.531888
C        1.441322       0.813381      -0.824800
C        2.423618      -1.222079       0.806366
C        2.810198       0.744090      -0.535876
C        3.302394      -0.273387       0.279659
H        2.830376      -2.009339       1.431234
H        3.473611       1.488039      -0.957815
C        0.943418       1.927425      -1.721538
H        1.760608       2.578398      -2.039201
H        0.466748       1.528846      -2.622611
H        0.200028       2.549739      -1.213629
O        4.619344      -0.429006       0.615877
C        5.565240       0.497006       0.103077
H        5.353789       1.517108       0.442727
H        6.532183       0.181841       0.492715
H        5.592978       0.477820      -0.992115
C       -2.342460       0.957369       1.424877
H       -1.375959       0.936905       1.939711
C       -2.824799       2.413852       1.366451
H       -2.101930       3.041587       0.840310
H       -3.785237       2.489466       0.847188
H       -2.959230       2.815705       2.374648
C       -3.328610       0.073064       2.200018
H       -2.978312      -0.960320       2.247573
H       -3.451075       0.443074       3.221563
H       -4.314809       0.075877       1.725469
C        0.145626      -2.206903       1.129353
H       -0.498464      -1.776937       1.903290
H       -0.512168      -2.646432       0.374538
H        0.718325      -3.015343       1.588227

----------------------------------------
38
N-iPr-B-p-CN-aza | SCF Done:  E(RB3LYP) =  -755.749400067     A.U. after    1
cycles
C        3.366557      -0.001733      -0.000035
C        1.586912      -2.175777       0.001470
B        1.037840      -0.762841       0.000601
N        2.029351       0.295691      -0.000198
H        4.052355       0.836111      -0.000626
H        0.935360      -3.045325       0.002044
C        2.944490      -2.380318       0.001569
C        3.843205      -1.282582       0.000824
H        3.360805      -3.384871       0.002216
H        4.914163      -1.441091       0.000898
C       -0.521552      -0.451995       0.000353
C       -1.233180      -0.352334       1.218227
C       -1.233207      -0.354394      -1.217671
C       -2.610098      -0.131704       1.209341
C       -2.610126      -0.133756      -1.209132
C       -3.302992      -0.014816       0.000012
```

```
H       -3.155206      -0.055518       2.143089
H       -3.155255      -0.059157      -2.142996
C       -0.523025      -0.488503       2.547526
H        0.093516      -1.390673       2.578885
H        0.143015       0.359257       2.735840
H       -1.236621      -0.535770       3.372275
C       -0.523079      -0.492827      -2.546749
H        0.142910       0.354643      -2.736546
H        0.093507      -1.395018      -2.576565
H       -1.236693      -0.541557      -3.371398
C       -4.715455       0.214460      -0.000166
N       -5.856284       0.402419      -0.000312
C        1.661294       1.742807      -0.001179
H        0.571360       1.754656      -0.001081
C        2.143979       2.454237       1.268715
H        1.749116       3.473112       1.293999
H        1.802480       1.934718       2.166226
H        3.234531       2.521637       1.310207
C        2.143738       2.452473      -1.272154
H        3.234280       2.519816      -1.313950
H        1.802067       1.931709      -2.168877
H        1.748872       3.471313      -1.298780

---------------------------------------
38
N-iPr-B-p-CN-TS | SCF Done:  E(RB3LYP) =  -755.611707796     A.U. after    1 cycles
C       -1.729284      -2.189918      -0.070950
B       -1.131639      -0.861243       0.075473
N       -2.111207       0.459712       0.286939
C       -2.866663      -0.016674      -0.683437
C       -3.803158      -1.130269      -0.349396
C       -3.172124      -2.289043      -0.088538
H       -2.906708       0.473677      -1.663214
H       -1.121434      -3.085400      -0.157123
H       -3.702490      -3.194063       0.194225
H       -4.835306      -0.881308      -0.124258
C        0.414878      -0.523727      -0.032611
C        1.033251      -0.169926      -1.255184
C        1.217758      -0.674687       1.123534
C        2.407022       0.066816      -1.299559
C        2.589947      -0.432794       1.058361
C        3.188752      -0.052849      -0.146256
H        2.881331       0.337871      -2.235643
H        3.203083      -0.541287       1.945611
C        0.243913      -0.066516      -2.541467
H       -0.413861       0.810063      -2.550415
H        0.910283       0.023140      -3.401475
H       -0.389099      -0.945059      -2.684297
C        0.627801      -1.137860       2.438351
H        1.288725      -0.891510       3.272374
H       -0.350924      -0.693999       2.630662
H        0.483785      -2.222525       2.435381
N        5.732396       0.409112      -0.246722
C        4.595734       0.202607      -0.202149
C       -1.571133       1.850379       0.201717
H       -0.816182       1.895513      -0.591268
C       -2.704582       2.827240      -0.133706
H       -2.313289       3.846661      -0.155353
H       -3.155519       2.629862      -1.108798
H       -3.490097       2.780520       0.626091
C       -0.919928       2.221652       1.532945
H       -1.651417       2.161907       2.343250
H       -0.082437       1.567753       1.769454
H       -0.541665       3.246041       1.485987

---------------------------------------
38
N-iPr-B-p-CN-Dewar | SCF Done:  E(RB3LYP) =  -755.652509755     A.U. after    1
cycles
```

```
C        3.046721      -0.515867       0.838168
C        1.918441      -1.587193       1.051672
B        0.963034      -0.405222       0.479067
N        2.056098       0.464833       0.327775
H        3.657788      -0.156763       1.669623
H        1.740861      -2.011575       2.040747
C        2.702756      -2.413380       0.042421
C        3.654660      -1.492049      -0.165638
H        2.514613      -3.393337      -0.382623
H        4.499835      -1.425380      -0.839914
C       -0.582858      -0.261391       0.238062
C       -1.154436      -0.615793      -1.004099
C       -1.423745       0.168810       1.288999
C       -2.532558      -0.520058      -1.188477
C       -2.799571       0.259496       1.087286
C       -3.359626      -0.080442      -0.149099
H       -2.974020      -0.788800      -2.141190
H       -3.446890       0.592586       1.890252
C       -0.848229       0.528601       2.641023
H       -1.630248       0.849661       3.331407
H       -0.335699      -0.326232       3.093411
H       -0.115616       1.337502       2.564176
N       -5.915383       0.095851      -0.508975
C       -4.773216       0.016292      -0.347973
C       -0.286418      -1.097445      -2.145244
H        0.395960      -0.313104      -2.487244
H        0.331947      -1.948091      -1.845429
H       -0.891869      -1.407513      -2.998870
C        2.383762       1.802102      -0.161845
H        2.909954       2.318578       0.653927
C        3.336488       1.740388      -1.363873
H        2.861574       1.236061      -2.210170
H        3.614840       2.750416      -1.676477
H        4.255631       1.202722      -1.119872
C        1.119073       2.596584      -0.486538
H        0.561604       2.136532      -1.306338
H        0.455040       2.664109       0.377318
H        1.387043       3.612009      -0.788750

----------------------------------------
34
N-tBu-B-Ph-aza | SCF Done:  E(RB3LYP) =  -624.139904363     A.U. after    1 cycles
C        2.664386       0.620465       0.000031
C        0.486289       2.361983       0.000389
B        0.238697       0.865332       0.000179
N        1.431937       0.015070       0.000004
H        3.528523      -0.022047      -0.000118
H       -0.345153       3.061094       0.000523
C        1.762614       2.862502       0.000413
C        2.862363       1.974479       0.000229
H        1.954031       3.933051       0.000564
H        3.880625       2.343114       0.000235
C       -1.278820       0.390563       0.000134
C       -2.005035       0.268888      -1.197200
C       -2.005067       0.268403       1.197392
C       -3.373967       0.002011      -1.202414
C       -3.374003       0.001526       1.202455
C       -4.064196      -0.138851      -0.000016
H       -3.901989      -0.089233      -2.145843
H       -3.902052      -0.090094       2.145833
H       -1.494585       0.390810       2.148037
H       -1.494517       0.391669      -2.147777
H       -5.128851      -0.345163      -0.000073
C        1.394609      -1.511113      -0.000244
C        2.798564      -2.145353      -0.000833
H        2.669844      -3.229395      -0.001064
H        3.378021      -1.888682      -0.890855
H        3.378574      -1.889137       0.888960
C        0.671458      -1.996415      -1.269250
```

```
H       -0.368852      -1.680607      -1.290792
H        1.171395      -1.615321      -2.163923
H        0.691643      -3.089039      -1.308176
C        0.672299      -1.996852       1.269076
H        1.172809      -1.616038       2.163546
H       -0.368004      -1.681072       1.291390
H        0.692526      -3.089489       1.307636
```

```
34
N-tBu-B-Ph-TS | SCF Done:  E(RB3LYP) =  -624.011919248     A.U. after    1 cycles
C        0.613333      -2.459809      -0.277737
B        0.265171      -1.033325      -0.227711
N        1.463204       0.089614      -0.227200
C        2.069761      -0.630595       0.698024
C        2.812225      -1.846974       0.238244
C        2.009997      -2.821021      -0.227650
H        2.180810      -0.260110       1.724040
H       -0.151223      -3.228087      -0.336235
H        2.393947      -3.753005      -0.633611
H        3.882283      -1.751406       0.076977
C       -1.223440      -0.516019      -0.066303
C       -1.841679      -0.370449       1.186317
C       -2.027245      -0.334103      -1.204411
C       -3.190745      -0.036406       1.300576
C       -3.378761      -0.005428      -1.098799
C       -3.964211       0.151452       0.156022
H       -3.974203       0.124088      -1.996398
H       -3.640349       0.065392       2.282699
H       -5.014633       0.407131       0.241731
H       -1.264154      -0.539178       2.089815
H       -1.591652      -0.462102      -2.190360
C        1.337135       1.582433      -0.093125
C        0.745032       2.047982       1.248820
H        0.764639       3.140317       1.286147
H       -0.288199       1.723069       1.362519
H        1.323072       1.688604       2.104551
C        2.780972       2.110665      -0.228903
H        3.228216       1.779972      -1.168876
H        2.779574       3.203031      -0.210027
H        3.411332       1.759830       0.593725
C        0.504493       2.109797      -1.266569
H        0.890602       1.721371      -2.212117
H       -0.543888       1.830277      -1.178157
H        0.564341       3.200977      -1.294505
```

```
34
N-tBu-B-Ph-Dewar | SCF Done:  E(RB3LYP) =  -624.057588492     A.U. after    1
cycles
C        2.001028       1.126875      -0.718542
C        0.689653       1.984695      -0.823944
B       -0.012701       0.609812      -0.341270
N        1.230693      -0.054254      -0.248374
H        2.628268       0.957596      -1.596386
H        0.403344       2.437221      -1.774714
C        1.349879       2.866609       0.225232
C        2.460799       2.124364       0.340641
H        1.001805       3.760902       0.731256
H        3.324921       2.160905       0.992959
C       -1.509682       0.211947      -0.150553
C       -2.394404       1.145519       0.419449
C       -2.053886      -1.012073      -0.577609
C       -3.746570       0.858753       0.592130
C       -3.409672      -1.297736      -0.430305
C       -4.258781      -0.365829       0.165175
H       -4.402767       1.592307       1.048160
H       -3.804505      -2.246054      -0.778991
H       -5.313130      -0.588737       0.287195
```

```
H       -1.412832      -1.748026      -1.051400
H       -2.014715       2.112492       0.734703
C        1.819803      -1.334141       0.186279
C        2.019021      -2.243006      -1.043906
H        2.479686      -3.192801      -0.756421
H        1.061354      -2.457639      -1.524532
H        2.667258      -1.762868      -1.782156
C        3.181796      -1.068049       0.852620
H        3.069127      -0.428628       1.731295
H        3.629125      -2.012117       1.173216
H        3.880331      -0.586435       0.163215
C        0.893437      -2.014574       1.204891
H        0.743145      -1.373731       2.077137
H       -0.084747      -2.238836       0.779540
H        1.338469      -2.954958       1.541537
```

```
37
N-tBu-B-o-Tol-aza | SCF Done:  E(RB3LYP) =  -663.465328672     A.U. after    1
cycles
C       -2.738739       0.635636      -0.117395
C       -0.520301       2.294274      -0.425577
B       -0.307510       0.805476      -0.213666
N       -1.521668       0.001488      -0.044593
H       -3.618480       0.024668      -0.005764
H        0.327598       2.960909      -0.557913
C       -1.782176       2.825667      -0.474480
C       -2.903699       1.978403      -0.318205
H       -1.946952       3.888458      -0.635867
H       -3.912349       2.370900      -0.354645
C        1.194875       0.282686      -0.241472
C        2.065338       0.440260       0.863809
C        1.749482      -0.176684      -1.447328
C        3.413877       0.090204       0.738100
C        3.096175      -0.518517      -1.565168
C        3.932899      -0.393519      -0.461082
H        4.071353       0.208165       1.594205
H        3.486781      -0.870739      -2.513834
H        1.117414      -0.254518      -2.326753
H        4.982966      -0.655241      -0.533080
C        1.563521       0.982027       2.183969
H        1.023125       1.923047       2.049763
H        0.871508       0.286231       2.669274
H        2.390176       1.160990       2.874928
C       -1.539331      -1.514207       0.143484
C       -2.929292      -2.048029       0.543942
H       -2.826499      -3.114604       0.752656
H       -3.312025      -1.571094       1.449637
H       -3.672136      -1.950648      -0.250766
C       -1.139622      -2.182345      -1.184311
H       -1.801654      -1.857881      -1.991722
H       -0.113361      -1.945677      -1.458548
H       -1.221713      -3.269179      -1.092952
C       -0.570395      -1.911855       1.269531
H        0.459026      -1.649738       1.038953
H       -0.856790      -1.431818       2.208820
H       -0.614433      -2.994044       1.417188
```

```
37
N-tBu-B-o-Tol-TS | SCF Done:  E(RB3LYP) =  -663.336757439     A.U. after    1
cycles
C        0.637498      -2.330704      -0.758781
B        0.322533      -0.920098      -0.493013
N        1.559476       0.153986      -0.294250
C        2.126639      -0.724690       0.511014
C        2.843955      -1.872946      -0.127207
C        2.021765      -2.737170      -0.749744
H        2.223689      -0.530456       1.585855
```

```
H       -0.145149      -3.055819      -0.960605
H        2.386155      -3.602871      -1.296227
H        3.917151      -1.780042      -0.268181
C       -1.156513      -0.375433      -0.320609
C       -1.924978      -0.549322       0.854933
C       -1.794524       0.163743      -1.450330
C       -3.264266      -0.141814       0.865724
C       -3.132219       0.554113      -1.432406
C       -3.868908       0.411134      -0.260776
H       -3.592808       0.961149      -2.326044
H       -3.847872      -0.274831       1.771550
H       -4.910111       0.712908      -0.225085
H       -1.232203       0.266229      -2.373185
C        1.477022       1.607693       0.092326
C        0.753049       1.863254       1.425032
H        0.807031       2.929027       1.662322
H       -0.297884       1.582508       1.365018
H        1.217011       1.325762       2.256412
C        2.943062       2.076599       0.201532
H        3.480931       1.886993      -0.730178
H        2.974501       3.148989       0.407253
H        3.468517       1.563768       1.012541
C        0.799073       2.376722      -1.046995
H        1.269662       2.134806      -2.002970
H       -0.263082       2.149039      -1.112952
H        0.905129       3.451142      -0.874805
C       -1.346479      -1.195698       2.093340
H       -0.957115      -2.191998       1.869882
H       -0.513839      -0.618073       2.506982
H       -2.103759      -1.288536       2.874916

---------------------------------------
37
N-tBu-B-o-Tol-Dewar | SCF Done:  E(RB3LYP) =  -663.382733410     A.U. after    1
cycles
C       -2.145863       1.165857       0.530768
C       -0.850042       2.049593       0.558265
B       -0.117746       0.648592       0.197981
N       -1.343154      -0.046935       0.218061
H       -2.793103       1.086353       1.405715
H       -0.585524       2.610258       1.455850
C       -1.497517       2.797972      -0.597946
C       -2.595981       2.029799      -0.645960
H       -1.149442       3.632320      -1.197122
H       -3.449870       1.982214      -1.311114
C        1.364280       0.245507      -0.110095
C        1.783617       0.129746      -1.445073
C        2.323116       0.063437       0.911308
C        3.105220      -0.164525      -1.776591
C        3.643908      -0.239261       0.568726
C        4.039779      -0.353503      -0.762991
H        3.400181      -0.246577      -2.817093
H        4.375453      -0.385354       1.357679
H        5.071264      -0.586380      -1.004012
H        1.063167       0.276537      -2.244531
C       -1.891355      -1.387451      -0.044674
C       -0.740760      -2.357212      -0.342433
H       -1.139091      -3.362677      -0.502150
H       -0.190300      -2.061743      -1.237128
H       -0.033203      -2.398725       0.488703
C       -2.659857      -1.870490       1.201140
H       -3.491799      -1.203346       1.441375
H       -3.073370      -2.868222       1.030779
H       -1.996648      -1.916816       2.068764
C       -2.847694      -1.327063      -1.251340
H       -3.688924      -0.656131      -1.058871
H       -2.324733      -0.971570      -2.142494
H       -3.255224      -2.319461      -1.463014
C        1.931726       0.186520       2.367128
```

```
H        1.520255       1.176938       2.586508
H        1.165713      -0.546254       2.641101
H        2.790553       0.030668       3.023326
```

40

N-tBu-B-Xylyl-aza | SCF Done: E(RB3LYP) = -702.790557797   A.U. after   1 cycles

```
C        2.743931       0.666383      -0.000228
C        0.510649       2.322885      -0.000608
B        0.308680       0.811853      -0.000301
N        1.531167       0.011138      -0.000060
H        3.627427       0.045480      -0.000068
H       -0.346352       2.991350      -0.000740
C        1.763781       2.871277      -0.000755
C        2.898342       2.021760      -0.000572
H        1.915873       3.948084      -0.000993
H        3.902646       2.426891      -0.000682
C       -1.196363       0.299601      -0.000118
C       -1.900139       0.150473      -1.215543
C       -1.900143       0.151390       1.215423
C       -3.258168      -0.182738      -1.201509
C       -3.258161      -0.181849       1.201644
C       -3.937005      -0.356192       0.000131
H       -3.787966      -0.300180      -2.141804
H       -3.787953      -0.298613       2.142028
H       -4.990768      -0.613355       0.000229
C       -1.202124       0.345671      -2.544596
H       -0.704506       1.318504      -2.596566
H       -0.429459      -0.412474      -2.714521
H       -1.910182       0.283712      -3.373810
C       -1.202147       0.347782       2.544310
H       -0.428151      -0.409019       2.714101
H       -0.706149       1.321462       2.596095
H       -1.909915       0.284721       3.373688
C        1.672994      -1.507556       0.000427
C        2.435437      -1.940445       1.270478
H        2.511502      -3.030249       1.298546
H        3.448979      -1.539110       1.316965
H        1.901607      -1.613764       2.166174
C        0.324760      -2.237095       0.000759
H       -0.272061      -2.011474       0.882556
H       -0.272122      -2.012187      -0.881174
H        0.528632      -3.311099       0.001187
C        2.435311      -1.941285      -1.269409
H        1.901376      -1.615225      -2.165269
H        3.448834      -1.539943      -1.316274
H        2.511413      -3.031106      -1.296750
```

40

N-tBu-B-Xylyl-TS | SCF Done: E(RB3LYP) = -702.660995020   A.U. after   1 cycles

```
C        0.721484      -2.356378      -0.572884
B        0.378425      -0.947883      -0.330737
N        1.601151       0.171960      -0.236453
C        2.239886      -0.678929       0.545811
C        2.950293      -1.817932      -0.112219
C        2.113459      -2.725517      -0.648208
H        2.390967      -0.467366       1.610788
H       -0.051311      -3.108099      -0.704736
H        2.464003      -3.598392      -1.192362
H        4.006644      -1.695048      -0.333433
C       -1.100252      -0.426138      -0.089160
C       -1.741140      -0.536605       1.169923
C       -1.851642       0.030106      -1.195808
C       -3.069283      -0.124760       1.311917
C       -3.181094       0.433285      -1.021938
C       -3.785695       0.371817       0.226622
```

```
H       -3.744810       0.790418      -1.878516
H       -3.549986      -0.205401       2.281730
H       -4.814465       0.691898       0.352690
C        1.508689       1.628388       0.147738
C        0.908824       1.880484       1.541320
H        0.965021       2.949408       1.763278
H       -0.137875       1.581484       1.581793
H        1.458093       1.361646       2.331229
C        2.965359       2.137045       0.112941
H        3.417726       1.950827      -0.863746
H        2.987195       3.212162       0.305677
H        3.578503       1.646331       0.874693
C        0.702682       2.375471      -0.919339
H        1.097529       2.166730      -1.915860
H       -0.351001       2.103252      -0.893313
H        0.777996       3.451498      -0.741164
C       -1.038412      -1.137545       2.367632
H       -0.713004      -2.159429       2.156834
H       -0.143370      -0.576750       2.651501
H       -1.702072      -1.160365       3.234856
C       -1.279469       0.034948      -2.598098
H       -1.576910      -0.874623      -3.131157
H       -1.651378       0.886976      -3.174105
H       -0.189807       0.062101      -2.605325

----------------------------------------
40
N-tBu-B-Xylyl-Dewar | SCF Done:  E(RB3LYP) =  -702.708009816     A.U. after    1
cycles
C        2.225422       0.683982      -1.060843
C        0.954015       1.479306      -1.523807
B        0.181958       0.429765      -0.553171
N        1.392470      -0.204494      -0.208889
H        2.862655       0.173809      -1.785421
H        0.697821       1.541895      -2.582573
C        1.634025       2.675224      -0.874612
C        2.709164       1.996349      -0.448201
H        1.318185       3.707144      -0.764018
H        3.565091       2.253575       0.164104
C       -1.324531       0.184706      -0.186177
C       -1.929893       0.888028       0.878178
C       -2.108762      -0.700075      -0.958665
C       -3.282200       0.684054       1.166439
C       -3.459555      -0.883310      -0.649541
C       -4.046249      -0.198956       0.410076
H       -3.739572       1.225794       1.988431
H       -4.055589      -1.565900      -1.247148
H       -5.095344      -0.348420       0.641435
C        1.905137      -1.262010       0.677165
C        2.585089      -2.351458      -0.175783
H        2.975681      -3.148938       0.462329
H        1.873226      -2.791719      -0.878551
H        3.422500      -1.943987      -0.748282
C        2.930116      -0.660564       1.657542
H        2.474230       0.127733       2.261465
H        3.310276      -1.434224       2.330160
H        3.784493      -0.232488       1.126757
C        0.738303      -1.876384       1.461141
H        0.243094      -1.130687       2.086336
H       -0.010698      -2.304158       0.792015
H        1.109769      -2.673111       2.111005
C       -1.503806      -1.453967      -2.123595
H       -1.052689      -0.772738      -2.852208
H       -0.714806      -2.138679      -1.796556
H       -2.259693      -2.043966      -2.646072
C       -1.129465       1.864264       1.712512
H       -0.302721       1.370021       2.232932
H       -0.687546       2.651616       1.094447
H       -1.756312       2.344926       2.466513
```

```
----------------------------------------
43
N-tBu-B-Mes-aza | SCF Done:  E(RB3LYP) =  -742.117755415     A.U. after    1 cycles
C       -3.107539       0.541752       0.000212
C       -0.970857       2.320432       0.003460
B       -0.683278       0.822959       0.001354
N       -1.860095      -0.044710      -0.000852
H       -3.954896      -0.127605      -0.000830
H       -0.152521       3.035720       0.005028
C       -2.252580       2.798009       0.004061
C       -3.337821       1.886299       0.002514
H       -2.464741       3.864643       0.005835
H       -4.363305       2.234407       0.003170
C        0.846456       0.393477       0.002556
C        1.560114       0.279189       1.212662
C        1.564356       0.284713      -1.209555
C        2.935176       0.019639       1.196361
C        2.936094       0.025626      -1.192200
C        3.643291      -0.117927       0.003881
H        3.465722      -0.069563       2.140505
H        3.468527      -0.058559      -2.136233
C        0.857769       0.428500       2.545641
H        0.277294       1.354468       2.591417
H        0.155947      -0.392902       2.729963
H        1.574094       0.437498       3.370070
C        0.862462       0.444394      -2.541479
H        0.139132      -0.359350      -2.719434
H        0.305366       1.384606      -2.590023
H        1.576573       0.431553      -3.367744
C       -1.917814      -1.568776      -0.003726
C       -2.656757      -2.041235      -1.273718
H       -2.671887      -3.133542      -1.303754
H       -3.691180      -1.696993      -1.318434
H       -2.143018      -1.683517      -2.169300
C       -0.531717      -2.223713      -0.006969
H        0.050941      -1.963322      -0.888633
H        0.052897      -1.968193       0.874750
H       -0.676968      -3.307285      -0.009920
C       -2.653585      -2.045926       1.266273
H       -2.137227      -1.692066       2.161874
H       -3.687627      -1.701009       1.314970
H       -2.669433      -3.138341       1.292016
C        5.127643      -0.397594      -0.003302
H        5.523099      -0.469292       1.012223
H        5.353098      -1.337832      -0.516789
H        5.679024       0.393497      -0.521561

----------------------------------------
43
N-tBu-B-Mes-TS | SCF Done:  E(RB3LYP) =  -741.988528531     A.U. after    1 cycles
C       -1.231039      -2.337455       0.526519
B       -0.775790      -0.955291       0.319938
N       -1.913569       0.255036       0.210448
C       -2.588199      -0.534489      -0.604732
C       -3.399600      -1.627616       0.012522
C       -2.647880      -2.603240       0.555494
H       -2.689127      -0.297920      -1.670418
H       -0.520447      -3.146542       0.668705
H       -3.077938      -3.456266       1.073545
H       -4.449786      -1.429979       0.207387
C        0.740858      -0.536041       0.130368
C        1.416063      -0.677618      -1.107374
C        1.493932      -0.149414       1.259707
C        2.773088      -0.363459      -1.201182
C        2.854202       0.154947       1.128362
C        3.510397       0.071734      -0.096692
H        3.413197       0.455008       2.010960
H        3.273259      -0.474390      -2.159514
```

```
C        0.712972      -1.213736      -2.335400
H        0.309786      -2.212421      -2.149039
H       -0.131318      -0.587921      -2.638482
H        1.400654      -1.273025      -3.181949
C        0.888246      -0.119502       2.647776
H        1.120068      -1.047513       3.181682
H        1.293043       0.706614       3.239338
H       -0.197963      -0.030144       2.627558
C        4.967812       0.441349      -0.234018
H        5.480379       0.406611       0.730030
H        5.487676      -0.233746      -0.919078
H        5.080944       1.456549      -0.630617
C       -1.701023       1.705236      -0.148492
C       -1.040011       1.929942      -1.518976
H       -0.018226       1.552652      -1.531883
H       -1.601547       1.464641      -2.333278
H       -1.008498       3.002943      -1.725871
C       -3.116200       2.321032      -0.151028
H       -3.612253       2.156287       0.808157
H       -3.051896       3.397250      -0.327641
H       -3.739376       1.887580      -0.938914
C       -0.876482       2.376239       0.954447
H       -1.317082       2.183513       1.934908
H        0.154419       2.026691       0.956503
H       -0.866854       3.457275       0.791270

----------------------------------------
43
N-tBu-B-Mes-Dewar | SCF Done:  E(RB3LYP) =  -742.035393465     A.U. after    1
cycles
C       -2.629755       0.653063       0.971206
C       -1.406773       1.499005       1.472771
B       -0.557197       0.453515       0.563419
N       -1.731094      -0.226510       0.179382
H       -3.283756       0.139060       1.677984
H       -1.201930       1.593021       2.540405
C       -2.093083       2.657756       0.765535
C       -3.125530       1.935318       0.306346
H       -1.804941       3.696710       0.645169
H       -3.959298       2.151082      -0.350943
C        0.970470       0.247251       0.272154
C        1.609974       0.945652      -0.773777
C        1.748667      -0.593443       1.096395
C        2.979932       0.778313      -0.990029
C        3.116946      -0.737277       0.854060
C        3.753114      -0.064333      -0.190110
H        3.456137       1.324695      -1.799746
H        3.701428      -1.386637       1.500305
C       -2.169661      -1.319009      -0.703827
C       -2.847621      -2.414114       0.143514
H       -3.184593      -3.237103      -0.492837
H       -2.151184      -2.815607       0.883951
H       -3.721117      -2.024083       0.672612
C       -3.172376      -0.773881      -1.738690
H       -2.719007       0.018392      -2.339289
H       -3.496127      -1.573684      -2.410271
H       -4.063087      -0.366485      -1.253268
C       -0.950852      -1.908168      -1.425702
H       -0.455369      -1.158004      -2.045335
H       -0.216468      -2.296020      -0.717263
H       -1.267781      -2.729977      -2.073318
C        1.119088      -1.338830       2.253941
H        0.640746      -0.652106       2.959929
H        0.346052      -2.035204       1.914032
H        1.865341      -1.913998       2.805998
C        0.826437       1.881670      -1.668377
H        0.056709       1.348430      -2.235933
H        0.312422       2.654128      -1.088596
H        1.480129       2.382290      -2.385756
```

```
C        5.226282      -0.258666      -0.458926
H        5.773210      -0.484264       0.459894
H        5.393570      -1.091359      -1.151459
H        5.671009       0.632864      -0.907922

----------------------------------------
49
N-tBu-B-pentamethylphenyl-aza | SCF Done:  E(RB3LYP) =  -820.759261466     A.U.
after    1 cycles
C       -3.502874      -0.000080       0.501678
C       -1.400581      -0.000145       2.320948
B       -1.081966      -0.000033       0.828920
N       -2.243862      -0.000004      -0.060875
H       -4.337153      -0.000062      -0.183938
H       -0.595492      -0.000176       3.050870
C       -2.690607      -0.000214       2.773928
C       -3.758989      -0.000179       1.841272
H       -2.923439      -0.000299       3.836306
H       -4.790963      -0.000231       2.169718
C        0.457298       0.000041       0.424799
C        1.162268      -1.215714       0.323906
C        1.162244       1.215818       0.323973
C        2.546861      -1.218628       0.075766
C        2.546867       1.218853       0.075762
C        3.230889       0.000113      -0.082373
C        0.419486      -2.532124       0.467903
H        0.760596      -3.098650       1.341226
H       -0.652160      -2.375301       0.593400
H        0.558217      -3.178844      -0.405252
C        0.419283       2.532125       0.468161
H       -0.652272       2.375082       0.594148
H        0.760643       3.098807       1.341279
H        0.557457       3.178785      -0.405127
C        4.709781      -0.000317      -0.414996
H        5.331999      -0.005072       0.489416
H        4.991147      -0.875737      -1.001202
H        4.993654       0.879046      -0.993884
C        3.310971      -2.524511      -0.024240
H        2.757817      -3.355858       0.409424
H        3.527814      -2.790512      -1.066526
H        4.270139      -2.468909       0.495943
C        3.310874       2.524856      -0.024044
H        3.530168       2.789640      -1.066106
H        2.756481       3.356578       0.407261
H        4.268779       2.470163       0.498594
C       -2.272909       0.000056      -1.585703
C       -3.000067       1.270884      -2.073984
H       -2.998663       1.296687      -3.166565
H       -4.039411       1.321074      -1.745475
H       -2.487119       2.165345      -1.712296
C       -0.875236      -0.000150      -2.215802
H       -0.296464       0.881119      -1.945271
H       -0.296762      -0.881637      -1.945345
H       -1.001903      -0.000082      -3.301842
C       -3.000477      -1.270510      -2.074057
H       -2.487795      -2.165159      -1.712455
H       -4.039826      -1.320404      -1.745517
H       -2.999117      -1.296231      -3.166640

----------------------------------------
49
N-tBu-B-pentamethylphenyl-TS | SCF Done:  E(RB3LYP) =  -820.630145576     A.U.
after    1 cycles
C       -1.639199       0.525169      -2.331099
B       -1.159949       0.136716      -0.994504
N       -2.279182      -0.318833       0.153327
C       -3.003409       0.718316      -0.228884
C       -3.813877       0.572589      -1.473591
C       -3.056753       0.540785      -2.587286
```

```
H       -3.131163       1.591818       0.420969
H       -0.937908       0.769442      -3.123799
H       -3.477609       0.413967      -3.581242
H       -4.844370       0.243637      -1.376090
C        0.366424       0.131525      -0.555172
C        1.045922       1.295817      -0.135027
C        1.095525      -1.066186      -0.731649
C        2.416390       1.242038       0.181743
C        2.468917      -1.115492      -0.432213
C        3.114934       0.026447       0.072929
C       -2.042991      -0.560259       1.624720
C       -1.429795       0.637174       2.370831
H       -1.384423       0.405212       3.438270
H       -0.417895       0.840384       2.023528
H       -2.027930       1.545933       2.265490
C       -3.440134      -0.869168       2.202720
H       -3.902733      -1.704704       1.672420
H       -3.354410      -1.134080       3.259192
H       -4.104914      -0.003560       2.126027
C       -1.158463      -1.799649       1.792011
H       -1.566845      -2.641105       1.228326
H       -0.139165      -1.614198       1.457741
H       -1.124666      -2.079547       2.848468
C        0.313204       2.620216      -0.037509
H       -0.739039       2.509072      -0.291484
H        0.381223       3.059612       0.963107
H        0.727219       3.353562      -0.737787
C        0.403990      -2.306959      -1.266398
H        0.772706      -2.565892      -2.265022
H        0.572216      -3.177723      -0.625320
H       -0.671616      -2.160357      -1.356731
C        3.154878       2.486312       0.635845
H        2.617395       3.397684       0.381186
H        3.305933       2.494632       1.722699
H        4.143713       2.555885       0.176724
C        4.571978      -0.047157       0.483961
H        4.842361      -1.043967       0.833201
H        5.244069       0.196627      -0.348948
H        4.797954       0.649712       1.291655
C        3.258859      -2.391379      -0.650163
H        3.386675      -2.957027       0.281577
H        2.770475      -3.054557      -1.362224
H        4.258374      -2.183044      -1.037308

----------------------------------------
49
N-tBu-B-pentamethylphenyl-Dewar | SCF Done:  E(RB3LYP) =  -820.677613333     A.U.
after    1 cycles
C        3.046837       0.027739      -1.149566
C        1.840138       0.495762      -2.037665
B        0.965222       0.084143      -0.730455
N        2.128071      -0.302343      -0.029661
H        3.702388      -0.783103      -1.473206
H        1.644710       0.021013      -3.000574
C        2.533460       1.849235      -2.026817
C        3.552273       1.465942      -1.242855
H        2.255031       2.801036      -2.466575
H        4.381130       1.988438      -0.780245
C       -0.570315       0.070333      -0.398433
C       -1.191281       1.214388       0.139937
C       -1.345640      -1.067883      -0.696190
C       -2.570040       1.209685       0.414309
C       -2.725375      -1.075754      -0.426457
C       -3.328588       0.052774       0.158124
C        2.540482      -0.783338       1.298405
C        3.166658      -2.185316       1.157351
H        3.488505      -2.561558       2.132510
H        2.442937      -2.889522       0.739351
H        4.042376      -2.165765       0.503101
```

```
C        3.576102       0.186361       1.898677
H        3.158084       1.191563       1.993015
H        3.882540      -0.153934       2.891587
H        4.473003       0.246141       1.276381
C        1.312001      -0.858827       2.214509
H        0.854272       0.124035       2.344018
H        0.553740      -1.530945       1.808559
H        1.608873      -1.230865       3.198866
C       -0.683895      -2.288347      -1.309529
H        0.397503      -2.162968      -1.379716
H       -0.865132      -3.192718      -0.719869
H       -1.053575      -2.486237      -2.321616
C       -0.360174       2.450466       0.429576
H        0.698528       2.277653       0.231723
H       -0.667577       3.301309      -0.188129
H       -0.447025       2.766436       1.474359
C       -3.570568      -2.289762      -0.758461
H       -3.067975      -2.960190      -1.453502
H       -3.810919      -2.875197       0.137777
H       -4.520683      -2.003780      -1.216043
C       -4.803513       0.024099       0.505378
H       -5.425818       0.361282      -0.333717
H       -5.137005      -0.980550       0.767248
H       -5.031888       0.670493       1.353100
C       -3.248350       2.439999       0.984158
H       -3.468177       2.324473       2.052735
H       -2.633216       3.331819       0.878463
H       -4.198516       2.641986       0.483600

----------------------------------------
44
N-tBu-B-p-OMe-aza | SCF Done:  E(RB3LYP) =  -817.345166262     A.U. after    1
cycles
C        3.477339      -0.359450      -0.173593
C        1.457153      -2.271644      -0.322594
B        1.078625      -0.816027      -0.107925
N        2.200991       0.131429      -0.038115
H        4.285796       0.349678      -0.122134
H        0.687572      -3.036331      -0.387487
C        2.767141      -2.653283      -0.445238
C        3.787730      -1.677582      -0.367975
H        3.046552      -3.692630      -0.602308
H        4.831994      -1.949212      -0.460613
C       -0.470304      -0.471403       0.024553
C       -1.129965      -0.610798       1.275781
C       -1.264208      -0.207594      -1.105367
C       -2.497710      -0.388810       1.384047
C       -2.646922       0.011761      -0.984421
C       -3.261038      -0.059929       0.260105
H       -3.002341      -0.479908       2.339565
H       -3.221691       0.225785      -1.877162
C       -0.377633      -1.069646       2.507285
H       -0.174532      -2.144446       2.452049
H        0.588908      -0.574511       2.615967
H       -0.957259      -0.887462       3.415022
C       -0.687006      -0.195470      -2.507403
H        0.397468      -0.304410      -2.511096
H       -1.098472      -1.024033      -3.093608
H       -0.936232       0.729247      -3.038032
O       -4.594558       0.151995       0.485959
C       -5.425146       0.470233      -0.619822
H       -5.437082      -0.338308      -1.359426
H       -6.427221       0.599839      -0.213226
H       -5.108590       1.400230      -1.105476
C        2.051353       1.641492       0.159441
C        3.405405       2.374542       0.224657
H        3.197382       3.434312       0.383672
H        4.027957       2.038608       1.057377
H        3.976785       2.294203      -0.703143
```

```
C        1.264764       2.234332      -1.022078
H        1.752526       1.996168      -1.970705
H        0.242619       1.864585      -1.048868
H        1.225684       3.322808      -0.924649
C        1.320346       1.918719       1.483683
H        0.308496       1.519943       1.478891
H        1.868843       1.488657       2.325660
H        1.253442       2.998552       1.642131

----------------------------------------
44
N-tBu-B-p-OMe-TS | SCF Done:  E(RB3LYP) =  -817.217530145     A.U. after    1
cycles
C       -1.608127      -2.347070       0.397971
B       -1.109489      -0.969800       0.278548
N       -2.219554       0.272983       0.137203
C       -2.846418      -0.473691      -0.752571
C       -3.720758      -1.569636      -0.235491
C       -3.028264      -2.582279       0.318557
H       -2.862602      -0.200849      -1.814228
H       -0.925884      -3.176727       0.560033
H       -3.510101      -3.444621       0.771495
H       -4.777633      -1.355736      -0.104649
C        0.420640      -0.570401       0.218846
C        1.186385      -0.678389      -0.962029
C        1.093299      -0.231233       1.419140
C        2.555412      -0.379269      -0.956090
C        2.454869       0.061906       1.409655
C        3.188256       0.005726       0.225155
H        3.113042      -0.465711      -1.879658
H        2.972590       0.326875       2.325118
C        0.574328      -1.160012      -2.259979
H        1.323500      -1.204150      -3.053671
H        0.142156      -2.156436      -2.139005
H       -0.234015      -0.508809      -2.604231
C        0.379976      -0.250257       2.754982
H       -0.690350      -0.067704       2.658365
H        0.491670      -1.232291       3.226865
H        0.799434       0.492359       3.438828
O        4.514076       0.326772       0.325157
C        5.318279       0.267579      -0.843460
H        6.319079       0.562785      -0.531426
H        5.352995      -0.746723      -1.256240
H        4.960287       0.960476      -1.613113
C       -1.953138       1.728825      -0.158463
C       -1.193418       1.982491      -1.471560
H       -1.127533       3.060534      -1.640722
H       -0.180558       1.584532      -1.424116
H       -1.704299       1.554557      -2.338081
C       -3.352485       2.375094      -0.239117
H       -3.917741       2.190761       0.677256
H       -3.255005       3.454757      -0.374951
H       -3.927174       1.980387      -1.082304
C       -1.194956       2.348557       1.019454
H       -1.707517       2.137634       1.960381
H       -0.173756       1.976951       1.082974
H       -1.152148       3.433437       0.890664

----------------------------------------
44
N-tBu-B-p-OMe-Dewar | SCF Done:  E(RB3LYP) =  -817.264247224     A.U. after    1
cycles
C       -2.961003       0.523761       1.020657
C       -1.776612       1.419041       1.528720
B       -0.887042       0.447618       0.575645
N       -2.030273      -0.283056       0.189803
H       -3.575537      -0.044433       1.721434
H       -1.559559       1.493297       2.595596
C       -2.534952       2.559409       0.866611
```

```
C       -3.534794       1.796313       0.401176
H       -2.304306       3.615312       0.772763
H       -4.389138       1.985830      -0.237515
C        0.642878       0.332896       0.251193
C        1.227428       1.105889      -0.780506
C        1.479288      -0.497379       1.019384
C        2.591963       1.021565      -1.035967
C        2.853665      -0.571783       0.754749
C        3.410257       0.183590      -0.275269
H        3.047864       1.607868      -1.825970
H        3.470437      -1.219690       1.364245
C       -2.425951      -1.370638      -0.719345
C       -3.016457      -2.531303       0.106004
H       -3.322177      -3.351184      -0.549900
H       -2.278383      -2.915391       0.814678
H       -3.896464      -2.210920       0.669936
C       -3.485347      -0.853230      -1.711080
H       -3.095388      -0.015846      -2.294766
H       -3.777799      -1.648631      -2.401958
H       -4.386174      -0.516540      -1.191313
C       -1.193888      -1.862335      -1.490171
H       -0.759892      -1.064327      -2.095981
H       -0.419406      -2.227842      -0.813348
H       -1.478784      -2.680374      -2.157192
C        0.913421      -1.330921       2.150162
H        0.358157      -0.714018       2.863522
H        0.219955      -2.092865       1.780209
H        1.705067      -1.843067       2.701180
C        0.378308       2.036003      -1.619107
H       -0.400214       1.490893      -2.162244
H       -0.131173       2.778540      -0.997613
H        0.983958       2.572089      -2.352363
O        4.736523       0.178955      -0.612201
C        5.621798      -0.647939       0.127538
H        5.349321      -1.705867       0.042010
H        6.607797      -0.494436      -0.308819
H        5.646773      -0.364381       1.185682

----------------------------------------
41
N-tBu-B-p-CN-aza | SCF Done:  E(RB3LYP) =  -795.060254640     A.U. after    1
cycles
C        3.295440       0.502244       0.000048
C        1.179842       2.308771       0.000133
B        0.880556       0.816224       0.000030
N        2.040543      -0.067941      -0.000018
H        4.133517      -0.178434       0.000020
H        0.372015       3.035765       0.000174
C        2.468714       2.768944       0.000189
C        3.541748       1.844092       0.000146
H        2.693856       3.832611       0.000267
H        4.571162       2.179879       0.000191
C       -0.660082       0.414638       0.000024
C       -1.372349       0.322824      -1.217333
C       -1.372327       0.322623       1.217373
C       -2.748122       0.097968      -1.208914
C       -2.748108       0.097783       1.208938
C       -3.441496      -0.023522       0.000010
H       -3.292637       0.021265      -2.143046
H       -3.292609       0.020948       2.143067
C       -0.661435       0.464986      -2.545298
H       -0.091406       1.396949      -2.592730
H        0.050544      -0.350302      -2.711818
H       -1.370360       0.458160      -3.375364
C       -0.661382       0.464419       2.545361
H        0.049513      -0.351745       2.712273
H       -0.090165       1.395662       2.592517
H       -1.370409       0.458788       3.375350
C       -4.852771      -0.257778      -0.000001
```

```
N       -5.993067      -0.449611      -0.000012
C        2.075950      -1.593578      -0.000114
C        2.805653      -2.078872       1.269831
H        2.804354      -3.171166       1.297807
H        3.845133      -1.750692       1.315643
H        2.297341      -1.715499       2.166219
C        0.678735      -2.223399      -0.000308
H        0.100595      -1.957559       0.882987
H        0.100770      -1.957382      -0.883666
H        0.802461      -3.309201      -0.000403
C        2.805900      -2.078722      -1.269977
H        2.297789      -1.715207      -2.166422
H        3.845407      -1.750599      -1.315539
H        2.804552      -3.171012      -1.298102

----------------------------------------
41
N-tBu-B-p-CN-TS | SCF Done:  E(RB3LYP) =  -794.929929094     A.U. after    1 cycles
C       -1.448913      -2.323919       0.482832
B       -0.980728      -0.943763       0.299643
N       -2.082027       0.280110       0.198973
C       -2.762112      -0.484253      -0.637796
C       -3.598707      -1.574495      -0.047551
C       -2.871535      -2.569060       0.491207
H       -2.854277      -0.221930      -1.698104
H       -0.751521      -3.146182       0.612888
H       -3.320369      -3.422229       0.992033
H       -4.650644      -1.366458       0.125042
C        0.554123      -0.566678       0.130917
C        1.230685      -0.697335      -1.107701
C        1.301035      -0.232366       1.284337
C        2.594091      -0.417986      -1.189593
C        2.665911       0.042233       1.181562
C        3.314115      -0.032181      -0.053220
H        3.110316      -0.507569      -2.138290
H        3.233556       0.310277       2.065516
C        0.521492      -1.180441      -2.353064
H       -0.295871      -0.516870      -2.647244
H        1.214239      -1.245706      -3.194369
H        0.082322      -2.167496      -2.190112
C        0.675119      -0.224776       2.662644
H        1.138393       0.530103       3.303083
H       -0.398584      -0.041740       2.630958
H        0.816642      -1.197513       3.145131
N        5.837695       0.508484      -0.234713
C        4.710029       0.266879      -0.153640
C       -1.846233       1.735826      -0.124205
C       -3.252585       2.370900      -0.123434
H       -3.171870       3.449705      -0.274390
H       -3.874928       1.965099      -0.926499
H       -3.758882       2.191143       0.827546
C       -1.020901       2.367921       1.000714
H       -1.473945       2.162948       1.972888
H        0.004916       2.003080       1.005490
H       -0.990263       3.451562       0.861219
C       -1.171485       1.981907      -1.484170
H       -0.152819       1.596093      -1.499005
H       -1.731167       1.540946      -2.313043
H       -1.126068       3.058344      -1.667804

----------------------------------------
41
N-tBu-B-p-CN-Dewar | SCF Done:  E(RB3LYP) =  -794.977117273     A.U. after    1
cycles
C       -2.831255       0.645091       0.936425
C       -1.620934       1.502238       1.450985
B       -0.756413       0.454863       0.563469
N       -1.914319      -0.236486       0.164638
H       -3.492201       0.131261       1.636223
```

H	-1.434261	1.606204	2.520782
C	-2.302959	2.651305	0.722772
C	-3.323494	1.918513	0.254129
H	-2.020687	3.691575	0.601824
H	-4.151619	2.124425	-0.413001
C	0.780918	0.262401	0.298992
C	1.421914	0.957672	-0.750572
C	1.549516	-0.558327	1.154756
C	2.793695	0.810847	-0.947306
C	2.920621	-0.692786	0.944664
C	3.547832	-0.014725	-0.106251
H	3.287296	1.339838	-1.754333
H	3.512155	-1.323779	1.597868
C	0.902800	-1.294325	2.307439
H	1.641020	-1.857783	2.880869
H	0.407027	-0.600658	2.993600
H	0.141558	-1.998065	1.958059
C	0.635343	1.865227	-1.669746
H	-0.121999	1.309425	-2.231075
H	0.107714	2.640664	-1.107296
H	1.288351	2.360138	-2.390854
C	-2.331074	-1.339824	-0.717549
C	-3.308453	-0.805738	-1.781588
H	-2.841455	-0.020359	-2.380778
H	-3.616176	-1.613031	-2.451390
H	-4.210461	-0.393504	-1.322094
C	-1.094293	-1.935312	-1.402321
H	-0.584152	-1.192551	-2.019215
H	-0.378626	-2.318084	-0.671915
H	-1.393697	-2.763947	-2.049003
C	-3.028197	-2.425177	0.126073
H	-2.350598	-2.817845	0.888418
H	-3.915475	-2.030758	0.628004
H	-3.347807	-3.255667	-0.509058
C	4.955578	-0.160465	-0.316192
N	6.093007	-0.279445	-0.486245

34

N-TMS-B-Ph-aza | SCF Done: E(RB3LYP) = -875.610968090 A.U. after 1 cycles

C	-2.541687	0.939104	0.057417
C	-0.333516	2.672301	0.040436
B	-0.109387	1.171003	0.026179
N	-1.303385	0.330355	0.020668
H	-3.407037	0.289614	0.057556
H	0.496935	3.372700	0.034531
C	-1.610171	3.170724	0.073965
C	-2.724793	2.292307	0.087421
H	-1.797745	4.242094	0.091862
H	-3.736057	2.679072	0.116173
C	1.365673	0.589309	0.023005
C	2.002325	0.170295	1.203164
C	2.139347	0.598655	-1.151104
C	3.334686	-0.244226	1.209807
C	3.469418	0.181198	-1.155183
C	4.071870	-0.246336	0.027439
H	3.797873	-0.559314	2.138995
H	4.037323	0.194806	-2.079546
H	1.693918	0.938593	-2.081336
H	1.454560	0.181326	2.140469
H	5.107511	-0.568173	0.028807
C	-3.030540	-2.117005	-0.327795
H	-2.984603	-3.205367	-0.440796
H	-3.698915	-1.911167	0.512674
H	-3.487736	-1.716891	-1.237048
C	-0.648058	-2.195073	1.573874
H	0.416357	-2.008973	1.723055
H	-1.192892	-1.768302	2.421182
H	-0.804979	-3.278869	1.590130

C   -0.226156   -2.067065   -1.507750
H   -0.551648   -1.592638   -2.438185
H   0.834670   -1.855219   -1.372430
H   -0.344150   -3.149356   -1.628302
Si   -1.269509   -1.494040   -0.055824

34
N-TMS-B-Ph-TS | SCF Done: E(RB3LYP) = -875.478112046   A.U. after   1 cycles
C   -0.374253   2.766499   -0.167242
B   -0.096843   1.324695   -0.154654
N   -1.316470   0.256615   -0.142129
C   -1.935133   1.006332   0.755637
C   -2.617007   2.258310   0.301857
C   -1.756798   3.194115   -0.137255
H   -2.069816   0.653668   1.788845
H   0.424998   3.500451   -0.189520
H   -2.084625   4.150239   -0.536771
H   -3.690736   2.227119   0.141078
Si   -1.282191   -1.548356   -0.160578
C   -0.105432   -2.124587   -1.495480
H   0.938892   -1.948964   -1.234014
H   -0.241327   -3.198748   -1.659629
H   -0.309100   -1.614969   -2.441202
C   -0.867392   -2.275427   1.526934
H   -1.066495   -3.352301   1.516712
H   0.183310   -2.131546   1.784751
H   -1.478260   -1.844886   2.326502
C   -3.058165   -1.995439   -0.600935
C   1.337438   0.674551   -0.016626
C   1.831427   0.146105   1.187051
C   2.208016   0.690913   -1.119740
C   3.129635   -0.352861   1.285974
C   3.508279   0.194450   -1.028153
C   3.972362   -0.334242   0.175067
H   3.487385   -0.745825   2.231917
H   4.158891   0.222351   -1.895890
H   -3.351543   -1.558641   -1.559190
H   -3.763039   -1.641610   0.158144
H   -3.173695   -3.081437   -0.675876
H   1.863366   1.103616   -2.062452
H   4.983067   -0.720259   0.249498
H   1.201054   0.146619   2.070829

34
N-TMS-B-Ph-Dewar | SCF Done: E(RB3LYP) = -875.524347186   A.U. after   1
cycles
C   1.599527   1.647310   -0.671050
C   0.185645   2.333268   -0.676422
B   -0.303619   0.832385   -0.339782
N   1.019083   0.303985   -0.324433
H   2.232817   1.656532   -1.559606
H   -0.180074   2.853751   -1.563398
Si   1.932299   -1.160490   0.071190
C   1.797673   -2.409181   -1.336560
H   2.381825   -3.309054   -1.117057
H   0.764516   -2.721039   -1.513337
H   2.176774   -1.985526   -2.271442
C   1.289781   -1.908172   1.675617
C   3.735986   -0.654762   0.277905
H   4.349778   -1.532141   0.504920
H   4.140412   -0.199384   -0.630847
H   3.865200   0.057879   1.097272
C   0.744444   3.162925   0.469901
C   1.942572   2.560151   0.496391
H   0.291709   3.936988   1.080645
H   2.801944   2.622808   1.153438
C   -1.716695   0.203465   -0.161660

C	-1.976876	-1.177698	-0.222035
C	-2.813941	1.056316	0.057722
C	-3.263885	-1.684103	-0.062739
C	-4.103530	0.558417	0.230237
C	-4.330865	-0.815598	0.169587
H	-3.438136	-2.753203	-0.119980
H	-4.930380	1.238311	0.404932
H	0.228112	-2.160808	1.618740
H	1.418041	-1.208509	2.506585
H	1.837360	-2.824263	1.920418
H	-1.163762	-1.869624	-0.409962
H	-2.648902	2.128452	0.093986
H	-5.333928	-1.208158	0.296616

37

N-TMS-B-o-Tol-aza | SCF Done: E(RB3LYP) = -914.936694336 A.U. after 1 cycles

C	-2.591510	0.992758	-0.126388
C	-0.310607	2.597768	-0.437950
B	-0.151783	1.099118	-0.245501
N	-1.381661	0.328259	-0.080930
H	-3.484403	0.393717	-0.004706
H	0.548553	3.249967	-0.569706
C	-1.562521	3.154132	-0.468601
C	-2.714822	2.340150	-0.310134
H	-1.702365	4.222798	-0.616131
H	-3.707461	2.772908	-0.331473
C	1.290796	0.438084	-0.270077
C	2.172449	0.516144	0.833843
C	1.774212	-0.135196	-1.456668
C	3.465357	-0.004729	0.723521
C	3.067644	-0.647452	-1.560369
C	3.916088	-0.588142	-0.459804
H	4.133135	0.051938	1.577876
H	3.408301	-1.082604	-2.493755
H	1.129271	-0.169890	-2.329608
H	4.924485	-0.982878	-0.520851
C	1.737821	1.160090	2.131850
H	1.459580	2.207462	1.982045
H	0.863139	0.659899	2.558522
H	2.536846	1.126665	2.875685
C	-3.186826	-1.961590	0.683629
H	-3.183452	-3.032537	0.912618
H	-3.504118	-1.437782	1.589749
H	-3.944878	-1.802909	-0.087969
C	-1.085890	-2.331518	-1.502199
H	-1.709344	-1.922462	-2.302748
H	-0.040458	-2.228828	-1.797101
H	-1.308112	-3.400873	-1.421345
C	-0.256969	-2.046445	1.479022
H	0.788238	-1.884528	1.214953
H	-0.456581	-1.538720	2.426849
H	-0.401366	-3.119573	1.645312
Si	-1.443557	-1.484250	0.137697

37

N-TMS-B-o-Tol-TS | SCF Done: E(RB3LYP) = -914.803153464 A.U. after 1 cycles

C	-0.396766	2.655157	-0.661384
B	-0.157023	1.218908	-0.466607
N	-1.414051	0.202599	-0.276823
C	-2.001657	1.102168	0.497294
C	-2.654298	2.285483	-0.138867
C	-1.764438	3.125232	-0.698553
H	-2.120681	0.929233	1.576874
H	0.422895	3.351286	-0.810860
H	-2.061505	4.022239	-1.235577

H	-3.725815	2.253329	-0.312655
Si	-1.414330	-1.574249	0.062523
C	-0.734929	-2.468628	-1.435752
H	0.346721	-2.359606	-1.525438
H	-0.961637	-3.536926	-1.354372
H	-1.195838	-2.098675	-2.355617
C	-0.485244	-2.030973	1.633045
H	-0.619495	-3.099186	1.834828
H	0.585152	-1.837318	1.540576
H	-0.858697	-1.488387	2.506572
C	-3.244696	-1.965940	0.280263
C	1.261861	0.529426	-0.323935
C	2.038430	0.559449	0.858068
C	1.824707	-0.052601	-1.471506
C	3.318237	-0.006648	0.851585
C	3.104533	-0.605486	-1.469377
C	3.852392	-0.589327	-0.296160
H	3.911559	0.019837	1.760506
H	3.512837	-1.038726	-2.376067
H	-3.819951	-1.690718	-0.608100
H	-3.676476	-1.438502	1.136909
H	-3.387811	-3.037347	0.452109
H	1.252551	-0.056858	-2.394210
H	4.849285	-1.016168	-0.273927
C	1.526429	1.212310	2.121618
H	1.256664	2.256177	1.943414
H	0.627485	0.715314	2.500377
H	2.279489	1.180022	2.912092

37

N-TMS-B-o-Tol-Dewar | SCF Done: E(RB3LYP) = -914.848212245 A.U. after 1 cycles

C	-1.730393	1.749271	0.211016
C	-0.308405	2.400498	0.092755
B	0.169018	0.853399	0.072208
N	-1.158185	0.359462	0.197032
H	-2.388252	1.962713	1.054592
H	0.045271	3.104093	0.847278
Si	-2.057831	-1.163979	0.279329
C	-0.991883	-2.468145	1.114005
H	-1.530270	-3.419920	1.170173
H	-0.059400	-2.641731	0.571984
H	-0.732814	-2.169489	2.133778
C	-2.556152	-1.725343	-1.451762
C	-3.612675	-0.839730	1.294968
H	-4.204988	-1.755500	1.388428
H	-3.368054	-0.499426	2.305565
H	-4.253570	-0.083233	0.832105
C	-0.824871	2.954453	-1.228230
C	-2.032675	2.376519	-1.143789
H	-0.345123	3.561496	-1.988430
H	-2.879341	2.303380	-1.816447
C	1.528818	0.104219	-0.109160
C	1.632884	-0.875538	-1.111912
C	2.683445	0.403991	0.651910
C	2.827844	-1.543659	-1.371555
C	3.871791	-0.285483	0.396718
C	3.952642	-1.249216	-0.607533
H	2.877771	-2.287658	-2.159056
H	4.751379	-0.062438	0.992753
H	-1.681066	-1.942307	-2.071056
H	-3.141881	-0.956729	-1.964862
H	-3.165681	-2.634046	-1.408935
H	0.761303	-1.108963	-1.715241
H	4.889595	-1.764483	-0.789248
C	2.653271	1.451504	1.742828
H	2.499826	2.451806	1.327004
H	1.843554	1.271605	2.456139

```
H        3.591722       1.463272       2.301052
```

```
40
N-TMS-B-Xylyl-aza | SCF Done:  E(RB3LYP) =  -954.260633774     A.U. after    1
cycles
C       -2.573920       1.026132       0.000563
C       -0.266557       2.613904       0.001496
B       -0.129835       1.097022       0.000644
N       -1.374884       0.337681       0.000170
H       -3.480739       0.430174       0.000253
H        0.606067       3.261830       0.001907
C       -1.507220       3.193077       0.001776
C       -2.677100       2.386007       0.001315
H       -1.627037       4.274273       0.002350
H       -3.662603       2.835163       0.001586
C        1.311746       0.433207       0.000358
C        1.987004       0.186819       1.215975
C        1.987279       0.188994      -1.215590
C        3.290532      -0.319334       1.202037
C        3.290730      -0.317254      -1.202317
C        3.941169      -0.576703      -0.000289
H        3.800551      -0.506822       2.141922
H        3.800893      -0.503177      -2.142430
H        4.953157      -0.967212      -0.000540
C        1.314395       0.460287       2.544668
H        0.914303       1.477245       2.590298
H        0.471774      -0.217757       2.720628
H        2.014512       0.335102       3.373515
C        1.315156       0.465928      -2.543809
H        0.465596      -0.204046      -2.716939
H        0.924863       1.486677      -2.590845
H        2.012707       0.332421      -3.373512
C       -2.590667      -1.902548      -1.548062
H       -2.762102      -2.982873      -1.594423
H       -3.567721      -1.413643      -1.578712
H       -2.047587      -1.614913      -2.453037
C       -0.010550      -2.459225      -0.002780
H        0.606491      -2.277241      -0.883554
H        0.608000      -2.277849       0.877051
H       -0.293152      -3.518702      -0.002915
C       -2.588449      -1.904939       1.546880
H       -2.041884      -1.623033       2.451563
H       -3.563452      -1.412268       1.582023
H       -2.764005      -2.984762       1.589421
Si      -1.602612      -1.473114      -0.000953
```

```
40
N-TMS-B-Xylyl-TS | SCF Done:  E(RB3LYP) =  -954.127854686     A.U. after    1
cycles
C       -0.455110       2.688705      -0.339041
B       -0.199962       1.244511      -0.245501
N       -1.455174       0.196579      -0.232604
C       -2.116340       1.034405       0.552892
C       -2.740662       2.250375      -0.044384
C       -1.822289       3.145090      -0.453932
H       -2.307176       0.788654       1.607779
H        0.360977       3.404608      -0.367272
H       -2.089146       4.080776      -0.938304
H       -3.791886       2.216052      -0.314051
Si      -1.461902      -1.595425       0.032365
C       -0.526883      -2.448267      -1.346354
H        0.548419      -2.274333      -1.286855
H       -0.698433      -3.527559      -1.272801
H       -0.877111      -2.123163      -2.329077
C       -0.796259      -2.094907       1.720180
H       -0.961030      -3.168130       1.864804
H        0.275585      -1.905494       1.804478
```

```
H       -1.303202      -1.579098       2.540884
C       -3.294456      -2.023564      -0.065934
C        1.214507       0.554517      -0.058214
C        1.842732       0.447260       1.205241
C        1.910328       0.116184      -1.207812
C        3.110909      -0.133632       1.302612
C        3.179478      -0.459245      -1.078721
C        3.774750      -0.595267       0.169754
H        3.585323      -0.216574       2.275368
H        3.704371      -0.796536      -1.967277
H       -3.723165      -1.721699      -1.025547
H       -3.870215      -1.535110       0.726805
H       -3.441499      -3.102818       0.041769
H        4.756742      -1.047250       0.260068
C        1.188928       0.987631       2.457644
H        1.009638       2.062575       2.372082
H        0.217536       0.523318       2.650392
H        1.818929       0.812886       3.332673
C        1.338819       0.315537      -2.595599
H        1.592516       1.311656      -2.973419
H        1.744557      -0.416962      -3.298043
H        0.250223       0.242604      -2.611821

----------------------------------------
40
N-TMS-B-Xylyl-Dewar | SCF Done:  E(RB3LYP) =  -954.172838074     A.U. after    1
cycles
C       -1.913756       1.292832       1.045330
C       -0.557329       2.050088       1.278884
B        0.052331       0.748210       0.525770
N       -1.223684       0.156702       0.344757
H       -2.548603       0.996164       1.881997
H       -0.224482       2.318557       2.282910
Si      -1.978210      -1.258985      -0.401322
C       -0.646617      -2.286632      -1.237691
H       -1.091259      -3.181921      -1.684536
H       -0.145465      -1.730510      -2.034087
H        0.120960      -2.611186      -0.530737
C       -3.260130      -0.675367      -1.655219
C       -2.843352      -2.259951       0.943051
H       -3.352793      -3.129722       0.515725
H       -2.130882      -2.624592       1.688767
H       -3.597688      -1.664388       1.466470
C       -1.176787       3.132772       0.409118
C       -2.324805       2.476418       0.181819
H       -0.785872       4.083318       0.061765
H       -3.186324       2.664200      -0.447821
C        1.497220       0.249743       0.170168
C        2.106683       0.624204      -1.047074
C        2.220015      -0.546583       1.085376
C        3.403101       0.190120      -1.337282
C        3.515583      -0.965530       0.769880
C        4.106504      -0.603113      -0.436399
H        3.864606       0.479590      -2.276187
H        4.065246      -1.578325       1.477443
H       -2.800559      -0.078371      -2.448032
H       -4.035414      -0.063560      -1.183938
H       -3.758848      -1.529187      -2.125277
H        5.112524      -0.933584      -0.671262
C        1.609943      -0.945932       2.412118
H        1.348071      -0.068277       3.012437
H        0.691798      -1.526091       2.278090
H        2.303918      -1.551290       2.998865
C        1.369279       1.492109      -2.043764
H        0.462286       1.002881      -2.413270
H        1.057094       2.440564      -1.596094
H        1.997434       1.722483      -2.906758

----------------------------------------
```

```
43
N-TMS-B-Mes-aza | SCF Done:  E(RB3LYP) =  -993.587935835     A.U. after    1 cycles
C       -2.973793       0.809682      -0.000033
C       -0.832869       2.615260       0.000465
B       -0.547187       1.118900       0.000160
N       -1.713197       0.241841      -0.000086
H       -3.817961       0.127849      -0.000228
H       -0.027814       3.345490       0.000657
C       -2.124099       3.070492       0.000507
C       -3.209536       2.152927       0.000242
H       -2.349093       4.134824       0.000728
H       -4.234258       2.503553       0.000255
C        0.950248       0.596285       0.000112
C        1.651360       0.416693       1.211482
C        1.651456       0.417413      -1.211337
C        2.997022       0.038943       1.194944
C        2.997096       0.039638      -1.194918
C        3.688894      -0.163086      -0.000016
H        3.520098      -0.092077       2.138594
H        3.520250      -0.090848      -2.138600
C        0.962686       0.629810       2.543215
H        0.482557       1.611414       2.593537
H        0.176996      -0.113692       2.717852
H        1.672231       0.557956       3.370406
C        0.962864       0.631292      -2.542987
H        0.177031      -0.111971      -2.718005
H        0.482925       1.613013      -2.592854
H        1.672425       0.559698      -3.370185
C       -2.703703      -2.106624      -1.547655
H       -2.774003      -3.198339      -1.590835
H       -3.721794      -1.710454      -1.581987
H       -2.187493      -1.772552      -2.452459
C       -0.083467      -2.410778      -0.000771
H        0.513848      -2.169195      -0.880770
H        0.513613      -2.170394       0.879727
H       -0.263143      -3.492517      -0.001516
C       -2.702846      -2.106904       1.547561
H       -2.185796      -1.773508       2.452134
H       -3.720693      -1.710185       1.582811
H       -2.773696      -3.198601       1.590310
Si      -1.762779      -1.581973      -0.000258
C        5.134089      -0.601497      -0.000102
H        5.661510      -0.237234       0.885030
H        5.214932      -1.694430      -0.001568
H        5.662134      -0.234843      -0.883881

----------------------------------------
43
N-TMS-B-Mes-TS | SCF Done:  E(RB3LYP) =  -993.455378839     A.U. after    1 cycles
C       -1.093987       2.654845      -0.306240
B       -0.658003       1.253171      -0.237067
N       -1.777086       0.056431      -0.212531
C       -2.518515       0.797492       0.597523
C       -3.300983       1.933145       0.029424
C       -2.509166       2.939206      -0.386534
H       -2.651996       0.520641       1.653553
H       -0.373979       3.467011      -0.343666
H       -2.900490       3.839879      -0.852456
H       -4.345103       1.771661      -0.221037
Si      -1.553909      -1.723810       0.031821
C       -0.537822      -2.441639      -1.366390
H        0.508132      -2.135735      -1.317225
H       -0.573805      -3.534522      -1.302582
H       -0.938000      -2.153179      -2.341459
C       -0.809472      -2.150971       1.706801
H       -0.837919      -3.237386       1.843728
H        0.231473      -1.830149       1.779718
H       -1.365568      -1.708180       2.538287
C        0.831449       0.737306      -0.087920
```

```
C        1.501702       0.695540       1.157469
C        1.555011       0.397979      -1.251657
C        2.832400       0.275568       1.218580
C        2.887981      -0.016033      -1.153059
C        3.542377      -0.097711       0.074678
H        3.428159      -0.272487      -2.060654
H        3.332444       0.249444       2.183045
C       -3.319298      -2.378781      -0.049295
H       -3.941132      -1.974301       0.755834
H       -3.328361      -3.469003       0.047496
H       -3.795262      -2.123342      -1.000081
C        0.819775       1.142268       2.431783
H        0.508456       2.187854       2.362137
H       -0.082802       0.561365       2.642338
H        1.487991       1.038851       3.289649
C        0.940261       0.538693      -2.628317
H        1.083804       1.555879      -3.008124
H        1.405101      -0.147628      -3.340821
H       -0.135330       0.355048      -2.623647
C        4.968710      -0.583594       0.171080
H        5.497522      -0.458039      -0.776555
H        5.522776      -0.043202       0.943062
H        5.005697      -1.647892       0.429457

---------------------------------------
43
N-TMS-B-Mes-Dewar | SCF Done:  E(RB3LYP) =  -993.500248193     A.U. after    1
cycles
C       -2.377175       1.214080       0.936924
C       -1.096039       2.084506       1.198991
B       -0.351608       0.808233       0.526219
N       -1.568999       0.111524       0.313722
H       -3.025876       0.899041       1.756117
H       -0.832427       2.415305       2.205030
Si      -2.175412      -1.385068      -0.407903
C       -0.730918      -2.333867      -1.144257
H       -1.083678      -3.276939      -1.574539
H       -0.237763      -1.768169      -1.938888
H        0.025411      -2.570545      -0.391789
C       -3.439082      -0.950598      -1.738760
C       -3.022478      -2.403135       0.935400
H       -3.442498      -3.325299       0.520834
H       -2.319576      -2.683096       1.725478
H       -3.844291      -1.850645       1.401333
C       -1.756067       3.082058       0.260091
C       -2.837877       2.328938       0.009170
H       -1.424104       4.047117      -0.108074
H       -3.680557       2.424462      -0.665178
C        1.141411       0.412736       0.254498
C        1.782696       0.789814      -0.944258
C        1.885258      -0.288270       1.227326
C        3.121327       0.451444      -1.155619
C        3.222905      -0.609989       0.984892
C        3.860212      -0.254150      -0.204717
H        3.600346       0.750874      -2.083973
H        3.782317      -1.147417       1.745900
H       -2.990742      -0.346970      -2.532987
H       -4.280731      -0.386088      -1.325890
H       -3.847400      -1.857261      -2.196911
C        1.252641      -0.687607       2.543707
H        0.894376       0.186975       3.096918
H        0.391049      -1.345939       2.395554
H        1.966585      -1.211913       3.182275
C        1.034177       1.563079      -2.008581
H        0.189036       0.990681      -2.404492
H        0.625195       2.497531      -1.612342
H        1.686919       1.814418      -2.847163
C        5.296783      -0.640177      -0.464452
H        5.866349      -0.707667       0.465726
```

```
H        5.358779      -1.617057      -0.957231
H        5.793438       0.084545      -1.114340

----------------------------------------
49
N-TMS-B-pentamethylphenyl-aza | SCF Done:  E(RB3LYP) =  -1072.23032862     A.U.
after    1 cycles
C        3.424128       0.703553      -0.000715
C        1.354498       2.597540      -0.002564
B        1.009100       1.115876      -0.001341
N        2.142078       0.188194      -0.000367
H        4.238440      -0.008590       0.000005
H        0.579287       3.359173      -0.003220
C        2.663774       2.999234      -0.002818
C        3.710073       2.038465      -0.001904
H        2.932079       4.053604      -0.003689
H        4.748196       2.347399      -0.002109
C       -0.509063       0.639495      -0.000778
C       -1.208699       0.493370      -1.217110
C       -1.208549       0.496109       1.215962
C       -2.568299       0.131532      -1.219016
C       -2.568123       0.134164       1.218865
C       -3.232863      -0.092140       0.000220
C       -0.497894       0.754857      -2.533108
H       -0.902283       1.641603      -3.033963
H        0.567116       0.933626      -2.385085
H       -0.596830      -0.081070      -3.231819
C       -0.497573       0.760608       2.531271
H        0.567166       0.940395       2.382522
H       -0.902770       1.647717       3.030815
H       -0.595305      -0.074276       3.231408
C       -4.675516      -0.557264       0.000835
H       -5.377163       0.286947      -0.000055
H       -4.903647      -1.163925      -0.876086
H       -4.903519      -1.161967       0.879141
C       -3.327700      -0.014105      -2.523434
H       -2.825156       0.491013      -3.346509
H       -3.442983      -1.066255      -2.813032
H       -4.332562       0.408242      -2.451610
C       -3.327281      -0.008815       2.523717
H       -3.442672      -1.060382       2.815376
H       -2.824474       0.497802       3.345709
H       -4.332092       0.413550       2.451285
C        3.702182      -2.394308       0.001573
H        3.581600      -3.482864       0.002712
H        4.287634      -2.137137      -0.885502
H        4.288424      -2.135387       0.887615
C        1.087793      -2.219762       1.548701
H        1.553942      -1.826012       2.455920
H        0.034897      -1.935390       1.551027
H        1.143031      -3.312812       1.594577
C        1.086379      -2.222919      -1.543554
H        0.033529      -1.938386      -1.545467
H        1.551756      -1.831081      -2.451988
H        1.141457      -3.316067      -1.587214
Si       1.974947      -1.630278       0.001547

----------------------------------------
49
N-TMS-B-pentamethylphenyl-TS | SCF Done:  E(RB3LYP) =  -1072.09680841     A.U.
after    1 cycles
C        1.499714      -2.595723      -0.585854
B        1.034870      -1.217451      -0.359513
N        2.134687      -0.009460      -0.234811
C        2.922719      -0.822267       0.455213
C        3.705848      -1.862971      -0.267723
C        2.913656      -2.838862      -0.752800
H        3.081634      -0.674187       1.533423
H        0.792482      -3.413503      -0.688327
```

```
H        3.299517      -3.676840      -1.327753
H        4.730084      -1.643972      -0.553676
Si       1.879271       1.715135       0.253553
C        0.793379       2.587056      -0.996300
H       -0.241119       2.243703      -0.951281
H        0.804961       3.662102      -0.786981
H        1.162992       2.441989      -2.014341
C        1.189829       1.882840       1.996765
H        1.231634       2.933638       2.302865
H        0.149235       1.557055       2.046088
H        1.762919       1.308823       2.730694
C        3.623908       2.426911       0.202061
C       -0.466545      -0.743889      -0.160846
C       -1.140968      -0.832093       1.075986
C       -1.171493      -0.308226      -1.305029
C       -2.485912      -0.429879       1.179129
C       -2.521064       0.075008      -1.206131
C       -3.160512       0.060529       0.046329
H        4.070131       2.315393      -0.790065
H        4.285302       1.936594       0.923665
H        3.609776       3.494103       0.444908
C       -0.430519      -1.382045       2.297618
H        0.608824      -1.617903       2.076372
H       -0.453259      -0.684102       3.140426
H       -0.895284      -2.313000       2.640125
C       -0.480213      -0.276369      -2.655758
H       -0.881814      -1.045782      -3.324611
H       -0.609059       0.686353      -3.158645
H        0.588942      -0.466356      -2.564010
C       -3.222904      -0.527095       2.500976
H       -4.239052      -0.905024       2.366482
H       -2.721936      -1.193646       3.200371
H       -3.308189       0.450393       2.992232
C       -4.586517       0.558227       0.173670
H       -5.315285      -0.248390       0.021694
H       -4.778687       0.981027       1.160212
H       -4.809609       1.334381      -0.558851
C       -3.291598       0.500719      -2.440855
H       -4.321528       0.139323      -2.415299
H       -3.336555       1.592838      -2.539802
H       -2.838862       0.115349      -3.353121

----------------------------------------
49
N-TMS-B-pentamethylphenyl-Dewar | SCF Done:  E(RB3LYP) =  -1072.14246992     A.U.
after    1 cycles
C        2.805577       0.542106      -1.400320
C        1.547365       1.178988      -2.093053
B        0.764621       0.433299      -0.880894
N        1.965778      -0.077201      -0.319904
H        3.455516      -0.154775      -1.932810
H        1.298483       0.957847      -3.132444
Si       2.523729      -1.020322       1.067311
C        1.057836      -1.385410       2.183861
H        1.377452      -1.993532       3.036508
H        0.612113      -0.467239       2.574953
H        0.273288      -1.931968       1.654793
C        3.836005      -0.026951       1.988801
C        3.294465      -2.623905       0.439605
H        3.682489      -3.223743       1.269289
H        2.560543      -3.231926      -0.097365
H        4.128807      -2.429872      -0.241602
C        2.220773       2.504661      -1.776399
C        3.283129       1.966680      -1.157372
H        1.905557       3.527810      -1.951971
H        4.120773       2.378573      -0.606988
C       -0.742479       0.254653      -0.477633
C       -1.364859       1.189620       0.372134
C       -1.490650      -0.817219      -1.003026
```

```
C       -2.717740       1.037161       0.722194
C       -2.844880      -0.971298      -0.658706
C       -3.446780      -0.060364       0.228688
H        3.430669       0.916833       2.364580
H        4.688735       0.208854       1.344694
H        4.219780      -0.591132       2.845019
C       -0.826797      -1.805350      -1.944792
H        0.242223      -1.609821      -2.041186
H       -0.932929      -2.837785      -1.597110
H       -1.256556      -1.760094      -2.951485
C       -0.563319       2.359668       0.912044
H        0.484841       2.300421       0.615408
H       -0.946965       3.318521       0.546794
H       -0.587062       2.401853       2.005814
C       -3.663711      -2.110064      -1.234085
H       -3.184967      -2.560218      -2.102012
H       -3.819479      -2.910362      -0.500002
H       -4.653188      -1.771666      -1.550898
C       -4.889656      -0.257542       0.648196
H       -5.584144       0.253089      -0.031472
H       -5.167559      -1.311846       0.656775
H       -5.076365       0.133001       1.648903
C       -3.399611       2.044187       1.627215
H       -3.533659       1.651830       2.642886
H       -2.830114       2.967880       1.713220
H       -4.392039       2.310149       1.255045

----------------------------------------
44
N-TMS-B-p-OMe-aza | SCF Done:  E(RB3LYP) =  -1068.81724868     A.U. after    1
cycles
C        3.364748      -0.607487      -0.164516
C        1.367322      -2.578135      -0.166286
B        0.971271      -1.114640      -0.059553
N        2.067694      -0.143767      -0.063459
H        4.152063       0.134366      -0.165682
H        0.621995      -3.369146      -0.171316
C        2.688770      -2.927540      -0.262010
C        3.697458      -1.928460      -0.260794
H        2.994437      -3.968686      -0.340940
H        4.744342      -2.196001      -0.335739
C       -0.555235      -0.691154       0.053120
C       -1.202968      -0.656321       1.314486
C       -1.337643      -0.471204      -1.095011
C       -2.552969      -0.334894       1.408345
C       -2.699554      -0.147808      -0.989544
C       -3.304431      -0.066567       0.261284
H       -3.050529      -0.293128       2.371008
H       -3.267635       0.028503      -1.894371
C       -0.456824      -1.025145       2.579320
H       -0.339985      -2.111836       2.651716
H        0.547901      -0.599033       2.606853
H       -0.993769      -0.689314       3.469281
C       -0.760414      -0.641557      -2.485513
H        0.313324      -0.452945      -2.515735
H       -0.913670      -1.667884      -2.837769
H       -1.243338       0.025526      -3.204691
O       -4.618294       0.254205       0.470779
C       -5.439508       0.517971      -0.656534
H       -5.508529      -0.354283      -1.316135
H       -6.427285       0.746973      -0.258978
H       -5.072053       1.376979      -1.229170
C        3.534887       2.490115       0.049156
H        3.377382       3.570891       0.130298
H        4.156431       2.191684       0.897935
H        4.101481       2.316869      -0.869897
C        0.892043       2.311949      -1.445475
H        1.352358       1.993309      -2.384743
H       -0.150447       1.991498      -1.445935
```

```
H        0.907122       3.407088      -1.425124
C        0.969080       2.146172       1.641531
H       -0.064639       1.800185       1.676062
H        1.493734       1.759283       2.519413
H        0.960315       3.238895       1.719170
Si       1.835468       1.666181       0.045776
```

```
44
N-TMS-B-p-OMe-TS | SCF Done:  E(RB3LYP) =  -1068.68467654     a.u. after    1
cycles
C       -1.502059       2.607846      -0.433702
B       -0.994450       1.236819      -0.285615
N       -2.053808      -0.019803      -0.273739
C       -2.874742       0.710326       0.465935
C       -3.683210       1.778848      -0.188797
C       -2.923536       2.811248      -0.600481
H       -3.047399       0.470888       1.525397
H       -0.825168       3.456583      -0.466886
H       -3.335335       3.671647      -1.121620
H       -4.701234       1.551160      -0.490046
Si      -1.746733      -1.772011       0.061911
C       -0.631615      -2.493281      -1.257488
H        0.395903      -2.137602      -1.170394
H       -0.621843      -3.583524      -1.152503
H       -0.996017      -2.259837      -2.260902
C       -1.059275      -2.088167       1.784940
H       -1.038648      -3.167865       1.968545
H       -0.040507      -1.710180       1.889916
H       -1.675344      -1.641043       2.570574
C       -3.468726      -2.527715      -0.068149
C        0.506691       0.804778      -0.039460
C        1.116252       0.855168       1.241395
C        1.305974       0.451029      -1.143203
C        2.455208       0.512225       1.395156
C        2.656930       0.111574      -0.973684
C        3.228881       0.130931       0.294990
H        2.930002       0.545214       2.369366
H        3.242930      -0.154599      -1.844418
C        0.345567       1.319544       2.457021
H        0.968125       1.282285       3.353422
H       -0.007647       2.345445       2.324088
H       -0.540755       0.705625       2.640312
C        0.759949       0.490647      -2.555342
H        1.247123      -0.253784      -3.190853
H       -0.316879       0.320192      -2.587325
H        0.938015       1.473843      -3.004055
O        4.529461      -0.192133       0.566203
C        5.375238      -0.565613      -0.511546
H        6.348273      -0.772994      -0.068596
H        5.008210      -1.466111      -1.016512
H        5.475424       0.244621      -1.242146
H       -4.148490      -2.125150       0.689716
H       -3.912974      -2.339714      -1.049538
H       -3.423435      -3.611680       0.076817
```

```
44
N-TMS-B-p-OMe-Dewar | SCF Done:  E(RB3LYP) =  -1068.72911273     A.U. after    1
cycles
C       -2.732138       1.070335       1.017265
C       -1.504605       2.013906       1.281410
B       -0.693760       0.812010       0.549887
N       -1.868660       0.043556       0.341365
H       -3.340180       0.687449       1.838749
H       -1.240136       2.328945       2.292372
Si      -2.394250      -1.460326      -0.426580
C       -0.897948      -2.320133      -1.168733
H       -1.202246      -3.262425      -1.636170
```

```
H       -0.416273      -1.707280      -1.934840
H       -0.144516      -2.549690      -0.411121
C       -3.662599      -1.055471      -1.762486
C       -3.206500      -2.551009       0.881012
H       -3.573735      -3.483015       0.439244
H       -2.501194      -2.813889       1.674840
H       -4.061576      -2.051841       1.347274
C       -2.249441       2.996000       0.390798
C       -3.285655       2.182107       0.137348
H       -1.989308       3.992062       0.048332
H       -4.147942       2.243966      -0.515846
C        0.813199       0.524496       0.228080
C        1.399419       0.986624      -0.974298
C        1.625142      -0.164441       1.147182
C        2.742805       0.743799      -1.239975
C        2.978283      -0.400915       0.869761
C        3.537098       0.049485      -0.324717
H        3.200501       1.091396      -2.159420
H        3.577248      -0.933621       1.597154
C        1.055061      -0.661738       2.459401
H        1.823351      -1.138201       3.071916
H        0.626880       0.158279       3.044889
H        0.255403      -1.391699       2.300328
O        4.843657      -0.132793      -0.687353
C        5.705079      -0.825837       0.202954
H        5.361273      -1.851471       0.377850
H        6.678883      -0.851472      -0.284041
H        5.794443      -0.304596       1.162627
H       -3.235650      -0.410149      -2.535448
H       -4.019749      -1.969181      -2.248578
H       -4.535957      -0.543887      -1.346587
C        0.574378       1.752404      -1.985717
H       -0.268701       1.157529      -2.350940
H        0.153173       2.662633      -1.547883
H        1.176202       2.044458      -2.848456

----------------------------------------
41
N-TMS-B-p-CN-aza | SCF Done:  E(RB3LYP) =  -1046.53007052     A.U. after    1
cycles
C       -3.161453       0.751709      -0.000118
C       -1.057947       2.602109      -0.000177
B       -0.748211       1.113255      -0.000022
N       -1.889464       0.209662      -0.000015
H       -3.990863       0.052352      -0.000112
H       -0.269160       3.349680      -0.000216
C       -2.359388       3.029628      -0.000262
C       -3.424617       2.090029      -0.000232
H       -2.605930       4.088871      -0.000363
H       -4.456198       2.419430      -0.000311
C        0.766948       0.632660      -0.000015
C        1.467221       0.478198       1.217502
C        1.467107       0.477821      -1.217548
C        2.821178       0.145454       1.209493
C        2.821065       0.145080      -1.209562
C        3.502354      -0.028578      -0.000040
H        3.358009       0.023632       2.143283
H        3.357810       0.022973      -2.143365
C        0.765825       0.669646       2.544686
H        0.274045       1.644947       2.596838
H       -0.011652      -0.084961       2.702872
H        1.468870       0.599479       3.376832
C        0.765591       0.668860      -2.544728
H       -0.011845      -0.085845      -2.702649
H        0.273739       1.644113      -2.597106
H        1.468574       0.598515      -3.376911
C        4.891069      -0.373092      -0.000052
N        6.012775      -0.653649      -0.000052
C       -2.831016      -2.159216      -1.548705
```

```
H       -2.877210      -3.252090      -1.592946
H       -3.857693      -1.786121      -1.583515
H       -2.321943      -1.813593      -2.453150
C       -0.204158      -2.402259       0.000187
H        0.386796      -2.151932      -0.881729
H        0.387069      -2.151424       0.881769
H       -0.357228      -3.487823       0.000516
C       -2.830985      -2.158901       1.549158
H       -2.321805      -1.813252       2.453532
H       -3.857595      -1.785619       1.583977
H       -2.877365      -3.251760       1.593534
Si      -1.905237      -1.618059       0.000163

----------------------------------------
41
N-TMS-B-p-CN-TS | SCF Done:  E(RB3LYP) =  -1046.39668597     A.U. after    1 cycles
C       -1.319794       2.632886      -0.283014
B       -0.862463       1.238626      -0.223514
N       -1.939711       0.022060      -0.195056
C       -2.689787       0.741593       0.628927
C       -3.504743       1.863286       0.077174
C       -2.743210       2.887807      -0.345594
H       -2.813340       0.447483       1.681320
H       -0.617169       3.460113      -0.314989
H       -3.159121       3.781188      -0.803263
H       -4.551560       1.682551      -0.147090
Si      -1.692015      -1.762192       0.016194
C       -0.664057      -2.429312      -1.397848
H        0.376437      -2.105308      -1.347289
H       -0.675207      -3.523577      -1.354476
H       -1.073339      -2.132927      -2.366702
C       -0.933168      -2.200246       1.681361
H       -0.944030      -3.288771       1.802103
H        0.103995      -1.867937       1.757571
H       -1.492577      -1.779799       2.522137
C       -3.446220      -2.438769      -0.072391
C        0.651016       0.777631      -0.088196
C        1.322127       0.738978       1.157508
C        1.374816       0.488846      -1.268247
C        2.664984       0.366991       1.213255
C        2.718558       0.120030      -1.192158
C        3.364882       0.045608       0.044801
H        3.180132       0.331578       2.166146
H        3.272011      -0.107082      -2.096259
C        0.625994       1.133279       2.440423
H       -0.245338       0.504917       2.643341
H        1.301836       1.049563       3.293788
H        0.265388       2.163299       2.385280
C        0.740116       0.629611      -2.634803
H        1.246322       0.001647      -3.371581
H       -0.319118       0.369416      -2.627137
H        0.807372       1.666490      -2.979393
N        5.849905      -0.662767       0.174042
C        4.739414      -0.346213       0.116293
H       -3.928322      -2.173979      -1.017390
H       -4.071514      -2.057955       0.741497
H       -3.439262      -3.530424       0.005398

----------------------------------------
41
N-TMS-B-p-CN-Dewar | SCF Done:  E(RB3LYP) =  -1046.44180050     A.U. after    1
cycles
C       -2.593622       1.191948       0.880882
C       -1.337337       2.098560       1.142556
B       -0.558097       0.824459       0.513742
N       -1.748964       0.092260       0.297624
H       -3.247543       0.880246       1.696764
H       -1.099134       2.460197       2.144042
Si      -2.313847      -1.437871      -0.396489
```

```
C       -0.832278      -2.376303      -1.069683
H       -1.157413      -3.335140      -1.486306
H       -0.327083      -1.823978      -1.866236
H       -0.093550      -2.586100      -0.291984
C       -3.549924      -1.059123      -1.767589
C       -3.170906      -2.428524       0.958700
H       -3.564548      -3.370255       0.562947
H       -2.481678      -2.672373       1.772384
H       -4.013365      -1.878413       1.389012
C       -2.004649       3.056898       0.167687
C       -3.064054       2.272213      -0.081088
H       -1.690464       4.021701      -0.215827
H       -3.900611       2.333170      -0.766623
C        0.952815       0.464079       0.275192
C        1.592025       0.818716      -0.933411
C        1.697725      -0.176536       1.290403
C        2.940057       0.519440      -1.121222
C        3.045090      -0.468088       1.086206
C        3.670843      -0.125826      -0.117573
H        3.433034       0.787665      -2.048479
H        3.619183      -0.961421       1.861954
C        1.051178      -0.545675       2.607581
H        1.764041      -1.031890       3.275878
H        0.665183       0.340246       3.121793
H        0.207152      -1.226496       2.464196
N        6.171993      -0.676887      -0.482406
C        5.054290      -0.430106      -0.319349
H       -3.093390      -0.468861      -2.567109
H       -4.411447      -0.499875      -1.390324
H       -3.930891      -1.984672      -2.211067
C        0.827966       1.521348      -2.033632
H        0.018998       0.895338      -2.422534
H        0.368843       2.445862      -1.672119
H        1.482518       1.777212      -2.868730

---------------------------------------
43
N-TBS-B-Ph-aza | SCF Done:  E(RB3LYP) =  -993.570179788     A.U. after    1 cycles
C        1.431685       2.138827       0.201386
C       -1.192311       2.769144      -0.548579
B       -0.855099       1.347655      -0.126650
N        0.531937       1.089317       0.250296
H        2.449804       1.923281       0.500610
H       -2.200413       3.047976      -0.842331
C       -0.216503       3.730259      -0.567342
C        1.112426       3.408012      -0.185252
H       -0.434360       4.752818      -0.867596
H        1.890042       4.161958      -0.187629
C       -2.005672       0.258165      -0.099352
C       -2.267622      -0.587201      -1.189724
C       -2.895835       0.195020       0.986614
C       -3.345018      -1.473803      -1.186400
C       -3.972831      -0.690061       1.000465
C       -4.199022      -1.533100      -0.086694
H       -3.520458      -2.113748      -2.044929
H       -4.638211      -0.718566       1.857034
C        2.243260      -1.277997      -0.689741
C        2.441807      -0.100921       2.212544
H        2.760981      -1.033037       2.688453
H        3.344812       0.441912       1.925637
H        1.923518       0.492101       2.972118
C       -0.008691      -1.695285       1.452913
H       -0.612778      -1.228196       2.233718
H       -0.691268      -2.086906       0.699362
H        0.523545      -2.537793       1.906773
Si       1.271846      -0.498737       0.780645
C        2.844673      -2.620865      -0.213596
H        3.413422      -3.083556      -1.029470
H        3.533386      -2.490767       0.626994
```

```
H        2.073290      -3.334592       0.089011
C        3.394036      -0.366252      -1.165133
H        4.112742      -0.154717      -0.367532
H        3.946962      -0.859007      -1.974615
H        3.029075       0.586931      -1.556540
C        1.306298      -1.547872      -1.884321
H        1.864917      -2.028920      -2.697038
H        0.478396      -2.210937      -1.619283
H        0.882861      -0.621753      -2.282661
H       -1.624914      -0.551748      -2.063303
H       -5.037339      -2.221113      -0.080875
H       -2.746007       0.850798       1.839518
```

```
43
N-TBS-B-Ph-TS | SCF Done:  E(RB3LYP) =  -993.440273591     A.U. after    1 cycles
C        1.268313       2.831875      -0.317648
B        1.035798       1.388753      -0.178606
N       -0.463860       0.819676       0.053406
C       -0.672584       1.790173       0.929118
C       -0.931327       3.176892       0.424455
C        0.132560       3.720037      -0.193066
H       -0.792215       1.566553       1.998956
H        2.262201       3.235405      -0.483049
H        0.104570       4.706815      -0.647898
H       -1.957838       3.529314       0.389979
Si      -1.076355      -0.874189       0.155647
C       -0.150858      -1.934703      -1.081162
H        0.870497      -2.135694      -0.755640
H       -0.662148      -2.894356      -1.203694
H       -0.100572      -1.453646      -2.060914
C       -0.908290      -1.546382       1.910629
H       -1.423948      -2.507304       1.999212
H        0.141526      -1.712613       2.161163
H       -1.335280      -0.879192       2.664778
C       -2.936090      -0.723087      -0.324237
C       -3.075176      -0.138980      -1.746020
H       -4.135283      -0.057031      -2.016746
H       -2.632559       0.857404      -1.821492
H       -2.596737      -0.775090      -2.496483
C       -3.576628      -2.129741      -0.294194
H       -3.097451      -2.817147      -0.997174
H       -3.537930      -2.582892       0.700922
H       -4.633861      -2.062730      -0.578276
C       -3.692404       0.180744       0.669473
H       -4.753190       0.234682       0.395230
H       -3.642432      -0.196964       1.695308
H       -3.315197       1.207921       0.665104
C        2.176022       0.299159      -0.084101
C        2.529463      -0.344736       1.113044
C        2.940937       0.003790      -1.225259
C        3.588047      -1.250050       1.169642
C        4.001460      -0.900859      -1.176521
C        4.325916      -1.535657       0.021090
H        4.574742      -1.108940      -2.073820
H        5.150198      -2.239173       0.061960
H        3.844475      -1.724445       2.111165
H        2.703175       0.492275      -2.164726
H        1.986097      -0.116859       2.024817
```

```
43
N-TBS-B-Ph-Dewar | SCF Done:  E(RB3LYP) =  -993.484505071     A.U. after    1
cycles
C       -0.555368       2.327614       0.465855
C        0.968480       2.603065       0.227231
B        1.043775       0.991129       0.218021
N       -0.358006       0.832431       0.440768
H       -1.064936       2.701223       1.354461
```

```
H        1.542451       3.190895       0.946202
Si      -1.610858      -0.368873       0.813214
C       -0.852020      -1.774504       1.816105
H       -1.637640      -2.435075       2.195573
H       -0.148799      -2.387430       1.249778
H       -0.313955      -1.370538       2.678454
C       -2.470553      -1.042469      -0.774741
C       -2.879411       0.512251       1.901129
H       -3.673466      -0.176264       2.204044
H       -2.407449       0.887112       2.814457
H       -3.352440       1.360528       1.399336
C       -1.452577      -1.742628      -1.697161
H       -0.659536      -1.062359      -2.019759
H       -0.986647      -2.606600      -1.213088
H       -1.953996      -2.112341      -2.600700
C       -3.560804      -2.061119      -0.374291
H       -3.146353      -2.919787       0.162637
H       -4.334489      -1.612001       0.255688
H       -4.059220      -2.449419      -1.271488
C       -3.130054       0.111317      -1.558314
H       -3.637461      -0.279857      -2.449421
H       -3.882006       0.638118      -0.962967
H       -2.391855       0.842536      -1.897664
C        0.505285       3.279293      -1.053970
C       -0.797891       3.018601      -0.869240
H        1.059074       3.758012      -1.854513
H       -1.686240       3.167934      -1.470986
C        2.253822       0.021549       0.061933
C        2.152988      -1.332390      -0.305830
C        3.548078       0.533731       0.273872
C        3.281384      -2.135764      -0.447048
C        4.681798      -0.266025       0.149598
C        4.550130      -1.605532      -0.212683
H        3.174240      -3.174323      -0.741403
H        5.429840      -2.231106      -0.318238
H        5.665703       0.154535       0.327243
H        3.664784       1.578466       0.542694
H        1.179865      -1.762632      -0.505482

----------------------------------------
46
N-TBS-B-o-Tol-aza | SCF Done:  E(RB3LYP) =  -1032.89598119     A.U. after    1
cycles
C        1.431138       2.174698       0.069393
C       -1.151511       2.589760      -0.931269
B       -0.779686       1.228362      -0.362435
N        0.587145       1.080187       0.131490
H        2.432086       2.042099       0.460857
H       -2.143232       2.784485      -1.330593
C       -0.228439       3.601036      -0.955832
C        1.078140       3.389879      -0.440931
H       -0.471682       4.580382      -1.362048
H        1.812081       4.186400      -0.442888
C       -1.866103       0.072891      -0.340268
C       -1.975009      -0.814376      -1.421772
C       -2.829755      -0.018504       0.690545
C       -2.972131      -1.787805      -1.484047
C       -3.825093      -0.998081       0.621846
C       -3.899592      -1.884306      -0.451465
H       -3.025717      -2.459002      -2.334579
H       -4.557463      -1.065557       1.420782
C        2.512691      -1.211114      -0.548534
C        2.404179       0.114173       2.294678
H        2.747815      -0.770726       2.838838
H        3.287938       0.706791       2.049300
H        1.789798       0.702178       2.983170
C        0.130912      -1.680512       1.430080
H       -0.561248      -1.229046       2.143760
H       -0.462121      -2.150193       0.646053
```

H	0.681961	-2.463888	1.960989
Si	1.379812	-0.428214	0.799845
C	3.148254	-2.492312	0.040252
H	3.809903	-2.953986	-0.702886
H	3.754764	-2.283536	0.927107
H	2.396728	-3.238479	0.312799
C	3.645762	-0.254438	-0.975619
H	4.281475	0.038108	-0.134299
H	4.292034	-0.750443	-1.710540
H	3.261569	0.654944	-1.444840
C	1.694058	-1.590992	-1.798940
H	2.343974	-2.074606	-2.538980
H	0.885135	-2.289250	-1.567811
H	1.253896	-0.711138	-2.276113
H	-1.266742	-0.742728	-2.241108
H	-4.681747	-2.635102	-0.482814
C	-2.800903	0.932552	1.867054
H	-3.629811	0.739533	2.551515
H	-1.870680	0.845345	2.438514
H	-2.869250	1.973994	1.538704

46

N-TBS-B-o-Tol-TS | SCF Done: E(RB3LYP) = -1032.76433373   A.U. after   1 cycles

C	1.207681	2.743322	-0.684159
B	0.950931	1.316657	-0.457174
N	-0.573993	0.794984	-0.197753
C	-0.781611	1.841995	0.585393
C	-1.006379	3.183098	-0.037274
C	0.086508	3.657254	-0.661941
H	-0.906835	1.718187	1.670245
H	2.209071	3.113928	-0.879940
H	0.089998	4.603937	-1.195951
H	-2.025189	3.544895	-0.136451
Si	-1.203530	-0.872216	0.100375
C	-0.534790	-2.025653	-1.217085
H	0.513998	-2.268838	-1.044912
H	-1.102998	-2.960750	-1.211281
H	-0.619579	-1.588435	-2.214847
C	-0.747221	-1.484174	1.824226
H	-1.228323	-2.447012	2.021343
H	0.332131	-1.633765	1.901827
H	-1.053931	-0.798517	2.618947
C	-3.115786	-0.698435	-0.073115
C	-3.481920	-0.204784	-1.489152
H	-4.571044	-0.113560	-1.585852
H	-3.044421	0.772892	-1.706993
H	-3.144288	-0.900472	-2.262743
C	-3.761411	-2.084871	0.155845
H	-3.411462	-2.828999	-0.565150
H	-3.568503	-2.471573	1.160938
H	-4.849572	-2.011023	0.040572
C	-3.693186	0.281547	0.967255
H	-4.785262	0.326584	0.873453
H	-3.468929	-0.022044	1.994430
H	-3.327119	1.303241	0.826785
C	2.065044	0.198226	-0.326794
C	2.748668	-0.097767	0.875694
C	2.475684	-0.456355	-1.499495
C	3.774946	-1.049581	0.864566
C	3.508245	-1.392602	-1.501994
C	4.154157	-1.699499	-0.308222
C	2.432875	0.629350	2.163358
H	1.394142	0.486000	2.475159
H	3.072831	0.280562	2.976931
H	2.580650	1.706502	2.047762
H	3.804253	-1.874873	-2.427343
H	4.956934	-2.428669	-0.288964

```
H        4.297478      -1.272959       1.789775
H        1.979657      -0.218815      -2.435485

----------------------------------------
46
N-TBS-B-o-Tol-Dewar | SCF Done:  E(RB3LYP) =  -1032.80807984     A.U. after    1
cycles
C        0.812059       1.762694      -1.229364
C       -0.677081       2.262109      -1.186572
B       -0.926466       0.784158      -0.576432
N        0.451041       0.436517      -0.614428
H        1.354076       1.693758      -2.173953
H       -1.195015       2.562639      -2.099277
Si       1.489945      -0.964029      -0.267550
C        0.502148      -2.185943       0.768512
H        1.099011      -3.080488       0.969653
H        0.204279      -1.757659       1.729078
H       -0.408460      -2.501941       0.256407
C        3.081125      -0.434366       0.680337
C        1.960260      -1.752378      -1.919678
H        2.632539      -2.605208      -1.786460
H        1.064874      -2.115230      -2.433269
H        2.455567      -1.040806      -2.586553
C        2.709040       0.313069       1.978271
H        2.131357       1.219291       1.781660
H        2.119804      -0.315043       2.653228
H        3.617607       0.607463       2.519245
C        3.886276      -1.699625       1.056022
H        3.325220      -2.364732       1.718380
H        4.187279      -2.275990       0.175738
H        4.804348      -1.413808       1.584687
C        3.983014       0.465400      -0.191928
H        4.886189       0.745238       0.365396
H        4.310559      -0.046645      -1.101593
H        3.488132       1.392160      -0.491364
C       -0.140896       3.346122      -0.268721
C        1.126857       2.908358      -0.276228
H       -0.638149       4.162258       0.244535
H        2.024107       3.205110       0.252893
C       -2.211962      -0.043395      -0.243863
C       -3.133603       0.356704       0.750895
C       -2.513180      -1.182808      -1.008381
C       -4.292055      -0.397064       0.957144
C       -3.680858      -1.916798      -0.808143
C       -4.572083      -1.524025       0.185661
H       -4.989416      -0.094416       1.732296
H       -5.481557      -2.088256       0.361005
H       -3.889266      -2.788117      -1.419348
C       -2.878011       1.585709       1.593837
H       -1.903736       1.542073       2.089858
H       -2.884462       2.492130       0.980174
H       -3.641268       1.700740       2.366251
H       -1.824103      -1.497103      -1.786604

----------------------------------------
49
N-TBS-B-Xylyl-aza | SCF Done:  E(RB3LYP) =  -1072.21894633     A.U. after    1
cycles
C        1.477277       2.106236       0.179533
C       -1.107903       2.602318      -0.767277
B       -0.757317       1.213533      -0.248020
N        0.623279       1.017008       0.193650
H        2.478154       1.945400       0.560198
H       -2.104983       2.831672      -1.134145
C       -0.171617       3.600710      -0.759953
C        1.136273       3.349566      -0.265723
H       -0.406699       4.601319      -1.116113
H        1.878898       4.137206      -0.230531
C       -1.904195       0.118963      -0.148791
```

```
C       -2.321920      -0.621916      -1.275875
C       -2.628424      -0.017124       1.058698
C       -3.397182      -1.511329      -1.170030
C       -3.700915      -0.909863       1.136254
C       -4.080487      -1.664796       0.030839
H       -3.706621      -2.078791      -2.042370
H       -4.248395      -1.006462       2.068753
C        2.783768      -1.078312      -0.499849
C        2.226542      -0.110830       2.437133
H        2.627766      -1.020187       2.894405
H        3.042701       0.612569       2.376219
H        1.475775       0.295930       3.121096
C        0.221188      -1.924221       1.039969
H       -0.548130      -1.665046       1.768010
H       -0.287278      -2.248074       0.131393
H        0.780101      -2.776252       1.440291
Si       1.436261      -0.519446       0.769701
C        3.414010      -2.390105       0.027189
H        4.187825      -2.733274      -0.670215
H        3.894338      -2.256641       1.001477
H        2.681326      -3.195979       0.118926
C        3.917415      -0.047253      -0.684166
H        4.404191       0.210809       0.261587
H        4.691041      -0.465592      -1.340104
H        3.565105       0.875117      -1.151300
C        2.147042      -1.353728      -1.877059
H        2.910565      -1.709271      -2.580258
H        1.371770      -2.123483      -1.824950
H        1.701749      -0.451718      -2.305253
H       -4.914670      -2.354639       0.100772
C       -2.270006       0.807225       2.277680
H       -2.254931       1.876473       2.046376
H       -2.989208       0.647450       3.083894
H       -1.277452       0.556453       2.666671
C       -1.626308      -0.473878      -2.612299
H       -0.659357      -0.985928      -2.625085
H       -2.230861      -0.898728      -3.416838
H       -1.434078       0.575226      -2.851815

----------------------------------------
49
N-TBS-B-Xylyl-TS | SCF Done:  E(RB3LYP) =  -1072.08920127     A.U. after    1
cycles
C        1.145985       2.764148      -0.461631
B        0.886444       1.331472      -0.271011
N       -0.654397       0.797807      -0.108221
C       -0.914407       1.851998       0.652088
C       -1.112751       3.186412       0.012215
C        0.023324       3.672564      -0.520933
H       -1.092090       1.736620       1.730469
H        2.156698       3.140170      -0.589117
H        0.063648       4.622372      -1.047863
H       -2.124548       3.534638      -0.169769
Si      -1.299143      -0.858086       0.237954
C       -0.497286      -2.127799      -0.882278
H        0.553266      -2.279328      -0.632466
H       -1.012224      -3.085983      -0.762135
H       -0.558583      -1.846555      -1.935618
C       -1.034237      -1.324548       2.046398
H       -1.562214      -2.256061       2.272156
H        0.026452      -1.492560       2.246524
H       -1.397181      -0.568695       2.748139
C       -3.181970      -0.715854      -0.152252
C       -3.395825      -0.291183      -1.620765
H       -4.468737      -0.221542      -1.839626
H       -2.947565       0.682674      -1.833141
H       -2.968463      -1.015075      -2.320793
C       -3.841038      -2.096898       0.069717
H       -3.418489      -2.865901      -0.582879
```

H	-3.745804	-2.441555	1.103833
H	-4.913126	-2.037043	-0.153734
C	-3.872549	0.305668	0.773008
H	-4.947030	0.342306	0.554782
H	-3.765688	0.047093	1.830896
H	-3.491511	1.321649	0.630916
C	1.991352	0.208227	-0.100522
C	2.586894	-0.082329	1.149486
C	2.465014	-0.455948	-1.254657
C	3.590435	-1.053120	1.231577
C	3.475688	-1.416756	-1.142163
C	4.029388	-1.725310	0.094964
C	2.201687	0.674687	2.401557
H	1.134789	0.594148	2.626642
H	2.751898	0.300557	3.267862
H	2.417918	1.741000	2.292812
C	1.933668	-0.115630	-2.630719
H	2.065571	-0.952856	-3.320876
H	0.875941	0.153284	-2.611875
H	2.467355	0.744698	-3.048013
H	3.829266	-1.924970	-2.034040
H	4.807354	-2.477278	0.172907
H	4.039441	-1.274586	2.194694

49

N-TBS-B-Xylyl-Dewar | SCF Done: E(RB3LYP) = -1072.13323304 A.U. after 1 cycles

C	0.920591	1.956771	-1.037486
C	-0.548683	2.485883	-0.874183
B	-0.819158	0.958704	-0.401877
N	0.539985	0.579944	-0.563800
H	1.427242	1.983746	-2.003532
H	-1.094006	2.905447	-1.721300
Si	1.526443	-0.894954	-0.504691
C	0.422962	-2.298090	0.091477
H	0.960180	-3.249797	0.039544
H	0.089495	-2.157011	1.122433
H	-0.471943	-2.386918	-0.528885
C	3.043230	-0.677143	0.661918
C	2.117104	-1.253564	-2.263543
H	2.771583	-2.129798	-2.296810
H	1.263185	-1.455405	-2.917125
H	2.669784	-0.413328	-2.693028
C	2.586217	-0.169863	2.045251
H	2.090588	0.802053	1.981102
H	1.892798	-0.867039	2.526020
H	3.450215	-0.060444	2.713266
C	3.746919	-2.041283	0.840792
H	3.096089	-2.780955	1.315562
H	4.091183	-2.457932	-0.111137
H	4.630486	-1.926157	1.481263
C	4.060292	0.319232	0.065141
H	4.911245	0.442994	0.747134
H	4.460007	-0.029827	-0.891371
H	3.628110	1.310188	-0.095799
C	0.053905	3.440807	0.141834
C	1.306308	2.972794	0.030393
H	-0.398835	4.203133	0.766875
H	2.231448	3.178672	0.555081
C	-2.103283	0.152245	0.003432
C	-2.505447	0.070393	1.354319
C	-2.901372	-0.456690	-0.989980
C	-3.669660	-0.625769	1.691299
C	-4.060228	-1.147126	-0.624445
C	-4.444022	-1.236145	0.709879
C	-2.518796	-0.366162	-2.451944
H	-3.253593	-0.869538	-3.083579
H	-2.453926	0.674710	-2.785359

H	-1.544093	-0.824931	-2.646024
H	-3.972110	-0.685786	2.732092
H	-5.345805	-1.773425	0.982841
H	-4.667521	-1.614680	-1.393125
C	-1.689266	0.730447	2.444588
H	-0.694689	0.282470	2.532564
H	-1.540329	1.796092	2.245546
H	-2.181728	0.639264	3.414960

58

N-TBS-B-pentamethylphenyl-aza | SCF Done: E(RB3LYP) = -1190.18486724   A.U. after   1 cycles

C	-2.492542	1.818187	0.000157
C	0.016206	3.054855	-0.013961
B	-0.065164	1.534731	-0.006700
N	-1.414352	0.955388	-0.001055
H	-3.472952	1.370363	0.006058
H	0.975962	3.564778	-0.019404
C	-1.124677	3.811152	-0.013117
C	-2.396053	3.180021	-0.005416
H	-1.085525	4.898379	-0.017919
H	-3.308159	3.764286	-0.003915
C	1.282775	0.687535	-0.003819
C	1.929255	0.385873	1.213414
C	1.928778	0.376047	-1.218872
C	3.158771	-0.298364	1.218348
C	3.158339	-0.307877	-1.218834
C	3.746419	-0.687127	0.001199
C	-3.657404	-1.278474	-0.003207
C	-0.977907	-1.614873	-1.521484
H	-1.218234	-2.679909	-1.578704
H	-1.328421	-1.140731	-2.441719
H	0.107977	-1.523018	-1.484493
C	-1.003086	-1.588177	1.566217
H	0.084132	-1.507909	1.538814
H	-1.358255	-1.088772	2.471180
H	-1.254676	-2.649168	1.646281
Si	-1.760136	-0.850089	0.009433
C	-3.748833	-2.828622	-0.006675
H	-4.804449	-3.125196	-0.007046
H	-3.289762	-3.273376	-0.892792
H	-3.289222	-3.278228	0.876864
C	-4.387381	-0.790324	-1.275659
H	-3.910795	-1.171581	-2.183399
H	-5.420742	-1.158997	-1.273452
H	-4.434713	0.296738	-1.364863
C	-4.402082	-0.795098	1.262595
H	-5.436346	-1.160797	1.245390
H	-3.937874	-1.182575	2.174065
H	-4.447681	0.291621	1.357376
C	1.308916	0.806849	-2.536459
H	1.918408	1.572393	-3.029714
H	1.207306	-0.024655	-3.239957
H	0.317704	1.236571	-2.392050
C	1.311561	0.829802	2.527703
H	0.320102	1.258040	2.380781
H	1.211276	0.005769	3.240074
H	1.922007	1.600445	3.011792
C	3.860648	-0.615664	2.524506
H	4.940295	-0.470639	2.445410
H	3.510626	0.016348	3.339001
H	3.699085	-1.656665	2.831947
C	5.027466	-1.497590	0.003982
H	5.918242	-0.856132	0.000332
H	5.097087	-2.137247	0.884383
H	5.095733	-2.145228	-0.870699
C	3.859705	-0.635709	-2.522632
H	4.939284	-0.489092	-2.445368

```
H        3.698896      -1.679451      -2.821036
H        3.508633      -0.011017      -3.342303

----------------------------------------
58
N-TBS-B-pentamethylphenyl-TS | SCF Done:  E(RB3LYP) =  -1190.05816405     A.U.
after    1 cycles
C       -0.161429       3.076812      -0.650386
B       -0.075474       1.636125      -0.365976
N       -1.457663       0.771089      -0.166784
C       -1.978840       1.793336       0.498592
C       -2.471813       2.983639      -0.251336
C       -1.461217       3.687863      -0.796897
H       -2.136280       1.734273       1.584629
H        0.736739       3.668083      -0.802067
H       -1.625103       4.581232      -1.394172
H       -3.527871       3.057866      -0.490181
Si      -1.687220      -0.945124       0.360818
C       -0.567180      -2.099741      -0.599360
H        0.480227      -1.956717      -0.332064
H       -0.837795      -3.135004      -0.369631
H       -0.663692      -1.960404      -1.678037
C       -1.371978      -1.119432       2.212574
H       -1.640029      -2.126487       2.546215
H       -0.312386      -0.970364       2.431258
H       -1.944235      -0.412817       2.819534
C       -3.534848      -1.316556      -0.047696
C       -3.799349      -1.126208      -1.556474
H       -4.849553      -1.347106      -1.785211
H       -3.595039      -0.102400      -1.879753
H       -3.186630      -1.796985      -2.165865
C       -3.844696      -2.782636       0.333397
H       -3.227750      -3.493428      -0.223416
H       -3.700045      -2.973742       1.401070
H       -4.891783      -3.013487       0.102419
C       -4.482626      -0.397721       0.748580
H       -5.526244      -0.649614       0.522674
H       -4.351339      -0.502116       1.829903
H       -4.354177       0.657971       0.490561
C        1.258171       0.800006      -0.168042
C        1.935598       0.717941       1.067183
C        1.828490       0.198747      -1.311661
C        3.136870      -0.009078       1.169307
C        3.040927      -0.506606      -1.215404
C        3.666383      -0.652200       0.035846
C        1.393509       1.437184       2.287293
H        0.443343       1.921801       2.070760
H        1.250739       0.764752       3.139069
H        2.077533       2.227182       2.616276
C        1.136619       0.315504      -2.656918
H        1.025345      -0.658747      -3.142515
H        0.146065       0.759847      -2.561524
H        1.703791       0.953522      -3.343896
C        4.922152      -1.491823       0.161811
H        5.829916      -0.894706       0.006152
H        5.004384      -1.947323       1.149073
H        4.940968      -2.300971      -0.568746
C        3.876849      -0.097853       2.490156
H        3.564071       0.677339       3.187139
H        3.710698      -1.062781       2.985528
H        4.955470       0.007905       2.353298
C        3.673894      -1.114296      -2.451949
H        4.762837      -1.045217      -2.421559
H        3.421128      -2.176747      -2.561512
H        3.345893      -0.612414      -3.361197

----------------------------------------
58
```

```
N-TBS-B-pentamethylphenyl-Dewar | SCF Done:  E(RB3LYP) =  -1190.10292099     A.U.
after    1 cycles
C        1.945754       1.751899      -1.285216
C        0.565603       2.500996      -1.306880
B        0.035269       1.103544      -0.675766
N        1.332314       0.518449      -0.680966
H        2.512023       1.579642      -2.202171
H        0.141232       2.878164      -2.239064
Si       2.075472      -1.060026      -0.360413
C        0.728107      -2.211279       0.272984
H        1.115914      -3.229178       0.375859
H        0.338394      -1.898360       1.244587
H       -0.116468      -2.242520      -0.419268
C        3.504188      -0.916543       0.923609
C        2.749900      -1.713781      -2.000510
H        3.265643      -2.670147      -1.871006
H        1.930871      -1.873597      -2.708191
H        3.456150      -1.018965      -2.463660
C        3.015633      -0.175566       2.185339
H        2.696241       0.845146       1.960904
H        2.174748      -0.689640       2.661099
H        3.822771      -0.117025       2.926968
C        3.968855      -2.333631       1.328280
H        3.172810      -2.903893       1.815274
H        4.322186      -2.914708       0.470410
H        4.802830      -2.266431       2.038413
C        4.711297      -0.161840       0.326571
H        5.509988      -0.076171       1.074567
H        5.131677      -0.683858      -0.537944
H        4.453951       0.852527       0.010823
C        1.239858       3.485689      -0.367754
C        2.412290       2.834760      -0.319664
H        0.867574       4.377754       0.124594
H        3.322453       2.974240       0.251339
C       -1.378302       0.546352      -0.278590
C       -1.861488       0.705270       1.034699
C       -2.190886      -0.068812      -1.251286
C       -3.132523       0.217046       1.384820
C       -3.464250      -0.554276      -0.906791
C       -3.918580      -0.438993       0.419866
C       -1.682826      -0.206752      -2.675078
H       -1.709268      -1.244736      -3.021727
H       -2.280653       0.381642      -3.379663
H       -0.650364       0.134041      -2.766785
C       -5.265076      -1.014631       0.809926
H       -6.071134      -0.278654       0.693347
H       -5.528651      -1.877793       0.198293
H       -5.278164      -1.340694       1.850380
C       -4.352899      -1.201447      -1.950759
H       -4.021880      -0.979592      -2.963843
H       -4.371022      -2.293625      -1.848995
H       -5.386295      -0.856570      -1.866516
C       -3.665611       0.389712       2.793347
H       -4.709658       0.712624       2.790204
H       -3.623154      -0.548165       3.360822
H       -3.100916       1.130535       3.356472
C       -1.001186       1.406682       2.069317
H       -0.829068       0.781797       2.951509
H       -0.022138       1.669583       1.666842
H       -1.464921       2.335014       2.420117

----------------------------------------
53
N-TBS-B-p-OMe-aza | SCF Done:  E(RB3LYP) =  -1186.77530735     A.U. after    1
cycles
C        2.579461       1.738419       0.041181
C        0.218838       2.890442      -0.920883
B        0.169639       1.497157      -0.305729
N        1.449974       0.944888       0.141201
```

```
H        3.503671       1.321531       0.421865
H       -0.680531       3.369956      -1.298431
C        1.401039       3.576920      -0.991451
C        2.595765       2.994883      -0.489761
H        1.452042       4.576736      -1.417207
H        3.533436       3.536325      -0.517680
C       -1.231936       0.776746      -0.118187
C       -1.881158       0.108749      -1.169404
C       -1.933266       0.925806       1.107729
C       -3.159595      -0.446934      -0.991638
C       -3.198506       0.375911       1.271423
C       -3.813688      -0.323668       0.228815
C        2.825659      -1.741666      -0.483019
C        2.804710      -0.468781       2.388947
H        2.908731      -1.419936       2.919444
H        3.810291      -0.075252       2.224534
H        2.284177       0.223367       3.057510
C        0.254703      -1.677820       1.262337
H       -0.342836      -1.151181       2.007425
H       -0.396787      -1.892143       0.415131
H        0.567017      -2.629620       1.704543
Si       1.805193      -0.724317       0.804410
C        3.126777      -3.127954       0.135181
H        3.703963      -3.731822      -0.575693
H        3.720708      -3.052360       1.051231
H        2.216012      -3.686046       0.368036
C        4.170120      -1.079779      -0.850999
H        4.800556      -0.901871       0.025917
H        4.733300      -1.739554      -1.522890
H        4.033445      -0.129431      -1.372048
C        2.010388      -1.942010      -1.776600
H        2.579859      -2.551686      -2.489353
H        1.064628      -2.458675      -1.590520
H        1.784730      -0.990798      -2.265382
C       -1.328033       1.708562       2.254164
H       -2.017294       1.765986       3.099015
H       -0.397600       1.257187       2.613228
H       -1.081807       2.730007       1.948440
C       -1.237180      -0.049490      -2.531547
H       -0.444262       0.682334      -2.694839
H       -0.797073      -1.044970      -2.653215
H       -1.975740       0.074634      -3.328561
H       -3.739318       0.488121       2.204617
H       -3.627645      -0.958006      -1.823865
C       -5.739850      -1.534743      -0.521644
H       -5.180772      -2.421822      -0.840258
H       -6.689543      -1.844893      -0.087864
H       -5.931080      -0.895915      -1.391214
O       -5.056308      -0.828916       0.502338

----------------------------------------
53
N-TBS-B-p-OMe-TS | SCF Done:  E(RB3LYP) =  -1186.64593719     A.U. after    1
cycles
C        0.157619       3.098011       0.570791
B        0.025044       1.658021       0.318099
N        1.382906       0.736127       0.161689
C        1.933740       1.710633      -0.546160
C        2.460791       2.917276       0.157497
C        1.479900       3.668561       0.691532
H        2.099588       1.598012      -1.626771
H       -0.718288       3.726953       0.699968
H        1.679390       4.573431       1.259807
H        3.522526       2.974681       0.375667
Si       1.563068      -1.015604      -0.252959
C        0.447316      -2.066100       0.825928
H       -0.603221      -1.934564       0.564971
H        0.699773      -3.121326       0.682912
H        0.565836      -1.835559       1.886724
```

```
C        1.193722      -1.329466      -2.075664
H        1.447736      -2.361483      -2.336092
H        0.129104      -1.192402      -2.278402
H        1.755185      -0.677478      -2.750328
C        3.413048      -1.399154       0.135284
C        3.721699      -1.108282       1.619495
H        4.772640      -1.336847       1.836913
H        3.548073      -0.059805       1.874434
H        3.111311      -1.720060       2.290242
C        3.680681      -2.895887      -0.146185
H        3.061782      -3.550224       0.474008
H        3.506494      -3.159473      -1.193746
H        4.727590      -3.134425       0.077571
C        4.360086      -0.563542      -0.748718
H        5.403179      -0.825736      -0.532390
H        4.196443      -0.740589      -1.816107
H        4.264521       0.510814      -0.563771
C       -1.324146       0.871028       0.071394
C       -1.945840       0.805567      -1.202737
C       -1.994738       0.298104       1.168484
C       -3.159393       0.143741      -1.358647
C       -3.222633      -0.358835       0.997997
C       -3.799343      -0.447258      -0.265652
H       -3.641736       0.086534      -2.328023
H       -3.710359      -0.789335       1.863530
C       -1.340834       1.493347      -2.406674
H       -0.326688       1.143359      -2.617372
H       -1.944661       1.321530      -3.300230
H       -1.270499       2.571684      -2.240520
C       -1.446512       0.413915       2.575344
H       -0.359609       0.506413       2.588405
H       -1.844456       1.307066       3.068889
H       -1.728508      -0.450530       3.182626
O       -4.983296      -1.075307      -0.537676
C       -5.695195      -1.675359       0.534354
H       -6.593275      -2.104540       0.092413
H       -5.110191      -2.470737       1.009531
H       -5.981657      -0.935246       1.289673

----------------------------------------
53
N-TBS-B-p-OMe-Dewar | SCF Done:  E(RB3LYP) =  -1186.68951334     A.U. after    1
cycles
C       -1.867644       1.945214       0.888531
C       -0.507732       2.712883       0.728199
B        0.046448       1.228222       0.380578
N       -1.232990       0.628023       0.534004
H       -2.412671       1.950564       1.833925
H       -0.080614       3.281491       1.556199
Si      -1.944952      -0.997301       0.558323
C       -0.589562      -2.225037       0.113160
H       -0.954029      -3.250110       0.229448
H       -0.241544      -2.107063      -0.915855
H        0.279508      -2.103993       0.763970
C       -3.420912      -1.136781      -0.671114
C       -2.547015      -1.327522       2.319251
H       -3.044223      -2.299019       2.398733
H       -1.702301      -1.331744       3.014658
H       -3.253462      -0.566969       2.663390
C       -2.997266      -0.655431      -2.074148
H       -2.685963       0.392154      -2.070267
H       -2.169179      -1.248147      -2.475341
H       -3.834767      -0.752071      -2.777005
C       -3.865248      -2.613813      -0.766136
H       -3.073765      -3.256946      -1.161099
H       -4.173648      -3.017131       0.203703
H       -4.725425      -2.703795      -1.441566
C       -4.623757      -0.298380      -0.187620
H       -5.451035      -0.377848      -0.904508
```

```
H       -4.999359      -0.644115       0.779845
H       -4.380568       0.762870      -0.090972
C       -1.223691       3.472563      -0.375370
C       -2.378884       2.799204      -0.265522
H       -0.884534       4.256696      -1.043941
H       -3.302284       2.800196      -0.832092
C        1.463004       0.632807       0.068879
C        1.929781       0.523378      -1.262793
C        2.321274       0.242529       1.112846
C        3.199966       0.020231      -1.522941
C        3.599134      -0.263103       0.838401
C        4.037909      -0.378532      -0.479118
H        3.566549      -0.067496      -2.539606
H        4.235600      -0.555544       1.663650
C        1.881481       0.356532       2.557721
H        2.693434       0.101524       3.241954
H        1.553274       1.372661       2.797454
H        1.041752      -0.310799       2.777312
O        5.264179      -0.858956      -0.849339
C        6.165305      -1.274638       0.165696
H        5.752712      -2.100885       0.755433
H        7.060999      -1.614902      -0.352044
H        6.426781      -0.446857       0.834318
C        1.057151       0.954992      -2.421352
H        0.146871       0.351316      -2.485884
H        0.739634       1.996753      -2.314552
H        1.587086       0.863222      -3.371412

----------------------------------------
50
N-TBS-B-p-CN-aza | SCF Done:  E(RB3LYP) =  -1164.48851108     A.U. after    1
cycles
C        2.359268       1.802972       0.206926
C       -0.036612       2.905082      -0.728447
B       -0.015671       1.466449      -0.234529
N        1.274458       0.943421       0.205011
H        3.293662       1.406190       0.583312
H       -0.950885       3.368315      -1.089964
C        1.107992       3.656649      -0.705275
C        2.319067       3.098692      -0.218016
H        1.113622       4.689996      -1.044307
H        3.225068       3.690175      -0.171778
C       -1.392593       0.672555      -0.161323
C       -1.958446       0.071006      -1.307860
C       -2.144854       0.699202       1.036639
C       -3.212035      -0.536483      -1.234459
C       -3.397777       0.091262       1.092690
C       -3.932343      -0.539418      -0.035864
C        2.902212      -1.589010      -0.498482
C        2.541480      -0.550944       2.448164
H        2.719805      -1.533186       2.895608
H        3.500839      -0.030314       2.408364
H        1.892463       0.006150       3.130292
C        0.189980      -1.830714       0.998962
H       -0.509167      -1.408570       1.721533
H       -0.362874      -2.021973       0.078661
H        0.527067      -2.793548       1.395700
Si       1.706425      -0.748773       0.765883
C        3.186540      -3.024313       0.007394
H        3.870539      -3.529326      -0.685092
H        3.663408      -3.029906       0.992396
H        2.280248      -3.632290       0.066690
C        4.254891      -0.859291      -0.641738
H        4.769600      -0.742670       0.317032
H        4.918075      -1.441228      -1.293508
H        4.146294       0.128335      -1.095567
C        2.245375      -1.676446      -1.891099
H        2.912671      -2.196466      -2.589420
H        1.304658      -2.234150      -1.868464
```

```
H        2.044988      -0.685502      -2.307627
C       -1.616978       1.397106       2.271735
H       -2.358676       1.393847       3.072684
H       -0.711598       0.916825       2.655791
H       -1.354992       2.437020       2.056963
C       -1.229976       0.078540      -2.633773
H       -0.338904      -0.554368      -2.608440
H       -1.872299      -0.287197      -3.437224
H       -0.897214       1.085905      -2.897537
H       -3.971150       0.109026       2.012412
H       -3.641794      -1.004232      -2.112806
C       -5.215738      -1.168199       0.031289
N       -6.251853      -1.678531       0.086390

---------------------------------------
50
N-TBS-B-p-CN-TS | SCF Done:  E(RB3LYP) =  -1164.35818493     a.u. after    1 cycles
C       -0.051187       3.071630       0.418541
B       -0.119350       1.614892       0.252724
N        1.243186       0.742394       0.078587
C        1.719926       1.695426      -0.711992
C        2.231234       2.962077      -0.107161
C        1.250323       3.703585       0.436926
H        1.849987       1.518056      -1.788601
H       -0.948167       3.669772       0.547198
H        1.438310       4.646211       0.943703
H        3.300603       3.077567       0.037779
Si       1.501206      -1.026319      -0.223107
C        0.441452      -2.045772       0.938095
H       -0.619075      -1.963026       0.698297
H        0.721297      -3.099444       0.843531
H        0.578076      -1.758980       1.982830
C        1.114550      -1.464834      -2.016181
H        1.421149      -2.494873      -2.221776
H        0.040629      -1.401461      -2.206499
H        1.626391      -0.825351      -2.740428
C        3.370007      -1.296437       0.155643
C        3.687495      -0.891032       1.610906
H        4.750846      -1.057022       1.823302
H        3.470631       0.163768       1.797724
H        3.116958      -1.482518       2.332872
C        3.701367      -2.795506      -0.030319
H        3.124067      -3.433484       0.644573
H        3.522481      -3.137045      -1.054222
H        4.761556      -2.970664       0.188151
C        4.260241      -0.479125      -0.801503
H        5.317987      -0.676317      -0.588592
H        4.088294      -0.735952      -1.851147
H        4.116618       0.599777      -0.686588
C       -1.467329       0.785954       0.120583
C       -2.131258       0.615379      -1.117492
C       -2.072087       0.289670       1.298549
C       -3.337202      -0.083469      -1.170329
C       -3.281990      -0.401184       1.226431
C       -3.912016      -0.602598      -0.005060
H       -3.844759      -0.220756      -2.118086
H       -3.741856      -0.788019       2.128696
C       -1.590068       1.220185      -2.393544
H       -0.584286       0.861571      -2.626463
H       -2.232858       0.980808      -3.243014
H       -1.524309       2.307786      -2.307398
C       -1.457107       0.530293       2.659981
H       -1.792326      -0.218759       3.381098
H       -0.366533       0.516914       2.627355
H       -1.747089       1.514046       3.042387
N       -6.137436      -1.916294      -0.129766
C       -5.143853      -1.327790      -0.073502

---------------------------------------
```

```
50
N-TBS-B-p-CN-Dewar | SCF Done:  E(RB3LYP) =  -1164.40218970     A.U. after    1
cycles
C       -1.722272       2.029111       0.689935
C       -0.336062       2.745247       0.511055
B        0.180564       1.222987       0.319435
N       -1.113085       0.667696       0.477108
H       -2.296921       2.131570       1.611519
H        0.078717       3.374692       1.299993
Si      -1.875902      -0.933154       0.618226
C       -0.531821      -2.224566       0.356570
H       -0.931681      -3.226660       0.537303
H       -0.125283      -2.206091      -0.657555
H        0.302475      -2.073927       1.045875
C       -3.291927      -1.149538      -0.666460
C       -2.564910      -1.074090       2.370861
H       -3.090248      -2.022301       2.519088
H       -1.754685      -1.029058       3.104724
H       -3.267289      -0.269247       2.604689
C       -2.788533      -0.808269      -2.084257
H       -2.452891       0.229119      -2.160374
H       -1.957540      -1.452665      -2.388175
H       -3.593077      -0.952178      -2.816509
C       -3.769968      -2.619266      -0.649414
H       -2.978463      -3.313387      -0.945394
H       -4.134937      -2.925426       0.336105
H       -4.599141      -2.751046      -1.355651
C       -4.494189      -0.244112      -0.321836
H       -5.287514      -0.371763      -1.069111
H       -4.924756      -0.490997       0.652837
H       -4.228975       0.816250      -0.310428
C       -0.993604       3.419590      -0.681304
C       -2.170012       2.787994      -0.552297
H       -0.611272       4.134282      -1.402030
H       -3.075984       2.764777      -1.145446
C        1.597578       0.574187       0.115216
C        2.103846       0.349759      -1.184293
C        2.402904       0.265632       1.234363
C        3.376661      -0.192975      -1.350492
C        3.673280      -0.276632       1.050029
C        4.164177      -0.511957      -0.238879
H        3.766782      -0.369634      -2.346173
H        4.291929      -0.517977       1.906632
C        1.904958       0.522151       2.639877
H        2.651979       0.236752       3.382627
H        1.673335       1.581005       2.791786
H        0.990716      -0.040029       2.852114
N        6.522885      -1.523216      -0.564845
C        5.468879      -1.070824      -0.419350
C        1.280936       0.696857      -2.405078
H        0.352053       0.120689      -2.440477
H        1.000076       1.754206      -2.407178
H        1.834275       0.497098      -3.324442

----------------------------------------
49
N-Hex-B-Mes-aza | SCF Done:  E(RB3LYP) =  -820.780661678     A.U. after    2 cycles
C       -0.023444       3.245486      -0.351479
C       -2.440195       2.356666       0.771926
B       -1.487561       1.340226       0.168183
N       -0.264494       1.897747      -0.381757
H        0.908929       3.576588      -0.796495
H       -3.388909       2.060289       1.211220
C       -2.118313       3.691001       0.763054
C       -0.898058       4.142321       0.193947
H       -2.791302       4.433668       1.185653
H       -0.647593       5.195476       0.176832
C       -1.778781      -0.216452       0.106357
C       -2.302164      -0.806610      -1.065174
```

```
C       -1.575016      -1.038433       1.236165
C       -2.584989      -2.175187      -1.096393
C       -1.863411      -2.404672       1.170455
C       -2.369895      -2.994922       0.011946
H       -2.985774      -2.610515      -2.007972
H       -1.691486      -3.022564       2.047837
C        0.797867       1.066826      -0.985469
H        0.357053       0.107680      -1.254856
H        1.129944       1.549719      -1.910989
C        1.994968       0.839722      -0.056160
H        2.408459       1.807065       0.252569
H        1.645828       0.344991       0.856169
C        3.092003      -0.004184      -0.714692
H        3.432847       0.492361      -1.632633
H        2.669795      -0.967392      -1.027624
C        4.296409      -0.254434       0.199984
H        4.718451       0.708488       0.515973
H        3.956643      -0.752653       1.116989
C        5.397679      -1.097386      -0.453132
H        5.736859      -0.599954      -1.370028
H        4.975916      -2.059645      -0.767762
C        6.596989      -1.342767       0.467033
H        7.363209      -1.945981      -0.027718
H        7.062151      -0.399692       0.771143
H        6.295671      -1.870642       1.377342
C       -2.571921       0.029213      -2.298985
H       -3.207532       0.889053      -2.067405
H       -1.648015       0.426925      -2.732827
H       -3.070658      -0.559613      -3.071819
C       -1.062454      -0.458457       2.538006
H       -1.783308       0.242604       2.970192
H       -0.878736      -1.244494       3.273811
H       -0.130083       0.097152       2.401608
C       -2.709796      -4.465908      -0.028485
H       -3.742662      -4.640276       0.293538
H       -2.610815      -4.870984      -1.038639
H       -2.060478      -5.043951       0.633837

---------------------------------------
49
N-Hex-B-Mes-TS | SCF Done:  E(RB3LYP) =  -820.642542757     A.U. after    1 cycles
C        1.198622      -2.698107      -0.254899
C        3.313331      -1.396660      -0.649689
B        2.090108      -0.848143      -0.050199
N        1.054643      -1.874012       0.767096
H        0.402437      -2.846311      -0.990772
H        4.056545      -0.756296      -1.116048
C        3.538139      -2.818475      -0.561136
C        2.432159      -3.533817      -0.272170
H        4.530596      -3.259839      -0.596770
H        2.399061      -4.517612       0.185588
C        1.560038       0.638171      -0.093834
C        1.029547       1.220428      -1.268453
C        1.692696       1.448891       1.057461
C        0.616451       2.555945      -1.258121
C        1.270487       2.781016       1.029627
C        0.718546       3.352477      -0.116744
H        0.211320       2.987933      -2.169129
H        1.385107       3.388720       1.923186
C       -0.278335      -1.406953       1.202132
H       -0.152261      -0.402067       1.603206
H       -0.539289      -2.053729       2.048037
C       -1.422745      -1.416549       0.184284
H       -1.153560      -0.800813      -0.679468
H       -1.602269      -2.432451      -0.187030
C       -2.721080      -0.882204       0.803886
H       -2.978506      -1.483534       1.685365
H       -2.553519       0.138865       1.167247
C       -3.903387      -0.884618      -0.171530
```

```
H       -3.645278      -0.285694      -1.054209
H       -4.072480      -1.906516      -0.535506
C       -5.202114      -0.346870       0.440156
H       -5.033574       0.674474       0.802300
H       -5.459240      -0.944074       1.323519
C       -6.379171      -0.352878      -0.539463
H       -7.288493       0.036261      -0.073262
H       -6.166155       0.264710      -1.417696
H       -6.595054      -1.366134      -0.892931
C        0.923600       0.440747      -2.561229
H        0.305538      -0.456095      -2.454323
H        0.485221       1.054786      -3.351057
H        1.905422       0.098220      -2.897306
C        2.351694       0.919666       2.314091
H        2.020314      -0.089997       2.567277
H        3.437747       0.865778       2.185190
H        2.150137       1.570922       3.167720
C        0.231875       4.781765      -0.118978
H        0.789241       5.394072       0.594030
H        0.332399       5.235845      -1.107874
H       -0.826560       4.837520       0.159689

----------------------------------------
49
N-Hex-B-Mes-Dewar | SCF Done:  E(RB3LYP) =  -820.684056759     A.U. after    1
cycles
C       -0.406993       2.707905       0.565633
C        1.116735       2.703470       0.956249
B        1.062983       1.185828       0.373810
N       -0.305447       1.313440       0.079539
H       -1.184016       2.893964       1.309593
H        1.426664       2.914364       1.980418
C        1.236636       3.819516      -0.074616
C       -0.056074       3.809166      -0.432092
H        2.096014       4.383468      -0.421649
H       -0.630012       4.327449      -1.191108
C        2.061396      -0.003443       0.165039
C        2.451596      -0.807281       1.258088
C        2.601667      -0.285818      -1.108036
C        3.346806      -1.861556       1.065793
C        3.499297      -1.345336      -1.264664
C        3.886416      -2.146489      -0.189760
H        3.630210      -2.475189       1.916686
H        3.906585      -1.549483      -2.251211
C       -1.321812       0.576192      -0.643762
H       -0.864309      -0.352361      -0.999036
H       -1.621175       1.148474      -1.534250
C       -2.564440       0.252843       0.195325
H       -2.255097      -0.323155       1.074998
H       -3.001990       1.185345       0.571481
C       -3.624312      -0.525125      -0.592017
H       -3.918429       0.054098      -1.476979
H       -3.183598      -1.456258      -0.971055
C       -4.874386      -0.857603       0.230374
H       -5.317136       0.072890       0.609073
H       -4.581805      -1.436532       1.115928
C       -5.935951      -1.637873      -0.553346
H       -6.228288      -1.059569      -1.438360
H       -5.493912      -2.567939      -0.930938
C       -7.181320      -1.965317       0.275608
H       -7.666423      -1.053468       0.638219
H       -7.917464      -2.521393      -0.311509
H       -6.926319      -2.573119       1.149544
C        1.899357      -0.544096       2.642683
H        2.147696       0.465376       2.985507
H        0.808260      -0.630873       2.664123
H        2.303489      -1.250664       3.370633
C        2.226787       0.550031      -2.313848
H        1.161629       0.462839      -2.550727
```

H	2.428763	1.612071	-2.146420
H	2.788882	0.239470	-3.197138
C	4.882981	-3.266215	-0.372029
H	4.848038	-3.670271	-1.386711
H	5.905800	-2.914961	-0.194925
H	4.694860	-4.085626	0.326252

7.2 IRC Scans

For a verification of the transitions states intrinsic reaction coordinates were calculated by using the local quadratic approximation.[33, 34]

IRC calculations confirm that the computed transition states connect either the dihydroazaborinine to the corresponding Dewar isomer or to a distinct intermediate. The latter has already been reported in several studies, where thermal ring opening likewise proceeds via a stepwise mechanism.[16, 35] In all cases, the transition state between the intermediate and the dihydroazaborinine constitutes the global maximum along the reaction coordinate and thus represents the rate-determining step.[15, 16, 35]

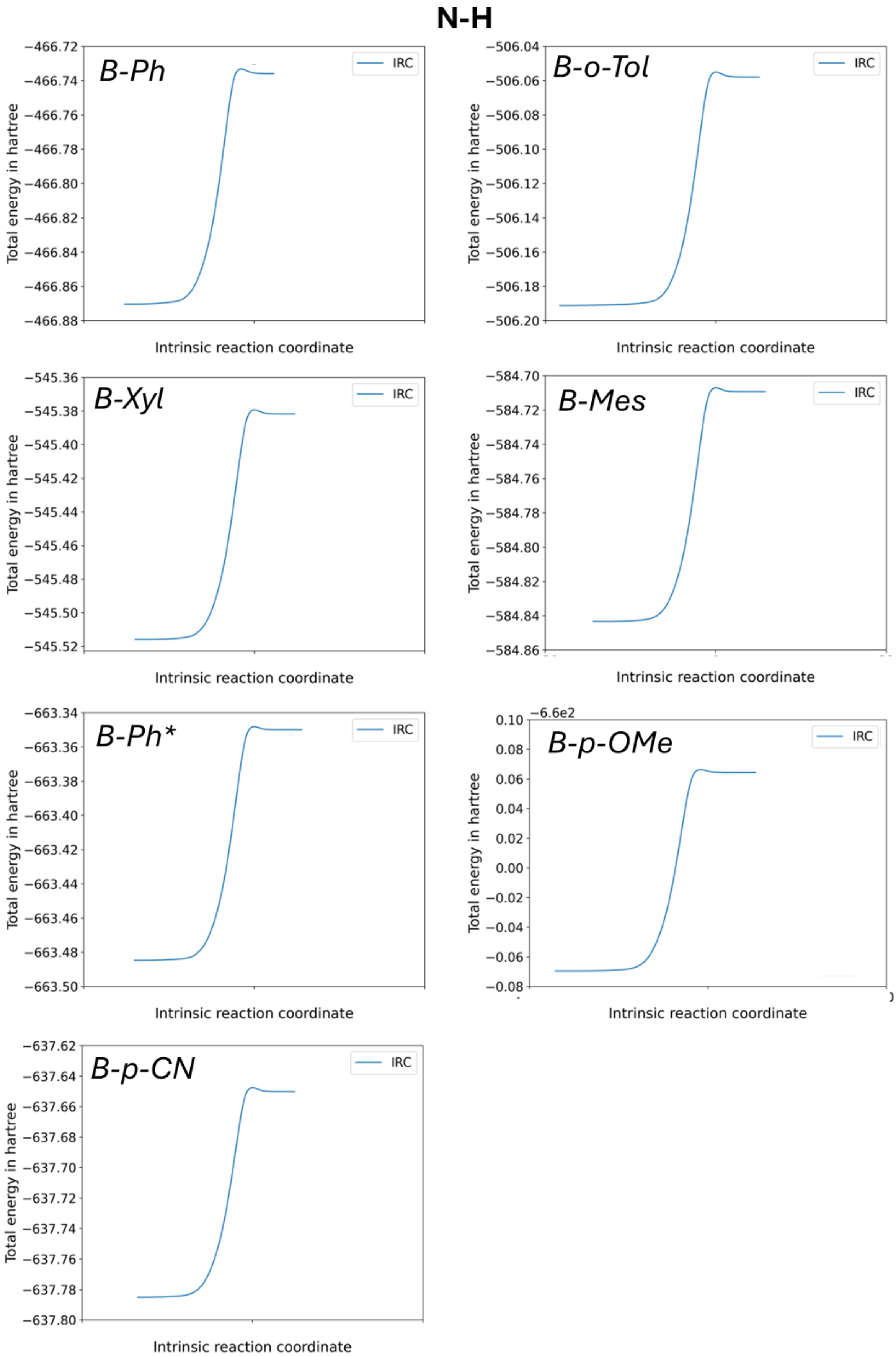

Figure S121. Intrinsic reaction coordinate of the thermal ring opening of the N-H substituted Dihydroazaborinines. All energies are given in Hartree.

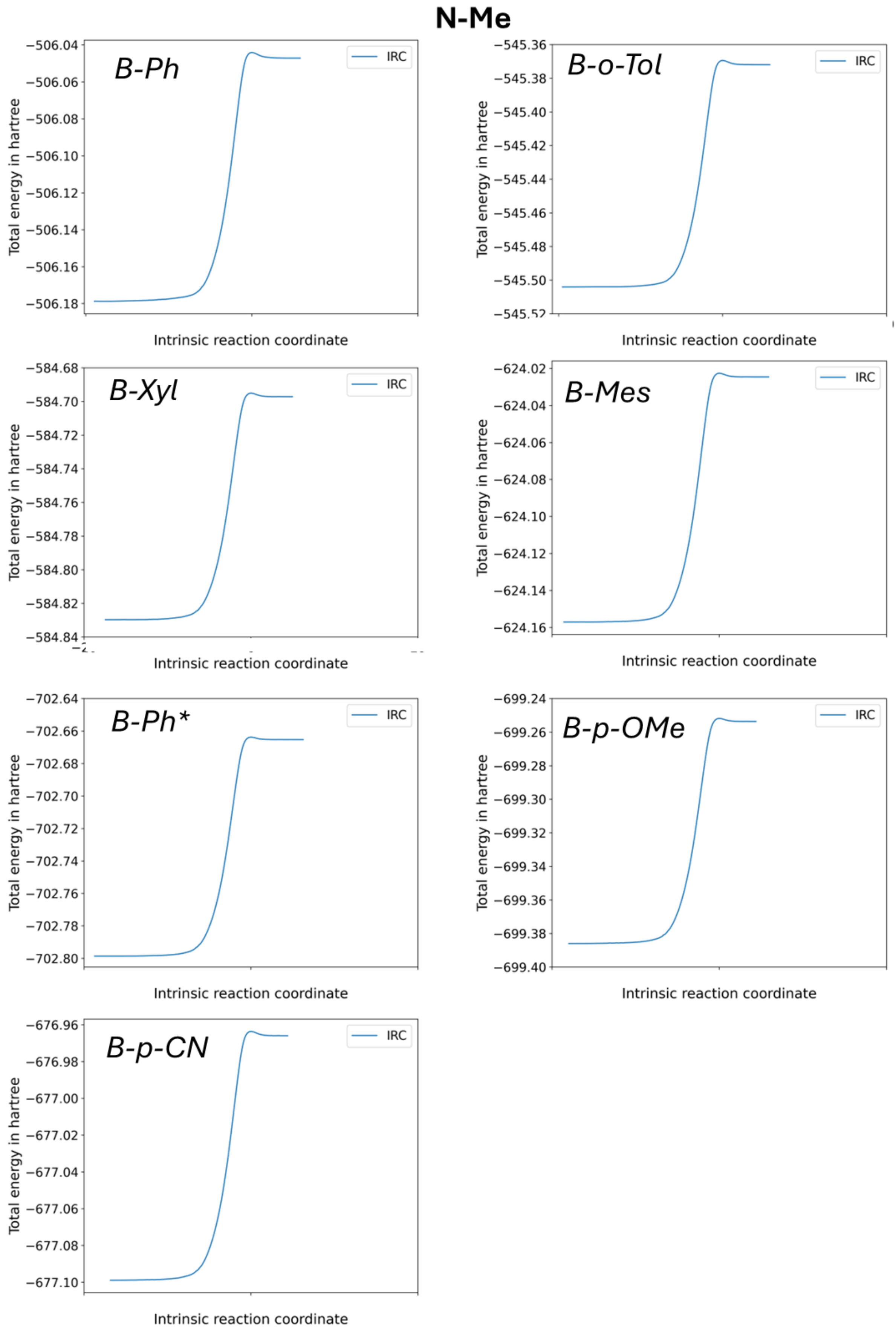

Figure S122. Intrinsic reaction coordinate of the thermal ring opening of the N-Me substituted Dihydroazaborinines. All energies are given in Hartree.

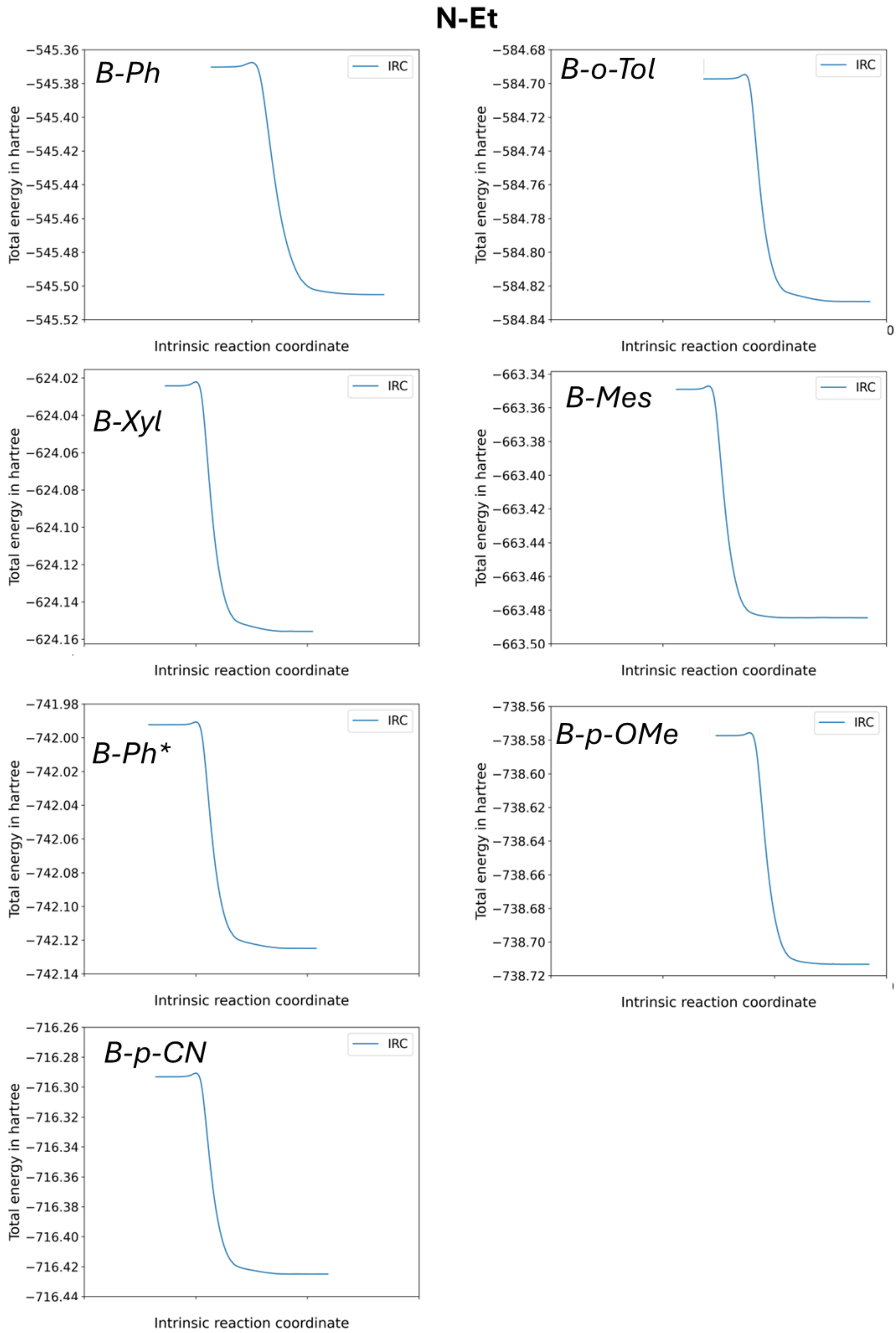

Figure S123. Intrinsic reaction coordinate of the thermal ring opening of the N-Et substituted Dihydroazaborinines. All energies are given in Hartree.

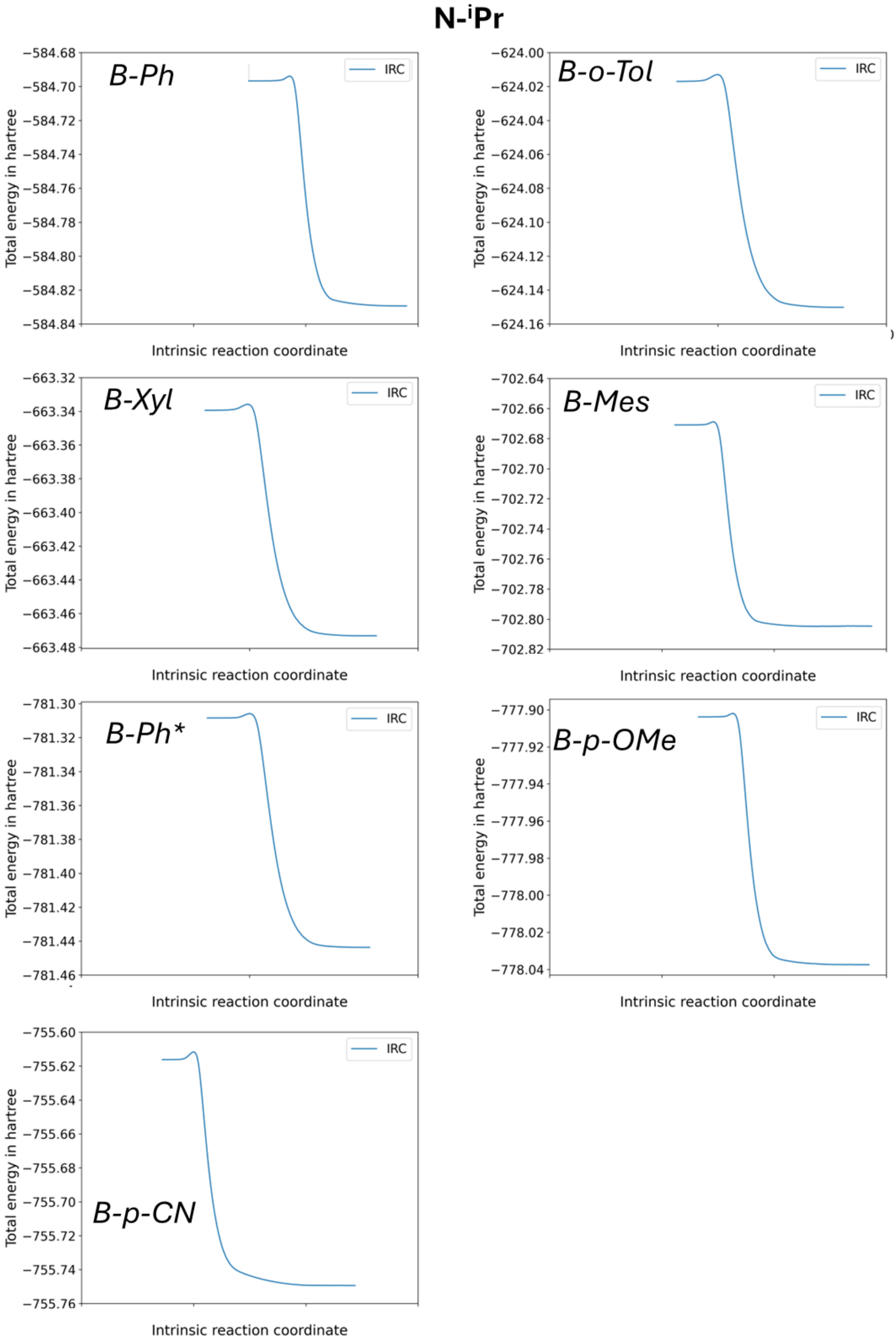

Figure S124. Intrinsic reaction coordinate of the thermal ring opening of the N-iPr substituted Dihydroazaborinines. All energies are given in Hartree.

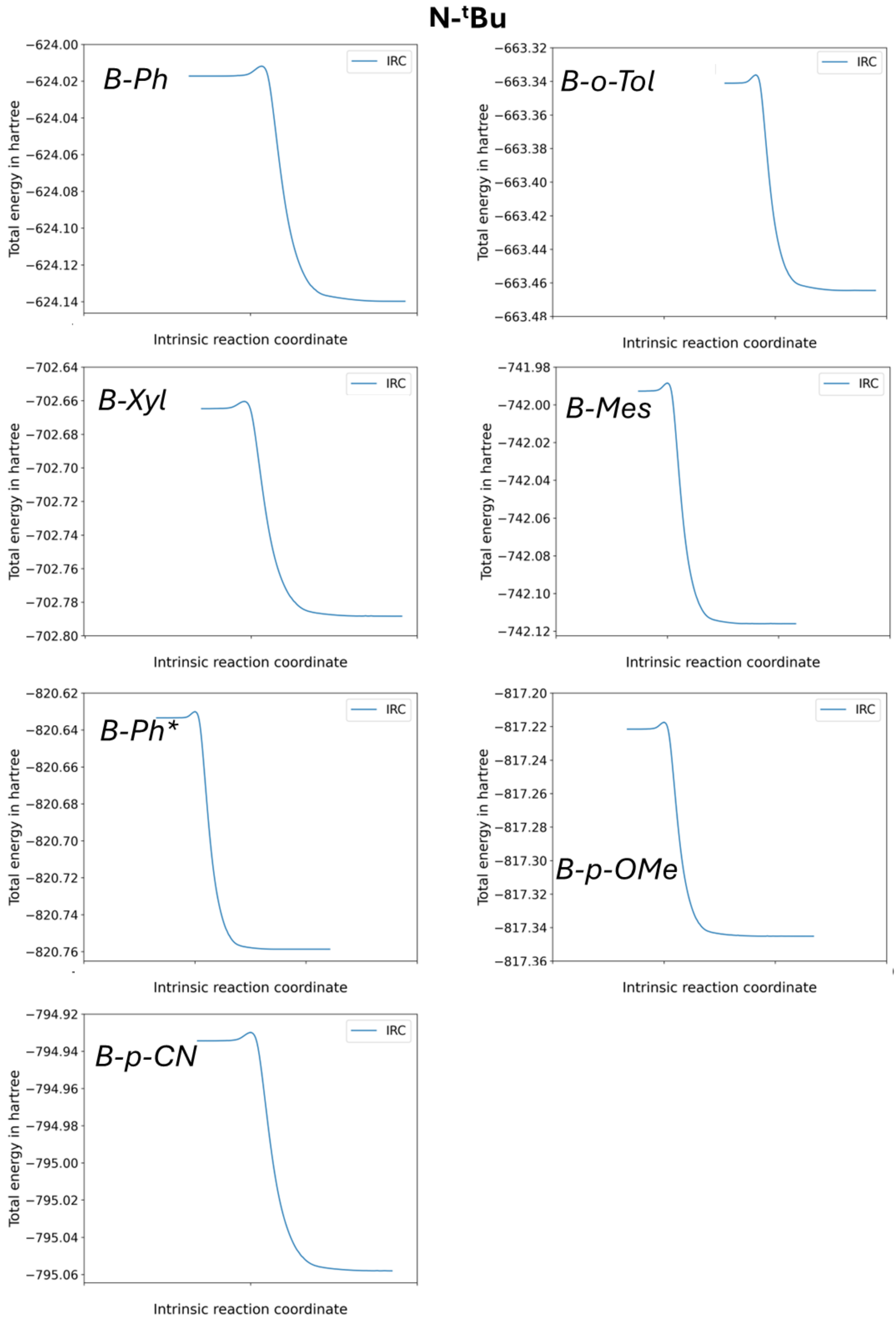

Figure S125. Intrinsic reaction coordinate of the thermal ring opening of the N-tBu substituted Dihydroazaborinines. All energies are given in Hartree.

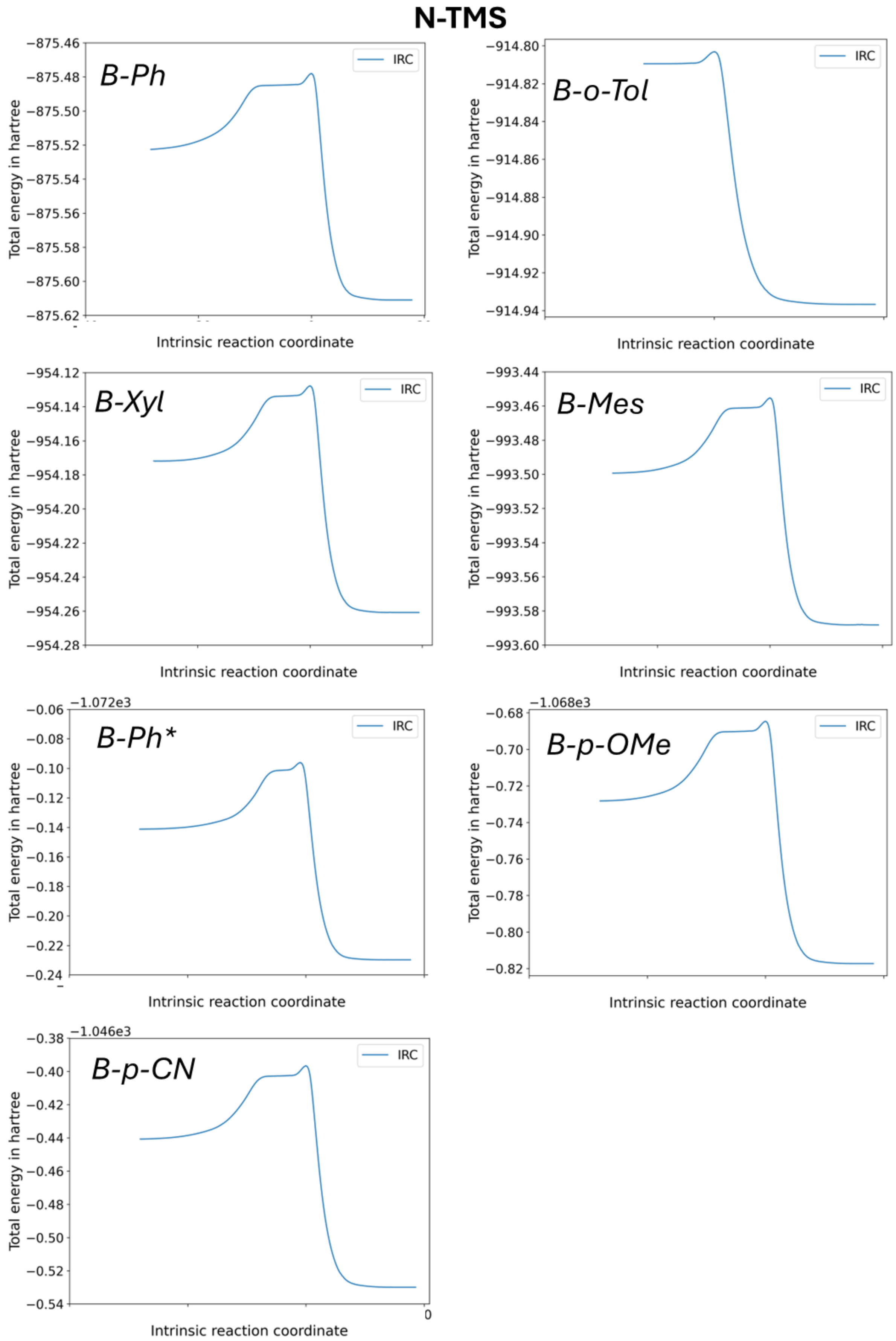

Figure S126. Intrinsic reaction coordinate of the thermal ring opening of the N-TMS substituted Dihydroazaborinines. All energies are given in Hartree.

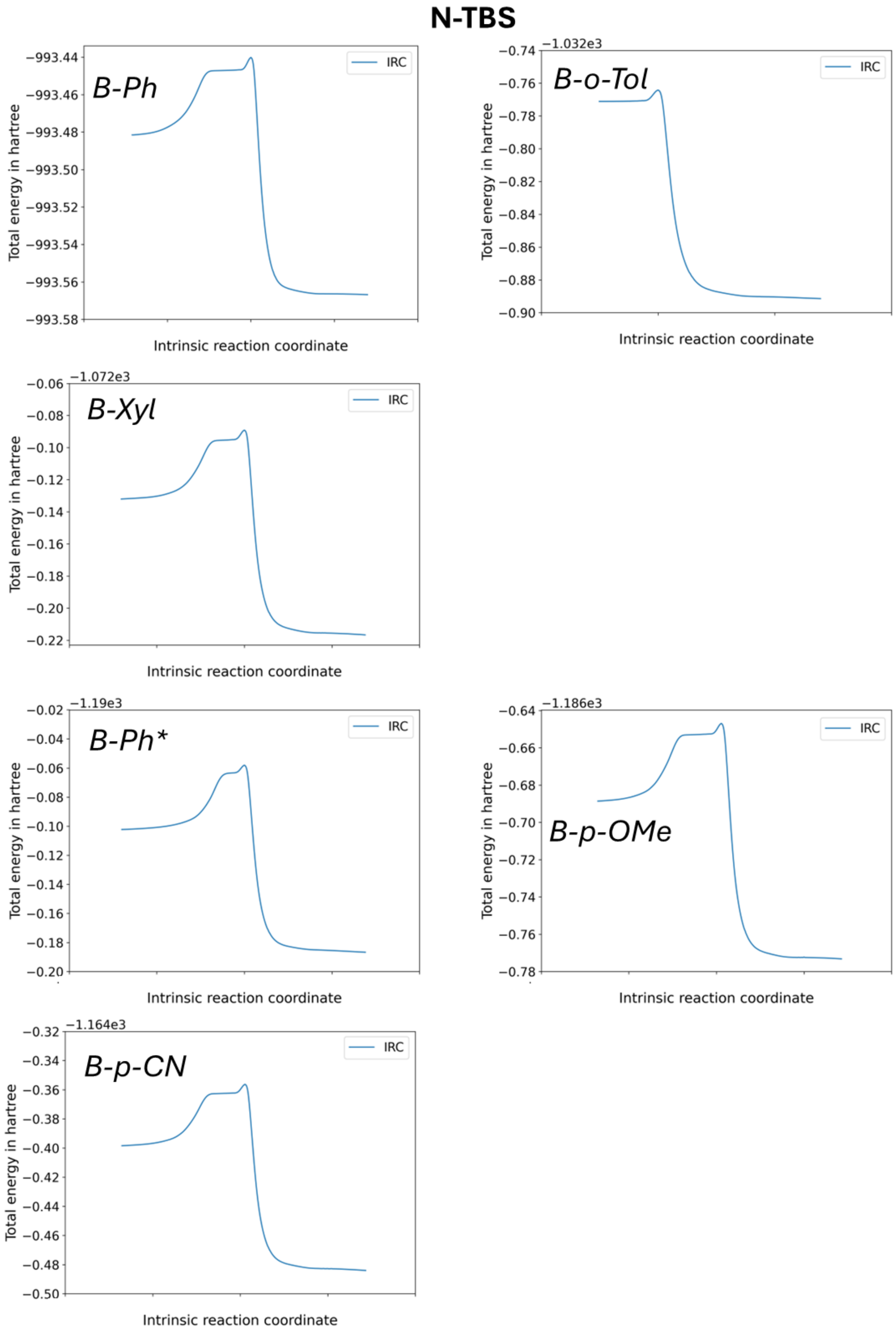

Figure S127. Intrinsic reaction coordinate of the thermal ring opening of the N-TBS substituted Dihydroazaborinines. All energies are given in Hartree.

8. References

(1) Sheldrick, G. M. A short history of SHELX. *Acta Cryst.* **2008**, *A64*, 112-122. DOI: https://doi.org/10.1107/S0108767307043930.
(2) Hübschle, C. B.; Sheldrick, G. M.; Dittrich, B. ShelXle: a Qt graphical user interface for SHELXL. *J. Appl. Cryst.* **2011**, *44* (6), 1281-1284. DOI: doi:10.1107/S0021889811043202.
(3) Sheldrick, G. Crystal structure refinement with SHELXL. *Acta Cryst.* **2015**, *C71* (1), 3-8. DOI: doi:10.1107/S2053229614024218.
(4) Dolomanov, O. V.; Bourhis, L. J.; Gildea, R. J.; Howard, J. A. K.; Puschmann, H. OLEX2: a complete structure solution, refinement and analysis program. *J. Appl. Crystallogr.* **2009**, *42* (2), 339-341. DOI: doi:10.1107/S0021889808042726.
(5) Bourhis, L. J.; Dolomanov, O. V.; Gildea, R. J.; Howard, J. A. K.; Puschmann, H. The anatomy of a comprehensive constrained, restrained refinement program for the modern computing environment - Olex2 dissected. *Acta Crystallographica Section A* **2015**, *71* (1), 59-75. DOI: doi:10.1107/S2053273314022207.
(6) Richter, R. C.; Biebl, S. M.; Einholz, R.; Walz, J.; Maichle-Mössmer, C.; Ströbele, M.; Bettinger, H. F.; Fleischer, I. Facile Energy Release from Substituted Dewar Isomers of 1,2-Dihydro-1,2-Azaborinines Catalyzed by Coinage Metal Lewis Acids. *Angew. Chem. Int. Ed.* **2024**, *63* (30), e202405818. DOI: https://doi.org/10.1002/anie.202405818.
(7) Marwitz, A. J. V.; Matus, M. H.; Zakharov, L. N.; Dixon, D. A.; Liu, S.-Y. A Hybrid Organic/Inorganic Benzene. *Angew. Chem. Int. Ed.* **2009**, *48* (5), 973-977. DOI: 10.1002/ange.200805554.
(8) Lamm, A. N.; Liu, S.-Y. How stable are 1,2-dihydro-1,2-azaborines toward water and oxygen? *Mol. BioSyst.* **2009**, *5* (11), 1303-1305. DOI: 10.1039/b904120f.
(9) Lee, H.; Alvarado, M.; Ingram, S.; Li, B.; Liu, S.-Y. N-Functionalization of 1,2-Azaborines. *Synlett* **2023**, *34* (18), 2169-2174. DOI: 10.1055/a-2108-9895.
(10) Dupont, J.; Beydoun, N.; Pfeffer, M. Reactions of cyclopalladated compounds. Part 21. Various examples of sulphur-assisted intramolecular palladation of aryl and alkyl groups. *J. Chem. Soc., Dalton Trans.* **1989**, *18* (9), 1715-1720. DOI: 10.1039/DT9890001715.
(11) Edel, K.; Yang, X.; Ishibashi, J. S. A.; Lamm, A. N.; Maichle-Mössmer, C.; Giustra Zachary, X.; Liu, S. Y.; Bettinger, H. F. The Dewar Isomer of 1,2-Dihydro-1,2-azaborinines: Isolation, Fragmentation, and Energy Storage. *Angew. Chem. Int. Ed.* **2018**, *57* (19), 5296-5300. DOI: 10.1002/anie.201712683.
(12) Baggett, A. W.; Vasiliu, M.; Li, B.; Dixon, D. A.; Liu, S.-Y. Late-Stage Functionalization of 1,2-Dihydro-1,2-azaborines via Regioselective Iridium-Catalyzed C-H Borylation: The Development of a New N,N-Bidentate Ligand Scaffold. *J. Am. Chem. Soc.* **2015**, *137* (16), 5536-5541. DOI: 10.1021/jacs.5b01916.
(13) Chen, M.; Unikela, K. S.; Ramalakshmi, R.; Li, B.; Darrigan, C.; Chrostowska, A.; Liu, S.-Y. A BN-Doped Cycloparaphenylene Debuts. *Angew. Chem. Int. Ed.* **2021**, *60* (3), 1556-1560. DOI: https://doi.org/10.1002/anie.202010556.
(14) Biebl, S. M.; Lienert, J. N.; Müller, A. J.; Ströbele, M.; Dreuw, A.; Wachtveitl, J.; Bettinger, H. F. Switching Shapes: Reversible Three Species Photoisomerization of Substituted 1,2-Dihydro-1,2-azaborinines. *J. Am. Chem. Soc.* **2026**, *148* (10), 10776-10787. DOI: 10.1021/jacs.5c20667.
(15) Biebl, S. M.; Richter, R. C.; Ströbele, M.; Fleischer, I.; Bettinger, H. F. High energy density dihydroazaborinine dyads and triad for molecular solar thermal energy storage. *Chem. Sci.* **2025**, *16* (33), 15231-15238. DOI: 10.1039/D5SC03159A.
(16) Biebl, S. M.; Ziemann, P.; Ströbele, M.; Bettinger, H. F. Mechanistic Insight into the Thermal Ring Opening of the Dewar Isomer of 1,2-Dihydro-1,2-azaborinines. *JACS Au* **2025**, *5* (10), 5006-5016. DOI: 10.1021/jacsau.5c00923.
(17) Kleemiss, F.; Dolomanov, O. V.; Bodensteiner, M.; Peyerimhoff, N.; Midgley, L.; Bourhis, L. J.; Genoni, A.; Malaspina, L. A.; Jayatilaka, D.; Spencer, J. L.; et al. Accurate crystal structures and chemical properties from NoSpherA2. *Chem. Sci.* **2021**, *12* (5), 1675-1692. DOI: 10.1039/d0sc05526c.
(18) Becke, A. D. Density-functional thermochemistry. III. The role of exact exchange *J. Chem. Phys.* **1993**, *98*, 5648-5652. DOI: 10.1063/1.464913.

(19) Lee, C.; Yang, W.; Parr, R. G. Development of the Colle-Salvetti correlation-energy formula into a functional of the electron density. *Phys. Rev. B* **1988**, *37*, 785-789. DOI: https://doi.org/10.1103/PhysRevB.37.785.
(20) Ditchfield, R.; Hehre, W. J.; Pople, J. A. Self-Consistent Molecular-Orbital Methods. IX. An Extended Gaussian-Type Basis for Molecular-Orbital Studies of Organic Molecules. *J. Chem. Phys.* **1971**, *54* (2), 724-728. DOI: 10.1063/1.1674902.
(21) Hehre, W. J.; Ditchfield, R.; Pople, J. A. Self---Consistent Molecular Orbital Methods. XII. Further Extensions of Gaussian---Type Basis Sets for Use in Molecular Orbital Studies of Organic Molecules. *J. Chem. Phys.* **1972**, *56* (5), 2257-2261. DOI: 10.1063/1.1677527.
(22) Dill, J. D.; Pople, J. A. Self-consistent molecular orbital methods. XV. Extended Gaussian-type basis sets for lithium, beryllium, and boron. *J. Chem. Phys.* **1975**, *62* (7), 2921-2923. DOI: 10.1063/1.430801.
(23) Hariharan, P. C.; Pople, J. A. The Influence of Polarization Functions on Molecular Orbital Hydrogenation Energies. *Theor. Chim. Acta* **1973**, *28*, 213-222. DOI: 10.1007/BF00533485.
(24) Neese, F. Software update: The ORCA program system—Version 5.0. *WIREs Computational Molecular Science* **2022**, *12* (5), e1606. DOI: https://doi.org/10.1002/wcms.1606.
(25) Neese, F. The ORCA program system. *WIREs Comput. Mol. Sci.* **2012**, *2* (1), 73-78. DOI: 10.1002/wcms.81.
(26) Stephens, P. J.; Devlin, F. J.; Chabalowski, C. F.; Frisch, M. J. Ab Initio Calculation of Vibrational Absorption and Circular Dichroism Spectra Using Density Functional Force Fields. *J. Phys. Chem.* **1994**, *98* (45), 11623-11627. DOI: https://doi.org/10.1021/j100096a001.
(27) Krishnan, R.; Binkley, J. S.; Seeger, R.; Pople, J. A. Self-consistent molecular orbital methods. XX. A basis set for correlated wave functions. *J. Chem. Phys.* **1980**, *72* (1), 650-654. DOI: 10.1063/1.438955.
(28) Clark, T.; Chandrasekhar, J.; Spitznagel, G. W.; Schleyer, P. V. R. Efficient diffuse function-augmented basis sets for anion calculations. III. The 3-21+G basis set for first-row elements, Li–F. *J. Comput. Chem.* **1983**, *4* (3), 294-301. DOI: https://doi.org/10.1002/jcc.540040303.
(29) McLean, A. D.; Chandler, G. S. Contracted Gaussian basis sets for molecular calculations. I. Second row atoms, Z=11–18. *J. Chem. Phys.* **1980**, *72* (10), 5639-5648. DOI: 10.1063/1.438980.
(30) Francl, M. M.; Pietro, W. J.; Hehre, W. J.; Binkley, J. S.; Gordon, M. S.; DeFrees, D. J.; Pople, J. A. Self-consistent molecular orbital methods. XXIII. A polarization-type basis set for second-row elements. *J. Chem. Phys.* **1982**, *77* (7), 3654-3665. DOI: 10.1063/1.444267.
(31) Spitznagel, G. W.; Clark, T.; von Ragué Schleyer, P.; Hehre, W. J. An evaluation of the performance of diffuse function-augmented basis sets for second row elements, Na-Cl. *J. Comput. Chem.* **1987**, *8* (8), 1109-1116. DOI: https://doi.org/10.1002/jcc.540080807.
(32) *Gaussian 16 Rev. C.01*; Frisch, M. J.; Trucks, G. W.; Schlegel, H. B.; Scuseria, G. E.; Robb, M. A.; Cheeseman, J. R.; Scalmani, G.; Barone, V.; Petersson, G. A.; Nakatsuji, H.; et al.: Wallingford, CT, 2016.
(33) Page, M.; McIver, J. W., Jr. On evaluating the reaction path Hamiltonian. *J. Chem. Phys.* **1988**, *88* (2), 922-935. DOI: 10.1063/1.454172.
(34) Page, M.; Doubleday, C.; McIver, J. W., Jr. Following steepest descent reaction paths. The use of higher energy derivatives with ab initio electronic structure methods. *J. Chem. Phys.* **1990**, *93* (8), 5634-5642. DOI: 10.1063/1.459634.
(35) Bettinger, H. F.; Hauler, O. Ring opening of 2-aza-3-borabicyclo[2.2.0]hex-5-ene, the Dewar form of 1,2-dihydro-1,2-azaborine: stepwise versus concerted mechanisms. *Beilstein J. Org. Chem.* **2013**, *9*, 761-766. DOI: 10.3762/bjoc.9.86.
